# Three‐Component Radical Cross‐Coupling: Asymmetric Vicinal Sulfonyl‐Esterification of Alkenes Involving Sulfur Dioxide

**DOI:** 10.1002/advs.202309069

**Published:** 2024-03-26

**Authors:** Zhiqian Chang, Xuemei Zhang, Haiping Lv, Haotian Sun, Zhong Lian

**Affiliations:** ^1^ Department of Dermatology State Key Laboratory of Biotherapy and Cancer Center West China Hospital Sichuan University Chengdu 610041 P. R. China

**Keywords:** asymmetric catalysis, copper catalysis, radical cross‐coupling, sulfonyl‐esterification of alkenes, sulfur dioxide

## Abstract

A novel catalytic system for radical cross‐coupling reactions based on copper and chiral Pyridyl‐bis(imidazole) (PyBim) ligands is described. It overcomes the challenges of chemoselectivity and enantioselectivity, achieving a highly enantioselective vicinal sulfonyl‐esterification reaction of alkenes involving sulfur dioxide. This strategy involves the use of earth‐abundant metal catalyst, mild reaction conditions, a broad range of substrates (84 examples), high yields (up to 97% yield), and exceptional control over enantioselectivity. The reaction system is compatible with different types of radical precursors, including *O*‐acylhydroxylamines, cycloketone oxime esters, aryldiazonium salts, and drug molecules. Chiral ligand PyBim is identified as particularly effective in achieving the desired high enantioselectivity. Mechanistic studies reveal that copper/PyBim system plays a vital role in C─O coupling, employing an outer‐sphere model. In addition, the side arm effect of ligand is observed.

## Graphical abstract

A novel catalytic system based on copper and chiral PyBim ligands is described for highly enantioselective vicinal sulfonyl‐esterification reaction of alkenes involving sulfur dioxide. This protocol overcomes challenges of chemoselectivity and enantioselectivity, with mild conditions, a broad range of substrates, high yields, and exceptional control over enantioselectivity.

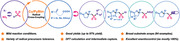

## 1 Introduction

**Figure 1:**
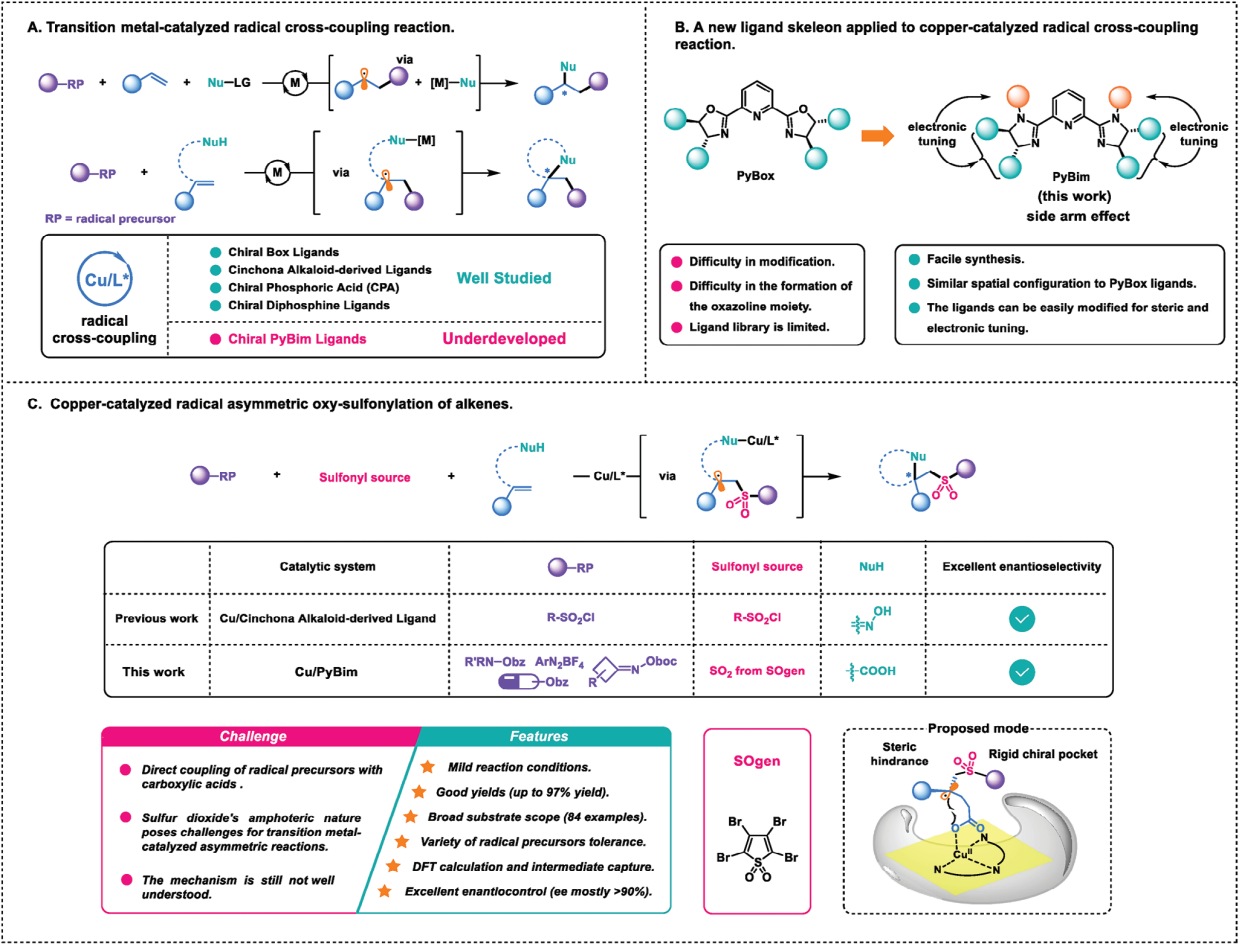
Overviews copper‐catalyzed radical cross‐coupling.

Transition metal‐catalyzed radical cross‐coupling reactions present extensive opportunities for the synthesis of intricate organic molecules.^[^ ^1^, ^2^, ^3^, ^4^ ^]^ Among the first‐row transition metals, copper, which is abundant on earth, has attracted significant attention due to its widespread application in radical cross‐coupling reactions.^[^ ^4^ ^]^ Additionally, copper‐catalyzed enantioconvergent radical cross‐coupling reactions are commonly accomplished by employing chiral ligands such as chiral bidentate oxazoline (Box) ligands,^[^ ^5^ ^]^ cinchona alkaloid‐derived ligands,^[^ ^6^ ^]^ chiral phosphoric acids,^[^ ^7^ ^]^ and chiral diphosphine ligands.^[^ ^8^ ^]^ To the best of our knowledge, the PyBim ligand has not been applied to copper‐catalyzed radical cross‐coupling reactions (**Figure** **1**A).

Chiral Pyridine‐Bis(Oxazoline) (PyBox) ligands, despite their distinct advantages in various reactions, face significant challenges in the modification of the ligand skeleton and synthesis of the oxazoline moiety, which prolong and complicate the creation of ligand libraries.^[^ ^9^ ^]^ In contrast, PyBim, bearing a similar spatial configuration to PyBox, offers a more versatile modification space that can be further adjusted electronically and conformationally across a wide range.^[^ ^9^, ^10^ ^]^ As a result, the Cu/PyBim system has the potential to complement radical cross‐coupling reactions, displaying distinct properties (Figure 1B). [Supplementary-material advs7592-supitem-0001]

Due to the high reactivity and short lifespan of radical intermediates compared to their ionic counterparts, there has been limited research on the copper‐catalyzed radical asymmetric oxy‐sulfonylation of alkenes. Recently, the Liu group achieved a significant advancement by developing the first asymmetric radical oxy‐sulfonylation of alkenes, utilizing oximes and sulfonyl chloride as the sources of oxy and sulfonyl, respectively (Figure 1C).^[^ ^6a^ ^]^ This groundbreaking achievement was made possible through the use of a novel catalyst, a combination of copper(I) and cinchona alkaloid‐derived sulfonamide ligand. Despite this significant breakthrough, the development of a universal strategy for the high enantioselective vicinal sulfonyl‐esterification of alkenes still poses several challenges:^[^ ^11^ ^]^ 1) In the multicomponent reaction, the radical precursor might couple with carboxylic acids directly;^[^ ^5^, ^7^, ^12^ ^]^ 2) Sulfur dioxide's versatility as an amphoteric ligand presents challenges for transition metal‐catalyzed asymmetric reactions involving its use;^[^ ^11^, ^13^ ^]^ 3) The mechanism for the asymmetric vicinal sulfonyl‐esterification of alkenes is still not well understood (Figure 1C, bottom).

In this context, as part of our ongoing efforts on sulfur dioxide insertion reactions, we developed a general and efficient method to achieve asymmetric vicinal sulfonyl‐esterification of alkenes utilizing Cu/PyBim system.^[^ ^14^ ^]^ The method encompasses the use of an earth‐abundant metal catalyst, along with mild reaction conditions, a wide range of substrates, and excellent control over enantioselectivity. Notably, this method can be applied to different types of radical precursors, including *O*‐acylhydroxylamines, cycloketone oxime esters, aryldiazonium salts, and drug molecules. Mechanism studies revealed that asymmetric coupling of C─O bonds was successfully achieved within the rigid chiral space formed by the tridentate non‐scorpionate ligand PyBim and copper salts, employing an outer‐sphere model.

## 2 Results and Discussion

Initially, we probed the reaction conditions using carboxylic acid **1a** and morpholino benzoate **2a** as the reaction partners (**Table** **1**). After screening various reaction parameters, the optimal conditions involve using Cu(MeCN)_4_PF_6_ as the catalyst, **L1** as the ligand, Na_2_CO_3_ as the base, 4Å molecular sieve (MS) as the additive, SOgen as the SO_2_ surrogate, and 2‐Me‐THF as the solvent. Stirring the mixture at room temperature for 12 h under argon, the desired chiral sulfonyl lactone (**3a**) was obtained in 93% isolated yield and 96% ee (entry 1). Investigation of ligands, including PyBim (**L1**‐**L3**), PyBox (**L4**‐**L5**), and Box (**L6**‐**L8**) (entries 1–8), revealed that PyBox ligand **L4** demonstrated higher enantioselectivity compared to the Box ligand. Furthermore, PyBim was identified as the most effective ligand for achieving exceptional enantioselectivity. Different solvents were tested, and 2‐Me‐THF was identified as the best one (entries 9–12). 4Å MS and Na_2_CO_3_ were found not to be necessary for maintaining great enantioselectivity, but they were beneficial for improving the yield (entries 13 and 14). In terms of the base, K_2_CO_3_ and Cs_2_CO_3_ were not as effective as Na_2_CO_3_ (entries 15 and 16). When using DABSO as the SO_2_ surrogate, the product was obtained in a much lower yield with moderate ee, and no product was obtained using either Na_2_S_2_O_5_ or K_2_S_2_O_5_ (entries 17–19). These results clearly indicated that the use of SOgen was essential for the successful transformation. When morpholine‐4‐sulfonyl chloride was used as the source of the sulfonyl group, the desired product could be obtained in a low yield (entry 20).

**Table 1:** Optimizations of reaction conditions.

Entry^a)^	Change from standard conditions	Yield^b)^	ee^c)^
1	none	93%	96%
2	**L2** instead of **L1**	81%	96%
3	**L3** instead of **L1**	30%	94%
4	**L4** instead of **L1**	83%	75%
5	**L5** instead of **L1**	78%	−23%
6	**L6** instead of **L1**	56%	−69%
7	**L7** instead of **L1**	54%	7%
8	**L8** instead of **L1**	18%	0%
9	CH_2_Cl_2_ instead of 2‐Me‐THF	76%	95%
10	MeCN instead of 2‐Me‐THF	16%	75%
11	THF instead of 2‐Me‐THF	59%	89%
12	Dioxane instead of 2‐Me‐THF	24%	66%
13	No 4Å MS added	65%	97%
14	No Na_2_CO_3_ added	75%	98%
15	K_2_CO_3_ instead of Na_2_CO_3_	77%	97%
16	Cs_2_CO_3_ instead of Na_2_CO_3_	52%	82%
17^e)^	DABSO as SO_2_ source	12%	63%
18^e)^	Na_2_S_2_O_5_ as SO_2_ source	N.D.^d)^	‐
19^e)^	K_2_S_2_O_5_ as SO_2_ source	N.D.^d)^	‐
20^e)^	Morpholine‐4‐sulfonyl chloride as sulfonyl precursor	32%	97%

^a)^ Reaction conditions. Chamber A: SOgen (0.31 mmol), 1‐methyl‐4‐vinylbenzene (0.30 mmol), tetradecane (1.0 mL), at 100 °C for 10 min. Chamber B: **1a** (0.2 mmol), **2a** (0.6 mmol, 3 equiv), Cu(MeCN)_4_PF_6_ (0.02 mmol, 10 mol%), **L1** (0.024 mmol, 12 mol%), Na_2_CO_3_ (0.22 mmol, 1.1 equiv), 4Å Molecular Sieve (60 mg), 2‐Me‐THF (2.0 mL) at room temperature for 12 h under argon atmosphere.

^b)^ Isolated yield of **3a;**

^c)^ The ee value of **3a** was determined by HPLC analysis on a chiral stationary phase

^d)^ ND: Not Detected;

^e)^ The reaction was set up in an 8 mL vial.

**Scheme 1:**
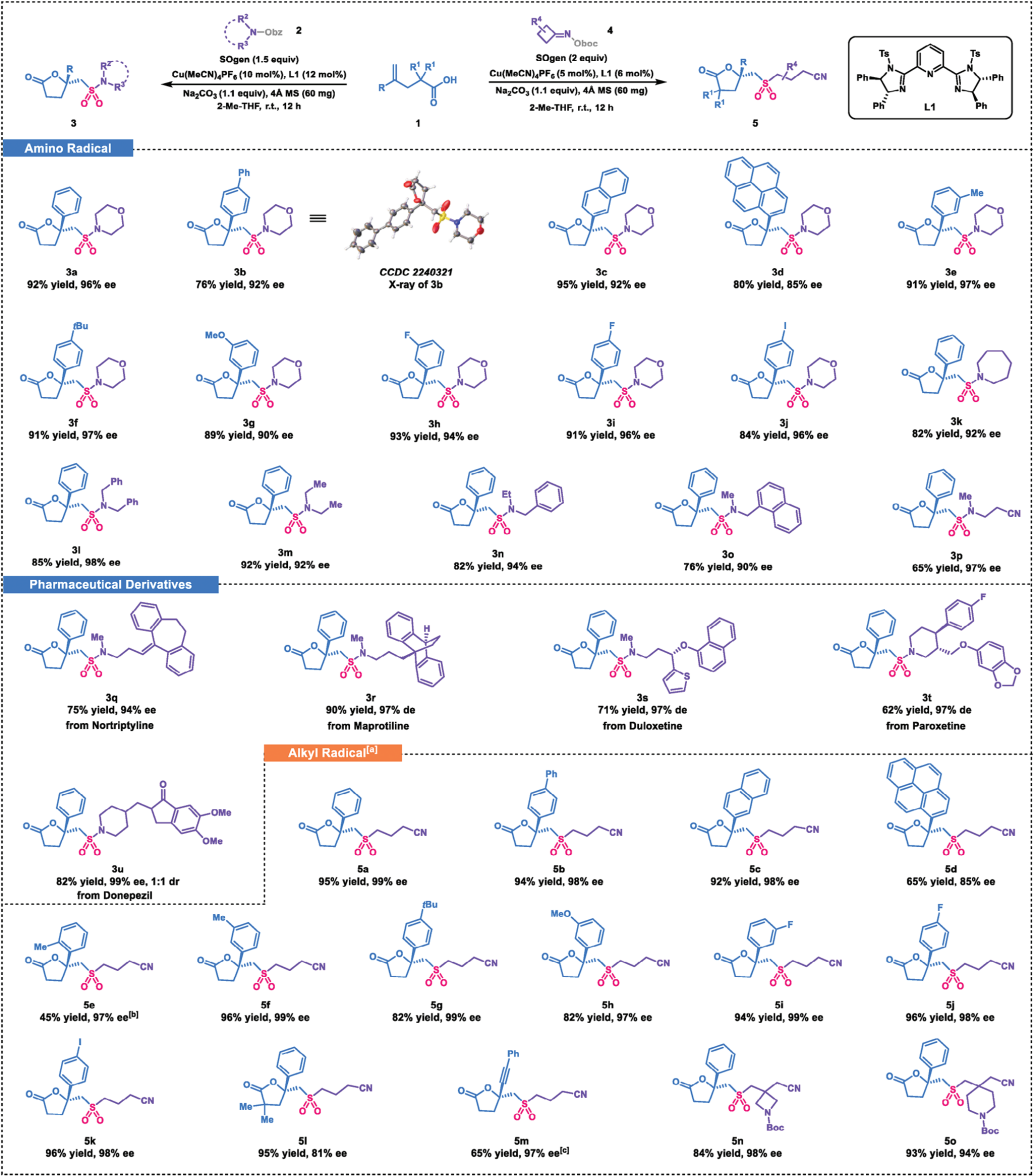
Substrate scope. a) SOgen (0.41 mmol), 1‐methyl‐4‐vinylbenzene (0.40 mmol), cycloketone oxime esters **4** (0.4 mmol, 2 equiv), Cu(MeCN)_4_PF_6_ (0.01 mmol, 5 mol %), **L1** (0.012 mmol, 6 mol %) were used. b) 24 h. c) 36 h.

Having established the optimized reaction conditions, we first examined the scope of the transformation with respect to the carboxylic acids **1** (**Scheme** **1**). Carboxylic acids with electron‐neutral substitutions yielded the corresponding vicinal sulfonyl‐esterification products (**3b‐3d**) with moderate to good yields and high enantioselectivities. The absolute configuration of **3b** was confirmed through X‐ray analysis.^[^ ^15^ ^]^ Furthermore, carboxylic acids carrying both electron‐donating and electron‐withdrawing substituents were found to be compatible with this transformation (**3e‐3j**). Next, we examined various electrophilic amines **2**, and observed that the optimal reaction conditions could accommodate a derivative containing a seven‐membered ring (**3k**). Additionally, different acyclic substrates provided the desired products with good yields and excellent enantioselectivities (**3l‐3p**). Notably, several commercially available blockbuster drugs underwent the reaction successfully (**3q‐3u**). This strategy enabled the modification of antidepressants such as *Nortriptyline*, *Maprotiline*, *Duloxetine*, and *Paroxetine*, as well as the anti‐Alzheimer's drug *Donepezil*.

Subsequently we proceeded to investigate the asymmetric vicinal sulfonyl‐esterification of alkenes using alternative radical species (Scheme 1, bottom). Encouragingly, we observed that cycloketone oxime esters **4** and carboxylic acid **1** were also compatible with the current reaction system, albeit with slightly modified conditions. We then focused on examining the impact of substituents on reactant **1** on the reaction outcome. Electron‐neutral substitutions on substrates led to the formation of desired products (**5a**‐**5d**) with excellent enantioselectivities (up to 99% ee). Monosubstituted 4‐phenylpenta‐4‐enoic acids, with methyl or *tert*‐butyl at *para*, *meta* and *ortho* positions, yielded products (**5e**‐**5 g**) with moderate to good yields, indicating that steric bulk influenced the reaction efficiency. Furthermore, electron‐withdrawing substituents such as *m*‐OMe group and halogens were well‐tolerated (**5h**‐**5k**). Notably, the *gem*‐dimethyl carboxylic acid was compatible with the reaction conditions, affording the product (**5l**) with excellent yield but slightly decreased enantioselectivity. In addition, the substrate containing an alkyne moiety underwent 1,2‐addition to yield the five‐membered ring product (**5m**). Subsequent evaluation of two other cycloketone oxime esters revealed their effectiveness in the transformation, yielding products **5n** and **5o** with excellent enantioselectivities.

**Scheme 2:**
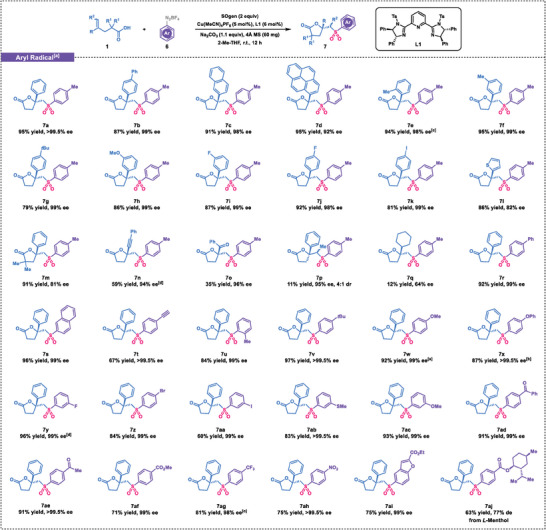
Substrate scope. a) SOgen (0.41 mmol), 1‐methyl‐4‐vinylbenzene (0.40 mmol), aryldiazonium salt **6** (0.4 mmol, 2 equiv), Cu(MeCN)_4_PF_6_ (0.01 mmol, 5 mol %), **L1** (0.012 mmol, 6 mol %) were used. b) 18 h. c) 24 h. d) 36 h. e) 48 h.

Encouraged by these promising results, we sought to expand the scope of radical species to the aryl radicals for the asymmetric vicinal sulfonyl‐esterification of alkenes. To our satisfaction, the reaction between aryldiazonium salts **6** and carboxylic acid **1** proceeded smoothly, yielding the desired products (**Scheme** **2**). The catalytic system displayed excellent functional group tolerance, as carboxylic acids with various substitutions all performed well, providing the corresponding products (**7a**‐**7k**) in good to excellent yields and high enantioselectivities.^[^ ^16^ ^]^ Substrates containing thiofuran or *gem*‐ dimethyl groups showed a slight decrease in chiral selectivity, resulting in products (**7l** and **7m**) with 81% and 82% ee, respectively. Notably, reactant **1** lacking styrene moiety could be tolerated under the standard reaction conditions, resulting in products with high enantioselectivity (**7n** and **7o**), albeit with diminished yields. We examined the utilization of internal olefin as a substrate under standard conditions. Although the yield was relatively low, we were able to obtain product **7p** with a 95% ee and a 4:1 dr. Furthermore, we also investigated the reactivity of non‐activated olefins as substrates under standard conditions, which resulted in a lower yield and moderate enantioselectivity in the formation of product **7q**.

Furthermore, we investigated arange of substituted aryldiazonium salts in the transformation (Scheme 2), and the optimized conditions proved to be applicable to biphenyl and naphthoyl diazonium salts as well (**7r** and **7s**). Importantly, substrates containing terminal alkynes were amenable to the reaction and afforded the desired products (**7t**) in moderate yield but exceptional enantioselectivity. Aryldiazonium salts substituted with electron‐donating groups, such as methyl (**7u**), *tert*‐butyl (**7v**), 4‐methoxy (**7w**), and phenoxy (**7x**), were also suitable reactants. In addition, halide‐substituted aryldiazonium salts were well‐tolerated (**7y**‐**7aa**), opening up possibilities for further conversions through cross‐coupling reactions. Substrates with electron‐deficient substituents, such as *meta*‐methylthio (**7ab**), *meta*‐methoxy (**7ac**), benzoyl (**7ad**), formyl (**7ae**), and ester (**7af**) groups, also underwent the transformation efficiently. Furthermore, substrates with strongly electron‐withdrawing trifluoromethyl and nitro groups (**7ag**‐**7ah**) were both compatible with this strategy. Finally, a heteroaromatic diazonium salt proved to be an effective radical precursor, providing the corresponding products (**7ai**) in an acceptable yield and high enantioselectivity. Of particular interest, even a substrate derived from l‐menthol demonstrated good reactivity under the current conditions (**7aj**), albeit with a slightly lower enantiomeric excess.

**Figure 2:**
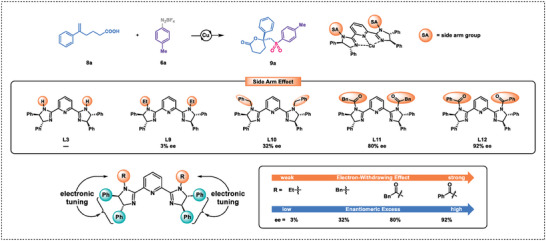
Influence of side arm on the asymmetric synthesis of six‐membered lactones.

**Scheme 3:**
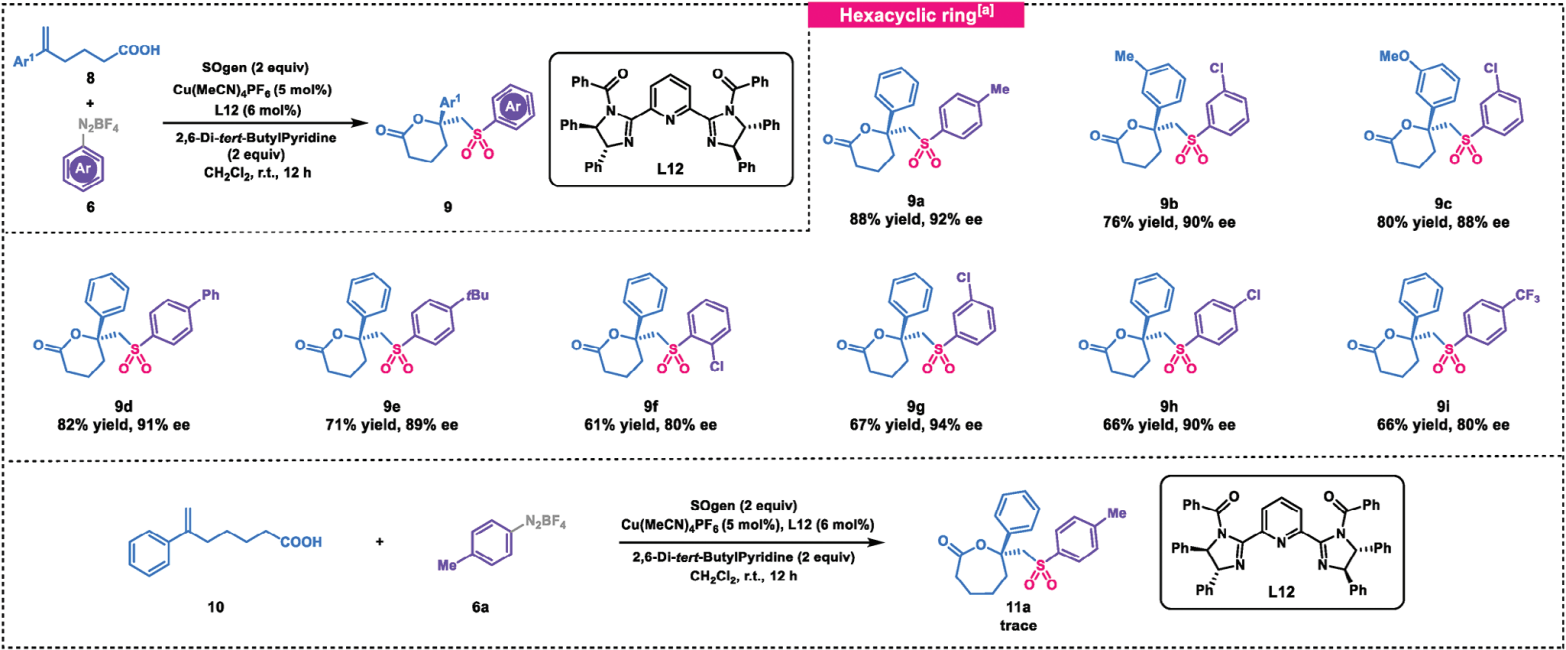
Substrate scope. a) SOgen (0.41 mmol), 1‐methyl‐4‐vinylbenzene (0.40 mmol), aryldiazonium salt **6** (0.4 mmol, 2 equiv), Cu(MeCN)_4_PF_6_ (0.01 mmol, 5 mol %), **L12** (0.012 mmol, 6 mol %), 2,6‐di‐*tert*‐ButylPyridine (0.4 mmol, 2 equiv), CH_2_Cl_2_ (2.0 mL) were used.

Next, we turned our attention to evaluating the range of substrates that could be utilized for the synthesis of six‐membered lactones from aryldiazonium salts **6** and 5‐hexenoic acid **8** in the presence of a copper catalyst. As shown in **Figure** **2**, a strong side arm effect was observed in this reaction, highlighting the essential role of Pybim's side arms in promoting high levels of enantioselectivity.^[^ ^17^ ^]^ In the presence of a hydrogen side arm, PyBim yielded only trace amount of six‐membered lactone **9a**. When the side arm was ethyl or benzyl, **9a** with low enantioselectivity was obtained. Intriguingly, upon employing benzoyl or benzylcarbonyl as the side arm, a substantial enhancement in enantioselectivity of **9a** was observed. Overall, the results indicated that the enantioselectivity of the reaction progressively improved with the enhanced electron‐withdrawing effect of the PyBim ligand's side arm. Based on the aforementioned experiments and literature reports,^[^ ^9^, ^10^ ^]^ it was observed that PyBim had the capability to modulate the enantioselectivity of a reaction by manipulating the electronic effect of the ligand through modification of the side arm. As shown in **Scheme** **3**, a wide range of substitutions on the aromatic rings of reactants **6** and **8** were compatible with the established reaction, leading to the formation of products **9a**‐**9i** in moderate to high yields and high enantioselectivities. We attempted to synthesize the seven‐membered ring product **11a**. Unfortunately, only trace amounts of the desired product were obtained.

**Scheme 4:**
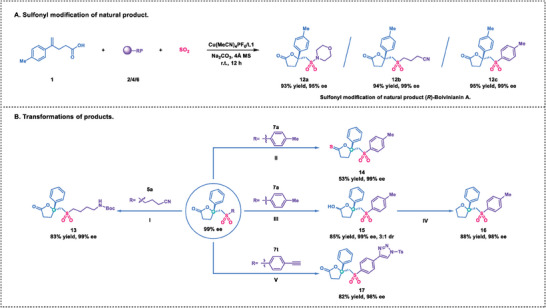
Synthetic applications. Reaction conditions: (I) NiCl_2_·6H_2_O (3.0 equiv), (Boc)_2_O (3.0 equiv), NaBH_4_ (10 equiv), MeOH, r.t., 12 h; (II) Lawesson reagent (2.25 equiv), toluene, reflux. (III) DIBAl‐H (1.1 equiv), CH_2_Cl_2_, −78°C, 1 h. (IV) Et_3_SiH (5 equiv), BF_3_·Et_2_O (4 equiv), CH_2_Cl_2_, r.t., 3 h. (V) TsN_3_ (1.1 equiv), CuTc (10 mol%), toluene, r.t., 12 h.

To demonstrate the practicality of our strategy for the asymmetric vicinal sulfonyl‐esterification of alkenes, we conducted several synthetic applications (**Scheme** **4**). Using this method, we successfully achieved the sulfonyl modification of the natural product (*R*)‐Bovinianin A in a single step (**12a**‐**12c**).^[^ ^18^ ^]^

Furthermore, the chiral sulfonyl lactones produced through this process could be readily converted into other valuable compounds with different functional groups. For instance, treatment of **5a** with nickel‐mediated reduction yielded Boc‐protected amine **13** in 83% yield with 99% ee. The reaction of **7a** with Lawesson reagent furnished the thiolated product **14** with enantioselective preservation. Additionally, the carbonyl group of **7a** could be selectively reduced to form hemiacetal **15**, which could then be further reduced to produce compound **16**. Likewise, compound **7t** underwent a copper‐catalyzed click reaction, leading to the formation of triazole compound **17**.

**Figure 3:**
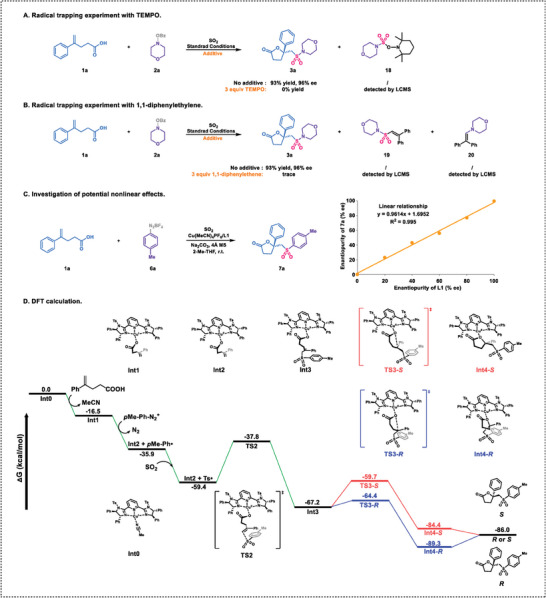
Mechanistic studies.

In order to gain a better understanding of the mechanism, a series of experiments were conducted to gain a comprehensive understanding of the mechanism (**Figure** **3**). First, when 3.0 equiv. TEMPO was added under the standard conditions, the desired product **3a** was not detected, but the TEMPO‐adduct **18** was observed (Figure 3A). Second, the addition of 3.0 equiv. 1,1‐diphenylethylene did not lead to the formation of the desired product **3a**. Instead, compounds **19** and **20**, derived from nitrogen radicals and sulfonyl radicals, were detected (Figure 3B). These results strongly suggested a radical process in the reaction. Additionally, we observed a linear correlation between the enantiopurity of product **7a** and the enantiopurity of ligand **L1**, indicating that a single chiral ligand and a copper complex were involved in the enantiocontrol step (Figure 3C).^[^ ^19^ ^]^

To get more information of the mechanism, we conducted density functional theory (DFT) calculations (for more details, refer to the Supporting Information)(Figure 3D).^[^ ^20^ ^]^ The catalyst Cu(MeCN)_4_PF_6_ undergoes ligand exchange with **L1** to form the Cu^I^MeCN‐**L1** complex (**Int0**). The deprotonation of carboxylic acid (**1a**) complexed with **Int0** yields the Cu^I^‐**L1**‐enoic acid (**Int1**), leading to a decrease in Gibbs energy by 16.5 kcal mol^−1^. **Int1**, along with the *p*‐methylphenyl diazonium compound, produce Cu^II^‐**L1**‐enoic acid (**Int2**) and an aryl radical through single electron transfer (SET), resulting in a decrease in the Gibbs energy by 19.4 kcal mol^−1^. Subsequently, the aryl radical captures sulfur dioxide to form a sulfonyl radical with a barrierless process. Although aryl radicals are theoretically capable of directly adding to alkenes, DFT calculations carried out by the Lei group reveal the presence of an energy barrier hindering this addition reaction.^[^ ^21^ ^]^ Therefore, in the presence of sulfur dioxide, the aryl radicals exhibit high chemical selectivity for capturing sulfur dioxide, rather than undergoing addition reactions with alkenes. This is consistent with the fact that by‐products of uncaptured SO_2_ were not detected in the experiment. The sulfonyl radical addition to the C═C bond of the Cu^II^‐**L1**‐enoic acid (**Int2**) via the **TS2** transition state, forming a new divalent copper complex (**Int3**) with the spin‐polarized singlet state. The **Int3** with the triplet state T^1^ was also evaluated, whose energy was higher than that of **Int3** with the spin‐polarized singlet state. The process releases an energy of 7.8 kcal mol^−1^, which is consistent with the documented high propensity of radicals towards unsaturated bonds.^[^ ^22^ ^]^ After the radical addition, **Int3** approaches the carbon radical with the Cu‐coordinated oxygen and passes through the **TS3** transition state to form the new divalent copper complex **Int4**. **TS3** undergoes distinct reactions to produce diastereoisomers **Int4‐*R*** and **Int4‐*S*** via **TS3‐*R*** and **TS3‐*S***, respectively. In this process, an intramolecular radical attack occurs onto the carboxylate oxygen atom, creating a chiral carbon atom according to the face of the approaching planar phenyl radical center. Subsequently, **Int4‐*R*** and **Int4‐*S*** release different enantiomers. Computational results suggest that **TS3‐*R*** is favored by 4.7 kcal mol^−1^, indicating a strong preference for the formation of the *R*‐product.

**Figure 4:**
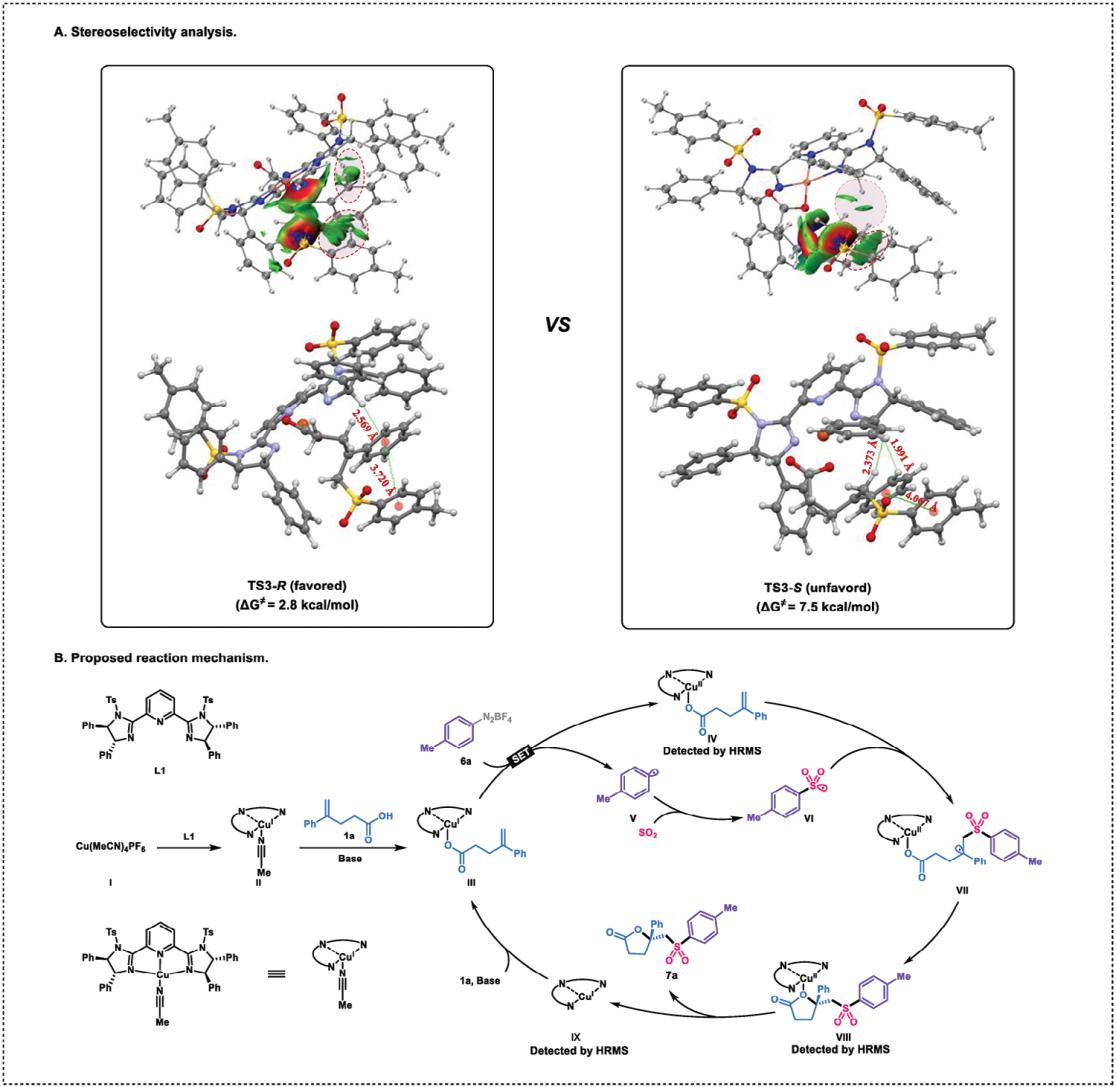
Stereoselectivity analysis and proposed reaction mechanism.

To provide a more comprehensive explanation of the enantioselective nature of the reaction, we conducted an analysis of the interactions between the ligand and the substrate at the transition states **TS3‐*R*** and **TS3‐*S*** (**Figure** **4**A). The comparative analysis of non‐covalent interactions in the two transition states reveals that **TS3‐*R*** possesses more favorable non‐covalent interactions. The *π*–*π* interactions between the Ts group and the aromatic group on the substrate are stronger in **TS3‐*R*** compared to **TS3‐*S***. Furthermore, **TS3‐*R*** demonstrates C–H–*π* interactions between the aromatic group on the substrate and the hydrogen on the imidazole of the ligand, while these interactions are absent in **TS3‐*S***. Moreover, **TS3‐*S*** exhibits a stronger H‐H repulsion between the substrate and the ligand. We measured the non‐bonding interaction distances and found that the distance between the Ts group and the aromatic group on the substrate was 0.347 Å longer in **TS3‐*S*** compared to **TS3‐*R***. Additionally, the distance between the hydrogen on the ligand's imidazole and the phenyl group on the substrate was 2.569 Å, indicating the potential formation of a C–H–*π* interaction. Therefore, **TS3‐*R*** is considered more stable.

Combining experimental results and literature reports,^[^ ^6a^ ^]^ we propose a potential mechanism for the reaction as illustrated in Figure 4B. First, a strong chelating Cu^I^‐PyBim species **II** is generated and then combines with reactant **1a** in the presence of a base, resulting in the formation of [PyBim/Cu^I^O_2_CR] species **III**. Then, species **III** undergoes a SET process with aryldiazonium salt **6a,** leading to the formation of [PyBim/Cu^II^O_2_CR] species **IV** and *p*‐methylphenyl radical **V**. Next, the *p*‐methylphenyl radical **V** captures SO_2_ to generate a sulfonyl radical **VI**, which subsequently adds to species **IV**, resulting in the formation of an alkyl radical **VII**. Then, an intramolecular radical substitution occurs, generating Cu^II^ complex **VIII**. The dissociation of species VIII leads to the formation of product **7a** and Cu^I^‐PyBim **IX**. Finally, Cu^I^‐PyBim **IX** combines with substrate 1a in the presence of a base to generate complex III for the next catalytic cycle. Intermediates **IV**, **VIII** and **IX** have been identified in the experiments by HRMS (for more details, refer to the Supporting Information).

## 3 Conclusion

In summary, we have developed a novel Cu/PyBim catalytic system and applied it to the asymmetric vicinal sulfonyl‐esterification of alkenes involving sulfur dioxide. The reaction is conducted under mild conditions and demonstrates good substrate tolerance, providing satisfactory product yields with excellent enantiocontrol. Notably, this catalytic system is applicable to different types of radical precursors, including *O*‐acylhydroxylamines, cycloketone oxime esters, aryldiazonium salts, and drug molecules. In the reaction, SOgen acts as a surrogate for sulfur dioxide and plays a crucial role. Mechanism studies and DFT calculation revealed that asymmetric coupling of C─O bonds was successfully achieved within the rigid chiral space formed by the tridentate non‐scorpionate ligand PyBim and copper salts, employing an outer‐sphere model.

## Conflict of Interest

The authors declare no conflict of interest.

## Supporting information

Supporting Information

## Data Availability

The data that support the findings of this study are available in the supplementary material of this article.
