# Supplementary material for: Three‐Component Radical Cross‐Coupling: Asymmetric Vicinal Sulfonyl‐Esterification of Alkenes Involving Sulfur Dioxide
Source: Adv Sci (Weinh). 2024 Mar 26;11(23):2309069. doi: 10.1002/advs.202309069 (PMC11186061; doi:10.1002/advs.202309069)
Supplement: Supplementary file 1 — Supporting Information [file ADVS-11-2309069-s001.pdf]

ADVANCED SCIENCE
Open Access

Supporting Information

for *Adv. Sci.*, DOI 10.1002/advs.202309069

Three-Component Radical Cross-Coupling: Asymmetric Vicinal Sulfonyl-Esterification of Alkenes Involving Sulfur Dioxide

*Zhiqian Chang, Xuemei Zhang, Haiping Lv, Haotian Sun and Zhong Lian**

Supporting Information

Three-Component Radical Cross-Coupling: Asymmetric Vicinal Sulfonyl-Esterification of Alkenes Involving Sulfur Dioxide

Zhiqian Chang,‡[a] Xuemei Zhang,‡[a] Haiping Lv,[a] Haotian Sun,[a] and Zhong Lian*[a]

[a] Department of Dermatology, State Key Laboratory of Biotherapy and Cancer Center, West China Hospital, Sichuan University, Chengdu 610041, China. E-mail: lianzhong@scu.edu.cn

‡These authors contributed equally.

Contents

Contents 1

General information 2

1. Optimization of the reaction conditions of compound **9** 3
2. General synthetic procedures and experimental methods 4
3. Characterization data of products 13
4. Transformations of **5a** 42
5. Transformations of **7a** 43
6. Transformations of **7r** 45
7. Nonlinear effect studies 45
8. Mechanistic experiments 47
9. Single crystal X-Ray diffraction data. 50
10. DFT calculations 58
11. Mass spectrometry experiments and proposed reaction mechanism 86
12. References 88
13. The spectrums of ^{1}H NMR, ^{13}C NMR, ^{19}F NMR, HPLC chromatograms. 91

General information

All reactions were carried out in oven dried two-chamber under argon atmosphere glovebox (Vigor, SGI800-750TS-F). Extra dry 2-Methyltetrahydrofuran (2-Me-THF), DCE and DCM were purchased from Energy Chemical. ^{1}H, ^{19}F, ^{13}C NMR spectra were recorded in $(CD_3)_2SO$ (or $CDCl_3$) on Bruker Avance 400 MHz or 600 MHz spectrometers. High-resolution mass data were recorded on a high-resolution mass spectrometer in the ESI mode. The molecular ion $[M+H]^+$, $[M+K]^+$, $[M+Na]^+$ and $[M–Ts+Na]^+$are given in m/z units. Column chromatography was generally performed on silica gel (200-300 mesh). For thin layer chromatography (TLC), Yantai pre-coated TLC plates (HSGF 254) were used, and compounds were visualized with a UV light at 254 nm. Further visualization was achieved by staining with $KMnO_4$ followed by heating. Enantiomeric excesses (ee) were determined by HPLC analysis on DIONEX UHPLC system with Daicel chiral columns (Chiralpak AD-H, IB, IA, OD-H, OJ-H, AS-H columns). Optical rotations were measured on a Hanon P850 polarimeter, and reported as $[\alpha]_\lambda^T$ (concentration (c): g/100 mL, in $CHCl_3$ or CH_2Cl_2).

1. Optimization of the reaction conditions of compound 9

1.1 Evaluation of the chiral ligand

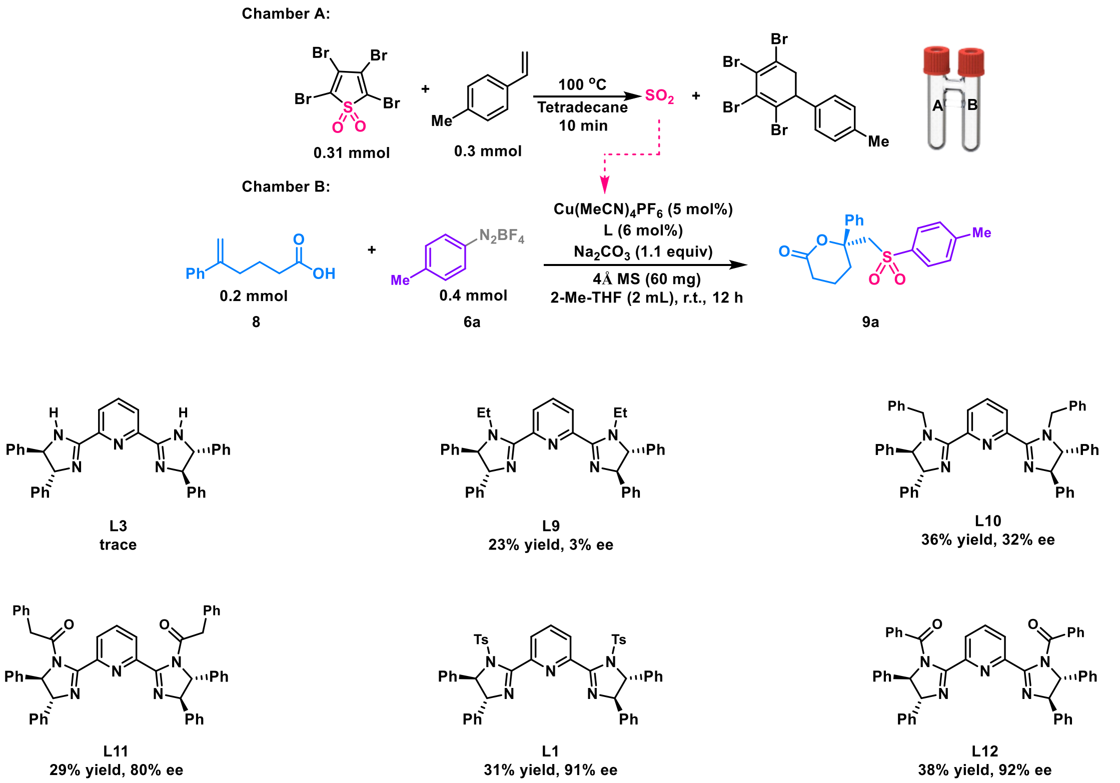

Reaction conditions. Chamber A: SOgen (177.0 mg, 0.41 mmol), 1-methyl-4-vinylbenzene (47.3 mg, 0.40 mmol), tetradecane (1.0 mL), at 100 °C for 10 min. Chamber B: **8** (38.0 mg, 0.2 mmol), **6a** (123.6 mg, 0.6 mmol, 3 equiv), Cu(MeCN)$_4$PF$_6$ (3.7 mg, 0.01 mmol, 5 mol%), **L** (0.012 mmol, 6 mol%), Na_2CO_3 (23.3 mg, 0.22 mmol, 1.1 equiv), 4Å Molecular Sieve (60 mg), 2-Me-THF (2.0 mL) at room temperature for 12 h under argon atmosphere.

1.2 Evaluation of the solvent.

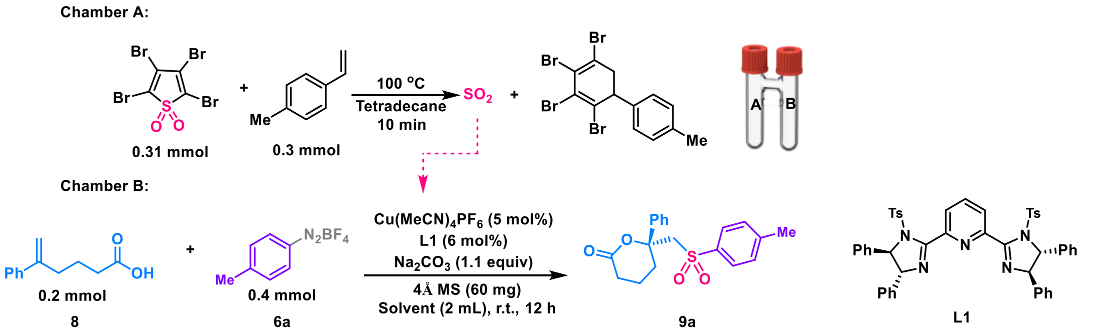

Entry	Solvent	Yield[b]	ee[c]
1	2-Me-THF	31%	91%
2	*t*-AmOH	30%	60%
3	DCM	48%	89%

[a] Reaction conditions. Chamber A: SOgen (177.0 mg, 0.41 mmol), 1-methyl-4-vinylbenzene (47.3 mg, 0.40 mmol), tetradecane (1.0 mL), at 100 °C for 10 min. Chamber B: **8** (38.0 mg, 0.2 mmol), **6a** (123.6 mg, 0.6 mmol, 3 equiv), Cu(MeCN)$_4$PF$_6$ (3.7 mg, 0.01 mmol, 5 mol%), **L1** (9.9 mg, 0.012 mmol, 6 mol%), Na_2CO_3 (23.3 mg, 0.22

mmol, 1.1 equiv), 4Å Molecular Sieve (60 mg), Solvent (2.0 mL) at room temperature for 12 h under argon atmosphere. [b] Isolated yield of **9a**. [c] The ee value of **9a** was determined by HPLC analysis on a chiral stationary phase.

1.3 Evaluation of the base and ligand

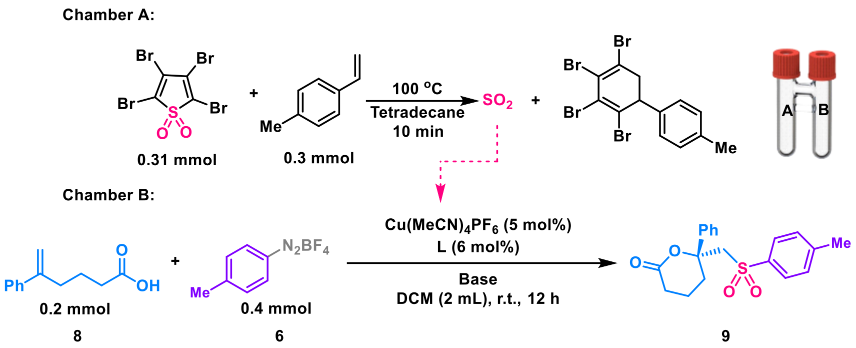

Entry	Ligand	Base	Equiv	Yield[b]	ee[c]
1	**L1**	Na_2CO_3	1.1	28%	91%
2	**L1**	2,6-di-*tert*-Butylpyridine	2.0	83%	90%
3	**L12**	2,6-di-*tert*-Butylpyridine	2.0	88%	92%

[a] Reaction conditions. Chamber A: SOgen (177.0 mg, 0.41 mmol), 1-methyl-4-vinylbenzene (47.3 mg, 0.40 mmol), tetradecane (1.0 mL), at 100 °C for 10 min. Chamber B: **8** (38.0 mg, 0.2 mmol), **6a** (123.6 mg, 0.6 mmol, 3 equiv), $Cu(MeCN)_4PF_6$ (3.7 mg, 0.01 mmol, 5 mol%), **L** (0.012 mmol, 6 mol%), Base (0.22 mmol/ 0.4 mmol), DCM (2.0 mL) at room temperature for 12 h under argon atmosphere. [b] Isolated yield of **9a**. [c] The ee value of **9a** was determined by HPLC analysis on a chiral stationary phase.

2. General synthetic procedures and experimental methods

2.1 General procedures for the synthesis of unsaturated carboxylic acids 1.[1]

a. General procedure for synthesis of ketone precursors using Friedel-Craft reaction.

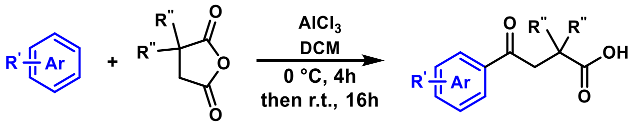

A 100 mL, three-necked, round-bottomed flask is charged with powdered dihydrofuran-2,5-dione (1.0 equiv) and arene (1.0 equiv) under dry nitrogen. The resulting white mixture was cooled to 0 °C before anhydrous aluminum trichloride (1.2 equiv) was added in one portion. The reaction mixture was stirred over a period of 4 h before allowing it to warm to room temperature for 16 h. The reaction was poured in ice and 10 mL of concentrated hydrochloric acid was added under stirring at 0 °C. The organic layer was separated and the aqueous layer was extracted with DCM twice. The combined organic layers were washed with water, dried over $MgSO_4$ and concentrated. Product was engaged in the next step without further purification.

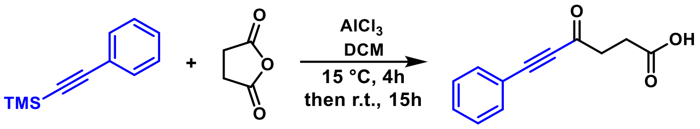

To a solution of succinic anhydride (1.2 g, 12 mmol, 1.2 equiv) in DCM (125 mL) in a 250 mL round-bottom flask fitted with a thermometer, and solvent addition funnel was added aluminum trichloride (2.3 g, 17 mmol). The reaction mass was cooled under stirring to 15 °C and a solution of trimethyl(phenylethynyl)silane (2.0 mL, 10 mmol) in 10 mL of DCM was added dropwise and the reaction mixture was stirred for 16 h at rt. The reaction was poured in ice and 10 mL of concentrated hydrochloric acid was added under stirring at 0 °C. The organic layer was separated and the aqueous layer was extracted with DCM twice (2×50 mL). The combined organic layers were washed with water, dried over $MgSO_4$ and concentrated to give the expected target compound.

b. General procedure for synthesis of ketone precursors using Grignard reaction.[2]

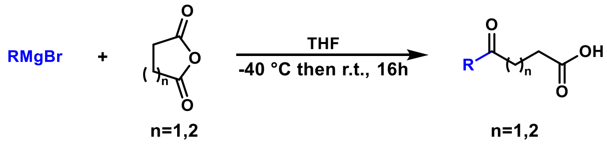

Anhydride (22 mmol, 1.1 equiv) was added to an oven-dried 100 mL three-neck round bottom flask under nitrogen, THF (1 mL/mmol) was added and the reaction mixture was cooled to -40 °C. RMgBr (20 mmol, 1.0 equiv) was added dropwise, followed by stirring for 1 h. After that, the solution was warmed to rt and then the solution was stirred at room temperature for 12 h. The reaction mixture was subsequently acidified with aqueous HCl (0.1 M) to pH=1 and extracted with ethyl acetate. The combined organic extracts were washed with brine and then dried over Na_2SO_4, concentrated in vacuo to give crude product. The residues were purified by flash column chromatography (silica-gel, petroleum ether/EtOAc = 3/1) to obtain product.

c. General procedure for synthesis of enoic acid using Wittig reaction.[1]

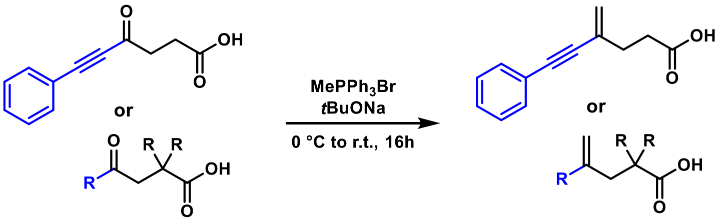

Under nitrogen, to a solution of *t*BuONa (2.6 equiv) in dry THF (0.5 M) was

added bromo(methyl)triphenylphosphorane (1.3 equiv) in portions at 0 °C. The mixture was stirred at 0 °C for 30 min and a solution of ketone (1.0 equiv) in dry THF (1 M) was added dropwise and the reaction was stirred at 0 °C for 1 h and at rt overnight. The solvent was removed in vacuo and the residue diluted with DCM and aqueous NaOH (1 M). The aqueous layer was separated, washed with dichloromethane, and acidified to pH 1 with concentrated HCl. DCM was added and the organic compound was extracted twice with DCM. The organic layer was washed with water, dried over $MgSO_4$ and concentrated. The crude product was purified by SiO_2 column chromate-graphy to give pure enoic acid.

d. General procedure for synthesis of enoic acid using Wittig reaction.[1]

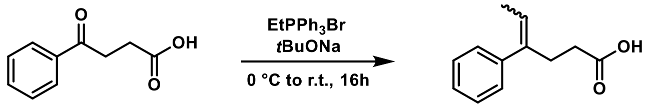

Under nitrogen, to a solution of *t*BuONa (2.6 equiv) in dry THF (0.5 M) was added bromo(Ethyl)triphenylphosphorane (1.3 equiv) in portions at 0 °C. The mixture was stirred at 0 °C for 30 min and a solution of ketone (1.0 equiv) in dry THF (1 M) was added dropwise and the reaction was stirred at 0 °C for 1 h and at rt overnight. The solvent was removed in vacuo and the residue diluted with DCM and aqueous NaOH (1 M). The aqueous layer was separated, washed with dichloromethane, and acidified to pH 1 with concentrated HCl. DCM was added and the organic compound was extracted twice with DCM. The organic layer was washed with water, dried over $MgSO_4$ and concentrated. The crude product was purified by SiO_2 column chromate-graphy to give pure enoic acid.

e. General procedure for synthesis of enoic acid using Knoevenagel Condensation reaction.[3]

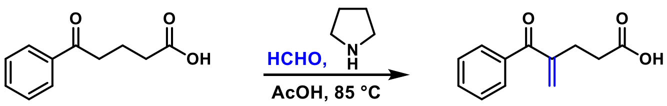

To a solution of keto acid (576.6 mg, 3.0 mmol, 1.0 equiv) in acetic acid (6 mL) was added pyrrolidine (0.1 mL, 1.2 mmol, 0.4 equiv) and formaldehyde solution (36.5-38% in H_2O, 1mL, 13.2 mmol, 4.4 equiv) at room temperature. The mixture was then stirred for 48 h at 85 °C. After evaporation of acetic acid, water and EtOAc were added. The organic layer was washed with water, and dried over magnesium sulfate. Concentration of the organic layer offered the crude product that was further purified by flash column chromatography (hexane/EtOAc) to give the α, β-

unsaturated ketone.

2.2 General procedures for the synthesis of aryldiazonium salts.[4]

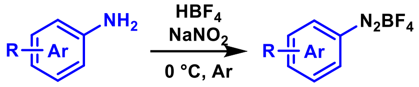

An oven-dried 100 mL round-bottom-flask equipped with a magnetic stir bar was charged with Arylamine (1 equiv, 20 mmol). The flask was sealed with a rubber septum and connected to a Schlenk line though a needle. The flask was then evacuated and backfilled with argon (This sequence was repeated a total of three times). The solution of HBF_4 (6.8 mL HBF_4 (50% wt) in 8 mL H_2O) was added via syringe. The mixture was the cooled to 0 °C, and $NaNO_2$ aqueous (1.4 g in 3 mL H_2O) was added dropwise via a syringe on 0 °C. The resulting mixture was stirred for 40 min at 0 °C on argon atmosphere. Then, the solvent was removed by filtered, and the result solid was dissolved in acetone, crystallized from the solution by added ice cold diethyl ether. The remaining solid was washed by ice cold diethyl ether (25 mL) three times to afford the product.

2.3 General procedures for the synthesis of *O*-(*tert*-butoxycarbonyl)oximes.[5, 6]

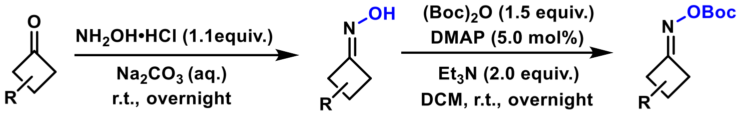

The ketone (5.0 mmol, 1.0 equiv) and hydroxylamine hydrochloride (5.5 mmol, 1.1 equiv) were placed in a 100 mL flask equipped with stirrer. The pH of the solution was held at 7–8 by adding saturated aq. sodium carbonate (10 mL). The resulting solution was stirred at 40 °C. After extraction with DCM, the solution was dried over Na_2SO_4 and evaporated to provide crude products which were used in the next step without further purification.

To the mixture of cyclobutanone oximes (1.0 equiv) prepared by conventional methods, triethylamine (2.0 equiv), DMAP (5.0 mol%) and dichloromethane (0.5 M) in a flask was added di-*tert*-butyl dicarbonate (1.5 equiv) slowly at room temperature. The mixture was stirred at room temperature for 30 minutes. Water was then added to quench the reaction. The resulting mixture was extracted with dichloromethane for three times. The organic phases were combined and dried over anhydrous Na_2SO_4. After the removal of solvent under reduced pressure, the crude product was purified by column chromatography on silica gel with ethyl acetate/petroleum ether as the eluent to give the O-(*tert*-butoxycarbonyl) oximes.

2.4 General procedures for the synthesis of *O*-benzoyl-*N*-hydroxylamines.[7-11]

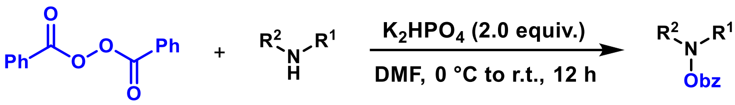

To a stirred suspension of benzoyl peroxide (12.0 mmol, 1.2 equiv) and K_2HPO_4 (20.0 mmol, 2.0 equiv) in DMF (25.0 mL) at 0 °C was added secondary amine (10.0 mmol). The reaction mixture was stirred at room temperature for 12 h, quenching with water and stirring vigorously for several minutes until all solids dissolved. The mixture was extracted with ethyl acetate, the organic layer was washed with saturated aq. $NaHCO_3$, water and brine, dried over anhydrous $MgSO_4$ and concentrated under reduced pressure. The crude product was then chromatographed on silica gel to afford the desired compound.

2.5 General procedures for the preparation of compound **3**.

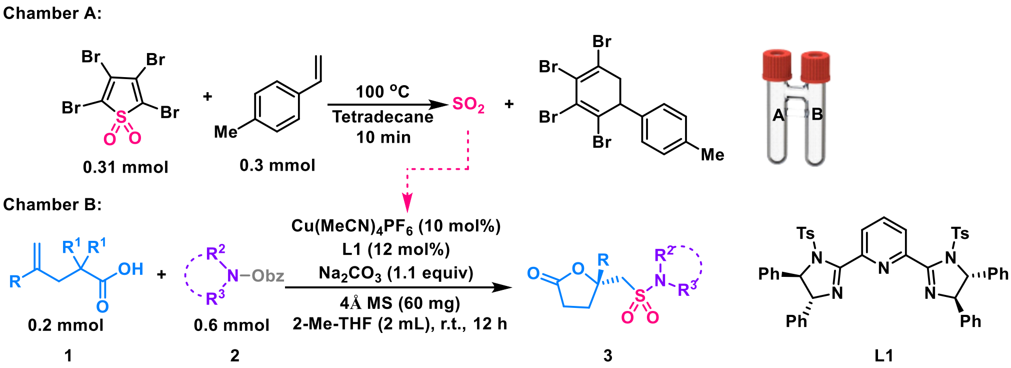

In an argon fulfilled glovebox, perbromothiophene 1,1-dioxide (SOgen) (133.8 mg, 0.31 mmol), 1-methyl-4-vinylbenzene (35.4 mg, 0.30 mmol), were added into chamber A with a magnetic stirring bar, followed by addition of tetradecane (1.0 mL). $Cu(MeCN)_4PF_6$ (7.5 mg, 0.020 mmol, 10 mol%), **L1** (19.9 mg, 0.024 mmol, 12 mol%), Compound **1** (0.2 mmol, 1.0 equiv), Compound **2** (0.6 mmol, 3 equiv), Na_2CO_3 (23.3 mg, 0.22 mmol, 1.1 equiv), 4Å MS (60 mg) and 2-Me-THF (2.0 mL) were added into chamber B with a magnetic stirring bar. The two-chamber was sealed and removed out of the glovebox. The chamber A was allowed to stir at 100 °C using heating mantle with 600-800 rpm stirring speed for 10 min. The two-chamber was allowed to stir at room temperature for 12 h. Upon completion, the reaction mixture was chromatographed on SiO_2 column. The residue was purified by flash silica gel column chromatography using petroleum ether/ethyl acetate/ triethylamine (v/v/v=100/100/1 to 50/100/1) as eluent to afford pure products **3**.

2.6 General procedures for the preparation of **racemic-3** for UHPLC analysis.

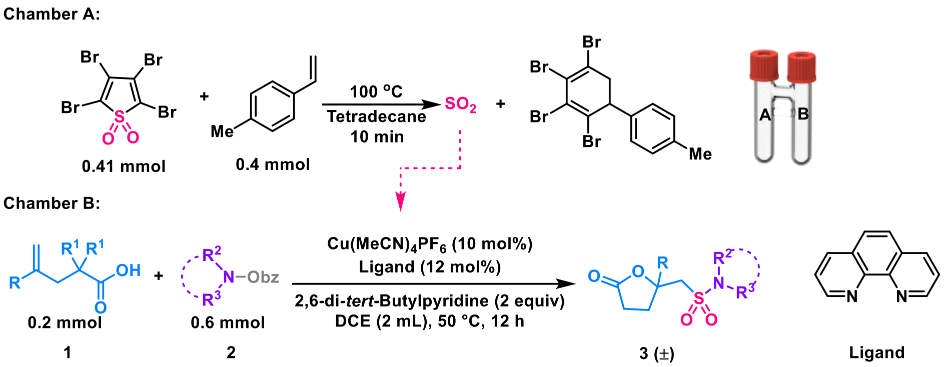

In an argon fulfilled glovebox, SOgen (133.8 mg, 0.31 mmol), 1-methyl-4-vinylbenzene (35.4 mg, 0.30 mmol), were added into chamber A with a magnetic stirring bar, followed by addition of tetradecane (1.0 mL). $Cu(MeCN)_4PF_6$ (0.020 mmol, 7.5 mg, 10 mol%), **Ligand** (0.024 mmol, 4.3 mg, 12 mol%), Compound **1** (0.2 mmol, 1.0 equiv), Compound **2** (0.6 mmol, 3.0 equiv), 2,6-di-*tert*-Butylpyridine (76.5 mg, 0.4 mmol, 2.0 equiv) and DCE (2.0 mL) were added into chamber B with a magnetic stirring bar. The two-chamber was sealed and removed out of the glovebox. The chamber A was allowed to stir at 100 °C using heating mantle with 600-800 rpm stirring speed for 10 min. The two-chamber was allowed to stir at 50 ℃ for 12 h. Upon completion, the reaction mixture was chromatographed on SiO_2 column. The residue was purified by flash silica gel column chromatography using petroleum ether/ethyl acetate/ triethylamine (v/v/v=100/100/1 to 50/100/1) as eluent to afford pure products **3**(±).

2.7 General procedures for the preparation of compound **5**.

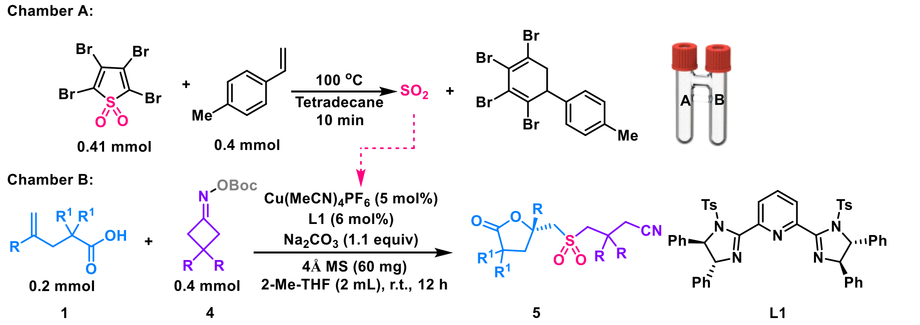

In an argon fulfilled glovebox, SOgen (177.0 mg, 0.41 mmol), 1-methyl-4-vinylbenzene (47.3 mg, 0.40 mmol), were added into chamber A with a magnetic stirring bar, followed by addition of tetradecane (1.0 mL). $Cu(MeCN)_4PF_6$ (3.7 mg , 0.010 mmol, 5 mol%), **L1** (9.9 mg, 0.012 mmol, 6 mol%), Compound **1** (0.2 mmol, 1.0 equiv), Compound **6** (0.4 mmol, 2.0 equiv), Na_2CO_3 (23.3 mg, 0.22 mmol, 1.1 equiv) and 4Å MS (60 mg) and 2-Me-THF (2.0 mL) were added into chamber B with a magnetic stirring bar. The two-chamber was sealed and removed out of the glovebox.

The chamber A was allowed to stir at 100 °C using heating mantle with 600-800 rpm stirring speed for 10 min. The two-chamber was allowed to stir at room temperature for 12 h. Upon completion, the reaction mixture was chromatographed on SiO_2 column. The residue was purified by flash silica gel column chromatography using petroleum ether /ethyl acetate (v/v =1/1 to 1/3) as eluent to afford pure products **5**.

2.8 General procedures for the preparation of **racemic-5** for UHPLC analysis

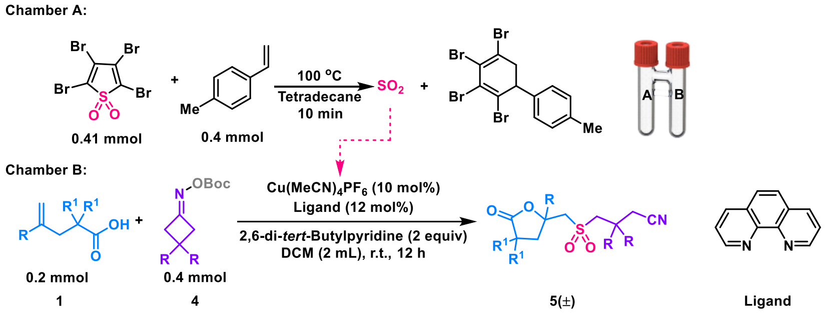

In an argon fulfilled glovebox, SOgen (177.0 mg, 0.41 mmol), 1-methyl-4-vinylbenzene (47.3 mg, 0.40 mmol), were added into chamber A with a magnetic stirring bar, followed by addition of tetradecane (1.0 mL). $Cu(MeCN)_4PF_6$ (7.5 mg, 0.020 mmol, 10 mol%), **Ligand** (4.3 mg, 0.024 mmol, 12 mol%), Compound **1** (0.2 mmol, 1.0 equiv), Compound **6** (0.4 mmol, 2.0 equiv), 2,6-di-*tert*-Butylpyridine (76.5 mg, 0.4 mmol, 2.0 equiv) and DCM (2.0 mL) were added into chamber B with a magnetic stirring bar. The two-chamber was sealed and removed out of the glovebox. The chamber A was allowed to stir at 100 °C using heating mantle with 600-800 rpm stirring speed for 10 min. The two-chamber was allowed to stir at room temperature ℃ for 12 h. Upon completion, the reaction mixture was chromatographed on SiO_2 column. The residue was purified by flash silica gel column chromatography using petroleum ether /ethyl acetate (v/v =1/1 to 1/3) as eluent to afford pure products **5** (±).

2.9 General procedures for the preparation of compound **7**

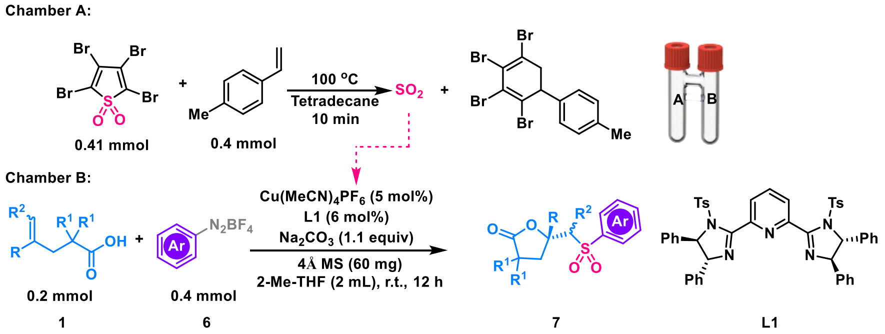

In an argon fulfilled glovebox, SOgen (177.0 mg, 0.41 mmol), 1-methyl-4-vinylbenzene (47.3 mg, 0.40 mmol), were added into chamber A with a magnetic

stirring bar, followed by addition of tetradecane (1.0 mL). $Cu(MeCN)_4PF_6$ (3.7 mg, 0.010 mmol, 5 mol%), **L1** (9.9 mg, 0.012 mmol, 6 mol%), Compound **1** (0.2 mmol, 1.0 equiv), Compound **4** (0.4 mmol, 2.0 equiv), Na_2CO_3 (23.3 mg, 0.22 mmol, 1.1 equiv) and 4Å MS (60 mg) and 2-Me-THF (2.0 mL) were added into chamber B with a magnetic stirring bar. The two-chamber was sealed and removed out of the glovebox. The chamber A was allowed to stir at 100 °C using heating mantle with 600-800 rpm stirring speed for 10 min. The two-chamber was allowed to stir at room temperature for 12 h. Upon completion, the reaction mixture was chromatographed on SiO_2 column. The residue was purified by flash silica gel column chromatography using dichloromethane/ethyl acetate (v/v =50/1 to 30/1) as eluent to afford pure products **7**.

2.10 General procedures for the preparation of **racemic-7** for UHPLC analysis

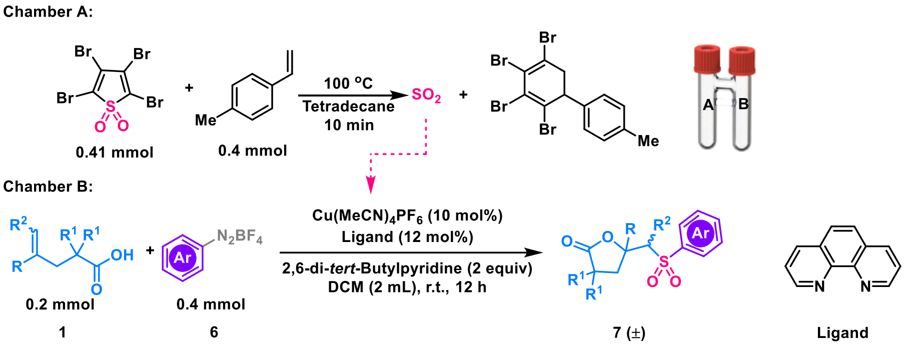

In an argon fulfilled glovebox, SOgen (177.0 mg, 0.41 mmol), 1-methyl-4-vinylbenzene (47.3 mg, 0.40 mmol), were added into chamber A with a magnetic stirring bar, followed by addition of tetradecane (1.0 mL). $Cu(MeCN)_4PF_6$ (7.5 mg, 0.020 mmol, 10 mol%), **Ligand** (4.3 mg, 0.024 mmol, 12 mol%), Compound **1** (0.2 mmol, 1.0 equiv), Compound **2** (0.4 mmol, 2.0 equiv), 2,6-di-*tert*-Butylpyridine (76.5 mg, 0.4 mmol, 2.0 equiv) and DCM (2.0 mL) were added into chamber B with a magnetic stirring bar. The two-chamber was sealed and removed out of the glovebox. The chamber A was allowed to stir at 100 °C using heating mantle with 600-800 rpm stirring speed for 10 min. The two-chamber was allowed to stir at room temperature °C for 12 h. Upon completion, the reaction mixture was chromatographed on SiO_2 column. The residue was purified by flash silica gel column chromatography using dichloromethane/ethyl acetate (v/v =50/1 to 30/1) as eluent to afford pure products **7** (±).

2.11 General procedures for the preparation of compound **9**

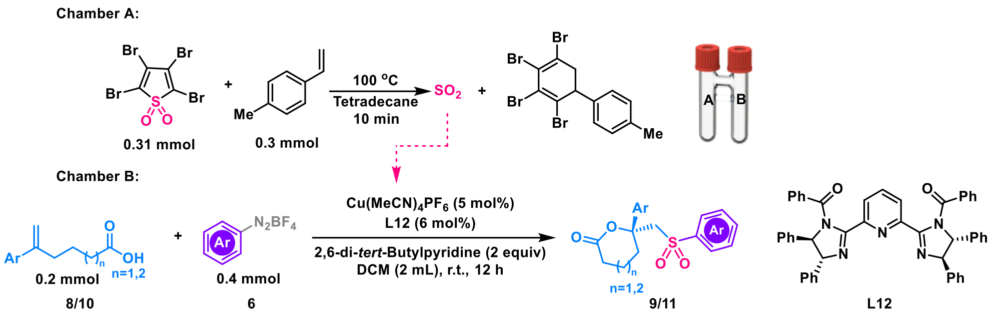

In an argon fulfilled glovebox, SOgen (177.0 mg, 0.41 mmol), 1-methyl-4-vinylbenzene (47.3 mg, 0.40 mmol), were added into chamber A with a magnetic stirring bar, followed by addition of tetradecane (1.0 mL). $Cu(MeCN)_4PF_6$ (3.7 mg, 0.010 mmol, 5 mol%), **L12** (8.7 mg, 0.012 mmol, 6 mol%), Compound **8** (0.2 mmol, 1.0 equiv), Compound **4** (0.4 mmol, 2.0 equiv), 2,6-di-*tert*-Butylpyridine (76.5 mg, 0.4 mmol, 2.0 equiv) and DCM (2.0 mL) were added into chamber B with a magnetic stirring bar. The two-chamber was sealed and removed out of the glovebox. The chamber A was allowed to stir at 100 °C using heating mantle with 600-800 rpm stirring speed for 10 min. The two-chamber was allowed to stir at room temperature for 12 h. Upon completion, the reaction mixture was chromatographed on SiO_2 column. The residue was purified by flash silica gel column chromatography using dichloromethane/ethyl acetate (v/v =50/1 to 30/1) as eluent to afford pure products **9**.

2.12 General procedures for the preparation of **racemic-9** for UHPLC analysis

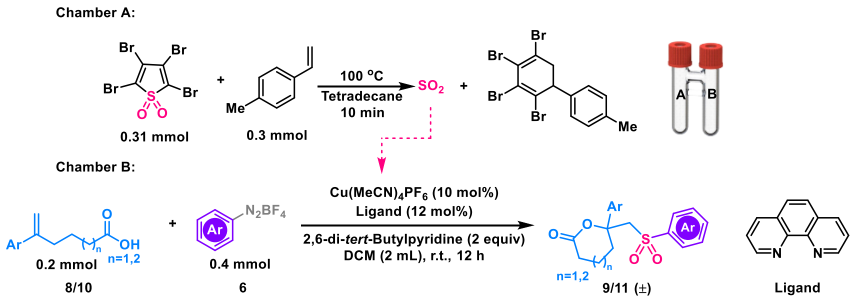

In an argon fulfilled glovebox, SOgen (177.0 mg, 0.41 mmol), 1-methyl-4-vinylbenzene (47.3 mg, 0.40 mmol), were added into chamber A with a magnetic stirring bar, followed by addition of tetradecane (1.0 mL). $Cu(MeCN)_4PF_6$ (7.5 mg, 0.020 mmol, 10 mol%), **Ligand** (4.3 mg, 0.024 mmol, 12 mol%), Compound **8** (0.2 mmol, 1.0 equiv), Compound **4** (0.2 mmol, 2.0 equiv), 2,6-di-*tert*-Butylpyridine (76.5 mg, 0.4 mmol, 2.0 equiv) and DCM (2.0 mL) were added into chamber B with a magnetic stirring bar. The two-chamber was sealed and removed out of the glovebox. The chamber A was allowed to stir at 100 °C using heating mantle with 600-800 rpm stirring speed for 10 min. The two-chamber was allowed to stir at room temperature °C

for 12 h. Upon completion, the reaction mixture was chromatographed on SiO_2 column. The residue was purified by flash silica gel column chromatography using dichloromethane/ethyl acetate (v/v =50/1 to 30/1) as eluent to afford pure products **9** (±).

3. Characterization data of products

(*R*)-5-((morpholinosulfonyl)methyl)-5-phenyldihydrofuran-2(3*H*)-one

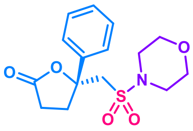

Compound 3a. (92% yield, 96% ee (*R*)). White solid, 59.9 mg at 0.20 mmol scale. The ee of **3a** was determined by HPLC analysis: (Chiralcel AD-H column, 1.0 mL/min, hexane/isopropanol = 50/50, 210 nm, 25 °C, t_{major} = 15.66 min (*R*), t_{minor} = 8.02 min (*S*)); $[\alpha]^{16}_{D}$ +4.1 (*c* 1.13, $CHCl_3$) for 96% ee (*R*). ^1H NMR (400 MHz, $CDCl_3$) δ 2.45 – 2.57 (m, 1H), 2.58 – 2.69 (m, 1H), 2.75 – 2.88 (m, 1H), 3.10 – 3.37 (m, 5H), 3.45 – 3.58 (m, 2H), 3.62 – 3.87 (m, 4H), 7.33 – 7.60 (m, 5H). ^{13}C NMR (101 MHz, $CDCl_3$) δ 28.3, 32.6, 45.6, 58.4, 66.5, 84.5, 124.5, 128.7, 129.1, 142.2, 175.4. HRMS (ESI) m/z calcd for $C_{15}H_{19}NNaO_5S^+$ $[M+Na]^+$ 348.0876, found 348.0873.

(*R*)-5-([1,1'-biphenyl]-4-yl)-5-((morpholinosulfonyl)methyl)dihydrofuran-2(3*H*)-one

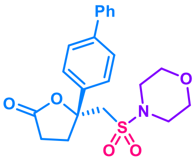

Compound 3b. (76% yield, 92% ee (*R*)). White solid, 61.0 mg at 0.20 mmol scale. The ee of **3b** was determined by HPLC analysis: (Chiralcel AD-H column, 1.0 mL/min, hexane/isopropanol = 50/50, 210 nm, 25 °C, t_{major} = 24.76 min (*R*), t_{minor} = 16.10 min (*S*)); $[\alpha]^{17}_{D}$ −4.8 (*c* 1.18, $CHCl_3$) for 92% ee (*R*). ^1H NMR (400 MHz, $CDCl_3$) δ 2.39 – 2.52 (m Hz, 1H), 2.52 – 2.62 (m, 1H), 2.69 – 2.82 (m, 1H), 3.06 – 3.27 (m, 5H), 3.38 – 3.52 (m, 2H), 3.58 – 3.79 (m, 4H), 7.27 – 7.46 (m, 5H), 7.49 – 7.61 (m, 4H). ^{13}C NMR (101 MHz, $CDCl_3$) δ 28.4, 32.6, 45.6, 58.4, 66.5, 84.5, 125.0, 127.1, 127.7, 127.8, 128.9, 140.0, 141.1, 141.6, 175.4. HRMS (ESI) m/z calcd for $C_{21}H_{23}NNaO_5S^+$ $[M+Na]^+$ 424.1189, found 424.1187.

(*R*)-5-((morpholinosulfonyl)methyl)-5-(naphthalen-2-yl)dihydrofuran-2(3*H*)-one

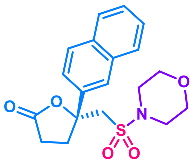

Compound 3c. (95% yield, 92% ee (*R*)). White solid, 71.3 mg at 0.20 mmol scale. The ee of **3c** was determined by HPLC analysis: (Chiralcel AD-H column, 1.0 mL/min, hexane/isopropanol = 50/50, 210 nm, 25 °C, t_{major} = 16.28 min (*R*), t_{minor} = 10.66 min (*S*)); $[\alpha]^{15}_{D}$ −2.0 (*c* 1.55, $CHCl_3$) for 92% ee (*R*). ^1H NMR (400 MHz, $CDCl_3$) δ 2.45 – 2.60 (m,

1H), 2.65 – 2.77 (m, 1H), 2.78 – 2.92 (m, 1H), 3.12 – 3.37 (m, 5H), 3.62 (s, 2H), 3.64 – 3.82 (m, 4H), 7.37 – 7.50 (m, 1H), 7.50 – 7.61 (m, 2H), 7.78 – 8.07 (m, 4H). ^{13}C NMR (101 MHz, $CDCl_3$) δ 28.3, 32.6, 45.6, 58.2, 66.4, 84.7, 122.2, 123.5, 127.0, 127.1, 127.7, 128.3, 129.2, 132.9, 132.9, 139.3, 175.6. HRMS (ESI) m/z calcd for $C_{19}H_{21}NNaO_5S^+$ $[M+Na]^+$ 398.1033, found 398.1031.

(*R*)-5-((morpholinosulfonyl)methyl)-5-(pyren-1-yl)dihydrofuran-2(3*H*)-one

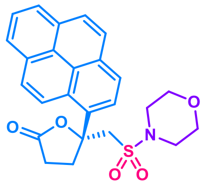

Compound 3d. (80% yield, 85% ee (*R*)). White solid, 71.9 mg at 0.20 mmol scale. The ee of **3d** was determined by HPLC analysis: (Chiralcel AD-H column, 1.0 mL/min, hexane/isopropanol = 60/40, 210 nm, 25 °C, t_{major} = 20.76 min (*R*), t_{minor} = 28.73 min (*S*)); $[\alpha]^{15}_D$ −22.7 (*c* 1.21, $CHCl_3$) for 85% ee (*R*). ^1H NMR (400 MHz, $CDCl_3$) δ 2.52 – 2.69 (m, 1H), 2.93 – 3.13 (m, 2H), 3.13 – 3.34 (m, 4H), 3.58 – 3.80 (m, 5H), 3.80 – 3.95 (m, 1H), 4.13 (d, *J* = 16.0 Hz, 1H), 8.03 – 8.16 (m, 3H), 8.16 – 8.33 (m, 5H), 8.35 (d, *J* = 8.2 Hz, 1H). ^{13}C NMR (101 MHz, $CDCl_3$) δ 28.8, 32.9, 45.7, 57.4, 66.4, 85.7, 122.4, 122.6, 124.9, 125.0, 125.9, 126.0, 126.4, 126.7, 127.4, 128.3, 128.9, 130.0, 131.4, 131.9, 135.8, 175.7. HRMS (ESI) m/z calcd for $C_{25}H_{23}NNaO_5S^+$ $[M+Na]^+$ 472.1189, found 472.1190.

(*R*)-5-((morpholinosulfonyl)methyl)-5-(*m*-tolyl)dihydrofuran-2(3*H*)-one

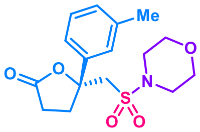

Compound 3e. (91% yield, 97% ee (*R*)). White solid, 61.8 mg at 0.20 mmol scale. The ee of **3e** was determined by HPLC analysis: (Chiralcel AD-H column, 1.0 mL/min, hexane/isopropanol = 50/50, 210 nm, 25 °C, t_{major} = 9.08 min (*R*), t_{minor} = 6.44 min (*S*)); $[\alpha]^{16}_D$ +1.9 (*c* 1.28, $CHCl_3$) for 97% ee (*R*). ^1H NMR (400 MHz, $CDCl_3$) δ 2.41 (s, 3H), 2.46 – 2.66 (m, 2H), 2.75 – 2.88 (m, 1H), 3.15 – 3.31 (m, 5H), 3.45 – 3.56 (m, 2H), 3.66 – 3.81 (m, 4H), 7.14 – 7.26 (m, 3H), 7.33 (t, *J* = 7.6 Hz, 1H). ^{13}C NMR (101 MHz, $CDCl_3$) δ 21.7, 28.4, 32.6, 45.6, 58.4, 66.5, 84.6, 121.5, 125.1, 129.0, 129.4, 139.0, 142.4, 175.7. HRMS (ESI) m/z calcd for $C_{16}H_{21}NNaO_5S^+$ $[M+Na]^+$ 362.1033, found 362.1031.

(*R*)-5-(4-(*tert*-butyl)phenyl)-5-((morpholinosulfonyl)methyl)dihydrofuran-2(3*H*)-one

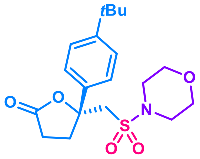

Compound 3f. (91% yield, 97% ee (*R*)). White solid, 69.4 mg at 0.20 mmol scale. The ee of **3f** was determined by HPLC analysis: (Chiralcel AD-H column, 1.0 mL/min, hexane/isopropanol = 50/50, 210 nm, 25 °C, t_{major} = 7.88 min (*R*), t_{minor} = 7.06 min (*S*)); $[\alpha]^{15}_D$

−10.0 (*c* 1.18, $CHCl_3$) for 97% ee (*R*). ^{1}H NMR (400 MHz, $CDCl_3$) δ 1.34 (s, 9H), 2.45 – 2.57 (m, 1H), 2.57 – 2.68 (m, 1H), 2.74 – 2.86 (m, 1H), 3.13 – 3.33 (m, 5H), 3.45 – 3.58 (m, 2H), 3.66 – 3.80 (m, 4H), 7.34 (d, *J* = 8.0 Hz, 2H), 7.45 (d, *J* = 8.0 Hz, 2H). ^{13}C NMR (101 MHz, $CDCl_3$) δ 28.5, 31.3, 32.4, 34.7, 45.6, 58.4, 66.5, 84.6, 124.3, 126.0, 139.4, 151.9, 175.8. HRMS (ESI) m/z calcd for $C_{19}H_{27}NNaO_5S^+$ $[M+Na]^+$ 404.1502, found 404.1501.

(*R*)-5-(3-methoxyphenyl)-5-((morpholinosulfonyl)methyl)dihydrofuran-2(3*H*)-one

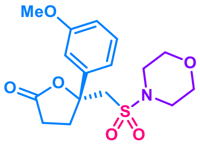

Compound 3g. (89% yield, 90% ee (*R*)). White solid, 63.3 mg at 0.20 mmol scale. The ee of **3g** was determined by HPLC analysis: (Chiralcel AD-H column, 1.0 mL/min, hexane/isopropanol = 50/50, 210 nm, 25 °C, t_{major} = 12.81 min (*R*), t_{minor} = 9.65 min (*S*)); $[\alpha]^{15}_{D}$ +20.1 (*c* 1.37, $CHCl_3$) for 90% ee (*R*). ^{1}H NMR (400 MHz, $CDCl_3$) δ 2.44 – 2.66 (m, 2H), 2.75 – 2.87 (m, 1H), 3.14 – 3.31 (m, 5H), 3.43 – 3.59 (m, 2H), 3.64 – 3.78 (m, 4H), 3.84 (s, 3H), 6.85 – 6.93 (m, 1H), 6.92 – 7.00 (m, 2H), 7.35 (t, *J* = 8.24 Hz, 1H). ^{13}C NMR (101 MHz, $CDCl_3$) δ 28.3, 32.6, 45.6, 55.4, 58.2, 66.5, 84.4, 110.6, 113.6, 116.6, 130.2, 144.0, 160.0, 175.4. HRMS (ESI) m/z calcd for $C_{16}H_{21}NNaO_6S^+$ $[M+Na]^+$ 378.0982, found 378.0983.

(*R*)-5-(3-fluorophenyl)-5-((morpholinosulfonyl)methyl)dihydrofuran-2(3*H*)-one

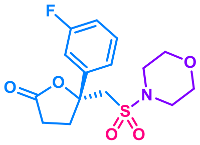

Compound 3h. (93% yield, 94% ee (*R*)). White solid, 63.9 mg at 0.20 mmol scale. The ee of **3h** was determined by HPLC analysis: (Chiralcel AD-H column, 1.0 mL/min, hexane/isopropanol = 50/50, 210 nm, 25 °C, t_{major} = 10.61min (*R*), t_{minor} = 7.11 min (*S*)); $[\alpha]^{15}_{D}$ +1.4 (*c* 0.86, $CHCl_3$) for 94% ee (*R*). ^{1}H NMR (400 MHz, $CDCl_3$) δ 2.46 – 2.66 (m, 2H), 2.75 – 2.90 (m, 1H), 3.12 – 3.34 (m, 5H), 3.41 – 3.58 (m, 2H), 3.67 – 3.85 (m, 4H), 7.03 – 7.11 (m, 1H), 7.12 – 7.26 (m, 2H), 7.36 – 7.48 (m, 1H). ^{19}F NMR (376 MHz, $CDCl_3$) δ -110.7. ^{13}C NMR (101 MHz, $CDCl_3$) δ 28.3, 32.8, 45.7, 58.1, 66.5, 84.0, 112.1 (d, *J* = 23.6 Hz), 115.8 (d, *J* = 21.1 Hz), 120.2 (d, *J* = 3.1 Hz), 130.9 (d, *J* = 8.2 Hz), 144.9 (d, *J* = 6.8 Hz), 163.0 (d, *J* = 249.3 Hz), 175.2. HRMS (ESI) m/z calcd for $C_{15}H_{18}FNNaO_5S^+$ $[M+Na]^+$ 366.0782, found 366.0780.

(*R*)-5-(4-fluorophenyl)-5-((morpholinosulfonyl)methyl)dihydrofuran-2(3*H*)-one

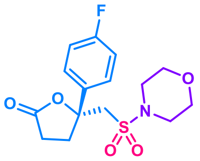

Compound 3i. (91% yield, 96% ee (*R*)). White solid, 62.5 mg at 0.20 mmol scale. The ee of **3i** was determined by HPLC analysis: (Chiralcel AD-H column, 1.0 mL/min, hexane/isopropanol = 50/50,

210 nm, 25 °C, t_{major} = 15.23 min (*R*), t_{minor} = 8.32 min (*S*)); $[\alpha]^{15}_{D}$ +2.8 (*c* 1.39, $CHCl_3$) for 96% ee (*R*). ^1H NMR (400 MHz, $CDCl_3$) δ 2.42 – 2.68 (m, 2H), 2.75 – 2.88 (m, 1H), 3.09 – 3.31 (m, 5H), 3.41 – 3.57 (m, 2H), 3.66 – 3.79 (m, 4H), 7.06 – 7.18 (m, 2H), 7.36 – 7.48 (m, 2H). ^{19}F NMR (376 MHz, $CDCl_3$) δ -112.9. ^{13}C NMR (101 MHz, $CDCl_3$) δ 28.3, 32.8, 45.6, 58.4, 66.4, 84.2, 115.9 (d, *J* = 21.8 Hz), 126.6 (d, *J* = 8.3 Hz), 137.9 (d, *J* = 3.3 Hz), 162.6 (d, *J* = 249.6 Hz), 175.2. HRMS (ESI) m/z calcd for $C_{15}H_{18}FNNaO_5S^+$ $[M+Na]^+$ 366.0782, found 366.0781.

(*R*)-5-(4-iodophenyl)-5-((morpholinosulfonyl)methyl)dihydrofuran-2(3*H*)-one

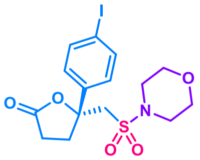

Compound 3j. (84% yield, 96% ee (*R*)). White solid, 75.8 mg at 0.20 mmol scale. The ee of **3j** was determined by HPLC analysis: (Chiralcel AD-H column, 1.0 mL/min, hexane/isopropanol = 50/50, 210 nm, 25 °C, t_{major} = 20.83 min (*R*), t_{minor} = 11.05 min (*S*)); $[\alpha]^{15}_{D}$ −3.0 (*c* 1.65, $CHCl_3$) for 96% ee (*R*). ^1H NMR (400 MHz, $CDCl_3$) δ 2.40 – 2.67 (m, 2H), 2.73 – 2.90 (m, 1H), 3.05 – 3.32 (m, 5H), 3.40 – 3.55 (m, 2H), 3.64 – 3.81 (m, 4H), 7.17 (d, *J* = 8.0 Hz, 2H), 7.69 – 7.82 (m, 2H). ^{13}C NMR (101 MHz, $CDCl_3$) δ 28.2, 32.7, 45.6, 58.1, 66.4, 84.2, 94.5, 126.5, 138.1, 141.9, 175.1. HRMS (ESI) m/z calcd for $C_{15}H_{18}INNaO_5S^+$ $[M+Na]^+$ 473.9843, found 473.9842.

(*R*)-5-((azepan-1-ylsulfonyl)methyl)-5-phenyldihydrofuran-2(3*H*)-one

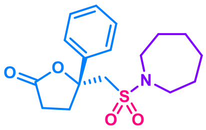

Compound 3k. (82% yield, 92% ee (*R*)). White solid, 55.3 mg at 0.20 mmol scale. The ee of **3k** was determined by HPLC analysis: (Chiralcel AD-H column, 1.0 mL/min, hexane/isopropanol = 50/50, 210 nm, 25 °C, t_{major} = 9.74 min (*R*), t_{minor} = 6.48 min (*S*)); $[\alpha]^{15}_{D}$ +11.8 (*c* 0.94, $CHCl_3$) for 92% ee (*R*). ^1H NMR (400 MHz, $CDCl_3$) δ 1.48 – 1.54 (m, 3H), 1.56 – 1.72 (m, 5H), 2.35 – 2.56 (m, 2H), 2.67 – 2.78 (m, 1H), 3.08 – 3.28 (m, 5H), 3.33 – 3.57 (m, 2H), 7.24 – 7.30 (m, 1H), 7.30 – 7.37 (m, 4H). ^{13}C NMR (101 MHz, $CDCl_3$) δ 26.9, 28.5, 29.6, 32.5, 48.3, 59.3, 85.0, 124.6, 128.6, 129.0, 142.4, 175.8. HRMS (ESI) m/z calcd for $C_{17}H_{23}NNaO_4S^+$ $[M+Na]^+$ 360.1240, found 360.1240.

(*R*)-*N*,*N*-dibenzyl-1-(5-oxo-2-phenyltetrahydrofuran-2-yl)methanesulfonamide

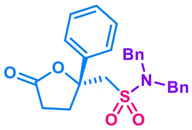

Compound 3l. (85% yield, 98% ee (*R*)). White solid, 74.0 mg at 0.20 mmol scale. The ee of **3l** was determined by HPLC analysis: (Chiralcel OD-H column, 1.0 mL/min, hexane/isopropanol = 80/20, 210 nm, 25 °C, t_{major} = 24.98 min (*R*), t_{minor} = 21.14 min (*S*)); $[\alpha]^{19}_{D}$ +2.1 (*c* 2.15, $CHCl_3$) for 98% ee (*R*). ^1H NMR (400 MHz, $CDCl_3$) δ 2.42 – 2.56 (m, 1H), 2.56 – 2.69 (m, 1H), 2.74 – 2.88 (m, 1H), 3.14 – 3.27 (m, 1H), 3.34 – 3.50 (m, 2H), 4.25 –

4.49 (m, 4H), 7.23 – 7.54 (m, 15H). ^{13}C NMR (101 MHz, $CDCl_3$) δ 28.4, 32.6, 50.4, 62.4, 84.9, 124.6, 128.1, 128.5, 128.8, 128.9, 135.3, 142.2, 175.5. HRMS (ESI) m/z calcd for $C_{25}H_{25}NNaO_4S^+$ $[M+Na]^+$ 458.1397, found 458.1397.

(*R*)-*N*,*N*-diethyl-1-(5-oxo-2-phenyltetrahydrofuran-2-yl)methanesulfonamide

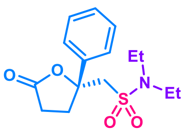

Compound 3m. (92% yield, 92% ee (*R*)). White solid, 57.3 mg at 0.20 mmol scale. The ee of **3m** was determined by HPLC analysis: (Chiralcel AD-H column, 1.0 mL/min, hexane/isopropanol = 70/30, 210 nm, 25 °C, t_{major} = 9.32 min (*R*), t_{minor} = 6.64 min (*S*)); $[\alpha]^{19}_{D}$ +16.7 (*c* 1.18, $CHCl_3$) for 92% ee (*R*). ^{1}H NMR (400 MHz, $CDCl_3$) δ 1.10 (t, *J* = 8.0 Hz, 6H), 2.33 – 2.47 (m, 1H), 2.47 – 2.58 (m, 1H), 2.69 – 2.82 (m, 1H), 3.07 – 3.29 (m, 5H), 3.34 – 3.46 (m, 2H), 7.26 – 7.30 (m, 1H), 7.30 – 7.44 (m, 4H). ^{13}C NMR (101 MHz, $CDCl_3$) δ 14.5, 28.5, 32.3, 41.8, 61.1, 85.0, 124.5, 128.5, 128.9, 142.6, 175.7. HRMS (ESI) m/z calcd for $C_{15}H_{21}NNaO_4S^+$ $[M+Na]^+$ 334.1083, found 334.1081.

(*R*)-*N*-benzyl-*N*-ethyl-1-(5-oxo-2-phenyltetrahydrofuran-2-yl)methanesulfon-amide

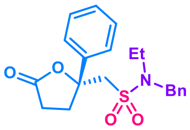

Compound 3n. (82% yield, 94% ee (*R*)). White solid, 61.2 mg at 0.20 mmol scale. The ee of **3n** was determined by HPLC analysis: (Chiralcel AD-H column, 1.0 mL/min, hexane/isopropanol = 70/30, 210 nm, 25 °C, t_{major} = 9.52 min (*R*), t_{minor} = 8.47 min (*S*)); $[\alpha]^{19}_{D}$ +6.9 (*c* 1.21, $CHCl_3$) for 94% ee (*R*). ^{1}H NMR (400 MHz, $CDCl_3$) δ 1.04 (t, *J* = 7.2 Hz, 3H), 2.42 – 2.53 (m, 1H), 2.54 – 2.64 (m, 1H), 2.75 – 2.86 (m, 1H), 3.11 – 3.32 (m, 3H), 3.42 – 3.55 (m, 2H), 4.24 – 4.44 (m, 2H), 7.26 – 7.37 (m, 6H), 7.38 – 7.43 (m, 4H). ^{13}C NMR (101 MHz, $CDCl_3$) δ 13.7, 28.4, 32.5, 42.1, 50.6, 61.5, 85.0, 124.6, 127.9, 128.3, 128.5, 128.7, 128.9, 136.2, 142.4, 175.6. HRMS (ESI) m/z calcd for $C_{20}H_{23}NNaO_4S^+$ $[M+Na]^+$ 396.1240, found 396.1238.

(*R*)-*N*-methyl-*N*-(naphthalen-1-ylmethyl)-1-(5-oxo-2-phenyltetrahydrofuran-2-yl) methane-sulfonamide

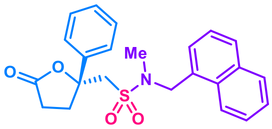

Compound 3o. (76% yield, 90% ee (*R*)). Colorless oil, 62.2 mg at 0.20 mmol scale. The ee of **3o** was determined by HPLC analysis: (Chiralcel AD-H column, 1.0 mL/min, hexane/isopropanol = 95/5, 210 nm, 25 °C, t_{major} = 75.32 min (*R*), t_{minor} = 83.08 min (*S*)); $[\alpha]^{18}_{D}$ +10.4 (*c* 0.83, $CHCl_3$) for 90% ee (*R*). ^{1}H NMR (400 MHz, $CDCl_3$) δ 2.38 – 2.51 (m, 1H), 2.52 – 2.60 (m, 1H), 2.62 (s, 3H), 2.71 – 2.84 (m, 1H), 3.15 – 3.24 (m, 1H), 3.43 – 3.66 (m, 2H), 4.55 – 4.77 (m, 2H), 7.22 – 7.55 (m, 9H), 7.70 – 7.82 (m,

2H), 8.17 (d, J = 8.4 Hz, 1H). ^{13}C NMR (101 MHz, $CDCl_3$) δ 28.4, 32.7, 34.1, 52.0, 58.4, 84.8, 123.7, 124.6, 125.1, 126.1, 126.8, 127.4, 128.6, 128.7, 129.0, 129.2, 130.5, 131.6, 133.9, 142.3, 175.6. HRMS (ESI) m/z calcd for $C_{23}H_{23}NNaO_4S^+$ $[M+Na]^+$ 432.1240, found 432.1240.

(*R*)-*N*-(2-cyanoethyl)-*N*-methyl-1-(5-oxo-2-phenyltetrahydrofuran-2-yl)methane-sulfonamide

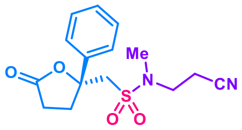

Compound 3p. (65% yield, 97% ee (*R*)). White solid, 41.9 mg at 0.20 mmol scale. The ee of **3p** was determined by HPLC analysis: (Chiralcel AD-H column, 1.0 mL/min, hexane/isopropanol = 70/30, 210 nm, 25 °C, t_{major} = 69.45 min (*R*), t_{minor} = 60.82 min (*S*)); $[\alpha]^{19}_D$ +11.8 (*c* 0.98, $CHCl_3$) for 97% ee (*R*). ^{1}H NMR (400 MHz, $CDCl_3$) δ 2.42 – 2.68 (m, 4H), 2.70 – 2.82 (m, 1H), 2.95 (s, 3H), 3.06 – 3.18 (m, 1H), 3.34 – 3.53 (m, 2H), 3.58 – 3.75 (m, 2H), 7.32 – 7.68 (m, 5H). ^{13}C NMR (101 MHz, $CDCl_3$) δ 18.3, 28.1, 33.4, 35.4, 46.2, 59.5, 84.7, 117.7, 124.5, 128.8, 129.2, 141.7, 175.6. HRMS (ESI) m/z calcd for $C_{15}H_{18}N_2NaO_4S^+$ $[M+Na]^+$ 345.0879, found 345.0876.

(*R*)-*N*-(3-(10,11-dihydro-5*H*-dibenzo[*a*,*d*][7]annulen-5-ylidene)propyl)-*N*-methyl-1-(5-oxo-2-phenyltetrahydrofuran-2-yl)methanesulfonamide

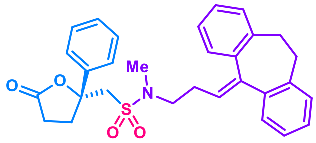

Compound 3q. (75% yield, 94% ee (*R*)). White solid, 75.2 mg at 0.20 mmol scale. The ee of **3q** was determined by HPLC analysis: (Chiralcel AD-H column, 1.0 mL/min, hexane/isopropanol = 70/30, 210 nm, 25 °C, t_{major} = 10.55 min (*R*), t_{minor} = 9.10 min (*S*)); $[\alpha]^{19}_D$ +4.1 (*c* 3.00, $CHCl_3$) for 94% ee (*R*). ^{1}H NMR (400 MHz, $CDCl_3$) δ 2.39 (t, J = 7.0 Hz, 2H), 2.45 – 2.54 (m, 1H), 2.54 – 2.64 (m, 1H), 2.70 (s, 3H), 2.73 – 2.88 (m, 2H), 2.89 – 3.04 (m, 1H), 3.12 – 3.27 (m, 3H), 3.27 – 3.54 (m, 4H), 5.83 (t, J = 7.4 Hz, 1H), 7.00 – 7.08 (m, 1H), 7.08 – 7.26 (m, 6H), 7.25 – 7.29 (m, 1H), 7.33 – 7.49 (m, 5H).^{13}C NMR (101 MHz, $CDCl_3$) δ 28.0, 28.4, 31.9, 32.5, 33.7, 34.1, 49.5, 59.0, 84.7, 124.5, 125.8, 126.1, 126.6, 127.3, 127.7, 127.9, 128.1, 128.5, 128.6, 129.0, 130.1, 137.0, 139.4, 139.7, 140.7, 142.3, 145.4, 175.5. HRMS (ESI) m/z calcd for $C_{30}H_{31}NNaO_4S^+$ $[M+Na]^+$ 524.1866, found 524.1868.

***N*-(3-((9*r*,10*R*)-9,10-ethanoanthracen-9(10*H*)-yl)propyl)-N-methyl-1-((*R*)-5-oxo-2-phenyltetrahydrofuran-2-yl)methanesulfonamide**

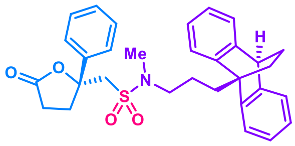

Compound 3r. (90% yield, 97% de). White solid, 92.8 mg at 0.20 mmol scale. The de of **3r** was determined by HPLC analysis: (Chiralcel AD-H column, 1.0 mL/min,

hexane/isopropanol = 70/30, 210 nm, 25 °C, t_{major} = 11.02 min, t_{minor} = 8.06 min); $[\alpha]^{19}_{D}$ +5.5 (*c* 1.80, $CHCl_3$) for 97% de, ^{1}H NMR (400 MHz, $CDCl_3$) δ 1.51 – 1.59 (m, 2H), 1.78 – 1.86 (m, 2H), 1.97 – 2.09 (m, 2H), 2.44 – 2.56 (m, 3H), 2.56 – 2.66 (m, 1H), 2.77 – 2.88 (m, 1H), 2.93 (s, 3H), 3.21 – 3.31 (m, 1H), 3.31 – 3.48 (m, 2H), 3.55 (d, *J* = 14.8 Hz, 1H), 3.61 (d, *J* = 14.8 Hz, 1H), 4.28 (t, *J* = 2.73 Hz, 1H), 7.04 – 7.15 (m, 4H), 7.16 – 7.22 (m, 2H), 7.24 – 7.27 (m, 2H), 7.35 – 7.46 (m, 5H). ^{13}C NMR (101 MHz, $CDCl_3$) δ 23.5, 27.6, 27.9, 28.4, 29.7, 32.6, 34.4, 44.5, 44.6, 50.9, 58.8, 84.9, 121.2, 123.4, 124.6, 125.4, 125.4, 128.6, 129.0, 142.4, 145.0, 145.1, 175.7. HRMS (ESI) m/z calcd for $C_{31}H_{33}NNaO_4S^+$ $[M+Na]^+$ 538.2023, found 538.2024.

***N*-methyl-*N*-((*S*)-3-(naphthalen-1-yloxy)-3-(thiophen-2-yl)propyl)-1-((*R*)-5-oxo-2-phenyl-tetrahydrofuran-2-yl)methanesulfonamide**

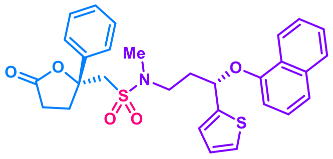

Compound 3s. (71% yield, 97% de). White solid, 76.1 mg at 0.20 mmol scale. The de of **3s** was determined by HPLC analysis: (Chiralcel AD-H column, 1.0 mL/min, hexane/isopropanol = 80/20, 210 nm, 25 °C, t_{major} = 21.46 min, t_{minor} = 25.48 min); $[\alpha]^{19}_{D}$ +24.3 (*c* 1.34, $CHCl_3$) for 97% de. ^{1}H NMR (400 MHz, $CDCl_3$) δ 2.29 – 2.40 (m, 1H), 2.44 – 2.62 (m, 3H), 2.68 – 2.79 (m, 1H), 2.87 (s, 3H), 3.07 – 3.18 (m, 1H), 3.36 – 3.56 (m, 4H), 5.72 – 5.80 (m, 1H), 6.87 (d, *J* = 8.0 Hz, 1H), 6.92 – 6.98 (m, 1H), 7.11 (d, *J* = 4.0 Hz, 1H), 7.20 – 7.25 (m, 1H), 7.28 – 7.46 (m, 7H), 7.47 – 7.56 (m, 2H), 7.75 – 7.86 (m, 1H), 8.28 – 8.37 (m, 1H). ^{13}C NMR (101 MHz, $CDCl_3$) δ 28.3, 32.6, 34.9, 37.8, 46.8, 58.8, 73.6, 84.7, 107.1, 120.9, 122.0, 124.5, 125.0, 125.1, 125.4, 125.7, 126.0, 126.4, 126.7, 127.6, 128.5, 128.9, 134.6, 142.1, 144.2, 152.9, 175.5. HRMS (ESI) m/z calcd for $C_{29}H_{29}NNaO_5S_2^+$ $[M+Na]^+$ 558.1379, found 558.1381.

(*R*)-5-((((3*S*,4*R*)-3-((benzo[*d*][1,3]dioxol-5-yloxy)methyl)-4-(4-fluorophenyl)pi-peridin-1-yl)sulfonyl) methyl)-5-phenyldihydrofuran-2(3*H*)-one

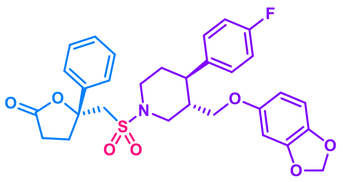

Compound 3t. (62% yield, 97% de). White solid, 70.4 mg at 0.20 mmol scale. The de of **3t** was determined by HPLC analysis: (Chiralcel AD-H column, 1.0 mL/min, hexane/isopropanol = 60/40, 210 nm, 25 °C, t_{major} = 26.77 min, t_{minor} = 20.52 min); $[\alpha]^{19}_{D}$ −29.3 (*c* 1.24, $CHCl_3$) for 97% de. ^{1}H NMR (400 MHz, $CDCl_3$) δ 1.80 – 1.93 (m, 2H), 2.10 – 2.26 (m, 1H), 2.45 – 2.71 (m, 3H), 2.75 – 2.96 (m, 3H), 3.17 – 3.28 (m, 1H), 3.40 – 3.57 (m, 2H), 3.58 – 3.67 (m, 2H), 3.79 – 3.90 (m, 1H), 4.00 – 4.10 (m, 1H), 5.91 (s, 2H), 6.16 (dd, *J* = 8.4, 2.4 Hz, 1H), 6.38 (d, *J* =

2.8 Hz, 1H), 6.65 (d, J = 8.0 Hz, 1H), 6.93 – 7.05 (m, 2H), 7.10 – 7.20 (m, 2H), 7.29 – 7.54 (m, 5H). ^{19}F NMR (376 MHz, $CDCl_3$) δ -115.7. ^{13}C NMR (101 MHz, $CDCl_3$) δ 28.4, 32.7, 33.6, 41.9, 43.3, 46.0, 48.9, 58.7, 68.5, 84.8, 98.1, 101.2, 105.7, 107.9, 115.7 (d, J = 21.2 Hz), 124.6, 128.6, 128.8 (d, J = 7.8 Hz), 129.0, 138.3 (d, J = 3.2 Hz,), 141.9, 142.1, 148.2, 154.0, 161.7 (d, J = 250.0 Hz), 175.5. HRMS (ESI) m/z calcd for $C_{30}H_{30}FNNaO_7S^+$ $[M+Na]^+$ 590.1619, found 590.1621.

(5*R*)-5-(((4-((5,6-dimethoxy-1-oxo-2,3-dihydro-1*H*-inden-2-yl)methyl)piperidin-1-yl)sulfonyl) methyl)-5-phenyldihydrofuran-2(3*H*)-one

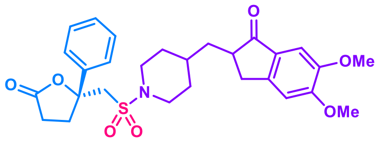

Compound 3u. (82% yield, 1:1 dr, 99% ee). White solid, 86.5 mg at 0.20 mmol scale. The ee of **3u** was determined by HPLC analysis: (Chiralcel AD-H column, 0.5 mL/min, hexane/isopropanol = 30/70, 210 nm, 25 °C, t_{major} = 65.88 min, 81.37 min, t_{minor} =31.90 min, 38.01 min); $[\alpha]^{18}_D$ −8.2 (c 1.71, $CHCl_3$) for 1:1 dr, 99% ee. ^{1}H NMR (400 MHz, $CDCl_3$) δ 1.20 – 1.37 (m, 4H), 1.67 – 1.77 (m, 2H), 1.77 – 1.87 (m, 1H), 2.33 – 2.83 (m, 7H), 3.09 – 3.25 (m, 2H), 3.33 – 3.49 (m, 2H), 3.57 – 3.77 (m, 2H), 3.83 (s, 3H), 3.89 (s, 3H), 6.79 (s, 1H), 7.09 (s, 1H), 7.24 – 7.30 (m, 1H), 7.31 – 7.42 (m, 4H). ^{13}C NMR (101 MHz, $CDCl_3$) δ 22.7, 28.4, 29.7, 31.5, 32.3, 32.36, 32.43, 33.4, 33.5, 33.8, 33.9, 38.3, 38.4, 44.9, 45.0, 45.7, 45.90, 45.93, 56.1, 56.3, 58.6, 84.7, 104.4, 107.4, 124.48, 124.49, 128.5, 129.0, 129.1, 129.2, 142.5, 148.6, 149.5, 155.6, 175.6, 207.19, 207.20. HRMS (ESI) m/z calcd for $C_{28}H_{33}NNaO_7S^+$ $[M+Na]^+$ 550.1870, found 550.1873.

(*R*)-4-(((5-oxo-2-phenyltetrahydrofuran-2-yl)methyl)sulfonyl)butanenitrile

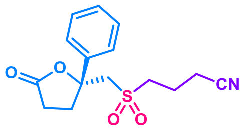

Compound 5a. (95% yield, 99% ee (*R*)). White solid, 58.4 mg at 0.20 mmol scale. The ee of **5a** was determined by HPLC analysis: (Chiralcel IB column, 1.0 mL/min, hexane/isopropanol = 50/50, 210 nm, 25 °C, t_{major} = 31.90 min (*R*), t_{minor} = 28.78 min (*S*)); $[\alpha]^{16}_D$ +1.8 (c 1.37, $CHCl_3$) for 99% ee (*R*). ^{1}H NMR (400 MHz, $CDCl_3$) δ 2.10 (p, J = 8.0 Hz, 2H), 2.32 – 2.60 (m, 4H), 2.60 – 2.72 (m, 1H), 2.92 – 3.01 (m, 1H), 3.03 – 3.17 (m, 2H), 3.51 – 3.65 (m, 2H), 7.22 – 7.50 (m, 5H). ^{13}C NMR (101 MHz, $CDCl_3$) δ 16.3, 18.2, 27.8, 33.1, 53.6, 62.4, 84.1, 118.1, 124.4, 129.0, 129.3, 141.2, 175.1. HRMS (ESI) m/z calcd for $C_{15}H_{17}NNaO_4S^+$ $[M+Na]^+$ 330.0770, found 330.0765.

(*R*)-4-(((2-([1,1'-biphenyl]-4-yl)-5-oxotetrahydrofuran-2-yl)methyl)sulfonyl) butanenitrile

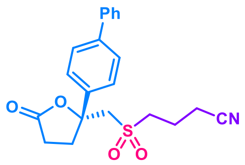

Compound 5b. (94% yield, 98% ee (*R*)). White solid, 72.1 mg at 0.20 mmol scale. The ee of **5b** was determined by HPLC analysis: (Chiralcel IA column, 1.0 mL/min, hexane/isopropanol = 50/50, 210 nm, 25 °C, t_{major} = 16.78 min (*R*), t_{minor} = 26.91 min (*S*)); $[\alpha]^{17}_{D}$ −13.3 (*c* 1.31, $CHCl_3$) for 98% ee (*R*). ^{1}H NMR (400 MHz, $CDCl_3$) δ 2.22 (p, *J* = 7.23 Hz, 2H), 2.45 – 2.87 (m, 5H), 3.03 – 3.33 (m, 3H), 3.62 – 3.84 (m, 2H), 7.35 – 7.44 (m, 1H), 7.43 – 7.53 (m, 4H), 7.56 – 7.64 (m, 2H), 7.69 (d, *J* = 8.0 Hz, 2H). ^{13}C NMR (101 MHz, $CDCl_3$) δ 16.3, 18.2, 27.9, 33.2, 53.7, 62.3, 84.1, 118.2, 124.9, 127.1, 127.87, 127.94, 129.0, 139.8, 140.1, 141.9, 175.2. HRMS (ESI) m/z calcd for $C_{21}H_{21}NNaO_4S^+$ $[M+Na]^+$ 406.1083, found 406.1083.

(*R*)-4-(((2-(naphthalen-2-yl)-5-oxotetrahydrofuran-2-yl)methyl)sulfonyl) butanenitrile

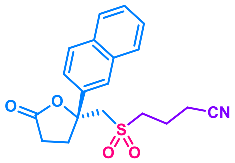

Compound 5c. (92% yield, 98% ee (*R*)). White solid, 65.8 mg at 0.20 mmol scale. The ee of **5c** was determined by HPLC analysis: (Chiralcel IA column, 1.0 mL/min, hexane/isopropanol = 60/40, 210 nm, 25 °C, t_{major} = 15.80 min (*R*), t_{minor} = 13.91 min (*S*)); $[\alpha]^{15}_{D}$ −10.0 (*c* 1.26, $CHCl_3$) for 98% ee (*R*). ^{1}H NMR (400 MHz, $CDCl_3$) δ 2.16 (p, *J* = 7.2 Hz, 2H), 2.42 – 2.60 (m, 3H), 2.63 – 2.81 (m, 2H), 3.03 – 3.15 (m, 1H), 3.15 – 3.31 (m, 2H), 3.73 (s, 2H), 7.39 (dd, *J* = 8.6, 2.0 Hz, 1H), 7.49 – 7.60 (m, 2H), 7.81 – 7.97 (m, 4H). ^{13}C NMR (101 MHz, $CDCl_3$) δ 16.4, 18.2, 27.9, 33.2, 53.8, 62.2, 84.3, 118.3, 122.0, 123.5, 127.30, 127.34, 127.8, 128.5, 129.6, 132.9, 133.0, 138.3, 175.4. HRMS (ESI) m/z calcd for $C_{19}H_{19}NNaO_4S^+$ $[M+Na]^+$ 380.0927, found 380.0928.

(*R*)-4-(((5-oxo-2-(pyren-1-yl)tetrahydrofuran-2-yl)methyl)sulfonyl)butanenitrile

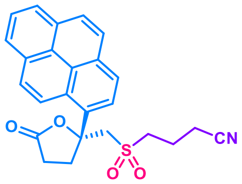

Compound 5d. (65% yield, 85% ee (*R*)). White solid, 56.1 mg at 0.20 mmol scale. The ee of **5d** was determined by HPLC analysis: (Chiralcel AD-H column, 1.0 mL/min, hexane/isopropanol = 60/40, 210 nm, 25 °C, t_{major} = 29.32 min (*R*), t_{minor} = 17.34 min (*S*)); $[\alpha]^{15}_{D}$ +18.9 (*c* 0.66, $CHCl_3$) for 85% ee (*R*). ^{1}H NMR (400 MHz, $CDCl_3$) δ 2.00 – 2.20 (m, 2H), 2.36 – 2.60 (m, 3H), 2.82 – 2.94 (m, 1H), 2.94 – 3.29 (m, 3H), 3.60 – 3.73 (m, 1H), 3.80 (d, *J* = 16.0 Hz, 1H), 4.22 (d, *J* = 16.0 Hz, 1H), 7.82 – 8.38 (m, 9H). ^{13}C NMR (101 MHz, $CDCl_3$) δ 16.3, 18.2, 28.2, 33.2, 53.9, 62.0, 85.3, 118.1, 122.1, 122.5, 124.7, 125.0, 125.8, 126.0, 126.5, 126.7, 127.3, 128.4, 128.9, 130.0, 131.3, 132.0, 134.6, 175.2. HRMS (ESI) m/z calcd for $C_{25}H_{21}NNaO_4S^+$ $[M+Na]^+$ 454.1083, found 454.1082.

(*R*)-4-(((5-oxo-2-(o-tolyl)tetrahydrofuran-2-yl)methyl)sulfonyl)butanenitrile

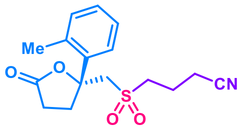

Compound 5e. (45% yield, 97% ee (*R*)). Colorless oil, 28.9 mg at 0.20 mmol scale. The ee of **5e** was determined by HPLC analysis: (Chiralcel IB column, 1.0 mL/min, hexane/isopropanol = 60/40, 210 nm, 25 °C, t_{major} = 25.70 min (*R*), t_{minor} = 22.41 min (*S*)); $[\alpha]^{15}_{D}$ +14.5 （*c* 0.20, $CHCl_3$) for 97% ee (*R*). ^1H NMR (400 MHz, $CDCl_3$) δ 2.22 (p, *J* = 7.20 Hz, 2H), 2.48 (s, 3H), 2.49 – 2.73 (m, 4H), 2.77 – 2.89 (m, 1H), 3.08 – 3.18 (m, 1H), 3.21 – 3.32 (m, 2H), 3.68 (d, *J* = 15.6 Hz, 1H), 3.79 (d, *J* = 15.6 Hz, 1H), 7.24 – 7.36 (m, 4H), 7.52 (dd, *J* = 7.8, 2.0 Hz, 1H). ^{13}C NMR (151 MHz, $CDCl_3$) δ 16.3, 18.2, 21.5, 27.9, 32.5, 53.8, 61.1, 84.5, 118.0, 124.4, 126.7, 129.0, 133.0, 133.1, 139.6, 175.1. HRMS (ESI) m/z calcd for $C_{16}H_{19}NNaO_4S^+$ $[M+Na]^+$ 344.0927, found 344.0926.

(*R*)-4-(((5-oxo-2-(m-tolyl)tetrahydrofuran-2-yl)methyl)sulfonyl)butanenitrile

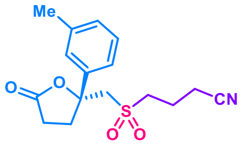

Compound 5f. (96% yield, 99% ee (*R*)). Colorless oil, 61.7 mg at 0.20 mmol scale. The ee of **5f** was determined by HPLC analysis: (Chiralcel IB column, 1.0 mL/min, hexane/isopropanol = 60/40, 210 nm, 25 °C, t_{major} = 23.57 min (*R*), t_{minor} = 22.63 min (*S*)); $[\alpha]^{15}_{D}$ +5.3 (*c* 1.06, $CHCl_3$) for 99% ee (*R*). ^1H NMR (400 MHz, $CDCl_3$) δ 2.10 – 2.20 (m, 2H), 2.37 (s, 3H), 2.44 – 2.63 (m, 4H), 2.65 – 2.75 (m, 1H), 2.96 – 3.08 (m, 1H), 3.09 – 3.28 (m, 2H), 3.54 – 3.67 (m, 2H), 7.12 – 7.20 (m, 3H), 7.27 – 7.33 (m, 1H). ^{13}C NMR (101 MHz, $CDCl_3$) δ 16.3, 18.2, 21.6, 27.9, 33.0, 53.6, 62.4, 84.1, 118.2, 121.4, 125.0, 129.1, 129.7, 139.2, 141.2, 175.3. HRMS (ESI) m/z calcd for $C_{16}H_{19}NNaO_4S^+$ $[M+Na]^+$ 344.0927, found 344.0923.

(*R*)-4-(((2-(4-(*tert*-butyl)phenyl)-5-oxotetrahydrofuran-2-yl)methyl)sulfonyl) butanenitrile

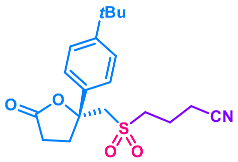

Compound 5g. (82% yield, 99% ee (*R*)). Colorless oil, 59.6 mg at 0.20 mmol scale. The ee of **5g** was determined by HPLC analysis: (Chiralcel AD-H column, 1.0 mL/min, hexane/isopropanol = 80/20, 210 nm, 25 °C, t_{major} = 19.99 min (*R*), t_{minor} = 18.20 min (*S*)); $[\alpha]^{15}_{D}$ −3.3 (*c* 1.35, $CHCl_3$) for 99% ee (*R*). ^1H NMR (400 MHz, $CDCl_3$) δ 1.34 (s, 9H), 2.18 (p, *J* = 7.29 Hz, 2H), 2.43 – 2.79 (m, 5H), 2.98 – 3.29 (m, 3H), 3.59 – 3.72 (m, 2H), 7.33 (d, *J* = 8.0 Hz, 2H), 7.46 (d, *J* = 8.0 Hz, 2H). ^{13}C NMR (101 MHz, $CDCl_3$) δ 16.3, 18.2, 27.9, 31.2, 32.8, 34.7, 53.6, 62.3, 84.2,

118.2, 124.2, 126.1, 138.3, 152.2, 175.3. HRMS (ESI) m/z calcd for $C_{19}H_{25}NNaO_4S^+$ $[M+Na]^+$ 386.1397, found 386.1395.

(*R*)-4-(((2-(3-methoxyphenyl)-5-oxotetrahydrofuran-2-yl)methyl)sulfonyl)butanenitrile

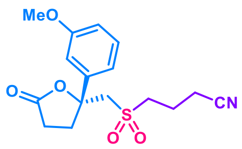

Compound 5h. (82% yield, 97% ee (*R*)). Colorless oil, 55.3 mg at 0.20 mmol scale. The ee of **5h** was determined by HPLC analysis: (Chiralcel OD-H column, 1.0 mL/min, hexane/isopropanol = 60/40, 210 nm, 25 °C, t_{major} =48.29 min (*R*), t_{minor} = 44.58 min (*S*)); $[\alpha]^{15}_D$ +8.0 (*c* 0.85, $CHCl_3$) for 97% ee (*R*). ^1H NMR (400 MHz, $CDCl_3$) δ 2.10 (p, *J* = 7.28 Hz, 2H), 2.34 – 2.59 (m, 4H), 2.60 – 2.71 (m, 1H), 2.88 – 3.19 (m, 3H), 3.45 – 3.67 (m, 2H), 3.76 (s, 3H), 6.71 – 6.93 (m, 3H), 7.29 (t, *J* = 7.8 Hz, 1H). ^{13}C NMR (101 MHz, $CDCl_3$) δ 16.4, 18.2, 27.9, 33.2, 53.7, 55.5, 62.3, 84.0, 110.7, 113.9, 116.6, 118.3, 130.5, 143.0, 160.2, 175.2. HRMS (ESI) m/z calcd for $C_{16}H_{19}NNaO_5S^+$ $[M+Na]^+$ 360.0876, found 360.0873.

(*R*)-4-(((2-(3-fluorophenyl)-5-oxotetrahydrofuran-2-yl)methyl)sulfonyl)butanenitrile

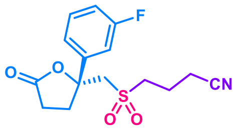

Compound 5i. (94% yield, 99% ee (*R*)). Colorless oil, 61.2 mg at 0.20 mmol scale. The ee of **5i** was determined by HPLC analysis: (Chiralcel AD-H column, 1.0 mL/min, hexane/isopropanol = 70/30, 210 nm, 25 °C, t_{major} = 14.58 min (*R*), t_{minor} = 12.70 min (*S*)); $[\alpha]^{18}_D$ +8.8 (*c* 1.70, $CHCl_3$) for 99% ee (*R*). ^1H NMR (400 MHz, $CDCl_3$) δ 2.18 (p, *J* = 8.0 Hz, 2H), 2.43 – 2.63 (m, 4H), 2.70 – 2.85 (m, 1H), 3.03 – 3.31 (m, 3H), 3.58 – 3.76 (m, 2H), 7.02 – 7.24 (m, 3H), 7.36 – 7.51 (m, 1H). ^{19}F NMR (376 MHz, $CDCl_3$) δ -110.3. ^{13}C NMR (101 MHz, $CDCl_3$) δ 16.3, 18.1, 27.7, 33.5, 53.7, 61.9, 83.6 (d, *J* = 2.0 Hz), 112.0 (d, *J* = 23.6 Hz), 115.9 (d, *J* = 21.2 Hz), 118.3, 120.2 (d, *J* = 3.0 Hz), 131.0 (d, *J* = 8.2 Hz), 144.0 (d, *J* = 6.8 Hz), 162.9 (d, *J* = 249.2 Hz), 174.9. HRMS (ESI) m/z calcd for $C_{15}H_{16}FNNaO_4S^+$ $[M+Na]^+$ 348.0676, found 348.0669.

(*R*)-4-(((2-(4-fluorophenyl)-5-oxotetrahydrofuran-2-yl)methyl)sulfonyl)butanenitrile

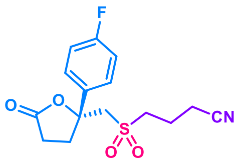

Compound 5j. (96% yield, 98% ee (*R*)). White solid, 62.5 mg at 0.20 mmol scale. The ee of **5j** was determined by HPLC analysis: (Chiralcel AD-H column, 1.0 mL/min, hexane/isopropanol = 60/40, 210 nm, 25 °C, t_{major} = 11.90 min (*R*), t_{minor} = 10.16 min (*S*)); $[\alpha]^{17}_D$ +4.8 (*c* 1.58, $CHCl_3$) for 98% ee (*R*). ^1H NMR (400 MHz, $CDCl_3$) δ 2.20

(p, J = 7.2 Hz, 2H), 2.41 – 2.69 (m, 4H), 2.70 – 3.82 (m, 1H), 3.03 – 3.30 (m, 3H), 3.56 – 3.74 (m, 2H), 7.08 – 7.19 (m, 2H), 7.32 – 7.47 (m, 2H). ^{19}F NMR (376 MHz, $CDCl_3$) δ -112.2. ^{13}C NMR (101 MHz, $CDCl_3$) δ 16.3, 18.1, 27.8, 33.4, 53.7, 62.4, 83.8, 116.3 (d, J = 21.9 Hz), 118.2, 126.5 (d, J = 8.3 Hz), 137.1 (d, J = 3.3 Hz), 162.7 (d, J = 250.4 Hz), 174.9. HRMS (ESI) m/z calcd for $C_{15}H_{16}FNNaO_4S^+$ $[M+Na]^+$ 348.0676, found 348.0673.

(*R*)-4-(((2-(4-iodophenyl)-5-oxotetrahydrofuran-2-yl)methyl)sulfonyl)butanenitrile

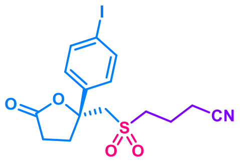

Compound 5k. (96% yield, 98% ee (*R*)). Colorless oil, 83.2 mg at 0.20 mmol scale. The ee of **5k** was determined by HPLC analysis: (Chiralcel IB column, 1.0 mL/min, hexane/isopropanol = 50/50, 210 nm, 25 °C, t_{major} = 37.27 min (*R*), t_{minor} = 32.18 min (*S*)); $[\alpha]^{17}_D$ −8.2 (*c* 1.64, $CHCl_3$) for 98% ee (*R*). ^{1}H NMR (400 MHz, $CDCl_3$) δ 2.16 (p, J = 7.22 Hz, 2H), 2.39 – 2.60 (m, 4H), 2.66 – 2.78 (m, 1H), 3.05 – 3.25 (m, 3H), 3.53 – 3.70 (m, 2H), 7.03 – 7.16 (m, 2H), 7.67 – 7.80 (m, 2H). ^{13}C NMR (101 MHz, $CDCl_3$) δ 16.3, 18.1, 27.7, 33.4, 53.8, 62.0, 83.8, 94.8, 118.2, 126.4, 138.3, 141.1, 174.9. HRMS (ESI) m/z calcd for $C_{15}H_{16}INNaO_4S^+$ $[M+Na]^+$ 455.9737, found 455.9736.

(*R*)-4-(((4,4-dimethyl-5-oxo-2-phenyltetrahydrofuran-2-yl)methyl)sulfonyl)butanenitrile

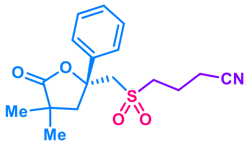

Compound 5l. (95% yield, 81% ee (*R*)). White solid, 63.7 mg at 0.20 mmol scale. The ee of **5l** was determined by HPLC analysis: (Chiralcel AD-H column, 1.0 mL/min, hexane/isopropanol = 70/30, 210 nm, 25 °C, t_{major} = 14.25 min (*R*), t_{minor} = 9.87 min (*S*)); $[\alpha]^{15}_D$ −11.6 (*c* 1.19, $CHCl_3$) for 81% ee (*R*). ^{1}H NMR (400 MHz, $CDCl_3$) δ 0.95 (s, 3H), 1.37 (s, 3H), 2.20 (p, J = 8.0 Hz, 2H), 2.49 – 2.69 (m, 3H), 3.07 – 3.30 (m, 3H), 3.49 – 3.67 (m, 2H), 7.33 – 7.51 (m, 5H). ^{13}C NMR (101 MHz, $CDCl_3$) δ 16.4, 18.2, 25.4, 26.3, 40.3, 46.9, 54.1, 63.1, 118.3, 124.3, 128.9, 129.4, 142.6, 180.5. HRMS (ESI) m/z calcd for $C_{17}H_{21}NNaO_4S^+$ $[M+Na]^+$ 358.1083, found 358.1082.

(*R*)-4-(((5-oxo-2-(phenylethynyl)tetrahydrofuran-2-yl)methyl)sulfonyl) butanenitrile

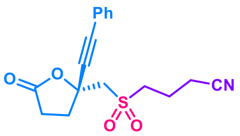

Compound 5m. (65% yield, 97% ee (*R*)). Colorless oil, 43.1 mg at 0.20 mmol scale. The ee of **5m** was determined by HPLC analysis: (Chiralcel IA column, 1.0 mL/min, hexane/isopropanol

= 50/50, 210 nm, 25 °C, t_{major} = 9.64 min (*R*), t_{minor} = 8.68 min (*S*)); $[\alpha]^{15}_{D}$ −14.4 (*c* 0.10, $CHCl_3$) for 97% ee (*R*). ^{1}H NMR (400 MHz, $CDCl_3$) δ 2.29 (p, *J* = 7.24 Hz, 2H), 2.53 – 2.68 (m, 2H), 2.69 – 2.81 (m, 2H), 2.84 – 2.99 (m, 2H), 3.34 – 3.47 (m, 2H), 3.67 – 3.81 (m, 2H), 7.32 – 7.45 (m, 3H), 7.45 – 7.52 (m, 2H). ^{13}C NMR (101 MHz, $CDCl_3$) δ 16.4, 18.3, 28.3, 34.9, 53.6, 61.1, 76.1, 84.9, 89.1, 118.2, 120.5, 128.7, 130.0, 132.0, 174.6. HRMS (ESI) m/z calcd for $C_{17}H_{17}NNaO_4S^+$ $[M+Na]^+$ 354.0770, found 354.0763.

***tert*-butyl (*R*)-3-(cyanomethyl)-3-((((5-oxo-2-phenyltetrahydrofuran-2-yl)methyl) sulfonyl)methyl) azetidine-1-carboxylate**

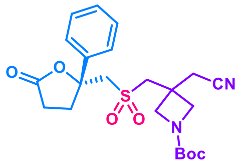

Compound 5n. (84% yield, 98% ee (*R*)). Colorless oil, 75.4 mg at 0.20 mmol scale. The ee of **5n** was determined by HPLC analysis: (Chiralcel OD-H column, 1.0 mL/min, hexane/isopropanol = 50/50, 210 nm, 25 °C, t_{major} = 59.32 min (*R*), t_{minor} = 51.76 min (*S*)); $[\alpha]^{18}_{D}$ +12.9 (*c* 2.14, $CHCl_3$) for 98% ee (*R*). ^{1}H NMR (400 MHz, $CDCl_3$) δ 1.46 (s, 9H), 2.46 – 2.58 (m, 1H), 2.62 – 2.81 (m, 2H), 2.97 – 3.20 (m, 3H), 3.27 (d, *J* = 13.97 Hz, 1H), 3.52 (d, *J* = 13.90 Hz, 1H), 3.63 – 3.75 (m, 2H), 3.75 – 3.86 (m, 2H), 3.89 – 4.04 (m, 2H), 7.38 – 7.52 (m, 5H). ^{13}C NMR (101 MHz, $CDCl_3$) δ 26.0, 27.8, 28.4, 33.3, 33.4, 58.5, 59.2, 64.7, 80.6, 84.0, 116.6, 124.6, 129.2, 129.4, 140.8, 155.9, 175.0. HRMS (ESI) m/z calcd for $C_{22}H_{28}N_2NaO_6S^+$ $[M+Na]^+$ 471.1560, found 471.1563.

***tert*-butyl (*R*)-4-(cyanomethyl)-4-((((5-oxo-2-phenyltetrahydrofuran-2-yl)methyl) sulfonyl) methyl) piperidine-1-carboxylate**

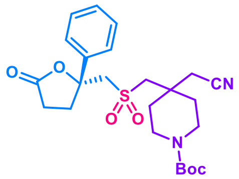

Compound 5o. (93% yield, 94% ee (*R*)). Colorless oil, 88.6 mg at 0.20 mmol scale. The ee of **5o** was determined by HPLC analysis: (Chiralcel IA column, 1.0 mL/min, hexane/isopropanol = 50/50, 210 nm, 25 °C, t_{major} = 9.71 min (*R*), t_{minor} = 7.19 min (*S*)); $[\alpha]^{19}_{D}$ −7.3 (*c* 2.03, $CHCl_3$) for 94% ee (*R*). ^{1}H NMR (400 MHz, $CDCl_3$) δ 1.39 (s, 9H), 1.46 – 1.61 (m, 2H), 1.67 – 1.87 (m, 2H), 2.33 – 2.50 (m, 1H), 2.49 – 2.60 (m, 1H), 2.61 – 2.77 (m, 2H), 2.80 – 2.99 (m, 2H), 3.00 – 3.34 (m, 4H), 3.36 – 3.82 (m, 4H), 7.24 – 7.46 (m, 5H). ^{13}C NMR (101 MHz, $CDCl_3$) δ 27.4, 27.9, 28.5, 33.3, 34.3, 34.7, 36.1, 57.9, 65.2, 80.1, 84.1, 117.1, 124.6, 129.1, 129.4, 141.2, 154.6, 175.2. HRMS (ESI) m/z calcd for $C_{24}H_{32}N_2NaO_6S^+$ $[M+Na]^+$ 499.1873, found 499.1873.

(*R*)-5-phenyl-5-(tosylmethyl)dihydrofuran-2(3*H*)-one

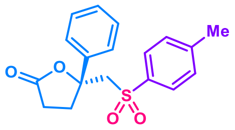

Compound 7a. (95% yield, >99.5% ee (*R*)). White solid, 62.8 mg at 0.20 mmol scale. The ee of **7a** was determined by HPLC analysis: (Chiralcel AD-H column, 1.0 mL/min, hexane/isopropanol = 90/10, 210 nm, 25 °C, t_{major} = 48.06 min (*R*), t_{minor} = 42.32 min (*S*)); $[\alpha]^{25}_{D}$ + 8.5 (*c* 0.90, $CHCl_3$) for >99.5% ee (*R*) [ref. 12: $[\alpha]^{24}_{D}$ = −3.2 (c = 1.50, $CHCl_3$) for 74% ee (*S*)]. ^1H NMR (400 MHz, $CDCl_3$) δ 2.44 (s, 3H), 2.45 – 2.56 (m, 1H), 2.59 – 2.70 (m, 1H), 2.80 – 3.91 (m, 1H), 3.32 – 3.38 (m, 1H), 3.69 – 3.82 (m, 2H), 7.25 – 7.41 (m, 7H), 7.71 (d, *J* = 8.2 Hz, 2H). ^{13}C NMR (101 MHz, $CDCl_3$) δ 21.7, 28.3, 32.5, 65.1, 84.8, 124.5, 127.9, 128.5, 128.9, 129.9, 137.6, 141.9, 145.0, 175.4. HRMS (ESI) m/z calcd for $C_{18}H_{18}NaO_4S^+$ $[M+Na]^+$ 353.0818, found 353.0815.

(*R*)-5-([1,1'-biphenyl]-4-yl)-5-(tosylmethyl)dihydrofuran-2(3*H*)-one

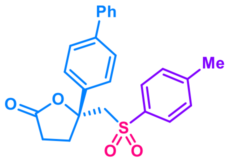

Compound 7b. (87% yield, 99% ee (*R*)). White solid, 70.7 mg at 0.20 mmol scale. The ee of **7b** was determined by HPLC analysis: (Chiralcel OD-H column, 1.0 mL/min, hexane/isopropanol = 90/10, 210 nm, 25 °C, t_{major} = 88.98 min (*R*), t_{minor} = 104.66 min (*S*)); $[\alpha]^{16}_{D}$ −25.9 (*c* 1.39, $CHCl_3$) for 99% ee (*R*). ^1H NMR (400 MHz, $CDCl_3$) δ 2.42 (s, 3H), 2.51 – 2.61 (m, 1H), 2.66 – 2.77 (m, 1H), 2.83 – 2.95 (m, 1H), 3.32 – 3.44 (m, 1H), 3.82 (s, 2H), 7.30 (d, *J* = 8.0 Hz, 2H), 7.35 – 7.43 (m, 3H), 7.43 – 7.50 (m, 2H), 7.52 – 7.59 (m, 4H), 7.66 – 7.76 (m, 2H). ^{13}C NMR (101 MHz, $CDCl_3$) δ 21.6, 28.3, 32.5, 65.1, 84.7, 125.2, 127.1, 127.5, 127.8, 127.9, 128.9, 129.9, 137.5, 140.0, 140.5, 141.5, 145.0, 175.3. HRMS (ESI) m/z calcd for $C_{24}H_{22}NaO_4S^+$ $[M+Na]^+$ 429.1131, found 429.1132.

(*R*)-5-(naphthalen-2-yl)-5-(tosylmethyl)dihydrofuran-2(3*H*)-one

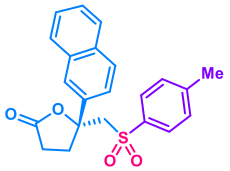

Compound 7c. (91% yield, 98% ee (*R*)). White solid, 69.2 mg at 0.20 mmol scale. The ee of **7c** was determined by HPLC analysis: (Chiralcel AS-H column, 1.0 mL/min, hexane/isopropanol = 50/50, 210 nm, 25 °C, t_{major} = 50.17 min (*R*), t_{minor} = 62.00 min (*S*)); $[\alpha]^{15}_{D}$ −24.1 (*c* 1.32, $CHCl_3$) for 98% ee (*R*). ^1H NMR (400 MHz, $CDCl_3$) δ 2.34 (s, 3H), 2.47 – 2.59 (m, 1H), 2.69 – 2.81 (m, 1H), 2.82 – 2.94 (m, 1H), 3.33 – 3.44 (m, 1H), 3.82 – 3.94 (m, 2H), 7.18 (d, *J* = 8.0 Hz, 2H), 7.34 (dd, *J* = 8.6, 2.0 Hz, 1H), 7.49 – 7.56 (m, 2H), 7.65 (d, *J* = 8.0 Hz, 2H), 7.73 – 7.86 (m, 4H). ^{13}C NMR (101 MHz, $CDCl_3$) δ 21.7, 28.3, 32.5, 64.9, 84.9, 122.3, 123.8, 127.0, 127.6, 128.0, 128.4, 129.2,

129.8, 132.8, 132.9, 137.3, 138.5, 145.0, 175.6. HRMS (ESI) m/z calcd for $C_{22}H_{20}NaO_4S^+$ [M+Na]$^+$ 403.0975, found 403.0975.

(*R*)-5-(pyren-1-yl)-5-(tosylmethyl)dihydrofuran-2(3*H*)-one

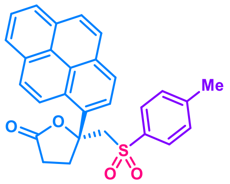

Compound 7d. (95% yield, 92% ee (*R*)). Light pink solid, 86.4 mg at 0.20 mmol scale. The ee of **7d** was determined by HPLC analysis: (Chiralcel AD-H column, 1.0 mL/min, hexane/isopropanol = 80/20, 210 nm, 25 °C, t_{major} = 25.80 min (*R*), t_{minor} = 31.72 min (*S*)); $[\alpha]^{15}_D$ −76.0 (*c* 2.11, $CHCl_3$) for 92% ee (*R*). ^{1}H NMR (400 MHz, $CDCl_3$) δ 1.84 (s, 3H), 2.46 – 2.66 (m, 1H), 2.83 – 3.18 (m, 2H), 3.54 – 3.83 (m, 1H), 3.92 (d, *J* = 15.4 Hz, 1H), 4.35 (d, *J* = 15.4 Hz, 1H), 6.25 – 7.00 (m, 2H), 7.19 – 7.51 (m, 2H), 7.87 – 8.24 (m, 9H). ^{13}C NMR (101 MHz, $CDCl_3$) δ 21.1, 28.4, 33.2, 64.0, 85.9, 122.9, 123.1, 124.6, 124.7, 125.6, 125.8, 126.1, 126.4, 127.1, 127.23, 127.27, 128.2, 128.6, 129.19, 129.24, 129.5, 131.2, 132.0, 144.4, 175.3. HRMS (ESI) m/z calcd for $C_{28}H_{22}NaO_4S^+$ [M+Na]$^+$ 477.1131, found 477.1132.

(*R*)-5-(o-tolyl)-5-(tosylmethyl)dihydrofuran-2(3*H*)-one

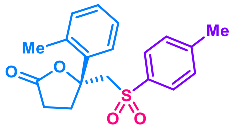

Compound 7e. (94% yield, 98% ee (*R*)). White solid, 64.8 mg at 0.20 mmol scale. The ee of **7e** was determined by HPLC analysis: (Chiralcel AD-H column, 1.0 mL/min, hexane/isopropanol = 90/10, 210 nm, 25 °C, t_{major} = 41.08 min (*R*), t_{minor} = 35.93 min (*S*)); $[\alpha]^{16}_D$ +42.7 (*c* 1.82, $CHCl_3$) for 98% ee (*R*). ^{1}H NMR (400 MHz, $CDCl_3$) δ 2.41 (s, 3H), 2.45 (s, 3H), 2.48 – 2.61 (m, 1H), 2.61 – 2.73 (m, 1H), 2.93 – 3.07 (m, 1H), 3.44 – 3.55 (m, 1H), 3.72 (d, *J* = 15.2 Hz, 1H), 3.85 (d, *J* = 15.2 Hz, 1H), 7.10 – 7.27 (m, 3H), 7.33 (d, *J* = 8.0 Hz, 2H), 7.45 (d, *J* = 7.6 Hz, 1H), 7.74 (d, *J* = 8.0 Hz, 2H). ^{13}C NMR (101 MHz, $CDCl_3$) δ 21.3, 21.8, 28.5, 32.0, 63.4, 85.4, 124.9, 126.4, 127.9, 128.7, 130.0, 132.8, 133.3, 137.5, 140.3, 145.2, 175.6. HRMS (ESI) m/z calcd for $C_{19}H_{20}NaO_4S^+$ [M+Na]$^+$ 367.0975, found 367.0971.

(*R*)-5-(m-tolyl)-5-(tosylmethyl)dihydrofuran-2(3*H*)-one

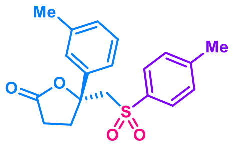

Compound 7f. (95% yield, 99% ee (*R*)). White solid, 65.4 mg at 0.20 mmol scale. The ee of **7f** was determined by HPLC analysis: (Chiralcel OD-H column, 1.0 mL/min, hexane/isopropanol = 80/20, 210 nm, 25 °C, t_{major} = 19.82 min (*R*), t_{minor} = 23.99 min (*S*)); $[\alpha]^{16}_D$ +7.2 (*c* 1.55, $CHCl_3$) for 99% ee (*R*). ^{1}H NMR (400 MHz, $CDCl_3$) δ 2.32 (s, 3H), 2.44 (s, 3H), 2.47 – 2.57 (m, 1H), 2.58 – 2.69 (m, 1H), 2.79 – 2.91 (m, 1H), 3.31 – 3.42 (m, 1H), 3.69 – 3.80 (m, 2H), 7.11 (d, *J* = 6.55 Hz, 3H), 7.17 – 7.26 (m,

1H), 7.27 – 7.35 (m, 2H), 7.71 (d, J = 8.0 Hz, 2H). ^{13}C NMR (101 MHz, $CDCl_3$) δ 21.5, 21.8, 28.4, 32.5, 65.1, 84.9, 121.6, 125.2, 128.0, 128.9, 129.3, 129.9, 137.6, 138.8, 141.9, 145.0, 175.6. HRMS (ESI) m/z calcd for $C_{19}H_{20}NaO_4S^+$ $[M+Na]^+$ 367.0975, found 367.0971.

(*R*)-5-(4-(*tert*-butyl)phenyl)-5-(tosylmethyl)dihydrofuran-2(3*H*)-one

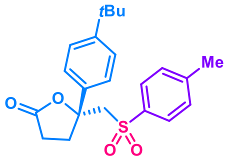

Compound 7g. (79% yield, 99% ee (*R*)). White solid, 61.1 mg at 0.20 mmol scale. The ee of **7g** was determined by HPLC analysis: (Chiralcel IA column, 1.0 mL/min, hexane/isopropanol = 90/10, 210 nm, 25 °C, t_{major} = 24.41 min (*R*), t_{minor} = 28.82 min (*S*)); $[\alpha]^{15}_{D}$ −11.8 (*c* 1.37, $CHCl_3$) for 99% ee (*R*). ^{1}H NMR (400 MHz, $CDCl_3$) δ 1.30 (s, 9H), 2.43 (s, 3H), 2.45 – 2.58 (m, 1H), 2.61 – 2.71 (m, 1H), 2.80 – 2.90 (m, 1H), 3.30 – 3.42 (m, 1H), 3.70 – 3.84 (m, 2H), 7.23 (d, J = 8.0 Hz, 2H), 7.27 – 7.36 (m, 4H), 7.69 (d, J = 8.0 Hz, 2H). ^{13}C NMR (101 MHz, $CDCl_3$) δ 21.8, 28.4, 31.3, 32.3, 34.6, 65.2, 84.9, 124.4, 125.8, 128.0, 129.9, 137.6, 138.8, 144.9, 151.7, 175.6. HRMS (ESI) m/z calcd for $C_{22}H_{26}NaO_4S^+$ $[M+Na]^+$ 409.1444, found 409.1442.

(*R*)-5-(3-methoxyphenyl)-5-(tosylmethyl)dihydrofuran-2(3*H*)-one

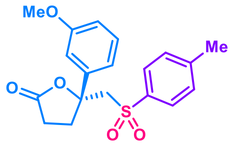

Compound 7h. (86% yield, 99% ee (*R*)). White solid, 62.0 mg at 0.20 mmol scale. The ee of **7h** was determined by HPLC analysis: (Chiralcel OD-H column, 1.0 mL/min, hexane/isopropanol = 80/20, 210 nm, 25 °C, t_{major} = 30.20 min (*R*), t_{minor} = 38.01 min (*S*)); $[\alpha]^{15}_{D}$ +9.4 (*c* 1.48, $CHCl_3$) for 99% ee (*R*). ^{1}H NMR (400 MHz, $CDCl_3$) δ 2.34 (s, 3H), 2.36 – 2.47 (m, 1H), 2.48 – 2.58 (m, 1H), 2.70 – 2.81 (m, 1H), 3.20 – 3.31 (m, 1H), 3.48 – 3.83 (m, 5H), 6.65 – 6.87 (m, 3H), 7.13 – 7.24 (m, 3H), 7.56 – 7.67 (m, 2H). ^{13}C NMR (101 MHz, $CDCl_3$) δ 21.8, 28.3, 32.6, 55.4, 65.0, 84.7, 110.5, 113.8, 116.8, 128.0, 130.0, 130.2, 137.5, 143.7, 145.1, 159.9, 175.5. HRMS (ESI) m/z calcd for $C_{19}H_{20}NaO_5S^+$ $[M+Na]^+$ 383.0924, found 383.0921.

(*R*)-5-(3-fluorophenyl)-5-(tosylmethyl)dihydrofuran-2(3*H*)-one

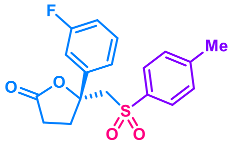

Compound 7i. (87% yield, 99% ee (*R*)). White solid, 60.6 mg at 0.20 mmol scale. The ee of **7i** was determined by HPLC analysis: (Chiralcel OD-H column, 1.0 mL/min, hexane/isopropanol = 80/20, 210 nm, 25 °C, t_{major} = 26.17 min (*R*), t_{minor} = 30.34 min (*S*)); $[\alpha]^{16}_{D}$ +12.1 (*c* 1.43, $CHCl_3$) for 99% ee (*R*). ^{1}H NMR (400 MHz, $CDCl_3$) δ 2.44 (s, 3H), 2.47 – 2.67 (m, 2H), 2.80 – 2.94 (m, 1H), 3.27 – 3.41 (m, 1H), 3.67 – 3.82 (m, 2H), 6.90 – 7.08 (m, 2H), 7.08 – 7.16 (m, 1H), 7.29 – 7.39 (m, 3H), 7.61 – 7.79 (m,

2H). ^{19}F NMR (376 MHz, $CDCl_3$) δ -111.0. ^{13}C NMR (101 MHz, $CDCl_3$) δ 21.6, 28.1, 32.7, 64.8, 84.1, 112.1 (d, J = 23.2 Hz), 115.5 (d, J = 21.2 Hz), 120.3 (d, J = 3.2 Hz), 127.9, 129.9, 130.7 (d, J = 8.2 Hz), 137.4, 144.4 (d, J = 6.6 Hz), 145.2, 162.8 (d, J =248.8 Hz), 175.0. HRMS (ESI) m/z calcd for $C_{18}H_{17}FNaO_4S^+$ $[M+Na]^+$ 371.0724, found 371.0721.

(*R*)-5-(4-fluorophenyl)-5-(tosylmethyl)dihydrofuran-2(3*H*)-one

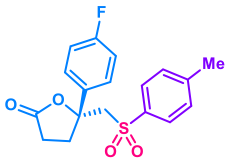

Compound 7j. (92% yield, 98% ee (*R*)). White solid, 64.1 mg at 0.20 mmol scale. The ee of **7j** was determined by HPLC analysis: (Chiralcel IA column, 1.0 mL/min, hexane/isopropanol = 80/20, 210 nm, 25 °C, t_{major} = 19.10 min (*R*), t_{minor} = 23.84 min (*S*)); $[\alpha]^{16}_D$ +11.3 (*c* 1.56, $CHCl_3$) for 98% ee (*R*). ^{1}H NMR (400 MHz, $CDCl_3$) δ 2.45 (s, 3H), 2.46 – 2.58 (m, 1H), 2.60 – 2.71 (m, 1H), 2.80 – 2.93 (m, 1H), 3.28 – 3.40 (m, 1H), 3.67 – 3.78 (m, 2H), 6.98 – 7.08 (m, 2H), 7.29 – 7.43 (m, 4H), 7.70 (d, J = 8.0 Hz, 2H). ^{19}F NMR (376 MHz, $CDCl_3$) δ -113.0. ^{13}C NMR (101 MHz, $CDCl_3$) δ 21.7, 28.2, 32.6, 65.2, 84.4, 115.8 (d, J = 21.8 Hz), 126.7 (d, J = 8.4 Hz), 127.9, 129.9, 137.38, 137.43 (d, J = 3.4 Hz), 145.1, 162.6 (d, J = 249.6 Hz), 175.0. HRMS (ESI) m/z calcd for $C_{18}H_{17}FNaO_4S^+$ $[M+Na]^+$ 371.0724, found 371.0722.

(*R*)-5-(4-iodophenyl)-5-(tosylmethyl)dihydrofuran-2(3*H*)-one

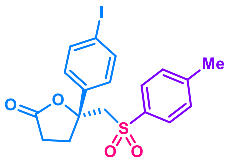

Compound 7k. (81% yield, 99% ee (*R*)). White solid, 73.9 mg at 0.20 mmol scale. The ee of **7k** was determined by HPLC analysis: (Chiralcel OD-H column, 1.0 mL/min, hexane/isopropanol = 80/20, 210 nm, 25 °C, t_{major} = 42.03 min (*R*), t_{minor} = 55.99 min (*S*)); $[\alpha]^{17}_D$ −23.1 (*c* 1.66, $CHCl_3$) for 99% ee (*R*). ^{1}H NMR (400 MHz, $CDCl_3$) δ 2.45 (s, 3H), 2.47 – 2.56 (m, 1H), 2.56 – 2.66 (m, 1H), 2.78 – 2.90 (m, 1H), 3.20 – 3.32 (m, 1H), 3.74 (s, 2H), 7.02 – 7.08 (m, 2H), 7.28 – 7.36 (m, 2H), 7.59 – 7.68 (m, 4H). ^{13}C NMR (101 MHz, $CDCl_3$) δ 21.7, 28.1, 32.6, 64.8, 84.3, 94.5, 126.7, 127.9, 129.9, 137.2, 137.9, 141.0, 145.1, 174.9. HRMS (ESI) m/z calcd for $C_{18}H_{17}INaO_4S^+$ $[M+Na]^+$ 478.9784, found 478.9785.

(*S*)-5-(thiophen-2-yl)-5-(tosylmethyl)dihydrofuran-2(3*H*)-one

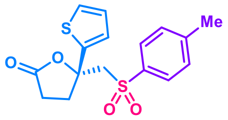

Compound 7l. (86% yield, 82% ee (*S*)). White solid, 57.9 mg at 0.20 mmol scale. The ee of **7l** was determined by HPLC analysis: (Chiralcel AD-H column, 1.0 mL/min, hexane/isopropanol = 80/20, 210 nm, 25 °C, t_{major} = 27.40 min (*S*), t_{minor} = 34.88 min (*R*)); $[\alpha]^{15}_D$ −1.7 (*c* 1.03, $CHCl_3$) for 82% ee (*S*). ^{1}H NMR (400 MHz, $CDCl_3$) δ 2.45 (s,

3H), 2.58 – 2.70 (m, 1H), 2.74 – 2.88 (m, 2H), 3.27 – 3.41 (m, 1H), 3.79 – 3.98 (m, 2H), 6.82 – 7.05 (m, 2H), 7.18 – 7.42 (m, 3H), 7.70 (d, *J* = 8.0 Hz, 2H). ^{13}C NMR (101 MHz, $CDCl_3$) δ 21.7, 28.6, 33.2, 65.3, 83.1, 125.0, 126.4, 127.2, 127.9, 129.9, 137.3, 144.7, 145.1, 174.8. HRMS (ESI) m/z calcd for $C_{16}H_{16}NaO_4S_2^+$ $[M+Na]^+$ 359.0382, found 359.0380.

(*R*)-3,3-dimethyl-5-phenyl-5-(tosylmethyl)dihydrofuran-2(3*H*)-one

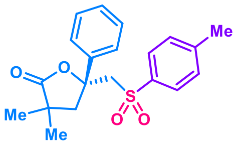

Compound 7m. (91% yield, 81% ee (*R*)). White solid, 65.2 mg at 0.20 mmol scale. The ee of **7m** was determined by HPLC analysis: (Chiralcel OD-H column, 1.0 mL/min, hexane/isopropanol = 80/20, 210 nm, 25 °C, t_{major} = 14.90 min (*R*), t_{minor} = 11.15 min (*S*)); $[\alpha]^{17}_{D}$ −17.6 (*c* 0.64, $CHCl_3$) for 81% ee (*R*). ^{1}H NMR (400 MHz, $CDCl_3$) δ 0.93 (s, 3H), 1.44 (s, 3H), 2.46 (s, 3H), 2.61 (d, *J* = 13.2 Hz, 1H), 3.42 (d, *J* = 13.2 Hz, 1H), 3.65 – 3.76 (m, 2H), 7.29 – 7.44 (m, 7H), 7.67 – 7.80 (m, 2H). ^{13}C NMR (101 MHz, $CDCl_3$) δ 21.7, 25.5, 26.3, 40.3, 46.3, 65.6, 124.5, 128.0, 128.3, 128.9, 129.8, 138.0, 143.0, 144.9, 180.6. HRMS (ESI) m/z calcd for $C_{20}H_{22}NaO_4S^+$ $[M+Na]^+$ 381.1131, found 381.1129.

(*R*)-5-(phenylethynyl)-5-(tosylmethyl)dihydrofuran-2(3*H*)-one

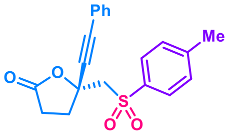

Compound 7n. (59% yield, 94% ee (*R*)). White solid, 41.8 mg at 0.20 mmol scale. The ee of **7n** was determined by HPLC analysis: (Chiralcel IA column, 1.0 mL/min, hexane/isopropanol = 80/20, 210 nm, 25 °C, t_{major} = 14.16 min (*R*), t_{minor} = 16.78 min (*S*)); $[\alpha]^{16}_{D}$ −18.4 (*c* 0.54, $CHCl_3$) for 94% ee (*R*). ^{1}H NMR (400 MHz, $CDCl_3$) δ 2.36 (s, 3H), 2.66 – 3.08 (m, 4H), 3.79 (d, *J* = 14.6 Hz, 1H), 3.92 (d, *J* = 14.6 Hz, 1H), 7.23 – 7.43 (m, 7H), 7.85 (d, *J* = 8.0 Hz, 2H). ^{13}C NMR (101 MHz, $CDCl_3$) δ 21.6, 28.4, 34.6, 63.3, 76.6, 85.1, 88.5, 120.8, 128.3, 128.4, 129.4, 129.9, 131.8, 136.8, 145.4, 174.6. HRMS (ESI) m/z calcd for $C_{20}H_{18}NaO_4S^+$ $[M+Na]^+$ 377.0818, found 377.0818.

(*R*)-5-benzoyl-5-(tosylmethyl)dihydrofuran-2(3*H*)-one

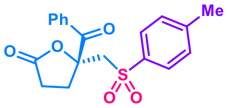

Compound 7o. (35% yield, 96% ee (*R*)). White solid, 25.1 mg at 0.20 mmol scale. The ee of **7o** was determined by HPLC analysis: (Chiralcel IA column, 1.0 mL/min, hexane/isopropanol = 80/20, 210 nm, 25 °C, t_{major} = 21.22 min (*R*), t_{minor} = 25.16 min (*S*)); $[\alpha]^{17}_{D}$ +42.2 (*c* 0.52, $CHCl_3$) for 96% ee (*R*). ^{1}H NMR (400 MHz, $CDCl_3$) δ 2.38 (s, 3H), 2.39 – 2.50 (m, 1H), 2.61 – 2.83 (m, 3H), 3.73 (d, *J* = 14.8 Hz, 1H), 4.02 (d, *J* = 14.8 Hz, 1H), 7.28 (d, *J* = 8.0 Hz, 2H), 7.35 (t, *J* = 8.0 Hz, 2H), 7.50 (t, *J* = 8.0 Hz, 1H), 7.69 (d, *J* = 8.0 Hz,

2H), 7.84 (d, J = 8.0 Hz, 2H). ^{13}C NMR (101 MHz, $CDCl_3$) δ 21.7, 27.4, 30.1, 62.1, 87.4, 128.1, 128.7, 130.0, 133.6, 133.6, 137.3, 145.4, 174.6, 196.7. HRMS (ESI) m/z calcd for $C_{19}H_{18}NaO_5S^+$ $[M+Na]^+$ 381.0767, found 381.0766.

(5*R*)-5-(1-([1,1'-biphenyl]-4-ylsulfonyl)ethyl)-5-phenyldihydrofuran-2(3*H*)-one

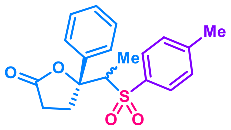

Compound 7p. (11% yield, 95% ee, 4:1 dr (5*R*)). White solid, 7.6 mg at 0.20 mmol scale. The ee of **7p** was determined by HPLC analysis: (Chiralcel OD-H column, 1.0 mL/min, hexane/isopropanol = 95/5, 210 nm, 25 °C, t_{major} = 44.90 min, 80.25 min, t_{minor} = 53.91 min, 59.24 min); $[\alpha]^{25}_D$ –47.2 (*c* 0.24, CH_2Cl_2) for 95% ee, 4:1 dr. ^{1}H NMR (400 MHz, $CDCl_3$) δ 0.96 (d, J = 7.2 Hz, 2.36H), 1.17 (d, J = 7.2 Hz, 0.66H), 2.21 – 2.47 (m, 4H), 2.47 – 2.68 (m, 1H), 2.73 – 2.96 (m, 1H), 3.10 – 3.80 (m, 2H), 7.20 – 7.48 (m, 7H), 7.61 – 7.76 (m, 2H). ^{13}C NMR (101 MHz, $CDCl_3$) δ 11.2, 13.3, 21.7, 28.4, 28.6, 30.8, 32.9, 67.4, 67.7, 87.7, 88.6, 124.8, 126.7, 128.3, 128.5, 128.9, 129.3, 129.8, 129.9, 135.6, 141.9, 142.9, 145.0, 145.1, 175.1, 176.0. HRMS (ESI) m/z calcd for $C_{19}H_{20}NaO_4S^+$ $[M+Na]^+$ 367.0975, found 367.0980.

(*R*)-5-cyclohexyl-5-(tosylmethyl)dihydrofuran-2(3*H*)-one

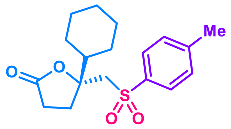

Compound 7q. (12% yield, 64% ee (*R*)). White solid, 8.1 mg at 0.20 mmol scale. The ee of **7q** was determined by HPLC analysis: (Chiralcel AD-H column, 1.0 mL/min, hexane/isopropanol = 80/20, 210 nm, 25 °C, t_{major} = 13.54 min (*R*), t_{minor} = 15.28 min (*S*)); $[\alpha]^{25}_D$ –9.1 (*c* 0.44, $CHCl_3$) for 64% ee (*R*). ^{1}H NMR (400 MHz, $CDCl_3$) δ 0.79 – 0.97 (m, 2H), 0.99 – 1.18 (m, 3H), 1.52 (dt, J = 12.0, 3.0 Hz, 1H), 1.56 – 1.66 (m, 3H), 1.67 – 1.76 (m, 2H), 2.10 – 2.26 (m, 1H), 2.38 (s, 3H), 2.39 – 2.57 (m, 1H), 2.67 – 2.93 (m, 2H), 3.37 (d, J = 14.9 Hz, 1H), 3.48 (d, J = 14.9 Hz, 1H), 7.29 (d, J = 8.0 Hz, 2H), 7.62 – 7.77 (m, 2H). ^{13}C NMR (101 MHz, $CDCl_3$) δ 21.8, 25.9, 25.9, 26.1, 26.2, 26.5, 27.3, 29.1, 48.3, 61.9, 86.8, 127.9, 130.0, 138.2, 145.1, 176.4. HRMS (ESI) m/z calcd for $C_{18}H_{25}O_4S^+$ $[M+H]^+$ 337.1468, found 337.1474.

(*R*)-5-(([1,1'-biphenyl]-4-ylsulfonyl)methyl)-5-phenyldihydrofuran-2(3*H*)-one

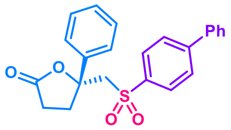

Compound 7r. (92% yield, 99% ee (*R*)). White solid, 72.2 mg at 0.20 mmol scale. The ee of **7r** was determined by HPLC analysis: (Chiralcel IA column, 1.0 mL/min, hexane/isopropanol = 80/20, 210 nm, 25 °C, t_{major} = 26.11 min (*R*), t_{minor} = 23.51 min (*S*)); $[\alpha]^{18}_D$ +24.5 (*c* 1.83, $CHCl_3$) for 99% ee (*R*). ^{1}H NMR (400 MHz, $CDCl_3$) δ 2.47 – 2.59 (m, 1H), 2.64 – 2.74 (m, 1H), 2.83 – 2.94 (m, 1H), 3.32 – 3.44 (m, 1H), 3.78 –

3.89 (m, 2H), 7.29 – 7.40 (m, 5H), 7.41 – 7.54 (m, 3H), 7.58 – 7.65 (m, 2H), 7.68 – 7.75 (m, 2H), 7.84 – 7.93 (m, 2H). ^{13}C NMR (101 MHz, $CDCl_3$) δ 28.3, 32.7, 65.2, 84.8, 124.7, 127.5, 128.0, 128.6, 128.7, 128.8, 129.0, 129.2, 138.9, 139.2, 141.8, 146.9, 175.5. HRMS (ESI) m/z calcd for $C_{23}H_{20}NaO_4S^+$ $[M+Na]^+$ 415.0975, found 415.0974.

(*R*)-5-((naphthalen-2-ylsulfonyl)methyl)-5-phenyldihydrofuran-2(3*H*)-one

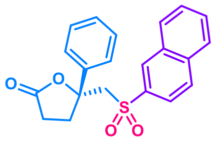

Compound 7s. (96% yield, 99% ee (*R*)). White solid, 70.4 mg at 0.20 mmol scale. The ee of **7s** was determined by HPLC analysis: (Chiralcel IA column, 1.0 mL/min, hexane/isopropanol = 80/20, 210 nm, 25 °C, t_{major} = 20.93 min (*R*), t_{minor} = 25.39 min (*S*)); $[\alpha]^{18}_{D}$ +10.2 (*c* 1.82, $CHCl_3$) for 99% ee (*R*). ^{1}H NMR (400 MHz, $CDCl_3$) δ 2.46 – 2.60 (m, 1H), 2.64 – 2.76 (m, 1H), 2.82 – 2.96 (m, 1H), 3.32 – 3.46 (m, 1H), 3.78 – 3.92 (m, 2H), 7.21 – 7.39 (m, 5H), 7.57 – 7.74 (m, 2H), 7.76 – 7.85 (m, 1H), 7.87 – 8.04 (m, 3H), 8.32 – 8.42 (m, 1H). ^{13}C NMR (101 MHz, $CDCl_3$) δ 28.4, 32.7, 65.1, 84.8, 122.4, 124.7, 127.8, 128.1, 128.7, 128.9, 129.6, 129.66, 129.70, 130.1, 132.1, 135.4, 137.2, 141.6, 175.5. HRMS (ESI) m/z calcd for $C_{21}H_{18}NaO_4S^+$ $[M+Na]^+$ 389.0818, found 389.0816.

(*R*)-5-(((4-ethynylphenyl)sulfonyl)methyl)-5-phenyldihydrofuran-2(3*H*)-one

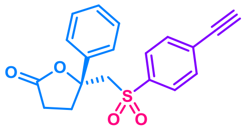

Compound 7t. (67% yield, >99.5% ee (*R*)). White solid, 45.6 mg at 0.20 mmol scale. The ee of **7t** was determined by HPLC analysis: (Chiralcel IA column, 1.0 mL/min, hexane/isopropanol = 90/10, 210 nm, 25 °C, t_{major} = 37.33 min (*R*), t_{minor} = 44.93 min (*S*)); $[\alpha]^{16}_{D}$ +10.6 (*c* 1.21, $CHCl_3$) for >99.5% ee (*R*). ^{1}H NMR (400 MHz, $CDCl_3$) δ 2.34 – 2.47 (m, 1H), 2.52 – 2.62 (m, 1H), 2.68 – 2.80 (m, 1H), 3.10 – 3.32 (m, 2H), 3.69 (s, 2H), 7.15 – 7.30 (m, 5H), 7.51 (d, *J* = 8.2 Hz, 2H), 7.68 (d, *J* = 8.0 Hz, 2H). ^{13}C NMR (101 MHz, $CDCl_3$) δ 28.1, 32.7, 65.1, 81.8, 84.5, 124.5, 127.9, 128.1, 128.7, 129.0, 132.8, 140.2, 141.5, 175.1. HRMS (ESI) m/z calcd for $C_{19}H_{16}NaO_4S^+$ $[M+Na]^+$ 363.0662, found 363.0659.

(*R*)-5-phenyl-5-((*o*-tolylsulfonyl)methyl)dihydrofuran-2(3*H*)-one

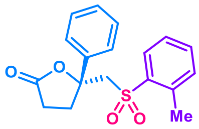

Compound 7u. (84% yield, 99% ee (*R*)). White solid, 55.5 mg at 0.20 mmol scale. The ee of **7u** was determined by HPLC analysis: (Chiralcel OD-H column, 1.0 mL/min, hexane/isopropanol = 90/10, 210 nm, 25 °C, t_{major} = 41.74 min (*R*), t_{minor} = 56.26 min (*S*)); $[\alpha]^{17}_{D}$ −3.8 (*c* 1.45, $CHCl_3$) for 99% ee (*R*). ^{1}H NMR (400 MHz, $CDCl_3$) 2.33 – 2.46 (m, 1H), 2.56 (s, 3H),

2.56 – 2.63 (m, 1H), 2.69 – 2.80 (m, 1H), 3.22 –3.34 (m, 1H), 3.66 (d, *J* = 15.0 Hz, 1H), 3.72 (d, *J* = 15.0 Hz, 1H), 7.16 – 7.30 (m, 7H), 7.40 (td, *J* = 7.6, 1.4 Hz, 1H), 7.79 (d, *J* = 8.0 Hz, 1H). ^{13}C NMR (101 MHz, $CDCl_3$) δ 20.4, 28.3, 32.3, 64.2, 84.8, 124.6, 126.7, 128.6, 128.9, 129.9, 132.8, 134.0, 137.7, 138.3, 141.8, 175.2. HRMS (ESI) m/z calcd for $C_{18}H_{18}NaO_4S^+$ $[M+Na]^+$ 353.0818, found 353.0814.

(*R*)-5-(((4-(*tert*-butyl)phenyl)sulfonyl)methyl)-5-phenyldihydrofuran-2(3*H*)-one

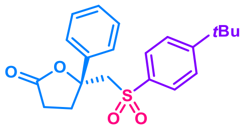

Compound 7v. (97% yield, >99.5% ee (*R*)). White solid, 72.3 mg at 0.20 mmol scale. The ee of **7v** was determined by HPLC analysis: (Chiralcel IA column, 1.0 mL/min, hexane/isopropanol = 90/10, 210 nm, 25 °C, t_{major} = 21.28 min (*R*), t_{minor} = 25.27 min (*S*)); $[\alpha]^{17}_D$ +11.6 (*c* 3.36, $CHCl_3$) for >99.5% ee (*R*). ^{1}H NMR (400 MHz, Chloroform-*d*) δ 1.34 (s, 9H), 2.45 – 2.58 (m, 1H), 2.62 – 2.73 (m, 1H), 2.82 – 2.95 (m, 1H), 3.30 – 3.42 (m, 1H), 3.77 (s, 2H), 7.26 – 7.37 (m, 5H), 7.51 (d, *J* = 8.6 Hz, 2H), 7.74 (d, *J* = 8.6 Hz, 2H). ^{13}C NMR (101 MHz, $CDCl_3$) 28.3, 31.0, 32.6, 35.3, 65.1, 84.8, 124.7, 126.3, 127.7, 128.5, 128.8, 137.3, 141.7, 157.8, 175.4. HRMS (ESI) m/z calcd for $C_{21}H_{24}NaO_4S^+$ $[M+Na]^+$ 395.1288, found 395.1287.

(*R*)-5-(((4-methoxyphenyl)sulfonyl)methyl)-5-phenyldihydrofuran-2(3*H*)-one

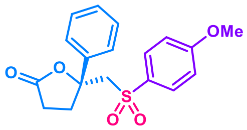

Compound 7w. (92% yield, 99% ee (*R*)). White solid, 63.7 mg at 0.20 mmol scale. The ee of **7w** was determined by HPLC analysis: (Chiralcel IA column, 1.0 mL/min, hexane/isopropanol = 80/20, 210 nm, 25 °C, t_{major} = 21.61 min (*R*), t_{minor} = 24.28 min (*S*)); $[\alpha]^{18}_D$ +5.4 (*c* 1.56, $CHCl_3$) for 99% ee (*R*). ^{1}H NMR (400 MHz, $CDCl_3$) δ 2.34 – 2.48 (m, 1H), 2.50 – 2.61 (m, 1H), 2.71 – 2.83 (m, 1H), 3.22 – 3.35 (m, 1H), 3.57 – 3.75 (m, 2H), 3.79 (s, 3H), 6.82 – 6.92 (m, 2H), 7.15 – 7.32 (m, 5H), 7.55 – 7.80 (m, 2H). ^{13}C NMR (101 MHz, $CDCl_3$) δ 28.3, 32.5, 55.7, 65.3, 84.8, 114.4, 124.5, 128.5, 128.9, 130.2, 132.0, 142.0, 163.9, 175.4. HRMS (ESI) m/z calcd for $C_{18}H_{18}NaO_5S^+$ $[M+Na]^+$ 369.0767, found 369.0762.

(*R*)-5-(((4-phenoxyphenyl)sulfonyl)methyl)-5-phenyldihydrofuran-2(3*H*)-one

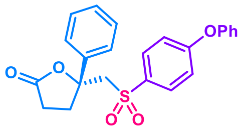

Compound 7x. (87% yield, >99.5% ee (*R*)). White solid, 71.1 mg at 0.20 mmol scale. The ee of **7x** was determined by HPLC analysis: (Chiralcel OD-H column, 1.0 mL/min, hexane/isopropanol = 60/40, 210 nm, 25 °C, t_{major} = 21.16 min (*R*), t_{minor} = 25.97 min (*S*)); $[\alpha]^{18}_D$ +28.3 (*c* 1.78, $CHCl_3$) for >99.5% ee (*R*). ^{1}H NMR (400 MHz, $CDCl_3$) δ 2.33 – 2.47 (m, 1H), 2.48 – 2.61 (m, 1H), 2.67 – 2.80 (m, 1H),

3.17 – 3.31 (m, 1H), 3.60 – 3.75 (m, 2H), 6.83 – 6.95 (m, 2H), 6.95 – 7.02 (m, 2H), 7.15 (t, J = 7.6 Hz, 1H), 7.19 – 7.29 (m, 5H), 7.29 – 7.37 (m, 2H), 7.60 – 7.71 (m, 2H). ^{13}C NMR (101 MHz, $CDCl_3$) δ 28.3, 32.7, 65.2, 84.8, 117.6, 120.6, 124.7, 125.3, 128.7, 129.0, 130.36, 130.39, 133.8, 141.8, 154.9, 162.7, 175.5. HRMS (ESI) m/z calcd for $C_{23}H_{20}NaO_5S^+$ $[M+Na]^+$ 431.0924, found 431.0926.

(*R*)-5-(((3-fluorophenyl)sulfonyl)methyl)-5-phenyldihydrofuran-2(3*H*)-one

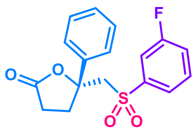

Compound 7y. (96% yield, 99% ee (*R*)). White solid, 64.2 mg at 0.20 mmol scale. The ee of **7y** was determined by HPLC analysis: (Chiralcel IA column, 1.0 mL/min, hexane/isopropanol = 80/20, 210 nm, 25 °C, t_{major} = 13.71 min (*R*), t_{minor} = 16.46 min (*S*)); $[\alpha]^{18}_D$ +26.6 (*c* 1.61, $CHCl_3$) for 99% ee (*R*). ^{1}H NMR (400 MHz, $CDCl_3$) δ 2.42 – 2.57 (m, 1H), 2.62 – 2.72 (m, 1H), 2.77 – 2.89 (m, 1H), 3.21 – 3.34 (m, 1H), 3.76 – 3.89 (m, 2H), 7.23 – 7.40 (m, 6H), 7.44 – 7.56 (m, 2H), 7.59 – 7.66 (m, 1H). ^{19}F NMR (376 MHz, $CDCl_3$) δ -109.0. ^{13}C NMR (101 MHz, $CDCl_3$) δ 28.2, 32.9, 65.0, 84.6, 115.4 (d, J = 24.7 Hz), 121.3 (d, J = 21.3 Hz), 123.9 (d, J = 3.4 Hz), 124.7, 128.8, 129.0, 131.2 (d, J = 7.7 Hz), 141.3, 142.3 (d, J = 6.5 Hz), 162.3 (d, J = 253.7 Hz), 175.3. HRMS (ESI) m/z calcd for $C_{17}H_{15}FNaO_4S^+$ $[M+Na]^+$ 357.0567, found 357.0568.

(*R*)-5-(((4-bromophenyl)sulfonyl)methyl)-5-phenyldihydrofuran-2(3*H*)-one

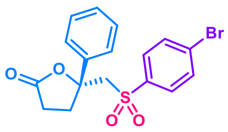

Compound 7z. (84% yield, 99% ee (*R*)). White solid, 66.4 mg at 0.20 mmol scale. The ee of **7z** was determined by HPLC analysis: (Chiralcel OD-H column, 1.0 mL/min, hexane/isopropanol = 80/20, 210 nm, 25 °C, t_{major} = 43.40 min (*R*), t_{minor} = 40.50 min (*S*)); $[\alpha]^{17}_D$ −4.8 (*c* 2.32, $CHCl_3$) for 99% ee (*R*). ^{1}H NMR (400 MHz, $CDCl_3$) ^{1}H NMR (400 MHz, $CDCl_3$) δ 2.35 – 2.46 (m, 1H), 2.51 – 2.61 (m, 1H), 2.68 – 2.79 (m, 1H), 3.14 – 3.27 (m, 1H), 3.71 (s, 2H), 7.12 – 7.32 (m, 5H), 7.38 – 7.73 (m, 4H). ^{13}C NMR (101 MHz, $CDCl_3$) δ 28.1, 32.9, 65.1, 84.5, 124.5, 128.6, 129.0, 129.3, 129.5, 132.5, 139.4, 141.4, 175.2. HRMS (ESI) m/z calcd for $C_{17}H_{15}{}^{79}BrNaO_4S^+$ $[M+Na]^+$ 416.9767, found 416.9769.

(*R*)-5-(((3-iodophenyl)sulfonyl)methyl)-5-phenyldihydrofuran-2(3*H*)-one

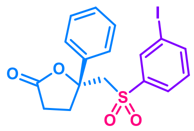

Compound 7aa. (60% yield, 99% ee (*R*)). White solid, 53.1 mg at 0.20 mmol scale. The ee of **7aa** was determined by HPLC analysis: (Chiralcel OD-H column, 1.0 mL/min, hexane/isopropanol = 80/20, 210 nm, 25 °C, t_{major} = 39.50 min (*R*), t_{minor} = 36.99 min (*S*)); $[\alpha]^{17}_D$ +15.9 (*c* 0.72, $CHCl_3$) for 99% ee (*R*). ^{1}H NMR (400 MHz, $CDCl_3$) δ 2.46 – 2.58 (m,

1H), 2.64 – 2.74 (m, 1H), 2.78 – 2.89 (m, 1H), 3.23 – 3.33 (m, 1H), 3.80 (s, 2H), 7.25 – 7.42 (m, 6H), 7.79 (d, *J* = 8.0 Hz, 1H), 7.94 (d, *J* = 8.0 Hz, 1H), 8.05 (t, *J* = 1.8 Hz, 1H). ^{13}C NMR (101 MHz, $CDCl_3$) δ 28.1, 32.8, 65.1, 84.5, 94.3, 124.7, 127.1, 128.8, 128.9, 130.8, 136.5, 141.0, 141.9, 142.8, 175.0. HRMS (ESI) m/z calcd for $C_{17}H_{15}INaO_4S^+$ $[M+Na]^+$ 464.9628, found 464.9629.

(*R*)-5-(((3-(methylthio)phenyl)sulfonyl)methyl)-5-phenyldihydrofuran-2(3*H*)-one

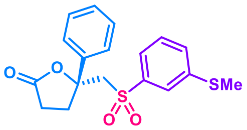

Compound 7ab. (83% yield, >99.5% ee (*R*)). White solid, 60.2 mg at 0.20 mmol scale. The ee of **7ab** was determined by HPLC analysis: (Chiralcel IA column, 1.0 mL/min, hexane/isopropanol = 80/20, 210 nm, 25 °C, t_{major} = 15.62 min (*R*), t_{minor} = 18.55 min (*S*)); $[\alpha]^{18}_{D}$ +29.8 (*c* 1.46, $CHCl_3$) for >99.5% ee (*R*). ^{1}H NMR (400 MHz, $CDCl_3$) δ 2.44 – 2.58 (m, 4H), 2.60 – 2.70 (m, 1H), 2.79 – 2.91 (m, 1H), 3.26 – 3.39 (m, 1H), 3.73 – 3.86 (m, 2H), 7.23 – 7.38 (m, 5H), 7.39 – 7.48 (m, 2H), 7.52 – 7.57 (m, 1H), 7.58 – 7.63 (m, 1H). ^{13}C NMR (101 MHz, $CDCl_3$) δ 15.3, 28.3, 32.8, 65.1, 84.7, 124.0, 124.5, 124.6, 128.7, 129.0, 129.5, 131.3, 141.1, 141.5, 141.6, 175.4. HRMS (ESI) m/z calcd for $C_{18}H_{18}NaO_4S_2^+$ $[M+Na]^+$ 385.0539, found 385.0536.

(*R*)-5-(((3-methoxyphenyl)sulfonyl)methyl)-5-phenyldihydrofuran-2(3*H*)-one

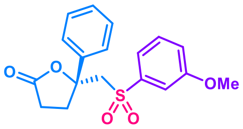

Compound 7ac. (93% yield, 99% ee (*R*)). White solid, 64.4 mg at 0.20 mmol scale. The ee of **7ac** was determined by HPLC analysis: (Chiralcel IA column, 1.0 mL/min, hexane/isopropanol = 90/10, 210 nm, 25 °C, t_{major} = 30.08 min (*R*), t_{minor} = 36.18 min (*S*)); $[\alpha]^{17}_{D}$ −6.2 (*c* 1.64, $CHCl_3$) for 99% ee (*R*). ^{1}H NMR (400 MHz, $CDCl_3$) δ 2.36 – 2.50 (m, 1H), 2.51 – 2.64 (m, 1H), 2.72 – 2.86 (m, 1H), 3.23 – 3.36 (m, 1H), 3.58 – 3.74 (m, 2H), 3.79 (s, 3H), 7.00 – 7.11 (m, 1H), 7.14 – 7.40 (m, 8H). ^{13}C NMR (101 MHz, $CDCl_3$) 28.2, 32.6, 55.8, 65.1, 84.6, 112.2, 120.0, 120.8, 124.5, 128.6, 128.9, 130.3, 141.6, 141.9, 160.0, 175.3. HRMS (ESI) m/z calcd for $C_{18}H_{18}NaO_5S^+$ $[M+Na]^+$ 369.0767, found 369.0766.

(*R*)-5-(((4-benzoylphenyl)sulfonyl)methyl)-5-phenyldihydrofuran-2(3*H*)-one

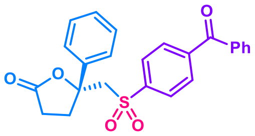

Compound 7ad. (91% yield, 99% ee (*R*)). White solid, 76.5 mg at 0.20 mmol scale. The ee of **7ad** was determined by HPLC analysis: (Chiralcel IA column, 1.0 mL/min, hexane/isopropanol = 80/20, 210 nm, 25 °C, t_{major} = 25.21 min (*R*), t_{minor} = 27.91 min (*S*)); $[\alpha]^{16}_{D}$ +2.4 (*c* 1.64, $CHCl_3$) for 99% ee (*R*). ^{1}H NMR (400 MHz, $CDCl_3$) δ 2.45 – 2.57 (m, 1H), 2.62 – 2.73 (m, 1H), 2.77 – 2.89 (m, 1H), 3.26 – 3.39 (m, 1H), 3.82 –

3.94 (m, 2H), 7.29 – 7.41 (m, 5H), 7.53 (t, *J* = 7.6 Hz, 2H), 7.66 (t, *J* = 7.6 Hz, 1H), 7.76 – 7.84 (m, 2H), 7.85 – 7.99 (m, 4H). ^{13}C NMR (101 MHz, $CDCl_3$) δ 28.1, 32.9, 65.0, 84.5, 124.6, 128.0, 128.6, 128.7, 129.0, 130.2, 130.4, 133.4, 136.4, 141.4, 142.3, 143.2, 175.1, 195.1. HRMS (ESI) m/z calcd for $C_{24}H_{20}NaO_5S^+$ $[M+Na]^+$ 443.0924, found 443.0924.

(*R*)-5-(((4-acetylphenyl)sulfonyl)methyl)-5-phenyldihydrofuran-2(3*H*)-one

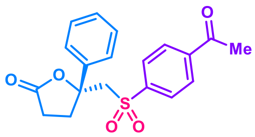

Compound 7ae. (91% yield, >99.5% ee (*R*)). White solid, 65.2 mg at 0.20 mmol scale. The ee of **7ae** was determined by HPLC analysis: (Chiralcel IA column, 1.0 mL/min, hexane/isopropanol = 80/20, 210 nm, 25 °C, t_{major} = 29.00 min (*R*), t_{minor} = 33.49 min (*S*)); $[\alpha]^{16}_D$ +11.7 (*c* 1.50, $CHCl_3$) for >99.5% ee (*R*). ^{1}H NMR (400 MHz, DMSO-d_6) δ 2.29 – 2.46 (m, 2H), 2.64 (s, 3H), 2.68 – 2.81 (m, 1H), 2.82 – 2.94 (m, 1H), 4.44 (s, 2H), 7.16 – 7.37 (m, 5H), 7.79 (d, *J* = 8.2 Hz, 2H), 8.01 (d, *J* = 8.2 Hz, 2H). ^{13}C NMR (101 MHz, DMSO-d_6) δ 27.6, 27.7, 35.0, 63.2, 84.8, 125.3, 128.3, 128.4, 128.7, 129.0, 140.5, 141.5, 144.4, 176.0, 198.0. HRMS (ESI) m/z calcd for $C_{19}H_{18}NaO_5S^+$ $[M+Na]^+$ 381.0767, found 381.0766.

methyl (*R*)-4-(((5-oxo-2-phenyltetrahydrofuran-2-yl)methyl)sulfonyl)benzoate

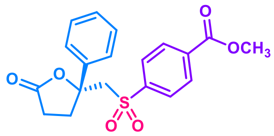

Compound 7af. (71% yield, 99% ee (*R*)). White solid, 53.2 mg at 0.20 mmol scale. The ee of **7af** was determined by HPLC analysis: (Chiralcel AD-H column, 1.0 mL/min, hexane/isopropanol = 80/20, 210 nm, 25 °C, t_{major} = 31.73 min (*R*), t_{minor} = 27.44 min (*S*)); $[\alpha]^{16}_D$ +5.2 (*c* 1.47, $CHCl_3$) for 99% ee (*R*). ^{1}H NMR (400 MHz, $CDCl_3$) δ 2.43 – 2.57 (m, 1H), 2.61 – 2.72 (m, 1H), 2.76 – 2.90 (m, 1H), 3.26 – 3.38 (m, 1H), 3.83 (s, 2H), 3.98 (s, 3H), 7.21 – 7.42 (m, 5H), 7.88 (d, *J* = 8.2 Hz, 2H), 8.16 (d, *J* = 8.0 Hz, 2H). ^{13}C NMR (101 MHz, $CDCl_3$) δ 28.0, 32.8, 52.7, 64.9, 84.4, 124.5, 128.1, 128.7, 129.0, 130.4, 134.9, 141.3, 144.1, 165.4, 175.1. HRMS (ESI) m/z calcd for $C_{19}H_{18}NaO_6S^+$ $[M+Na]^+$ 397.0716, found 397.0717.

(*R*)-5-phenyl-5-(((4-(trifluoromethyl)phenyl)sulfonyl)methyl)dihydrofuran-2(3*H*)-one

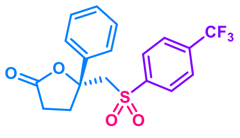

Compound 7ag. (81% yield, 98% ee (*R*)). White solid, 62.3 mg at 0.20 mmol scale. The ee of **7ag** was determined by HPLC analysis: (Chiralcel IA column, 1.0 mL/min, hexane/isopropanol = 80/20, 210 nm, 25 °C, t_{major} = 12.00 min (*R*), t_{minor} = 19.90 min (*S*)); $[\alpha]^{18}_D$ +11.3 (*c* 1.30, $CHCl_3$) for 98% ee (*R*). ^{1}H NMR (400 MHz, $CDCl_3$) δ 2.45

– 2.57 (m, 1H), 2.62 – 2.72 (m, 1H), 2.77 – 2.89 (m, 1H), 3.21 – 3.34 (m, 1H), 3.87 (s, 2H), 7.24 – 7.36 (m, 5H), 7.77 (d, J = 8.0 Hz, 2H), 7.94 (d, J = 8.0 Hz, 2H). ^{19}F NMR (376 MHz, $CDCl_3$) δ -63.3. ^{13}C NMR (101 MHz, $CDCl_3$) δ 28.1, 33.2, 65.0, 84.4, 123.1 (q, J = 274.4 Hz), 124.6, 126.4 (q, J = 3.8 Hz), 128.76, 128.83, 129.1, 135.4 (q, J = 33.3 Hz), 141.1, 143.7, 175.3. HRMS (ESI) m/z calcd for $C_{18}H_{15}F_3NaO_4S^+$ $[M+Na]^+$ 407.0535, found 407.0534.

(*R*)-5-(((4-nitrophenyl)sulfonyl)methyl)-5-phenyldihydrofuran-2(3*H*)-one

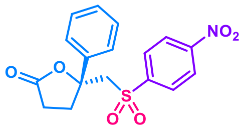

Compound 7ah. (75% yield, >99.5% ee (*R*)). Yellow solid, 54.2 mg at 0.20 mmol scale. The ee of **7ah** was determined by HPLC analysis: (Chiralcel OD-H column, 1.0 mL/min, hexane/isopropanol = 90/10, 210 nm, 25 °C, t_{major} = 36.78 min (*R*), t_{minor} = 32.16 min (*S*)); $[\alpha]^{16}_D$ −3.1 (*c* 1.15, $CHCl_3$) for >99.5% ee (*R*). ^{1}H NMR (400 MHz, DMSO-d_6) δ 2.30 – 2.45 (m, 2H), 2.67 – 2.92 (m, 2H), 4.48 – 4.63 (m, 2H), 7.10 – 7.39 (m, 5H), 7.74 – 7.98 (m, 2H), 8.16 – 8.41 (m, 2H). ^{13}C NMR (101 MHz, DMSO-d_6) δ 27.7, 35.2, 63.1, 84.7, 124.5, 125.3, 128.3, 128.7, 129.8, 141.2, 146.1, 150.4, 176.0. HRMS (ESI) m/z calcd for $C_{17}H_{15}NNaO_6S^+$ $[M+Na]^+$ 384.0512, found 384.0511.

ethyl (*R*)-5-(((5-oxo-2-phenyltetrahydrofuran-2-yl)methyl)sulfonyl)benzofuran-2-carboxylate

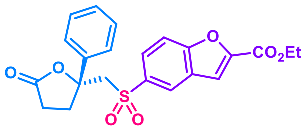

Compound 7ai. (75% yield, 99% ee (*R*)). White solid, 64.3 mg at 0.20 mmol scale. The ee of **7ai** was determined by HPLC analysis: (Chiralcel AD-H column, 1.0 mL/min, hexane/isopropanol = 80/20, 210 nm, 25 °C, t_{major} = 20.95 min (*R*), t_{minor} = 15.85 min (*S*)); $[\alpha]^{15}_D$ −13.5 (*c* 1.11, $CHCl_3$) for 99% ee (*R*). ^{1}H NMR (400 MHz, $CDCl_3$) δ 1.45 (t, J = 7.2 Hz, 3H), 2.43 – 2.57 (m, 1H), 2.60 – 2.73 (m, 1H), 2.77 – 2.92 (m, 1H), 3.26 – 3.40 (m, 1H), 3.78 – 3.93 (m, 2H), 4.48 (q, J = 7.2 Hz, 2H), 7.19 – 7.39 (m, 5H), 7.59 (s, 1H), 7.68 (d, J = 8.8 Hz, 1H), 7.88 (dd, J = 8.0, 1.6 Hz, 1H), 8.19 (d, J = 2.0 Hz, 1H). ^{13}C NMR (101 MHz, $CDCl_3$) δ 14.4, 28.2, 32.9, 62.2, 65.4, 84.7, 113.5, 113.7, 124.63, 124.65, 126.8, 127.4, 128.7, 129.0, 136.4, 141.5, 148.2, 157.7, 158.9, 175.4. HRMS (ESI) m/z calcd for $C_{22}H_{20}NaO_7S^+$ $[M+Na]^+$ 451.0822, found 451.0822.

(1*S*,2*R*,4*R*)-2-isopropyl-4-methylcyclohexyl 4-((((*R*)-5-oxo-2-phenyltetrahydrofuran-2-yl)methyl)sulfonyl)benzoate

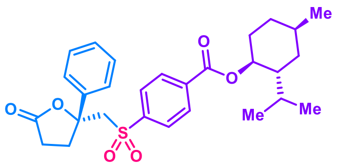

Compound 7aj. (63% yield, 77% de). White solid, 62.8 mg at 0.20 mmol scale. The de of **7aj** was determined by HPLC analysis: (Chiralcel AD-H column, 1.0 mL/min, hexane/isopropanol = 80/20, 210 nm, 25 °C, t_{major} = 14.37 min, t_{minor} = 35.18 min); $[\alpha]^{16}_{D}$ −27.1 (*c* 1.33, $CHCl_3$) for 77% de. ^1H NMR (400 MHz, $CDCl_3$) δ 0.82 (d, *J* = 7.0 Hz, 3H), 0.95 (t, *J* = 6.4 Hz, 7H), 1.06 – 1.19 (m, 2H), 1.50 – 1.65 (m, 2H), 1.71 – 1.82 (m, 2H), 1.86 – 1.99 (m, 1H), 2.06 – 2.20 (m, 1H), 2.45 – 2.60 (m, 1H), 2.62 – 2.73 (m, 1H), 2.79 – 2.92 (m, 1H), 3.26 – 3.42 (m, 1H), 3.82 (s, 2H), 4.98 (td, *J* = 10.8, 4.4 Hz, 1H), 7.23 – 7.39 (m, 5H), 7.89 (d, *J* = 8.2 Hz, 2H), 8.17 (d, *J* = 8.4 Hz, 2H). ^{13}C NMR (101 MHz, $CDCl_3$) δ 16.5, 20.7, 22.0, 23.6, 26.6, 28.1, 31.5, 32.8, 34.2, 40.8, 47.2, 65.0, 84.4, 124.5, 128.0, 128.7, 129.0, 130.3, 135.6, 141.5, 143.9, 164.4, 175.1. HRMS (ESI) m/z calcd for $C_{28}H_{34}NaO_6S^+$ $[M+Na]^+$ 521.1968, found 521.1970.

(*R*)-6-phenyl-6-(tosylmethyl)tetrahydro-2*H*-pyran-2-one

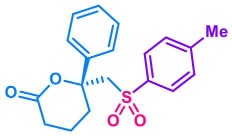

Compound 9a. (88% yield, 92% ee (*R*)). White solid, 60.6 mg at 0.20 mmol scale. The ee of **9a** was determined by HPLC analysis: (Chiralcel IA column, 1.0 mL/min, hexane/isopropanol = 80/20, 210 nm, 25 °C, t_{major} = 19.21 min (*R*), t_{minor} = 16.15 min (*S*)); $[\alpha]^{16}_{D}$ −33.8 (*c* 0.80, $CHCl_3$) for 92% ee (*R*). ^1H NMR (400 MHz, $CDCl_3$) δ 1.36 – 1.50 (m, 1H), 1.72 – 1.80 (m, 1H), 2.28 – 2.52 (m, 6H), 2.79 – 2.93 (m, 1H), 3.54 – 3.66 (m, 2H), 7.16 – 7.30 (m, 7H), 7.65 (d, *J* = 8.0 Hz, 2H). ^{13}C NMR (101 MHz, $CDCl_3$) δ 16.2, 21.7, 29.3, 30.3, 66.5, 84.5, 125.0, 128.0, 128.3, 129.0, 129.8, 137.8, 141.5, 144.8, 169.7. HRMS (ESI) m/z calcd for $C_{19}H_{20}NaO_4S^+$ $[M+Na]^+$ 367.0975, found 367.0973.

(*R*)-6-(((3-chlorophenyl)sulfonyl)methyl)-6-(m-tolyl)tetrahydro-2*H*-pyran-2-one

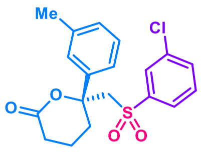

Compound 9b. (76% yield, 90% ee (*R*)). White solid, 57.6 mg at 0.20 mmol scale. The ee of **9b** was determined by HPLC analysis: (Chiralcel IA column, 1.0 mL/min, hexane/isopropanol = 90/10, 210 nm, 25 °C, t_{major} = 19.71 min (*R*), t_{minor} = 22.58 min (*S*)); $[\alpha]^{15}_{D}$ −25.4 (*c* 1.29, $CHCl_3$) for 90% ee (*R*). ^1H NMR (400 MHz, $CDCl_3$) δ 1.47 – 1.64 (m, 1H), 1.78 – 1.91 (m, 1H), 2.32 (s, 3H), 2.39 – 2.63 (m, 3H), 2.78 – 2.91 (m, 1H), 3.75 (s, 2H), 7.00 – 7.16 (m, 3H), 7.23 (t, *J* = 8.0 Hz, 1H), 7.47 (t, *J* = 8.0 Hz, 1H), 7.53 – 7.61 (m, 1H), 7.67 – 7.83 (m, 2H). ^{13}C NMR (101 MHz, $CDCl_3$) δ 16.2, 21.6, 29.3, 30.6, 66.5, 84.3, 122.1, 125.7, 126.3, 128.2, 129.0, 129.4, 130.5, 134.0, 135.4, 139.0,

140.7, 142.2, 169.7 HRMS (ESI) m/z calcd for $C_{19}H_{19}ClNaO_4S^+$ $[M+Na]^+$ 401.0585, found 401.0583.

(*R*)-6-(((3-chlorophenyl)sulfonyl)methyl)-6-(3-methoxyphenyl)tetrahydro-2*H*-pyran-2-one

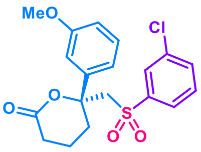

Compound 9c. (80% yield, 88% ee (*R*)). Colorless oil, 63.2 mg at 0.20 mmol scale. The ee of **9c** was determined by HPLC analysis: (Chiralcel IA column, 1.0 mL/min, hexane/isopropanol = 80/20, 210 nm, 25 °C, t_{major} = 14.63 min (*R*), t_{minor} = 16.82 min (*S*)); $[\alpha]^{15}_{D}$ −18.5 (*c* 1.48, $CHCl_3$) for 88% ee (*R*). ^1H NMR (400 MHz, $CDCl_3$) δ 1.47 – 1.60 (m, 1H), 1.76 – 1.86 (m, 1H), 2.35 – 2.61 (m, 3H), 2.74 – 2.86 (m, 1H), 3.70 (s, 2H), 3.74 (s, 3H), 6.71 – 6.87 (m, 3H), 7.24 (d, *J* = 8.0 Hz, 1H), 7.44 (t, *J* = 8.0 Hz, 1H), 7.51 – 7.57 (m, 1H), 7.66 – 7.77 (m, 2H). ^{13}C NMR (101 MHz, $CDCl_3$) δ 16.2, 29.2, 30.6, 55.3, 66.3, 84.1, 111.4, 113.5, 117.2, 126.2, 128.1, 130.2, 130.5, 133.9, 135.3, 142.1, 142.4, 160.0, 169.4. HRMS (ESI) m/z calcd for $C_{19}H_{19}ClNaO_5S^+$ $[M+Na]^+$ 417.0534, found 417.0532.

(*R*)-6-(([1,1'-biphenyl]-4-ylsulfonyl)methyl)-6-phenyltetrahydro-2*H*-pyran-2-one

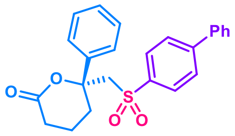

Compound 9d. (82% yield, 91% ee (*R*)). Colorless oil, 66.7 mg at 0.20 mmol scale. The ee of **9d** was determined by HPLC analysis: (Chiralcel AD-H column, 1.0 mL/min, hexane/isopropanol = 80/20, 210 nm, 25 °C, t_{major} = 38.25 min (*R*), t_{minor} = 28.22 min (*S*)); $[\alpha]^{16}_{D}$ −20.0 (*c* 1.81, $CHCl_3$) for 91% ee (*R*). ^1H NMR (400 MHz, $CDCl_3$) δ 1.42 – 1.58 (m, 1H), 1.76 – 1.88 (m, 1H), 2.35 – 2.61 (m, 3H), 2.87 – 2.99 (m, 1H), 3.68 – 3.79 (m, 2H), 7.26 – 7.36 (m, 5H), 7.39 – 7.49 (m, 3H), 7.54 – 7.60 (m, 2H), 7.64 – 7.71 (m, 2H), 7.84 – 7.90 (m, 2H). ^{13}C NMR (101 MHz, $CDCl_3$) δ 16.3, 29.3, 30.5, 66.6, 84.4, 125.0, 127.4, 127.8, 128.4, 128.5, 128.6, 129.0, 129.1, 139.1, 139.2, 141.3, 146.7, 169.6. HRMS (ESI) m/z calcd for $C_{24}H_{22}NaO_4S^+$ $[M+Na]^+$ 429.1131, found 429.1131.

(*R*)-6-(((4-(tert-butyl)phenyl)sulfonyl)methyl)-6-phenyltetrahydro-2*H*-pyran-2-one

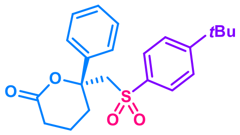

Compound 9e. (71% yield, 89% ee (*R*)). Colorless oil, 54.9 mg at 0.20 mmol scale. The ee of **9e** was determined by HPLC analysis: (Chiralcel AD-H column, 1.0 mL/min, hexane/isopropanol = 80/20, 210 nm, 25 °C, t_{major} = 13.63 min (*R*), t_{minor} = 10.81 min (*S*)); $[\alpha]^{16}_{D}$ −13.2 (*c* 1.80, $CHCl_3$) for 89% ee (*R*). ^1H NMR (400 MHz, $CDCl_3$) δ 1.25

(s, 9H), 1.39 – 1.52 (m, 1H), 1.68 – 1.81 (m, 1H), 2.28 – 2.55 (m, 3H), 2.70 – 2.90 (m, 1H), 3.50 – 3.70 (m, 2H), 7.05 – 7.31 (m, 5H), 7.42 (d, *J* = 8.2 Hz, 2H), 7.67 (d, *J* = 8.2 Hz, 2H). ^{13}C NMR (101 MHz, $CDCl_3$) δ 16.3, 29.4, 30.4, 31.1, 35.3, 66.6, 84.5, 125.1, 126.3, 127.9, 128.5, 129.1, 137.6, 141.4, 157.8, 169.8. HRMS (ESI) m/z calcd for $C_{22}H_{26}NaO_4S^+$ $[M+Na]^+$ 409.1444, found 409.1450.

(*R*)-6-(((2-chlorophenyl)sulfonyl)methyl)-6-phenyltetrahydro-2*H*-pyran-2-one

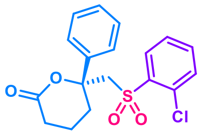

Compound 9f. (61% yield, 80% ee (*R*)). White solid, 44.5 mg at 0.20 mmol scale. The ee of **9f** was determined by HPLC analysis: (Chiralcel IA column, 1.0 mL/min, hexane/isopropanol = 90/10, 210 nm, 25 °C, t_{major} = 38.30 min (*R*), t_{minor} = 42.64 min (*S*)); $[\alpha]^{16}_{D}$ −9.6 (*c* 1.39, $CHCl_3$) for 80% ee (*R*). ^{1}H NMR (400 MHz, $CDCl_3$) δ 1.46 – 1.58 (m, 1H), 1.76 – 1.92 (m, 1H), 2.31 – 2.53 (m, 2H), 2.53 – 2.65 (m, 1H), 2.77 – 2.92 (m, 1H), 3.97 (d, *J* = 15.2 Hz, 1H), 4.14 (d, *J* = 15.2 Hz, 1H), 7.23 – 7.46 (m, 6H), 7.48 – 7.69 (m, 2H), 7.97 (d, *J* = 8.0 Hz, 1H). ^{13}C NMR (101 MHz, $CDCl_3$) δ 16.2, 29.3, 30.7, 64.1, 84.2, 125.1, 127.5, 128.6, 129.1, 131.4, 131.9, 132.5, 134.9, 137.8, 140.8, 169.5. HRMS (ESI) m/z calcd for $C_{18}H_{17}ClNaO_4S^+$ $[M+Na]^+$ 387.0428, found 387.0428.

(*R*)-6-(((3-chlorophenyl)sulfonyl)methyl)-6-phenyltetrahydro-2*H*-pyran-2-one

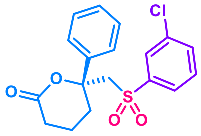

Compound 9g. (67% yield, 94% ee (*R*)). Colorless oil, 48.9 mg at 0.20 mmol scale. The ee of **9g** was determined by HPLC analysis: (Chiralcel IA column, 1.0 mL/min, hexane/isopropanol = 80/20, 210 nm, 25 °C, t_{major} = 13.68 min (*R*), t_{minor} = 16.18 min (*S*)); $[\alpha]^{16}_{D}$ −24.7 (*c* 0.96, $CHCl_3$) for 94% ee (*R*). ^{1}H NMR (400 MHz, $CDCl_3$) δ 1.43 – 1.58 (m, 1H), 1.76 – 1.87 (m, 1H), 2.38 – 2.56 (m, 3H), 2.75 – 2.90 (m, 1H), 3.65 – 3.76 (m, 2H), 7.21 – 7.36 (m, 5H), 7.44 (t, *J* = 8.0 Hz, 1H), 7.52 – 7.57 (m, 1H), 7.67 – 7.78 (m, 2H). ^{13}C NMR (101 MHz, $CDCl_3$) δ 16.2, 29.2, 30.6, 66.4, 84.2, 125.0, 126.2, 128.2, 128.6, 129.0, 130.5, 133.9, 135.3, 140.8, 142.2, 169.4. HRMS (ESI) m/z calcd for $C_{18}H_{17}ClNaO_4S^+$ $[M+Na]^+$ 387.0428, found 387.0426.

(*R*)-6-(((4-chlorophenyl)sulfonyl)methyl)-6-phenyltetrahydro-2*H*-pyran-2-one

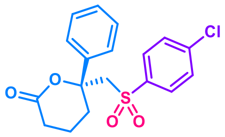

Compound 9h. (66% yield, 90% ee (*R*)). Colorless oil, 48.2 mg at 0.20 mmol scale. The ee of **9h** was determined by HPLC analysis: (Chiralcel OJ-H column, 1.0 mL/min, hexane/isopropanol = 50/50, 210 nm, 25 °C, t_{major} = 23.33 min (*R*), t_{minor} = 33.32 min (*S*)); $[\alpha]^{16}_{D}$ −27.8 (*c* 0.93, $CHCl_3$) for 90% ee (*R*). ^{1}H NMR (400 MHz, $CDCl_3$) δ 1.42 – 1.56 (m, 1H), 1.76 – 1.86 (m, 1H), 2.38 – 2.58 (m, 3H), 2.80 – 2.94 (m, 1H), 3.63 –

3.75 (m, 2H), 7.23 – 7.36 (m, 5H), 7.43 – 7.48 (m, 2H), 7.71 – 7.78 (m, 2H). ^{13}C NMR (101 MHz, $CDCl_3$) δ 16.2, 29.3, 30.7, 66.5, 84.3, 124.9, 128.5, 129.1, 129.5, 129.6, 139.1, 140.5, 141.1, 169.5. HRMS (ESI) m/z calcd for $C_{18}H_{17}ClNaO_4S^+$ $[M+Na]^+$ 387.0428, found 387.0428.

(*R*)-6-phenyl-6-(((4-(trifluoromethyl)phenyl)sulfonyl)methyl)tetrahydro-2*H*-pyran-2-one

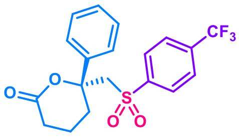

Compound 9i. (66% yield, 80% ee (*R*)). White solid, 52.6 mg at 0.20 mmol scale. The ee of **9i** was determined by HPLC analysis: (Chiralcel IA column, 1.0 mL/min, hexane/isopropanol = 80/20, 210 nm, 25 °C, t_{major} = 11.74 min (*R*), t_{minor} = 13.08 min (*S*)); $[\alpha]^{16}_D$ −21.9 (*c* 1.20, $CHCl_3$) for 80% ee (*R*). ^1H NMR (400 MHz, $CDCl_3$) δ 1.47 – 1.63 (m, 1H), 1.79 – 1.93 (m, 1H), 2.39 – 2.66 (m, 3H), 2.83 – 2.95 (m, 1H), 3.78 (s, 2H), 7.21 – 7.44 (m, 5H), 7.78 (d, *J* = 8.2 Hz, 2H), 7.98 (d, *J* = 8.2 Hz, 2H). ^{19}F NMR (376 MHz, $CDCl_3$) δ -63.2. ^{13}C NMR (101 MHz, $CDCl_3$) δ 16.2, 29.3, 30.9, 66.4, 84.3, 120.5 (q, *J* = 274.6 Hz), 125.0, 126.4 (q, *J* = 3.5 Hz)., 128.7, 128.8, 129.2, 135.4 (q, *J* = 33.5 Hz)., 140.9, 144.0, 169.5. HRMS (ESI) m/z calcd for $C_{19}H_{17}F_3NaO_4S^+$ $[M+Na]^+$ 421.0692, found 421.0690.

(*R*)-5-((morpholinosulfonyl)methyl)-5-(*p*-tolyl)dihydrofuran-2(3*H*)-one

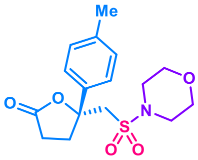

Compound 12a. (93% yield, 95% ee (*R*)). White solid, 63.1 mg at 0.20 mmol scale. The ee of **12a** was determined by HPLC analysis: (Chiralcel AD-H column, 1.0 mL/min, hexane/isopropanol = 50/50, 210 nm, 25 °C, t_{major} = 18.11 min (*R*), t_{minor} = 8.97 min (*S*)); $[\alpha]^{15}_D$ +9.3 (*c* 1.33, $CHCl_3$) for 95% ee (*R*). ^1H NMR (400 MHz, $CDCl_3$) δ 2.38 (s, 3H), 2.44 – 2.54 (m, 1H), 2.55 – 2.65 (m, 1H), 2.72 – 2.84 (m, 1H), 3.11 – 3.31 (m, 5H), 3.44 – 3.57 (m, 2H), 3.65 – 3.80 (m, 4H), 7.20 – 7.34 (m, 4H). ^{13}C NMR (101 MHz, $CDCl_3$) δ 21.2, 28.4, 32.7, 45.6, 58.4, 66.5, 84.6, 124.5, 129.7, 138.7, 139.2, 175.7. HRMS (ESI) m/z calcd for $C_{16}H_{21}NNaO_5S^+$ $[M+Na]^+$ 362.1033, found 362.1031.

(*R*)-4-(((5-oxo-2-(p-tolyl)tetrahydrofuran-2-yl)methyl)sulfonyl)butanenitrile

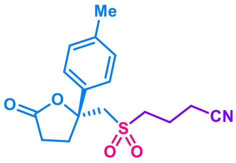

Compound 12b. (94% yield, 99% ee (*R*)). White solid, 60.4 mg at 0.20 mmol scale. The ee of **12b** was determined by HPLC analysis: (Chiralcel IB column, 1.0 mL/min, hexane/isopropanol = 50/50, 210 nm, 25 °C, t_{major} = 26.48 min (*R*), t_{minor} = 19.86 min (*S*)); $[\alpha]^{16}_D$ +10.5 (*c* 1.66, $CHCl_3$) for 99% ee (*R*). ^1H NMR (400 MHz, $CDCl_3$) δ 2.02 – 2.15 (m, 2H), 2.30 (s, 3H), 2.35 – 2.58 (m, 4H), 2.58 – 2.68 (m, 1H), 2.88 – 2.99 (m,

1H), 2.99 – 3.12 (m, 2H), 3.50 – 3.64 (m, 2H), 7.20 (m, 4H). ^{13}C NMR (101 MHz, $CDCl_3$) δ 16.4, 18.2, 21.2, 27.9, 33.1, 53.6, 62.6, 84.2, 118.2, 124.4, 130.0, 138.0, 139.2, 175.3. HRMS (ESI) m/z calcd for $C_{16}H_{19}NNaO_4S^+$ $[M+Na]^+$ 344.0927, found 344.0918.

(*R*)-5-(p-tolyl)-5-(tosylmethyl)dihydrofuran-2(3*H*)-one

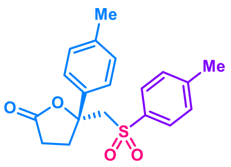

Compound 12c. (95% yield, 99% ee (*R*)). White solid, 65.4 mg at 0.20 mmol scale. The ee of **12c** was determined by HPLC analysis: (Chiralcel IA column, 1.0 mL/min, hexane/isopropanol = 90/10, 210 nm, 25 °C, t_{major} = 40.51 min (*R*), t_{minor} = 46.26 min (*S*)); $[\alpha]^{16}_D$ −4.0 (*c* 1.74, $CHCl_3$) for 99% ee (*R*). ^{1}H NMR (400 MHz, $CDCl_3$) δ 2.34 (s, 3H), 2.45 (s, 3H), 2.47 – 2.56 (m, 1H), 2.60 – 2.69 (m, 1H), 2.77 – 2.89 (m, 1H), 3.28 – 3.40 (m, 1H), 3.67 – 3.79 (m, 2H), 7.00 – 7.26 (m, 4H), 7.31 (d, *J* = 8.0 Hz, 2H), 7.62 – 7.79 (m, 2H). ^{13}C NMR (101 MHz, $CDCl_3$) δ 21.0, 21.7, 28.3, 32.4, 65.2, 84.8, 124.5, 127.9, 129.5, 129.8, 137.6, 138.5, 138.8, 144.9, 175.4. HRMS (ESI) m/z calcd for $C_{19}H_{20}NaO_4S^+$ $[M+Na]^+$ 367.0975, found 367.0974.

4. Transformations of 5a

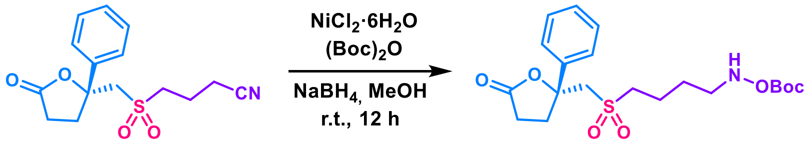

5a
99% ee

13
83% yield, 99% ee

A mixture of **5a** (30.7 mg, 0.1 mmol, 1 equiv), $NiCl_2·6H_2O$ (71.4 mg, 0.3 mmol, 3 equiv), Boc_2O (65.4 mg, 0.3 mmol, 3 equiv) in 2 mL of MeOH was added to a 15 mL vial, the resulting mixture was stirred at 0 °C and $NaBH_4$ (75.7 mg, 2 mmol, 20 equiv) was added slowly. Then the mixture was warmed up to room temperature and stirred overnight. The mixture was neutralized by saturated sodium bicarbonate solution and extracted with EA (10 mL×3). The combined organic phase was washed with brine, dried Na_2SO_4, and filtered. The filtrates were concentrated under reduced pressure. The residue was purified by silica gel chromatography to give the desired product **13** (34.2 mg, 83%) as a colorless oil.

***tert*-butyl (*R*)-(4-(((5-oxo-2-phenyltetrahydrofuran-2-yl)methyl)sulfonyl)butyl) carbamate**

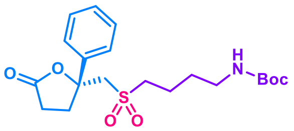

Compound 13. (83% yield, 99% ee (*R*)). Colorless oil, 34.2 mg at 0.10 mmol scale. The ee of **13** was determined by

HPLC analysis: (Chiralcel AD-H column, 1.0 mL/min, hexane/isopropanol = 70/30, 210 nm, 25 °C, t_{major} = 10.60 min (*R*), t_{minor} = 7.74 min (*S*)); $[\alpha]^{17}_{D}$ −9.3 (*c* 1.31, $CHCl_3$) for 99% ee (*R*). ^{1}H NMR (400 MHz, $CDCl_3$) δ 1.35 (s, 9H), 1.46 – 1.56 (m, 2H), 1.66 – 1.78 (m, 2H), 2.30 – 2.47 (m, 1H), 2.47 – 2.59 (m, 1H), 2.60 – 2.77 (m, 1H), 2.78 – 2.98 (m, 2H), 2.98 – 3.10 (m, 2H), 3.11 – 3.24 (m, 1H), 3.51 (q, J = 15.41 Hz, 2H), 4.57 (s, 1H), 7.26 – 7.44 (m, 5H). ^{13}C NMR (101 MHz, $CDCl_3$) δ 19.1, 27.9, 28.4, 28.8, 29.7, 32.8, 39.5, 55.2, 61.5, 84.3, 124.4, 128.8, 129.2, 141.6, 156.0, 175.3. HRMS (ESI) m/z calcd for $C_{20}H_{29}NNaO_6S^+$ $[M+Na]^+$ 434.1608, found 434.1600.

5. Transformations of 7a

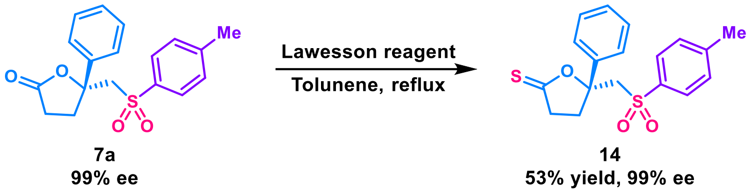

A solution of **7a** (66.1 mg, 0.2 mmol) and Lawesson reagent (181.8 mg, 0.45 mmol) in toluene (2.0 mL) was stirred and refluxed. After 12 h, the mixture was concentrated under reduced pressure. The residue was purified by flash chromatography on silica gel to afford the corresponding product **14** as a white solid in 53% yield (36.7 mg).

(*R*)-5-phenyl-5-(tosylmethyl)dihydrofuran-2(3*H*)-thione

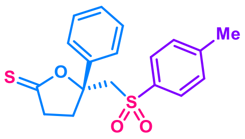

Compound 14. (53% yield, 99% ee (*R*)). White solid, 36.7 mg at 0.20 mmol scale. The ee of **14** was determined by HPLC analysis: (Chiralcel AD-H column, 1.0 mL/min, hexane/isopropanol = 85/15, 210 nm, 25 °C, t_{major} = 17.65 min (*R*), t_{minor} = 16.10 min (*S*)); $[\alpha]^{25}_{D}$ +9.5 (*c* 0.84, $CHCl_3$) for 99% ee (*R*). ^{1}H NMR (400 MHz, $CDCl_3$) δ 2.29 (s, 3H), 2.51 – 2.63 (m, 1H), 2.70 – 2.88 (m, 1H), 3.08 – 3.35 (m, 2H), 3.62 – 3.82 (m, 2H), 7.12 – 7.28 (m, 7H), 7.51 – 7.64 (m, 2H). ^{13}C NMR (101 MHz, $CDCl_3$) δ 21.8, 33.9, 44.1, 64.3, 94.5, 124.8, 128.1, 128.9, 129.0, 130.0, 137.3, 140.5, 145.1, 219.8. HRMS (ESI) m/z calcd for $C_{18}H_{19}O_3S_2^+$ $[M+H]^+$ 347.0770, found : 347.0768.

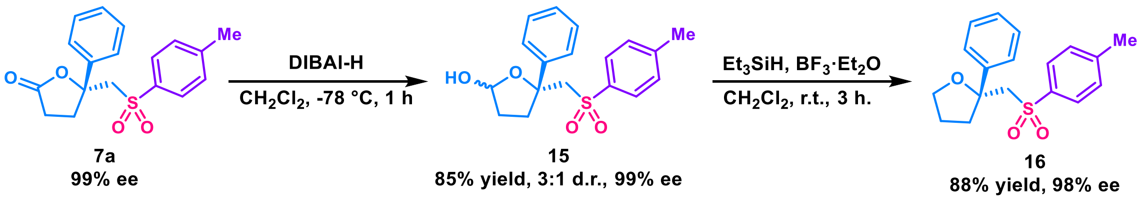

To a solution of **7a** (66.1 mg, 0.2 mmol) in CH_2Cl_2 (1 mL) was added a solution of DIBAL-H (1M in toluene, 0.2 mL, 0.22 mmol) at -78 °C and stirred for 1 hours.

The reaction was quenched by addition H_2O (0.4 mL) and NaOH (0.4 mL, 2N) and then the aqueous layer then extracted with CH_2Cl_2 (3 × 20 mL). The combined organics were then dried ($NaSO_4$) and concentrated in vacuo. The residue was purified by flash chromatography on silica gel to afford the corresponding product **15** as a white solid in 85% yield.

$BF_3{\cdot}OEt_2$ (0.12 mL, 0.50 mmol) was added dropwise to the solution of **15** (24.2 mg, 0.1 mmol) and Et_3SiH (63.3 mg, 0.4 mmol,) in dry CH_2Cl_2 (0.3 mL) at 0 °C under argon atmosphere. The mixture was allowed to warm to -10 °C during 1.5 h. Then saturated aqueous sodium bicarbonate solution (1 mL) was added and the mixture extracted with diethyl ether (10 mL) and dried ($MgSO_4$). Then the mixture was concentrated under reduced pressure. The residue was purified by flash chromatography on silica gel to afford the corresponding product **16** as a white solid in 88% yield

(5*R*)-5-phenyl-5-(tosylmethyl)tetrahydrofuran-2-ol

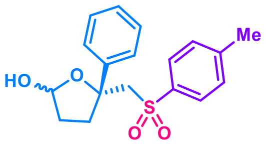

Compound 15. (85% yield, 3:1 dr, 99% ee). White solid, 56.5 mg at 0.20 mmol scale. The ee of **15** was determined by HPLC analysis: (Chiralcel AD-H column, 0.8 mL/min, hexane/isopropanol = 70/30, 210 nm, 25 °C, t_{major} = 14.77 min, 16.46 min, t_{minor} = 13.38 min, 18.61 min); $[\alpha]^{17}_{D}$ −5.8 (*c* 0.73, $CHCl_3$) for 3:1 dr, 99% ee. ^1H NMR (400 MHz, $CDCl_3$) δ 1.76 – 1.86 (m, 1H), 1.90 – 2.01 (m, 1H), 2.14 – 2.24 (m, 1H), 2.33 (s, 3H), 2.82 – 3.00 (m, 1H), 3.34 – 3.60 (m, 1H), 3.70 (s, 2H), 5.51 – 5.60 (m, 1H), 7.12 – 7.34 (m, 7H), 7.50 –7.72 (m, 2H). ^{13}C NMR (101 MHz, $CDCl_3$) 21.6, 33.1, 33.3, 35.3, 35.4, 65.9, 66.2, 84.8, 85.3, 99.8, 100.0, 124.9, 125.2, 127.3, 127.4, 127.7, 128.0, 128.2, 128.3, 129.56, 129.64, 138.1, 138.2, 144.0, 144.2, 144.4, 145.6. HRMS (ESI) m/z calcd for $C_{18}H_{20}NaO_4S^+$ $[M+Na]^+$ 355.0975, found : 355.0973.

(*R*)-2-phenyl-2-(tosylmethyl)tetrahydrofuran

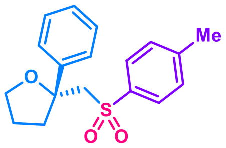

Compound 16. (88% yield, 98% ee (*R*)). White solid, 27.8 mg at 0.20 mmol scale. The ee of **16** was determined by HPLC analysis: (Chiralcel IB column, 0.8 mL/min, hexane/isopropanol = 95/5, 210 nm, 25 °C, t_{major} = 30.82min (*R*), t_{minor} = 29.50 min (*S*)); $[\alpha]^{17}_{D}$ –26.6 (*c* 0.91, $CHCl_3$) for 98% ee (*R*). ^1H NMR (400 MHz, $CDCl_3$) δ 1.69 – 1.87 (m, 1H), 1.97 – 2.15 (m, 1H), 2.25 – 2.36 (m, 1H), 2.43 (s, 3H), 2.74 – 2.91 (m, 1H), 3.58 – 3.77 (m, 2H), 3.79 – 3.91 (m, 1H), 3.98 (q, *J* = 8.0 Hz, 1H), 7.11 – 7.42

(m, 7H), 7.66 (d, J = 8.0 Hz, 2H). ^{13}C NMR (101 MHz, $CDCl_3$) δ 21.6, 25.2, 36.9, 65.4, 68.1, 84.0, 125.2, 127.2, 127.8, 128.3, 129.4, 138.4, 144.0, 144.6. HRMS (ESI) m/z calcd for $C_{18}H_{20}NaO_3S^+$ $[M+Na]^+$ 339.1025, found : 339.1019.

6. Transformations of 7t

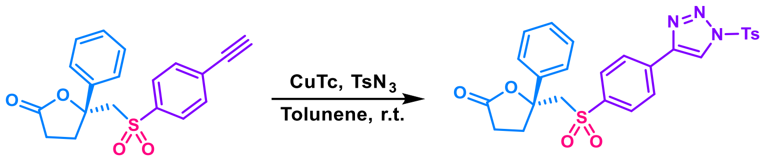

Copper(I) thiophene-2-carboxylate hydrate (CuTc) (1.9 mg, 0.01 mmol, 10 mol%) and **7t** (33.0 mg, 0.1 mmol, 1.0 equiv) were added to PhMe (1.0 mL) in a 15 mL vial. Then TsN_3 (21.7 mg, 0.11 mmol, 1.1 equiv) were slowly injected into the flask. The reaction was stirred at room temperature for one day. After terminal alkynes were completely reacted (monitored by TLC analysis). The reaction mixture was filtered to remove inorganic compound, the solvent was dried over and purified by flash column chromatography to get product **17** as a white solid in 82% yield.

(*R*)-5-phenyl-5-(((4-(1-tosyl-1H-1,2,3-triazol-4-yl)phenyl)sulfonyl)methyl)di-hydrofuran-2(3*H*)-one

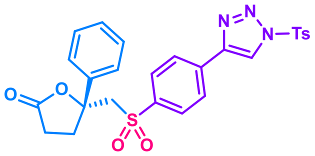

Compound 17. (82% yield, 98% ee (*R*)). White solid, 44.1 mg at 0.10 mmol scale. The ee of **17** was determined by HPLC analysis: (Chiralcel AD-H column, 1.0 mL/min, hexane/isopropanol = 50/50, 210 nm, 25 °C, t_{major} = 16.76 min (*R*), t_{minor} = 6.65 min (*S*)); $[\alpha]^{17}_D$ −1.8 (*c* 1.47, $CHCl_3$) for 98% ee (*R*). ^{1}H NMR (400 MHz, DMSO-d_6) δ 2.19 – 2.48 (m, 5H), 2.66 – 2.80 (m, 1H), 2.81 – 3.01 (m, 1H), 4.23 – 4.54 (m, 2H), 7.17 – 7.41 (m, 5H), 7.58 (d, J = 8.0 Hz, 2H), 7.76 (d, J = 8.0 Hz, 2H), 7.92 – 8.22 (m, 4H), 9.62 (s, 1H). ^{13}C NMR (101 MHz, DMSO-d_6) δ 21.8, 27.8, 34.9, 63.3, 84.9, 123.5, 125.3, 126.5, 128.2, 128.7, 128.8, 128.9, 131.4, 132.5, 133.7, 141.0, 141.7, 145.9, 148.5, 176.0. HRMS (ESI) m/z calcd for $C_{19}H_{17}N_3NaO_4S^+$ $[M–Ts+Na]^+$ 406.0832, found : 406.0829.

7. Nonlinear effect studies[13]

Investigation into non-linear effects was conducted by comparing the ee value of the chiral ligand **L1** and that of the product **7a**. 6 reactions containing **L1** of racemic, 20%, 40%, 60%, 80%, and > 99% optical purity were run in parallel. The nonlinear

effect study revealed a linear relationship between the ee of the product **7a** and the enantiopurity of the ligand **L1**, indicating a single chiral ligand is likely involved in the enantiodetermining transition state.

In an argon fulfilled glovebox, SOgen (177.0 mg, 0.41 mmol), 1-methyl-4-vinylbenzene (47.3 mg, 0.40 mmol), were added into chamber A with a magnetic stirring bar, followed by addition of tetradecane (1.0 mL). $Cu(MeCN)_4PF_6$ (3.7 mg, 0.010 mmol, 5 mol%), **L1** with different ee values (9.9 mg, 0.012 mmol, 6 mol%), Compound **1a** (35.2 mg, 0.2 mmol, 1.0 equiv), diazonium salt **4a** (82.4 mg, 0.4 mmol, 2 equiv), Na_2CO_3 (23.3 mg, 0.22 mmol, 1.1 equiv) and 4Å MS (60 mg) and 2-Me-THF (2.0 mL) were added into chamber B with a magnetic stirring bar. The two-chamber was sealed and removed out of the glovebox. The chamber A was allowed to stir at 100 °C using heating mantle with 600-800 rpm stirring speed for 10 min. The two-chamber was allowed to stir at room temperature for 12 h. Upon completion, the reaction mixture was chromatographed on SiO_2 column. The residue was purified by flash silica gel column chromatography using dichloromethane/ethyl acetate (v/v =50/1) as eluent to afford pure products **7a**. The ee of **7a** was determined by HPLC analysis: (Chiralcel AD-H column, 1.0 mL/min, hexane/isopropanol = 90/10, 210 nm, t_{major} = 48.06 min (*R*), t_{minor} = 42.32 min (*S*)); A graph of ee of product vs. ee of ligand was then plotted.

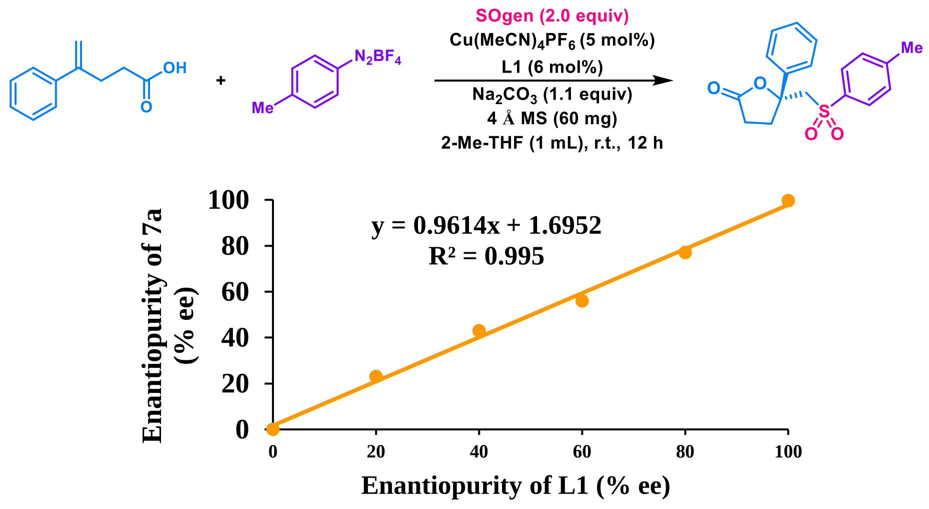

Table 1. Linear correlationa

entry	ee of **L1** (%)	ee of **7a** (%)[a]
1	0	0
2	20	23
3	40	43
4	60	56
5	80	77
6	100	99.6

a.The ee of the ligand was calculated by mixing different amounts of ligand (*R*,*R*)-**L1** and ligand (*S*,*S*)-**L1**, ee = [m((*R*,*R*)-**L1**) – m((*S*,*S*)-L1)]/[m((*S*,*S*)-**L1**) + m((*R*,*R*)-**L1**)].

8. Mechanistic experiments

a) Radical trapping experiment with TEMPO.

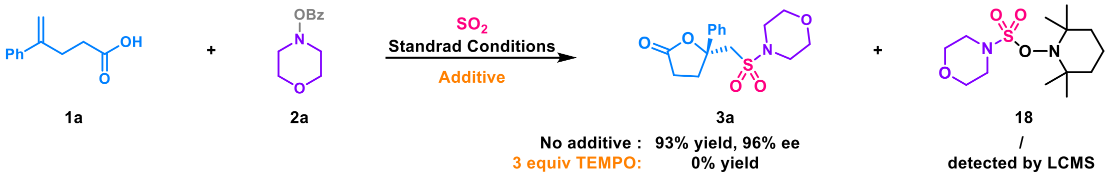

In an argon fulfilled glovebox, SOgen (133.8 mg, 0.31 mmol), 1-methyl-4-vinylbenzene (35.4 mg, 0.30 mmol), were added into chamber A with a magnetic stirring bar, followed by addition of tetradecane (1.0 mL). $Cu(MeCN)_4PF_6$ (7.5 mg, 0.020 mmol, 10 mol%), **L1** (19.9 mg, 0.024 mmol, 12 mol%), TEMPO (93.6 mg, 0.6 mmol, 3 equiv), Compound **1a** (35.2 mg, 0.2 mmol, 1.0 equiv), Compound **2a** (124.3mg, 0.6 mmol, 3 equiv), Na_2CO_3 (23.3 mg, 0.22 mmol, 1.1 equiv) and 4Å MS (60 mg) and 2-Me-THF (2.0 mL) were added into chamber B with a magnetic stirring bar. The two-chamber was sealed and removed out of the glovebox. The chamber A was allowed to stir at 100 °C using heating mantle with 600-800 rpm stirring speed for 10 min. The two-chamber was allowed to stir at room temperature for 12 h. TLC, GC and LC-MS analysis demonstrated the product **7a** is not founded. The sulfonyl radical combined with TEMPO **18** were detected by LC-MS.

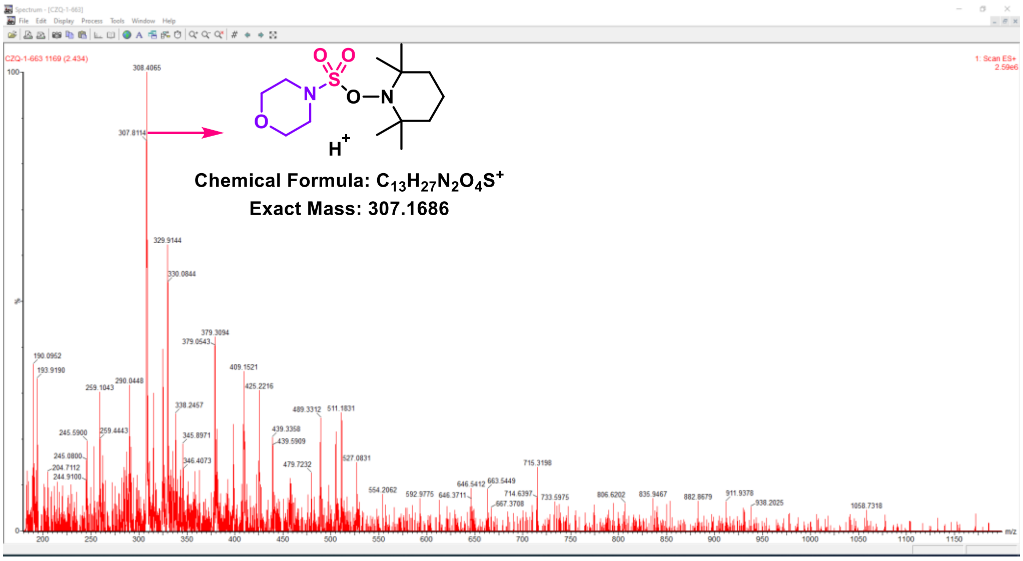

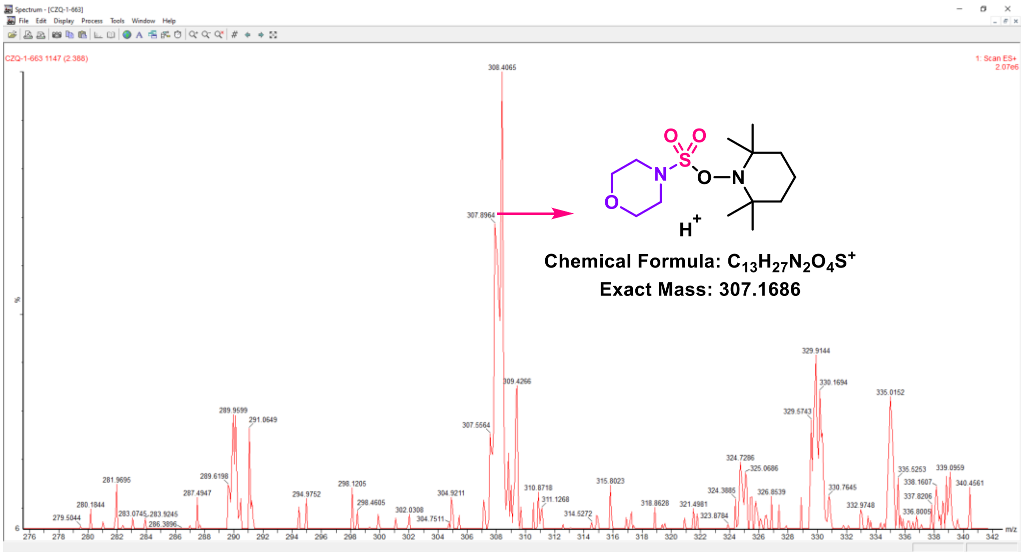

Figure S1 Radical trapping experiment with TEMPO

b) Radical trapping experiment with 1,1-diphenylethylene.

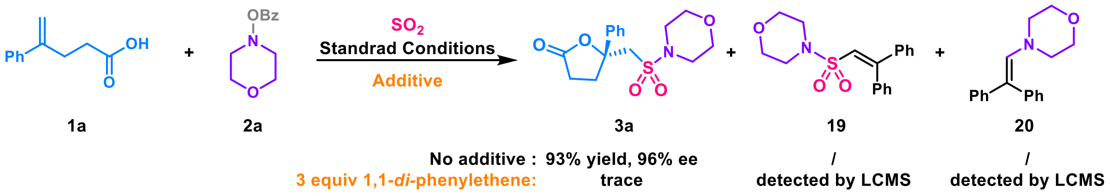

In an argon fulfilled glovebox, SOgen (133.8 mg, 0.31 mmol), 1-methyl-4-vinylbenzene (35.4 mg, 0.30 mmol), were added into chamber A with a magnetic stirring bar, followed by addition of tetradecane (1.0 mL). $Cu(MeCN)_4PF_6$ (7.5 mg, 0.020 mmol, 10 mol%), **L1** (19.9 mg, 0.024 mmol, 12 mol%), 1,1-*di*-phenylethene (0.6 mmol, 93.6 mg), Compound **1a** (35.2 mg, 0.2 mmol, 1.0 equiv), Compound **2a** (124.3mg, 0.6 mmol, 3 equiv), Na_2CO_3 (23.3 mg, 0.22 mmol, 1.1 equiv) and 4Å MS

(60 mg) and 2-Me-THF (2.0 mL) were added into chamber B with a magnetic stirring bar. The two-chamber was sealed and removed out of the glovebox. The chamber A was allowed to stir at 100 °C using heating mantle with 600-800 rpm stirring speed for 10 min. The two-chamber was allowed to stir at room temperature for 12 h. TLC, GC and LC-MS analysis demonstrated the product **7a** is trace. The Sulfonyl radical combined with 1,1- diphenylethylene **19** and nitrogen radical combined with 1,1-diphenylethylene **20** were detected by LC-MS.

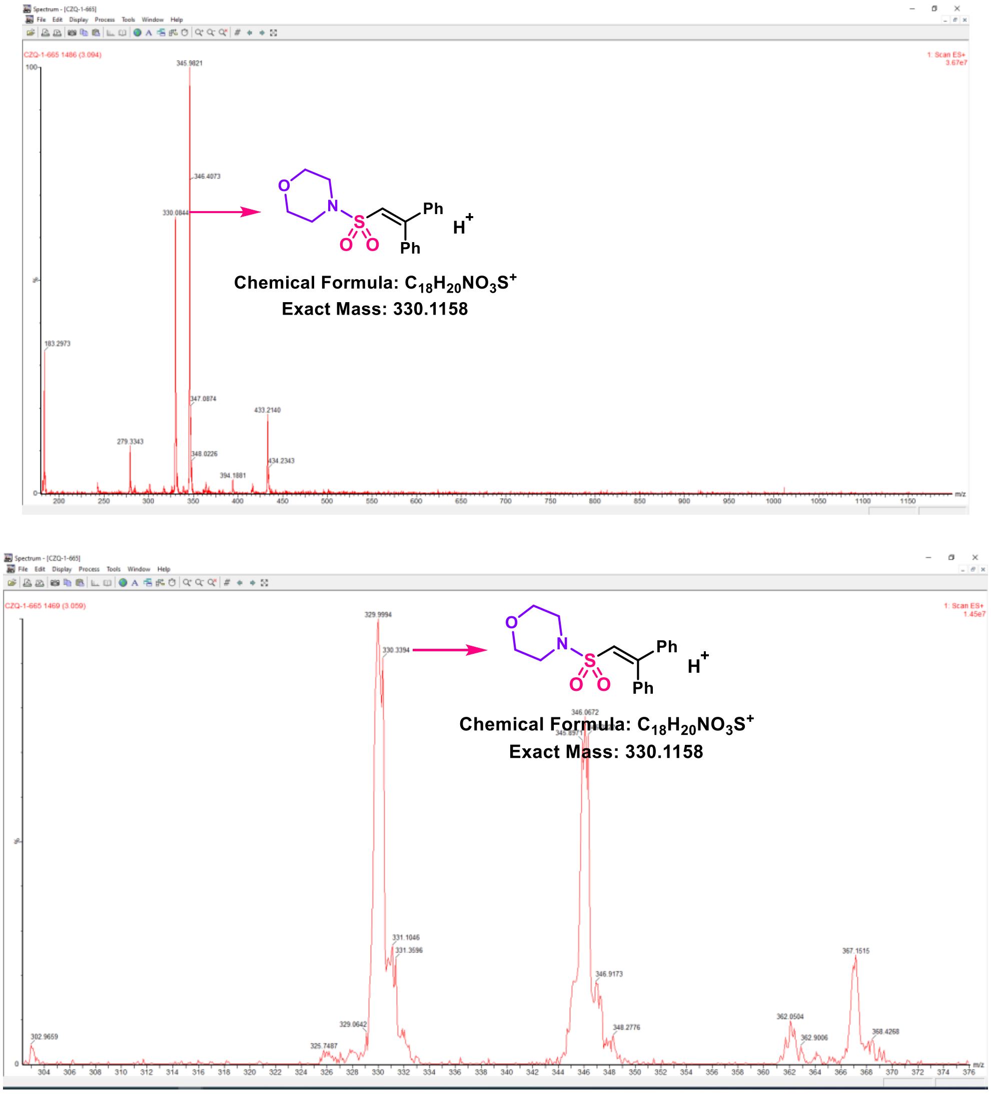

Figure S2 Radical trapping experiment with 1,1-diphenylethylene.

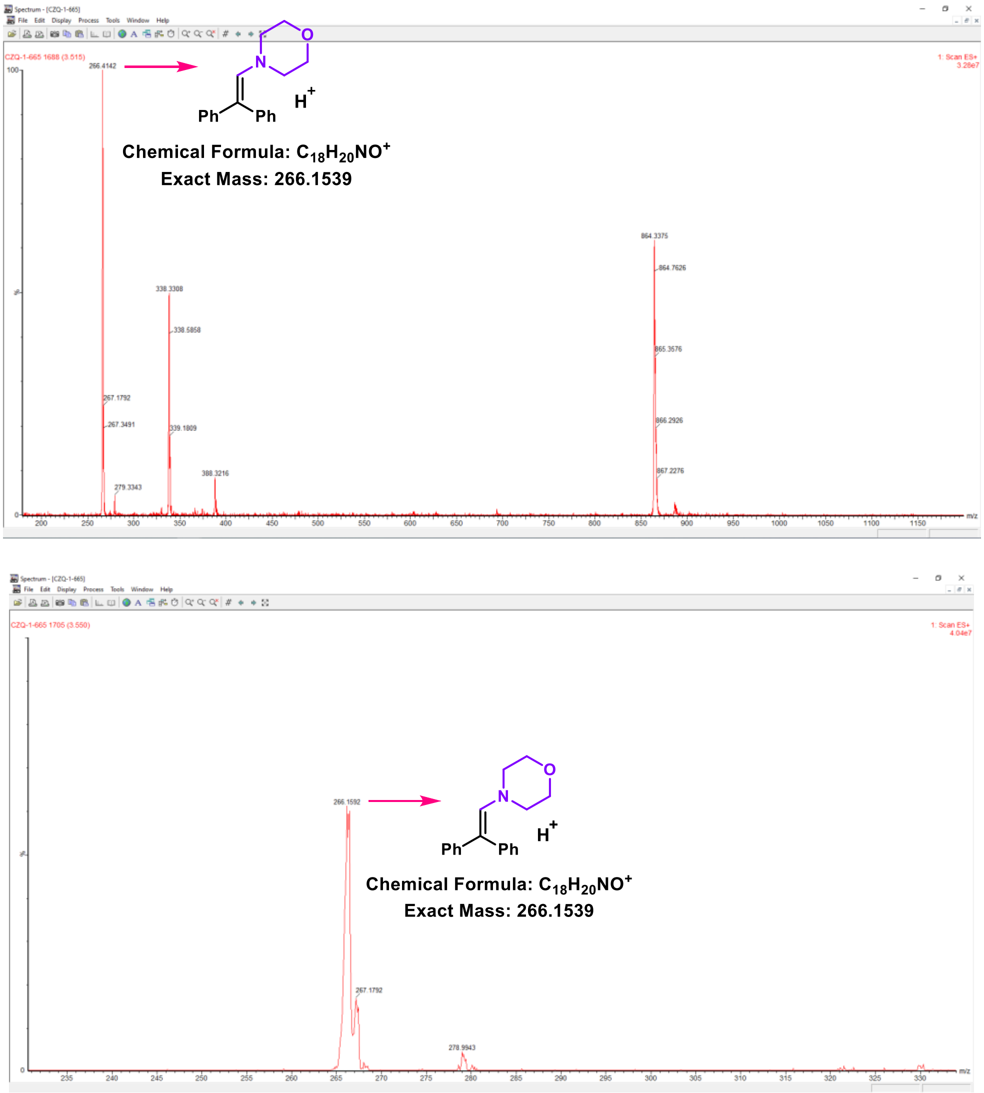

Figure S3 Radical trapping experiment with 1,1-diphenylethylene.

9. Single crystal X-Ray diffraction data.

X-ray crystal data for **3b** with 50% probability for the ellipsoid contour (CCDC 2240321). A crystal of **3b** was obtained by recrystallization from CH_2Cl_2/Hexane.

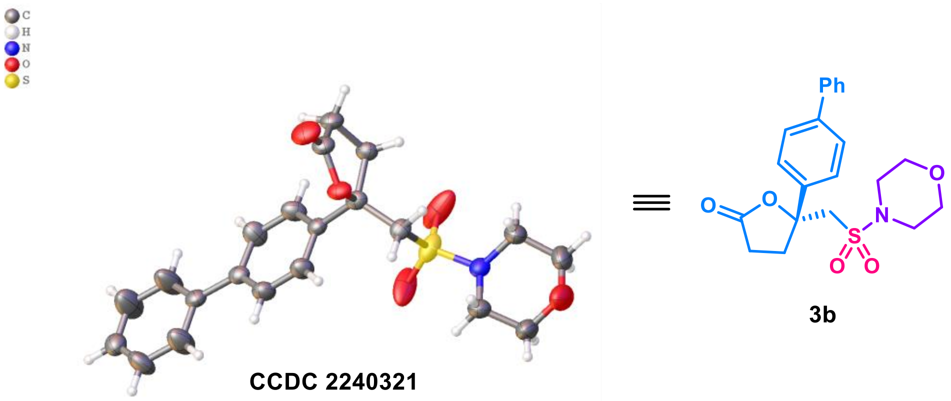

Figure S5. ORTEP illustration of **3b**.

Table 2 Crystal data and structure refinement for mo_LZ_CZQ_1_471_240K_0m_a.

Identification code	mo_LZ_CZQ_1_471_240K_0m_a
Empirical formula	$C_{21}H_{23}NO_5S$
Formula weight	401.46
Temperature/K	240.0
Crystal system	orthorhombic
Space group	$P2_12_12_1$
a/Å	5.6194(9)
b/Å	17.438(3)
c/Å	19.664(4)
α/°	90
β/°	90
γ/°	90
Volume/Å^3	1926.9(6)
Z	4
ρ_{calc}g/cm^3	1.384
μ/mm^{-1}	0.201
F(000)	848.0
Crystal size/mm^3	0.46 × 0.12 × 0.04
Radiation	MoKα (λ = 0.71073)
2Θ range for data collection/°	4.142 to 49.978
Index ranges	$-6 \le h \le 6$, $-20 \le k \le 20$, $-23 \le l \le 23$
Reflections collected	14629
Independent reflections	3373 [R_{int} = 0.0964, R_{sigma} = 0.0763]
Data/restraints/parameters	3373/0/253
Goodness-of-fit on F^2	1.057
Final R indexes [I>=2σ (I)]	R_1 = 0.0644, wR_2 = 0.1605
Final R indexes [all data]	R_1 = 0.0852, wR_2 = 0.1801
Largest diff. peak/hole / e Å^{-3}	0.35/-0.47
Flack parameter	0.01(9)

Table 3 Fractional Atomic Coordinates (×10^4) and Equivalent Isotropic Displacement Parameters (Å^2×10^3) for mo_LZ_CZQ_1_471_240K_0m_a. U_{eq} is defined as 1/3 of of the trace of the orthogonalised U_{IJ} tensor.

Atom	*x*	*y*	*z*	U(eq)
S1	4867(3)	5823.8(9)	2050.0(8)	52.7(5)
O1	909(6)	4338.0(19)	3124(2)	40.1(10)
O2	-180(8)	3195(2)	3502(3)	61.0(12)
O5	4883(12)	6505(3)	2460(2)	91(2)
O4	6982(8)	5395(4)	1981(3)	93(2)
C1	3479(9)	5270(3)	3623(3)	35.2(12)
N2	4066(9)	6078(3)	1289(2)	42.4(12)
O3	2088(12)	6383(3)	9(2)	84.2(18)
C21	3809(12)	7342(3)	6742(3)	52.6(15)
C2	5510(15)	6835(5)	6592(4)	91(3)
C3	5487(15)	6450(5)	5979(4)	82(3)
C4	3771(10)	6545(3)	5508(3)	40.2(13)
C5	3690(9)	6127(3)	4841(3)	36.6(12)
C6	1834(12)	6199(3)	4393(3)	49.4(15)
C7	1699(11)	5791(4)	3797(3)	48.1(15)
C8	3118(8)	4769(3)	2994(3)	35.8(12)
C9	2565(10)	5219(3)	2345(3)	37.8(12)
C10	1895(13)	6543(4)	1226(3)	55.6(17)
C11	1926(17)	6924(4)	545(3)	74(2)
C12	1420(11)	3602(3)	3340(3)	43.7(14)
C13	4014(10)	3479(3)	3323(3)	45.9(14)
C14	4962(10)	4130(3)	2886(3)	41.7(12)
C15	2060(14)	7463(4)	6290(4)	70(2)
C16	2013(13)	7085(5)	5680(4)	74(2)
C17	5499(10)	5624(4)	4653(3)	52.3(16)
C18	5386(10)	5216(3)	4050(3)	47.0(14)
C19	4227(15)	5517(4)	734(3)	63(2)
C20	4164(18)	5938(5)	70(4)	76(2)

Table 4 Anisotropic Displacement Parameters (Å^2×10^3) for mo_LZ_CZQ_1_471_240K_0m_a. The Anisotropic displacement factor exponent takes the form: -2π^2[h^{2}a*^{2}U$_{11}$+2hka*b*U$_{12}$+…].

Atom	U_{11}	U_{22}	U_{33}	U_{23}	U_{13}	U_{12}
S1	50.5(9)	61.6(10)	46.1(9)	18.2(7)	-12.2(7)	-23.6(7)
O1	39.5(19)	24.8(18)	56(3)	5.4(17)	3.3(17)	-5.2(15)
O2	62(3)	39(2)	82(3)	16(2)	4(2)	-13(2)
O5	153(5)	77(3)	42(3)	-1(2)	-25(3)	-70(4)
O4	37(2)	137(5)	104(4)	72(4)	-6(3)	-9(3)
C1	41(3)	28(3)	37(3)	5(2)	3(2)	2(2)
N2	58(3)	31(2)	38(3)	1(2)	-2(2)	-2(2)
O3	143(5)	66(3)	43(3)	-4(3)	-24(3)	20(4)
C21	73(4)	41(3)	44(4)	2(3)	2(3)	-3(3)
C2	100(6)	113(7)	60(5)	-23(5)	-32(5)	49(6)
C3	84(5)	96(6)	65(5)	-28(4)	-23(4)	53(5)
C4	50(3)	28(3)	43(3)	6(2)	2(3)	1(2)
C5	39(3)	28(3)	43(3)	4(2)	4(2)	-2(2)
C6	56(4)	42(3)	50(4)	-2(3)	-4(3)	15(3)
C7	47(3)	49(3)	49(4)	-4(3)	-9(3)	13(3)
C8	30(2)	33(3)	44(3)	2(2)	1(2)	-1(2)
C9	39(3)	34(3)	40(3)	2(2)	-5(2)	-8(2)
C10	79(4)	37(3)	50(4)	-1(3)	2(3)	13(3)
C11	128(6)	49(4)	44(4)	4(3)	-2(4)	28(4)
C12	55(4)	27(3)	49(4)	3(3)	3(3)	-2(2)
C13	59(4)	28(3)	51(4)	2(3)	-1(3)	4(3)
C14	44(3)	36(3)	45(3)	1(2)	2(3)	8(2)
C15	80(5)	66(5)	63(4)	-26(4)	2(4)	27(4)
C16	73(5)	88(5)	62(5)	-23(4)	-18(4)	37(4)
C17	43(3)	63(4)	51(4)	-9(3)	-10(3)	6(3)
C18	40(3)	43(3)	58(4)	-7(3)	-7(3)	8(2)
C19	101(6)	41(3)	49(4)	-1(3)	8(4)	17(3)
C20	134(7)	55(4)	40(4)	-5(3)	8(4)	12(5)

Table 5 Bond Lengths for mo_LZ_CZQ_1_471_240K_0m_a.

Atom	Atom	Length/Å	Atom	Atom	Length/Å
S1	O5	1.435(5)	C2	C3	1.379(10)
S1	O4	1.410(6)	C3	C4	1.347(9)
S1	N2	1.624(5)	C4	C5	1.501(8)
S1	C9	1.767(5)	C4	C16	1.406(8)
O1	C8	1.473(6)	C5	C6	1.371(8)
O1	C12	1.382(6)	C5	C17	1.392(8)
O2	C12	1.189(7)	C6	C7	1.372(8)
C1	C7	1.394(8)	C8	C9	1.530(8)
C1	C8	1.528(7)	C8	C14	1.535(7)
C1	C18	1.365(8)	C10	C11	1.494(9)
N2	C10	1.471(8)	C12	C13	1.474(9)
N2	C19	1.468(8)	C13	C14	1.521(8)
O3	C11	1.418(8)	C15	C16	1.369(9)
O3	C20	1.406(10)	C17	C18	1.384(8)
C21	C2	1.335(10)	C19	C20	1.498(10)
C21	C15	1.342(10)			

Table 6 Bond Angles for mo_LZ_CZQ_1_471_240K_0m_a.

Atom	Atom	Atom	Angle/°	Atom	Atom	Atom	Angle/°
O5	S1	N2	107.1(3)	C5	C6	C7	122.9(6)
O5	S1	C9	108.3(3)	C6	C7	C1	120.5(6)
O4	S1	O5	119.2(4)	O1	C8	C1	105.3(4)
O4	S1	N2	106.8(3)	O1	C8	C9	103.6(4)
O4	S1	C9	109.4(3)	O1	C8	C14	102.9(4)
N2	S1	C9	105.2(3)	C1	C8	C9	114.0(4)
C12	O1	C8	110.6(4)	C1	C8	C14	116.0(4)
C7	C1	C8	118.5(5)	C9	C8	C14	113.2(4)
C18	C1	C7	117.2(5)	C8	C9	S1	115.6(4)
C18	C1	C8	124.2(5)	N2	C10	C11	108.2(6)
C10	N2	S1	117.3(4)	O3	C11	C10	111.8(5)
C19	N2	S1	119.1(4)	O1	C12	C13	109.5(5)
C19	N2	C10	110.8(5)	O2	C12	O1	118.7(5)
C20	O3	C11	110.9(6)	O2	C12	C13	131.8(5)
C2	C21	C15	118.9(6)	C12	C13	C14	104.5(5)
C21	C2	C3	120.5(7)	C13	C14	C8	103.1(4)
C4	C3	C2	123.2(6)	C21	C15	C16	121.2(6)
C3	C4	C5	124.3(5)	C15	C16	C4	121.3(6)
C3	C4	C16	114.8(6)	C18	C17	C5	121.2(5)
C16	C4	C5	120.9(5)	C1	C18	C17	121.8(5)
C6	C5	C4	122.7(5)	N2	C19	C20	108.7(5)
C6	C5	C17	116.2(5)	O3	C20	C19	111.4(7)
C17	C5	C4	121.1(5)				

Table 7 Torsion Angles for mo_LZ_CZQ_1_471_240K_0m_a.

A	B	C	D	Angle/°	A	B	C	D	Angle/°
S1	N2	C10	C11	161.4(5)	C6	C5	C17	C18	-0.9(9)
S1	N2	C19	C20	-162.0(6)	C7	C1	C8	O1	-58.7(6)
O1	C8	C9	S1	178.8(4)	C7	C1	C8	C9	54.2(6)
O1	C8	C14	C13	-29.7(5)	C7	C1	C8	C14	-171.6(5)
O1	C12	C13	C14	-15.7(7)	C7	C1	C18	C17	3.7(9)
O2	C12	C13	C14	164.5(7)	C8	O1	C12	O2	175.9(5)
O5	S1	N2	C10	-50.7(5)	C8	O1	C12	C13	-3.9(6)
O5	S1	N2	C19	171.2(6)	C8	C1	C7	C6	173.5(5)
O5	S1	C9	C8	-77.9(5)	C8	C1	C18	C17	-172.7(5)
O4	S1	N2	C10	-179.4(5)	C9	S1	N2	C10	64.4(5)
O4	S1	N2	C19	42.5(6)	C9	S1	N2	C19	-73.8(5)
O4	S1	C9	C8	53.4(5)	C9	C8	C14	C13	-140.9(4)
C1	C8	C9	S1	65.0(5)	C10	N2	C19	C20	57.4(8)
C1	C8	C14	C13	84.6(5)	C11	O3	C20	C19	58.8(8)
N2	S1	C9	C8	167.9(4)	C12	O1	C8	C1	-100.3(5)
N2	C10	C11	O3	57.6(9)	C12	O1	C8	C9	139.7(5)
N2	C19	C20	O3	-57.7(9)	C12	O1	C8	C14	21.5(5)
C21	C2	C3	C4	-1.4(15)	C12	C13	C14	C8	27.9(6)
C21	C15	C16	C4	1.2(13)	C14	C8	C9	S1	-70.5(5)
C2	C21	C15	C16	-0.9(13)	C15	C21	C2	C3	0.9(13)
C2	C3	C4	C5	-179.3(8)	C16	C4	C5	C6	-5.5(9)
C2	C3	C4	C16	1.6(13)	C16	C4	C5	C17	176.3(6)
C3	C4	C5	C6	175.5(7)	C17	C5	C6	C7	1.5(9)
C3	C4	C5	C17	-2.7(9)	C18	C1	C7	C6	-3.1(9)
C3	C4	C16	C15	-1.5(12)	C18	C1	C8	O1	117.7(5)
C4	C5	C6	C7	-176.9(6)	C18	C1	C8	C9	-129.4(5)
C4	C5	C17	C18	177.4(5)	C18	C1	C8	C14	4.8(7)
C5	C4	C16	C15	179.4(7)	C19	N2	C10	C11	-57.2(7)
C5	C6	C7	C1	0.6(10)	C20	O3	C11	C10	-59.2(9)
C5	C17	C18	C1	-1.7(9)					

Table 8 Hydrogen Atom Coordinates (Å×10^4) and Isotropic Displacement Parameters (Å^2×10^3) for mo_LZ_CZQ_1_471_240K_0m_a.

Atom	*x*	*y*	*z*	U(eq)
H21	3836	7610	7157	63
H2	6732	6739	6907	110
H3	6730	6105	5886	98
H6	599	6542	4498	59
H7	396	5864	3505	58
H9A	1148	5534	2427	45
H9B	2172	4854	1984	45
H10A	1842	6930	1587	67
H10B	484	6216	1267	67
H11A	471	7227	490	89
H11B	3285	7275	522	89
H13A	4396	2980	3120	55
H13B	4690	3500	3782	55
H14A	5047	3980	2406	50
H14B	6544	4292	3038	50
H15	844	7815	6394	84
H16	782	7189	5370	89
H17	6821	5561	4940	63
H18	6657	4891	3932	56
H19A	2891	5157	760	76
H19B	5711	5225	772	76
H20A	5562	6272	37	91
H20B	4234	5568	-304	91

10. DFT calculations

Method:

All calculations were carried out with the Gaussian 16 software[14]. The PBE0 functional[15] was adopted for all calculations in combination with the D3BJ dispersion correction[16]. The def2-SVP basis set was employed for geometry optimization and frequency calculations, and the def2-TZVP basis set was used to for single point calculations. The SMD solvation model[17] was applied to simulate solvent effect in tetrahydrofuran solution. The harmonic frequency calculations were carried out to verify that there are no imaginary frequencies for the intermediate compounds, reactants, and products and only one imaginary frequency for the transition states. The stability of the wavefunctions for all the species have been tested with the option of stable=opt. The non-covalent interactions were analyzed by independent gradient model based on Hirshfeld partition (IGMH) analyses in Multiwfn[18]. The plots of IGMH were rendered by VMD program[19].

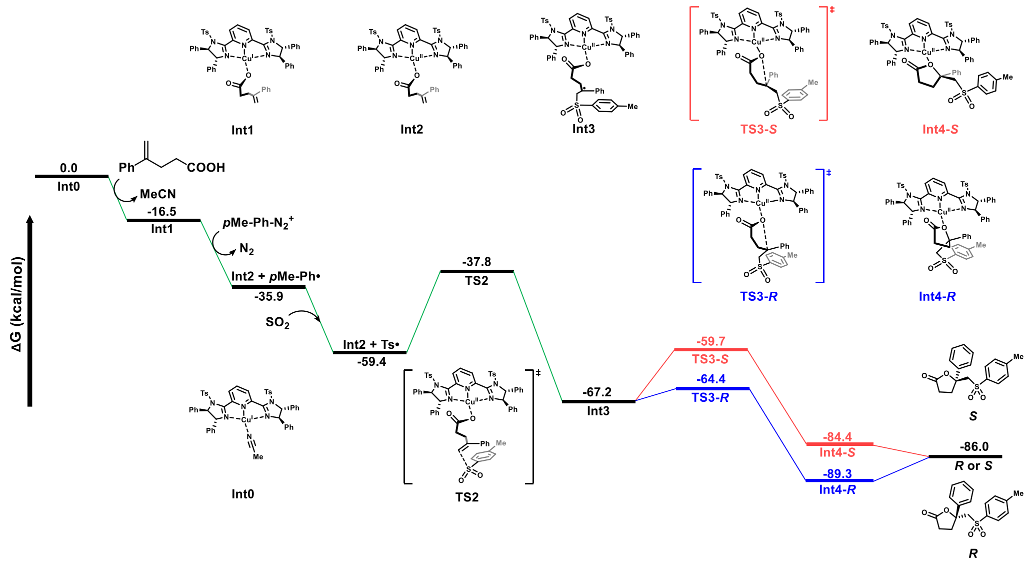

Figure S5. The potential energy surfaces of the catalytic cycle. All Gibbs free energies are in kcal/mol compared to **int0**.

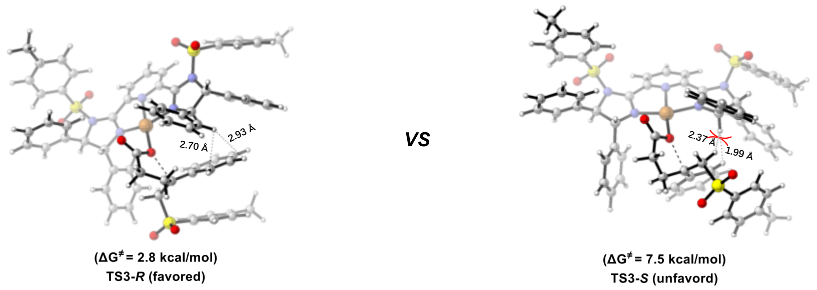

Figure S6. Optimized structures for the enantio-determining transition states **TS3-*R***

and **TS3-*S***.

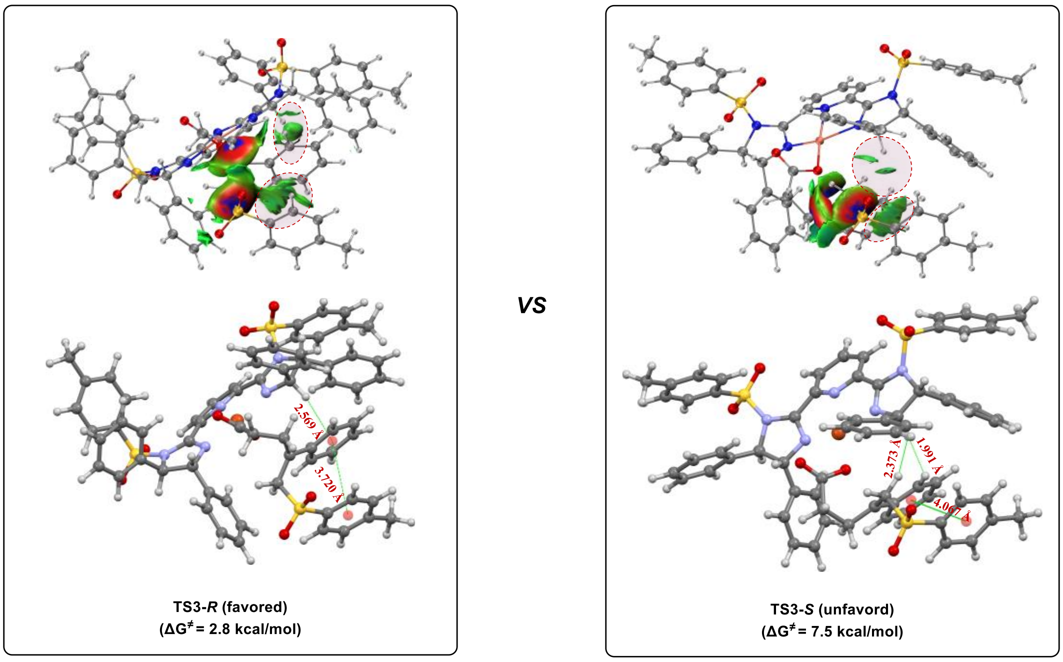

Figure S7. Non-bonding interactions and non-bonding interaction distances in the enantio-determining transition states **TS3-*R*** and **TS3-*S*.**

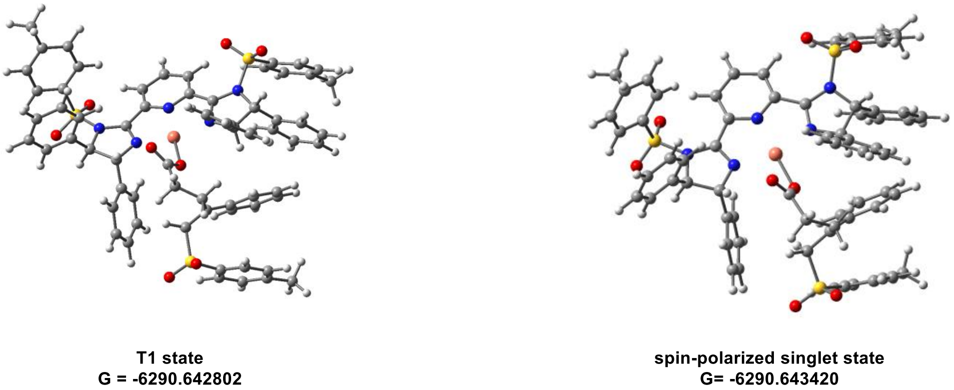

Figure S8. Energy comparison between T1 state and spin-polarized singlet state

To verify whether a six-membered ring transition state is formed, we performed a potential energy surface scan as follows: We fixed the distance between Cu-O (17-108) and gradually increased the distance between O-C (108-103) with an interval of 0.1 Å for each optimization. We searched for the lowest energy hexagonal ring structure in space by taking O as the center. If Cu (17) can bond with C (103), then it can form a low-energy stable position, indicating the existence of a hexagonal ring. However, if a stable structure with low energy cannot be found, it means that the formation of a hexagonal ring structure is impossible (Figure S10).

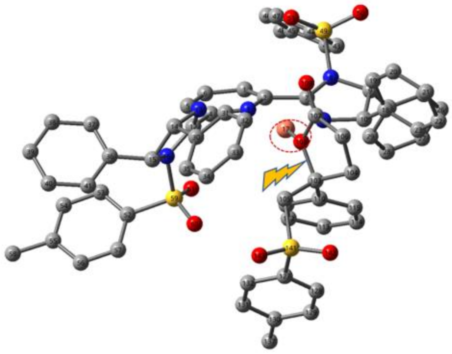

Figure S9 Method for verifying the existence of a six-membered ring transition state.

We conducted an energy scan at 30 different points and found energy fluctuations at points 9, 17, and 23. Upon observing the structures at points 9, 17, and 23, we found that their Cu-C distances are 3.457 Å, 2.076 Å, and 2.765 Å respectively. Although the Cu-C distance is closer in point 17, the fact that C is situated in a planar structure suggests the absence of a Cu-C bond. Furthermore, the energy level is significantly higher compared to the initial five-membered ring structure. Therefore, throughout the entire scanning process, no hexagonal ring structure with a Cu-C bond was observed.

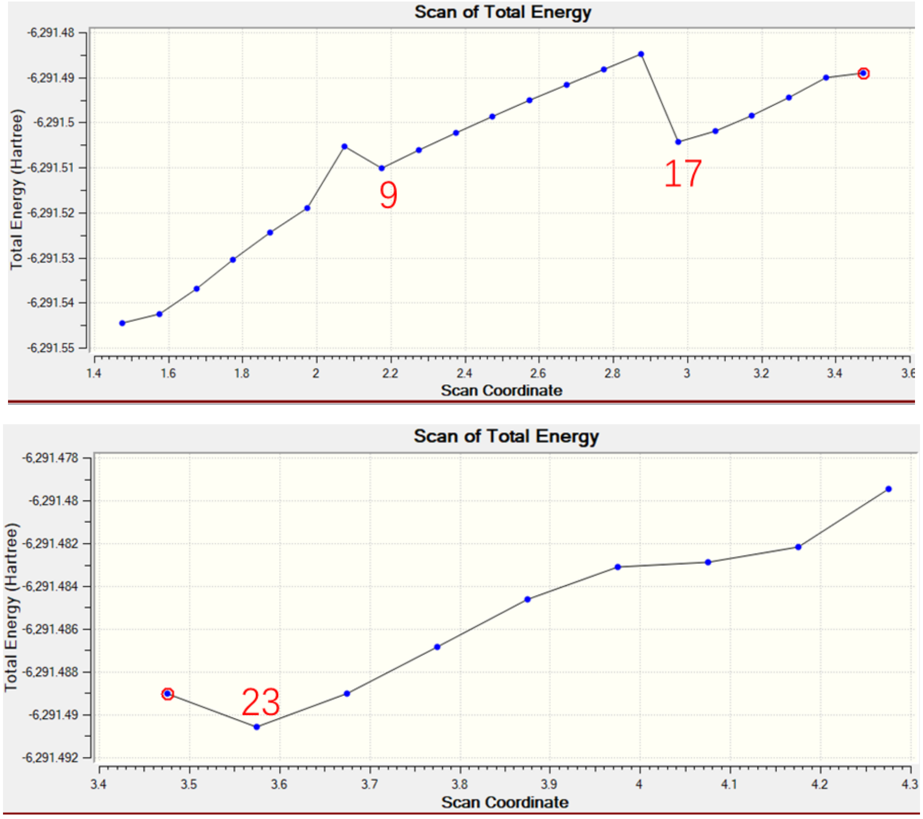

Figure S10 Energy scan at 30 different points

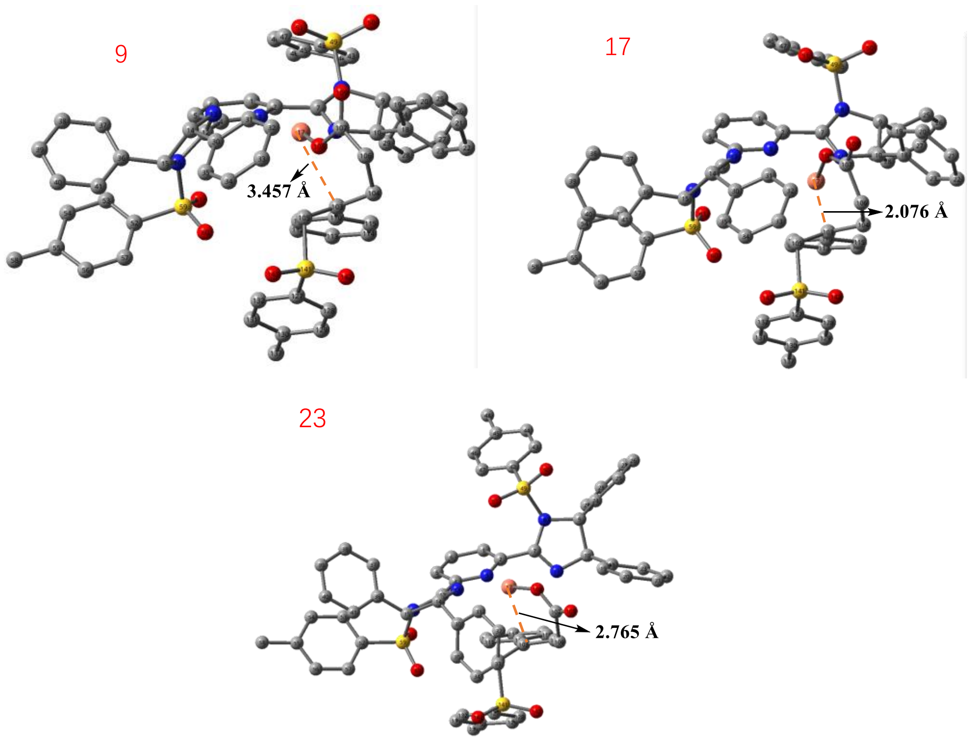

Figure S11 The structures at points 9, 17, and 23

Table 9 Calculated energies for all the species involved in the reactions (in Hartree)

Compounds	Thermal Correction	EE	G
Int0	0.753263	-5033.78551	-5033.03225
Ph-COO⁻	0.155531	-575.93681	-575.781279
MeCN	0.021344	-132.652347	-132.631003
4-Me-Ph-radical	0.082664	-270.660971	-270.578307
N_2	-0.012664	-109.441064	-109.453728
4-Me-Ph-N_2^+	0.094041	-379.946163	-379.852122
Int1	0.892084	-5477.100922	-5476.20884
Int2	0.898535	-5476.958381	-5476.05985
Ts-radical	0.090259	-819.139754	-819.049495
TS2	1.010542	-6296.085364	-6295.07482
Int3	1.018132	-6296.139803	-6295.12167
TS3-*R*	1.014507	-6296.131795	-6295.11729
TS3-*S*	1.01748	-6296.127251	-6295.10977
Int4-*R*	1.018024	-6296.174905	-6295.15688
Int4-*S*	1.023911	-6296.172993	-6295.14908
Cu-Product	0.714721	-4901.10717	-4900.39245
R-Product	0.278375	-1395.037595	-1394.75922
S-Product	0.278375	-1395.037595	-1394.75922
TS1	0.089018	-819.112326	-819.023308
SO_2	-0.017221	-548.416496	-548.433717

Table 10 Cartesian coordinates (in Å) of related structures

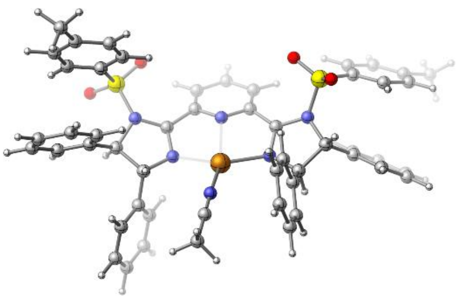

int0

Symbolic Z-matrix:

Charge = 1 Multiplicity = 1

C	-3.00654	-1.84733	2.81591
C	-2.23856	-1.39619	1.74463
N	-2.60662	-0.35058	1.00129
C	-3.71295	0.34171	1.30159
C	-4.53434	-0.03214	2.36508
C	-4.17279	-1.14984	3.11602
C	-0.85638	-1.81101	1.43384
N	-0.28967	-3.06272	1.58393
C	1.14743	-2.96265	1.26522
C	1.29432	-1.45042	0.88857
N	-0.03532	-0.8823	1.07884
C	-3.74209	1.61348	0.53461
N	-2.59245	2.17153	0.35113
C	-2.75876	3.34581	-0.48619
C	-4.28448	3.62565	-0.48645
N	-4.79562	2.30406	-0.04085
Cu	-0.98034	0.75247	0.16761
C	1.78165	-1.19745	-0.51542
C	1.04133	-1.67027	-1.60552
C	1.45929	-1.41002	-2.90718
C	2.62466	-0.67391	-3.13316
C	3.37044	-0.20719	-2.05149
C	2.94871	-0.4682	-0.74698
C	2.03118	-3.41901	2.39581
C	3.26557	-4.00548	2.10213
C	4.10048	-4.44328	3.12895
C	3.70376	-4.30504	4.45897
C	2.47373	-3.71671	4.75684
C	1.64316	-3.27186	3.73065
C	-1.92878	4.52495	-0.06183
C	-1.50111	5.44996	-1.02007
C	-0.76489	6.57104	-0.63972
C	-0.44437	6.77375	0.70285
C	-0.86503	5.85197	1.662
C	-1.6064	4.73398	1.28224
C	-4.80427	4.1033	-1.81466
C	-4.56038	3.37995	-2.98931
C	-5.01959	3.85181	-4.21447
C	-5.72324	5.0569	-4.28209
C	-5.96876	5.78161	-3.11812
C	-5.5122	5.30416	-1.88897
C	-0.57325	-5.548	2.55946
C	0.57739	-6.32632	2.43238
C	1.03922	-7.02045	3.5429
C	0.36909	-6.95088	4.77345
C	-0.80374	-6.18674	4.85642
C	-1.28118	-5.48095	3.75793
C	0.91714	-7.66036	5.97135
S	-1.07584	-4.53953	1.21023
O	-0.47385	-5.05094	-0.01383
O	-2.50251	-4.26581	1.27165
C	-6.92981	1.75883	-1.66127
C	-6.47421	0.62113	-2.32717
C	-6.8595	0.4236	-3.64714
C	-7.70064	1.33564	-4.30287
C	-8.16211	2.45206	-3.59218
C	-7.7834	2.67295	-2.27338
C	-8.07523	1.1416	-5.73888
S	-6.46569	2.01408	0.01765
O	-7.07598	3.2431	0.49938
O	-6.64285	0.75891	0.73232
H	-2.67822	-2.68889	3.42449
H	-5.42234	0.54314	2.61492
H	-4.78743	-1.45261	3.96617
H	1.35185	-3.58095	0.37995
H	1.98215	-0.96397	1.59684
H	-2.44991	3.06133	-1.50634
H	-4.53803	4.36495	0.28998
H	0.1286	-2.24773	-1.4319
H	0.87399	-1.78313	-3.75138
H	2.95398	-0.47002	-4.15532
H	4.28742	0.36272	-2.2225
H	3.52906	-0.09691	0.10217
H	3.56626	-4.134	1.05842
H	5.06047	-4.90696	2.88822
H	4.35177	-4.65919	5.26468
H	2.15485	-3.6108	5.79688
H	0.67147	-2.83238	3.96765
H	-1.74786	5.2869	-2.07316

H	-0.43589	7.28796	-1.39632
H	0.13653	7.64999	1.00178
H	-0.61321	6.00424	2.71473
H	-1.93166	4.00776	2.03142
H	-4.01929	2.43113	-2.94482
H	-4.83001	3.2762	-5.12391
H	-6.08251	5.42765	-5.24541
H	-6.51982	6.72418	-3.16353
H	-5.70973	5.86736	-0.97342
H	1.10724	-6.37761	1.48007
H	1.94682	-7.62316	3.45686
H	-1.35167	-6.14257	5.80092
H	-2.19705	-4.89079	3.82917
H	1.2137	-8.69099	5.72445
H	1.82154	-7.14391	6.33481
H	0.19134	-7.69127	6.79551
H	-5.8259	-0.10073	-1.82546
H	-6.50515	-0.46123	-4.18197
H	-8.82624	3.16738	-4.0839
H	-8.1324	3.54817	-1.72404
H	-7.45682	1.78841	-6.38443
H	-7.92052	0.10293	-6.06279
H	-9.12504	1.41552	-5.92019
N	0.00902	1.58787	-1.29731
C	0.6708	2.16389	-2.0491
C	1.49432	2.89829	-2.97871
H	1.16244	3.94652	-3.01477
H	2.54336	2.86022	-2.65172
H	1.41191	2.45423	-3.98132

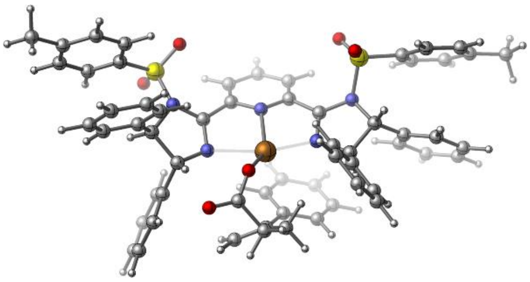

Int1

Symbolic Z-matrix:

Charge = 0 Multiplicity = 1

C	-2.77633	-3.56365	0.30388
C	-1.73931	-2.63537	0.37469
N	-1.95148	-1.3183	0.23288
C	-3.20744	-0.8509	0.11275
C	-4.30051	-1.71292	0.03387
C	-4.06981	-3.08603	0.11388
C	-0.3583	-2.90856	0.79673
N	0.34039	-4.10442	0.69112
C	1.581	-3.9732	1.49004
C	1.60446	-2.43517	1.76916
N	0.25355	-1.98966	1.46759
C	-3.2285	0.61413	0.30227
N	-2.36347	1.08806	1.1326
C	-2.43977	2.53575	1.12966
C	-3.74126	2.87821	0.34139
N	-4.01205	1.58391	-0.32623
Cu	-0.58802	-0.05893	1.21179
C	2.65299	-1.7435	0.92641
C	2.34873	-1.17804	-0.31381
C	3.36493	-0.66963	-1.12142
C	4.69283	-0.72377	-0.70071
C	5.00075	-1.26982	0.54534
C	3.98484	-1.7722	1.35474
C	1.54609	-4.86265	2.70395
C	2.56413	-5.79667	2.90792
C	2.52512	-6.66058	4.00258
C	1.46512	-6.59422	4.90515
C	0.44988	-5.65433	4.71417
C	0.49023	-4.79262	3.6214
C	-2.37822	3.14977	2.4988
C	-1.7418	4.38296	2.67222
C	-1.70601	4.98776	3.9284
C	-2.29906	4.35977	5.02379
C	-2.92907	3.12534	4.85755
C	-2.9709	2.52479	3.60057
C	-3.52301	4.02982	-0.60192
C	-2.69313	3.8847	-1.71964
C	-2.44258	4.96924	-2.55421
C	-3.01233	6.21407	-2.27696
C	-3.82825	6.36685	-1.15747
C	-4.08211	5.27739	-0.32271
C	0.68726	-6.65078	0.08105
C	1.82356	-7.38044	-0.25217
C	2.05562	-8.59246	0.3938
C	1.1745	-9.07313	1.36879
C	0.03011	-8.3162	1.6727
C	-0.22384	-7.11188	1.03425
C	1.44851	-10.3489	2.10175
S	0.42009	-5.08929	-0.69344
O	1.60496	-4.74374	-1.46891
O	-0.88895	-5.02468	-1.32582
C	-6.12697	2.75265	-1.54887
C	-5.68339	3.17715	-2.80273
C	-6.12941	4.39846	-3.28484

C	-7.01671	5.19357	-2.54066
C	-7.46292	4.72297	-1.29925
C	-7.02287	3.5044	-0.793
C	-7.45082	6.52553	-3.06549
S	-5.56942	1.21272	-0.92247
O	-6.41586	0.82601	0.20656
O	-5.36524	0.27127	-2.01504
H	-2.57719	-4.6287	0.41166
H	-5.31675	-1.33186	-0.04572
H	-4.91084	-3.78098	0.07388
H	2.44025	-4.2496	0.86514
H	1.82152	-2.25678	2.83258
H	-1.56141	2.88368	0.5577
H	-4.56178	3.11349	1.04031
H	1.31035	-1.12217	-0.64736
H	3.11331	-0.2241	-2.08724
H	5.48823	-0.32529	-1.33557
H	6.03602	-1.29249	0.89484
H	4.22807	-2.19767	2.33297
H	3.38694	-5.85827	2.19028
H	3.32317	-7.39378	4.14468
H	1.42917	-7.27352	5.76068
H	-0.38053	-5.59412	5.42232
H	-0.3128	-4.06408	3.47914
H	-1.25956	4.86077	1.81549
H	-1.20299	5.95014	4.05361
H	-2.26498	4.82953	6.01023
H	-3.3884	2.62562	5.71454
H	-3.45631	1.55387	3.47087
H	-2.24684	2.91135	-1.93833
H	-1.79666	4.84404	-3.42687
H	-2.81581	7.0657	-2.93328
H	-4.27529	7.3385	-0.93205
H	-4.72777	5.39419	0.55166
H	2.5232	-6.9937	-0.99523
H	2.94846	-9.17057	0.14261
H	-0.66667	-8.67609	2.434
H	-1.10448	-6.5227	1.29879
H	2.29361	-10.8962	1.66236
H	1.68954	-10.1392	3.15721
H	0.56587	-11.0069	2.09958
H	-4.99519	2.56027	-3.38285
H	-5.77959	4.74953	-4.2589
H	-8.16412	5.32431	-0.71534
H	-7.36776	3.13718	0.17514
H	-6.59691	7.22311	-3.08539
H	-7.82122	6.44419	-4.09906
H	-8.24011	6.97092	-2.44453
C	0.35263	-0.31114	5.49651
C	0.96488	-0.99875	4.30139
C	0.71753	0.92489	5.87865
C	2.0975	-0.25429	3.61732
C	1.62527	1.02364	2.86513
O	2.26802	2.07224	3.08994
O	0.64915	0.8625	2.10444
C	-0.7038	-1.04974	6.23906
C	-1.80072	-0.36739	6.79379
C	-2.78831	-1.04311	7.50566
C	-2.71143	-2.42612	7.6758
C	-1.63785	-3.12174	7.1213
C	-0.65132	-2.44354	6.40699
H	1.44721	1.50379	5.30357
H	0.27862	1.39713	6.76285
H	-1.88682	0.71123	6.6419
H	-3.63259	-0.48559	7.9204
H	-3.48818	-2.95895	8.23053
H	-1.5648	-4.20569	7.24512
H	0.18335	-3.00975	5.98846
H	1.29469	-2.00817	4.6041
H	0.16939	-1.15188	3.54972
H	2.89652	0.00385	4.33142
H	2.5417	-0.92841	2.86338

MeCN

Symbolic Z-matrix:

Charge = 0 Multiplicity = 1

C	-2.38198	-1.73649	0.03103
H	-2.02002	-2.77492	0.02291
H	-2.02	-1.22426	-0.87238
H	-3.48179	-1.74122	0.02275
C	-1.89921	-1.05363	1.21393
N	-1.5132	-0.50778	2.1591

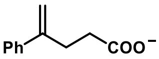

Symbolic Z-matrix:

Charge = -1 Multiplicity = 1

C	4.23984	9.87494	-10.2596
C	4.50357	11.34412	-10.0406
C	2.99572	9.37605	-10.3607
C	3.27637	12.21346	-9.83271
C	2.41538	12.37665	-11.1188
O	1.18777	12.17311	-10.9972

O	3.04773	12.70334	-12.1439
C	5.42344	8.98255	-10.3849
C	5.407	7.88952	-11.269
C	6.501	7.03597	-11.3845
C	7.6496	7.25729	-10.6234
C	7.6894	8.34415	-9.751
C	6.59381	9.19883	-9.63853
H	2.11863	10.03048	-10.3341
H	2.82467	8.30366	-10.4955
H	4.52445	7.72356	-11.8914
H	6.4605	6.19752	-12.0852
H	8.51116	6.59086	-10.7161
H	8.58298	8.5311	-9.14917
H	6.64667	10.03897	-8.94297
H	5.20303	11.45596	-9.19354
H	5.03633	11.73228	-10.9276
H	2.65135	11.834	-9.00787
H	3.61915	13.22254	-9.54214

4-Me-Ph-N_2^+

Symbolic Z-matrix:

Charge = 1 Multiplicity = 1

C	-2.47506	-0.20665	0.14585
C	-1.09594	-0.23018	0.1597
C	-0.42629	0.99778	0.00084
C	-1.09207	2.22527	-0.16915
C	-2.47231	2.20261	-0.1772
C	-3.18603	0.9991	-0.01865
H	-3.02398	-1.14331	0.26458
H	-0.53143	-1.15539	0.28538
H	-0.5257	3.14993	-0.29001
H	-3.01885	3.13845	-0.31106
C	-4.67429	0.99861	0.00272
H	-5.02508	0.97977	1.04952
H	-5.08908	1.89743	-0.47226
H	-5.08037	0.10187	-0.48708
N	0.93255	0.99706	0.01015
N	2.03979	0.99596	0.02008

N_2

Symbolic Z-matrix:

Charge = 0 Multiplicity = 1

N	3.63338	3.63338	2.07045
N	4.99403	4.99403	2.35537

4-Me-Ph radical

Symbolic Z-matrix:

Charge = 0 Multiplicity = 2

C	-2.47506	-0.20665	0.14585
C	-1.09594	-0.23018	0.1597
C	-0.42629	0.99778	0.00084
C	-1.09207	2.22527	-0.16915
C	-2.47231	2.20261	-0.1772
C	-3.18603	0.9991	-0.01865
H	-3.02398	-1.14331	0.26458
H	-0.53143	-1.15539	0.28538
H	-0.5257	3.14993	-0.29001
H	-3.01885	3.13845	-0.31106
C	-4.67429	0.99861	0.00272
H	-5.02508	0.97977	1.04952
H	-5.08908	1.89743	-0.47226
H	-5.08037	0.10187	-0.48708

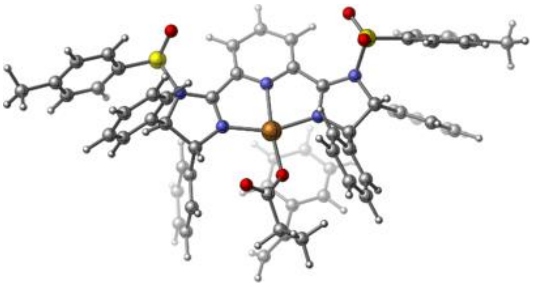

Int2

Symbolic Z-matrix:

Charge = 1 Multiplicity = 2

C	-3.47883	-3.55027	1.44144
C	-2.35099	-2.74874	1.33215
N	-2.4621	-1.42873	1.18387
C	-3.63172	-0.8203	1.07775
C	-4.81822	-1.54493	1.1899
C	-4.72314	-2.92068	1.38041
C	-0.9341	-3.13369	1.51871
N	-0.37549	-4.36802	1.33322
C	1.06683	-4.26055	1.68266
C	1.18762	-2.7959	2.21042
N	-0.1326	-2.2313	1.97673
C	-3.42268	0.64784	1.06
N	-2.44821	1.08132	1.78615
C	-2.38429	2.53858	1.67987
C	-3.54405	2.92045	0.70356
N	-4.05344	1.58853	0.29303
Cu	-0.87518	-0.32886	1.7968

C	2.31368	-2.01087	1.5969	H	1.33093	-2.82992	3.30192
C	2.16376	-1.38041	0.35818	H	-1.41336	2.80113	1.23376
C	3.2226	-0.66541	-0.19783	H	-4.33744	3.44109	1.26139
C	4.4413	-0.58602	0.47488	H	1.21386	-1.44118	-0.17968
C	4.59952	-1.22457	1.70605	H	3.09281	-0.16519	-1.16038
C	3.53904	-1.93251	2.26621	H	5.27081	-0.02344	0.03964
C	1.49442	-5.30403	2.67592	H	5.552	-1.16263	2.23783
C	2.71656	-5.95806	2.51122	H	3.65492	-2.41877	3.23878
C	3.12971	-6.9155	3.43785	H	3.34182	-5.72398	1.64554
C	2.32189	-7.22462	4.53115	H	4.08455	-7.42805	3.29798
C	1.1004	-6.5695	4.70017	H	2.64271	-7.97972	5.25291
C	0.68906	-5.61263	3.77706	H	0.46295	-6.81019	5.5546
C	-2.54461	3.23584	3.00758	H	-0.27295	-5.11083	3.90329
C	-1.71267	4.29881	3.36408	H	-0.88932	4.576	2.70494
C	-1.90668	4.9647	4.57414	H	-1.24716	5.79194	4.84816
C	-2.92815	4.57145	5.43746	H	-3.07401	5.09045	6.38814
C	-3.7664	3.51271	5.0833	H	-4.5705	3.20065	5.75437
C	-3.5782	2.85311	3.87169	H	-4.24242	2.02883	3.59608
C	-3.08014	3.77457	-0.44313	H	-1.9371	2.198	-1.386
C	-2.23768	3.2487	-1.42915	H	-1.13262	3.63751	-3.2379
C	-1.78635	4.05598	-2.46873	H	-1.81198	6.03265	-3.34357
C	-2.16558	5.39877	-2.52663	H	-3.28954	6.98036	-1.57591
C	-2.9919	5.92964	-1.53773	H	-4.10373	5.52946	0.27343
C	-3.44853	5.11874	-0.49894	H	1.63057	-6.41408	-1.29693
C	0.12561	-6.74711	0.21835	H	2.85066	-8.50973	-0.7346
C	1.27115	-7.0723	-0.50394	H	-0.02944	-9.36502	2.35558
C	1.9486	-8.2425	-0.17927	H	-1.27057	-7.29585	1.78762
C	1.50292	-9.0733	0.85635	H	3.0691	-10.5217	0.5305
C	0.33205	-8.72106	1.55023	H	2.71865	-10.164	2.24031
C	-0.36707	-7.56629	1.2374	H	1.60085	-11.1751	1.31127
C	2.26463	-10.2996	1.24457	H	-4.7616	2.03923	-2.89777
S	-0.69686	-5.246	-0.14101	H	-5.08013	4.08604	-4.26501
O	-0.00623	-4.53936	-1.20925	H	-7.45087	5.83802	-1.12009
O	-2.1385	-5.41921	-0.19862	H	-7.1196	3.79833	0.27033
C	-5.91193	2.82796	-1.23729	H	-5.45969	6.83503	-3.74326
C	-5.34324	2.87861	-2.513	H	-6.81627	6.1011	-4.61122
C	-5.52907	4.02352	-3.2708	H	-7.12089	7.06158	-3.13652
C	-6.27984	5.10613	-2.78076	C	0.40277	0.93997	5.6445
C	-6.8559	5.0077	-1.5078	C	1.62107	0.2763	5.04835
C	-6.67627	3.87342	-0.72423	C	0.23767	2.2711	5.66908
C	-6.43646	6.3424	-3.60627	C	2.36965	1.09916	4.01599
S	-5.67863	1.39907	-0.26179	C	1.54657	1.50538	2.80653
O	-6.54304	1.44693	0.913	O	1.74608	2.55367	2.22736
O	-5.65296	0.20417	-1.09299	O	0.6929	0.63054	2.33617
H	-3.38674	-4.62393	1.58952	C	-0.65331	0.03834	6.17221
H	-5.78735	-1.05217	1.16157	C	-2.00776	0.31555	5.91834
H	-5.63389	-3.51132	1.49455	C	-3.00953	-0.54655	6.36224
H	1.65969	-4.35764	0.76084	C	-2.67757	-1.69979	7.07408

C	-1.33576	-1.98464	7.33449
C	-0.33293	-1.13183	6.87792
H	0.98076	2.95487	5.2577
H	-0.64483	2.72553	6.12371
H	-2.26914	1.20618	5.34252
H	-4.05667	-0.31729	6.14666
H	-3.4615	-2.37501	7.42637
H	-1.06588	-2.88255	7.89551
H	0.71267	-1.37044	7.08704
H	2.30875	-0.04351	5.84898
H	1.3138	-0.66463	4.57251
H	2.8152	2.01158	4.43307
H	3.20447	0.49019	3.62862

SO_2

Symbolic Z-matrix:

Charge = 0 Multiplicity = 1

S	0	0	0.37665
O	0	1.24592	-0.37665
O	0	-1.24592	-0.37665

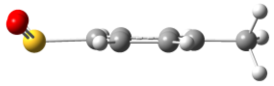

Ts radical

Symbolic Z-matrix:

Charge = 0 Multiplicity = 2

C	-0.36706	0.00144	-0.09208
C	0.3133	-1.21702	-0.07571
C	1.70109	-1.20172	-0.00901
C	2.4158	0.00678	0.02549
C	1.69814	1.21013	-0.0066
C	0.30746	1.22025	-0.07347
H	-0.23748	-2.16064	-0.09832
H	2.24538	-2.15103	0.01972
H	2.23861	2.16113	0.02358
H	-0.24588	2.16237	-0.09466
C	3.91295	-0.00261	0.0909
H	4.26578	-0.57484	0.96739
H	4.34234	-0.4875	-0.80455
H	4.3215	1.01838	0.15758
S	-2.14626	-0.00207	-0.23859
O	-2.65232	-1.29191	0.28258
O	-2.6577	1.28714	0.27862

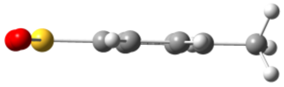

TS1

Symbolic Z-matrix:

Charge = 0 Multiplicity = 2

C	-0.29618	0.00902	0.08821
C	0.38828	-1.21889	0.06139
C	1.77309	-1.20203	0.0084
C	2.49772	0.00257	-0.0184
C	1.78022	1.20833	0.00758
C	0.3931	1.23194	0.06049
H	-0.16454	-2.16011	0.08139
H	2.31153	-2.15431	-0.01391
H	2.32326	2.15767	-0.01542
H	-0.15442	2.17622	0.07969
C	3.99538	-0.01055	-0.05411
H	4.40765	-0.32602	0.92168
H	4.37161	-0.72283	-0.80773
H	4.40249	0.98647	-0.28348
S	-2.18851	-0.00314	-0.04113
O	-2.74753	-0.08856	-1.40849
O	-2.95044	0.21631	1.20812

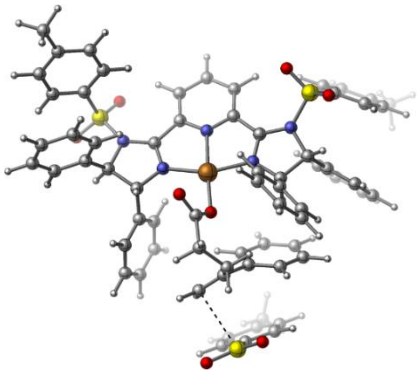

TS2

Symbolic Z-matrix:

Charge = 1 Multiplicity = 1

C	-2.22541	-3.51662	-1.4795
C	-1.78779	-2.41799	-0.7422
N	-0.48662	-2.13675	-0.68059
C	0.45975	-2.87087	-1.27025
C	0.09443	-4.00486	-1.99405
C	-1.26167	-4.30825	-2.09635
C	-2.5894	-1.3743	-0.03056

N	-3.93955	-1.30355	0.20328
C	-4.26072	0.04616	0.72238
C	-2.83862	0.64818	0.95479
N	-1.94877	-0.3151	0.34136
C	1.81061	-2.25319	-1.04784
N	1.80063	-1.08651	-0.48924
C	3.13775	-0.57496	-0.2853
C	4.06436	-1.74589	-0.72739
N	3.08022	-2.71798	-1.27982
Cu	0.04553	-0.37757	0.11774
C	-2.52063	0.91729	2.40712
C	-1.58773	0.167	3.12238
C	-1.30876	0.4705	4.45561
C	-1.97282	1.51699	5.09079
C	-2.9228	2.25969	4.38599
C	-3.19082	1.96401	3.05264
C	-5.13268	0.83088	-0.22383
C	-6.11485	1.67782	0.29667
C	-6.93406	2.41689	-0.55538
C	-6.78312	2.30684	-1.93716
C	-5.80181	1.46417	-2.46189
C	-4.97708	0.73406	-1.60967
C	3.38276	0.70996	-1.03611
C	4.2877	1.642	-0.51753
C	4.57964	2.80808	-1.222
C	3.96377	3.0575	-2.44842
C	3.05353	2.1364	-2.96528
C	2.76456	0.96618	-2.26315
C	4.88485	-2.33267	0.39035
C	4.26305	-2.8951	1.51176
C	5.027	-3.43865	2.53946
C	6.42155	-3.41581	2.4614
C	7.0457	-2.84629	1.35336
C	6.27872	-2.30988	0.31855
C	-6.31934	-2.22464	-0.66616
C	-7.44243	-1.49085	-0.28418
C	-8.37054	-1.13639	-1.25425
C	-8.19505	-1.50426	-2.59594
C	-7.06683	-2.26304	-2.94094
C	-6.12369	-2.62507	-1.98739
C	-9.17835	-1.06712	-3.63475
S	-5.08081	-2.54795	0.53719
O	-5.61199	-2.25724	1.85726
O	-4.40712	-3.80907	0.27881
C	4.00664	-5.26051	-0.88311
C	2.92457	-5.88434	-0.26204
C	3.17602	-6.79202	0.75914
C	4.48707	-7.09037	1.16051
C	5.55023	-6.46358	0.49559
C	5.3223	-5.54647	-0.52247
C	4.75132	-8.03407	2.29088
S	3.72654	-4.05663	-2.1336
O	5.00157	-3.58227	-2.63742
O	2.7048	-4.51477	-3.06099
H	-3.27892	-3.7643	-1.55778
H	0.8423	-4.6131	-2.49578
H	-1.57211	-5.17911	-2.67708
H	-4.77598	-0.06421	1.68474
H	-2.75215	1.59976	0.40584
H	3.25908	-0.38307	0.7912
H	4.7258	-1.43077	-1.54306
H	-1.05	-0.65044	2.63911
H	-0.5593	-0.11426	4.99401
H	-1.75294	1.75541	6.13438
H	-3.4512	3.08116	4.87644
H	-3.92353	2.55729	2.50031
H	-6.25084	1.7435	1.37976
H	-7.70303	3.07108	-0.13712
H	-7.43271	2.87541	-2.60735
H	-5.68162	1.37079	-3.54408
H	-4.22303	0.06367	-2.0295
H	4.76275	1.4494	0.44877
H	5.2742	3.53982	-0.80381
H	4.17969	3.98245	-2.98626
H	2.56195	2.32969	-3.92246
H	2.04653	0.25048	-2.67184
H	3.17198	-2.90985	1.58273
H	4.53356	-3.88224	3.40768
H	7.0214	-3.84276	3.26905
H	8.13634	-2.82381	1.28874
H	6.7657	-1.8752	-0.55836
H	-7.57801	-1.19432	0.75677
H	-9.24911	-0.55459	-0.96466
H	-6.92407	-2.57251	-3.97913
H	-5.24721	-3.2051	-2.28162
H	-10.2133	-1.19128	-3.28284
H	-9.0405	0.00468	-3.85718
H	-9.05473	-1.62437	-4.57348
H	1.89674	-5.66891	-0.5605

H	2.33657	-7.28386	1.2567
H	6.57869	-6.69016	0.78744
H	6.15022	-5.04626	-1.02598
H	5.02996	-7.47127	3.1979
H	3.86635	-8.6394	2.53084
H	5.58977	-8.70816	2.06076
C	0.23105	3.94648	0.6805
C	0.39723	3.74627	2.15598
C	1.45931	4.11407	-0.13055
C	1.40793	2.66326	2.53284
C	1.15761	1.30896	1.89641
O	0.47814	1.34554	0.80022
O	1.59701	0.27462	2.39474
C	-1.06317	4.04051	0.06188
C	-1.2419	3.85667	-1.33426
C	-2.49284	3.96059	-1.92598
C	-3.62146	4.26743	-1.15867
C	-3.47209	4.4551	0.21788
C	-2.22543	4.3347	0.81928
H	0.72154	4.69503	2.62216
H	-0.57075	3.48471	2.60522
H	2.35539	3.67631	0.33196
H	1.39976	3.73145	-1.15783
H	1.42864	2.52116	3.62302
H	2.43561	2.95819	2.25773
H	-0.38372	3.6133	-1.96297
H	-2.5926	3.8019	-3.00317
H	-4.60459	4.35355	-1.62623
H	-4.34312	4.70235	0.83088
H	-2.13859	4.50397	1.89321
C	0.82495	7.25585	-1.31368
C	-0.27224	7.8136	-0.65961
C	-1.34534	8.27055	-1.41456
C	-1.33567	8.18183	-2.81465
C	-0.20504	7.64386	-3.44419
C	0.87657	7.17709	-2.70296
H	-0.28152	7.87617	0.42994
H	-2.21487	8.69827	-0.90849
H	-0.17496	7.5863	-4.5355
H	1.75371	6.75165	-3.19561
C	-2.52344	8.62896	-3.60952
H	-2.88852	9.60986	-3.26896
H	-3.35565	7.91548	-3.48558
H	-2.29305	8.69488	-4.68204
S	2.13271	6.53284	-0.35669
O	1.79257	6.66486	1.06628
O	3.10768	5.93627	-1.27858

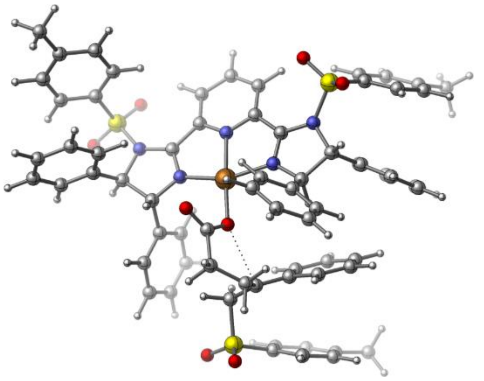

Int3

Symbolic Z-matrix:

Charge = 1 Multiplicity = 1

C	-2.22537	-3.51658	-1.47951
C	-1.78776	-2.41794	-0.74219
N	-0.48659	-2.13671	-0.68058
C	0.45978	-2.87082	-1.27025
C	0.09447	-4.00481	-1.99405
C	-1.26163	-4.30821	-2.09635
C	-2.58938	-1.37426	-0.03055
N	-3.93952	-1.30352	0.20329
C	-4.2607	0.04618	0.72242
C	-2.83859	0.6482	0.95482
N	-1.94874	-0.31507	0.34138
C	1.81064	-2.25314	-1.04784
N	1.80066	-1.08646	-0.48923
C	3.13777	-0.57491	-0.2853
C	4.06438	-1.74584	-0.72739
N	3.08024	-2.71792	-1.27984
Cu	0.04555	-0.37753	0.11777
C	-2.5206	0.9173	2.40715
C	-1.58771	0.167	3.12241
C	-1.30874	0.47049	4.45564
C	-1.9728	1.51697	5.09083
C	-2.92278	2.25968	4.38604
C	-3.19079	1.96402	3.05268
C	-5.13266	0.83092	-0.22378
C	-6.11483	1.67785	0.29674
C	-6.93404	2.41694	-0.55529
C	-6.7831	2.30691	-1.93707
C	-5.80179	1.46425	-2.46183
C	-4.97706	0.73413	-1.60962
C	3.38278	0.71002	-1.03611
C	4.28768	1.64208	-0.51751
C	4.5796	2.80816	-1.22198
C	3.96375	3.05757	-2.44841
C	3.05355	2.13644	-2.96529

C	2.7646	0.96621	-2.26316
C	4.88485	-2.33265	0.39036
C	4.26302	-2.8951	1.51174
C	5.02695	-3.43867	2.53945
C	6.4215	-3.41584	2.46142
C	7.04567	-2.84631	1.3534
C	6.27872	-2.30987	0.31858
C	-6.31931	-2.22462	-0.66615
C	-7.4424	-1.49083	-0.28417
C	-8.37052	-1.13638	-1.25424
C	-8.19502	-1.50425	-2.59593
C	-7.06681	-2.26303	-2.94093
C	-6.12366	-2.62506	-1.98738
C	-9.17833	-1.06713	-3.63474
S	-5.08078	-2.54793	0.5372
O	-5.61196	-2.25722	1.85727
O	-4.40708	-3.80905	0.27882
C	4.00661	-5.26047	-0.88318
C	2.92451	-5.8843	-0.26217
C	3.17591	-6.79199	0.75901
C	4.48694	-7.09036	1.16044
C	5.55013	-6.46356	0.49557
C	5.32225	-5.54643	-0.52249
C	4.75114	-8.03407	2.2908
S	3.72655	-4.05656	-2.13365
O	5.0016	-3.58221	-2.63744
O	2.70481	-4.51466	-3.06106
H	-3.27889	-3.76425	-1.55779
H	0.84233	-4.61304	-2.49579
H	-1.57208	-5.17906	-2.67709
H	-4.77594	-0.06421	1.68477
H	-2.75213	1.59979	0.40587
H	3.25912	-0.38303	0.7912
H	4.72584	-1.43072	-1.54305
H	-1.04998	-0.65044	2.63913
H	-0.55929	-0.11428	4.99404
H	-1.75293	1.75538	6.13443
H	-3.45118	3.08115	4.8765
H	-3.9235	2.55731	2.50036
H	-6.25082	1.74352	1.37983
H	-7.703	3.07112	-0.13702
H	-7.43269	2.8755	-2.60726
H	-5.6816	1.37089	-3.54402
H	-4.22302	0.06374	-2.02947

H	4.76271	1.4495	0.4488
H	5.27413	3.53993	-0.80377
H	4.17965	3.98252	-2.98625
H	2.56198	2.3297	-3.92248
H	2.0466	0.25049	-2.67187
H	3.17195	-2.90984	1.58269
H	4.53348	-3.88227	3.40765
H	7.02132	-3.8428	3.26907
H	8.13631	-2.82383	1.2888
H	6.76572	-1.87517	-0.55831
H	-7.57798	-1.1943	0.75678
H	-9.24909	-0.55458	-0.96465
H	-6.92404	-2.57249	-3.97911
H	-5.24718	-3.20507	-2.28162
H	-10.2133	-1.19136	-3.28284
H	-9.04054	0.00469	-3.85713
H	-9.05468	-1.62434	-4.57348
H	1.89669	-5.66886	-0.56068
H	2.33643	-7.28384	1.25653
H	6.57858	-6.69015	0.78746
H	6.1502	-5.04622	-1.02596
H	5.02969	-7.47127	3.19786
H	3.86617	-8.63944	2.53068
H	5.58963	-8.70812	2.06073
C	0.2311	3.94648	0.68055
C	0.39724	3.74626	2.15603
C	1.45938	4.11412	-0.13046
C	1.40794	2.66327	2.5329
C	1.15764	1.30897	1.89646
O	0.47817	1.34556	0.80027
O	1.597	0.27462	2.39481
C	-1.06311	4.04047	0.06188
C	-1.24178	3.85659	-1.33426
C	-2.49271	3.96046	-1.92602
C	-3.62136	4.26729	-1.15876
C	-3.47204	4.45499	0.2178
C	-2.2254	4.33464	0.81923
H	0.72151	4.69502	2.62224
H	-0.57075	3.48467	2.60524
H	2.35546	3.67643	0.33209
H	1.39988	3.73147	-1.15774
H	1.42865	2.52116	3.62308
H	2.43563	2.95821	2.25779
H	-0.38358	3.61324	-1.96294
H	-2.59243	3.80175	-3.00321
H	-4.60448	4.35337	-1.62635
H	-4.3431	4.70223	0.83076
H	-2.1386	4.50394	1.89317

C	0.63791	6.58418	-1.25088
C	-0.45933	7.14189	-0.59687
C	-1.53243	7.59875	-1.35186
C	-1.52272	7.50999	-2.75195
C	-0.39205	6.97206	-3.38144
C	0.68957	6.50538	-2.64016
H	-0.46864	7.20449	0.49268
H	-2.40201	8.02644	-0.84582
H	-0.36193	6.91448	-4.47274
H	1.56675	6.07997	-3.13277
C	-2.71049	7.95703	-3.54687
H	-3.07566	8.93791	-3.20632
H	-3.54265	7.24349	-3.42296
H	-2.48006	8.02297	-4.61937
S	1.94569	5.86128	-0.29384
O	1.92205	6.45862	1.04802
O	3.18326	5.91619	-1.08235

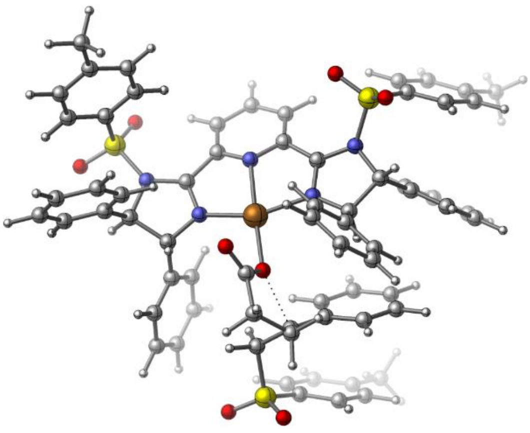

TS3-*R*

Symbolic Z-matrix:

Charge = 1 Multiplicity = 1

C	-2.12329	-3.0428	-1.86081
C	-1.68386	-2.11709	-0.91591
N	-0.3855	-1.86888	-0.76731
C	0.55938	-2.46891	-1.49399
C	0.19919	-3.42721	-2.44136
C	-1.15707	-3.70227	-2.61356
C	-2.50348	-1.18922	-0.08557
N	-3.85421	-1.223	0.16864
C	-4.24547	0.06191	0.7826
C	-2.85386	0.71773	1.06709
N	-1.91538	-0.13176	0.3629
C	1.9027	-1.87158	-1.19093
N	1.87938	-0.73405	-0.57772
C	3.21503	-0.24128	-0.32668
C	4.14656	-1.41532	-0.75075
N	3.17953	-2.32898	-1.4181
Cu	0.11352	-0.11647	0.17216
C	-2.52976	0.85804	2.53445
C	-1.71312	-0.05602	3.20159
C	-1.43785	0.10531	4.55985
C	-1.98681	1.17439	5.26441
C	-2.81737	2.08379	4.60548
C	-3.08391	1.92766	3.24804
C	-5.14587	0.87045	-0.11659
C	-6.06862	1.74756	0.45949
C	-6.91423	2.5114	-0.34308
C	-6.85131	2.39657	-1.73187
C	-5.93041	1.52323	-2.31175
C	-5.07865	0.76748	-1.50875
C	3.48552	1.05274	-1.05561
C	4.44112	1.93783	-0.54416
C	4.74081	3.11893	-1.21809
C	4.08239	3.43231	-2.40747
C	3.12363	2.55863	-2.91818
C	2.82698	1.37197	-2.24614
C	4.85793	-2.07875	0.40071
C	4.13875	-2.635	1.46634
C	4.80753	-3.24623	2.52225
C	6.20298	-3.30318	2.52884
C	6.92424	-2.74452	1.47553
C	6.25271	-2.13733	0.41401
C	-6.21343	-2.27585	-0.56612
C	-7.29224	-1.54232	-0.07059
C	-8.30881	-1.17755	-0.94312
C	-8.26621	-1.53675	-2.29834
C	-7.18484	-2.30393	-2.75515
C	-6.15388	-2.67505	-1.90012
C	-9.34272	-1.08573	-3.23371
S	-4.84786	-2.58665	0.49416
O	-5.27844	-2.45761	1.87598
O	-4.09636	-3.75158	0.05852
C	3.8822	-4.95583	-1.02299
C	2.69934	-5.56177	-0.59926
C	2.76634	-6.5328	0.39242
C	3.99371	-6.91351	0.95571
C	5.16462	-6.30217	0.48545
C	5.12049	-5.32232	-0.49827
C	4.05713	-7.93237	2.04962
S	3.8269	-3.68346	-2.23677
O	5.17921	-3.27992	-2.57426
O	2.88963	-4.04148	-3.28905
H	-3.17987	-3.23812	-2.01533

H	0.95188	-3.91848	-3.0533
H	-1.4633	-4.43043	-3.36705
H	-4.7594	-0.13941	1.7316
H	-2.82627	1.71895	0.61013
H	3.31545	-0.06334	0.75453
H	4.87985	-1.08378	-1.4952
H	-1.26755	-0.89026	2.65665
H	-0.78185	-0.60742	5.06517
H	-1.76856	1.30165	6.3277
H	-3.25227	2.92478	5.15152
H	-3.72233	2.64694	2.72947
H	-6.13636	1.82197	1.54842
H	-7.63603	3.18913	0.11953
H	-7.52267	2.98452	-2.36272
H	-5.8795	1.42359	-3.3989
H	-4.37644	0.07068	-1.97157
H	4.94902	1.69783	0.39451
H	5.47627	3.81179	-0.80412
H	4.31089	4.36555	-2.92638
H	2.59996	2.80092	-3.84678
H	2.06997	0.69436	-2.64828
H	3.04628	-2.59503	1.47608
H	4.23695	-3.68189	3.34606
H	6.72712	-3.78446	3.35833
H	8.01632	-2.78523	1.47497
H	6.81571	-1.71242	-0.42087
H	-7.32896	-1.25455	0.9812
H	-9.152	-0.59351	-0.5661
H	-7.14977	-2.61455	-3.80221
H	-5.32006	-3.27451	-2.26988
H	-10.3405	-1.21993	-2.78964
H	-9.22846	-0.00991	-3.44945
H	-9.30608	-1.62744	-4.18877
H	1.73553	-5.28642	-1.03224
H	1.84573	-7.01192	0.73444
H	6.13113	-6.59332	0.90438
H	6.03115	-4.83728	-0.85143
H	4.23269	-7.43856	3.0203
H	3.12151	-8.50309	2.12832
H	4.88794	-8.6364	1.89261
C	0.2924	3.70665	0.94169
C	0.34634	3.71104	2.43834
C	1.56496	3.96963	0.22376
C	1.32548	2.6494	2.91759
C	1.11129	1.38649	2.11985
O	0.518	1.60387	0.98469
O	1.47343	0.27521	2.49061
C	-0.95801	3.74978	0.21741

C	-1.03189	3.34687	-1.13741
C	-2.23341	3.37751	-1.83175
C	-3.40197	3.82878	-1.20987
C	-3.34961	4.2354	0.12489
C	-2.15321	4.18499	0.83246
H	0.64438	4.7079	2.80773
H	-0.64763	3.48861	2.84915
H	2.45647	3.69753	0.80639
H	1.65166	3.49581	-0.7632
H	1.22176	2.43281	3.99009
H	2.37658	2.95388	2.774
H	-0.13497	2.98673	-1.6451
H	-2.26292	3.04773	-2.87362
H	-4.34564	3.85608	-1.75909
H	-4.25452	4.59635	0.6206
H	-2.13371	4.5224	1.86976
C	0.66455	6.17855	-1.32037
C	-0.54642	6.76157	-0.9528
C	-1.49891	7.0086	-1.93392
C	-1.25827	6.681	-3.27666
C	-0.01963	6.12057	-3.61793
C	0.94644	5.86831	-2.64868
H	-0.73573	7.00763	0.0934
H	-2.45482	7.45811	-1.65234
H	0.19055	5.87844	-4.6631
H	1.91017	5.43074	-2.91821
C	-2.3176	6.90483	-4.31113
H	-2.75813	7.90964	-4.22262
H	-3.13959	6.18104	-4.17918
H	-1.92279	6.78532	-5.32964
S	1.84456	5.74725	-0.0679
O	1.49894	6.46772	1.16452
O	3.19145	5.89048	-0.63105

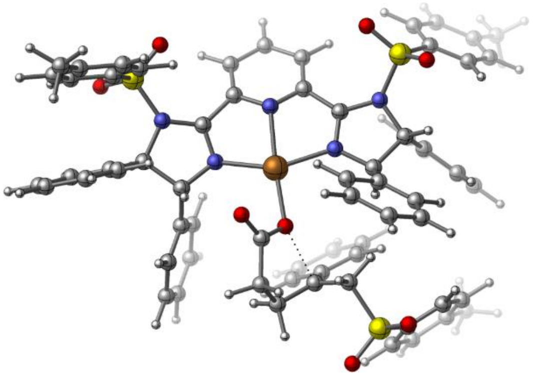

TS3-S

Symbolic Z-matrix:

Charge = 1 Multiplicity = 1

C	-0.90307	-3.13612	-1.20545	S	-3.31651	-3.42688	1.51714
C	-0.59781	-2.16916	-0.25094	O	-3.65958	-3.33298	2.92705
N	0.61328	-1.61287	-0.19218	O	-2.2826	-4.34052	1.06052
C	1.56558	-1.92293	-1.07256	C	6.05035	-2.69815	-0.32895
C	1.34116	-2.87495	-2.06885	C	5.55141	-3.0223	0.93276
C	0.09163	-3.48897	-2.11495	C	6.45022	-3.21474	1.97398
C	-1.55282	-1.46819	0.64136	C	7.83402	-3.10205	1.76966
N	-2.81697	-1.86496	1.02955	C	8.29908	-2.80253	0.48177
C	-3.50815	-0.69726	1.6031	C	7.41773	-2.59806	-0.57292
C	-2.32707	0.30651	1.77387	C	8.79497	-3.26505	2.90532
N	-1.26062	-0.25942	0.97829	S	4.92674	-2.48413	-1.66566
C	2.69027	-0.94854	-0.99062	O	5.66538	-2.14557	-2.87078
N	2.35652	0.26075	-0.67787	O	3.98588	-3.5943	-1.6488
C	3.53782	1.1076	-0.66693	H	-1.89509	-3.57655	-1.26436
C	4.63692	0.25942	-1.35725	H	2.10795	-3.11545	-2.80066
N	4.03286	-1.09828	-1.25596	H	-0.12038	-4.22487	-2.89304
Cu	0.65616	0.39533	0.49275	H	-3.9253	-0.9656	2.58261
C	-1.9188	0.60296	3.19923	H	-2.61504	1.26189	1.31072
C	-0.60974	0.4217	3.64702	H	3.80037	1.27814	0.38866
C	-0.24437	0.81101	4.9361	H	4.70147	0.5097	-2.428
C	-1.18559	1.38043	5.7913	H	0.13868	-0.00944	2.97967
C	-2.50089	1.55021	5.35529	H	0.78805	0.67269	5.26685
C	-2.86363	1.16283	4.06835	H	-0.89722	1.6922	6.79817
C	-4.59825	-0.16963	0.70015	H	-3.24503	1.99759	6.01838
C	-5.61155	0.62102	1.25209	H	-3.89283	1.31173	3.72802
C	-6.60389	1.16732	0.44001	H	-5.63065	0.80088	2.33089
C	-6.59647	0.92382	-0.93426	H	-7.39577	1.77536	0.88458
C	-5.59104	0.13179	-1.48864	H	-7.37861	1.34531	-1.57065
C	-4.59614	-0.41032	-0.67618	H	-5.5819	-0.07058	-2.56254
C	3.31725	2.44551	-1.3178	H	-3.82222	-1.043	-1.11582
C	3.93831	3.58235	-0.79216	H	4.54832	3.49187	0.11057
C	3.78166	4.82287	-1.41067	H	4.27021	5.7051	-0.98945
C	3.00321	4.9359	-2.56257	H	2.88044	5.907	-3.0489
C	2.37972	3.80395	-3.09055	H	1.76701	3.88646	-3.9918
C	2.53327	2.566	-2.47004	H	2.03397	1.68387	-2.87893
C	5.99713	0.42231	-0.7357	H	5.33939	0.05792	1.29614
C	6.18294	0.30721	0.64798	H	7.57814	0.39476	2.28563
C	7.44358	0.49263	1.20565	H	9.52259	0.95074	0.82829
C	8.53309	0.80259	0.38839	H	9.20287	1.16458	-1.63309
C	8.35499	0.92097	-0.98815	H	6.94892	0.81724	-2.62694
C	7.09105	0.72964	-1.54691	H	-6.02037	-2.69129	2.10271
C	-4.79551	-3.63335	0.58629	H	-8.07598	-2.79941	0.7214
C	-5.99091	-3.13131	1.10461	H	-5.87473	-4.76103	-2.41926
C	-7.13832	-3.19858	0.32652	H	-3.81137	-4.65673	-1.0489
C	-7.11379	-3.76697	-0.95638	H	-9.16914	-4.32906	-1.25492
C	-5.90491	-4.29146	-1.43297	H	-8.71575	-2.78696	-1.99615
C	-4.74197	-4.22907	-0.67206	H	-8.18836	-4.31688	-2.74551
C	-8.35762	-3.80796	-1.78693	H	4.47738	-3.11978	1.10596

H	6.07183	-3.46133	2.96922
H	9.37401	-2.71937	0.30247
H	7.78017	-2.35207	-1.57182
H	9.12269	-2.27701	3.27129
H	8.33788	-3.79786	3.75073
H	9.69872	-3.80823	2.59183
C	-0.48768	4.17096	1.22465
C	0.85386	4.82433	1.31068
C	-1.19382	3.99117	2.52031
C	1.78712	4.01847	2.20363
C	1.66418	2.54376	1.87309
O	0.57633	2.24463	1.23154
O	2.50814	1.71531	2.18995
C	-1.21491	4.02028	-0.01516
C	-2.50771	3.44389	-0.03894
C	-3.18848	3.23889	-1.2293
C	-2.60841	3.62102	-2.44106
C	-1.33914	4.20512	-2.44387
C	-0.64843	4.39595	-1.25518
H	0.71023	5.84122	1.71729
H	1.30408	4.92375	0.31679
H	-0.5055	3.80553	3.35611
H	-1.94448	3.19382	2.52807
H	2.83441	4.33021	2.08968
H	1.55254	4.13926	3.27468
H	-3.00044	3.1573	0.8885
H	-4.1801	2.78007	-1.21115
H	-3.1445	3.4657	-3.38051
H	-0.87895	4.50965	-3.38676
H	0.34738	4.83702	-1.2948
C	-3.49434	5.62188	2.08683
C	-3.44423	6.41325	0.94244
C	-4.54175	6.42394	0.08657
C	-5.67983	5.65316	0.35741
C	-5.70976	4.89038	1.53604
C	-4.6264	4.87048	2.4047
H	-2.55277	7.00348	0.72201
H	-4.50984	7.03585	-0.81836
H	-6.60195	4.30593	1.77585
H	-4.65098	4.28059	3.32356
C	-6.83405	5.61637	-0.59497
H	-6.78751	6.43967	-1.321
H	-6.83177	4.66901	-1.16062
H	-7.79528	5.67094	-0.06214
S	-2.06509	5.48035	3.1215
O	-1.19941	6.64172	2.89148
O	-2.50234	5.15271	4.48028

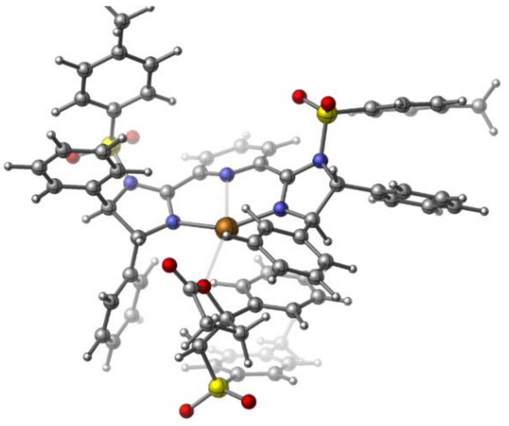

int4R

Symbolic Z-matrix:

Charge = 1 Multiplicity = 1

C	-0.83144	-2.58279	-2.28747
C	-0.69483	-1.93383	-1.06265
N	0.44045	-1.30913	-0.76156
C	1.51955	-1.30194	-1.54861
C	1.46767	-1.96167	-2.77433
C	0.27149	-2.58451	-3.13652
C	-1.70657	-1.6986	0.00075
N	-2.86616	-2.36616	0.2526
C	-3.587	-1.66578	1.33635
C	-2.57119	-0.54752	1.74722
N	-1.4982	-0.68127	0.77751
C	2.60467	-0.50291	-0.89466
N	2.22377	0.25964	0.08799
C	3.36405	0.88367	0.74513
C	4.59251	0.19819	0.075
N	3.95615	-0.5261	-1.05439
Cu	0.32317	0.1106	0.61709
C	-2.06757	-0.66308	3.16083
C	-1.03587	-1.55098	3.48117
C	-0.59816	-1.672	4.79835
C	-1.19748	-0.91762	5.80675
C	-2.23793	-0.04246	5.49474
C	-2.66841	0.08747	4.17548
C	-4.9397	-1.16569	0.90389
C	-5.93977	-1.01292	1.86882
C	-7.20434	-0.55425	1.505
C	-7.48263	-0.25684	0.1707
C	-6.48876	-0.41399	-0.79541
C	-5.22097	-0.86217	-0.43026
C	3.361	2.38515	0.61551
C	3.49068	3.1804	1.75657
C	3.55526	4.56935	1.64471

C	3.49388	5.17324	0.39033	H	3.54101	6.26044	0.30192
C	3.36732	4.38236	-0.75377	H	3.33511	4.84983	-1.74075
C	3.29453	2.99561	-0.64325	H	3.21144	2.38501	-1.54724
C	5.35717	-0.73563	0.97571	H	3.76001	-2.19114	1.15135
C	4.77817	-1.92085	1.44576	H	5.04557	-3.70267	2.62791
C	5.50089	-2.77496	2.27291	H	7.3772	-3.1245	3.28767
C	6.80848	-2.45098	2.64182	H	8.41073	-1.01304	2.46511
C	7.38699	-1.26961	2.18214	H	7.12183	0.50267	0.97296
C	6.66399	-0.41617	1.34867	H	-5.74904	-4.34902	1.18929
C	-4.66735	-4.1506	-0.67478	H	-7.96599	-4.21892	0.07991
C	-5.82059	-4.23574	0.10655	H	-6.03683	-3.8651	-3.75699
C	-7.05591	-4.16292	-0.52224	H	-3.81595	-3.99313	-2.66386
C	-7.15474	-4.00691	-1.91284	H	-9.21945	-4.57864	-2.1386
C	-5.97443	-3.95887	-2.67013	H	-8.89419	-2.85615	-2.38849
C	-4.72693	-4.02733	-2.06266	H	-8.43825	-4.02676	-3.64914
C	-8.49386	-3.86926	-2.56335	H	3.68537	-3.66629	-2.30798
S	-3.11344	-4.0835	0.12809	H	4.46192	-5.83898	-1.42562
O	-3.24498	-4.55559	1.495	H	8.24178	-4.07676	-0.34172
O	-2.04502	-4.5707	-0.72661	H	7.46963	-1.88018	-1.20169
C	5.53364	-2.64794	-1.78343	H	7.11892	-6.3256	0.80583
C	4.6799	-3.74837	-1.86536	H	6.10152	-7.20073	-0.37048
C	5.12181	-4.96969	-1.37112	H	7.7801	-6.75463	-0.77835
C	6.39928	-5.10803	-0.80878	C	-0.76791	2.98171	1.28121
C	7.23917	-3.98512	-0.76678	C	-1.56582	3.15671	2.57225
C	6.81761	-2.75313	-1.24684	C	0.147	4.17511	1.00673
C	6.8662	-6.41962	-0.26257	C	-0.5376	2.86469	3.65622
S	4.98605	-1.07246	-2.326	C	0.37639	1.86056	3.0284
O	6.09294	-0.13675	-2.303	O	0.13916	1.8653	1.66153
O	4.14585	-1.19499	-3.50523	O	1.19623	1.12491	3.48018
H	-1.76593	-3.05622	-2.57798	C	-1.49802	2.53576	0.03786
H	2.32263	-1.9575	-3.44643	C	-0.7461	2.20438	-1.10198
H	0.20163	-3.07603	-4.10877	C	-1.37857	1.76819	-2.26283
H	-3.70937	-2.36312	2.17606	C	-2.77069	1.68515	-2.30748
H	-3.04164	0.43756	1.61021	C	-3.52247	2.04632	-1.19126
H	3.32891	0.62268	1.81225	C	-2.8905	2.46678	-0.02122
H	5.2723	0.95461	-0.33444	H	-2.01099	4.15639	2.62828
H	-0.57215	-2.15817	2.69944	H	-2.36561	2.40477	2.61648
H	0.21485	-2.36084	5.03959	H	0.64513	4.52782	1.92152
H	-0.85302	-1.0126	6.83933	H	0.92951	3.93711	0.27324
H	-2.71319	0.54754	6.28222	H	-0.95461	2.44971	4.58278
H	-3.4803	0.77744	3.92825	H	0.06569	3.74534	3.93334
H	-5.73025	-1.27144	2.91079	H	0.34444	2.29743	-1.09286
H	-7.98116	-0.44322	2.2655	H	-0.78234	1.50995	-3.14138
H	-8.47795	0.09008	-0.11772	H	-3.27157	1.35632	-3.22138
H	-6.70426	-0.19322	-1.84392	H	-4.61266	2.00285	-1.22679
H	-4.45463	-1.00318	-1.19486	H	-3.49565	2.75688	0.83909
H	3.54528	2.70508	2.73948	C	-0.93369	5.46007	-1.3174
H	3.65503	5.18296	2.54332	C	-2.23319	5.28216	-1.78655

C	-2.43372	5.10469	-3.15
C	-1.35688	5.10226	-4.04907
C	-0.06418	5.3068	-3.54604
C	0.1552	5.49749	-2.18601
H	-3.0692	5.28085	-1.08556
H	-3.44954	4.96135	-3.52786
H	0.78356	5.32875	-4.23601
H	1.1611	5.67593	-1.80189
C	-1.59524	4.88679	-5.51159
H	-2.26158	5.6634	-5.91974
H	-2.08957	3.91779	-5.68835
H	-0.65735	4.90488	-6.08348
S	-0.65349	5.68456	0.41668
O	-1.95548	5.81824	1.08136
O	0.35896	6.72877	0.58433

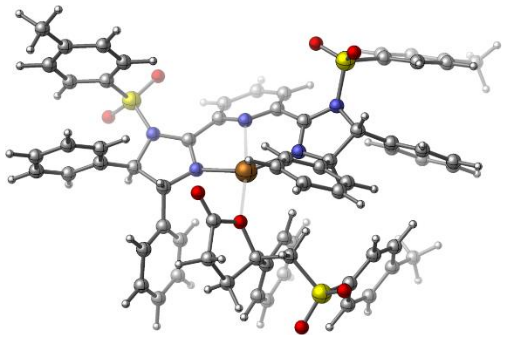

Int4*S*

Symbolic Z-matrix:

Charge = 1 Multiplicity = 1

C	-3.19816	-1.84154	2.88162
C	-2.42465	-1.46824	1.78337
N	-2.80828	-0.49607	0.96133
C	-3.9005	0.2305	1.20093
C	-4.7271	-0.05674	2.28812
C	-4.37082	-1.12587	3.11227
C	-1.00838	-1.83503	1.52159
N	-0.38024	-3.04686	1.70418
C	1.02515	-2.91036	1.25651
C	1.14143	-1.36645	1.05682
N	-0.22004	-0.87482	1.16075
C	-3.86769	1.4708	0.36809
N	-2.68988	1.96949	0.15238
C	-2.80358	3.16542	-0.66363
C	-4.31432	3.51634	-0.63726
N	-4.88323	2.21874	-0.18413
Cu	-1.11602	0.77403	0.44041
C	1.81039	-0.96467	-0.23053
C	1.05943	-0.70437	-1.38232
C	1.6956	-0.36085	-2.57482
C	3.08694	-0.2855	-2.62967
C	3.84058	-0.54817	-1.48495
C	3.20524	-0.88023	-0.28988
C	2.02348	-3.49061	2.21955
C	3.12287	-4.19871	1.72878
C	4.06381	-4.73685	2.60686
C	3.90616	-4.57693	3.98249
C	2.80981	-3.86754	4.47757
C	1.8765	-3.32185	3.60066
C	-1.9178	4.29296	-0.2098
C	-1.16833	5.00535	-1.15147
C	-0.37924	6.08369	-0.75468
C	-0.32674	6.45478	0.58897
C	-1.06908	5.74493	1.53305
C	-1.86442	4.67126	1.13562
C	-4.83153	4.02875	-1.95301
C	-4.64948	3.30066	-3.13572
C	-5.10492	3.80484	-4.34918
C	-5.73963	5.04844	-4.39722
C	-5.9206	5.77954	-3.22551
C	-5.47048	5.26871	-2.00753
C	-0.5197	-5.50514	2.81405
C	0.54635	-6.37992	2.60805
C	1.07662	-7.05418	3.70022
C	0.55871	-6.86722	4.99012
C	-0.53132	-6.00008	5.15659
C	-1.07737	-5.3155	4.07758
C	1.18011	-7.5594	6.16197
S	-1.13119	-4.576	1.4543
O	-0.58023	-5.1193	0.22192
O	-2.56043	-4.34877	1.59036
C	-7.08156	1.81966	-1.76416
C	-6.71315	0.66732	-2.45835
C	-7.14212	0.51675	-3.77084
C	-7.9409	1.4904	-4.39023
C	-8.314	2.62163	-3.65122
C	-7.88997	2.7967	-2.33967
C	-8.36194	1.34429	-5.81879
S	-6.57132	2.01106	-0.09204
O	-7.10151	3.25801	0.43407
O	-6.78987	0.74931	0.59789
H	-2.87913	-2.63185	3.55925
H	-5.60963	0.54052	2.5036
H	-4.99373	-1.36964	3.97535
H	1.12235	-3.41425	0.28315
H	1.71723	-0.94432	1.89733
H	-2.5178	2.89307	-1.69403

H	-4.5195	4.26103	0.1479
H	-0.0327	-0.76861	-1.34445
H	1.10015	-0.15389	-3.46773
H	3.58568	-0.01908	-3.56489
H	4.93111	-0.48582	-1.52065
H	3.79586	-1.07397	0.60984
H	3.23431	-4.34046	0.65018
H	4.91805	-5.29323	2.21319
H	4.63608	-5.00794	4.67235
H	2.67861	-3.74421	5.55555
H	1.01132	-2.78185	3.99316
H	-1.20309	4.70955	-2.20371
H	0.20343	6.63289	-1.49852
H	0.2964	7.29657	0.90138
H	-1.02907	6.02954	2.58763
H	-2.4436	4.11408	1.87621
H	-4.16058	2.32341	-3.10749
H	-4.96503	3.22528	-5.26496
H	-6.09463	5.44487	-5.35181
H	-6.41616	6.75295	-3.25599
H	-5.61807	5.83706	-1.08586
H	0.95657	-6.51995	1.60711
H	1.91661	-7.7369	3.55006
H	-0.95942	-5.85971	6.15229
H	-1.92711	-4.64511	4.22046
H	1.41278	-8.60942	5.9299
H	2.13222	-7.06958	6.42805
H	0.5286	-7.52985	7.04613
H	-6.09898	-0.10114	-1.98364
H	-6.85607	-0.37852	-4.32853
H	-8.94335	3.38529	-4.11507
H	-8.16909	3.68297	-1.76839
H	-7.71342	1.95457	-6.4704
H	-8.28991	0.30216	-6.15973
H	-9.39302	1.69582	-5.97124
C	1.43292	2.44812	0.18254
C	1.43423	3.59307	-0.8391
C	2.73425	1.63487	0.13844
C	1.02195	2.89454	-2.12211
C	0.18861	1.73565	-1.63649
O	0.42507	1.53952	-0.32062
O	-0.57547	1.04505	-2.24737
C	1.05274	2.82689	1.59777
C	0.6656	1.8205	2.49126
C	0.3453	2.13071	3.80974
C	0.40623	3.45303	4.25225
C	0.79323	4.45807	3.36915
C	1.11872	4.14683	2.0489
H	2.4091	4.09359	-0.87726
H	0.6719	4.33253	-0.55746
H	3.06602	1.46942	-0.89906
H	2.59173	0.64996	0.60695
H	0.44465	3.51414	-2.82046
H	1.87748	2.48088	-2.68226
H	0.60986	0.78454	2.14894
H	0.04569	1.335	4.4963
H	0.15516	3.69726	5.28752
H	0.85016	5.49536	3.70848
H	1.4391	4.94392	1.37649
C	4.05808	2.04887	2.64143
C	3.9733	3.13318	3.50788
C	3.89177	2.89641	4.87692
C	3.89017	1.59289	5.38857
C	4.00297	0.52098	4.48944
C	4.09866	0.73878	3.12085
H	3.96724	4.14777	3.10704
H	3.81938	3.74442	5.56299
H	4.02368	-0.50268	4.87263
H	4.20463	-0.10358	2.43316
C	3.73802	1.34687	6.8578
H	4.14468	2.17838	7.45124
H	2.6702	1.24974	7.11914
H	4.23566	0.41637	7.16697
S	4.20593	2.33891	0.8969
O	4.22415	3.78903	0.66558
O	5.31339	1.51538	0.40118

Cu-product

Symbolic Z-matrix:

Charge = 1 Multiplicity = 1

C	-3.00228	-1.83341	2.82954
C	-2.23768	-1.38808	1.753
N	-2.60656	-0.3441	1.00792
C	-3.71079	0.35097	1.30845
C	-4.53013	-0.01801	2.3757
C	-4.16707	-1.13291	3.1311
C	-0.85732	-1.80667	1.43827
N	-0.29467	-3.06067	1.58379
C	1.1419	-2.9653	1.26184
C	1.29341	-1.45151	0.88988
N	-0.03427	-0.87976	1.08338
C	-3.73953	1.62105	0.53845
N	-2.59	2.17899	0.35432
C	-2.75634	3.3538	-0.48264
C	-4.28398	3.63024	-0.48727

N	-4.79355	2.30992	-0.03791
Cu	-0.97736	0.75838	0.17177
C	1.78054	-1.19533	-0.51391
C	1.04209	-1.66957	-1.60494
C	1.45828	-1.4056	-2.907
C	2.62035	-0.66315	-3.13243
C	3.36446	-0.19444	-2.04979
C	2.94425	-0.46015	-0.74522
C	2.02677	-3.42918	2.38888
C	3.25373	-4.02943	2.09129
C	4.08971	-4.47326	3.11541
C	3.70061	-4.32757	4.4474
C	2.47742	-3.72591	4.74955
C	1.64665	-3.27512	3.7255
C	-1.93176	4.53522	-0.05252
C	-1.48172	5.45058	-1.00989
C	-0.75165	6.57487	-0.62495
C	-0.45951	6.79016	0.7226
C	-0.90236	5.87771	1.68165
C	-1.63747	4.75698	1.29629
C	-4.80273	4.10318	-1.81794
C	-4.55932	3.37523	-2.9902
C	-5.01775	3.8426	-4.21793
C	-5.72074	5.04833	-4.29078
C	-5.96651	5.7777	-3.12911
C	-5.51053	5.30416	-1.89767
C	-0.58014	-5.54529	2.55858
C	0.56342	-6.33262	2.4225
C	1.03068	-7.02962	3.53015
C	0.3731	-6.95358	4.76675
C	-0.79325	-6.17991	4.85978
C	-1.27553	-5.47157	3.76476
C	0.92897	-7.66052	5.96443
S	-1.08742	-4.53474	1.21246
O	-0.49296	-5.0473	-0.01442
O	-2.51266	-4.25636	1.28112
C	-6.925	1.75577	-1.65915
C	-6.4668	0.61694	-2.32214
C	-6.84978	0.41501	-3.64264
C	-7.69106	1.32428	-4.30238
C	-8.15532	2.44189	-3.59534
C	-7.77886	2.66696	-2.27591
C	-8.06102	1.12693	-5.74076
S	-6.46321	2.01642	0.01966
O	-7.07649	3.24552	0.49676
O	-6.63719	0.76288	0.73742
H	-2.67234	-2.67159	3.44444
H	-5.41684	0.55982	2.62842

H	-4.77948	-1.43118	3.98611
H	1.3411	-3.58151	0.37274
H	1.98215	-0.96783	1.60071
H	-2.44227	3.07057	-1.50258
H	-4.54024	4.37123	0.28787
H	0.13083	-2.25219	-1.43227
H	0.87345	-1.78077	-3.75237
H	2.94852	-0.45553	-4.15571
H	4.27947	0.38143	-2.22009
H	3.52445	-0.08672	0.10473
H	3.54858	-4.16421	1.04543
H	5.04484	-4.94799	2.87075
H	4.34954	-4.68673	5.25204
H	2.16389	-3.61384	5.79202
H	0.68015	-2.82332	3.9669
H	-1.70579	5.27768	-2.06776
H	-0.40491	7.28461	-1.38238
H	0.11692	7.66955	1.02599
H	-0.6726	6.03952	2.73942
H	-1.97982	4.03847	2.04716
H	-4.01903	2.42463	-2.94295
H	-4.82767	3.2624	-5.12601
H	-6.07993	5.41596	-5.25687
H	-6.51787	6.72149	-3.17802
H	-5.70897	5.87231	-0.98392
H	1.0853	-6.39152	1.46495
H	1.93285	-7.64126	3.43499
H	-1.33321	-6.13012	5.81007
H	-2.18727	-4.87412	3.84604
H	1.31232	-8.66038	5.69949
H	1.77813	-7.08704	6.38198
H	0.17468	-7.77047	6.76025
H	-5.81738	-0.10398	-1.81786
H	-6.49257	-0.472	-4.17455
H	-8.82056	3.15591	-4.09026
H	-8.13181	3.54428	-1.73036
H	-7.42517	1.75854	-6.38977
H	-7.92285	0.07999	-6.05631
H	-9.10787	1.41819	-5.93022

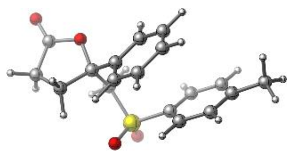

***R*-product**

Symbolic Z-matrix:

Charge = 0 Multiplicity = 1

C	2.04846	2.77065	1.97148
C	1.61314	3.49507	3.24561
C	3.2686	1.87678	2.2089
C	0.87748	2.40925	4.01356
C	0.32553	1.52277	2.9349
O	0.94011	1.83122	1.75697
O	-0.51213	0.66993	2.98589
C	2.19975	3.60482	0.72405
C	2.34021	2.95729	-0.51169
C	2.52215	3.69092	-1.67951
C	2.59144	5.08347	-1.62282
C	2.47878	5.7311	-0.39616
C	2.27822	4.99657	0.77257
H	2.47377	3.92418	3.77154
H	0.91666	4.30539	2.98668
H	3.2077	1.35658	3.17607
H	3.35504	1.10943	1.4268
H	0.06095	2.77033	4.65264
H	1.54264	1.79787	4.64554
H	2.3364	1.86455	-0.55929
H	2.62557	3.17199	-2.63544
H	2.7426	5.66249	-2.53664
H	2.54218	6.81982	-0.34357
H	2.20097	5.52253	1.72496
C	5.40113	2.96674	0.64564
C	5.56338	4.27168	0.19181
C	5.99798	4.47917	-1.11296
C	6.26759	3.40318	-1.9691
C	6.11553	2.09947	-1.47329
C	5.69536	1.87283	-0.16796
H	5.34473	5.10876	0.85629
H	6.12226	5.50185	-1.47875
H	6.34184	1.24814	-2.12087
H	5.59631	0.85533	0.21492
C	6.68253	3.6461	-3.3874
H	7.39934	4.47765	-3.45896
H	5.80753	3.92068	-4.00093
H	7.13699	2.75217	-3.83694
S	4.87405	2.68162	2.31458
O	4.71461	3.98041	2.98123
O	5.76306	1.6742	2.90031

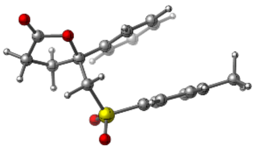

***S*-product**

Symbolic Z-matrix:

Charge = 0 Multiplicity = 1

C	2.04846	2.77065	-1.97148
C	1.61314	3.49507	-3.24561
C	3.2686	1.87678	-2.2089
C	0.87748	2.40925	-4.01356
C	0.32553	1.52277	-2.9349
O	0.94011	1.83122	-1.75697
O	-0.51213	0.66993	-2.98589
C	2.19975	3.60482	-0.72405
C	2.34021	2.95729	0.51169
C	2.52215	3.69092	1.67951
C	2.59144	5.08347	1.62282
C	2.47878	5.7311	0.39616
C	2.27822	4.99657	-0.77257
H	2.47377	3.92418	-3.77154
H	0.91666	4.30539	-2.98668
H	3.2077	1.35658	-3.17607
H	3.35504	1.10943	-1.4268
H	0.06095	2.77033	-4.65264
H	1.54264	1.79787	-4.64554
H	2.3364	1.86455	0.55929
H	2.62557	3.17199	2.63544
H	2.7426	5.66249	2.53664
H	2.54218	6.81982	0.34357
H	2.20097	5.52253	-1.72496
C	5.40113	2.96674	-0.64564
C	5.56338	4.27168	-0.19181
C	5.99798	4.47917	1.11296
C	6.26759	3.40318	1.9691
C	6.11553	2.09947	1.47329
C	5.69536	1.87283	0.16796
H	5.34473	5.10876	-0.85629
H	6.12226	5.50185	1.47875
H	6.34184	1.24814	2.12087
H	5.59631	0.85533	-0.21492
C	6.68253	3.6461	3.3874
H	7.39934	4.47765	3.45896
H	5.80753	3.92068	4.00093
H	7.13699	2.75217	3.83694
S	4.87405	2.68162	-2.31458
O	4.71461	3.98041	-2.98123
O	5.76306	1.6742	-2.90031

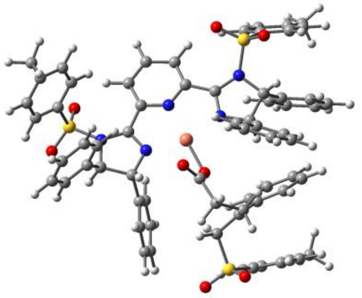

T1 test- int3

Symbolic Z-matrix:

Charge= 1 Multiplicity = 1

C	-2.22537	-3.51658	-1.47951
C	-1.78776	-2.41794	-0.74219
N	-0.48659	-2.13671	-0.68058
C	0.45978	-2.87082	-1.27025
C	0.09447	-4.00481	-1.99405
C	-1.26163	-4.30821	-2.09635
C	-2.58938	-1.37426	-0.03055
N	-3.93952	-1.30352	0.20329
C	-4.2607	0.04618	0.72242
C	-2.83859	0.6482	0.95482
N	-1.94874	-0.31507	0.34138
C	1.81064	-2.25314	-1.04784
N	1.80066	-1.08646	-0.48923
C	3.13777	-0.57491	-0.2853
C	4.06438	-1.74584	-0.72739
N	3.08024	-2.71792	-1.27984
Cu	0.04555	-0.37753	0.11777
C	-2.5206	0.9173	2.40715
C	-1.58771	0.167	3.12241
C	-1.30874	0.47049	4.45564
C	-1.9728	1.51697	5.09083
C	-2.92278	2.25968	4.38604
C	-3.19079	1.96402	3.05268
C	-5.13266	0.83092	-0.22378
C	-6.11483	1.67785	0.29674
C	-6.93404	2.41694	-0.55529
C	-6.7831	2.30691	-1.93707
C	-5.80179	1.46425	-2.46183
C	-4.97706	0.73413	-1.60962
C	3.38278	0.71002	-1.03611
C	4.28768	1.64208	-0.51751
C	4.5796	2.80816	-1.22198
C	3.96375	3.05757	-2.44841
C	3.05355	2.13644	-2.96529
C	2.7646	0.96621	-2.26316
C	4.88485	-2.33265	0.39036
C	4.26302	-2.8951	1.51174
C	5.02695	-3.43867	2.53945
C	6.4215	-3.41584	2.46142
C	7.04567	-2.84631	1.3534
C	6.27872	-2.30987	0.31858
C	-6.31931	-2.22462	-0.66615
C	-7.4424	-1.49083	-0.28417
C	-8.37052	-1.13638	-1.25424
C	-8.19502	-1.50425	-2.59593
C	-7.06681	-2.26303	-2.94093
C	-6.12366	-2.62506	-1.98738
C	-9.17833	-1.06713	-3.63474
S	-5.08078	-2.54793	0.5372
O	-5.61196	-2.25722	1.85727
O	-4.40708	-3.80905	0.27882
C	4.00661	-5.26047	-0.88318
C	2.92451	-5.8843	-0.26217
C	3.17591	-6.79199	0.75901
C	4.48694	-7.09036	1.16044
C	5.55013	-6.46356	0.49557
C	5.32225	-5.54643	-0.52249
C	4.75114	-8.03407	2.2908
S	3.72655	-4.05656	-2.13365
O	5.0016	-3.58221	-2.63744
O	2.70481	-4.51466	-3.06106
H	-3.27889	-3.76425	-1.55779
H	0.84233	-4.61304	-2.49579
H	-1.57208	-5.17906	-2.67709
H	-4.77594	-0.06421	1.68477
H	-2.75213	1.59979	0.40587
H	3.25912	-0.38303	0.7912
H	4.72584	-1.43072	-1.54305
H	-1.04998	-0.65044	2.63913
H	-0.55929	-0.11428	4.99404
H	-1.75293	1.75538	6.13443
H	-3.45118	3.08115	4.8765
H	-3.9235	2.55731	2.50036
H	-6.25082	1.74352	1.37983
H	-7.703	3.07112	-0.13702
H	-7.43269	2.8755	-2.60726
H	-5.6816	1.37089	-3.54402
H	-4.22302	0.06374	-2.02947
H	4.76271	1.4495	0.4488
H	5.27413	3.53993	-0.80377
H	4.17965	3.98252	-2.98625
H	2.56198	2.3297	-3.92248
H	2.0466	0.25049	-2.67187
H	3.17195	-2.90984	1.58269
H	4.53348	-3.88227	3.40765
H	7.02132	-3.8428	3.26907
H	8.13631	-2.82383	1.2888

H	6.76572	-1.87517	-0.55831
H	-7.57798	-1.1943	0.75678
H	-9.24909	-0.55458	-0.96465
H	-6.92404	-2.57249	-3.97911
H	-5.24718	-3.20507	-2.28162
H	-10.21332	-1.19136	-3.28284
H	-9.04054	0.00469	-3.85713
H	-9.05468	-1.62434	-4.57348
H	1.89669	-5.66886	-0.56068
H	2.33643	-7.28384	1.25653
H	6.57858	-6.69015	0.78746
H	6.1502	-5.04622	-1.02596
H	5.02969	-7.47127	3.19786
H	3.86617	-8.63944	2.53068
H	5.58963	-8.70812	2.06073
C	0.2311	3.94648	0.68055
C	0.39724	3.74626	2.15603
C	1.45938	4.11412	-0.13046
C	1.40794	2.66327	2.5329
C	1.15764	1.30897	1.89646
O	0.47817	1.34556	0.80027
O	1.597	0.27462	2.39481
C	-1.06311	4.04047	0.06188
C	-1.24178	3.85659	-1.33426
C	-2.49271	3.96046	-1.92602
C	-3.62136	4.26729	-1.15876
C	-3.47204	4.45499	0.2178
C	-2.2254	4.33464	0.81923
H	0.72151	4.69502	2.62224
H	-0.57075	3.48467	2.60524
H	2.35546	3.67643	0.33209
H	1.39988	3.73147	-1.15774
H	1.42865	2.52116	3.62308
H	2.43563	2.95821	2.25779
H	-0.38358	3.61324	-1.96294
H	-2.59243	3.80175	-3.00321
H	-4.60448	4.35337	-1.62635
H	-4.3431	4.70223	0.83076
H	-2.1386	4.50394	1.89317
C	0.63791	6.58418	-1.25088
C	-0.45933	7.14189	-0.59687
C	-1.53243	7.59875	-1.35186
C	-1.52272	7.50999	-2.75195
C	-0.39205	6.97206	-3.38144
C	0.68957	6.50538	-2.64016
H	-0.46864	7.20449	0.49268
H	-2.40201	8.02644	-0.84582
H	-0.36193	6.91448	-4.47274
H	1.56675	6.07997	-3.13277
C	-2.71049	7.95703	-3.54687
H	-3.07566	8.93791	-3.20632
H	-3.54265	7.24349	-3.42296
H	-2.48006	8.02297	-4.61937
S	1.94569	5.86128	-0.29384
O	1.92205	6.45862	1.04802
O	3.18326	5.91619	-1.08235

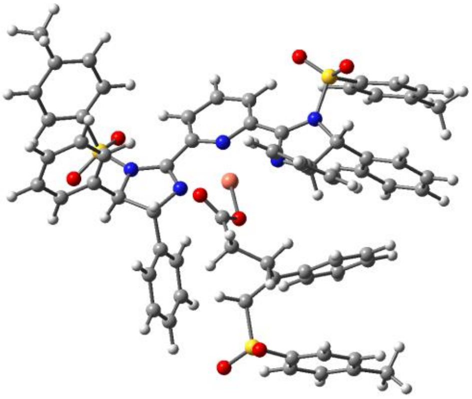

T1 test- T1

Symbolic Z-matrix:

Charge = 1 Multiplicity = 3

C	-2.22544	-3.51654	-1.47954
C	-1.78779	-2.41793	-0.74222
N	-0.48662	-2.13673	-0.6806
C	0.45974	-2.87087	-1.27026
C	0.0944	-4.00484	-1.99407
C	-1.26171	-4.3082	-2.09638
C	-2.58939	-1.37422	-0.03059
N	-3.93953	-1.30342	0.20323
C	-4.26067	0.04629	0.72233
C	-2.83855	0.64826	0.95477
N	-1.94872	-0.31505	0.34135
C	1.81062	-2.25323	-1.04783
N	1.80066	-1.08654	-0.48922
C	3.13779	-0.57502	-0.28528
C	4.06437	-1.74597	-0.72738
N	3.08021	-2.71804	-1.2798
Cu	0.04557	-0.37757	0.11775
C	-2.52057	0.91734	2.40711
C	-1.58771	0.16701	3.12237
C	-1.30875	0.47049	4.45561
C	-1.97279	1.51699	5.0908
C	-2.92274	2.25972	4.386
C	-3.19075	1.96407	3.05263
C	-5.13256	0.83106	-0.22391
C	-6.1147	1.67805	0.29657
C	-6.93385	2.41717	-0.5555

C	-6.78287	2.30711	-1.93727	H	-5.68138	1.37101	-3.54417
C	-5.80159	1.46439	-2.46198	H	-4.22291	0.06381	-2.02955
C	-4.97693	0.73424	-1.60974	H	4.7628	1.44932	0.4488
C	3.38281	0.70991	-1.03608	H	5.27424	3.53977	-0.80373
C	4.28774	1.64193	-0.51749	H	4.17972	3.98243	-2.98617
C	4.57969	2.80803	-1.22194	H	2.56199	2.32968	-3.9224
C	3.96381	3.05748	-2.44835	H	2.04658	0.25045	-2.67182
C	3.05357	2.13638	-2.96523	H	3.17198	-2.90981	1.58279
C	2.76461	0.96614	-2.26312	H	4.53355	-3.88223	3.40774
C	4.88486	-2.33276	0.39036	H	7.02138	-3.84289	3.26905
C	4.26304	-2.89513	1.5118	H	8.13634	-2.82406	1.28869
C	5.027	-3.43869	2.5395	H	6.76571	-1.87542	-0.55841
C	6.42154	-3.41593	2.46141	H	-7.57803	-1.19408	0.75663
C	7.0457	-2.84648	1.35334	H	-9.24905	-0.55429	-0.96484
C	6.27873	-2.31005	0.31852	H	-6.92396	-2.57222	-3.97927
C	-6.31933	-2.22442	-0.66627	H	-5.24718	-3.20488	-2.28172
C	-7.44241	-1.4906	-0.28432	H	-10.21322	-1.19096	-3.28307
C	-8.37049	-1.13611	-1.25441	H	-9.04036	0.00502	-3.85733
C	-8.19496	-1.50397	-2.59611	H	-9.05456	-1.624	-4.57368
C	-7.06675	-2.26277	-2.94108	H	1.89684	-5.66905	-0.56036
C	-6.12365	-2.62484	-1.98751	H	2.3368	-7.2839	1.2569
C	-9.17822	-1.06679	-3.63495	H	6.57889	-6.69001	0.78749
S	-5.08085	-2.54778	0.53711	H	6.15028	-5.04619	-1.02598
O	-5.61206	-2.25706	1.85717	H	5.03023	-7.47109	3.19804
O	-4.40721	-3.80893	0.27875	H	3.8667	-8.63933	2.53103
C	4.00672	-5.26055	-0.88304	H	5.59012	-8.70798	2.06093
C	2.92469	-5.88441	-0.26192	C	0.23096	3.94636	0.6805
C	3.17622	-6.79203	0.75929	C	0.39729	3.74624	2.15597
C	4.48729	-7.09031	1.16064	C	1.45915	4.11389	-0.13068
C	5.5504	-6.46349	0.49566	C	1.40803	2.66327	2.53281
C	5.3224	-5.54643	-0.52243	C	1.1577	1.30896	1.8964
C	4.75163	-8.03394	2.29103	O	0.47822	1.34554	0.80022
S	3.72652	-4.05672	-2.13356	O	1.5971	0.27462	2.39473
O	5.00153	-3.58234	-2.63744	C	-1.06332	4.04029	0.06199
O	2.70476	-4.5149	-3.0609	C	-1.24218	3.85628	-1.33411
H	-3.27895	-3.76419	-1.55783	C	-2.49319	3.96008	-1.92571
H	0.84225	-4.61309	-2.49582	C	-3.62174	4.26696	-1.15833
H	-1.57218	-5.17904	-2.67713	C	-3.47224	4.4548	0.21819
H	-4.77595	-0.06406	1.68467	C	-2.22551	4.33453	0.81947
H	-2.75203	1.59984	0.40583	H	0.72163	4.69503	2.62207
H	3.25913	-0.38314	0.79121	H	-0.57065	3.4847	2.60532
H	4.72582	-1.43086	-1.54306	H	2.35526	3.67614	0.33177
H	-1.04999	-0.65043	2.6391	H	1.39949	3.73121	-1.15794
H	-0.55932	-0.1143	4.99402	H	1.4288	2.52118	3.62298
H	-1.75292	1.75538	6.1344	H	2.4357	2.95821	2.25762
H	-3.45112	3.0812	4.87645	H	-0.38405	3.61287	-1.96288
H	-3.92343	2.55738	2.50031	H	-2.59305	3.80126	-3.00287
H	-6.25072	1.74374	1.37966	H	-4.60492	4.353	-1.6258
H	-7.70279	3.0714	-0.13726	H	-4.34321	4.70209	0.83125
H	-7.43241	2.87573	-2.60748	H	-2.13857	4.50392	1.89338

C	0.63775	6.58403	-1.25103
C	-0.45931	7.14191	-0.59686
C	-1.53246	7.5989	-1.3517
C	-1.52298	7.5101	-2.75179
C	-0.39247	6.972	-3.38144
C	0.68919	6.50519	-2.64032
H	-0.46844	7.20454	0.49268
H	-2.4019	8.02671	-0.84554
H	-0.36253	6.91438	-4.47275
H	1.56624	6.07966	-3.13305
C	-2.71082	7.95727	-3.54654
H	-3.07579	8.93821	-3.20597
H	-3.54306	7.24386	-3.42247
H	-2.48055	8.02313	-4.61908
S	1.9456	5.86101	-0.29417
O	1.92221	6.45837	1.04769
O	3.18305	5.91578	-1.08287

Cu-C scan

Symbolic Z-matrix:

Charge = 1 Multiplicity = 1

C	-2.98183	-3.29511	0.51597
C	-2.47211	-2.00164	0.53099
N	-1.15682	-1.74777	0.61668
C	-0.30479	-2.76788	0.69455
C	-0.73076	-4.09882	0.70501
C	-2.09077	-4.36122	0.61613
C	-3.29781	-0.79563	0.31577
N	-4.66132	-0.69288	0.54757
C	-5.11491	0.6101	0.00692
C	-3.7686	1.23937	-0.48764
N	-2.75632	0.23185	-0.2279
C	1.13618	-2.43737	0.82203
N	1.59287	-1.65875	1.7164
C	3.04856	-1.67389	1.65551
C	3.42956	-2.62617	0.48056
N	2.0975	-3.08476	0.02681
Cu	-0.68856	0.21372	0.23642
C	-3.42912	2.56687	0.13502
C	-2.93078	2.634	1.44011
C	-2.63773	3.86788	2.01992
C	-2.8352	5.04586	1.29768
C	-3.33122	4.98522	-0.00553
C	-3.62753	3.75039	-0.58248
C	-6.14675	0.43375	-1.07223
C	-7.3706	1.10137	-0.99273
C	-8.33934	0.91948	-1.98209
C	-8.08979	0.06385	-3.05412
C	-6.86541	-0.60505	-3.13997
C	-5.89917	-0.41952	-2.15514
C	3.64098	-0.27898	1.61853
C	3.0406	0.72976	2.38259
C	3.59171	2.00886	2.43038
C	4.74638	2.30553	1.7036
C	5.34672	1.30955	0.93579
C	4.80207	0.0244	0.89979
C	4.31561	-3.78568	0.85425
C	3.90016	-4.71233	1.81807
C	4.69495	-5.81322	2.1273
C	5.91712	-5.99914	1.47666
C	6.34054	-5.0757	0.52161
C	5.54111	-3.97438	0.21243
C	-6.35557	-2.67247	1.43749
C	-7.37632	-2.48689	0.50135
C	-8.13336	-3.58368	0.11398
C	-7.90093	-4.85944	0.65615
C	-6.88447	-5.00538	1.6075
C	-6.10729	-3.91957	2.00555
C	-8.72968	-6.02831	0.21824
S	-5.40402	-1.28865	1.97622
O	-6.31227	-0.23423	2.40024
O	-4.3358	-1.75309	2.84999
C	2.96869	-4.6162	-1.97481
C	2.75441	-5.82633	-1.31067
C	3.62626	-6.8787	-1.54752
C	4.69801	-6.74741	-2.44748
C	4.86927	-5.52818	-3.11317
C	4.01502	-4.45386	-2.87812
C	5.64769	-7.88597	-2.65764
S	1.9082	-3.25332	-1.65497
O	2.46446	-2.07166	-2.31302
O	0.51145	-3.59674	-1.88975
H	-4.05163	-3.47262	0.39658
H	0.00116	-4.90622	0.77641
H	-2.45973	-5.39	0.60975
H	-5.53759	1.20528	0.82614
H	-3.82314	1.37511	-1.57985
H	3.39169	-2.14383	2.59626
H	3.89681	-2.05998	-0.33831
H	-2.76549	1.7148	2.01078
H	-2.24342	3.90634	3.03913
H	-2.60199	6.01358	1.75228
H	-3.48785	5.90524	-0.57693
H	-4.01449	3.70157	-1.60536
H	-7.57069	1.76002	-0.1416

H	-9.29657	1.44446	-1.90893
H	-8.85062	-0.08454	-3.82656
H	-6.66579	-1.27832	-3.97919
H	-4.94483	-0.95341	-2.22002
H	2.12749	0.50852	2.93888
H	3.11209	2.77988	3.04094
H	5.17574	3.3116	1.73348
H	6.24552	1.53084	0.35392
H	5.30747	-0.74127	0.30471
H	2.93762	-4.57992	2.32364
H	4.35818	-6.53375	2.87883
H	6.54019	-6.86626	1.71636
H	7.29575	-5.21581	0.00654
H	5.86721	-3.2585	-0.54894
H	-7.57715	-1.50059	0.07462
H	-8.92884	-3.44771	-0.62523
H	-6.69586	-5.98814	2.04998
H	-5.3141	-4.03924	2.74739
H	-9.80435	-5.8318	0.381
H	-8.59773	-6.21992	-0.86209
H	-8.45882	-6.94514	0.7659
H	1.92827	-5.93772	-0.60307
H	3.47957	-7.82572	-1.01946
H	5.69594	-5.41075	-3.82017
H	4.16693	-3.49508	-3.3792
H	6.25592	-8.04964	-1.74868
H	5.10565	-8.82667	-2.85731
H	6.33472	-7.69186	-3.49671
C	0.79714	2.39311	-0.8839
C	0.48385	3.8613	-0.58453
C	2.29955	2.13221	-1.02684
C	0.78368	3.98765	0.90128
C	0.44594	2.63252	1.44665
O	0.43417	1.74278	0.38969
O	0.19941	2.26353	2.55175
C	-0.03849	1.70342	-1.94404
C	0.20608	0.34611	-2.21566
C	-0.60141	-0.36031	-3.10479
C	-1.65071	0.28675	-3.75704
C	-1.87337	1.64385	-3.52625
C	-1.07704	2.34725	-2.62199
H	1.07096	4.53293	-1.22482
H	-0.58702	4.05331	-0.7446
H	2.86849	2.62632	-0.22066
H	2.53566	1.05933	-0.96829
H	0.19267	4.75743	1.41834
H	1.84987	4.18624	1.11375
H	1.05985	-0.1758	-1.77643
H	-0.39334	-1.41832	-3.28494
H	-2.28539	-0.26288	-4.45875
H	-2.68063	2.1646	-4.04967
H	-1.27767	3.4065	-2.45452
C	2.58733	1.77139	-3.86317
C	1.62873	2.29011	-4.7332
C	1.18797	1.504	-5.79017
C	1.68875	0.20753	-5.98798
C	2.66559	-0.27537	-5.10809
C	3.1241	0.49959	-4.04737
H	1.23745	3.29768	-4.57658
H	0.43288	1.90072	-6.47605
H	3.0691	-1.28198	-5.24972
H	3.88269	0.11235	-3.36378
C	1.15904	-0.64444	-7.10154
H	1.10449	-0.07994	-8.04841
H	0.13307	-0.98655	-6.86867
H	1.78371	-1.53796	-7.26199
S	3.14145	2.75092	-2.49711
O	2.71893	4.1387	-2.72345
O	4.55749	2.45479	-2.26666

Cu-O scan 21

Symbolic Z-matrix:

Charge = 1 Multiplicity = 1

C	-2.84238	-3.1141	-0.04236
C	-2.33744	-1.84967	0.25009
N	-1.02435	-1.62288	0.39422
C	-0.16907	-2.64834	0.30556
C	-0.6028	-3.95492	0.07742
C	-1.95926	-4.18405	-0.11638
C	-3.19646	-0.64716	0.30724
N	-4.55669	-0.6585	0.60907
C	-5.06211	0.72298	0.42847
C	-3.81022	1.43904	-0.16656
N	-2.72035	0.48272	-0.05623
C	1.25728	-2.34696	0.56762
N	1.60483	-1.4816	1.43287
C	3.04625	-1.54317	1.61865
C	3.57297	-2.55392	0.55873
N	2.30803	-3.08684	-0.01303
Cu	-0.50208	0.28343	0.84398
C	-3.48243	2.78398	0.41962
C	-3.03128	2.89497	1.73879
C	-2.68395	4.13865	2.26116
C	-2.79306	5.28509	1.47126
C	-3.25191	5.18348	0.15795

C	-3.59235	3.9356	-0.36565
C	-6.27553	0.77612	-0.45705
C	-7.41432	1.47507	-0.05172
C	-8.54961	1.50684	-0.86413
C	-8.55335	0.83548	-2.08614
C	-7.41488	0.13797	-2.49888
C	-6.2823	0.11076	-1.6895
C	3.69338	-0.17418	1.63591
C	3.05418	0.85918	2.33475
C	3.61668	2.13293	2.39024
C	4.83024	2.39175	1.74947
C	5.47689	1.36658	1.06356
C	4.91323	0.09057	1.00837
C	4.45772	-3.64079	1.10655
C	3.95111	-4.58596	2.00613
C	4.76931	-5.60111	2.49569
C	6.10492	-5.67967	2.094
C	6.61759	-4.73515	1.20554
C	5.79491	-3.72113	0.71386
C	-6.30039	-2.72867	1.13553
C	-7.48534	-2.27139	0.5522
C	-8.3329	-3.19216	-0.0481
C	-8.03005	-4.56408	-0.0597
C	-6.85002	-4.99218	0.56062
C	-5.98065	-4.0845	1.1618
C	-8.96749	-5.53883	-0.70449
S	-5.21649	-1.57247	1.90925
O	-6.01925	-0.63107	2.67424
O	-4.10835	-2.28769	2.52668
C	3.63128	-4.52839	-1.8365
C	3.46039	-5.7692	-1.21668
C	4.48403	-6.70039	-1.30397
C	5.66665	-6.41867	-2.00967
C	5.79265	-5.17494	-2.63971
C	4.78385	-4.21936	-2.55395
C	6.77302	-7.42644	-2.05217
S	2.36985	-3.31961	-1.70656
O	2.84486	-2.07712	-2.32472
O	1.07706	-3.84035	-2.12829
H	-3.90949	-3.25502	-0.21942
H	0.11766	-4.77254	0.05078
H	-2.32739	-5.19234	-0.32244
H	-5.30596	1.14148	1.41494
H	-3.9865	1.578	-1.24719
H	3.19849	-1.99196	2.61885
H	4.10552	-2.01324	-0.23819
H	-2.92788	1.99996	2.35847
H	-2.30476	4.21015	3.2836
H	-2.51391	6.26034	1.88158
H	-3.34065	6.07904	-0.46464
H	-3.94759	3.85317	-1.39843
H	-7.41603	1.98726	0.91536
H	-9.43819	2.0539	-0.53488
H	-9.44555	0.85291	-2.71956
H	-7.41439	-0.39377	-3.45533
H	-5.3993	-0.45275	-2.00879
H	2.09451	0.66974	2.82062
H	3.08639	2.92839	2.92099
H	5.2679	3.39411	1.7835
H	6.42551	1.55757	0.55331
H	5.44571	-0.6923	0.46283
H	2.90246	-4.53574	2.31711
H	4.3627	-6.33821	3.19485
H	6.74682	-6.47907	2.47666
H	7.66219	-4.79122	0.88467
H	6.19272	-2.98906	0.0041
H	-7.74489	-1.20997	0.56557
H	-9.25679	-2.83855	-0.51617
H	-6.60589	-6.05861	0.57876
H	-5.06717	-4.42713	1.65372
H	-9.91888	-5.59235	-0.14421
H	-9.21736	-5.22678	-1.73374
H	-8.53712	-6.55239	-0.74147
H	2.54967	-5.9986	-0.65704
H	4.37079	-7.66852	-0.80714
H	6.70451	-4.94166	-3.19737
H	4.89541	-3.24068	-3.02648
H	7.21515	-7.5513	-1.04635
H	6.39834	-8.41663	-2.36506
H	7.57567	-7.12073	-2.74193
C	0.4471	1.32274	-1.34205
C	0.08981	2.65602	-0.76707
C	1.83723	0.84992	-1.19626
C	0.82796	3.23925	0.43014
C	0.41073	2.84378	1.8614
O	-0.1119	1.68698	2.06866
O	0.64755	3.65265	2.74726
C	-0.43348	0.6862	-2.28319
C	-0.13258	-0.58842	-2.82626
C	-0.97015	-1.18455	-3.75298
C	-2.12042	-0.51712	-4.1843
C	-2.43464	0.74789	-3.67353
C	-1.61526	1.33524	-2.72624
H	0.28894	3.35211	-1.61457
H	-0.99751	2.71929	-0.60196
H	2.29722	1.12159	-0.23288

H	2.00185	-0.21633	-1.40045	C	2.77419	0.06192	-4.59354
H	0.70567	4.3321	0.38854	H	1.03337	2.96647	-4.09191
H	1.91604	3.07448	0.35276	H	0.00181	2.24213	-6.23161
H	0.76133	-1.12909	-2.52032	H	2.53494	-1.2464	-6.28414
H	-0.7209	-2.17434	-4.1433	H	3.54686	-0.54553	-4.1194
H	-2.77667	-0.98394	-4.92504	C	0.53595	0.00407	-7.67588
H	-3.33031	1.27177	-4.01809	H	0.59591	0.79814	-8.44102
H	-1.87475	2.3227	-2.34389	H	-0.53703	-0.20642	-7.51308
C	2.33394	1.23552	-3.9863	H	1.00586	-0.90574	-8.08225
C	1.34871	2.03573	-4.57058	S	2.97874	1.68214	-2.40384
C	0.77927	1.62632	-5.76897	O	2.82705	3.12659	-2.22193
C	1.18608	0.43826	-6.39798	O	4.27559	1.04082	-2.20708
C	2.19684	-0.32538	-5.7997				

11. Mass spectrometry experiments and proposed reaction mechanism

The experiments were performed with a Waters Vion IMS Qtof instrument with an electrospray ion source, it was purchased from the Waters company. The instrument equipped a quadrupole mass filter after ion source for the selection of target ions. In the collision induced dissociation (CID) process, the mass-selected ions enter an argonfilled linear ion trap after accelerated by electric field. After collision, the ions enter a reflectron time-of-flight (TOF) region for determination. For a given ion of interest, it's type is defined via mass-to-charge ratio and collision induced dissociation. Collision induced dissociation was also employed to analyze structural fragments.

Procedure: In an argon fulfilled glovebox, SOgen (177.0 mg, 0.41 mmol), 1-methyl-4-vinylbenzene (47.3 mg, 0.40 mmol), were added into chamber A with a magnetic stirring bar, followed by addition of tetradecane (1.0 mL). $Cu(MeCN)_4PF_6$ (3.7 mg, 0.010 mmol), **L1** (9.9 mg, 0.012 mmol), Compound **1** (0.2 mmol, 1.0 equiv), Compound **4** (0.4 mmol, 2 equiv), Na_2CO_3 (0.22 mmol, 1.1 equiv) and 4Å MS (60 mg) and 2-Me-THF (2.0 mL) were added into chamber B with a magnetic stirring bar. The two-chamber was sealed and removed out of the glovebox. The chamber A was allowed to stir at 100 ^{o}C using heating mantle with 600-800 rpm stirring speed for 10 min. The two-chamber was allowed to stir at room temperature for 3 h. After 3 h, 10.0 ul reaction mixture was picked up with pipette and dissolved into 1.0 ml MeCN. Next, this dilution was transferred into an injection syringe, and injected into the high-resolution electrospray mass spectrometry by injection pump. At last, we collected and analysed MS data.

MS Instrument Type: Waters Vion® IMS QTof

Experiment Settings:

Experiment type:	ESI$^+$	Scan Mode:	MS
Capillary voltage:	3.0kv	Low mass:	50m/z
Soure temperature:	120°C	High mass:	2000m/z
Desolvation temperature:	450°C	Scan time:	0.200s
Cone gas:	50L/h		
Desolvation gas:	800L/h		

	Formula	Neutral mass (Da)	Observed m/z	Mass error (mDa)	Mass error (ppm)	Response	Adducts
1	C67H59CuN5O8S3	1220.28218	610.14161	2.1	1.8	15746	2x(-e)
2	C60H52CuN5O6S2	1065.26550	1065.26493	0.0	0.0	49280	-e
3	C49H41CuN5O4S2	890.18960	890.18830	-0.8	-0.8	52626	-e
4	C18H18O4S	330.09258	353.08226	0.5	1.3	6345868	+Na

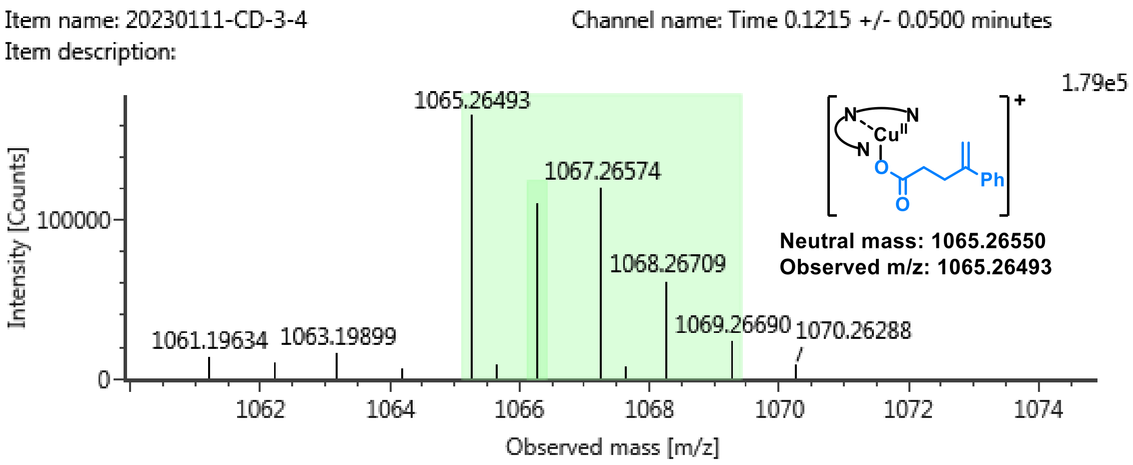

Figure S12 HRMS analysis of the Cu^{II} complex **IV**.

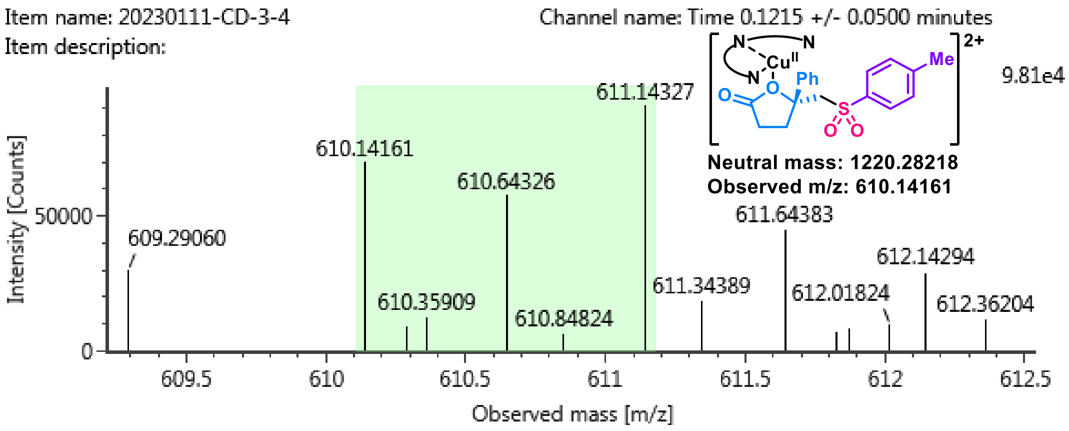

Figure S13 HRMS analysis of the Cu^{II} complex **VI**.

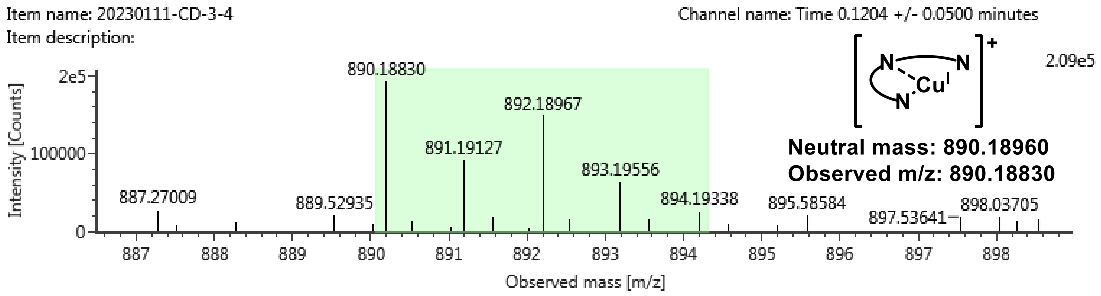

Figure S14 HRMS analysis of the Cu^{II} complex **VII**.

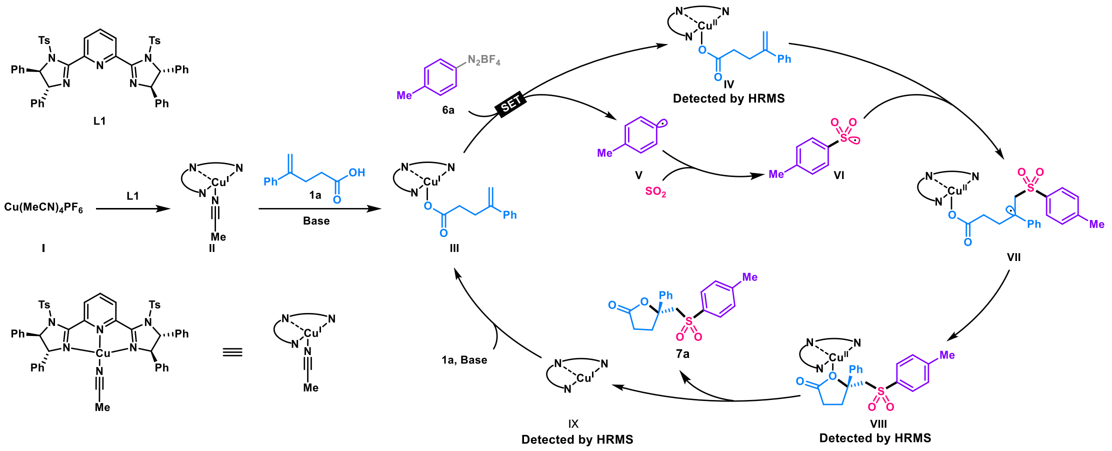

Figure S15 Proposed reaction mechanism.

12. References

[1] S. Alazet, F. Le Vaillant, S. Nicolai, T. Courant, J. Waser, *Chemistry – A European Journal* **2017**, *23*, 9501.

[2] K. Zhang, X. Zhang, J. Chen, Z. Liu, C. Pan, Y. Zhu, S. Wu, B. Fan, *Chemistry – An Asian Journal* **2021**, *16*, 1229.

[3] X. Jiang, S. Liu, S. Yang, M. Jing, L. Xu, P. Yu, Y. Wang, Y. Yeung, *Organic Letters* **2018**, *20*, 3259.

[4] Y. Wang, L. Deng, J. Zhou, X. Wang, H. Mei, J. Han, Y. Pan, *Advanced Synthesis & Catalysis* **2018**, *360*, 1060.

[5] J. Chen, Y. Liang, P. Wang, G. Li, B. Zhang, H. Qian, X. Huan, W. Guan, W. Xiao, J. Chen, *Journal of the American Chemical Society* **2021**, *143*, 13382.

[6] P. Wang, X. Wu, Y. Cheng, M. Jiang, W. Xiao, J. Chen, *Angewandte Chemie International Edition* **2021**, *60*, 22956.

[7] H. Zhao, X. Chen, H. Jiang, M. Zhang, *Organic Chemistry Frontiers* **2018**, *5*, 539.
[8] A. M. Berman, J. S. Johnson, *The Journal of Organic Chemistry* **2006**, *71*, 219.
[9] J. Jeon, C. Lee, H. Seo, S. Hong, *Journal of the American Chemical Society* **2020**, *142*, 20470.
[10] J. Wu, L. Li, M. Liu, L. Bai, X. Luan, *Angewandte Chemie International Edition* **2022**, *61*, e202113820.
[11] G. Chen, J. Xu, B. Xiong, H. Song, X. Zhang, X. Ma, Z. Lian, *Organic Letters* **2022**, *24*, 1207.
[12] Zhu, R.; Buchwald, S. L., Versatile enantioselective synthesis of functionalized lactones via copper-catalyzed radical oxyfunctionalization of alkenes. *Journal of the American Chemical Society* **2015,** *137*, 8069-8077.
[13] Satyanarayana, T.; Abraham, S.; Kagan, H. B., Nonlinear effects in asymmetric catalysis. *Angewandte Chemie International Edition* **2009,** *48*, 456-494.
[14] Gaussian 16, Revision B.01, M. J. Frisch, G. W. Trucks, H. B. Schlegel, G. E. Scuseria, M. A. Robb, J. R. Cheeseman, G. Scalmani, V. Barone, G. A. Petersson, H. Nakatsuji, X. Li, M. Caricato, A. V. Marenich, J. Bloino, B. G. Janesko, R. Gomperts, B. Mennucci, H. P. Hratchian, J. V. Ortiz, A. F. Izmaylov, J. L. Sonnenberg, D. Williams-Young, F. Ding, F. Lipparini, F. Egidi, J. Goings, B. Peng, A. Petrone, T. Henderson, D. Ranasinghe, V. G. Zakrzewski, J. Gao, N. Rega, G. Zheng, W. Liang, M. Hada, M. Ehara, K. Toyota, R. Fukuda, J. Hasegawa, M. Ishida, T. Nakajima, Y. Honda, O. Kitao, H. Nakai, T. Vreven, K. Throssell, J. A. Montgomery, Jr., J. E. Peralta, F. Ogliaro, M. J. Bearpark, J. J. Heyd, E. N. Brothers, K. N. Kudin, V. N. Staroverov, T. A. Keith, R. Kobayashi, J. Normand, K. Raghavachari, A. P. Rendell, J. C. Burant, S. S. Iyengar, J. Tomasi, M. Cossi, J. M. Millam, M. Klene, C. Adamo, R. Cammi, J. W. Ochterski, R. L. Martin, K. Morokuma, O. Farkas, J. B. Foresman, and D. J. Fox, Gaussian, Inc., Wallingford CT, 2016.
[15] Adamo C., Barone V. Toward reliable density functional methods without adjustable parameters: The PBE0 model. *Journal of Chemical Physics* **1999**, *110*, 6158-6170.
[16] Grimme S., Antony J., Ehrlich S., Krieg H., A consistent and accurate ab initio parametrization of density functional dispersion correction (DFT-D) for the 94 elements H-Pu. *Journal of Chemical Physics* **2010**, *132*, 154104.
[17] Marenich, A. V.; Cramer, C. J.; Truhlar, D. G., Universal solvation model based

on solute electron density and on a continuum model of the solvent defined by the bulk dielectric constant and atomic surface tensions. *The Journal of Physical Chemistry B* **2009,** *113*, (18), 6378.

[18] Lu, T.; Chen, F., *The Journal of Computational Chemistry* **2012**, *33,* 580-592.

[19] Humphrey et al., *J. Molec. Graphics* **1996**, *14* (1), 33-38.

13. The spectrums of ^{1}H NMR, ^{13}C NMR, ^{19}F NMR, HPLC chromatograms.

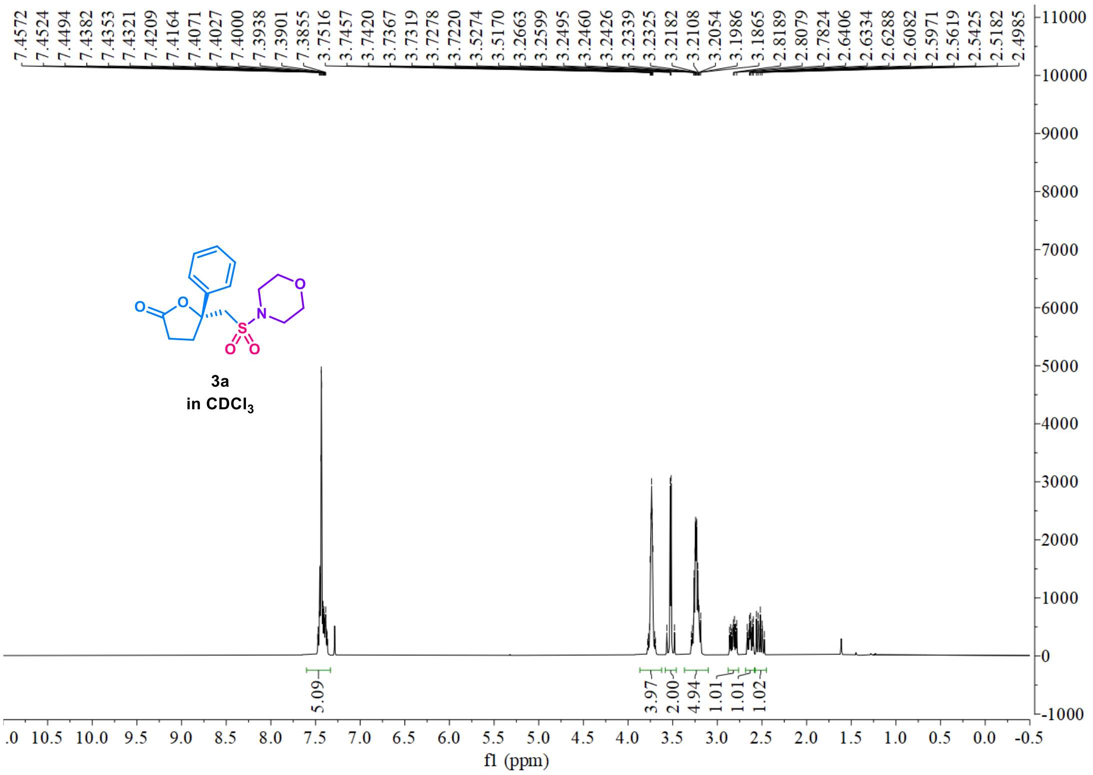

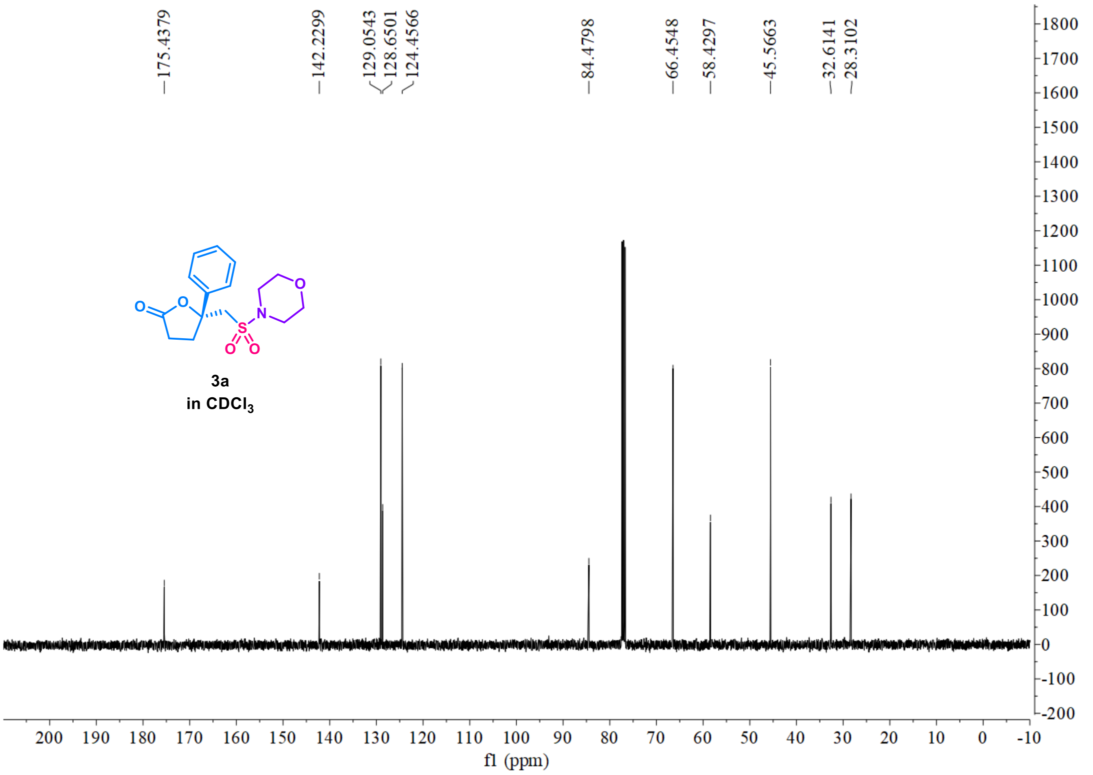

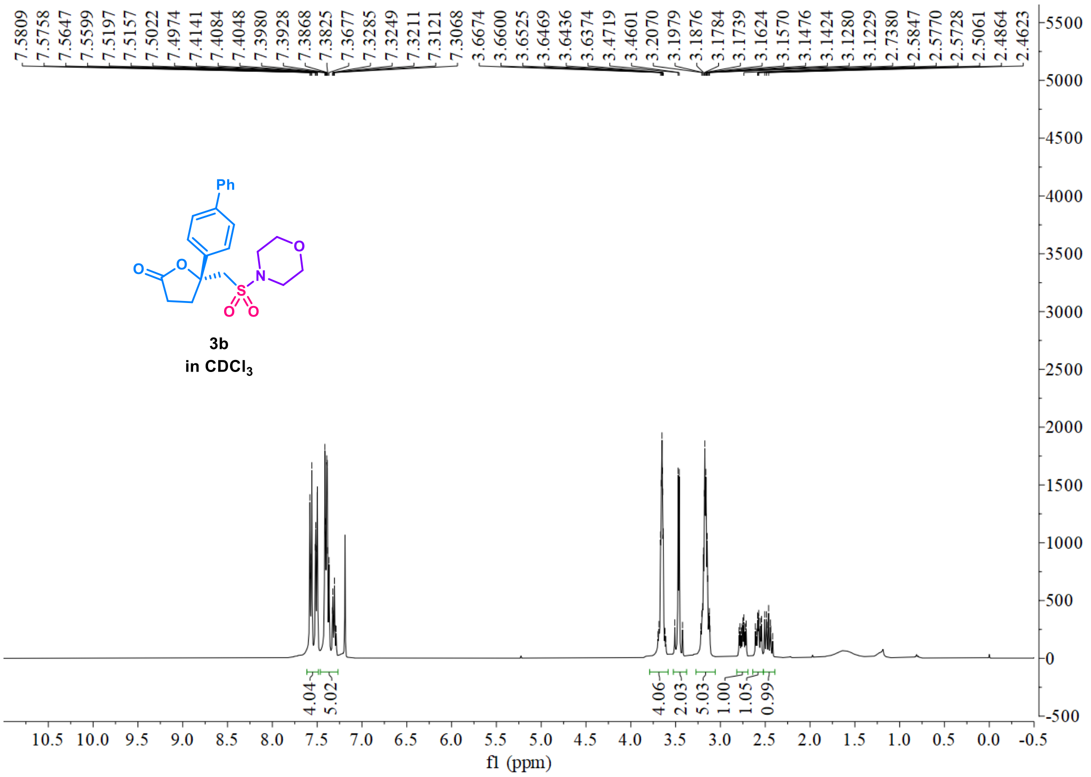
7.5809
7.5758
7.5647
7.5599
7.5197
7.5157
7.5022
7.4974
7.4141
7.4084
7.4048
7.3980
7.3928
7.3868
7.3825
7.3677
7.3285
7.3249
7.3211
7.3121
7.3068
3.6674
3.6600
3.6525
3.6469
3.6436
3.6374
3.4719
3.4601
3.2070
3.1979
3.1876
3.1784
3.1739
3.1624
3.1570
3.1476
3.1424
3.1280
3.1229
2.7380
2.5847
2.5770
2.5728
2.5061
2.4864
2.4623
Ph
O
O
S
O
O
N
O
3b
in $CDCl_3$
4.04
5.02
4.06
2.03
5.03
1.00
1.05
0.99
f1 (ppm)

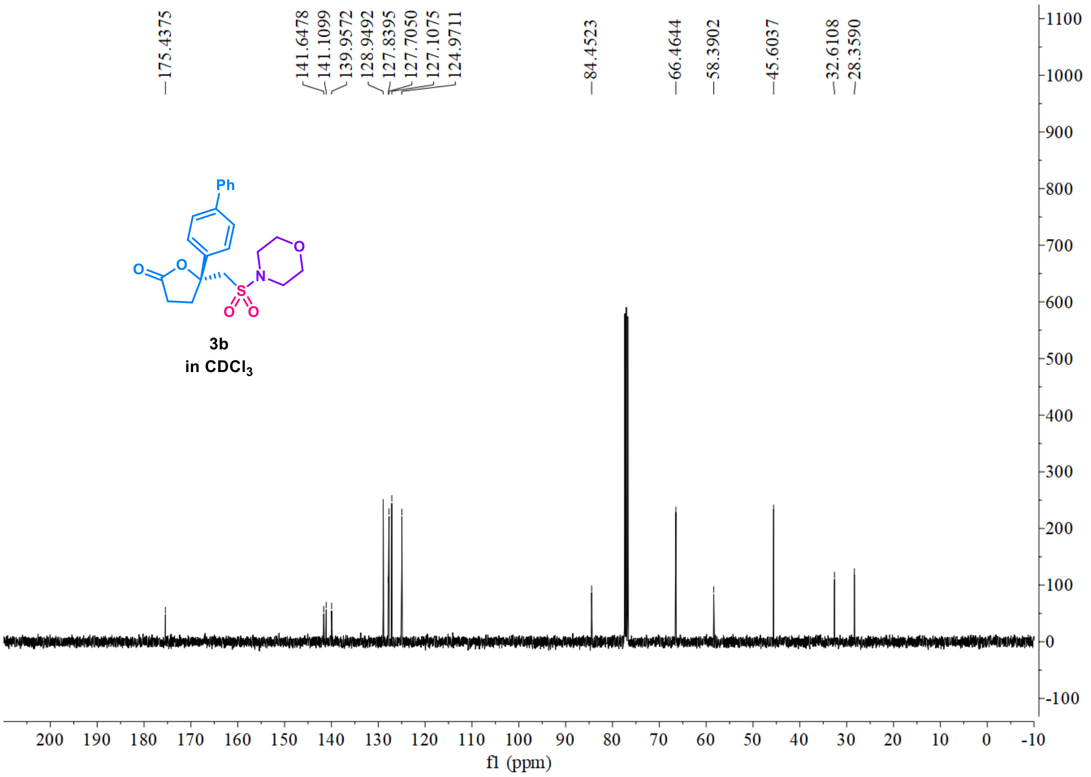
175.4375
141.6478
141.1099
139.9572
128.9492
127.8395
127.7050
127.1075
124.9711
84.4523
66.4644
58.3902
45.6037
32.6108
28.3590
Ph
O
O
S
O
O
N
O
3b
in $CDCl_3$
f1 (ppm)

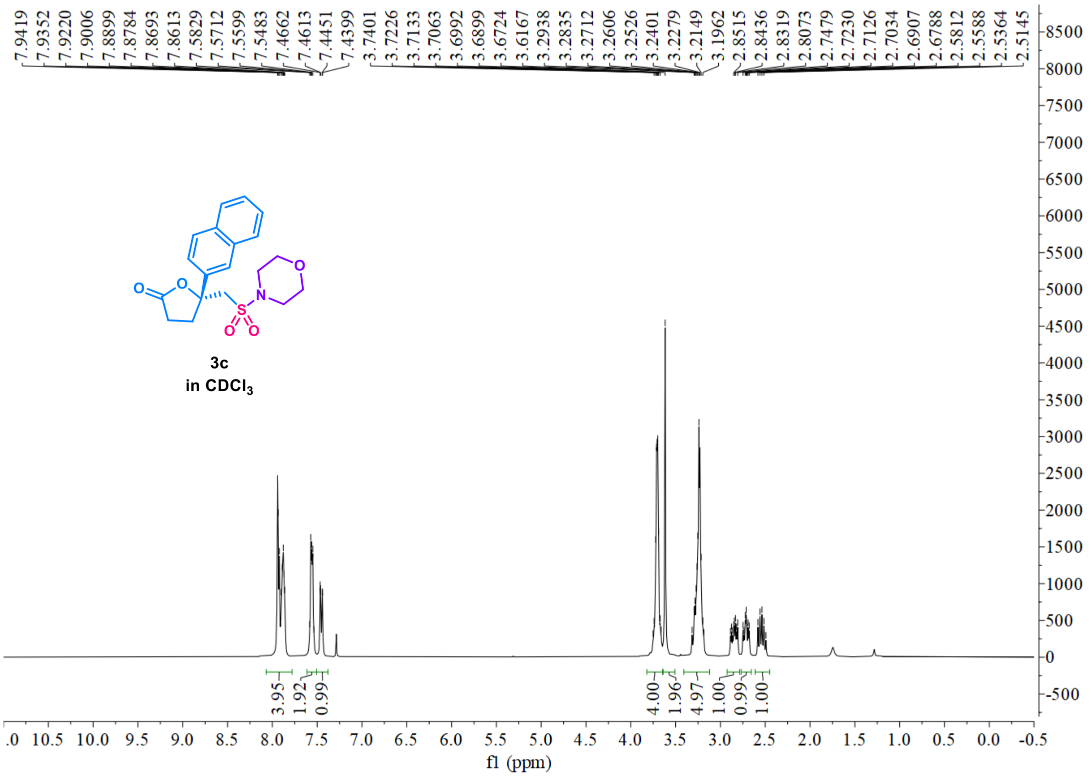
7.9419
7.9352
7.9220
7.9006
7.8899
7.8784
7.8693
7.8613
7.5829
7.5712
7.5599
7.5483
7.4662
7.4613
7.4451
7.4399
3.7401
3.7226
3.7133
3.7063
3.6992
3.6899
3.6724
3.6167
3.2938
3.2835
3.2712
3.2606
3.2526
3.2401
3.2279
3.2149
3.1962
2.8515
2.8436
2.8319
2.8073
2.7479
2.7230
2.7126
2.7034
2.6907
2.6788
2.5812
2.5588
2.5364
2.5145
3c
in CDCl$_3$
3.95
1.92
0.99
4.00
1.96
4.97
1.00
0.99
1.00
f1 (ppm)

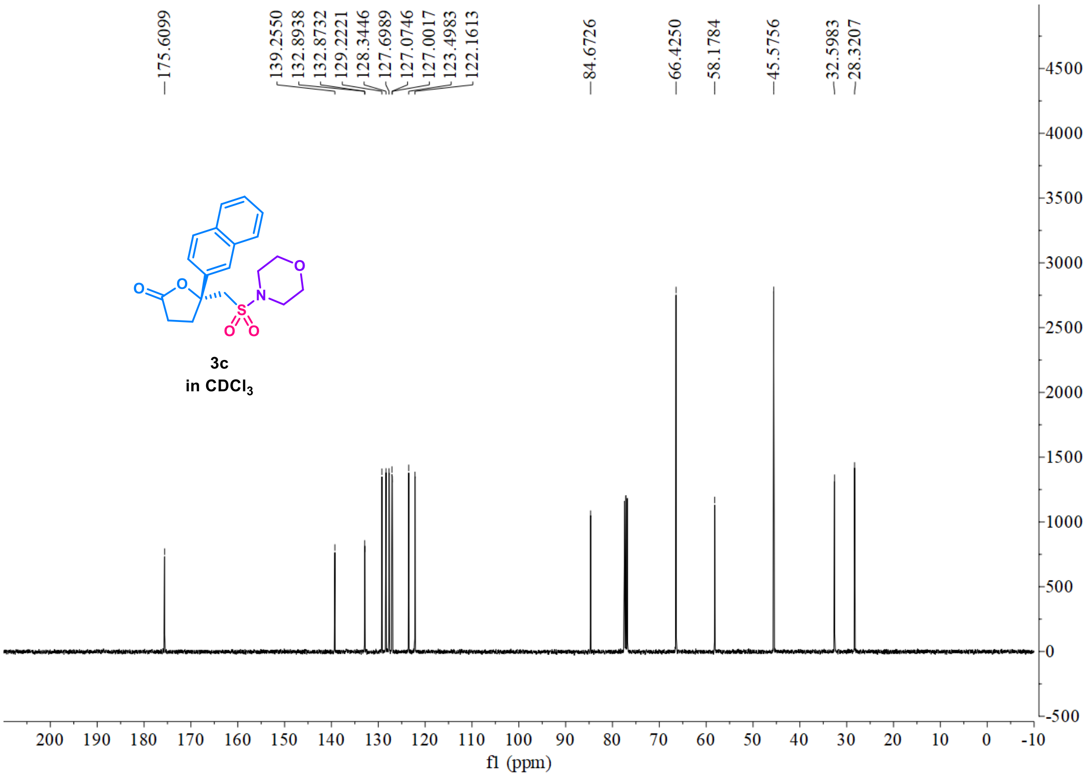
175.6099
139.2550
132.8938
132.8732
129.2221
128.3446
127.6989
127.0746
127.0017
123.4983
122.1613
84.6726
66.4250
58.1784
45.5756
32.5983
28.3207
3c
in CDCl$_3$
f1 (ppm)

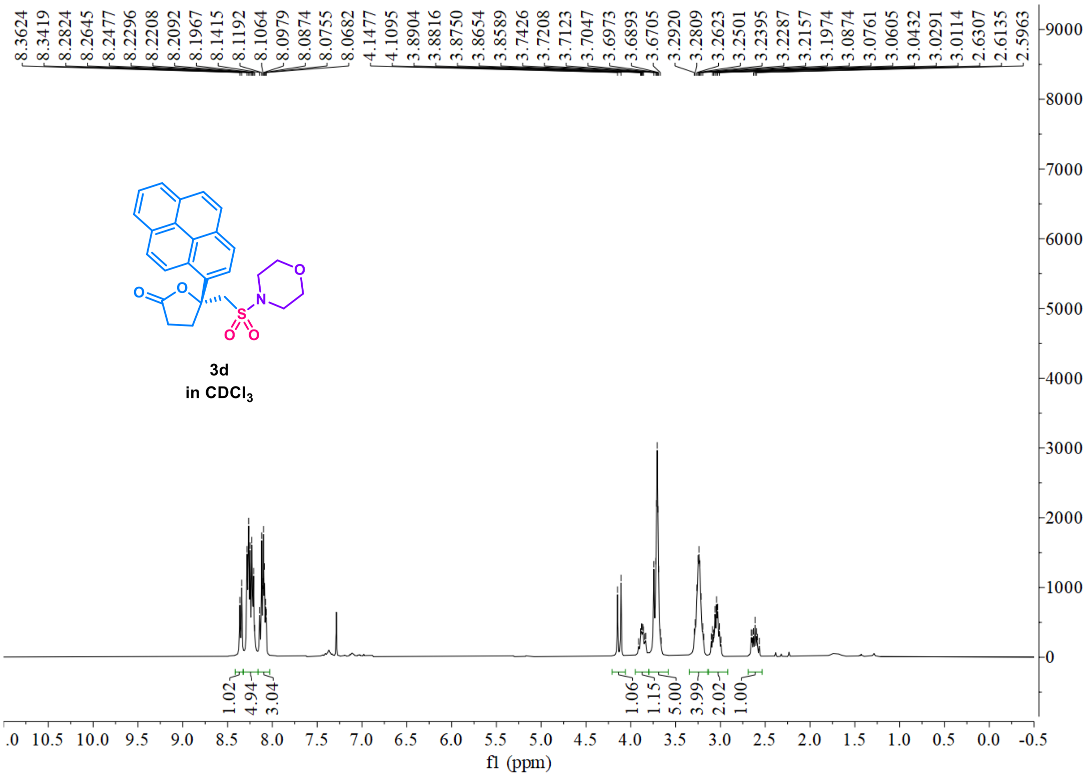

3d
in CDCl3
f1 (ppm)

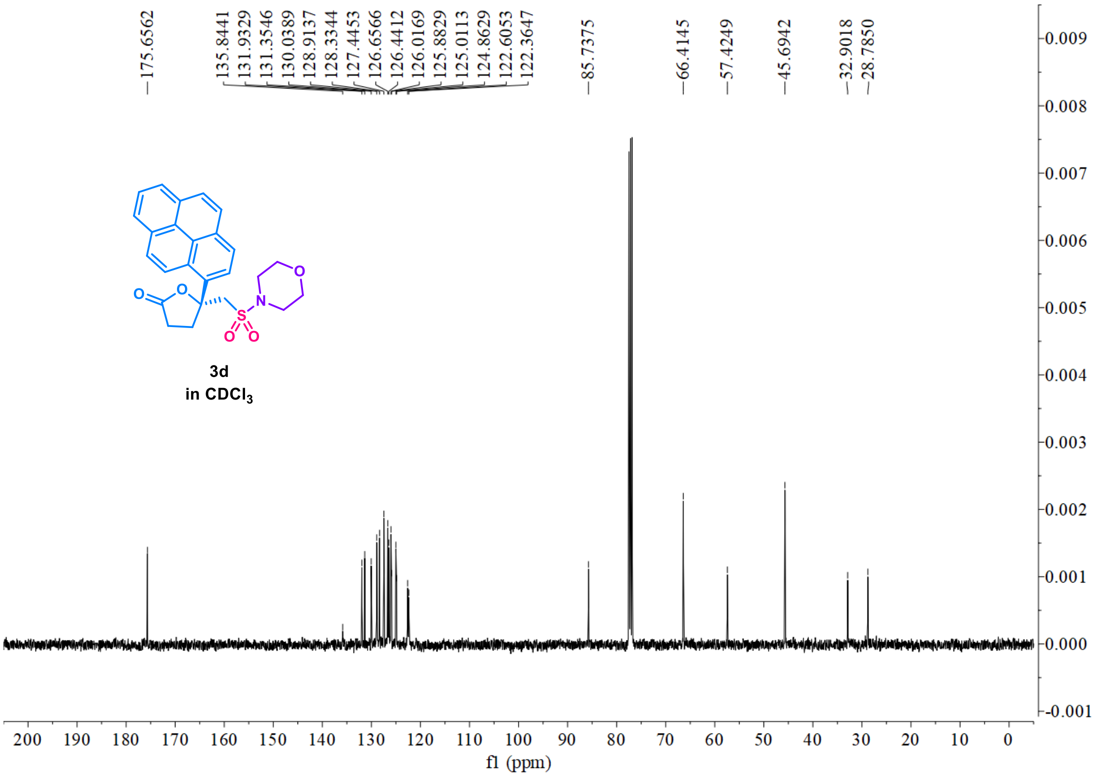

3d
in CDCl3
f1 (ppm)

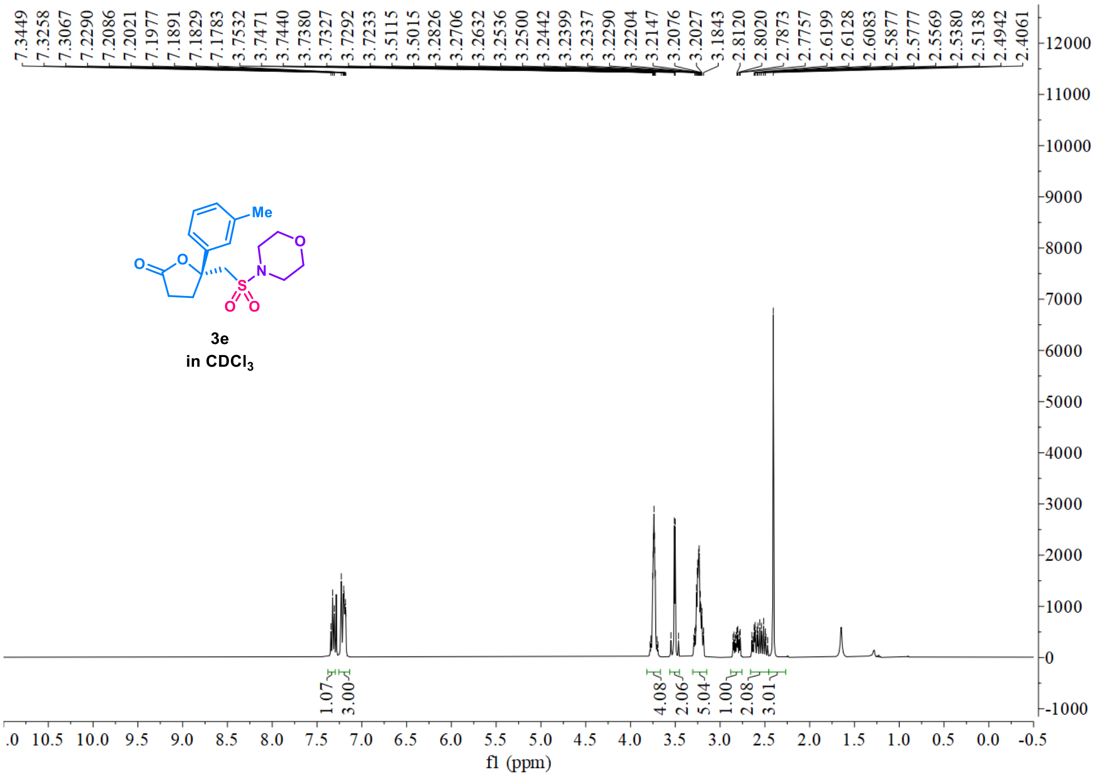

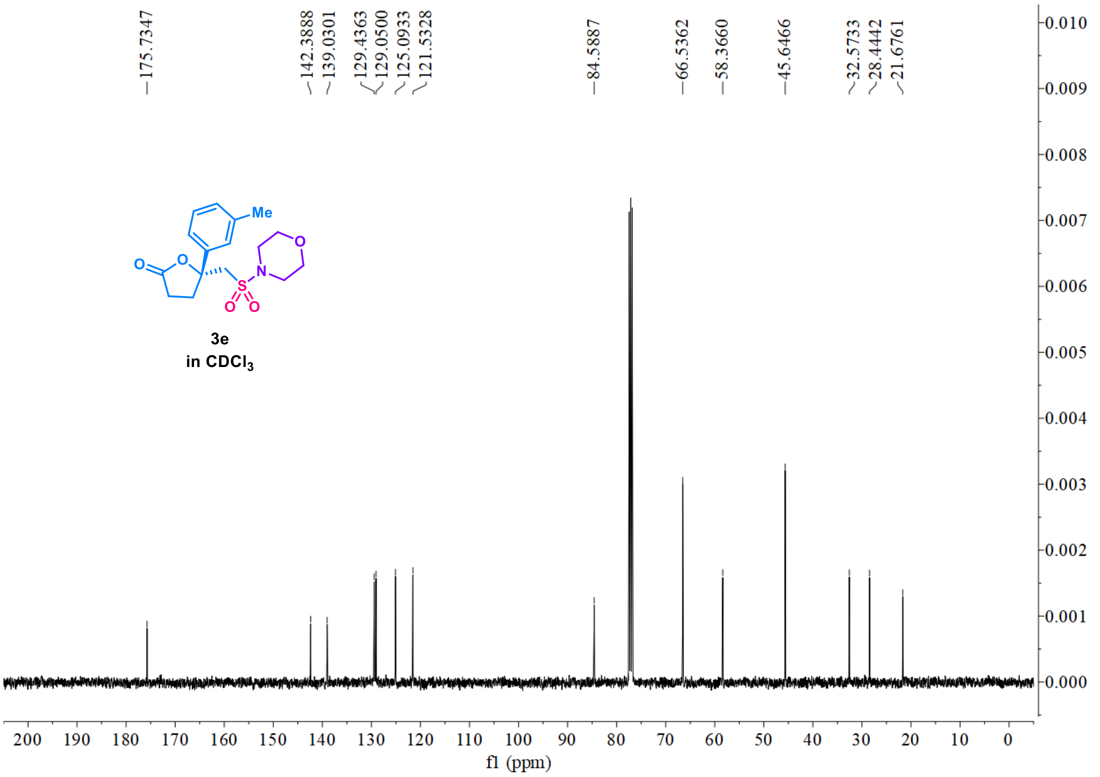

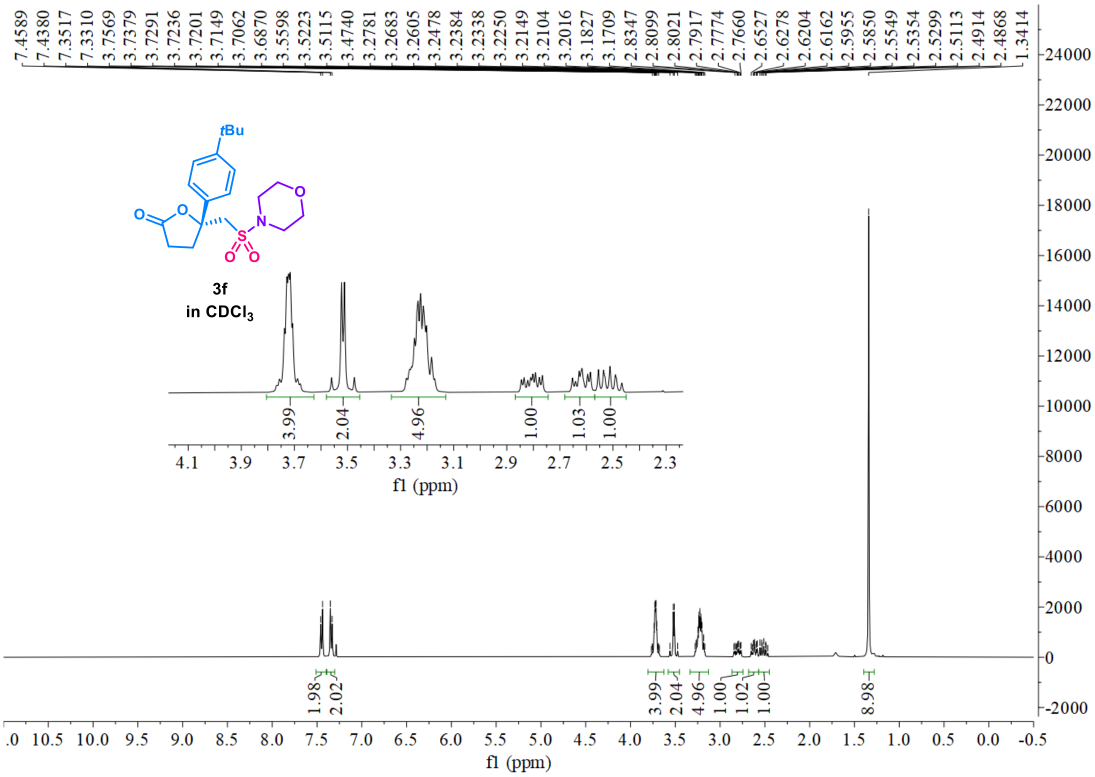

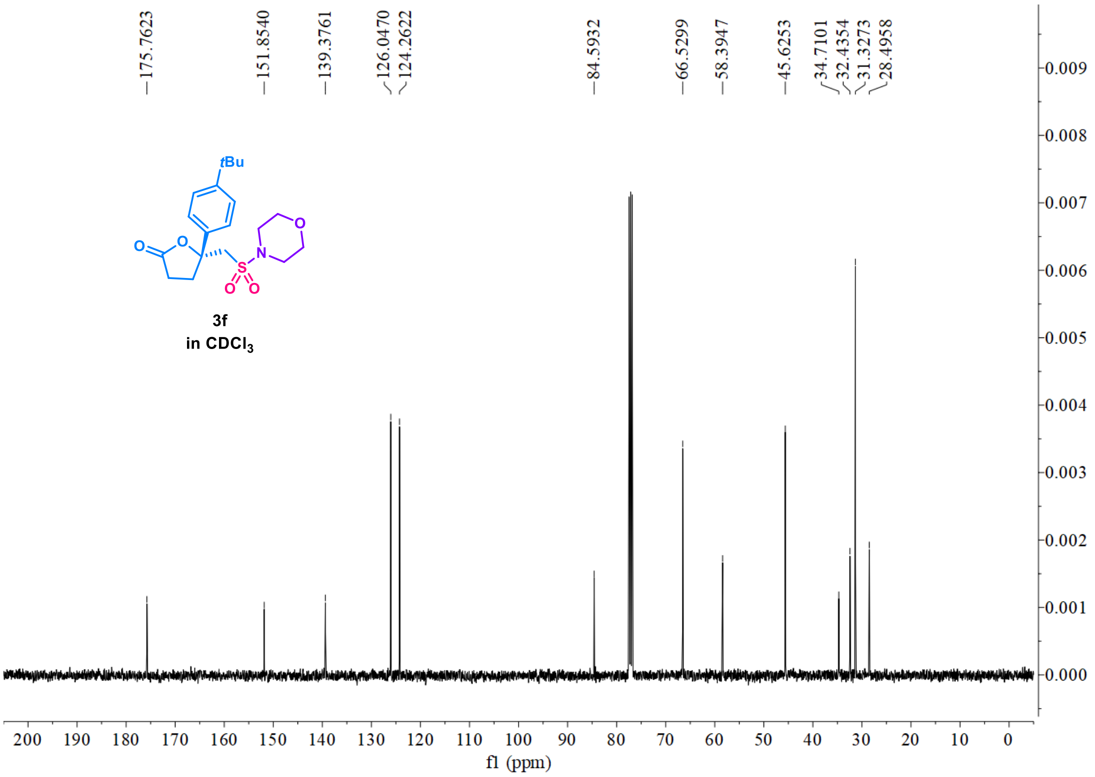

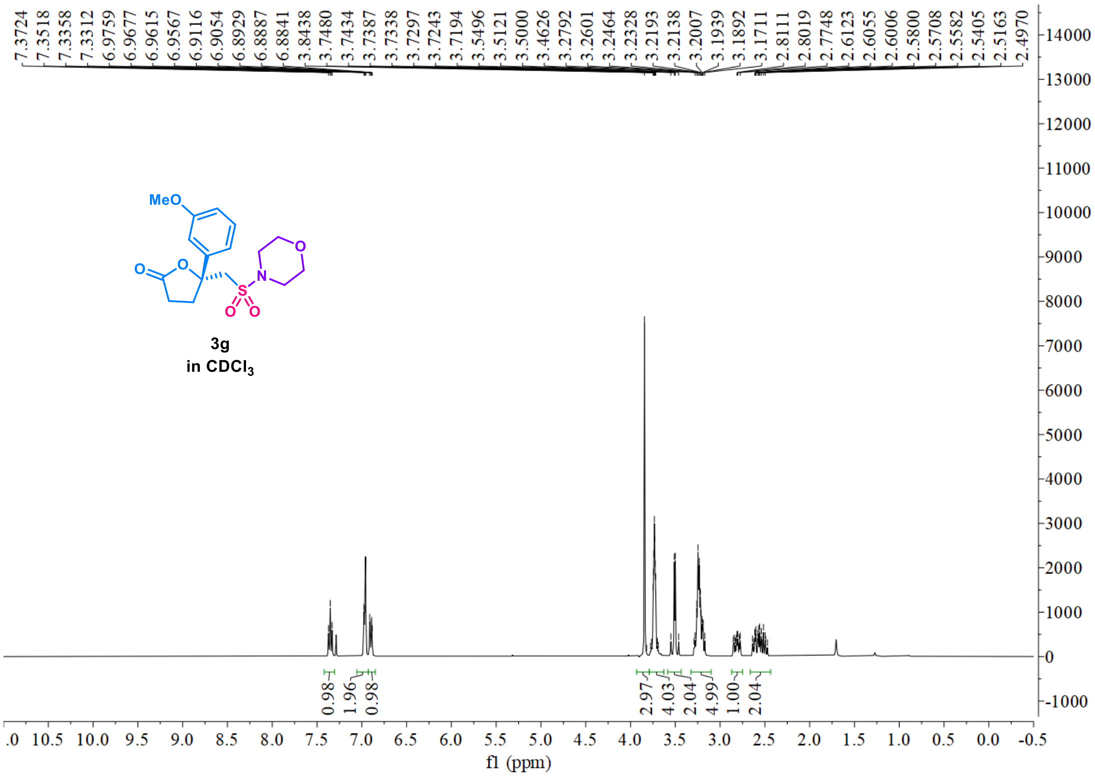
7.3724 7.3518 7.3358 7.3312 6.9759 6.9677 6.9615 6.9567 6.9116 6.9054 6.8929 6.8887 6.8841 3.8438 3.7480 3.7434 3.7387 3.7338 3.7297 3.7243 3.7194 3.5496 3.5121 3.5000 3.4626 3.2792 3.2601 3.2464 3.2328 3.2193 3.2138 3.2007 3.1939 3.1892 3.1711 2.8111 2.8019 2.7748 2.6123 2.6055 2.6006 2.5800 2.5708 2.5582 2.5405 2.5163 2.4970
MeO
3g
in CDCl$_3$
0.98 1.96 0.98 2.97 4.03 2.04 4.99 1.00 2.04
f1 (ppm)

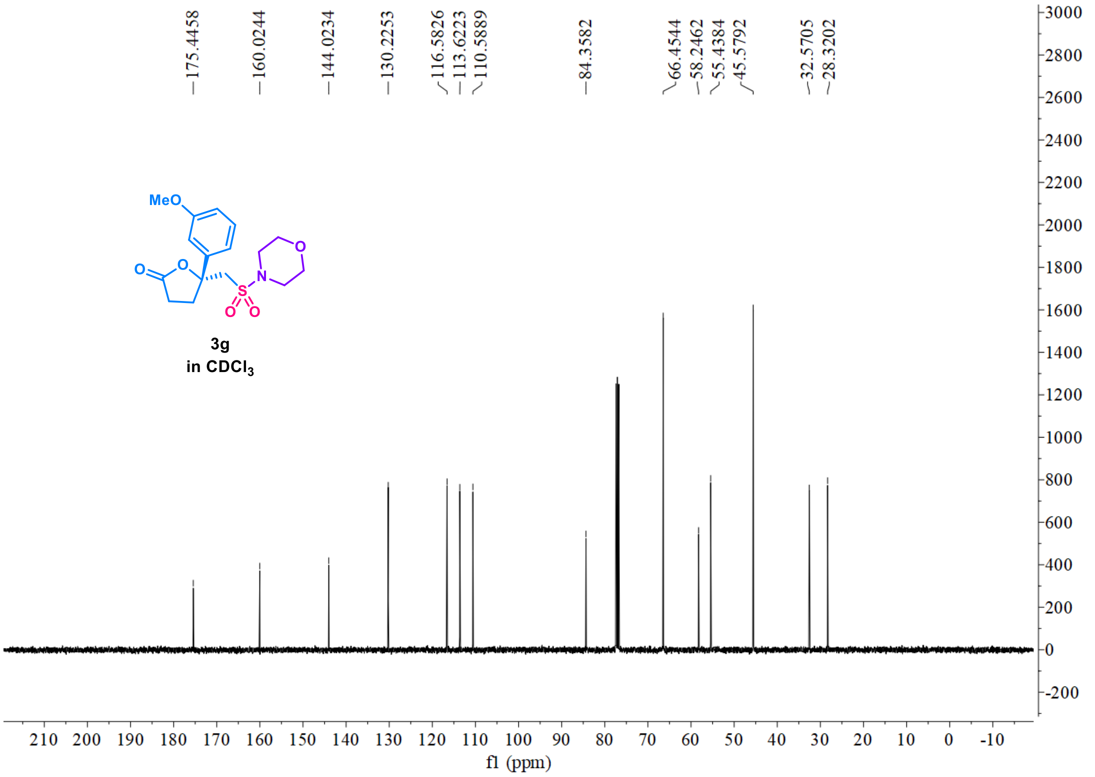
175.4458 160.0244 144.0234 130.2253 116.5826 113.6223 110.5889 84.3582 66.4544 58.2462 55.4384 45.5792 32.5705 28.3202
MeO
3g
in CDCl$_3$
f1 (ppm)

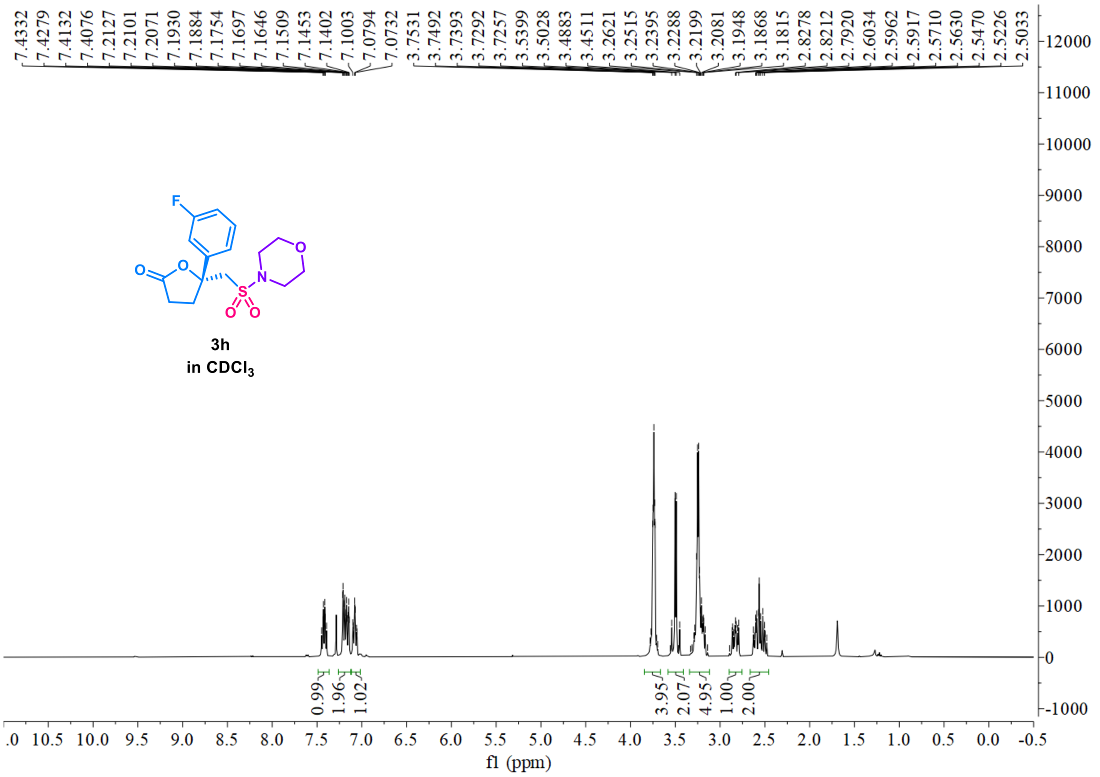
7.4332
7.4279
7.4132
7.4076
7.2127
7.2101
7.2071
7.1930
7.1884
7.1754
7.1697
7.1646
7.1509
7.1453
7.1402
7.1003
7.0794
7.0732
3.7531
3.7492
3.7393
3.7292
3.7257
3.5399
3.5028
3.4883
3.4511
3.2621
3.2515
3.2395
3.2288
3.2199
3.2081
3.1948
3.1868
3.1815
2.8278
2.8212
2.7920
2.6034
2.5962
2.5917
2.5710
2.5630
2.5470
2.5226
2.5033
3h
in CDCl3
0.99
1.96
1.02
3.95
2.07
4.95
1.00
2.00
f1 (ppm)

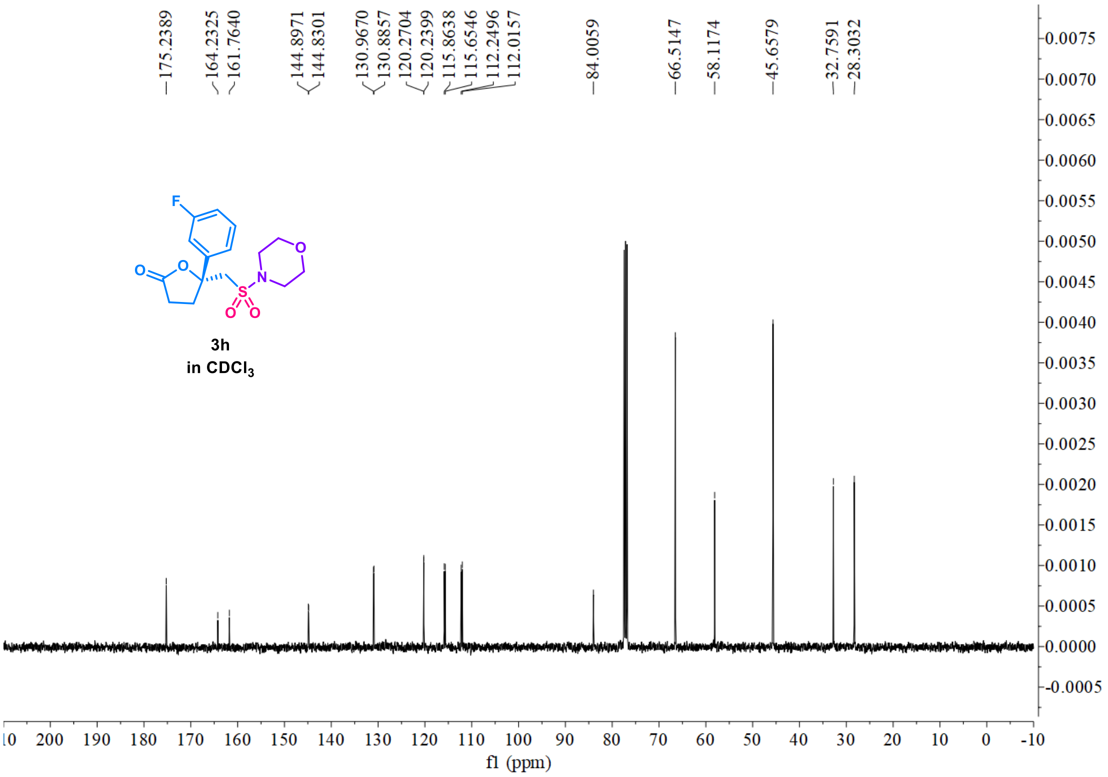
175.2389
164.2325
161.7640
144.8971
144.8301
130.9670
130.8857
120.2704
120.2399
115.8638
115.6546
112.2496
112.0157
84.0059
66.5147
58.1174
45.6579
32.7591
28.3032
3h
in CDCl3
f1 (ppm)

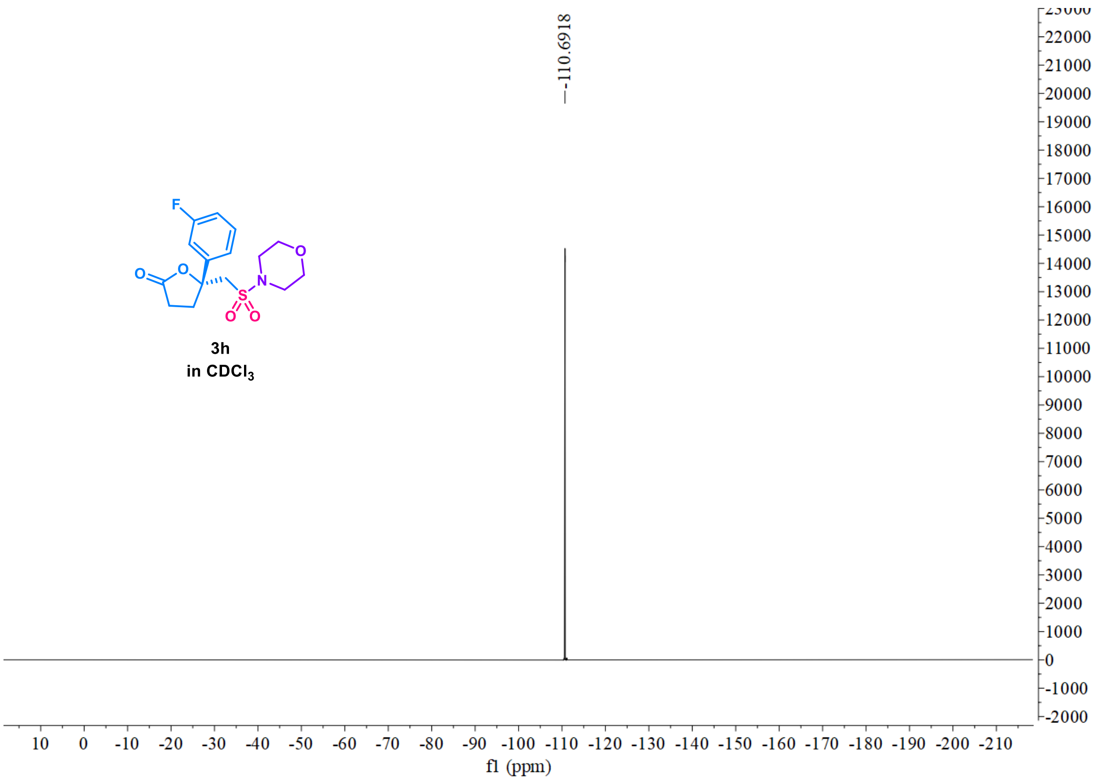
-110.6918
3h
in CDCl3
f1 (ppm)

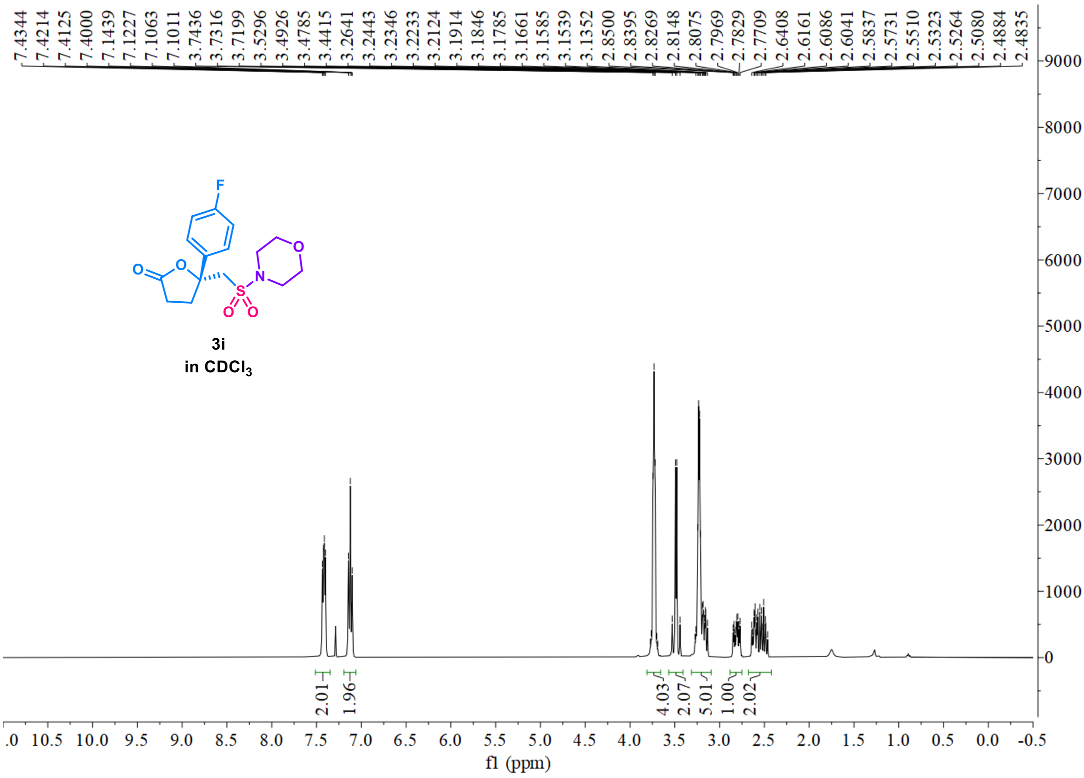
3i
in CDCl3
f1 (ppm)

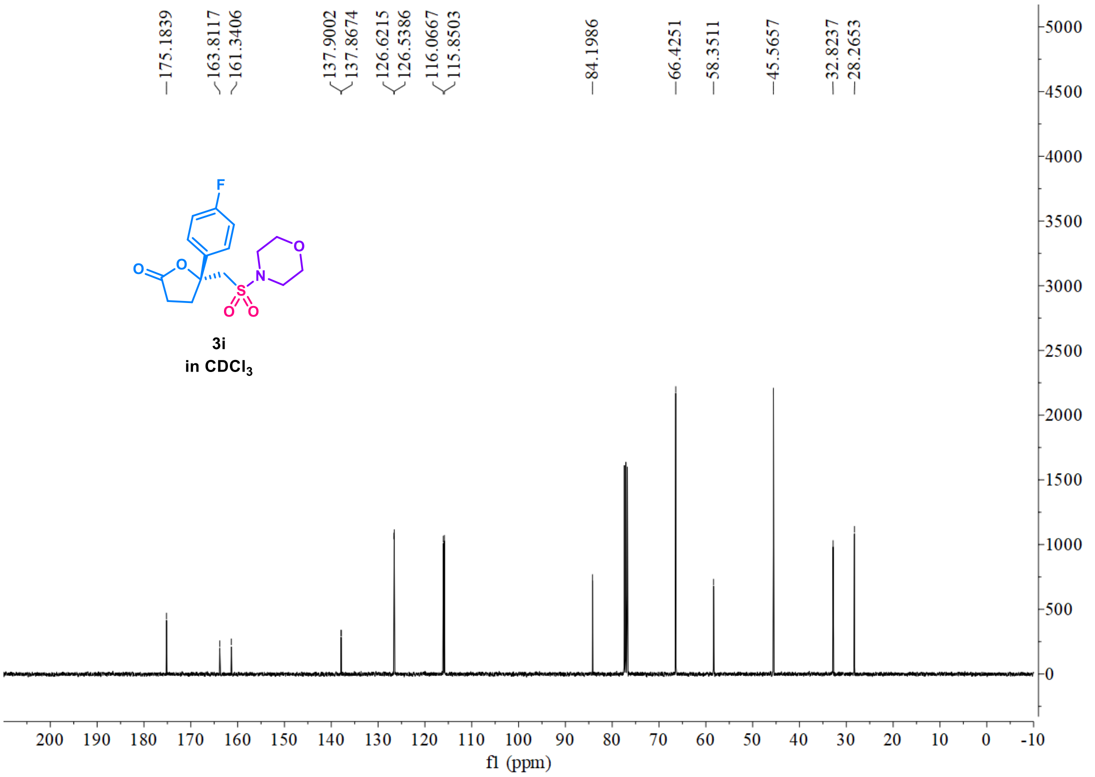
175.1839
163.8117
161.3406
137.9002
137.8674
126.6215
126.5386
116.0667
115.8503
84.1986
66.4251
58.3511
45.5657
32.8237
28.2653
F
O
O
O
N
S
O
O
3i
in CDCl3
f1 (ppm)

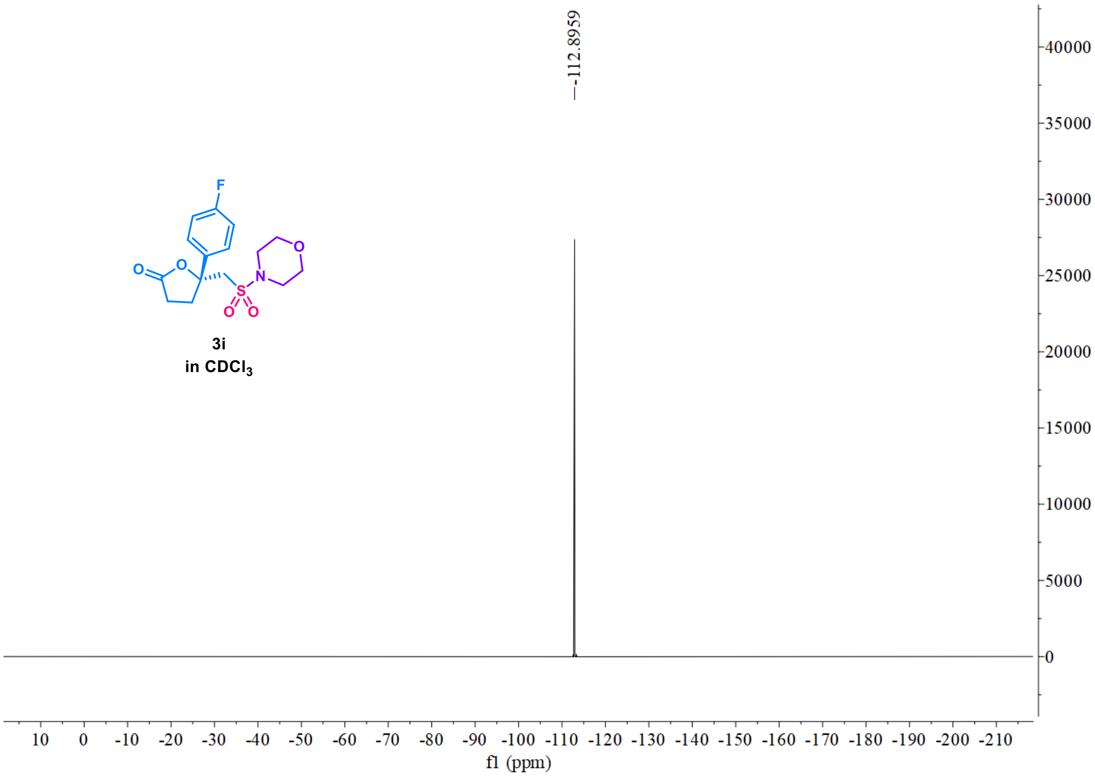
-112.8959
F
O
O
O
N
S
O
O
3i
in CDCl3
f1 (ppm)

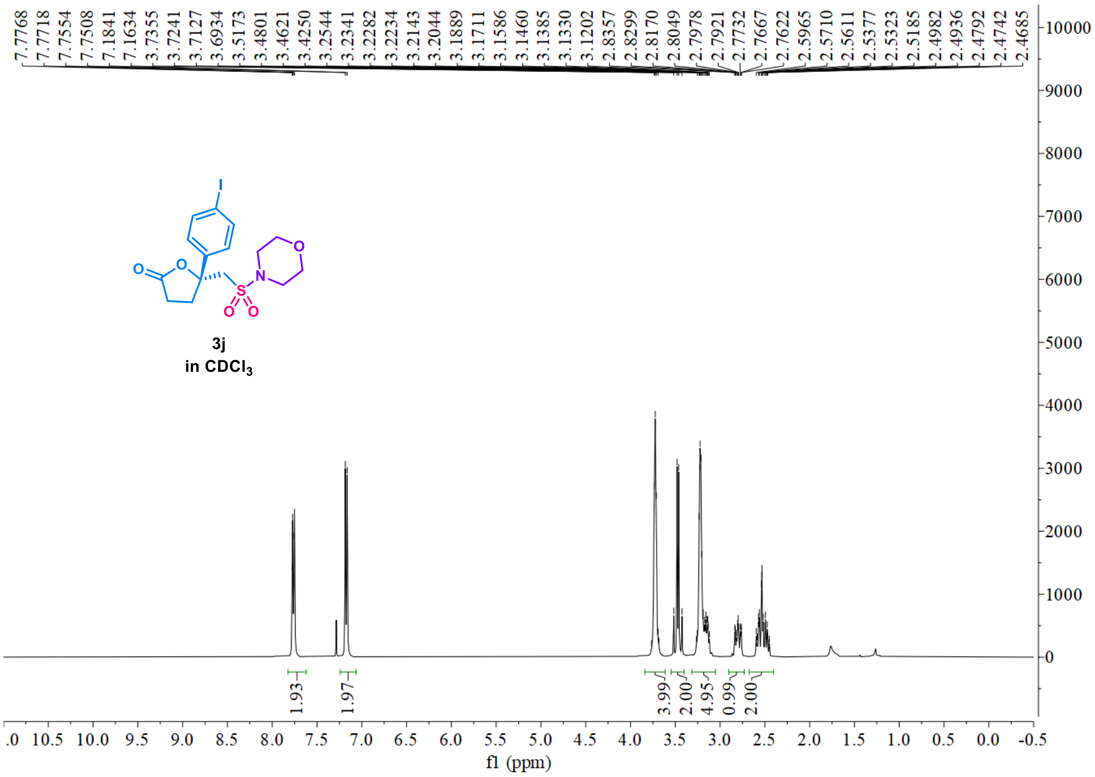

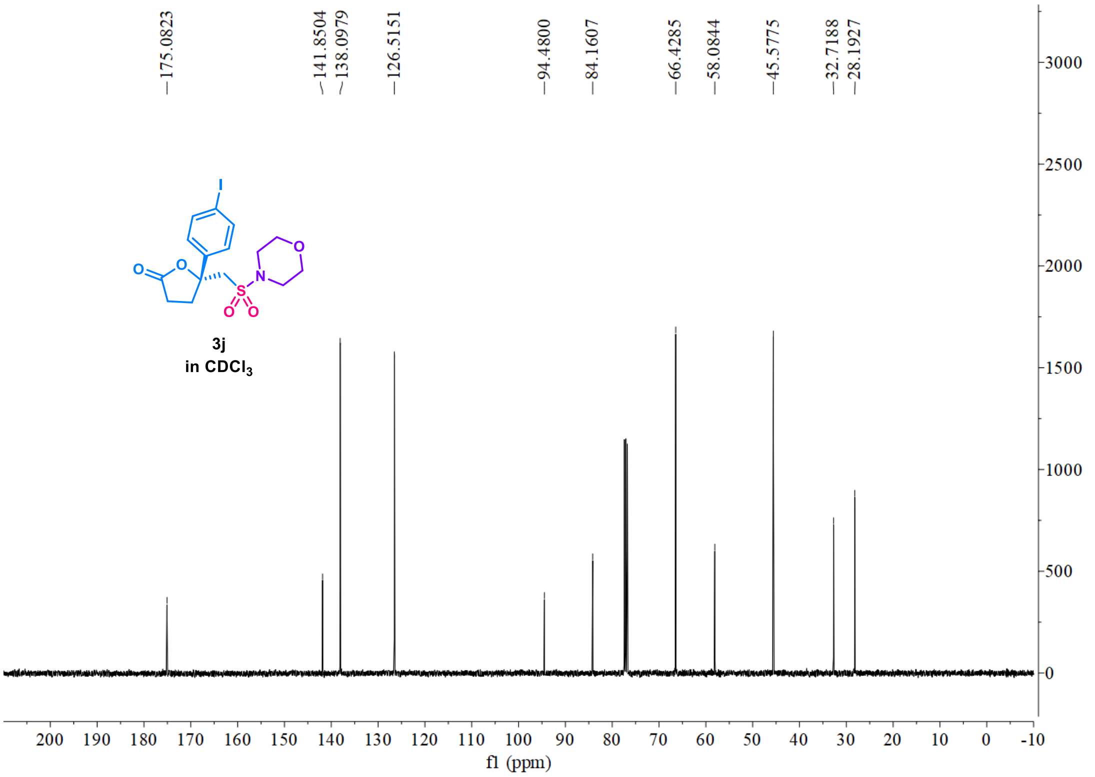

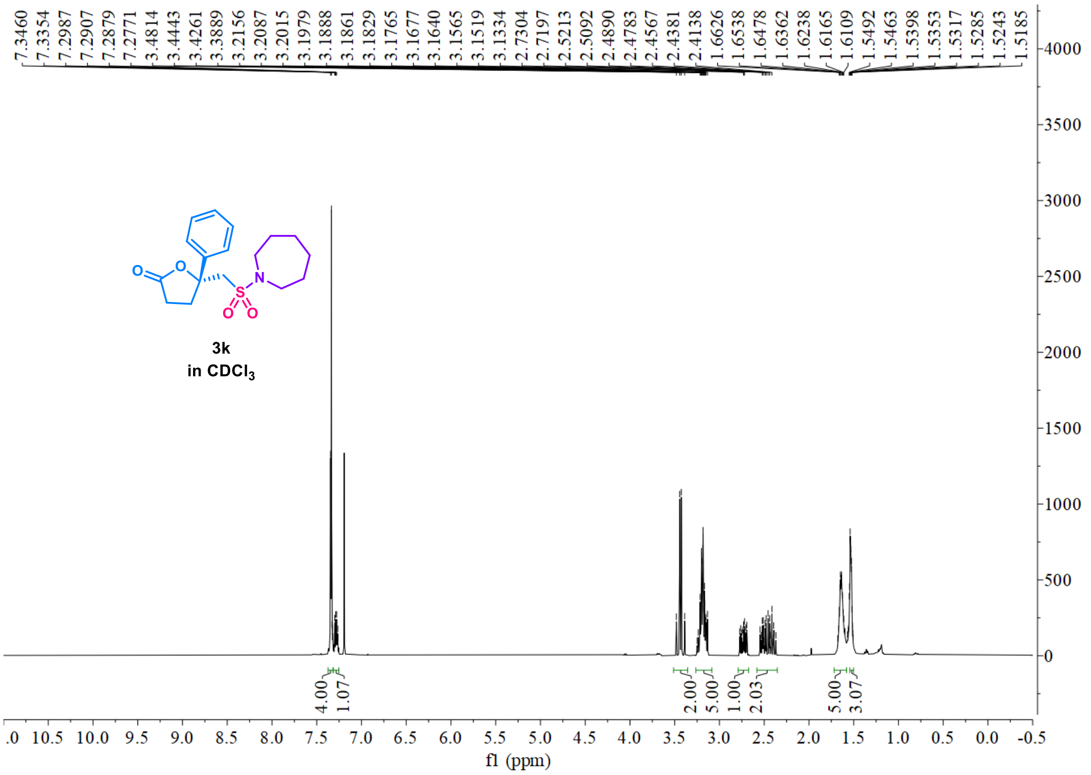
3k
in CDCl3
f1 (ppm)

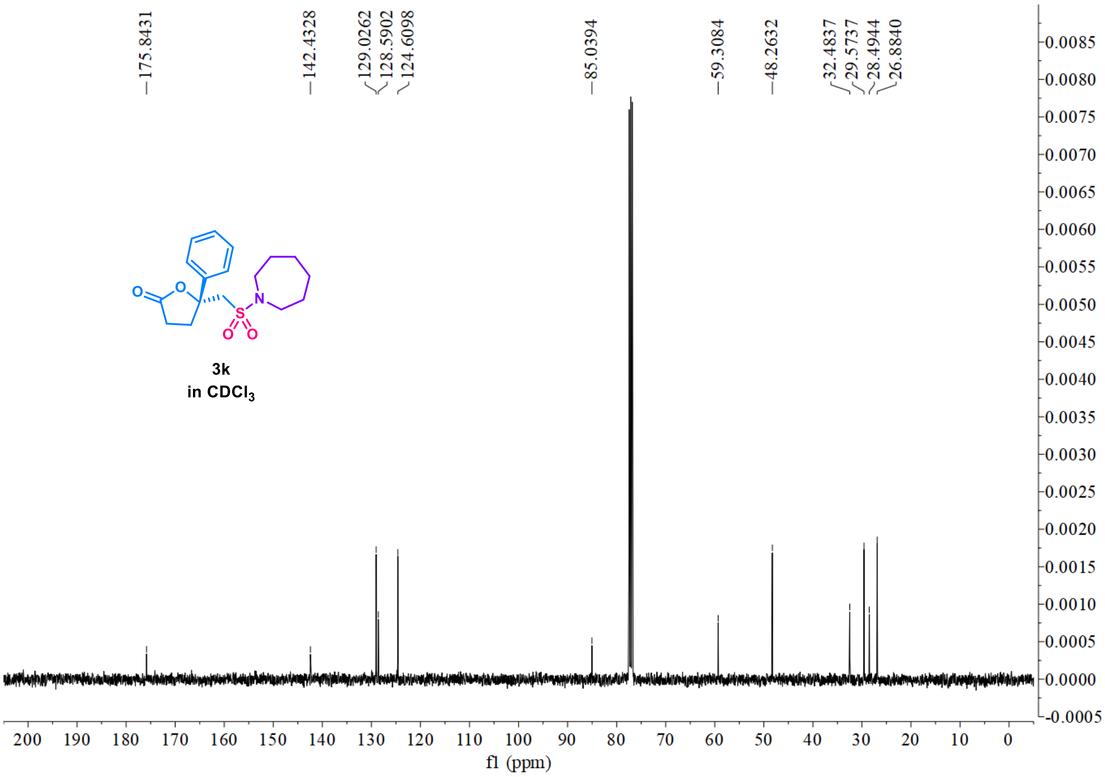
3k
in CDCl3
f1 (ppm)

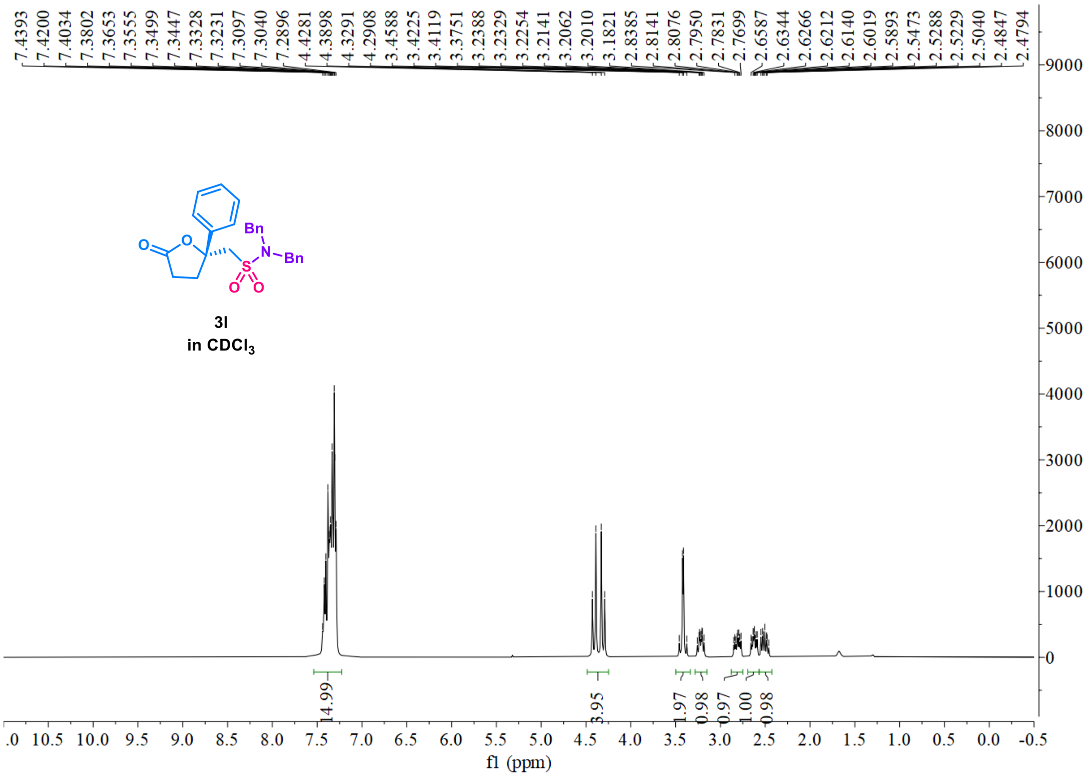
7.4393 7.4200 7.4034 7.3802 7.3653 7.3555 7.3499 7.3447 7.3328 7.3231 7.3097 7.3040 7.2896 4.4281 4.3898 4.3291 4.2908 3.4588 3.4225 3.4119 3.3751 3.2388 3.2329 3.2254 3.2141 3.2062 3.2010 3.1821 2.8385 2.8141 2.8076 2.7950 2.7831 2.7699 2.6587 2.6344 2.6266 2.6212 2.6140 2.6019 2.5893 2.5473 2.5288 2.5229 2.5040 2.4847 2.4794
Bn
N
Bn
O
O
S
O
O
3l
in CDCl3
14.99
3.95
1.97
0.98
0.97
1.00
0.98
f1 (ppm)

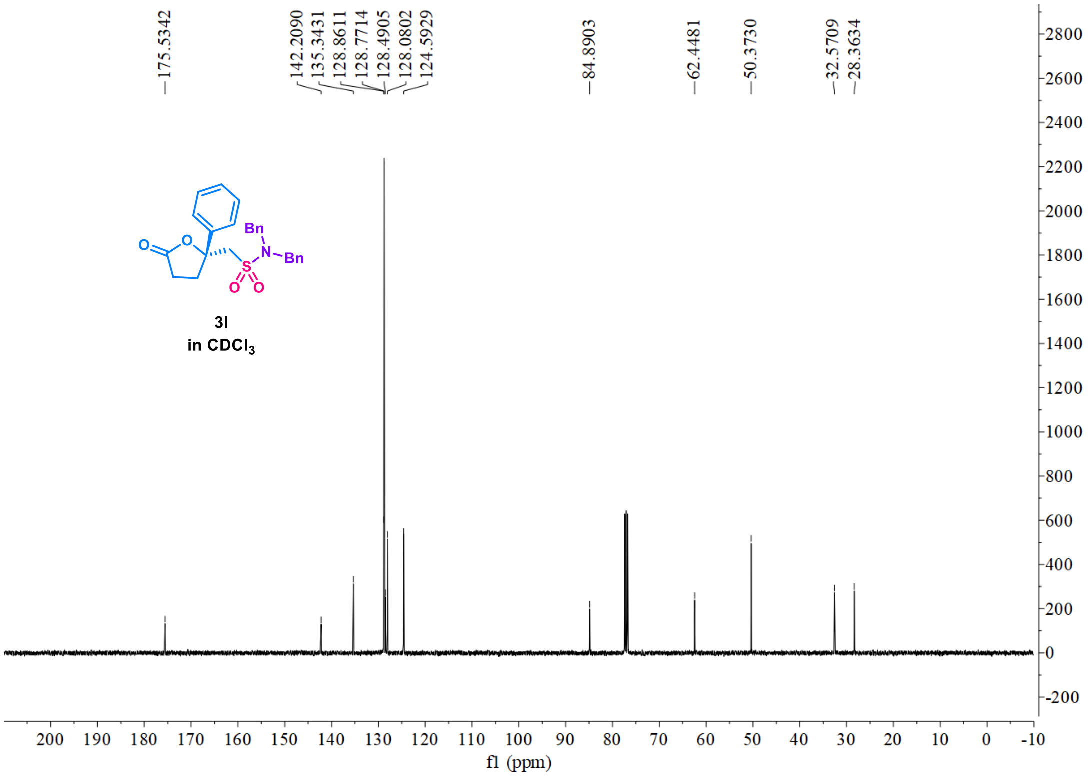
175.5342
142.2090 135.3431 128.8611 128.7714 128.4905 128.0802 124.5929
84.8903
62.4481
50.3730
32.5709 28.3634
Bn
N
Bn
O
O
S
O
O
3l
in CDCl3
f1 (ppm)

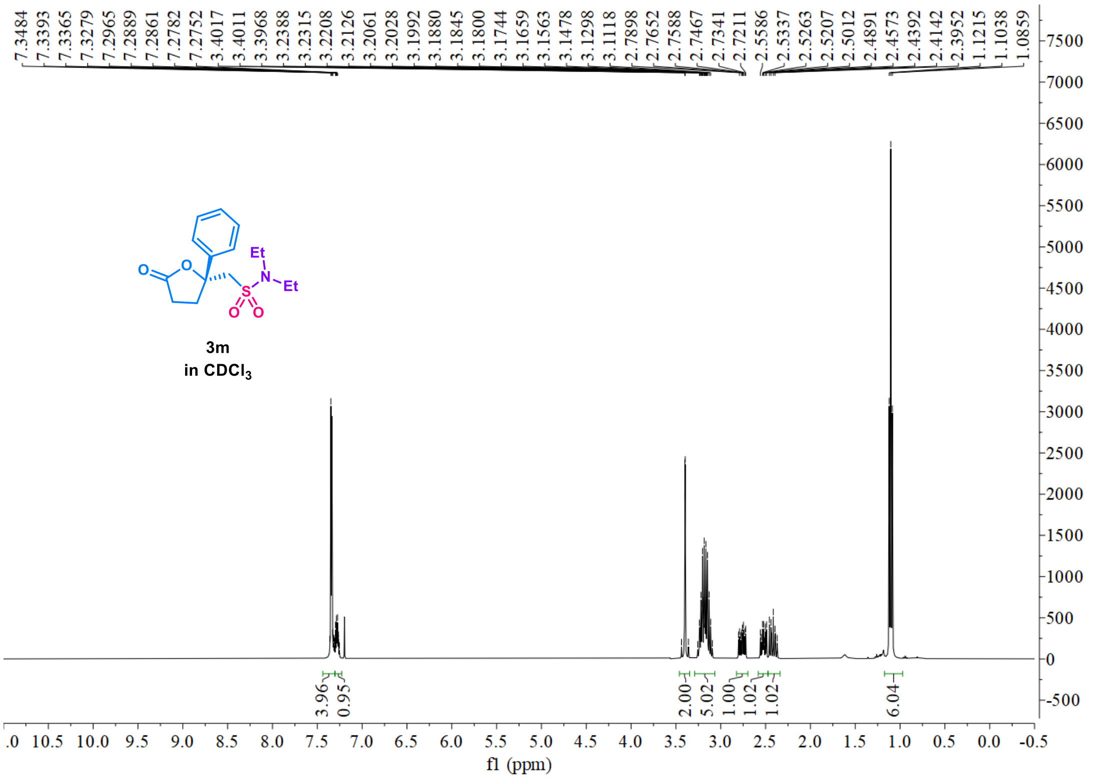
7.3484 7.3393 7.3365 7.3279 7.2965 7.2889 7.2861 7.2782 7.2752 3.4017 3.4011 3.3968 3.2388 3.2315 3.2208 3.2126 3.2061 3.2028 3.1992 3.1880 3.1845 3.1800 3.1744 3.1659 3.1563 3.1478 3.1298 3.1118 2.7898 2.7652 2.7588 2.7467 2.7341 2.7211 2.5586 2.5337 2.5263 2.5207 2.5012 2.4891 2.4573 2.4392 2.4142 2.3952 1.1215 1.1038 1.0859
Et
N
Et
O
O
S
O
O
3m
in $CDCl_3$
3.96
0.95
2.00
5.02
1.00
1.02
1.02
6.04
f1 (ppm)

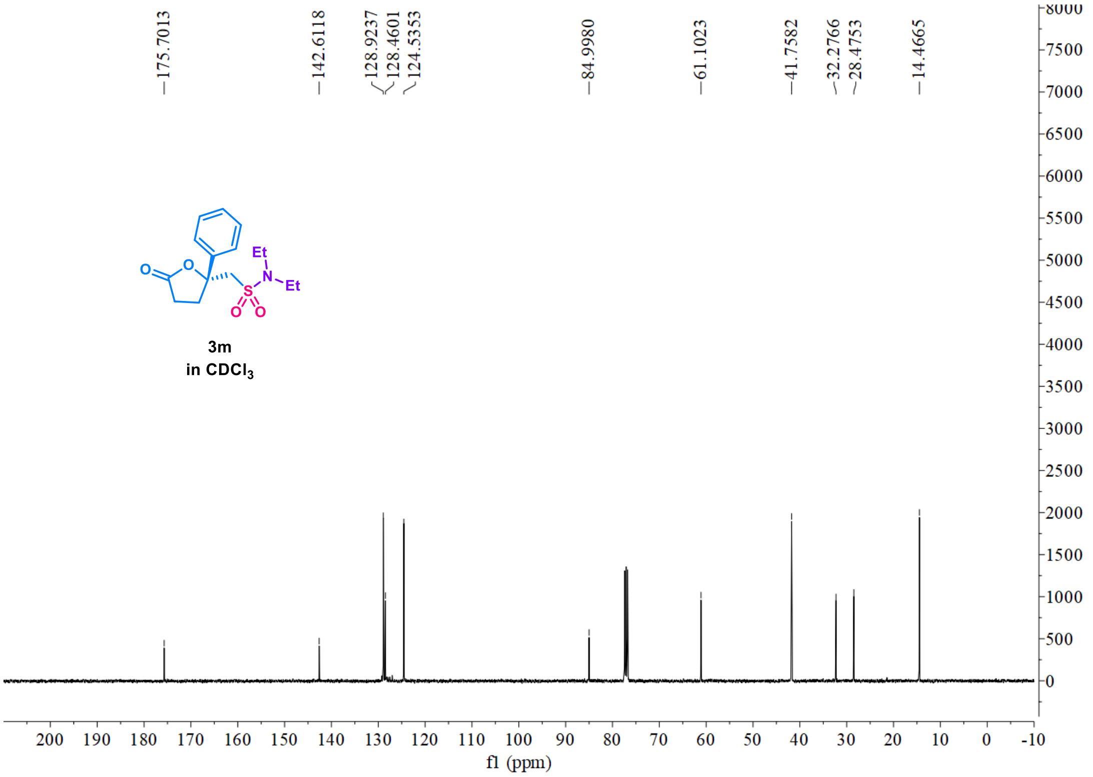
175.7013
142.6118
128.9237
128.4601
124.5353
84.9980
61.1023
41.7582
32.2766
28.4753
14.4665
Et
N
Et
O
O
S
O
O
3m
in $CDCl_3$
f1 (ppm)

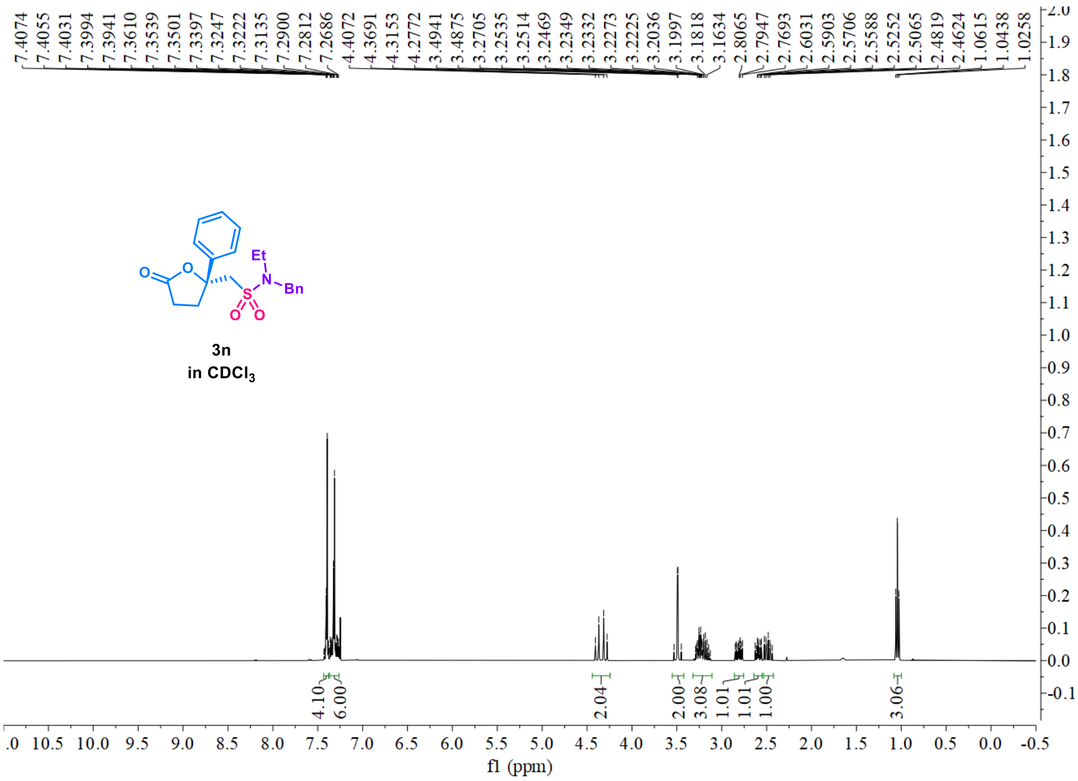

3n
in $CDCl_3$
7.4074
7.4055
7.4031
7.3994
7.3941
7.3610
7.3539
7.3501
7.3397
7.3247
7.3222
7.3135
7.2900
7.2812
7.2686
4.4072
4.3691
4.3153
4.2772
3.4941
3.4875
3.2705
3.2535
3.2514
3.2469
3.2349
3.2332
3.2273
3.2225
3.2036
3.1997
3.1818
3.1634
2.8065
2.7947
2.7693
2.6031
2.5903
2.5706
2.5588
2.5252
2.5065
2.4819
2.4624
1.0615
1.0438
1.0258
4.10
6.00
2.04
2.00
3.08
1.01
1.01
1.00
3.06
f1 (ppm)

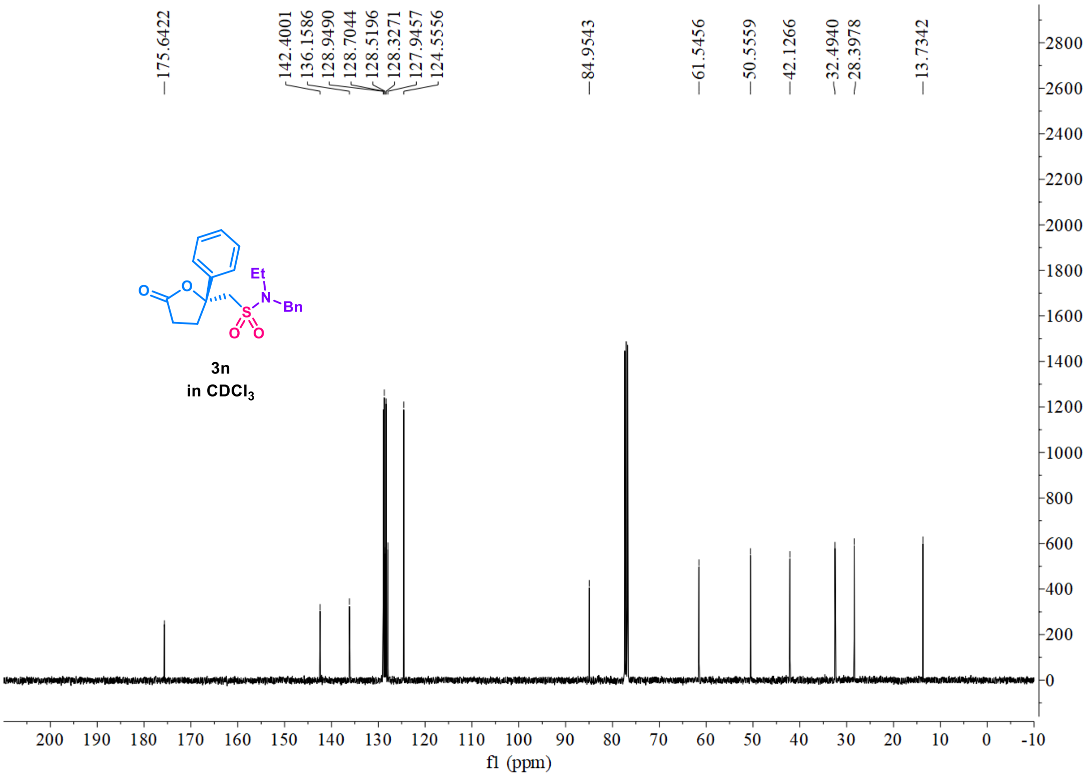

3n
in $CDCl_3$
175.6422
142.4001
136.1586
128.9490
128.7044
128.5196
128.3271
127.9457
124.5556
84.9543
61.5456
50.5559
42.1266
32.4940
28.3978
13.7342
f1 (ppm)

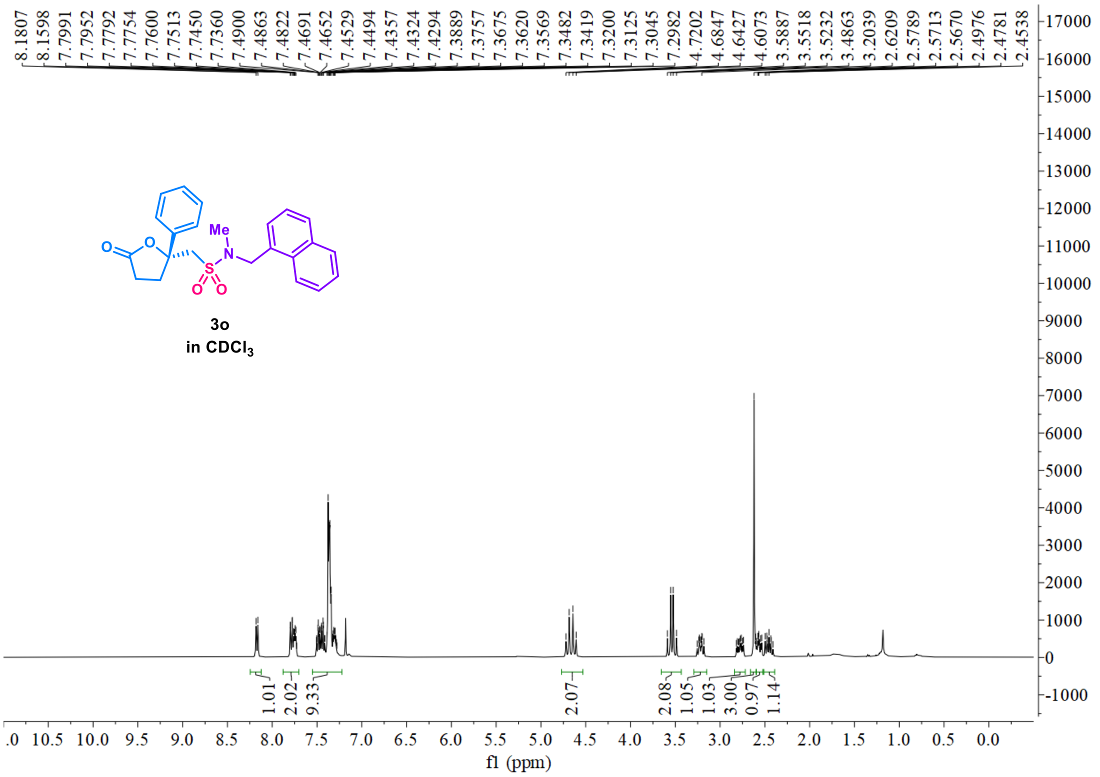
8.1807
8.1598
7.7991
7.7952
7.7792
7.7754
7.7600
7.7513
7.7450
7.7360
7.4900
7.4863
7.4822
7.4691
7.4652
7.4529
7.4494
7.4357
7.4324
7.4294
7.3889
7.3757
7.3675
7.3620
7.3569
7.3482
7.3419
7.3200
7.3125
7.3045
7.2982
4.7202
4.6847
4.6427
4.6073
3.5887
3.5518
3.5232
3.4863
3.2039
2.6209
2.5789
2.5713
2.5670
2.4976
2.4781
2.4538
Me
N
O
S
3o
in CDCl3
1.01
2.02
9.33
2.07
2.08
1.05
1.03
3.00
0.97
1.14
fl (ppm)

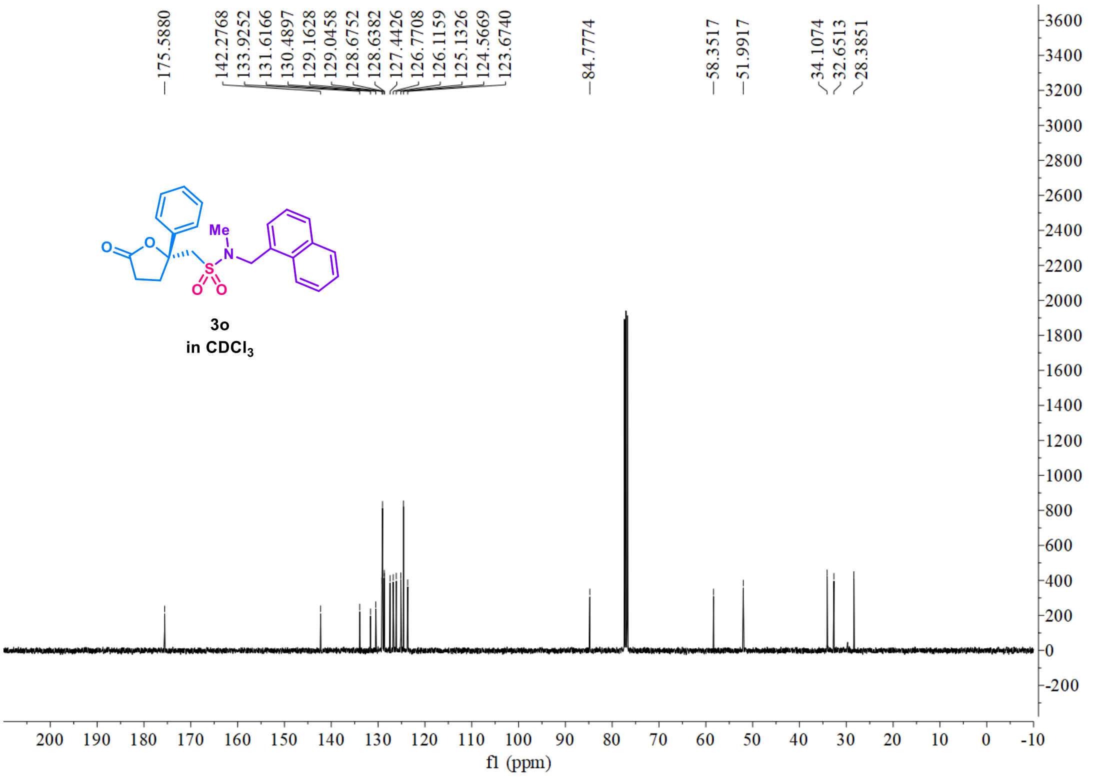
175.5880
142.2768
133.9252
131.6166
130.4897
129.1628
129.0458
128.6752
128.6382
127.4426
126.7708
126.1159
125.1326
124.5669
123.6740
84.7774
58.3517
51.9917
34.1074
32.6513
28.3851
Me
N
O
S
3o
in CDCl3
fl (ppm)

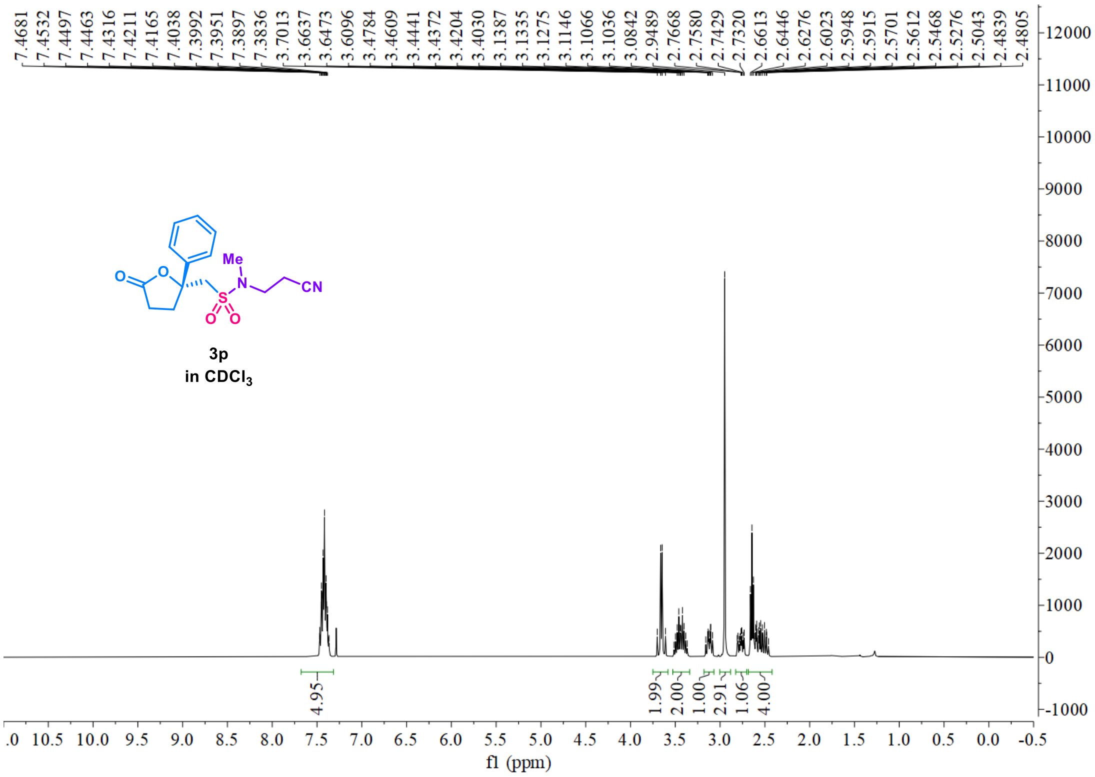
7.4681
7.4532
7.4497
7.4463
7.4316
7.4211
7.4165
7.4038
7.3992
7.3951
7.3897
7.3836
3.7013
3.6637
3.6473
3.6096
3.4784
3.4609
3.4441
3.4372
3.4204
3.4030
3.1387
3.1335
3.1275
3.1146
3.1066
3.1036
3.0842
2.9489
2.7668
2.7580
2.7429
2.7320
2.6613
2.6446
2.6276
2.6023
2.5948
2.5915
2.5701
2.5612
2.5468
2.5276
2.5043
2.4839
2.4805
3p
in CDCl3
4.95
1.99
2.00
1.00
2.91
1.06
4.00
f1 (ppm)

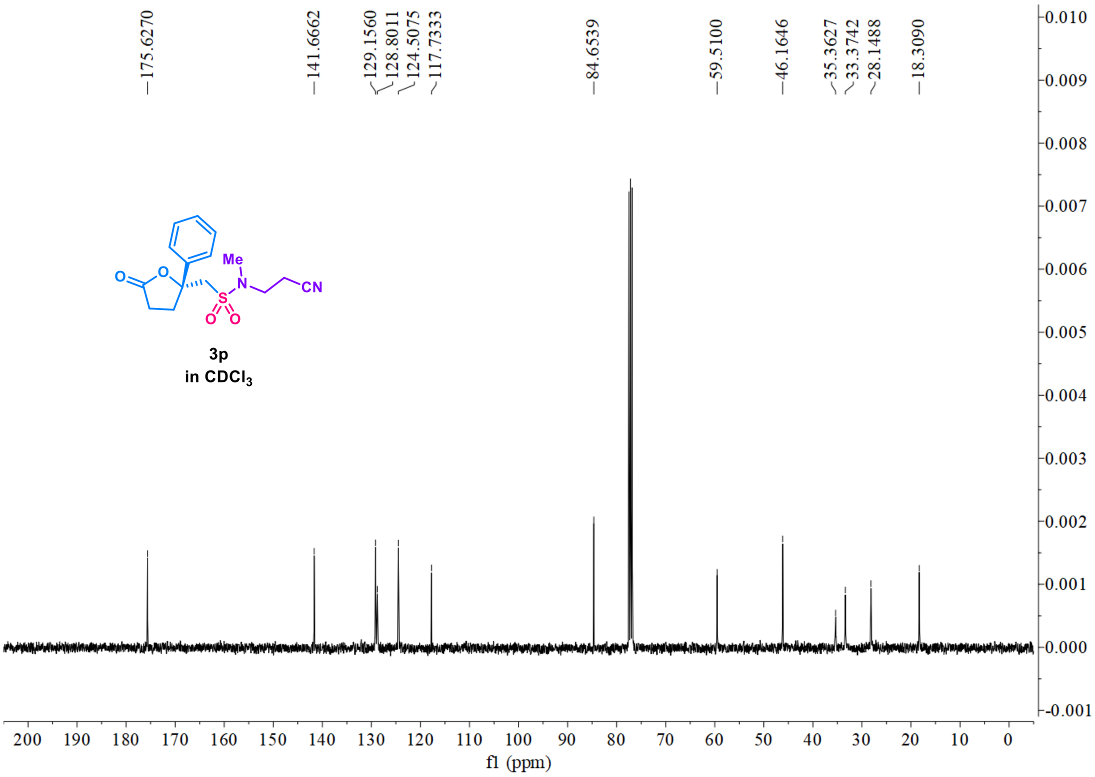
175.6270
141.6662
129.1560
128.8011
124.5075
117.7333
84.6539
59.5100
46.1646
35.3627
33.3742
28.1488
18.3090
3p
in CDCl3
f1 (ppm)

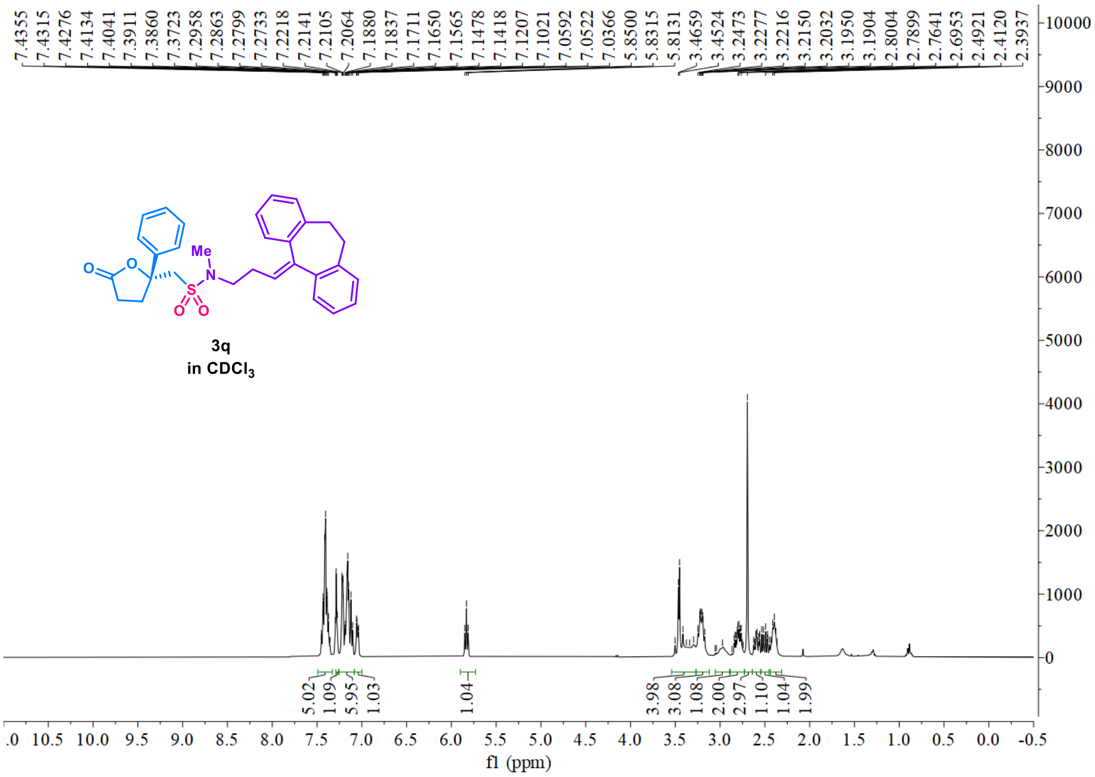
7.4355
7.4315
7.4276
7.4134
7.4041
7.3911
7.3860
7.3723
7.2958
7.2863
7.2799
7.2733
7.2218
7.2141
7.2105
7.2064
7.1880
7.1837
7.1711
7.1650
7.1565
7.1478
7.1418
7.1207
7.1021
7.0592
7.0522
7.0366
5.8500
5.8315
5.8131
3.4659
3.4524
3.2473
3.2277
3.2216
3.2150
3.2032
3.1950
3.1904
2.8004
2.7899
2.7641
2.6953
2.4921
2.4120
2.3937
Me
N
O
S
3q
in CDCl3
5.02
1.09
5.95
1.03
1.04
3.98
3.08
1.08
2.00
2.97
1.10
1.04
1.99
f1 (ppm)

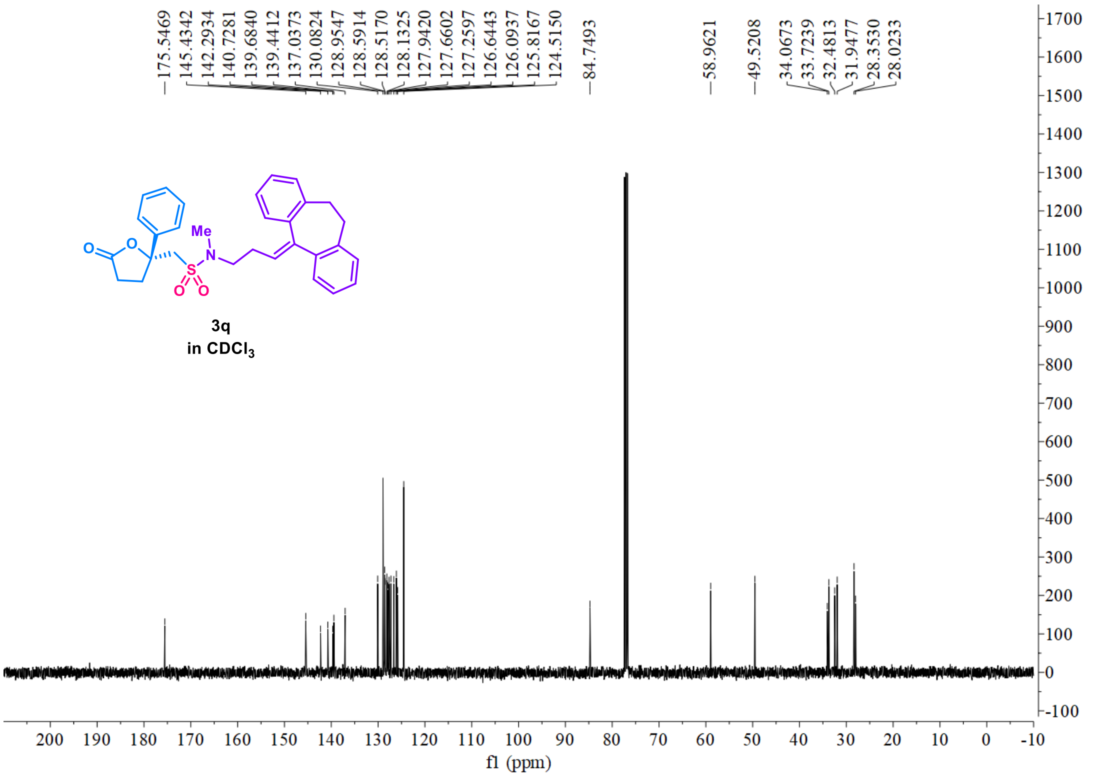
175.5469
145.4342
142.2934
140.7281
139.6840
139.4412
137.0373
130.0824
128.9547
128.5914
128.5170
128.1325
127.9420
127.6602
127.2597
126.6443
126.0937
125.8167
124.5150
84.7493
58.9621
49.5208
34.0673
33.7239
32.4813
31.9477
28.3530
28.0233
Me
N
O
S
3q
in CDCl3
f1 (ppm)

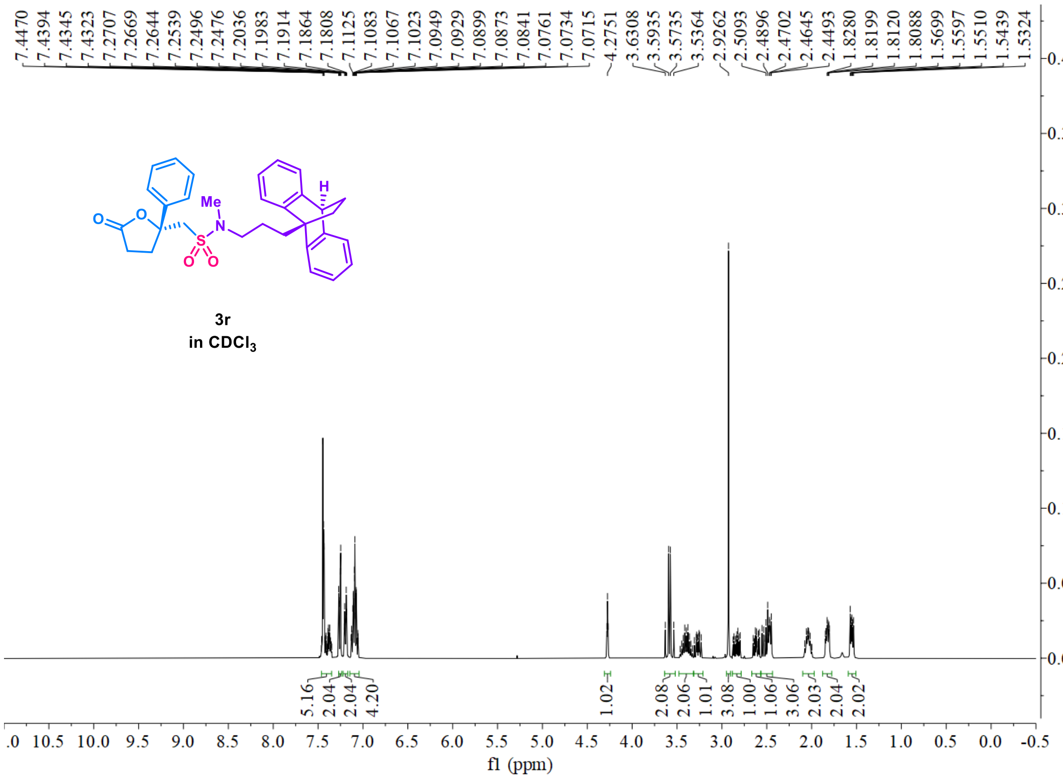
7.4470
7.4394
7.4345
7.4323
7.2707
7.2669
7.2644
7.2539
7.2496
7.2476
7.2036
7.1983
7.1914
7.1864
7.1808
7.1125
7.1083
7.1067
7.1023
7.0949
7.0929
7.0899
7.0873
7.0841
7.0761
7.0734
7.0715
4.2751
3.6308
3.5935
3.5735
3.5364
2.9262
2.5093
2.4896
2.4702
2.4645
2.4493
1.8280
1.8199
1.8120
1.8088
1.5699
1.5597
1.5510
1.5439
1.5324
Me
N
H
O
S
3r
in CDCl3
5.16
2.04
2.04
2.04
4.20
1.02
2.08
2.06
1.01
3.08
1.00
1.06
3.06
2.03
2.04
2.02
f1 (ppm)

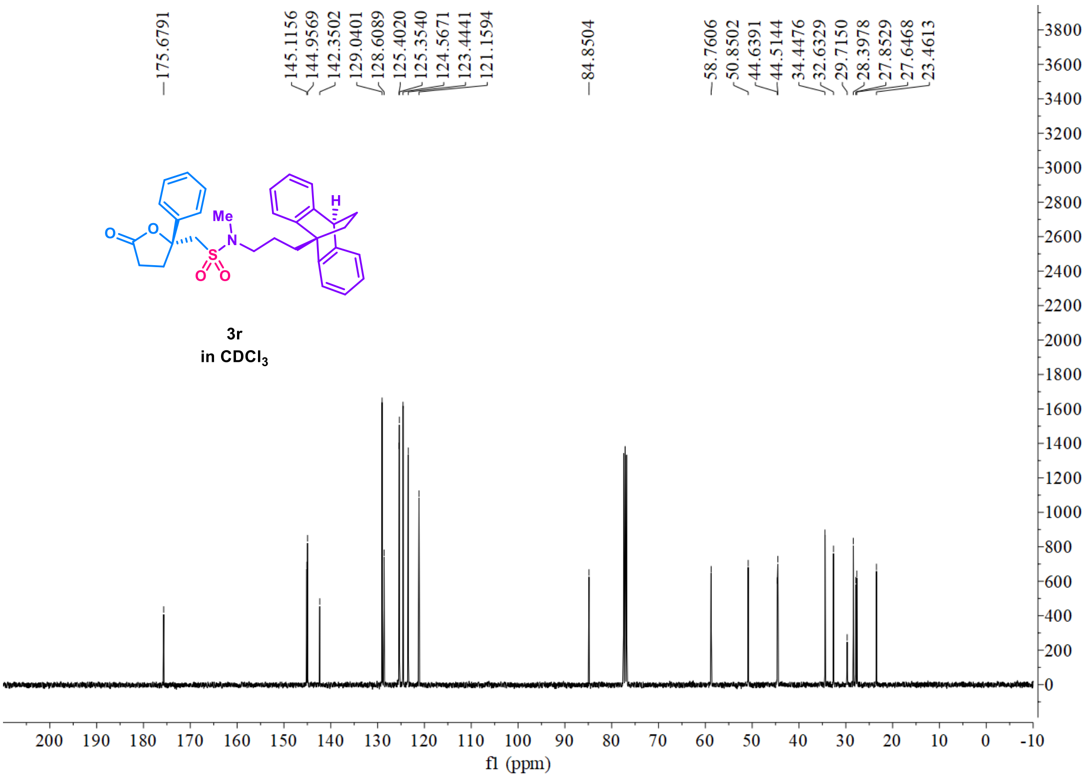
175.6791
145.1156
144.9569
142.3502
129.0401
128.6089
125.4020
125.3540
124.5671
123.4441
121.1594
84.8504
58.7606
50.8502
44.6391
44.5144
34.4476
32.6329
29.7150
28.3978
27.8529
27.6468
23.4613
Me
N
H
O
S
3r
in CDCl3
f1 (ppm)

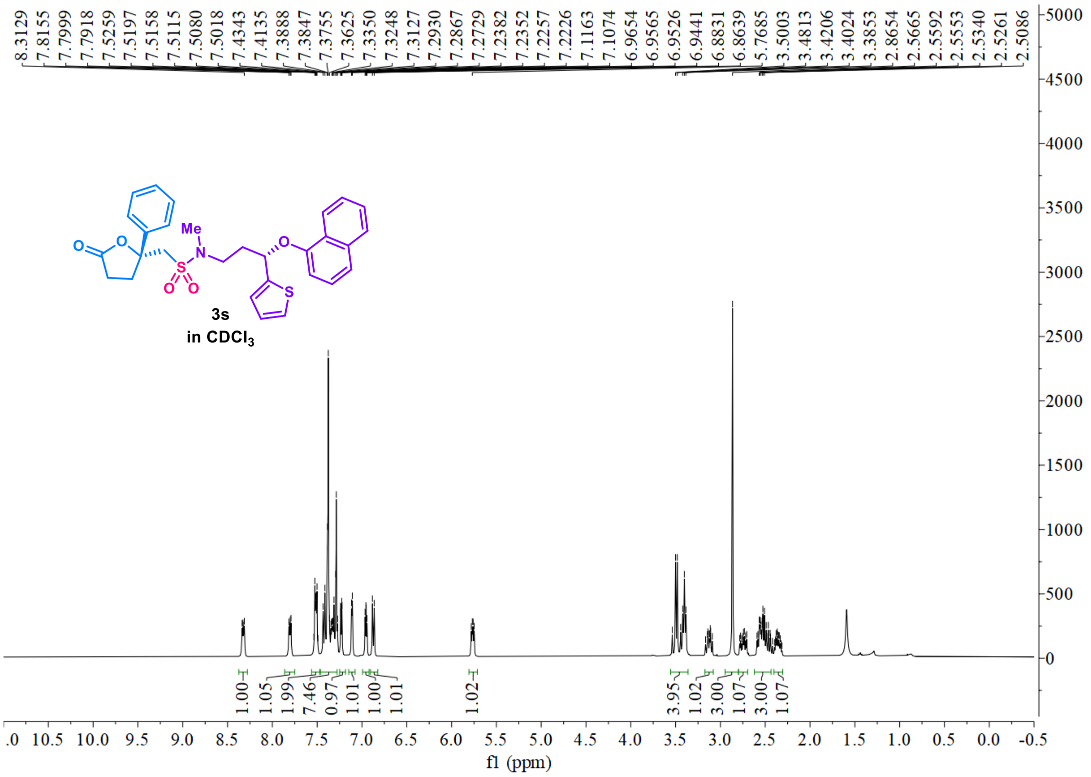

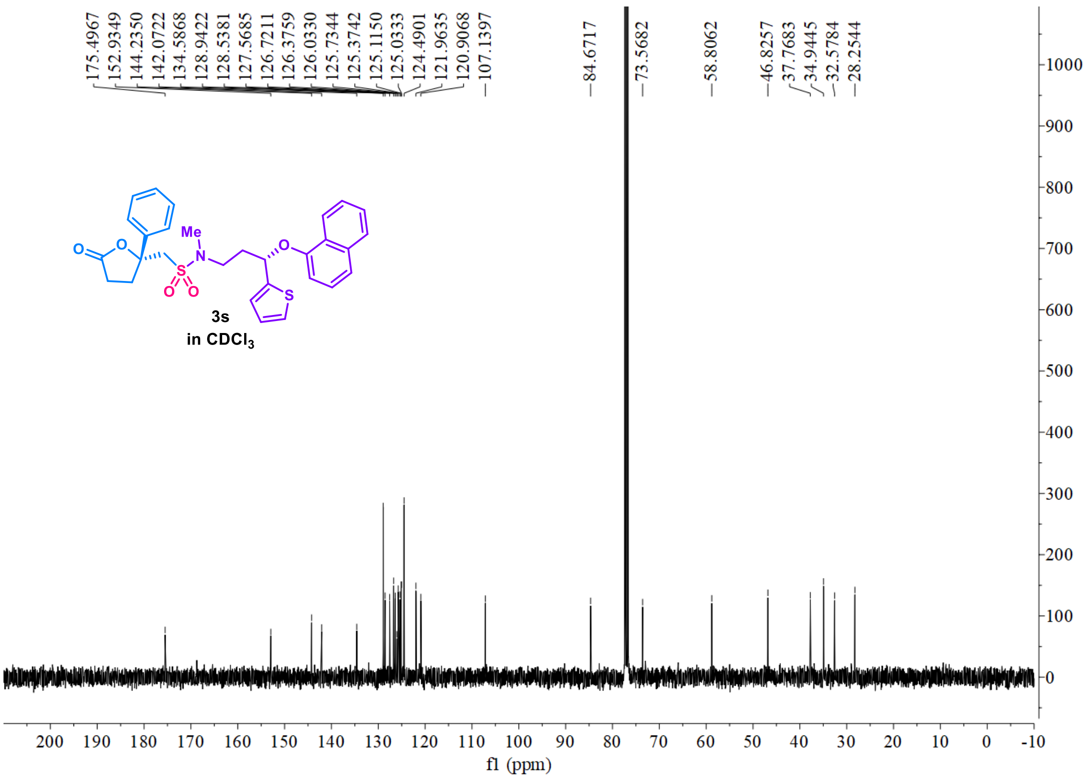

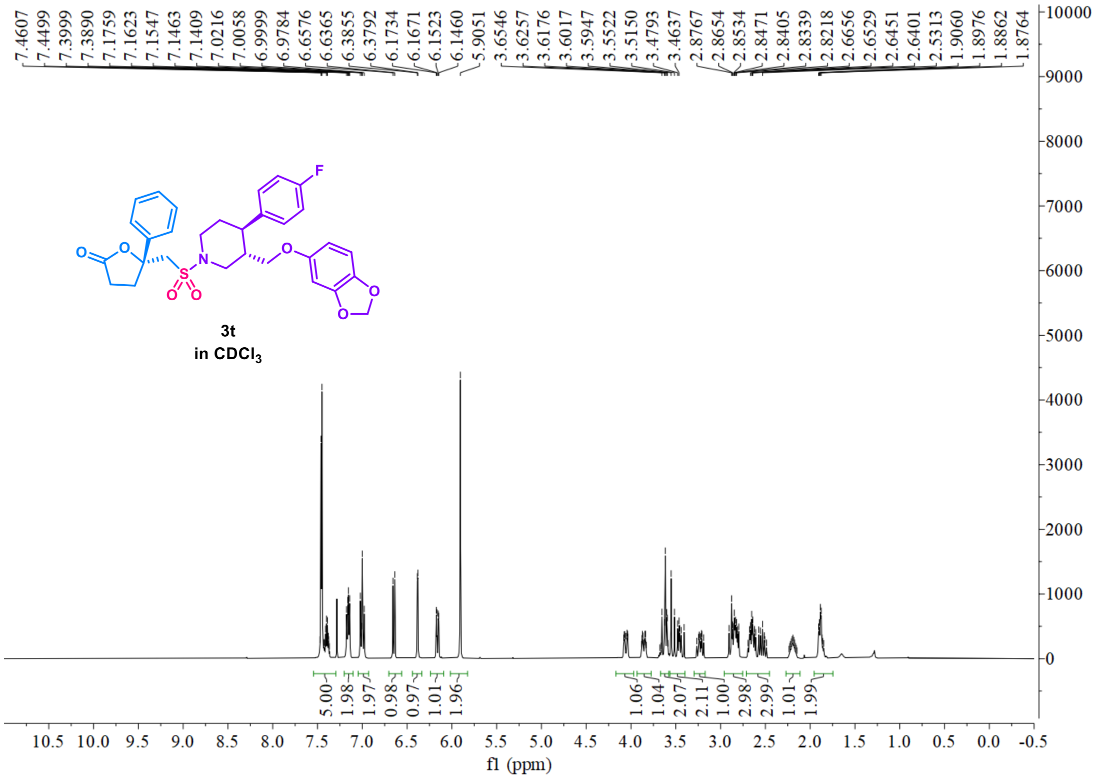

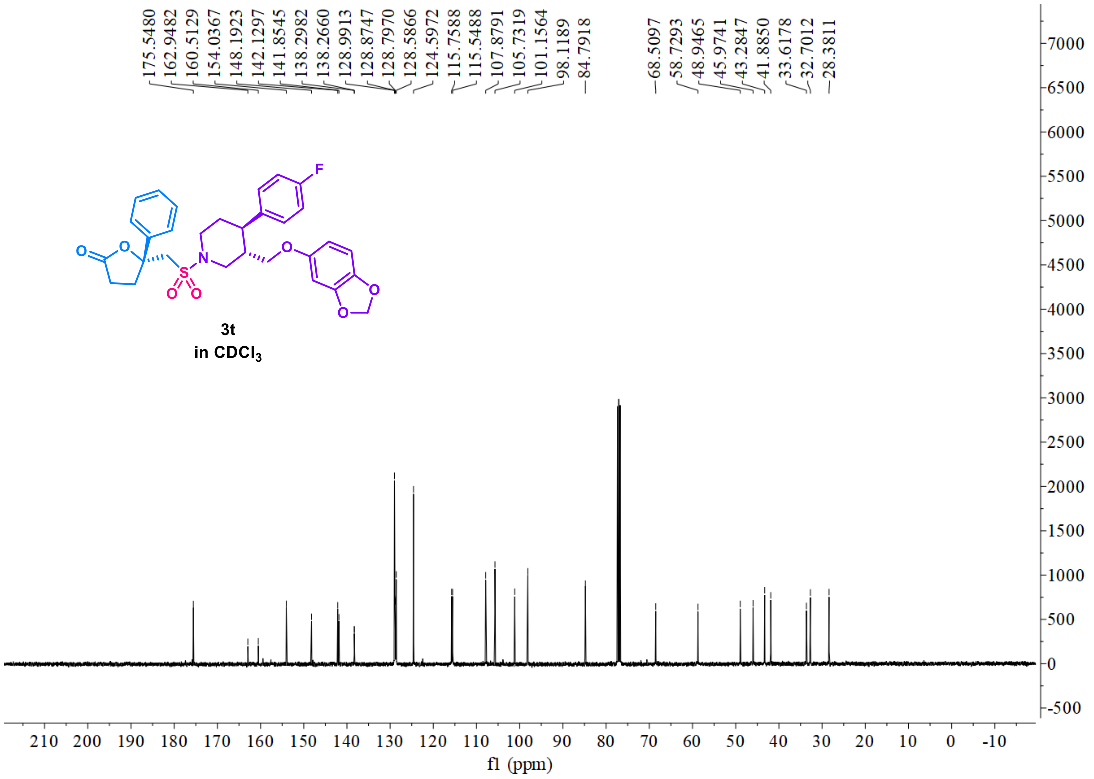

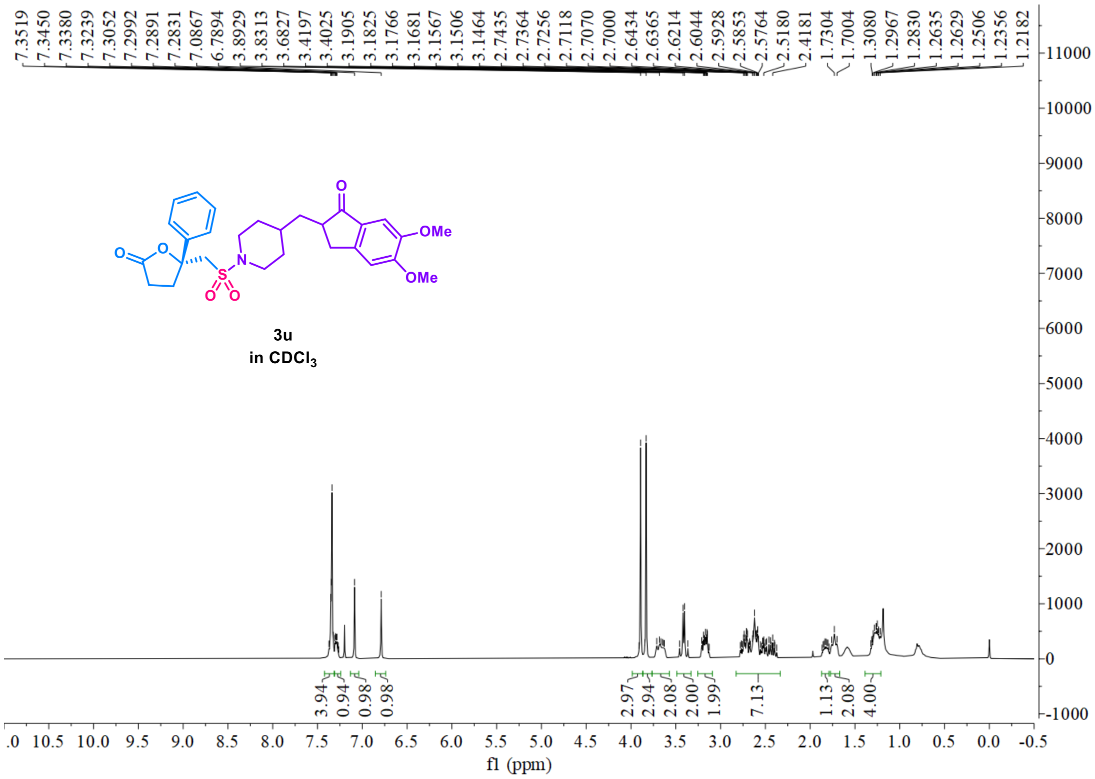

7.3519
7.3450
7.3380
7.3239
7.3052
7.2992
7.2891
7.2831
7.0867
6.7894
3.8929
3.8313
3.6827
3.4197
3.4025
3.1905
3.1825
3.1766
3.1681
3.1567
3.1506
3.1464
2.7435
2.7364
2.7256
2.7118
2.7070
2.7000
2.6434
2.6365
2.6214
2.6044
2.5928
2.5853
2.5764
2.5180
2.4181
1.7304
1.7004
1.3080
1.2967
1.2830
1.2635
1.2629
1.2506
1.2356
1.2182
OMe
OMe
3u
in CDCl3
3.94
0.94
0.98
0.98
2.97
2.94
2.08
2.00
1.99
7.13
1.13
2.08
4.00
f1 (ppm)

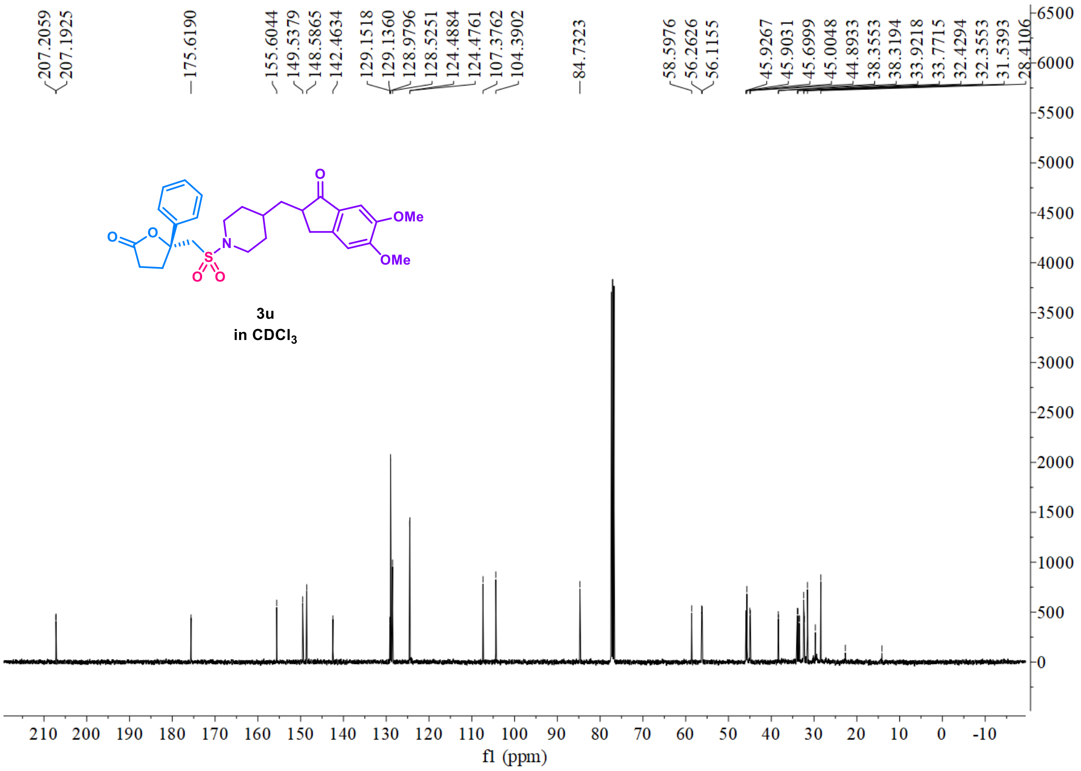

207.2059
207.1925
175.6190
155.6044
149.5379
148.5865
142.4634
129.1518
129.1360
128.9796
128.5251
124.4884
124.4761
107.3762
104.3902
84.7323
58.5976
56.2626
56.1155
45.9267
45.9031
45.6999
45.0048
44.8933
38.3553
38.3194
33.9218
33.7715
32.4294
32.3553
31.5393
28.4106
OMe
OMe
3u
in CDCl3
f1 (ppm)

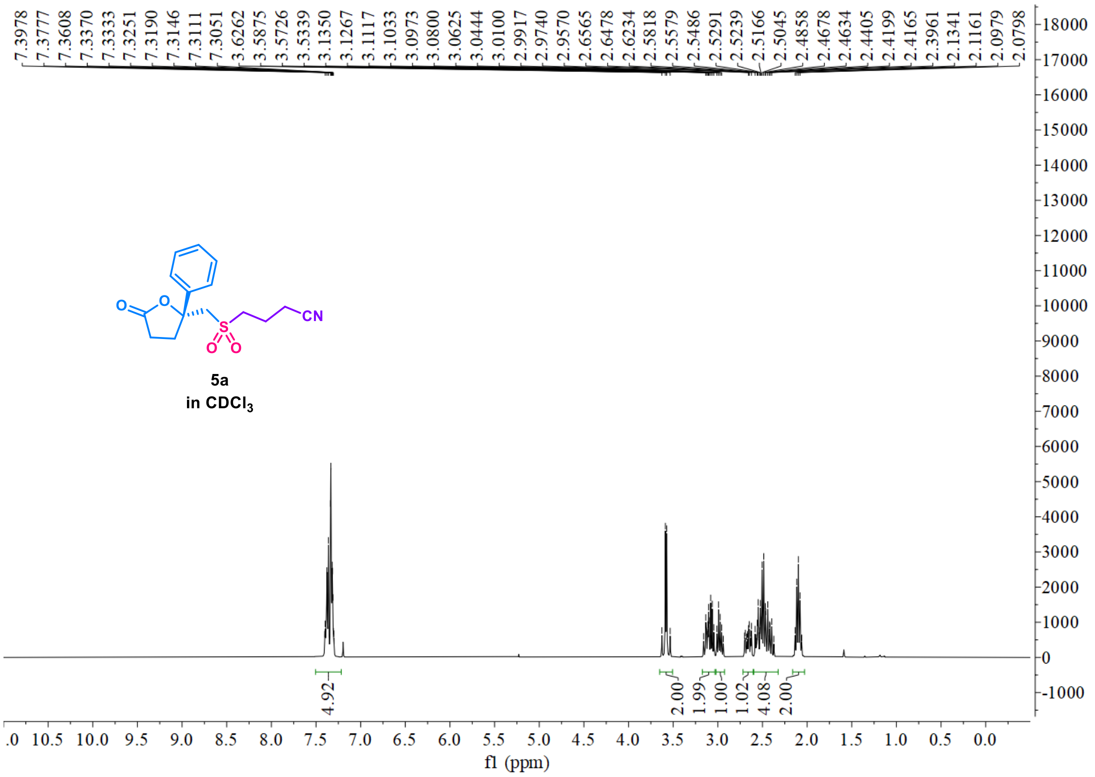

7.3978
7.3777
7.3608
7.3370
7.3333
7.3251
7.3190
7.3146
7.3111
7.3051
3.6262
3.5875
3.5726
3.5339
3.1350
3.1267
3.1117
3.1033
3.0973
3.0800
3.0625
3.0444
3.0100
2.9917
2.9740
2.9570
2.6565
2.6478
2.6234
2.5818
2.5579
2.5486
2.5291
2.5239
2.5166
2.5045
2.4858
2.4678
2.4634
2.4405
2.4199
2.4165
2.3961
2.1341
2.1161
2.0979
2.0798
CN
O
O
S
O
O
5a
in CDCl3
4.92
2.00
1.99
1.00
1.02
4.08
2.00
fl (ppm)

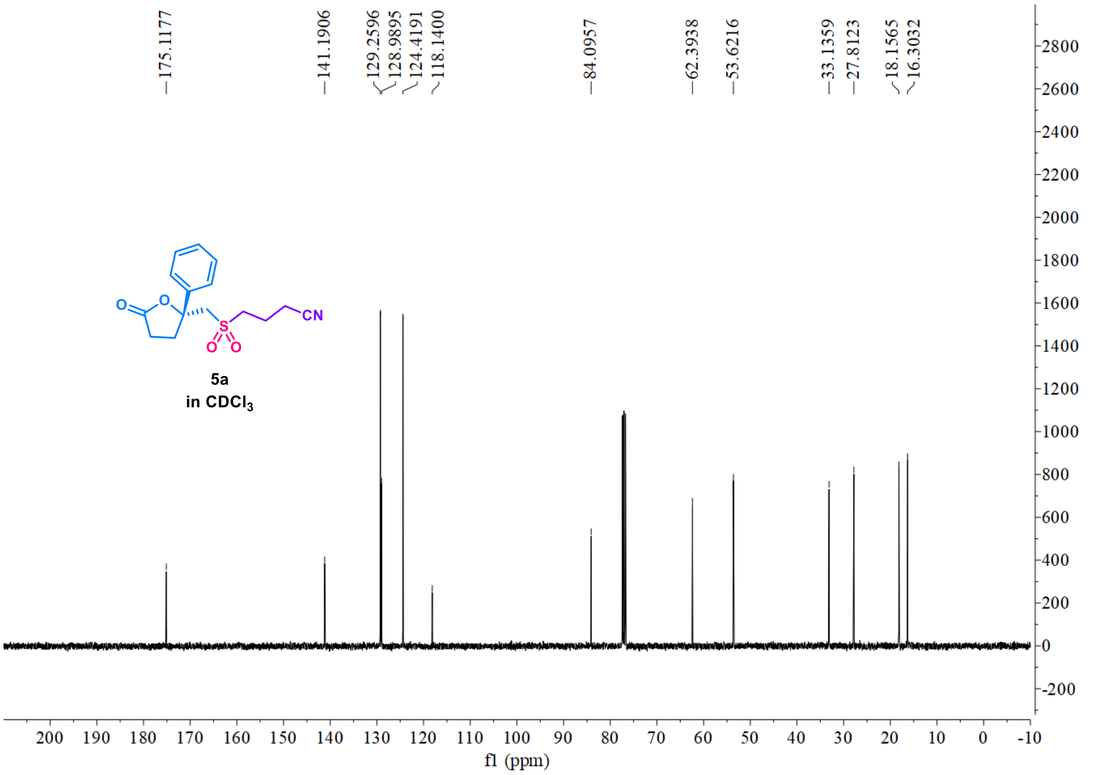

175.1177
141.1906
129.2596
128.9895
124.4191
118.1400
84.0957
62.3938
53.6216
33.1359
27.8123
18.1565
16.3032
CN
O
O
S
O
O
5a
in CDCl3
fl (ppm)

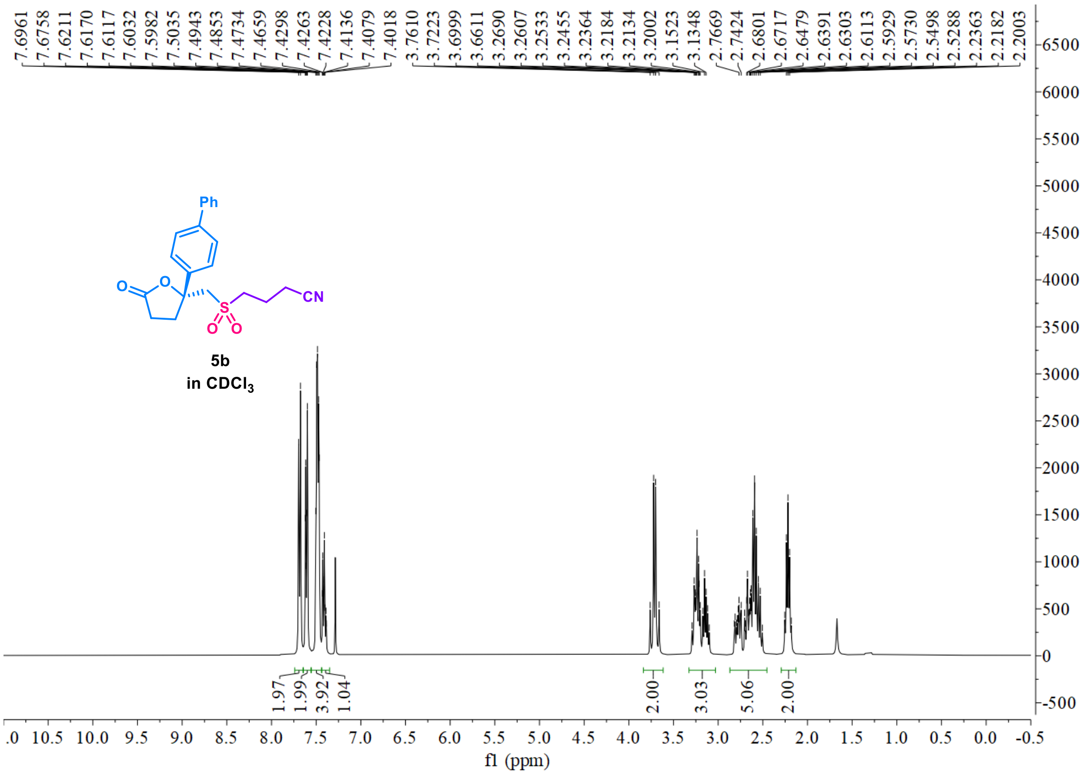

7.6961
7.6758
7.6211
7.6170
7.6117
7.6032
7.5982
7.5035
7.4943
7.4853
7.4734
7.4659
7.4298
7.4263
7.4228
7.4136
7.4079
7.4018
3.7610
3.7223
3.6999
3.6611
3.2690
3.2607
3.2533
3.2455
3.2364
3.2184
3.2134
3.2002
3.1523
3.1348
2.7669
2.7424
2.6801
2.6717
2.6479
2.6391
2.6303
2.6113
2.5929
2.5730
2.5498
2.5288
2.2363
2.2182
2.2003
Ph
O
O
S
O
O
CN
5b
in CDCl3
1.97
1.99
3.92
1.04
2.00
3.03
5.06
2.00
f1 (ppm)

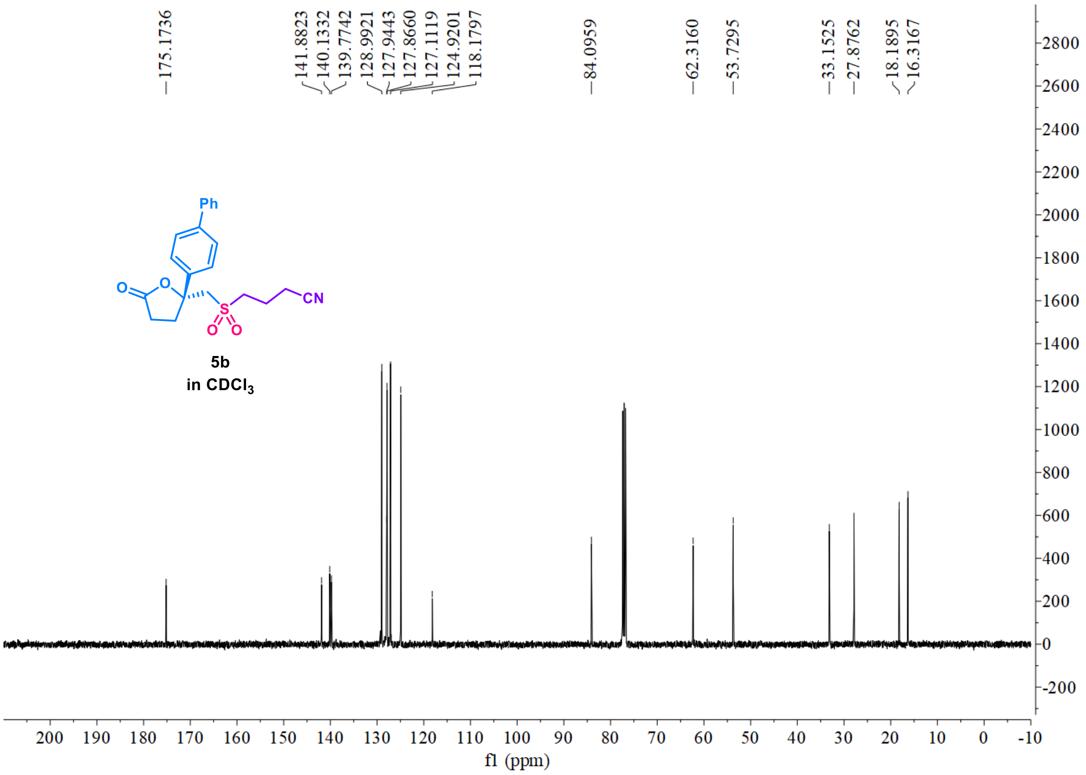

175.1736
141.8823
140.1332
139.7742
128.9921
127.9443
127.8660
127.1119
124.9201
118.1797
84.0959
62.3160
53.7295
33.1525
27.8762
18.1895
16.3167
Ph
O
O
S
O
O
CN
5b
in CDCl3
f1 (ppm)

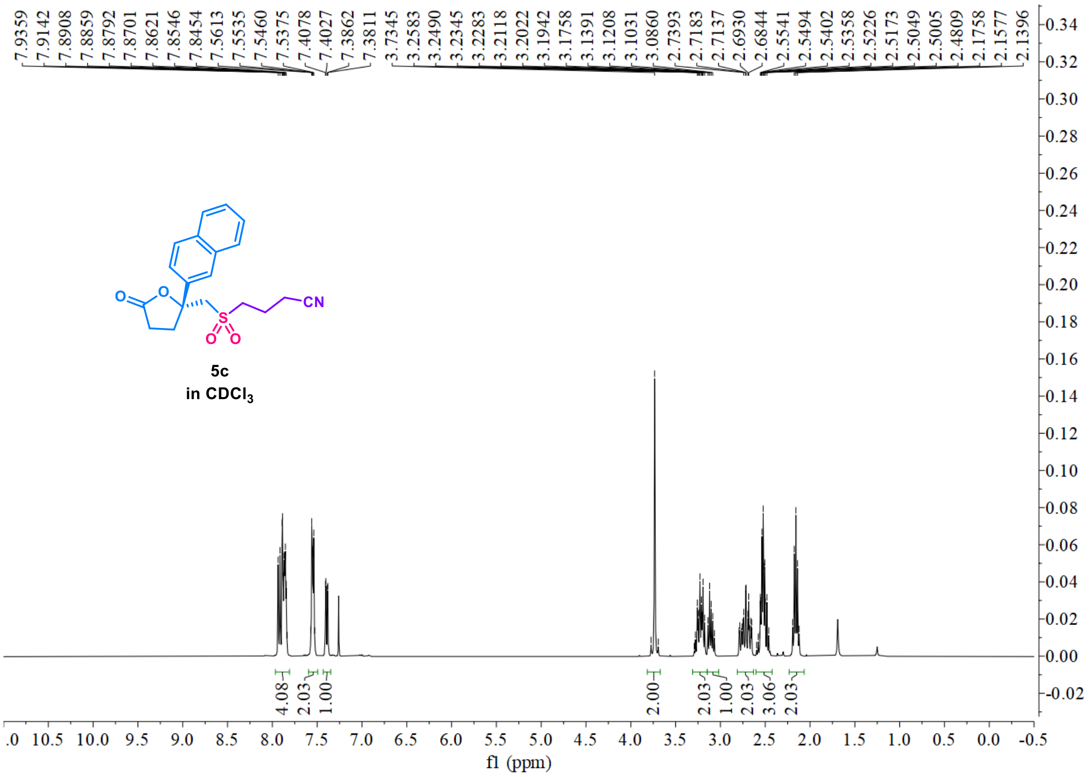

7.9359
7.9142
7.8908
7.8859
7.8792
7.8701
7.8621
7.8546
7.8454
7.5613
7.5535
7.5460
7.5375
7.4078
7.4027
7.3862
7.3811
3.7345
3.2583
3.2490
3.2345
3.2283
3.2118
3.2022
3.1942
3.1758
3.1391
3.1208
3.1031
3.0860
2.7393
2.7183
2.7137
2.6930
2.6844
2.5541
2.5494
2.5402
2.5358
2.5226
2.5173
2.5049
2.5005
2.4809
2.1758
2.1577
2.1396
CN
O
S
5c
in CDCl3
4.08
2.03
1.00
2.00
2.03
1.00
2.03
3.06
2.03
f1 (ppm)

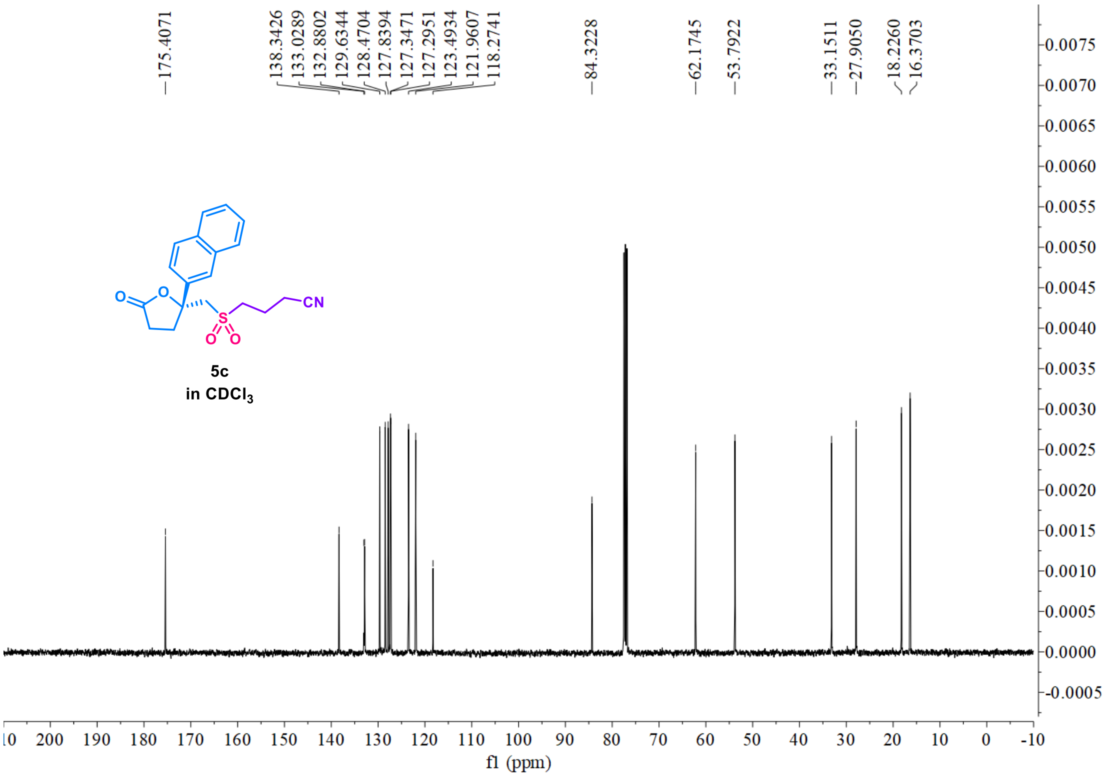

175.4071
138.3426
133.0289
132.8802
129.6344
128.4704
127.8394
127.3471
127.2951
123.4934
121.9607
118.2741
84.3228
62.1745
53.7922
33.1511
27.9050
18.2260
16.3703
CN
O
S
5c
in CDCl3
f1 (ppm)

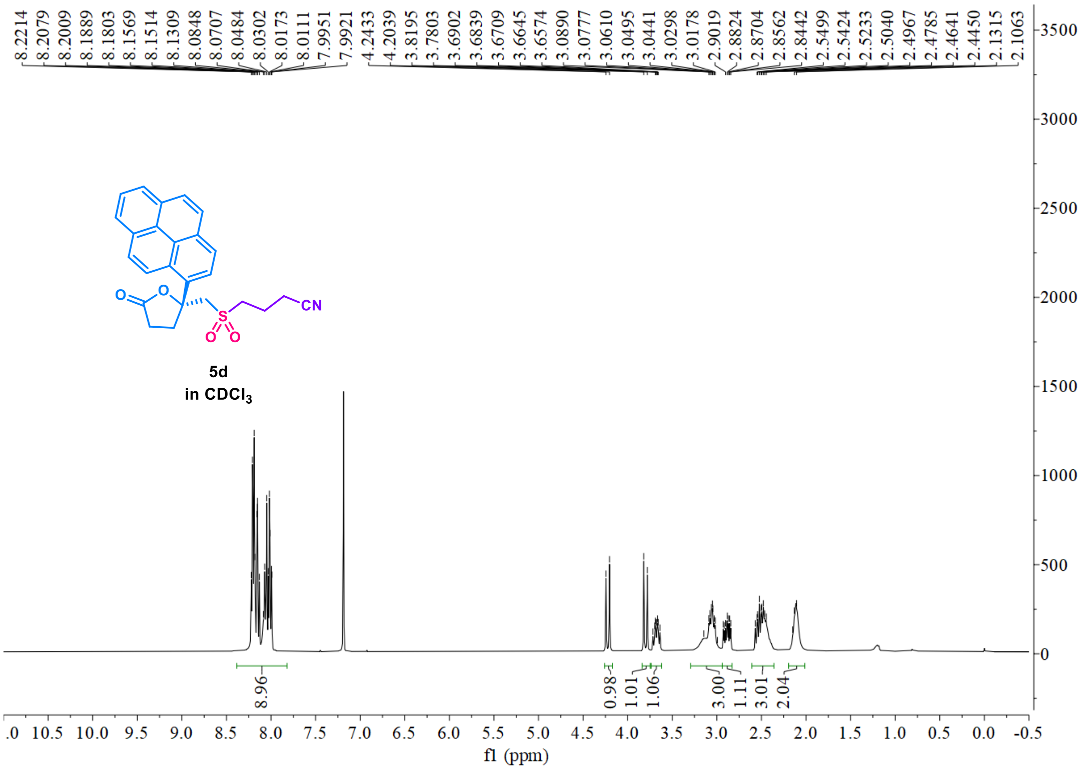
8.2214
8.2079
8.2009
8.1889
8.1803
8.1569
8.1514
8.1309
8.0848
8.0707
8.0484
8.0302
8.0173
8.0111
7.9951
7.9921
4.2433
4.2039
3.8195
3.7803
3.6902
3.6839
3.6709
3.6645
3.6574
3.0890
3.0777
3.0610
3.0495
3.0441
3.0298
3.0178
2.9019
2.8824
2.8704
2.8562
2.8442
2.5499
2.5424
2.5233
2.5040
2.4967
2.4785
2.4641
2.4450
2.1315
2.1063
5d
in $CDCl_3$
8.96
0.98
1.01
1.06
3.00
1.11
3.01
2.04
f1 (ppm)

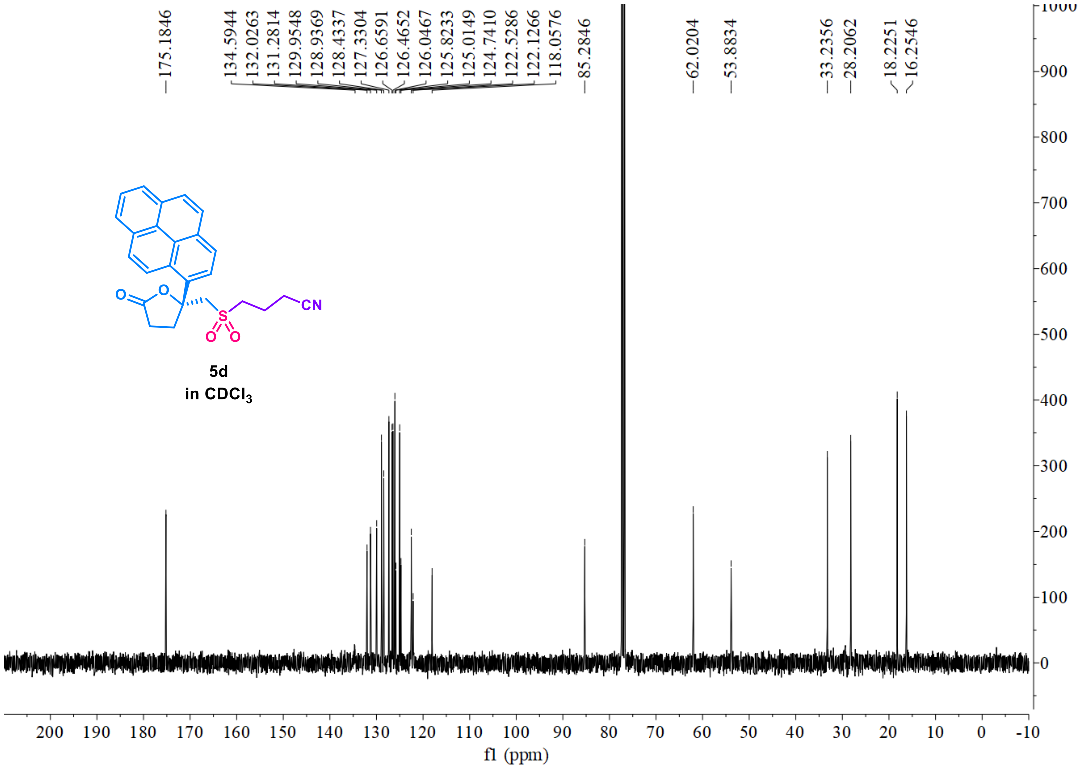
175.1846
134.5944
132.0263
131.2814
129.9548
128.9369
128.4337
127.3304
126.6591
126.4652
126.0467
125.8233
125.0149
124.7410
122.5286
122.1266
118.0576
85.2846
62.0204
53.8834
33.2356
28.2062
18.2251
16.2546
5d
in $CDCl_3$
f1 (ppm)

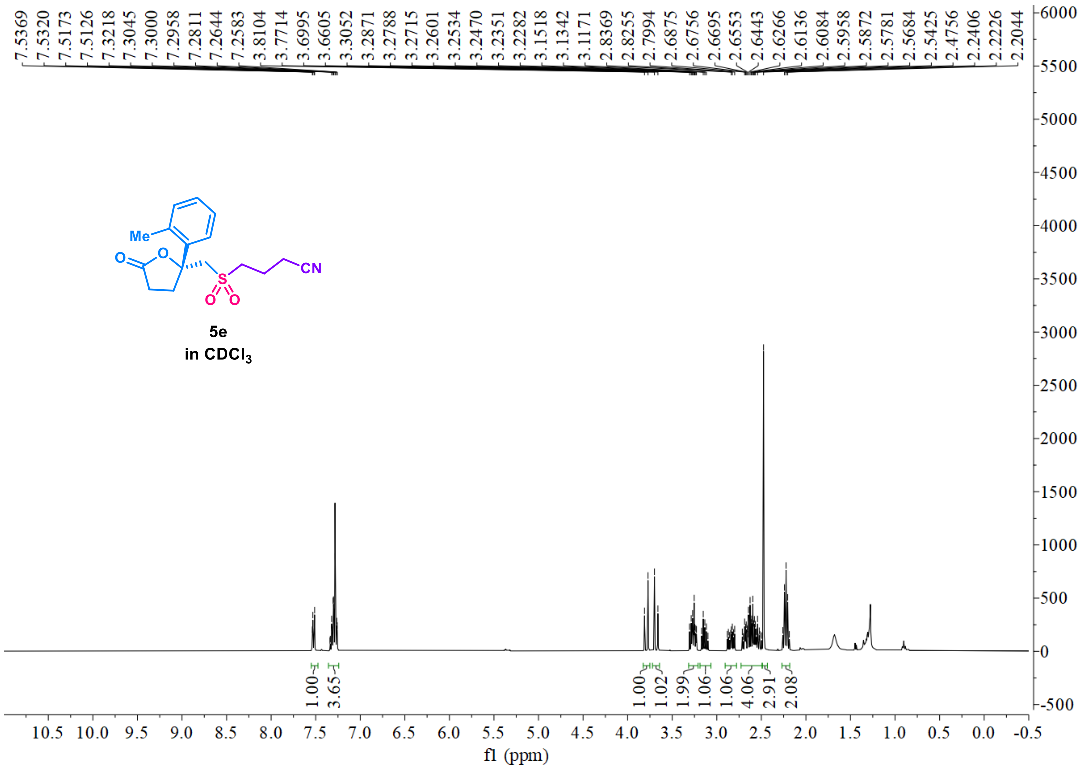
5e
in $CDCl_3$
f1 (ppm)

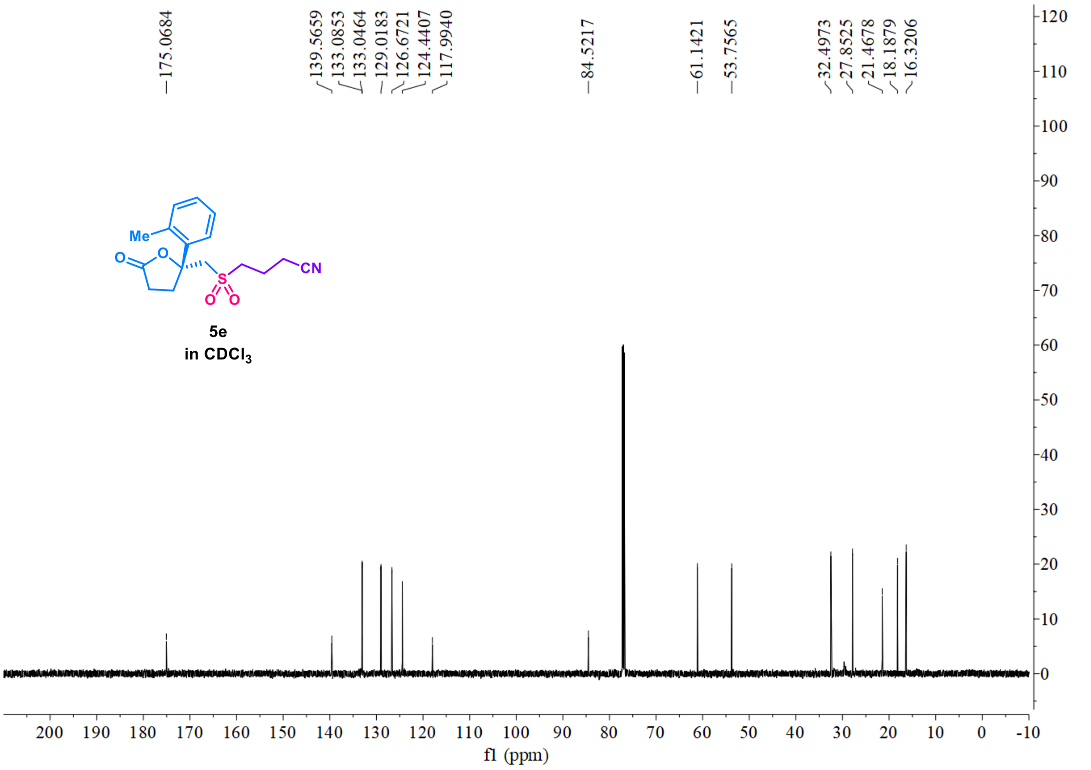
5e
in $CDCl_3$
175.0684
139.5659
133.0853
133.0464
129.0183
126.6721
124.4407
117.9940
84.5217
61.1421
53.7565
32.4973
27.8525
21.4678
18.1879
16.3206
f1 (ppm)

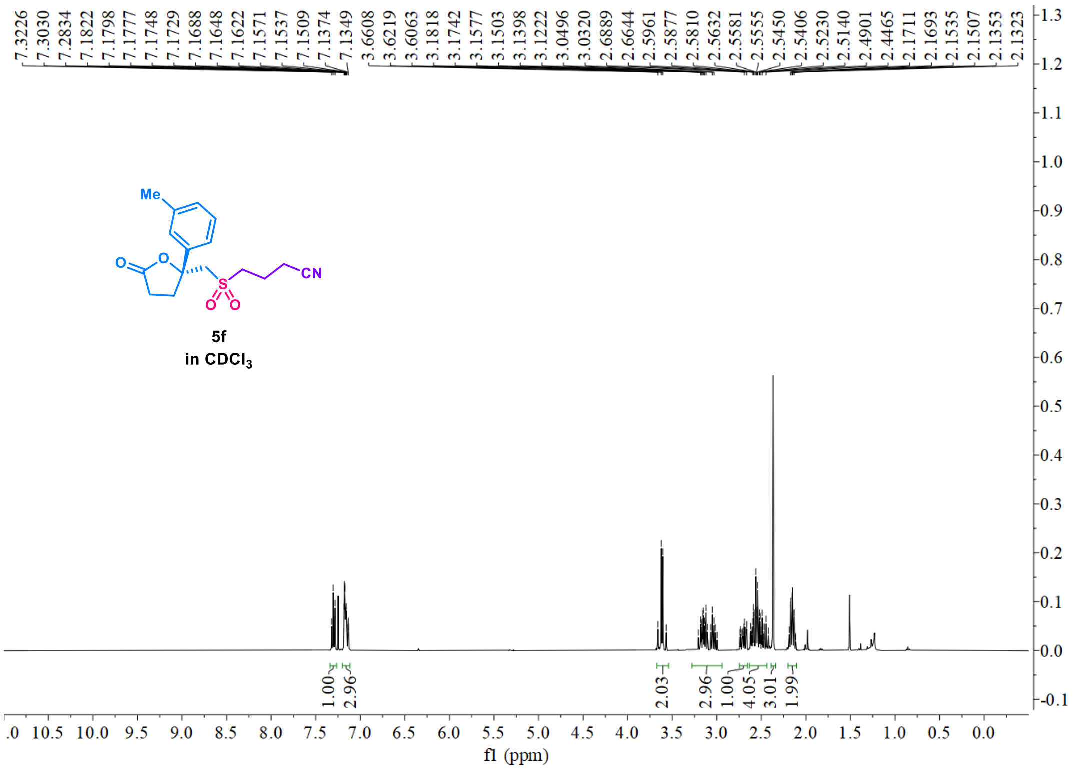
Me
O
O
S
O
O
CN
5f
in CDCl3
f1 (ppm)

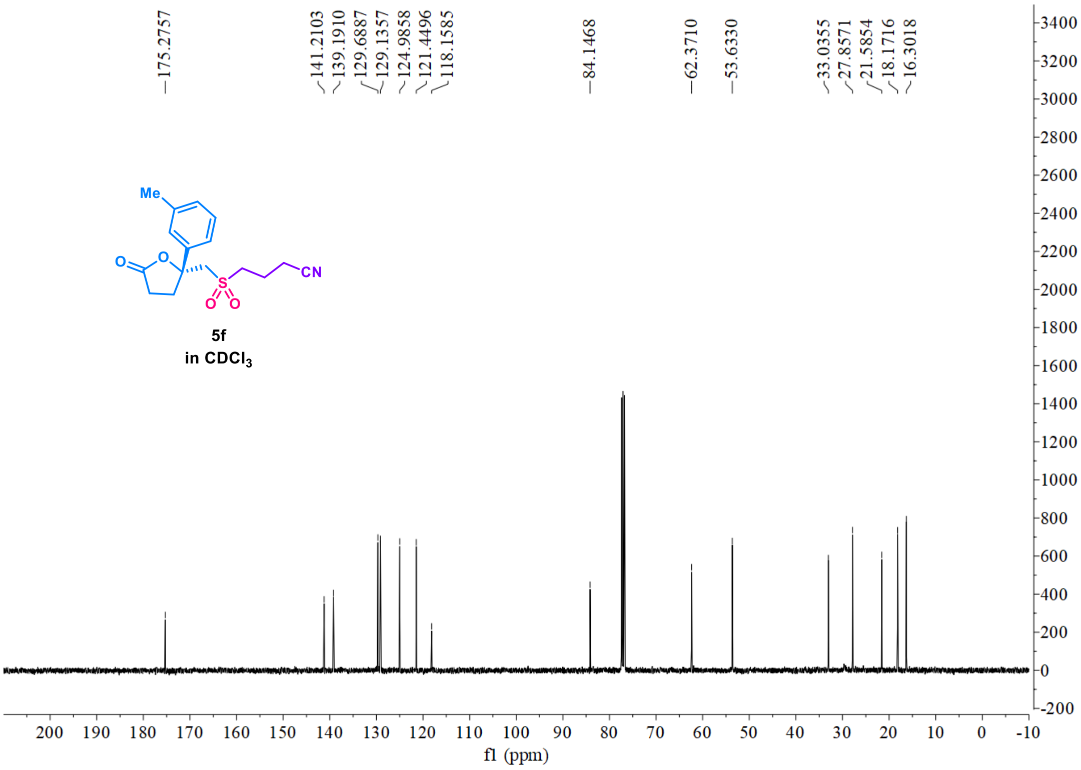
175.2757
141.2103
139.1910
129.6887
129.1357
124.9858
121.4496
118.1585
84.1468
62.3710
53.6330
33.0355
27.8571
21.5854
18.1716
16.3018
Me
O
O
S
O
O
CN
5f
in CDCl3
f1 (ppm)

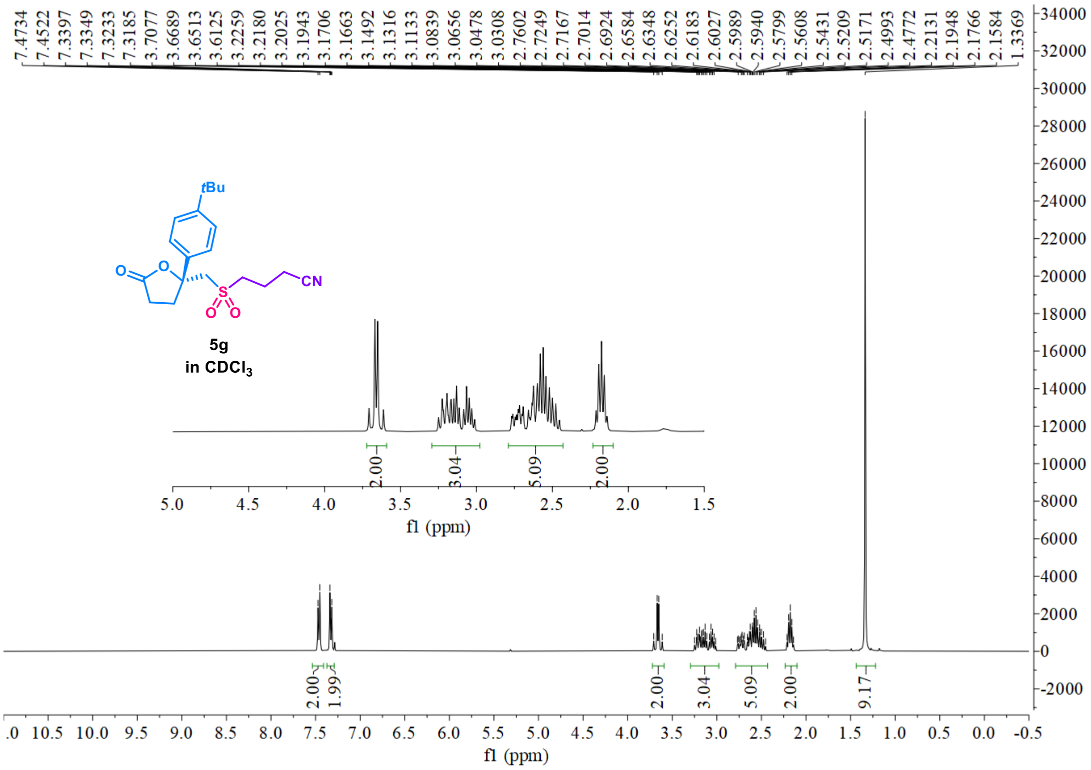
7.4734
7.4522
7.3397
7.3349
7.3233
7.3185
3.7077
3.6689
3.6513
3.6125
3.2259
3.2180
3.2025
3.1943
3.1706
3.1663
3.1492
3.1316
3.1133
3.0839
3.0656
3.0478
3.0308
2.7602
2.7249
2.7167
2.7014
2.6924
2.6584
2.6348
2.6252
2.6183
2.6027
2.5989
2.5940
2.5799
2.5608
2.5431
2.5209
2.5171
2.4993
2.4772
2.2131
2.1948
2.1766
2.1584
1.3369
tBu
O
O
S
O
O
CN
5g
in CDCl3
2.00
3.04
5.09
2.00
5.0 4.5 4.0 3.5 3.0 2.5 2.0 1.5
f1 (ppm)
2.00
1.99
2.00
3.04
5.09
2.00
9.17
f1 (ppm)

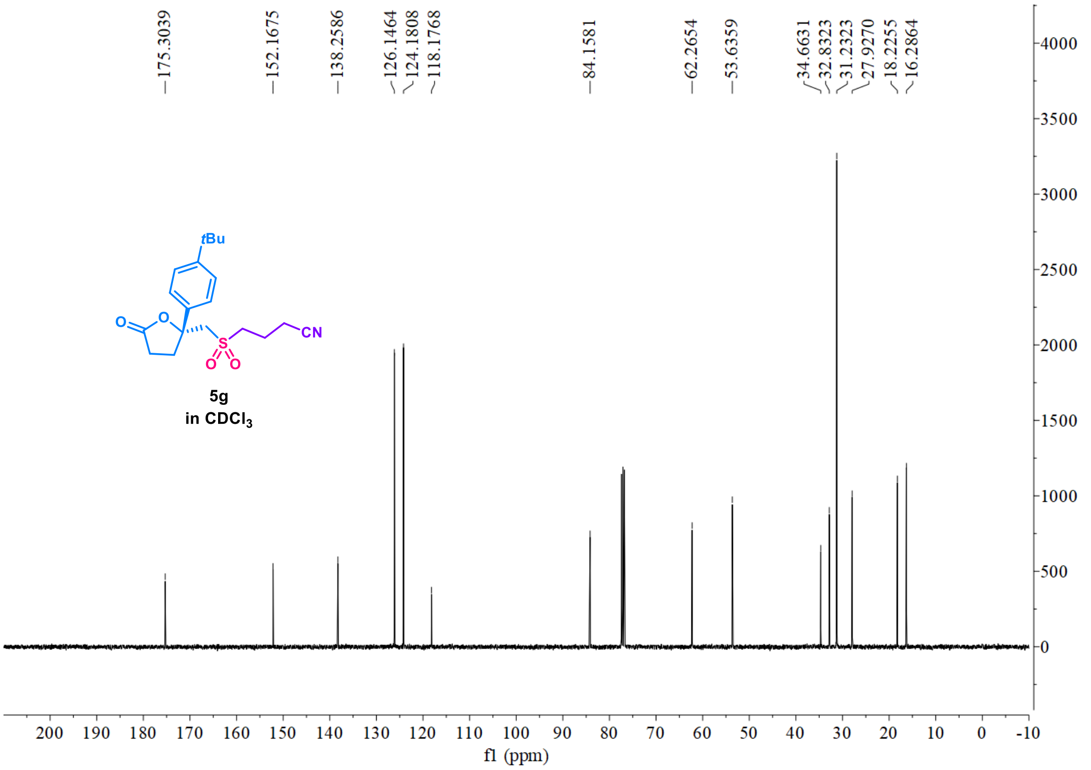
175.3039
152.1675
138.2586
126.1464
124.1808
118.1768
84.1581
62.2654
53.6359
34.6631
32.8323
31.2323
27.9270
18.2255
16.2864
tBu
O
O
S
O
O
CN
5g
in CDCl3
f1 (ppm)

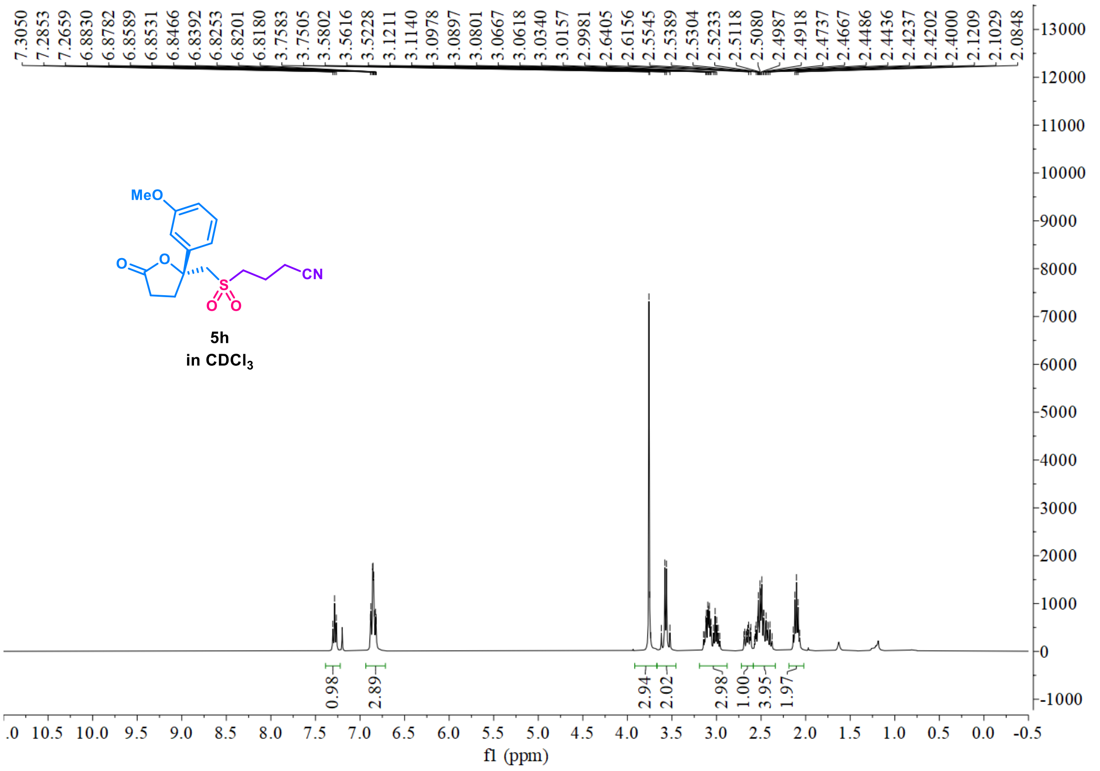
MeO
O
O
S
O O
CN
5h
in CDCl3
f1 (ppm)

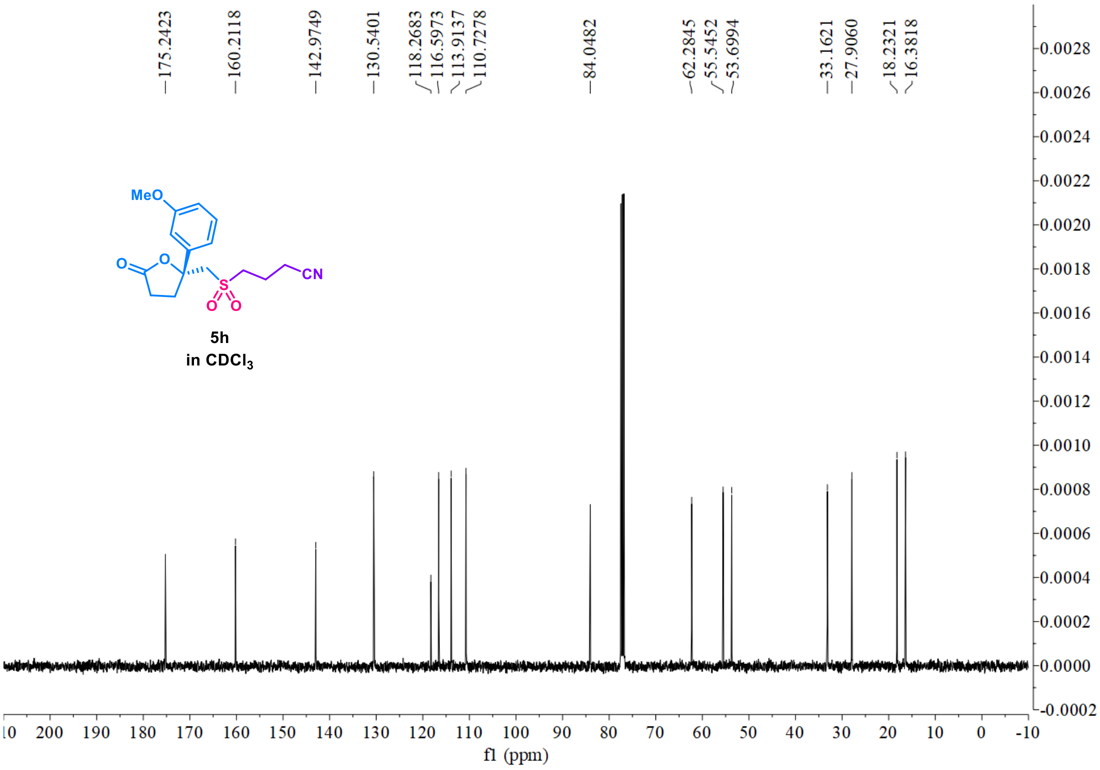
MeO
O
O
S
O O
CN
5h
in CDCl3
f1 (ppm)

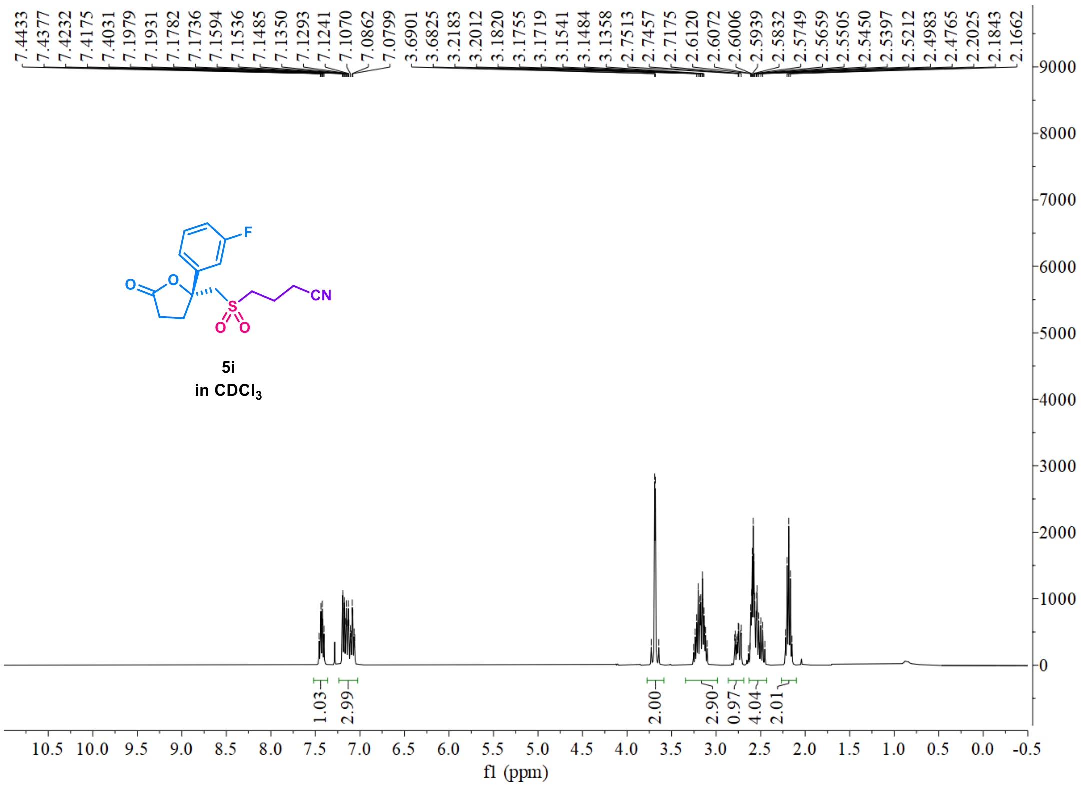
5i
in CDCl3
f1 (ppm)

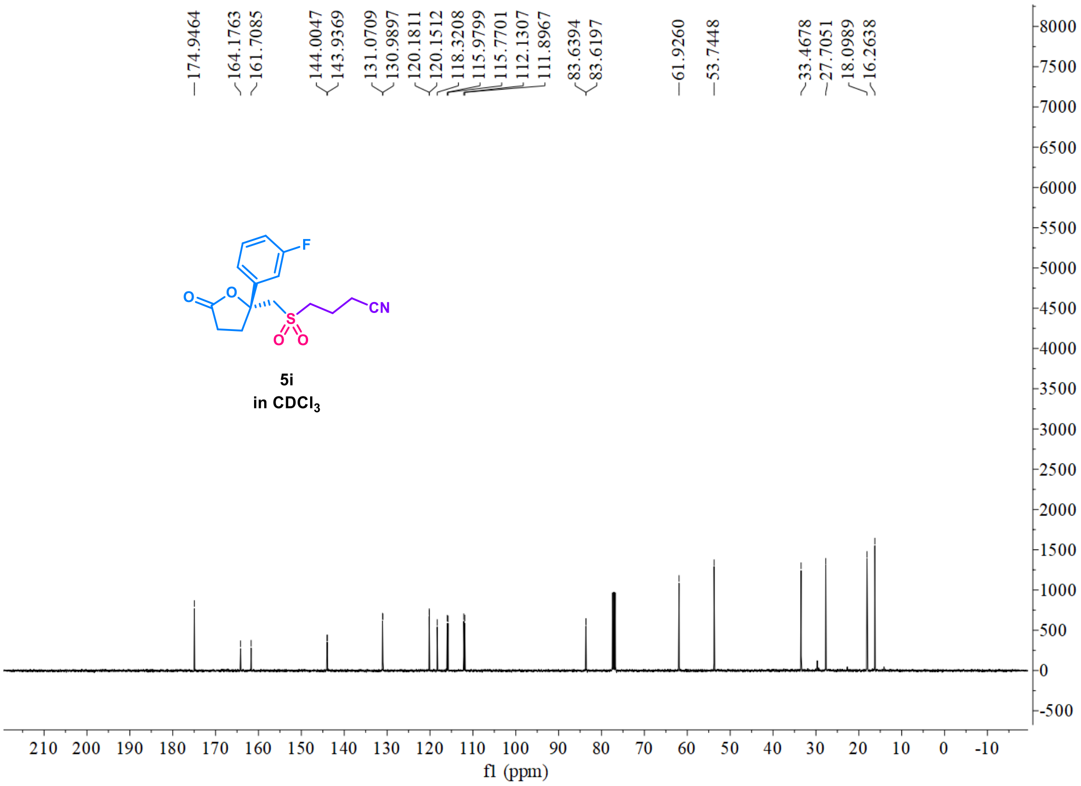
5i
in CDCl3
f1 (ppm)

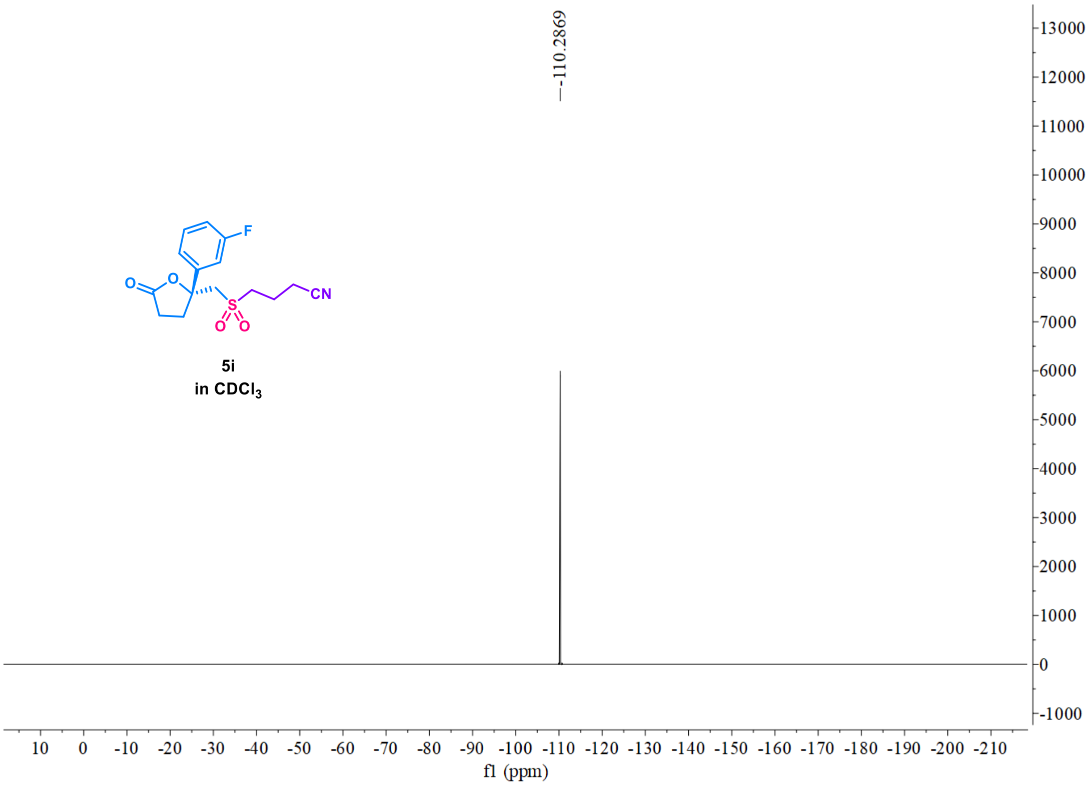
-110.2869
5i
in CDCl$_3$
f1 (ppm)

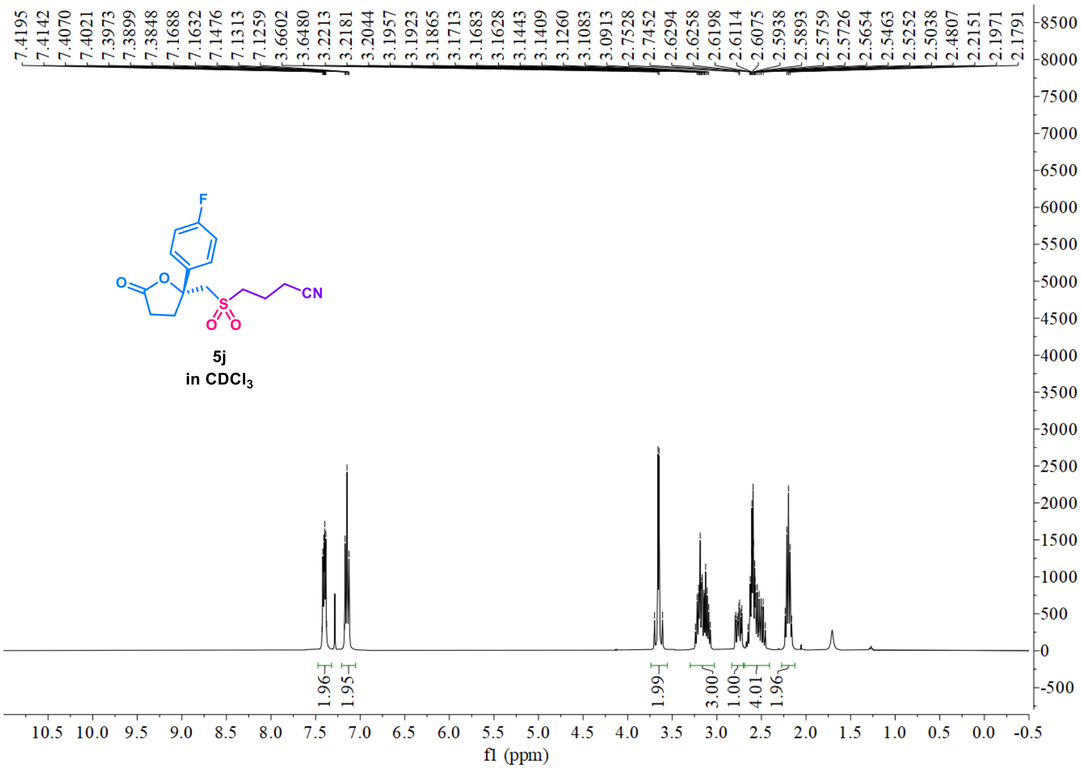
7.4195
7.4142
7.4070
7.4021
7.3973
7.3899
7.3848
7.1688
7.1632
7.1476
7.1313
7.1259
3.6602
3.6480
3.2213
3.2181
3.2044
3.1957
3.1923
3.1865
3.1713
3.1683
3.1628
3.1443
3.1409
3.1260
3.1083
3.0913
2.7528
2.7452
2.6294
2.6258
2.6198
2.6114
2.6075
2.5938
2.5893
2.5759
2.5726
2.5654
2.5463
2.5252
2.5038
2.4807
2.2151
2.1971
2.1791
5j
in CDCl$_3$
1.96
1.95
1.99
3.00
1.00
4.01
1.96
f1 (ppm)

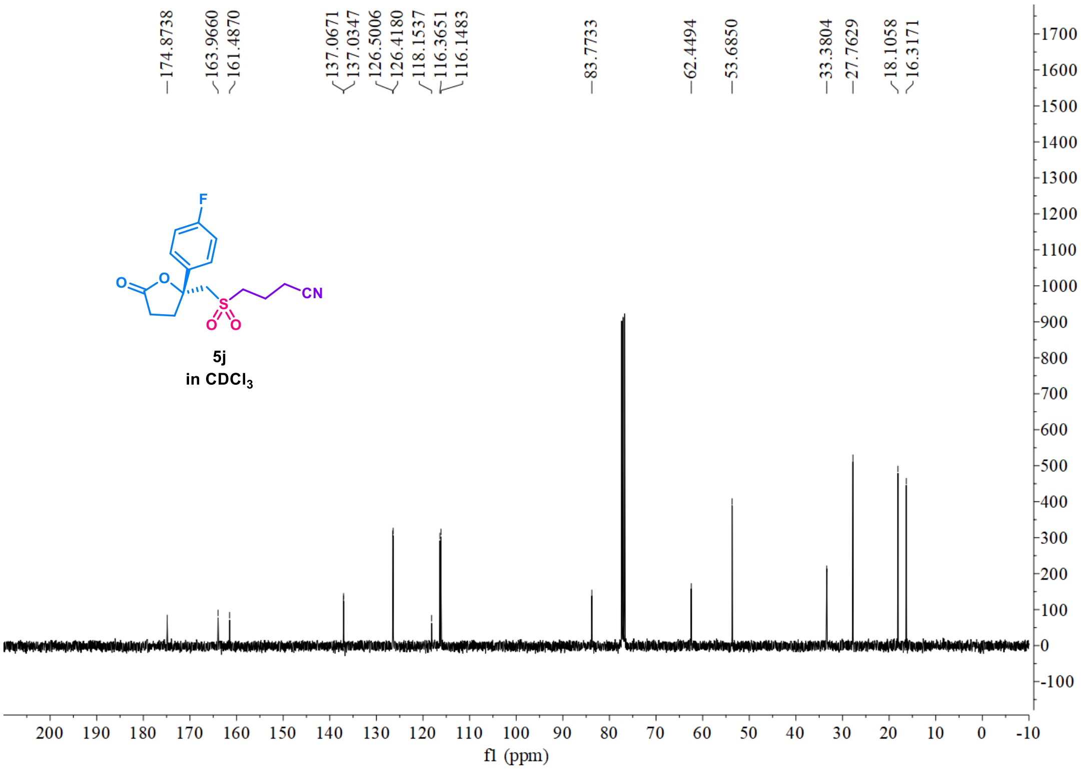
174.8738
163.9660
161.4870
137.0671
137.0347
126.5006
126.4180
118.1537
116.3651
116.1483
83.7733
62.4494
53.6850
33.3804
27.7629
18.1058
16.3171
5j
in CDCl3
f1 (ppm)

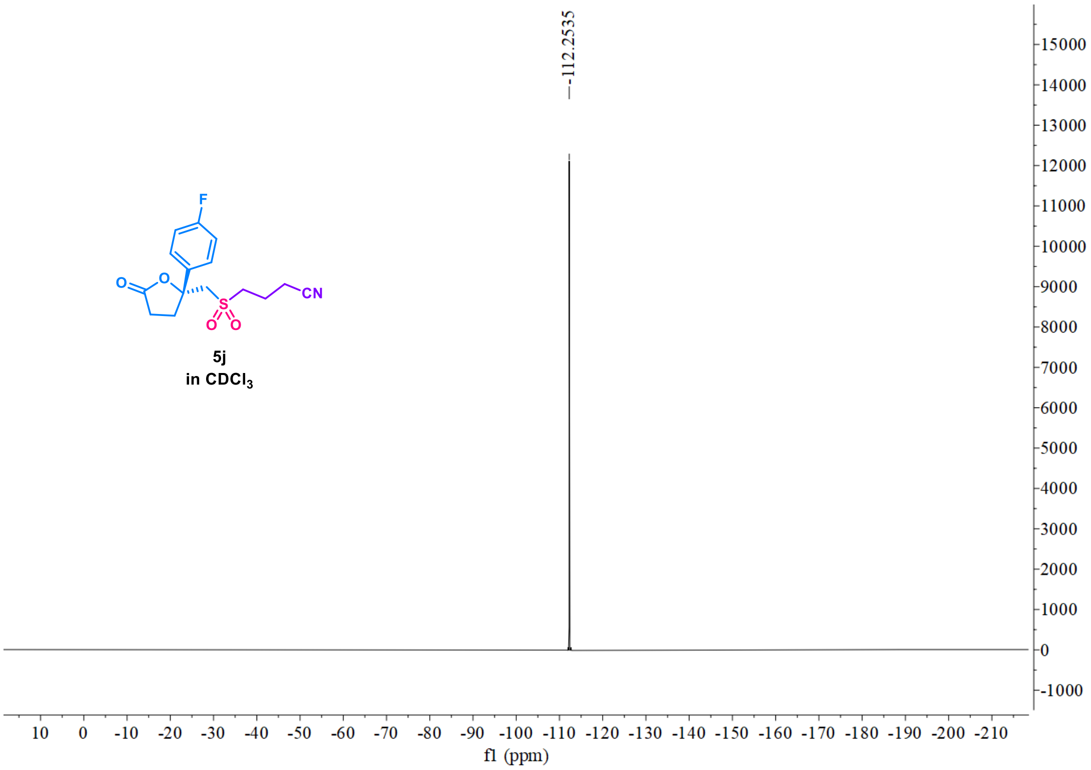
-112.2535
5j
in CDCl3
f1 (ppm)

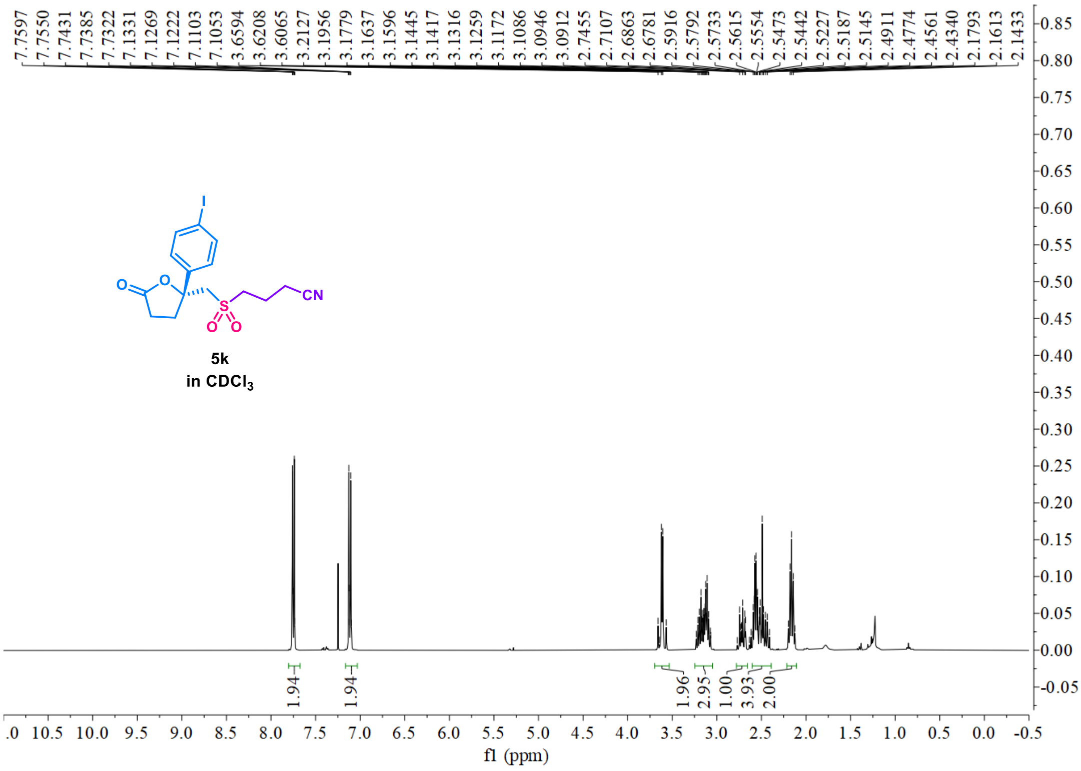
7.7597
7.7550
7.7431
7.7385
7.7322
7.1331
7.1269
7.1222
7.1103
7.1053
3.6594
3.6208
3.6065
3.2127
3.1956
3.1779
3.1637
3.1596
3.1445
3.1417
3.1316
3.1259
3.1172
3.1086
3.0946
3.0912
2.7455
2.7107
2.6863
2.6781
2.5916
2.5792
2.5733
2.5615
2.5554
2.5473
2.5442
2.5227
2.5187
2.5145
2.4911
2.4774
2.4561
2.4340
2.1793
2.1613
2.1433
5k
in $CDCl_3$
1.94
1.94
1.96
2.95
1.00
3.93
2.00
f1 (ppm)

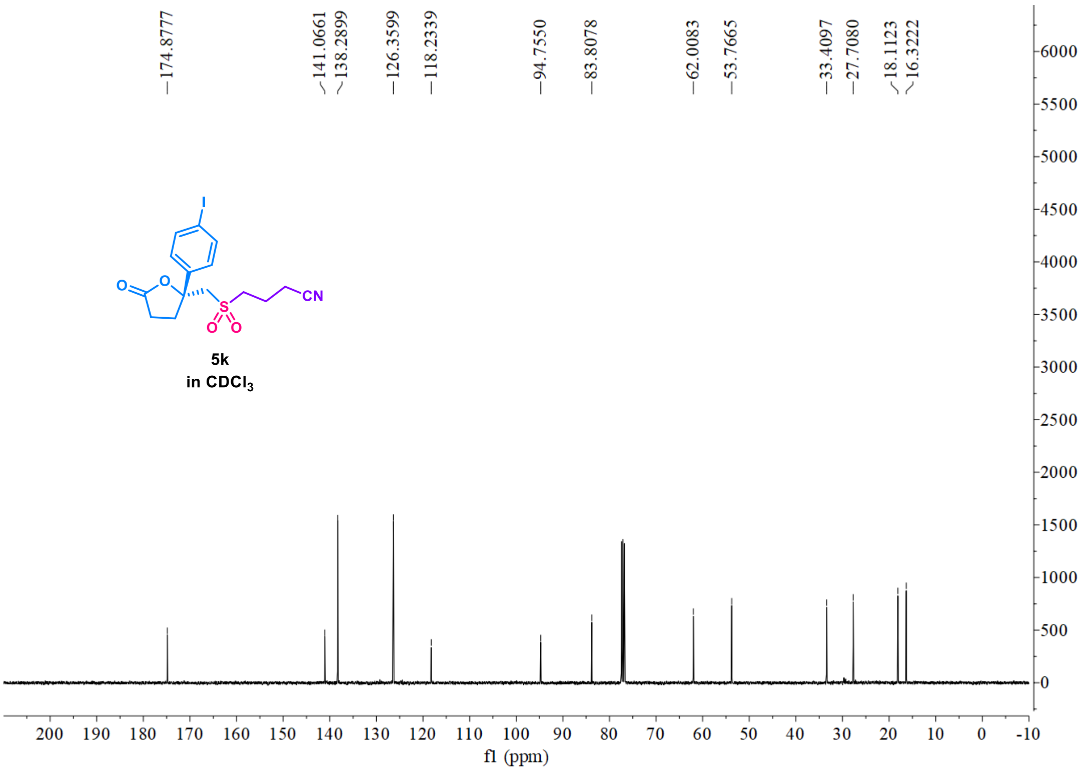
174.8777
141.0661
138.2899
126.3599
118.2339
94.7550
83.8078
62.0083
53.7665
33.4097
27.7080
18.1123
16.3222
5k
in $CDCl_3$
f1 (ppm)

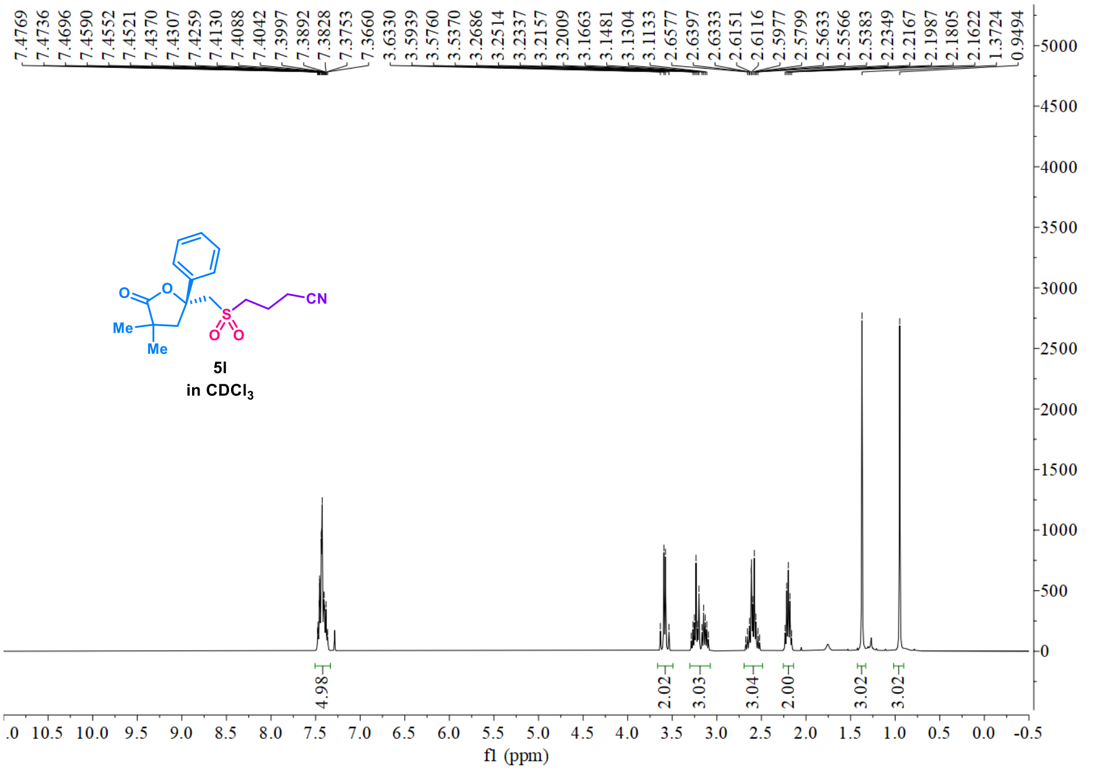
7.4769
7.4736
7.4696
7.4590
7.4552
7.4521
7.4370
7.4307
7.4259
7.4130
7.4088
7.4042
7.3997
7.3892
7.3828
7.3753
7.3660
3.6330
3.5939
3.5760
3.5370
3.2686
3.2514
3.2337
3.2157
3.2009
3.1663
3.1481
3.1304
3.1133
2.6577
2.6397
2.6333
2.6151
2.6116
2.5977
2.5799
2.5633
2.5566
2.5383
2.2349
2.2167
2.1987
2.1805
2.1622
1.3724
0.9494
Me
Me
O
O
S
O
O
CN
5l
in CDCl3
4.98
2.02
3.03
3.04
2.00
3.02
3.02
f1 (ppm)

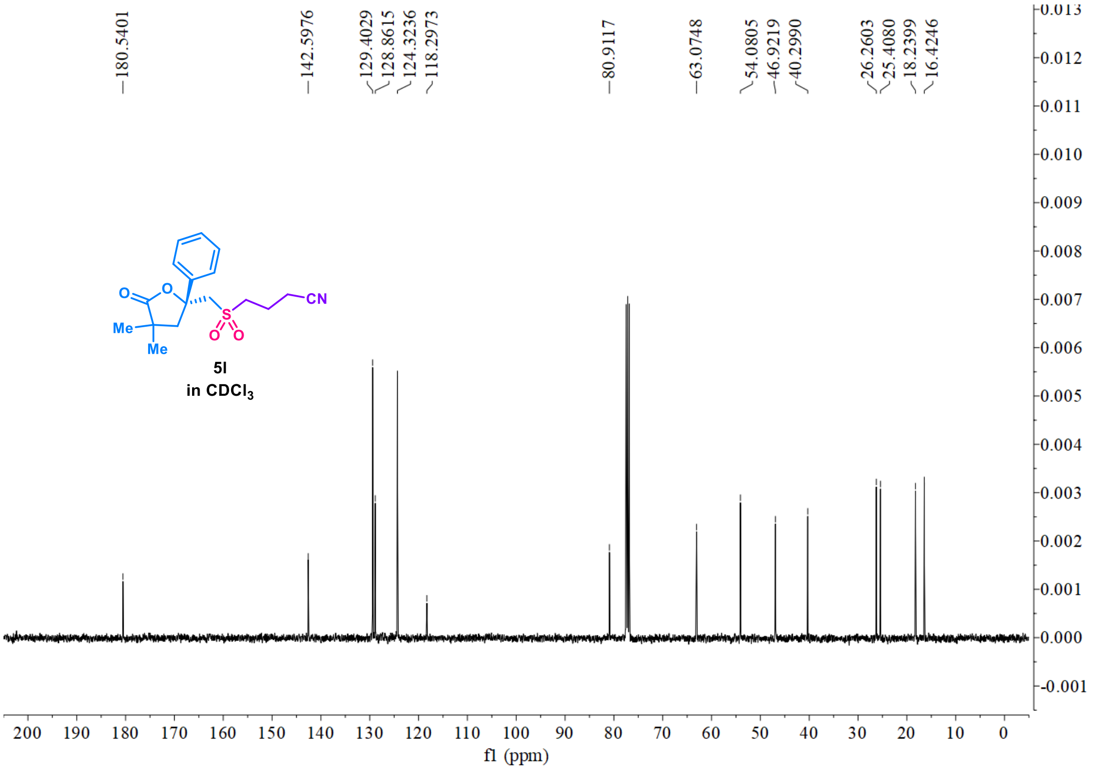
180.5401
142.5976
129.4029
128.8615
124.3236
118.2973
80.9117
63.0748
54.0805
46.9219
40.2990
26.2603
25.4080
18.2399
16.4246
Me
Me
O
O
S
O
O
CN
5l
in CDCl3
f1 (ppm)

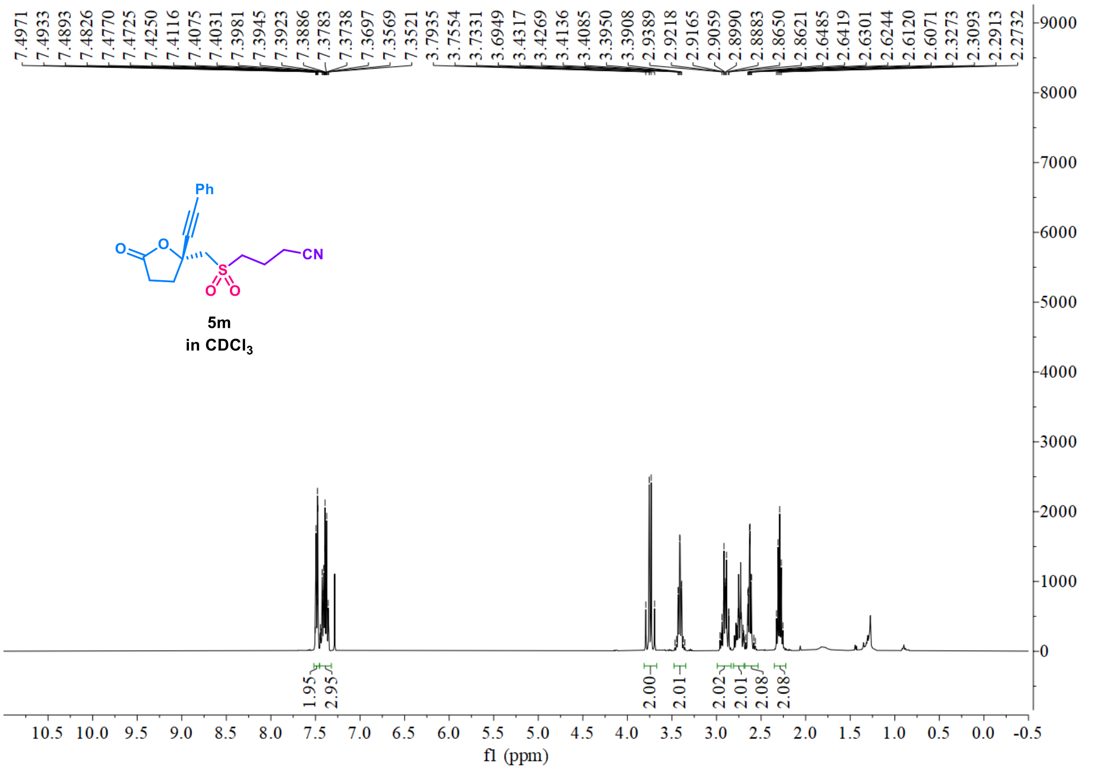
7.4971
7.4933
7.4893
7.4826
7.4770
7.4725
7.4250
7.4116
7.4075
7.4031
7.3981
7.3945
7.3923
7.3886
7.3783
7.3738
7.3697
7.3569
7.3521
3.7935
3.7554
3.7331
3.6949
3.4317
3.4269
3.4136
3.4085
3.3950
3.3908
2.9389
2.9218
2.9165
2.9059
2.8990
2.8883
2.8650
2.8621
2.6485
2.6419
2.6301
2.6244
2.6120
2.6071
2.3273
2.3093
2.2913
2.2732
Ph
O
O
CN
S
O O
5m
in CDCl3
1.95
2.95
2.00
2.01
2.02
2.01
2.08
2.08
f1 (ppm)

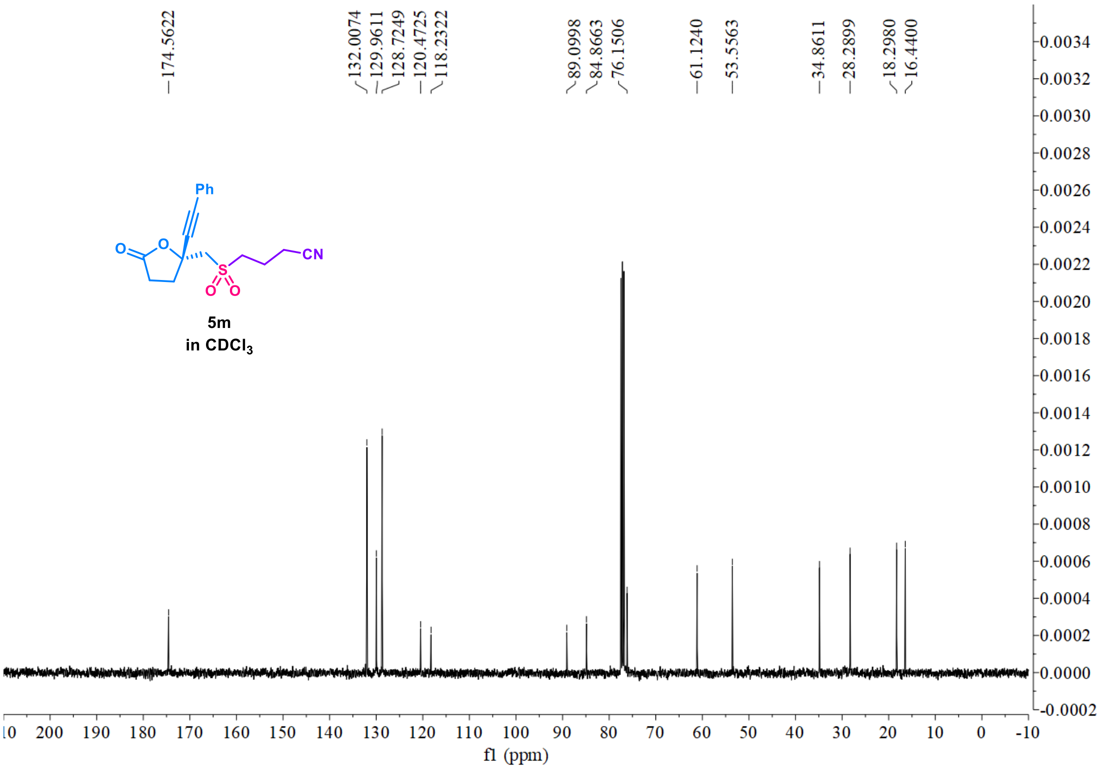
174.5622
132.0074
129.9611
128.7249
120.4725
118.2322
89.0998
84.8663
76.1506
61.1240
53.5563
34.8611
28.2899
18.2980
16.4400
Ph
O
O
CN
S
O O
5m
in CDCl3
f1 (ppm)

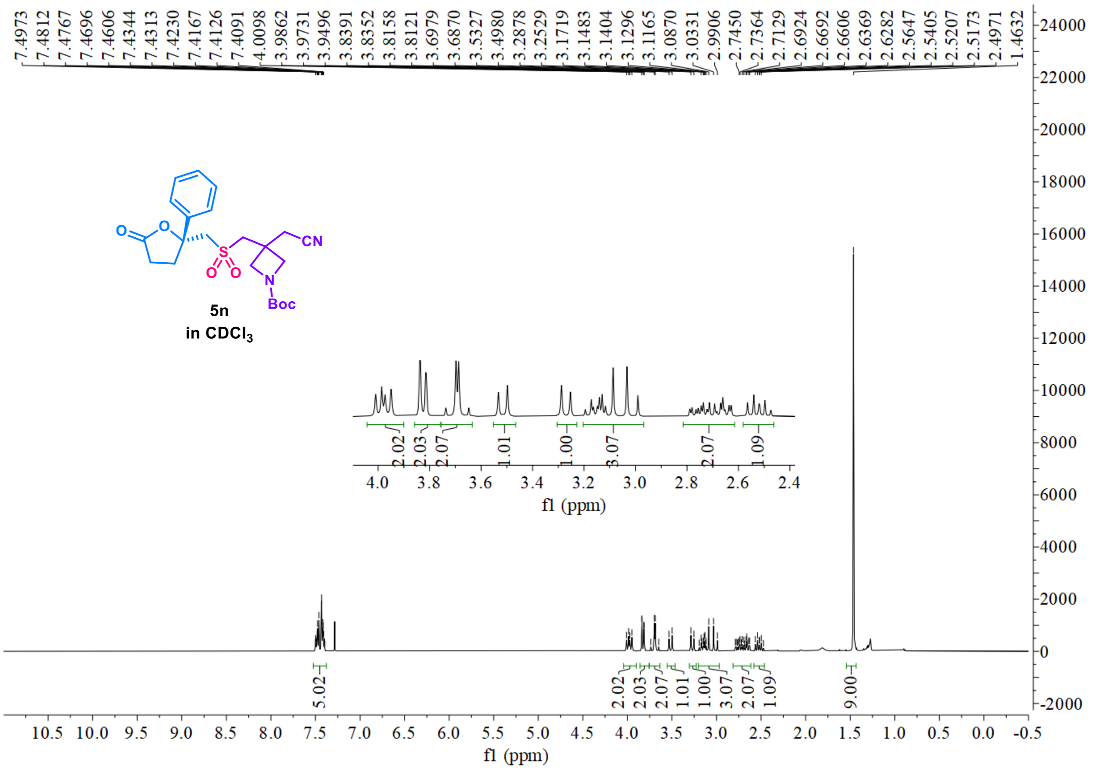
7.4973
7.4812
7.4767
7.4696
7.4606
7.4344
7.4313
7.4230
7.4167
7.4126
7.4091
4.0098
3.9862
3.9731
3.9496
3.8391
3.8352
3.8158
3.8121
3.6979
3.6870
3.5327
3.4980
3.2878
3.2529
3.1719
3.1483
3.1404
3.1296
3.1165
3.0870
3.0331
2.9906
2.7450
2.7364
2.7129
2.6924
2.6692
2.6606
2.6369
2.6282
2.5647
2.5405
2.5207
2.5173
2.4971
1.4632
CN
O
O
S
O O
N
Boc
5n
in CDCl3
2.02
2.03
2.07
1.01
1.00
3.07
2.07
1.09
5.02
9.00
f1 (ppm)

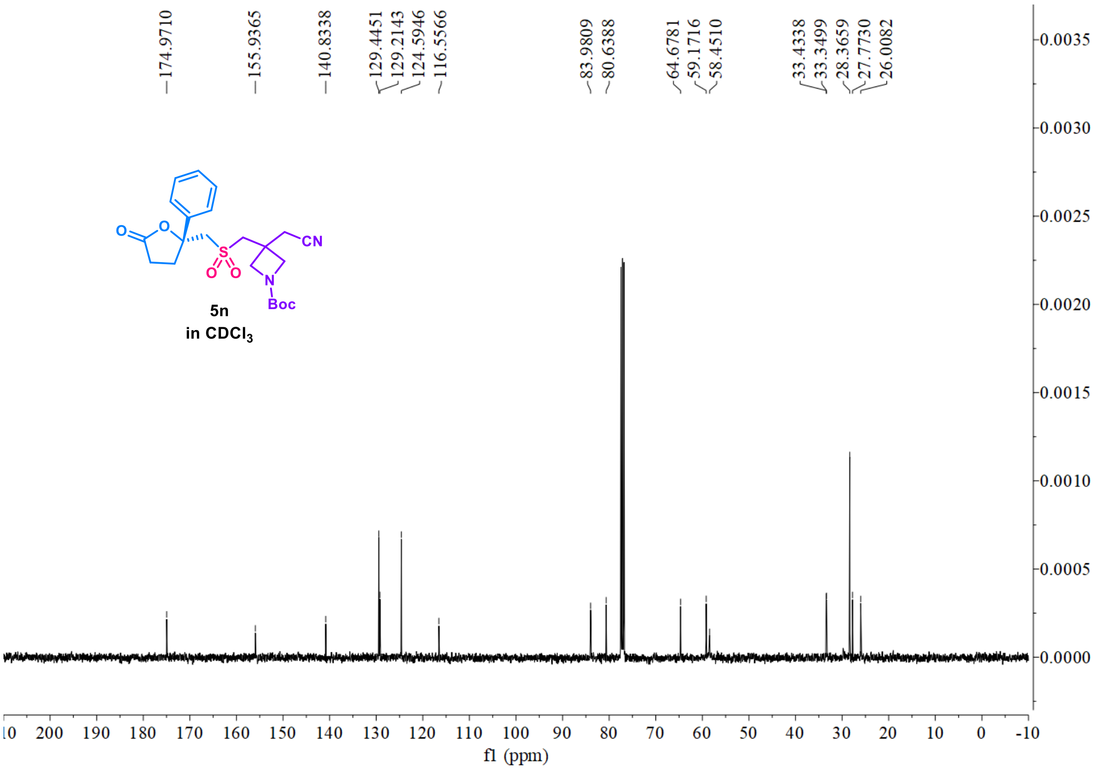
174.9710
155.9365
140.8338
129.4451
129.2143
124.5946
116.5566
83.9809
80.6388
64.6781
59.1716
58.4510
33.4338
33.3499
28.3659
27.7730
26.0082
CN
O
O
S
O O
N
Boc
5n
in CDCl3
f1 (ppm)

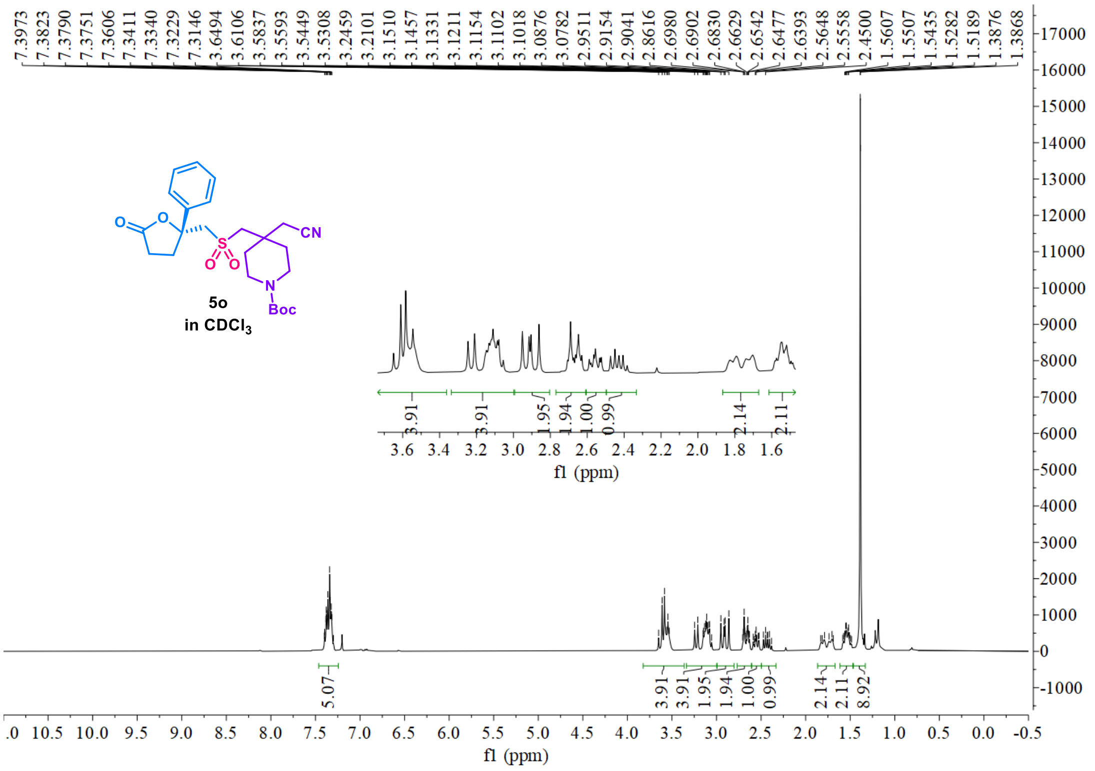

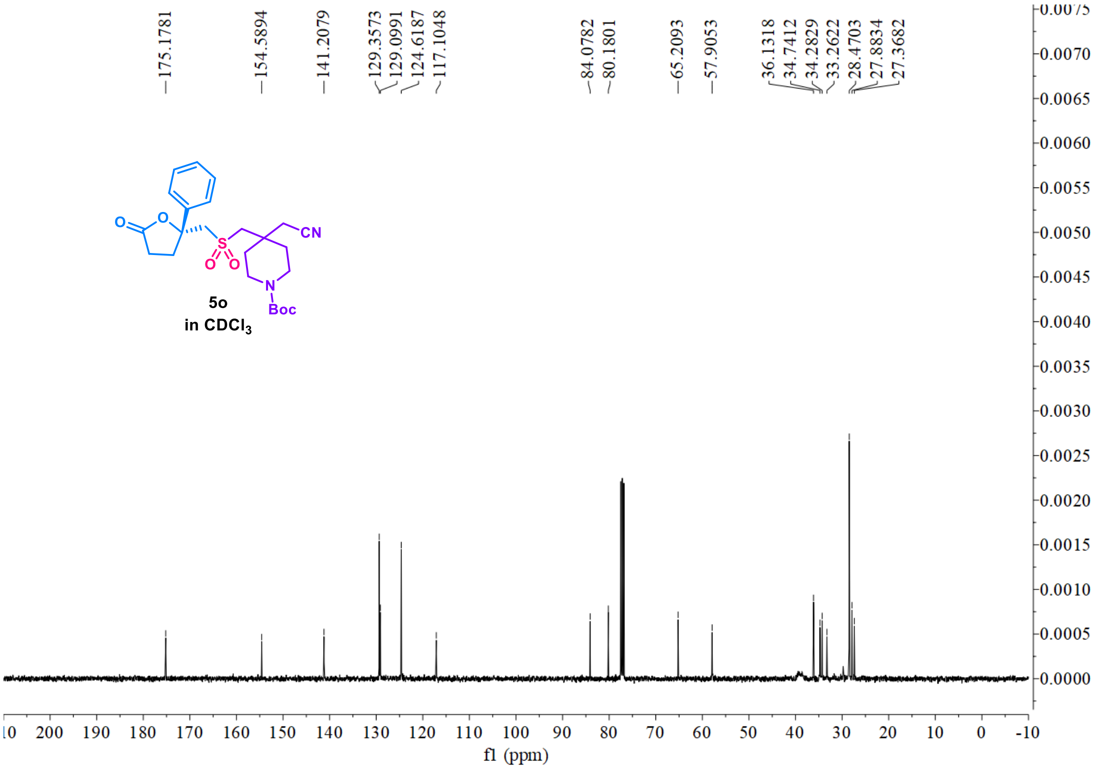

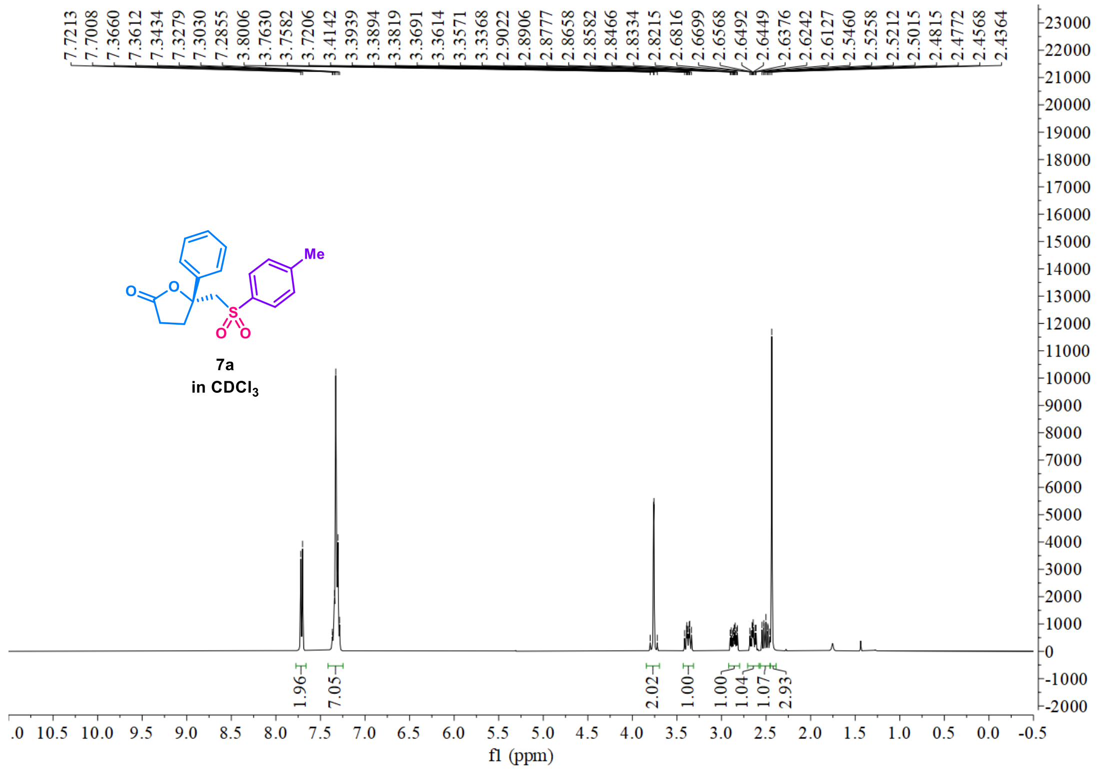
7a
in CDCl3
f1 (ppm)

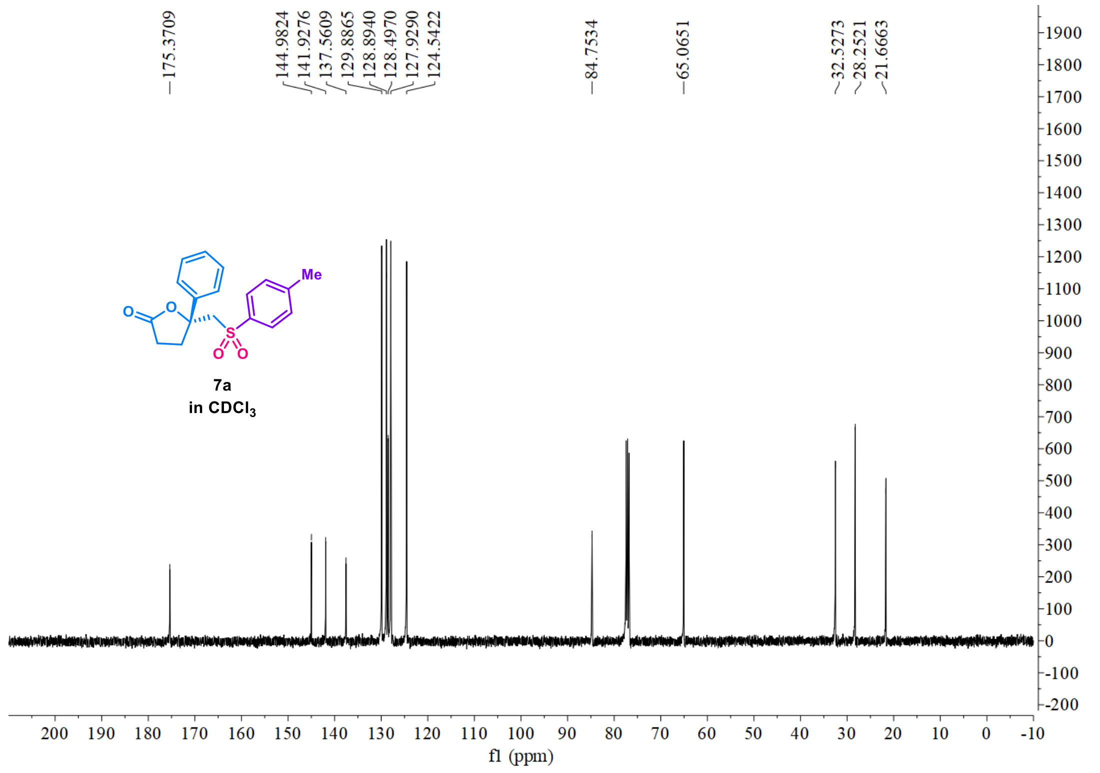
7a
in CDCl3
f1 (ppm)

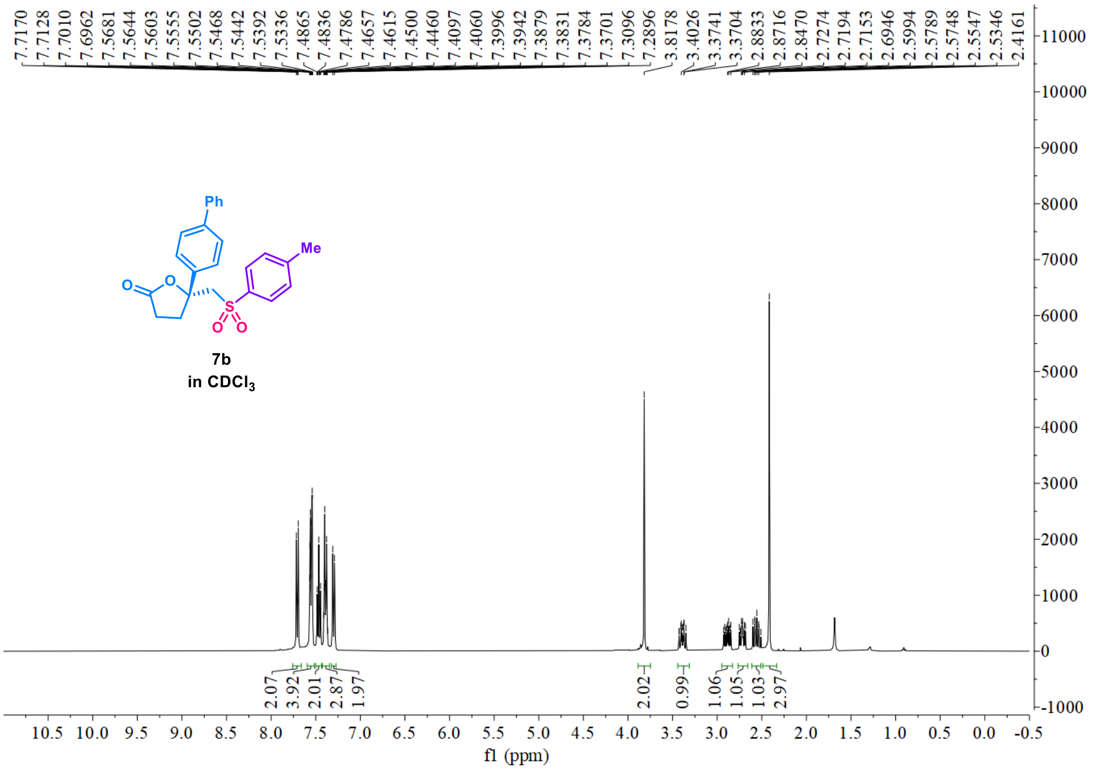
7.7170
7.7128
7.7010
7.6962
7.5681
7.5644
7.5603
7.5555
7.5502
7.5468
7.5442
7.5392
7.5336
7.4865
7.4836
7.4786
7.4657
7.4615
7.4500
7.4460
7.4097
7.4060
7.3996
7.3942
7.3879
7.3831
7.3784
7.3701
7.3096
7.2896
3.8178
3.4026
3.3741
3.3704
2.8833
2.8716
2.8470
2.7274
2.7194
2.7153
2.6946
2.5994
2.5789
2.5748
2.5547
2.5346
2.4161
Ph
Me
O
O
S
O
O
7b
in CDCl3
2.07
3.92
2.01
2.87
1.97
2.02
0.99
1.06
1.05
1.03
2.97
f1 (ppm)

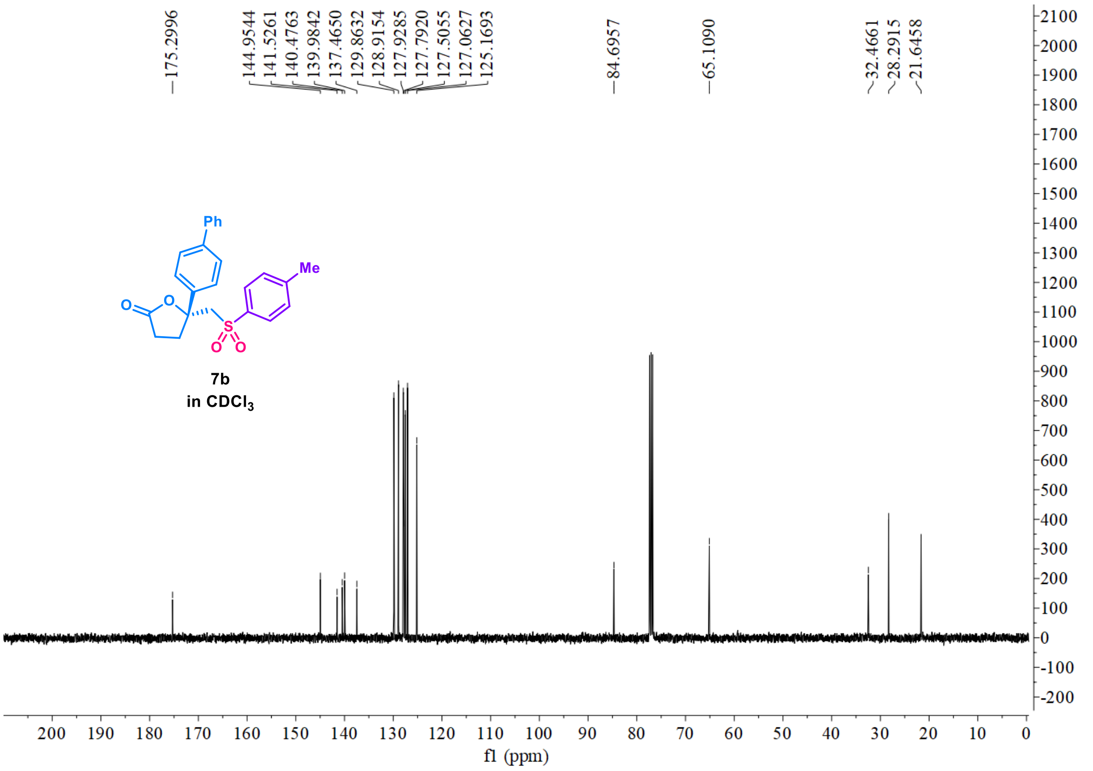
175.2996
144.9544
141.5261
140.4763
139.9842
137.4650
129.8632
128.9154
127.9285
127.7920
127.5055
127.0627
125.1693
84.6957
65.1090
32.4661
28.2915
21.6458
Ph
Me
O
O
S
O
O
7b
in CDCl3
f1 (ppm)

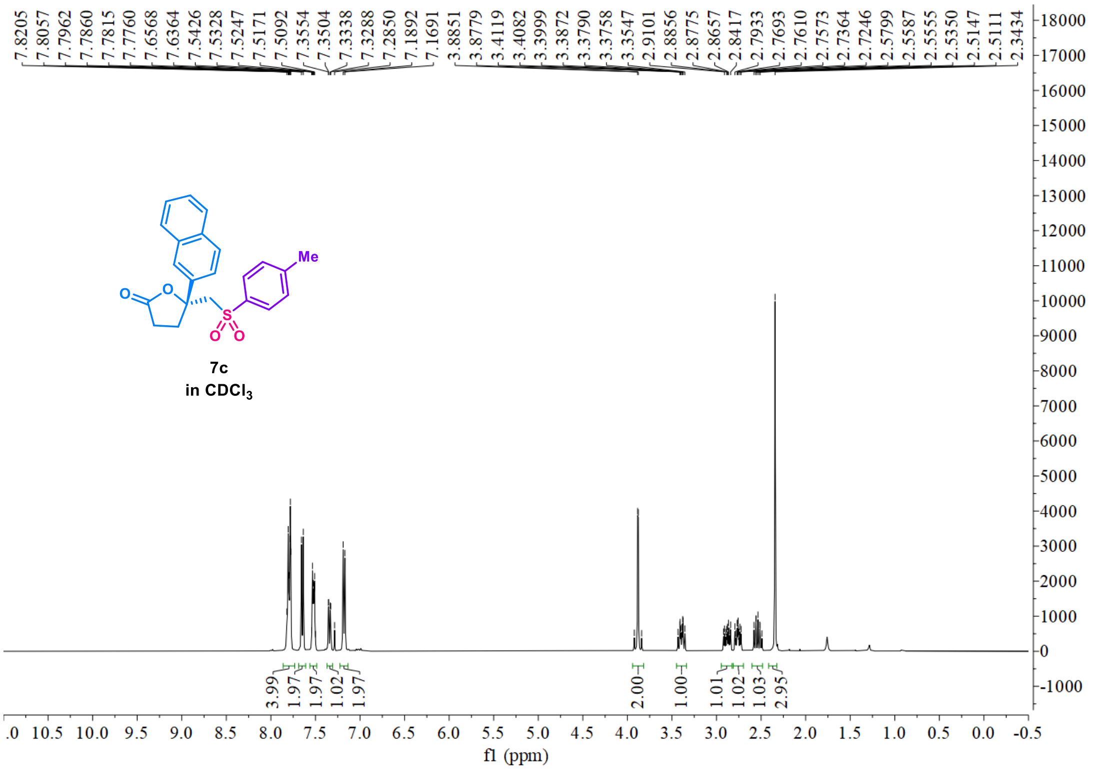
7.8205
7.8057
7.7962
7.7860
7.7815
7.7760
7.6568
7.6364
7.5426
7.5328
7.5247
7.5171
7.5092
7.3554
7.3504
7.3338
7.3288
7.2850
7.1892
7.1691
3.8851
3.8779
3.4119
3.4082
3.3999
3.3872
3.3790
3.3758
3.3547
2.9101
2.8856
2.8775
2.8657
2.8417
2.7933
2.7693
2.7610
2.7573
2.7364
2.7246
2.5799
2.5587
2.5555
2.5350
2.5147
2.5111
2.3434
Me
O
O
S
O
O
7c
in CDCl3
3.99
1.97
1.97
1.02
1.97
2.00
1.00
1.01
1.02
1.03
2.95
f1 (ppm)

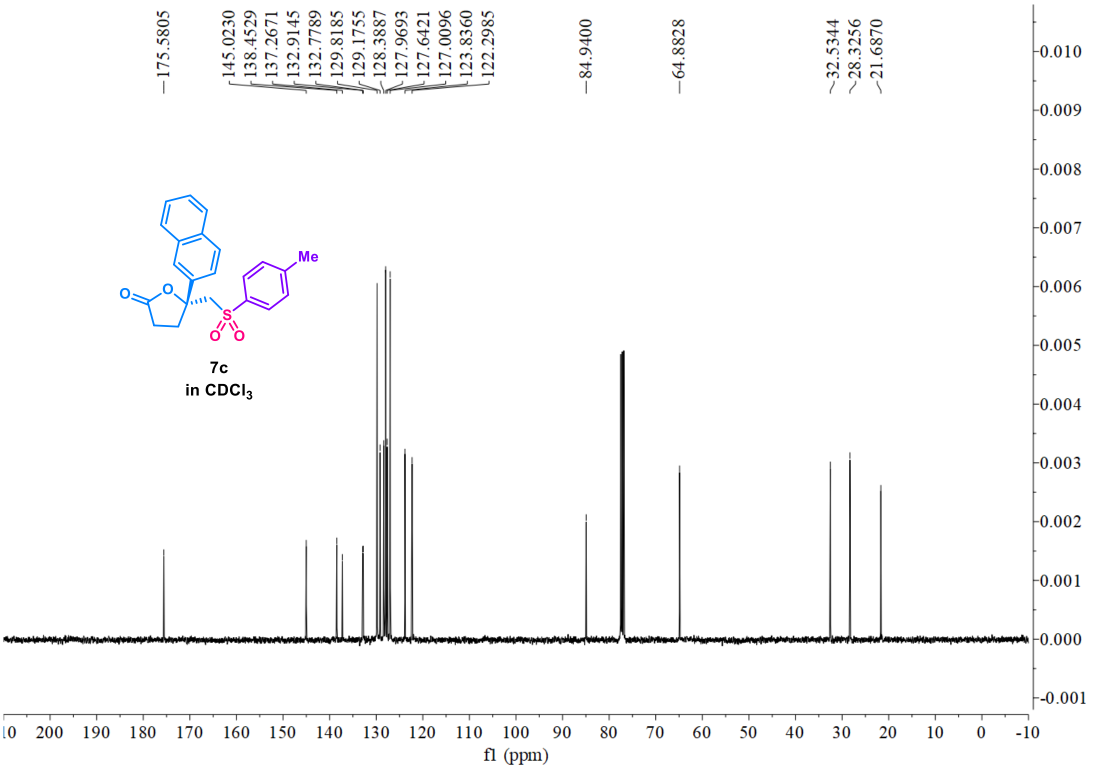
175.5805
145.0230
138.4529
137.2671
132.9145
132.7789
129.8185
129.1755
128.3887
127.9693
127.6421
127.0096
123.8360
122.2985
84.9400
64.8828
32.5344
28.3256
21.6870
Me
O
O
S
O
O
7c
in CDCl3
f1 (ppm)

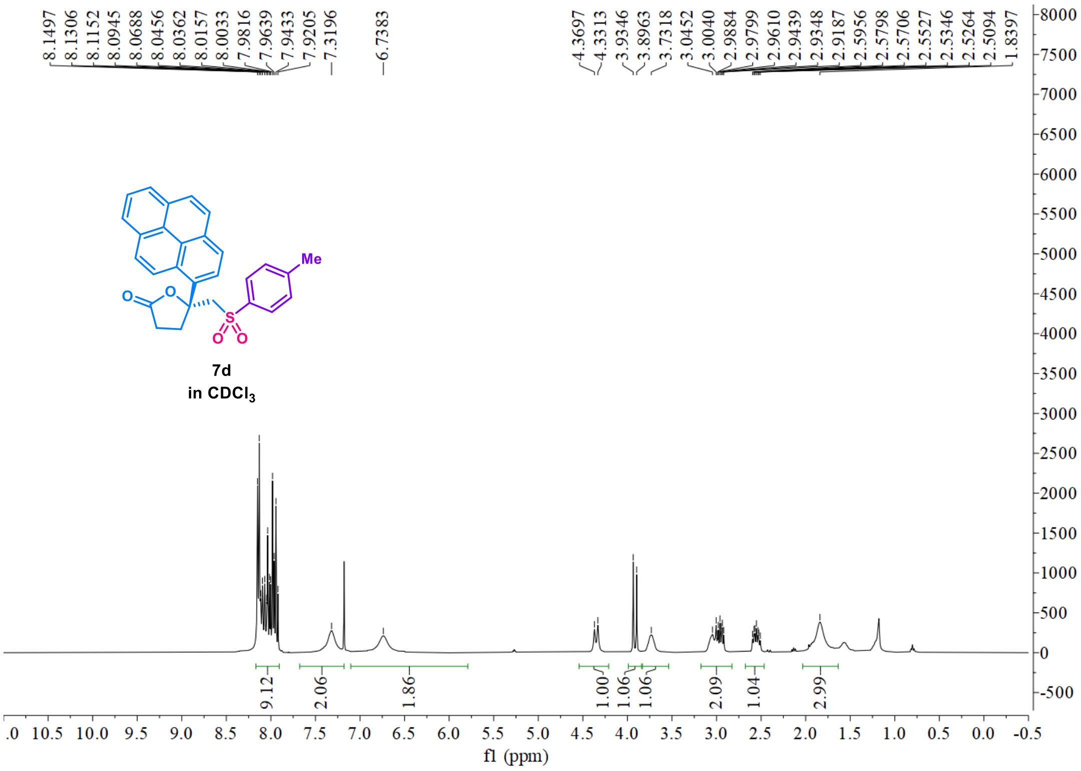
8.1497
8.1306
8.1152
8.0945
8.0688
8.0456
8.0362
8.0157
8.0033
7.9816
7.9639
7.9433
7.9205
7.3196
6.7383
4.3697
4.3313
3.9346
3.8963
3.7318
3.0452
3.0040
2.9884
2.9799
2.9610
2.9439
2.9348
2.9187
2.5956
2.5798
2.5706
2.5527
2.5346
2.5264
2.5094
1.8397
Me
7d
in CDCl3
9.12
2.06
1.86
1.00
1.06
1.06
2.09
1.04
2.99
f1 (ppm)

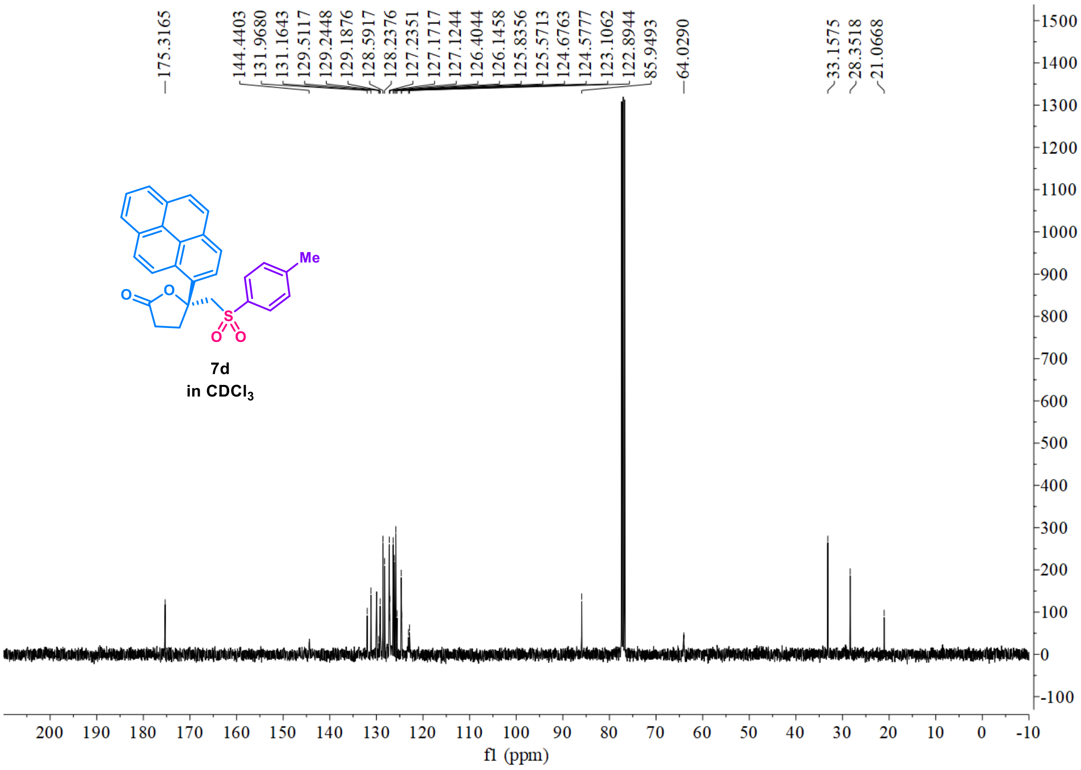
175.3165
144.4403
131.9680
131.1643
129.5117
129.2448
129.1876
128.5917
128.2376
127.2351
127.1717
127.1244
126.4044
126.1458
125.8356
125.5713
124.6763
124.5777
123.1062
122.8944
85.9493
64.0290
33.1575
28.3518
21.0668
Me
7d
in CDCl3
f1 (ppm)

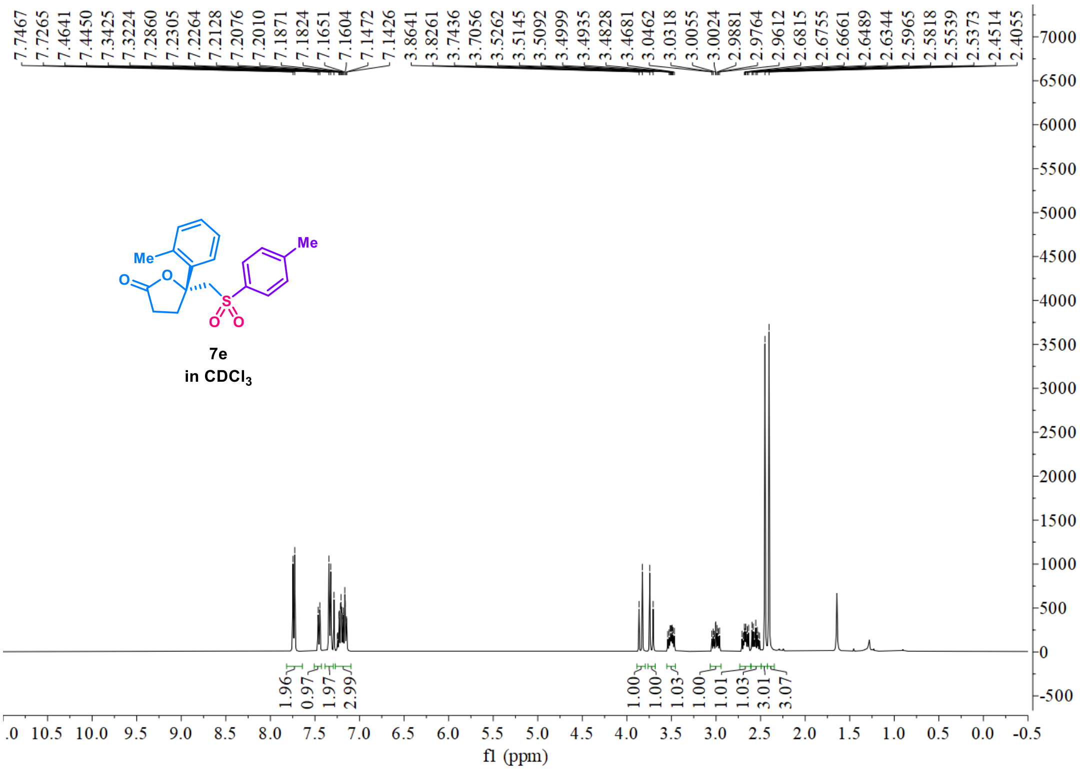
7.7467 7.7265 7.4641 7.4450 7.3425 7.3224 7.2860 7.2305 7.2264 7.2128 7.2076 7.2010 7.1871 7.1824 7.1651 7.1604 7.1472 7.1426
3.8641 3.8261 3.7436 3.7056 3.5262 3.5145 3.5092 3.4999 3.4935 3.4828 3.4681 3.0462 3.0318 3.0055 3.0024 2.9881 2.9764 2.9612 2.6815 2.6755 2.6661 2.6489 2.6344 2.5965 2.5818 2.5539 2.5373 2.4514 2.4055
Me
O
Me
S
O
O
7e
in $CDCl_3$
1.96 0.97 1.97 2.99
1.00 1.00 1.03 1.00 1.01 1.03 3.01 3.07
f1 (ppm)

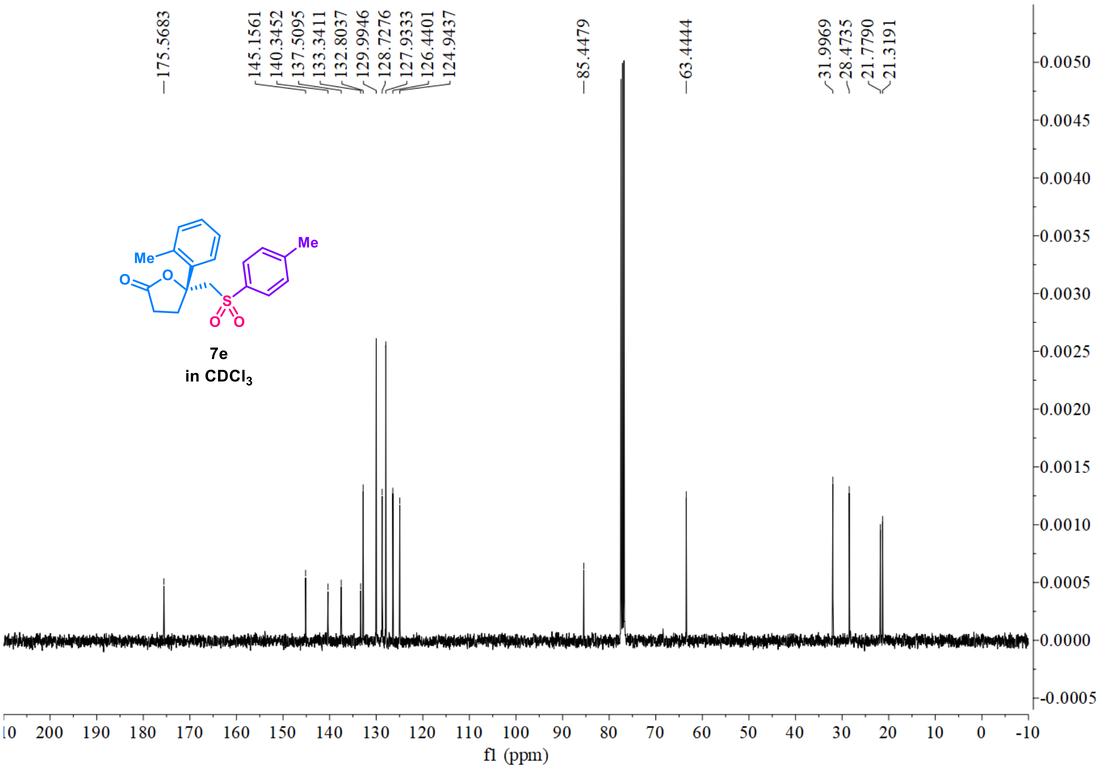
175.5683
145.1561 140.3452 137.5095 133.3411 132.8037 129.9946 128.7276 127.9333 126.4401 124.9437
85.4479
63.4444
31.9969 28.4735 21.7790 21.3191
Me
O
Me
S
O
O
7e
in $CDCl_3$
f1 (ppm)

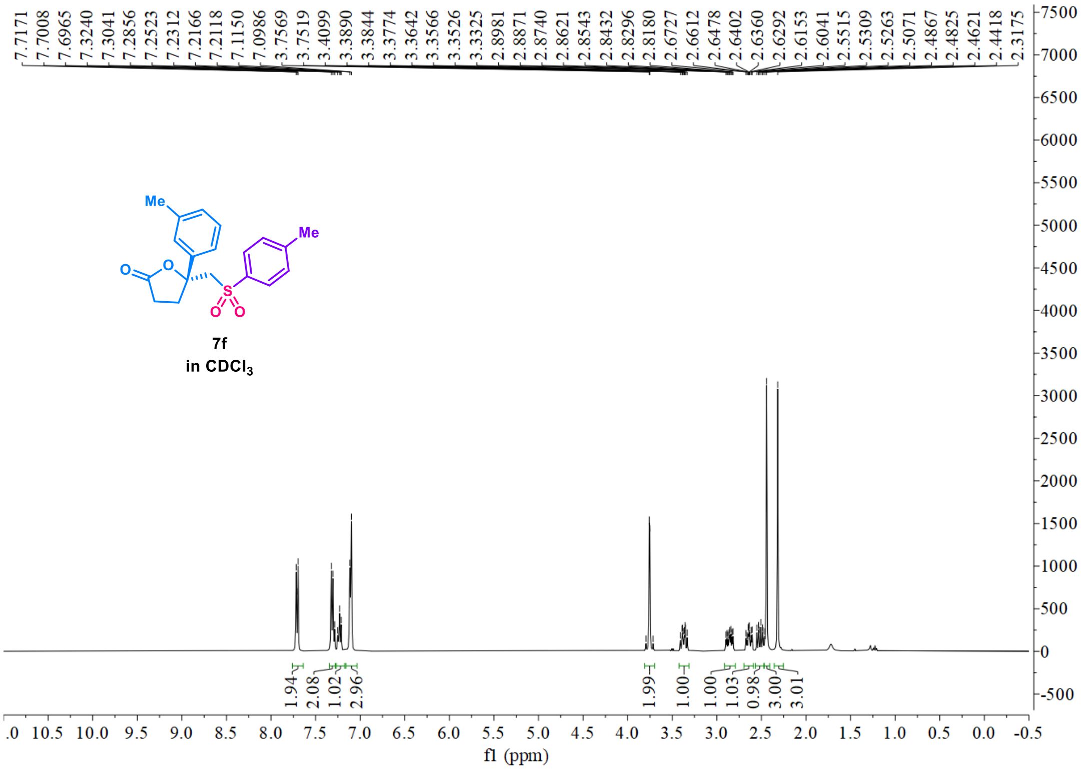
7f
in CDCl3
f1 (ppm)

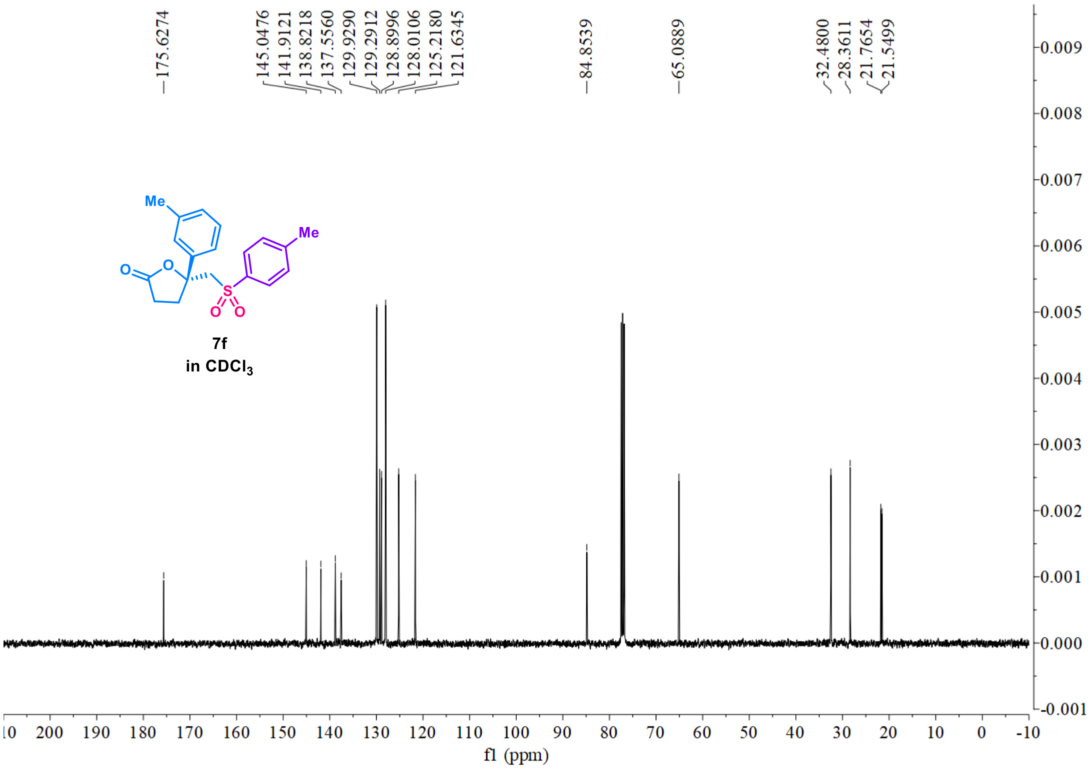
7f
in CDCl3
f1 (ppm)

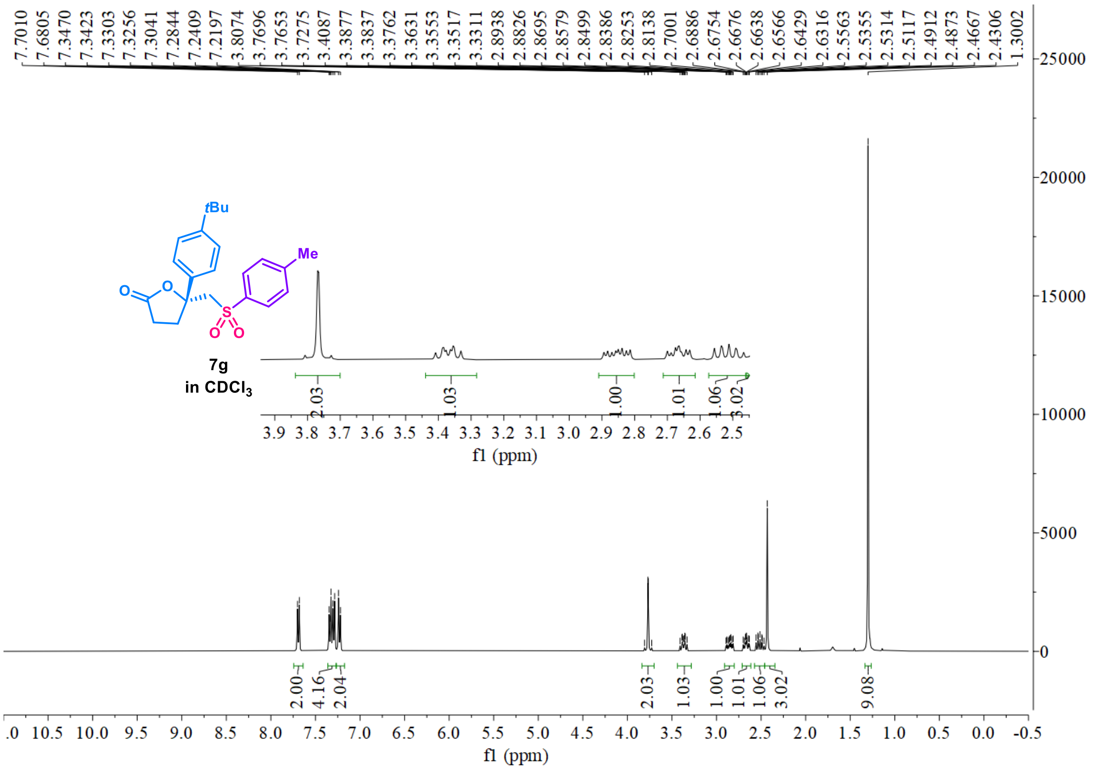
7.7010
7.6805
7.3470
7.3423
7.3303
7.3256
7.3041
7.2844
7.2409
7.2197
3.8074
3.7696
3.7653
3.7275
3.4087
3.3877
3.3837
3.3762
3.3631
3.3553
3.3517
3.3311
2.8938
2.8826
2.8695
2.8579
2.8499
2.8386
2.8253
2.8138
2.7001
2.6886
2.6754
2.6676
2.6638
2.6566
2.6429
2.6316
2.5563
2.5355
2.5314
2.5117
2.4912
2.4873
2.4667
2.4306
1.3002
tBu
Me
O
O
S
O
O
7g
in CDCl3
2.03
1.03
1.00
1.01
1.06
3.02
f1 (ppm)
2.00
4.16
2.04
9.08
f1 (ppm)

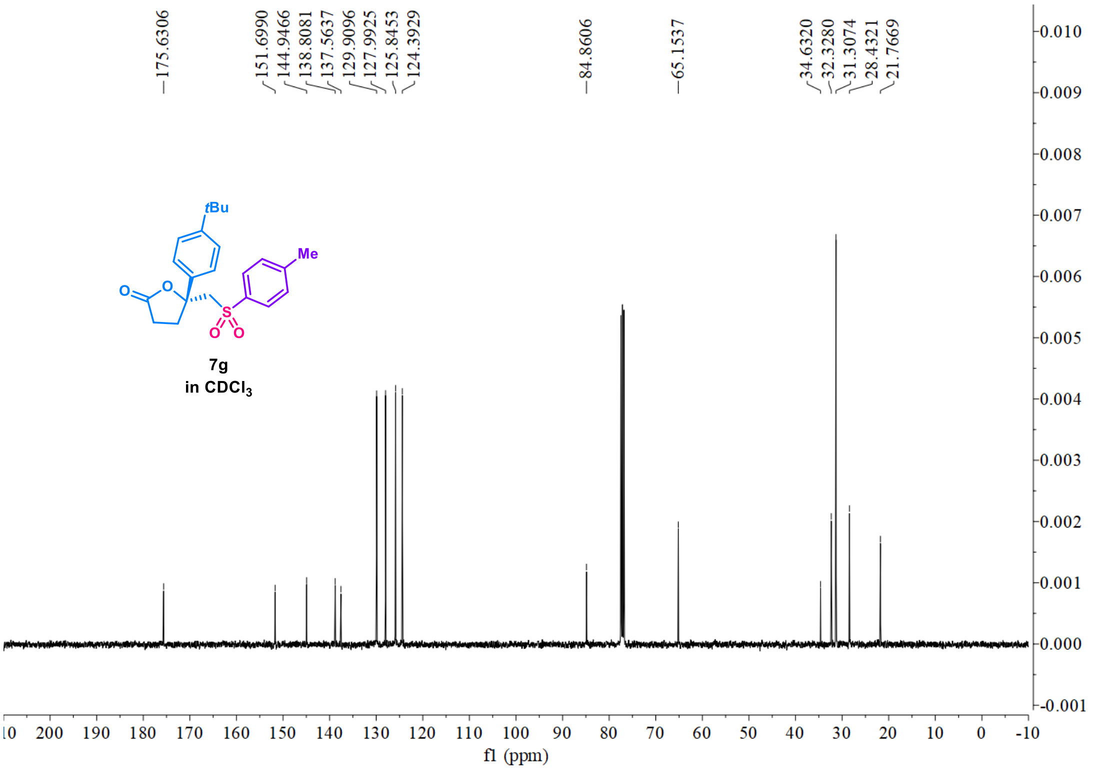
175.6306
151.6990
144.9466
138.8081
137.5637
129.9096
127.9925
125.8453
124.3929
84.8606
65.1537
34.6320
32.3280
31.3074
28.4321
21.7669
tBu
Me
O
O
S
O
O
7g
in CDCl3
f1 (ppm)

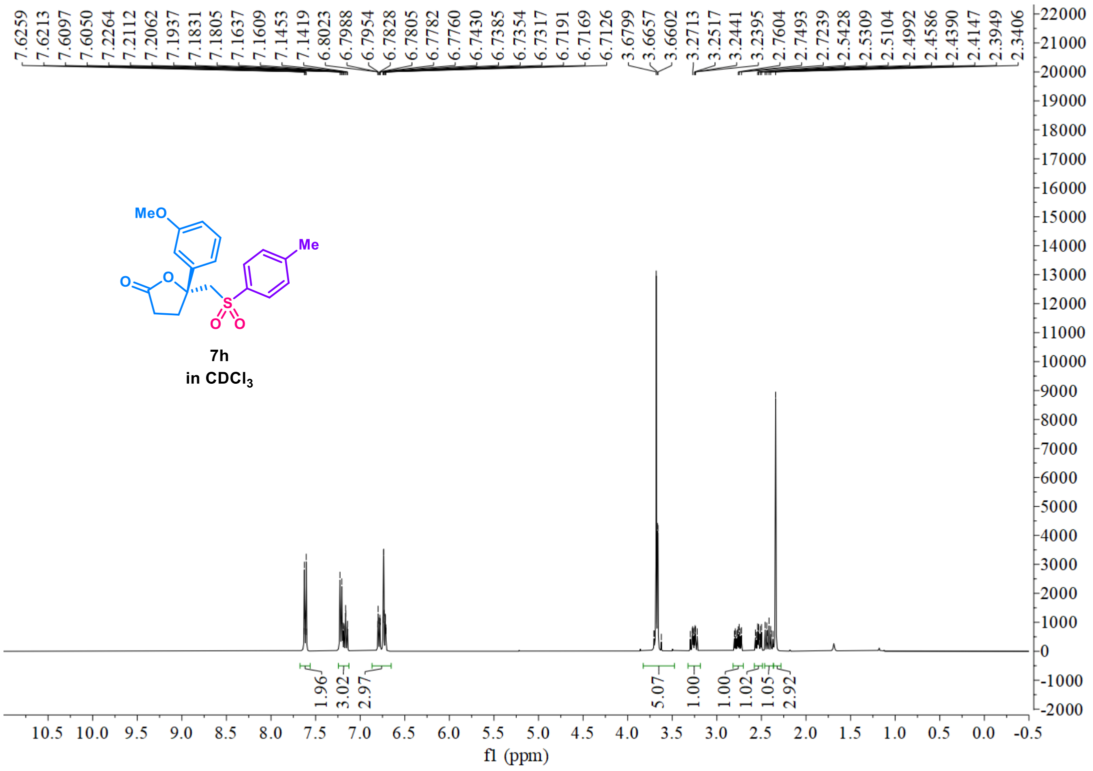
7.6259
7.6213
7.6097
7.6050
7.2264
7.2112
7.2062
7.1937
7.1831
7.1805
7.1637
7.1609
7.1453
7.1419
6.8023
6.7988
6.7954
6.7828
6.7805
6.7782
6.7760
6.7430
6.7385
6.7354
6.7317
6.7191
6.7169
6.7126
3.6799
3.6657
3.6602
3.2713
3.2517
3.2441
3.2395
2.7604
2.7493
2.7239
2.5428
2.5309
2.5104
2.4992
2.4586
2.4390
2.4147
2.3949
2.3406
MeO
Me
O
O
S
O
O
7h
in CDCl3
1.96
3.02
2.97
5.07
1.00
1.00
1.02
1.05
2.92
f1 (ppm)

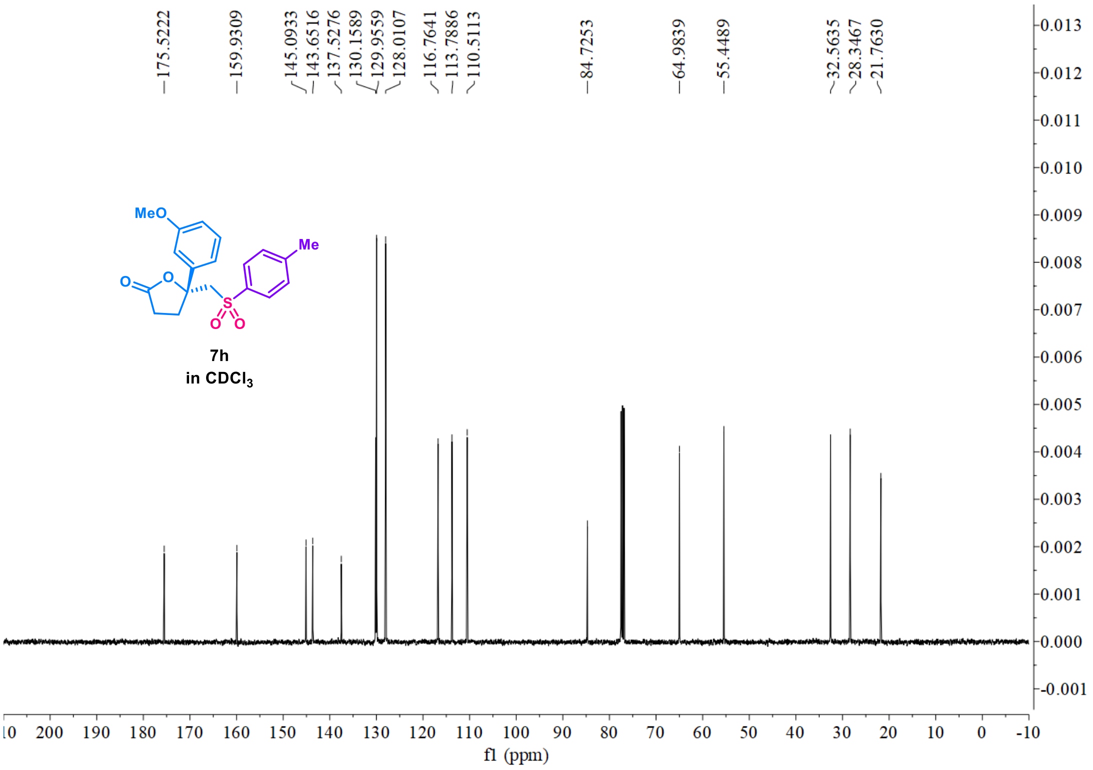
175.5222
159.9309
145.0933
143.6516
137.5276
130.1589
129.9559
128.0107
116.7641
113.7886
110.5113
84.7253
64.9839
55.4489
32.5635
28.3467
21.7630
MeO
Me
O
O
S
O
O
7h
in CDCl3
f1 (ppm)

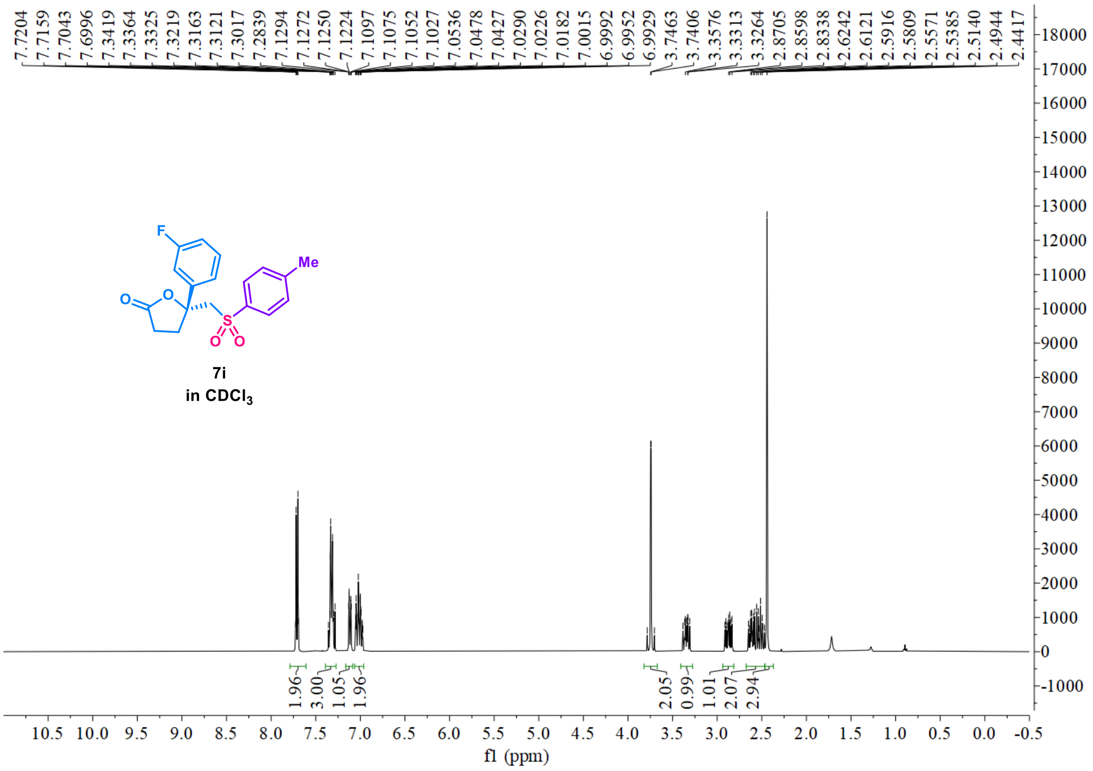
7.7204
7.7159
7.7043
7.6996
7.3419
7.3364
7.3325
7.3219
7.3163
7.3121
7.3017
7.2839
7.1294
7.1272
7.1250
7.1224
7.1097
7.1075
7.1052
7.1027
7.0536
7.0478
7.0427
7.0290
7.0226
7.0182
7.0015
6.9992
6.9952
6.9929
3.7463
3.7406
3.3576
3.3313
3.3264
2.8705
2.8598
2.8338
2.6242
2.6121
2.5916
2.5809
2.5571
2.5385
2.5140
2.4944
2.4417
F
Me
O
O
S
O
O
7i
in CDCl3
1.96
3.00
1.05
1.96
2.05
0.99
1.01
2.07
2.94
f1 (ppm)

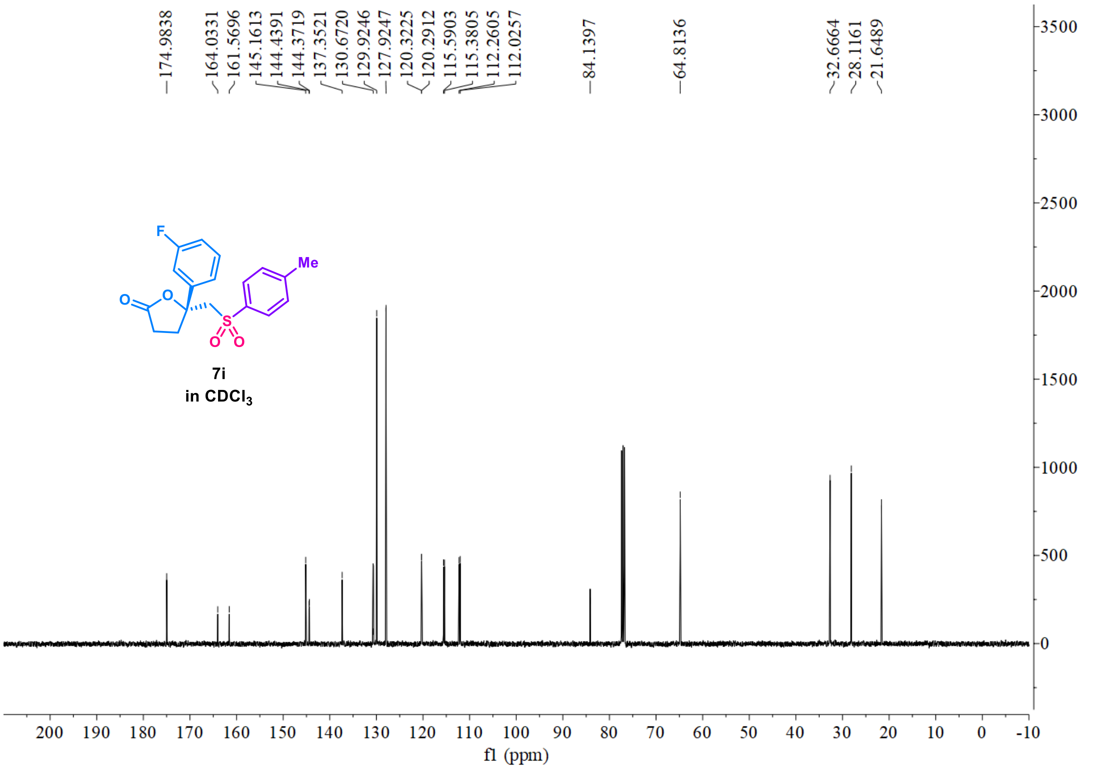
174.9838
164.0331
161.5696
145.1613
144.4391
144.3719
137.3521
130.6720
129.9246
127.9247
120.3225
120.2912
115.5903
115.3805
112.2605
112.0257
84.1397
64.8136
32.6664
28.1161
21.6489
F
Me
O
O
S
O
O
7i
in CDCl3
f1 (ppm)

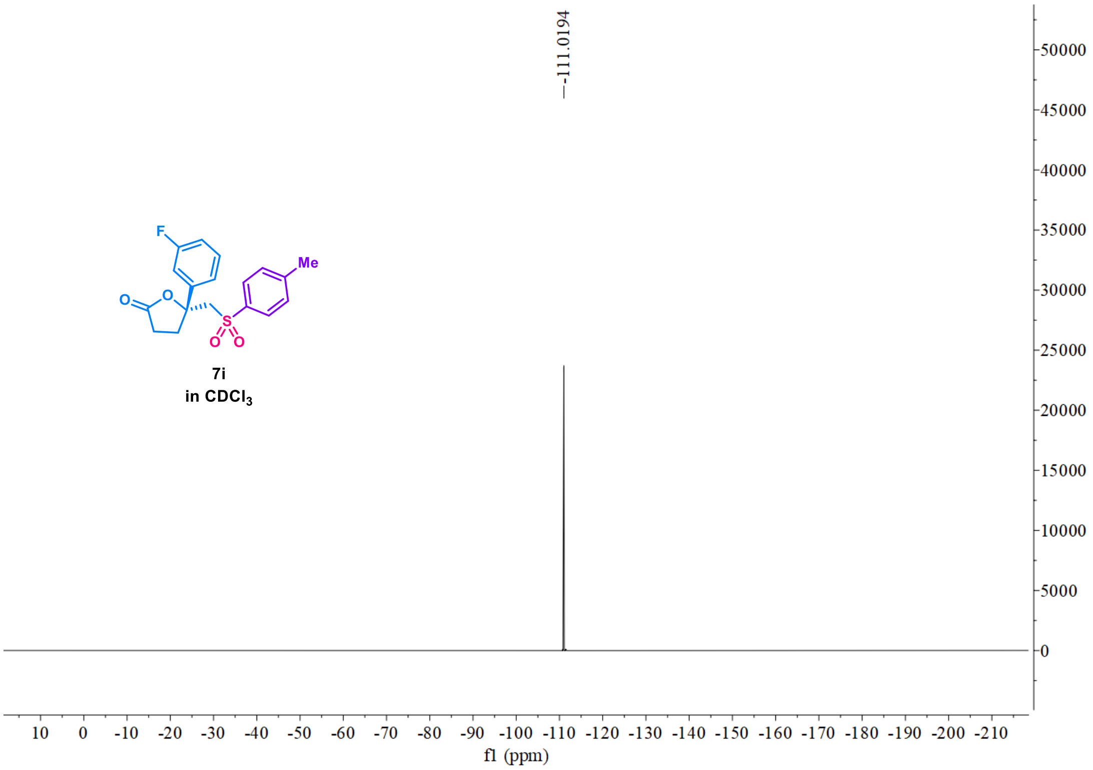

-111.0194
7i
in CDCl3
f1 (ppm)

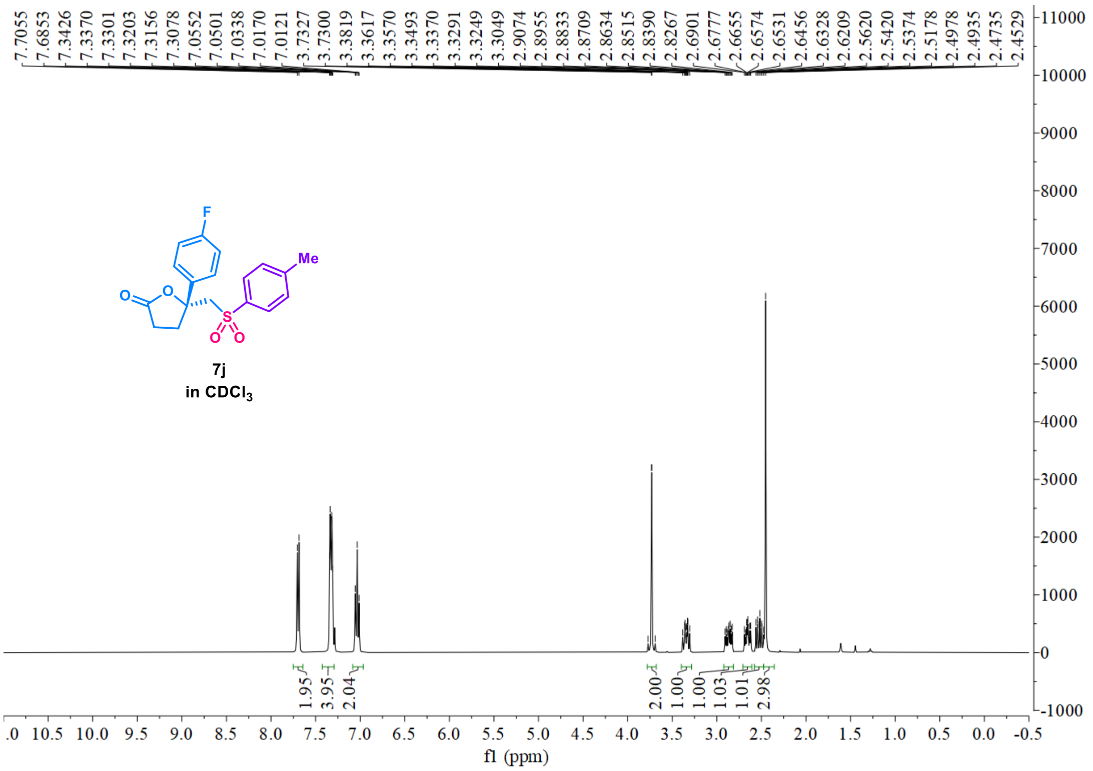

7.7055
7.6853
7.3426
7.3370
7.3301
7.3203
7.3156
7.3078
7.0552
7.0501
7.0338
7.0170
7.0121
3.7327
3.7300
3.3819
3.3617
3.3570
3.3493
3.3370
3.3291
3.3249
3.3049
2.9074
2.8955
2.8833
2.8709
2.8634
2.8515
2.8390
2.8267
2.6901
2.6777
2.6655
2.6574
2.6531
2.6456
2.6328
2.6209
2.5620
2.5420
2.5374
2.5178
2.4978
2.4935
2.4735
2.4529
7j
in CDCl3
1.95
3.95
2.04
2.00
1.00
1.00
1.03
1.01
2.98
f1 (ppm)

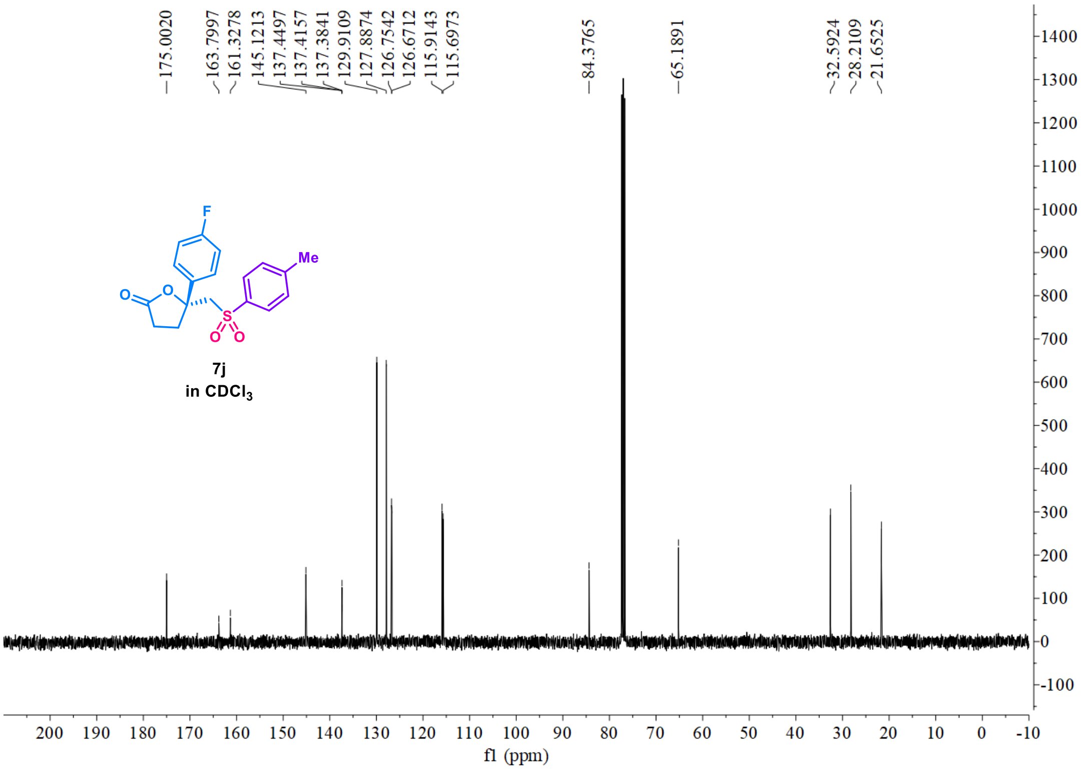

175.0020
163.7997
161.3278
145.1213
137.4497
137.4157
137.3841
129.9109
127.8874
126.7542
126.6712
115.9143
115.6973
84.3765
65.1891
32.5924
28.2109
21.6525
F
Me
O
O
S
O
O
7j
in $CDCl_3$
f1 (ppm)

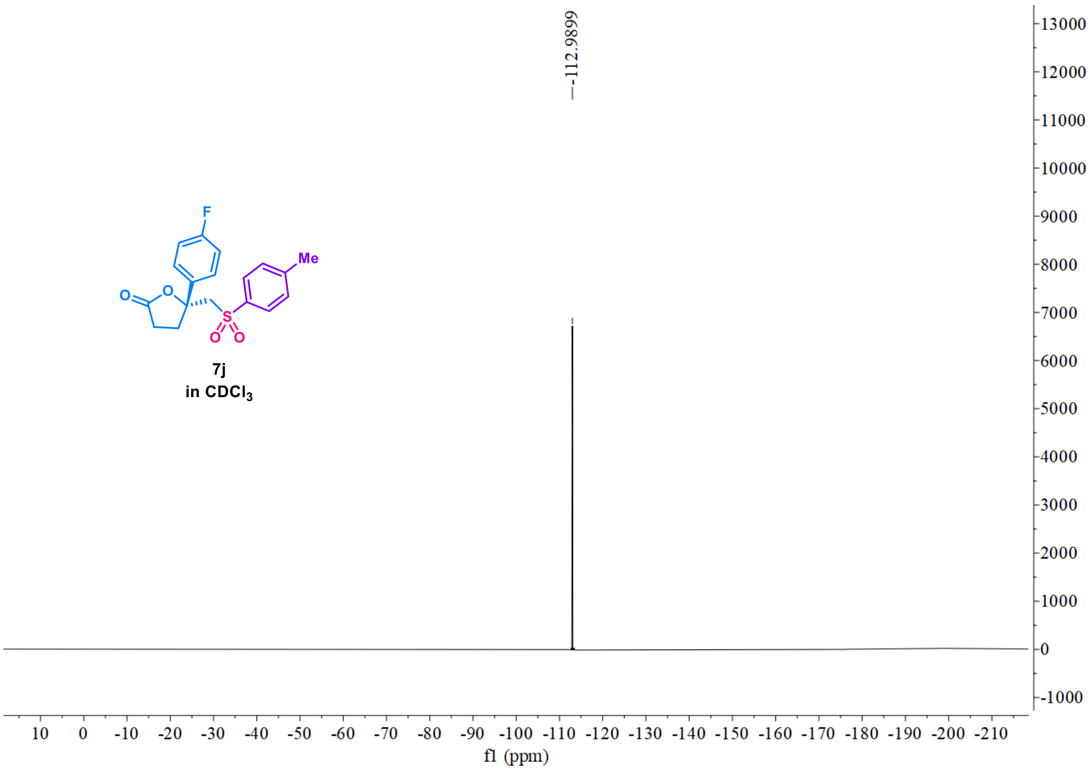

-112.9899
F
Me
O
O
S
O
O
7j
in $CDCl_3$
f1 (ppm)

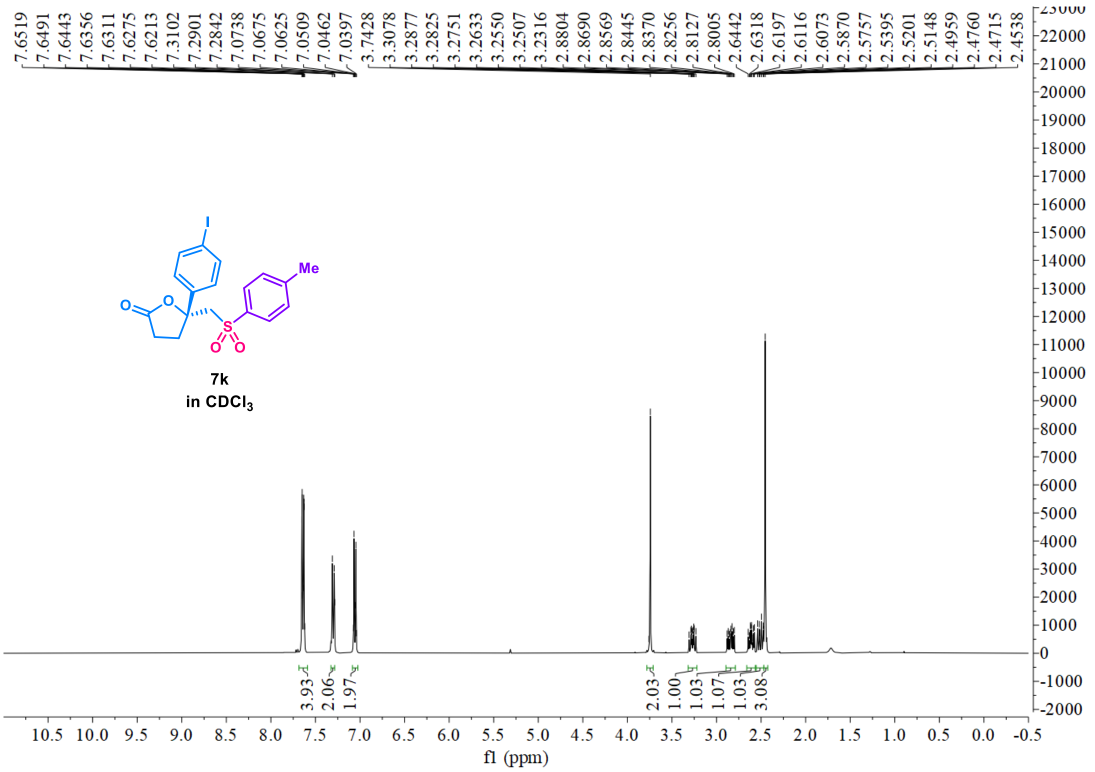
7k
in CDCl3
f1 (ppm)

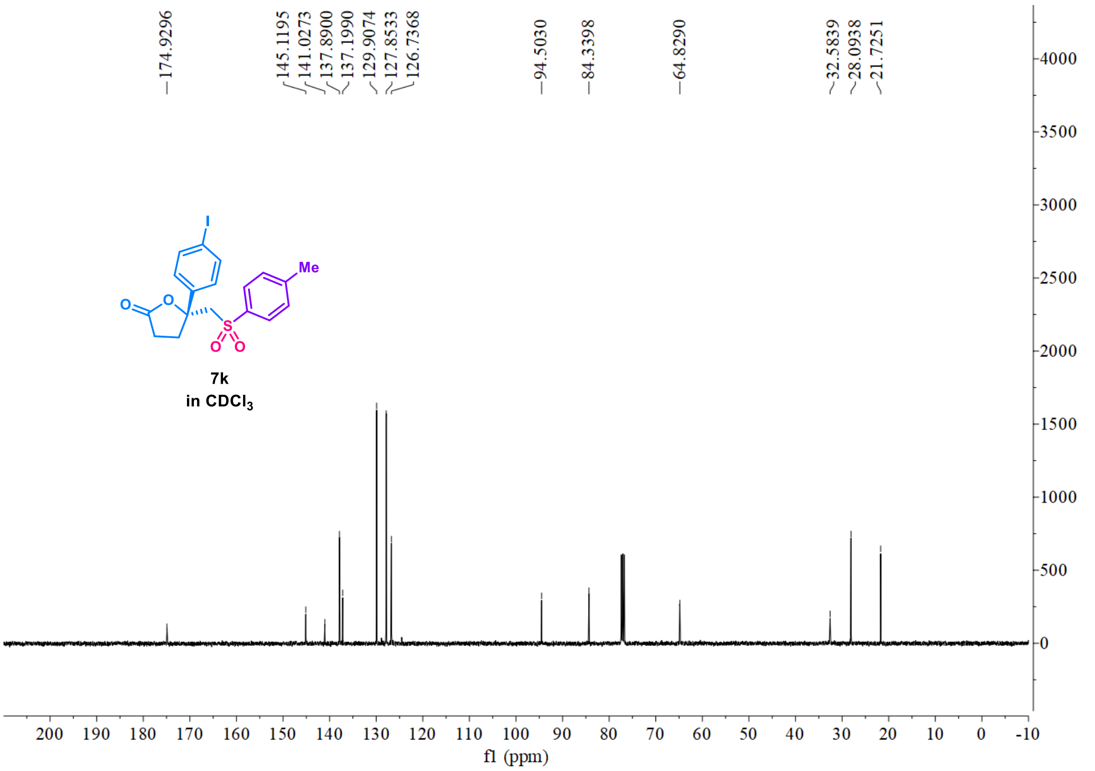
7k
in CDCl3
f1 (ppm)

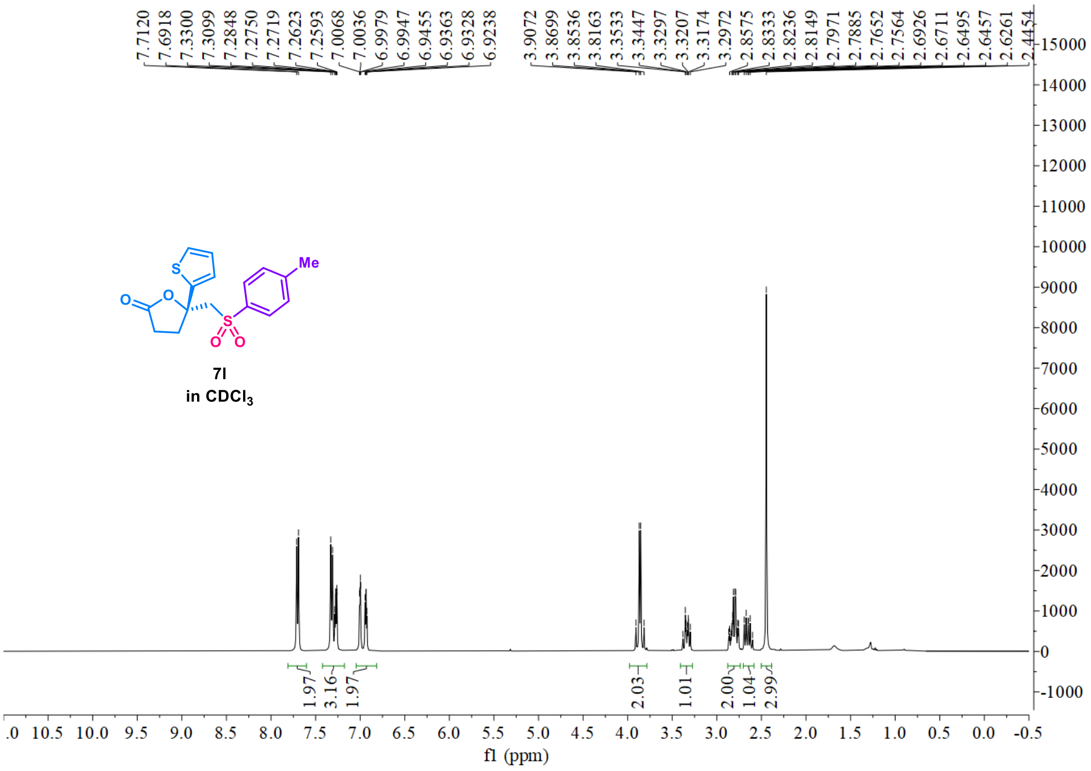

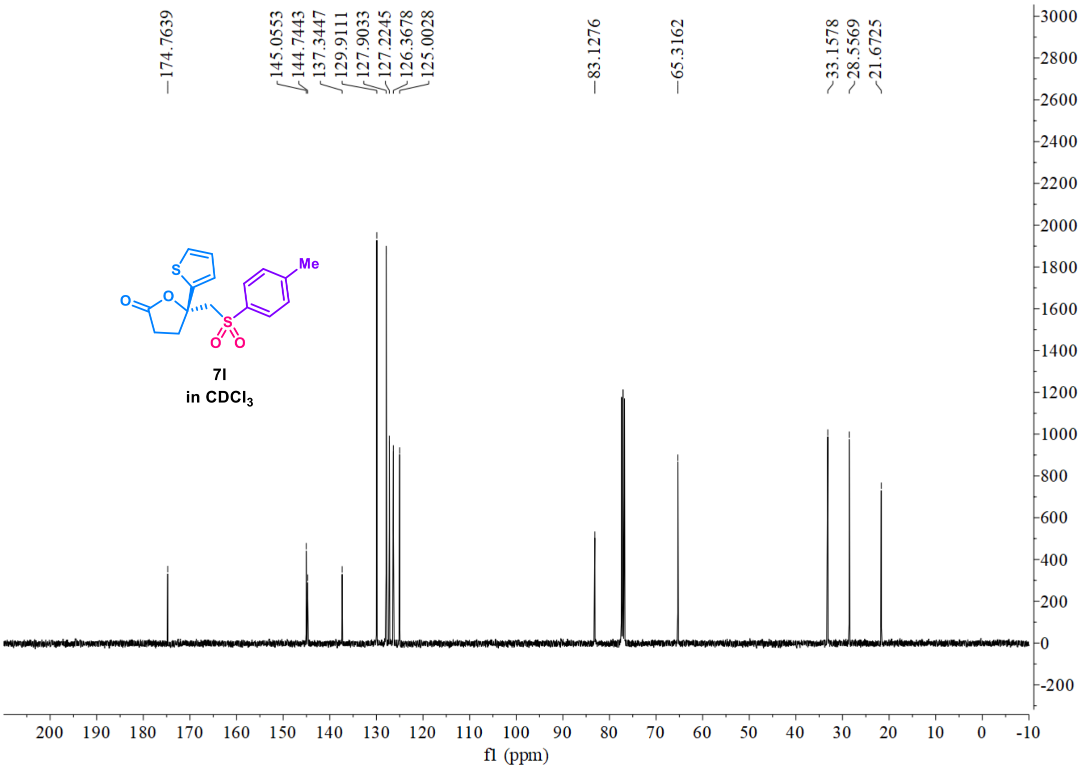

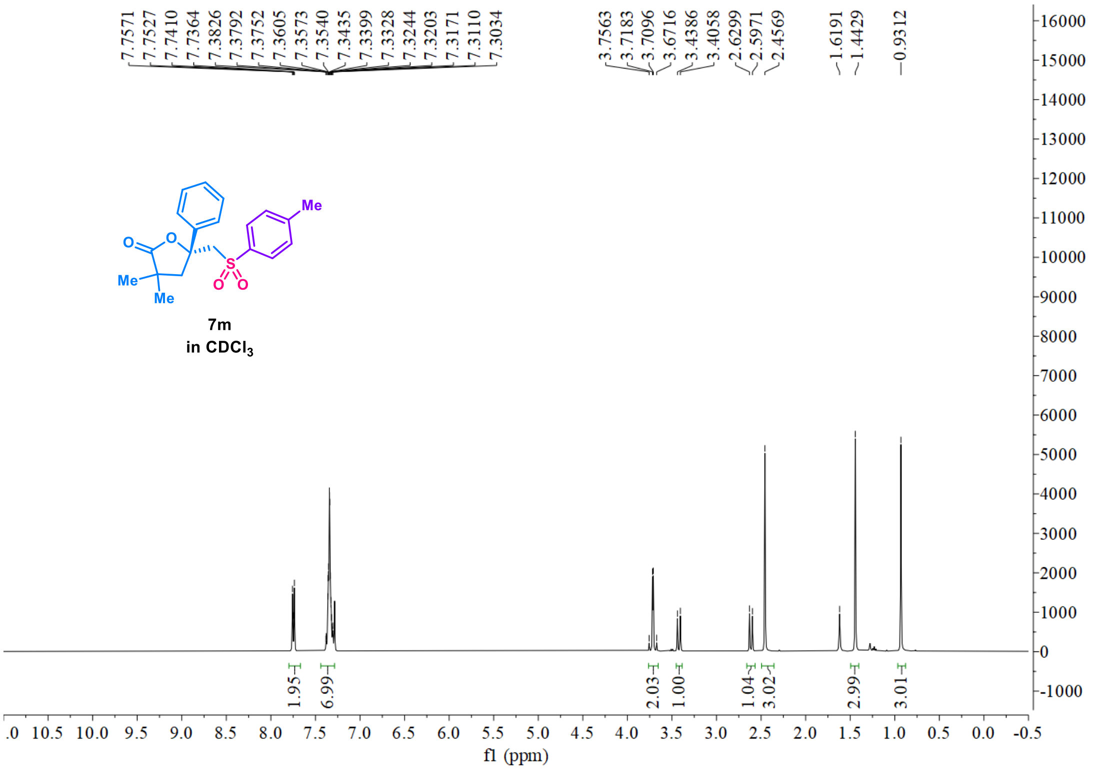
7.7571
7.7527
7.7410
7.7364
7.3826
7.3792
7.3752
7.3605
7.3573
7.3540
7.3435
7.3399
7.3328
7.3244
7.3203
7.3171
7.3110
7.3034
3.7563
3.7183
3.7096
3.6716
3.4386
3.4058
2.6299
2.5971
2.4569
1.6191
1.4429
0.9312
Me
O
O
Me
Me
S
O
O
7m
in CDCl3
1.95
6.99
2.03
1.00
1.04
3.02
2.99
3.01
f1 (ppm)

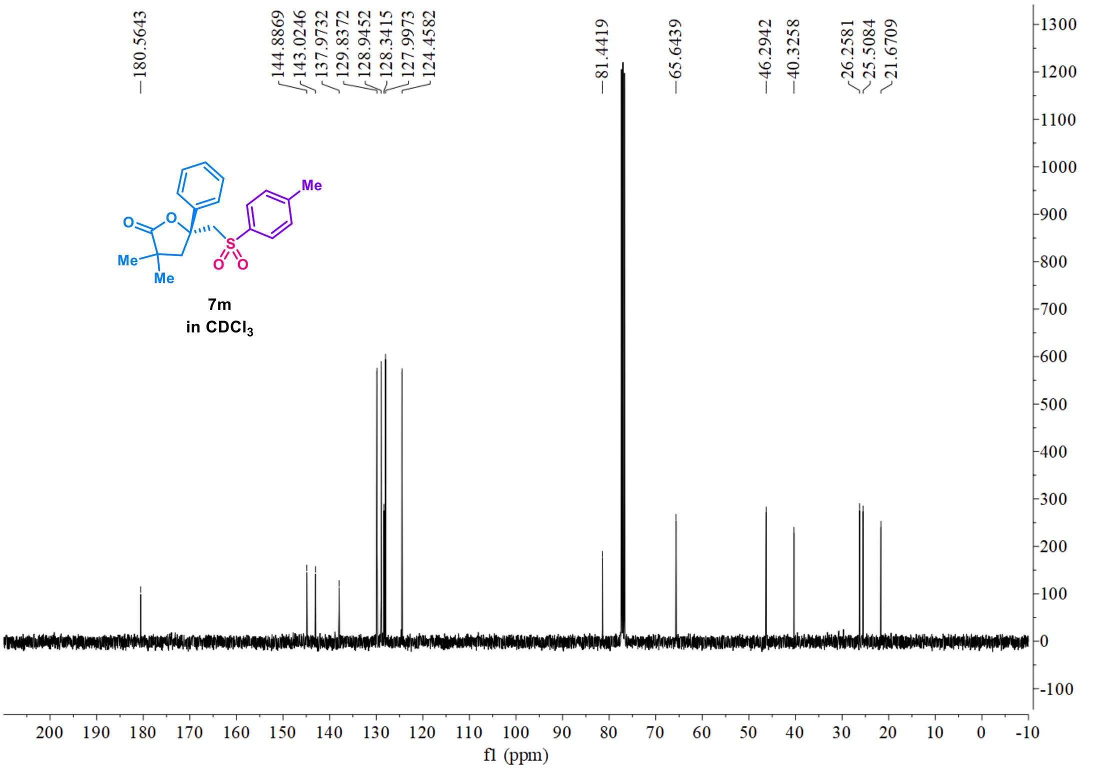
180.5643
144.8869
143.0246
137.9732
129.8372
128.9452
128.3415
127.9973
124.4582
81.4419
65.6439
46.2942
40.3258
26.2581
25.5084
21.6709
Me
O
O
Me
Me
S
O
O
7m
in CDCl3
f1 (ppm)

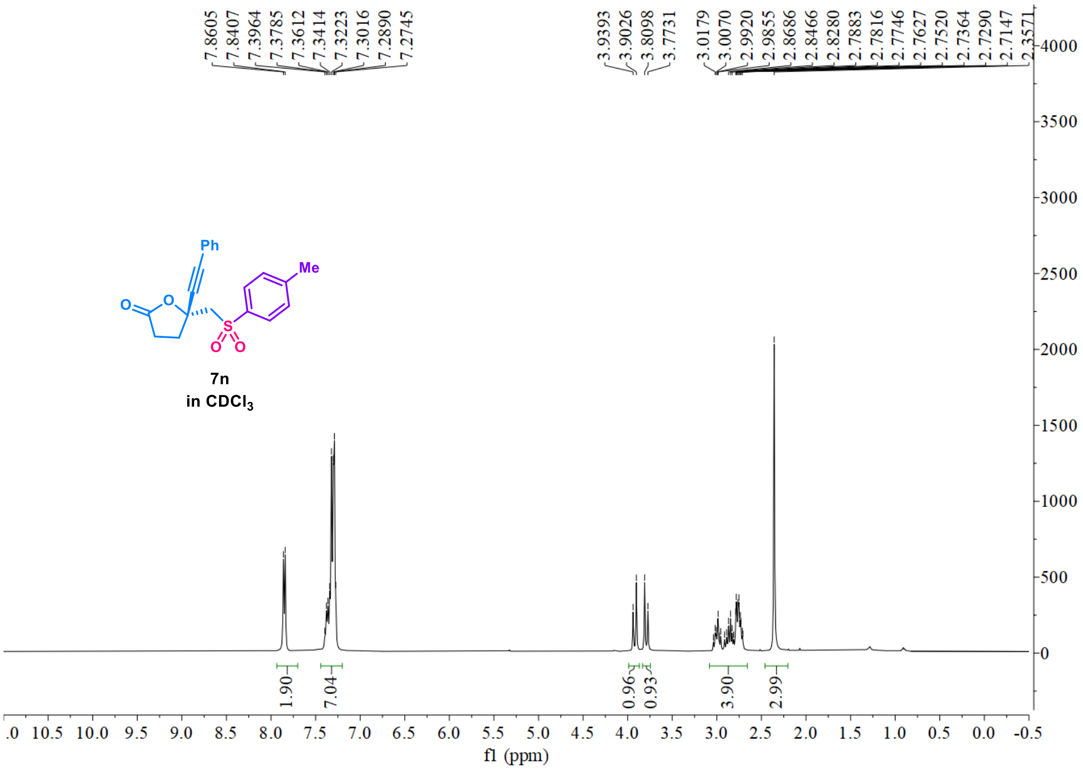

7n
in CDCl3
f1 (ppm)

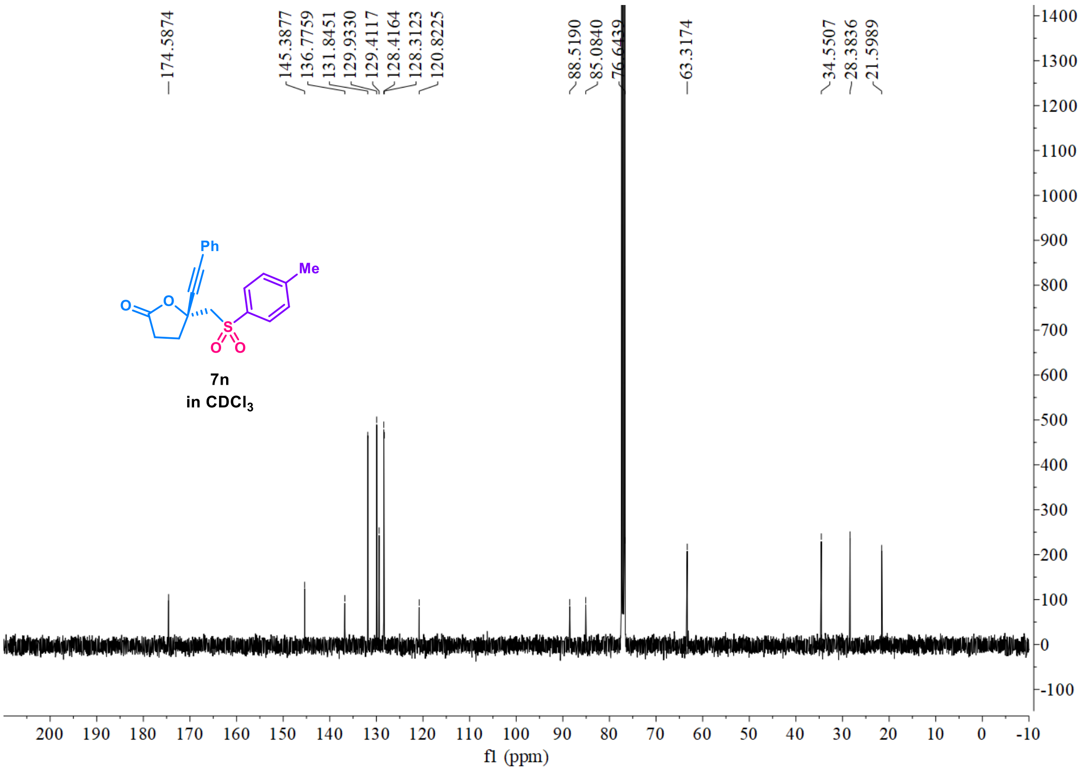

7n
in CDCl3
f1 (ppm)

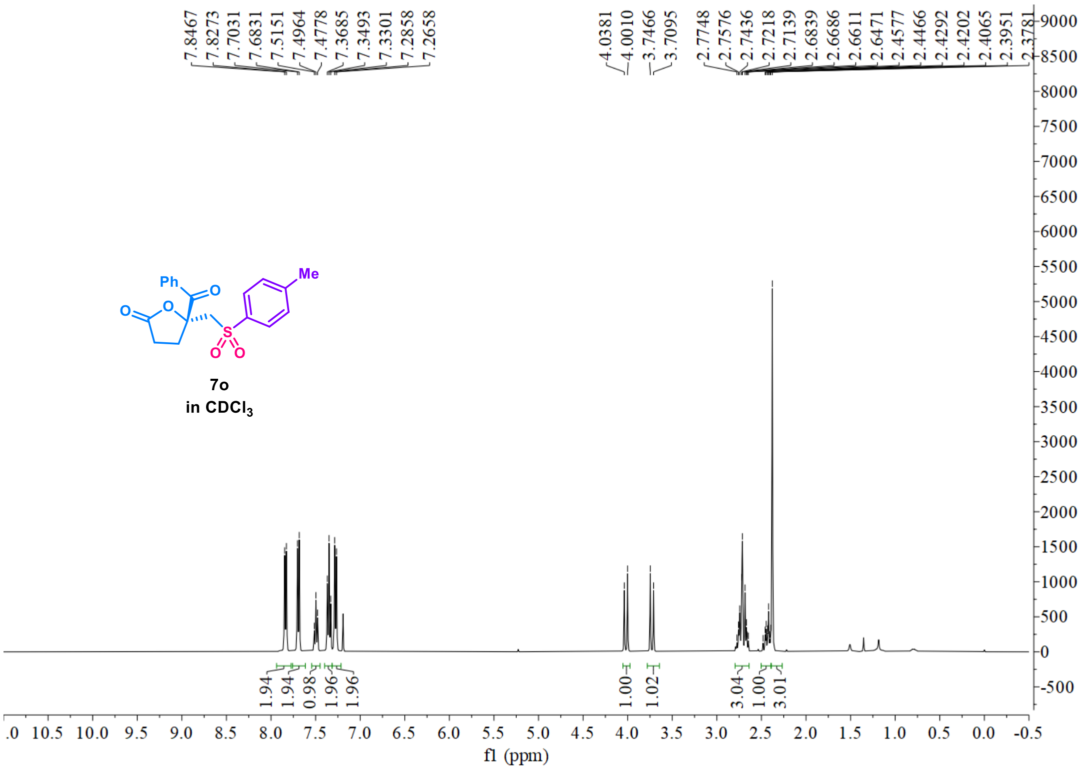
7.8467
7.8273
7.7031
7.6831
7.5151
7.4964
7.4778
7.3685
7.3493
7.3301
7.2858
7.2658
4.0381
4.0010
3.7466
3.7095
2.7748
2.7576
2.7436
2.7218
2.7139
2.6839
2.6686
2.6611
2.6471
2.4577
2.4466
2.4292
2.4202
2.4065
2.3951
2.3781
Ph
O
O
O
S
O
O
Me
7o
in CDCl3
1.94
1.94
0.98
1.96
1.96
1.00
1.02
3.04
1.00
3.01
f1 (ppm)

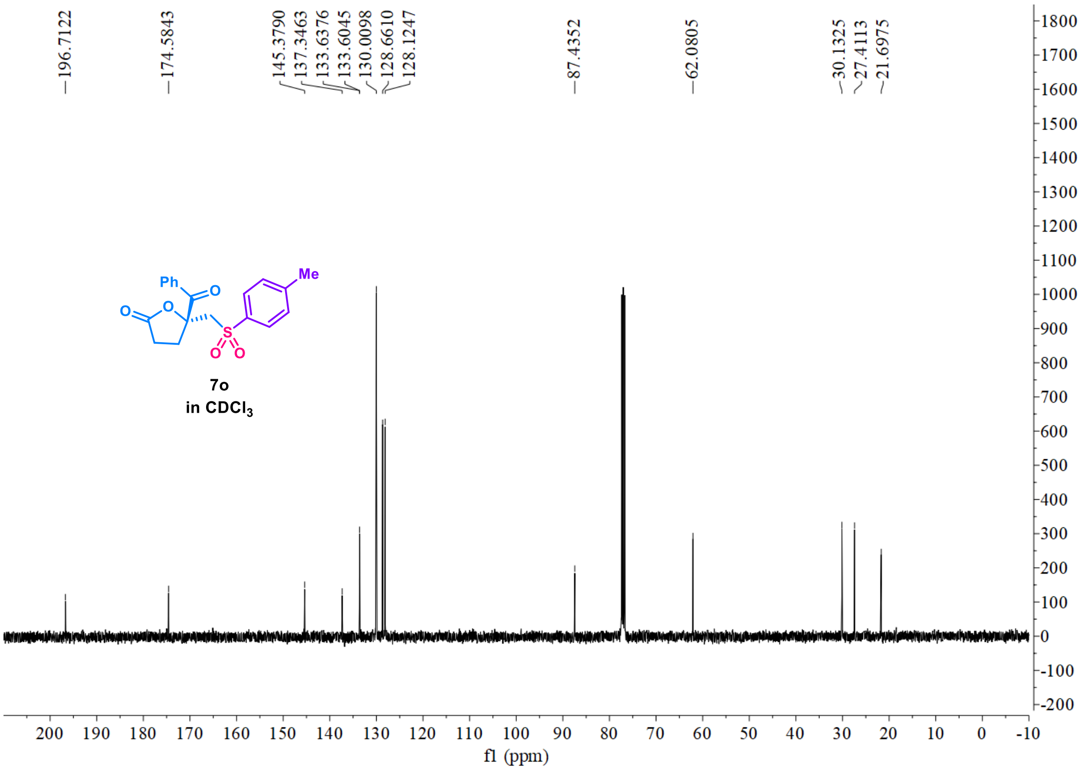
196.7122
174.5843
145.3790
137.3463
133.6376
133.6045
130.0098
128.6610
128.1247
87.4352
62.0805
30.1325
27.4113
21.6975
Ph
O
O
O
S
O
O
Me
7o
in CDCl3
f1 (ppm)

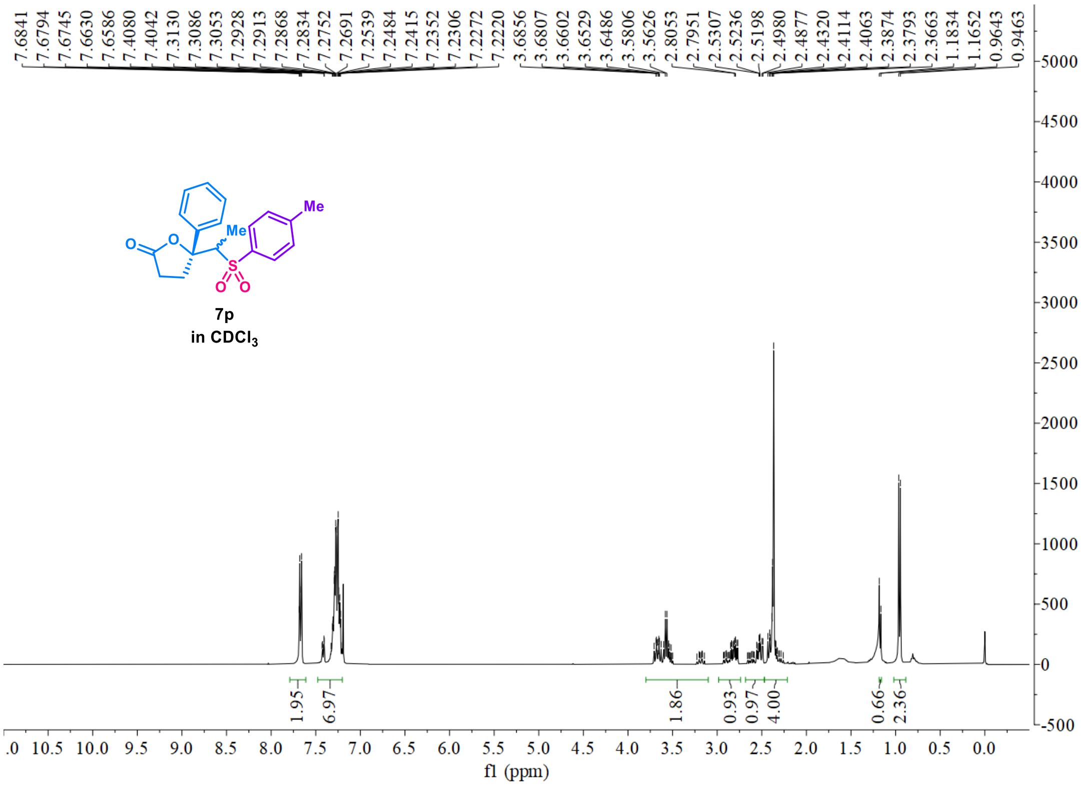
7.6841 7.6794 7.6745 7.6630 7.6586 7.4080 7.4042 7.3130 7.3086 7.3053 7.2928 7.2913 7.2868 7.2834 7.2752 7.2691 7.2539 7.2484 7.2415 7.2352 7.2306 7.2272 7.2220 3.6856 3.6807 3.6602 3.6529 3.6486 3.5806 3.5626 2.8053 2.7951 2.5307 2.5236 2.5198 2.4980 2.4877 2.4320 2.4114 2.4063 2.3874 2.3793 2.3663 1.1834 1.1652 0.9643 0.9463
Me
Me
O
O
S
O
O
7p
in CDCl3
1.95
6.97
1.86
0.93
0.97
4.00
0.66
2.36
f1 (ppm)

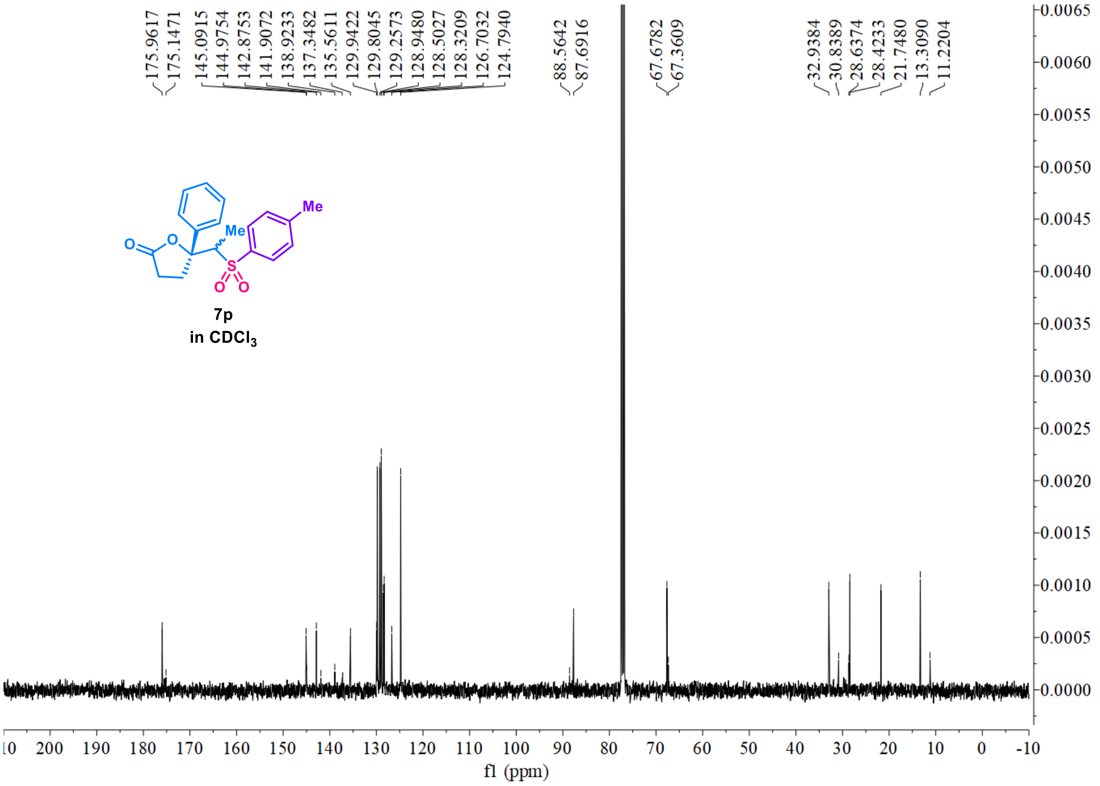
175.9617 175.1471 145.0915 144.9754 142.8753 141.9072 138.9233 137.3482 135.5611 129.9422 129.8045 129.2573 128.9480 128.5027 128.3209 126.7032 124.7940 88.5642 87.6916 67.6782 67.3609 32.9384 30.8389 28.6374 28.4233 21.7480 13.3090 11.2204
Me
Me
O
O
S
O
O
7p
in CDCl3
f1 (ppm)

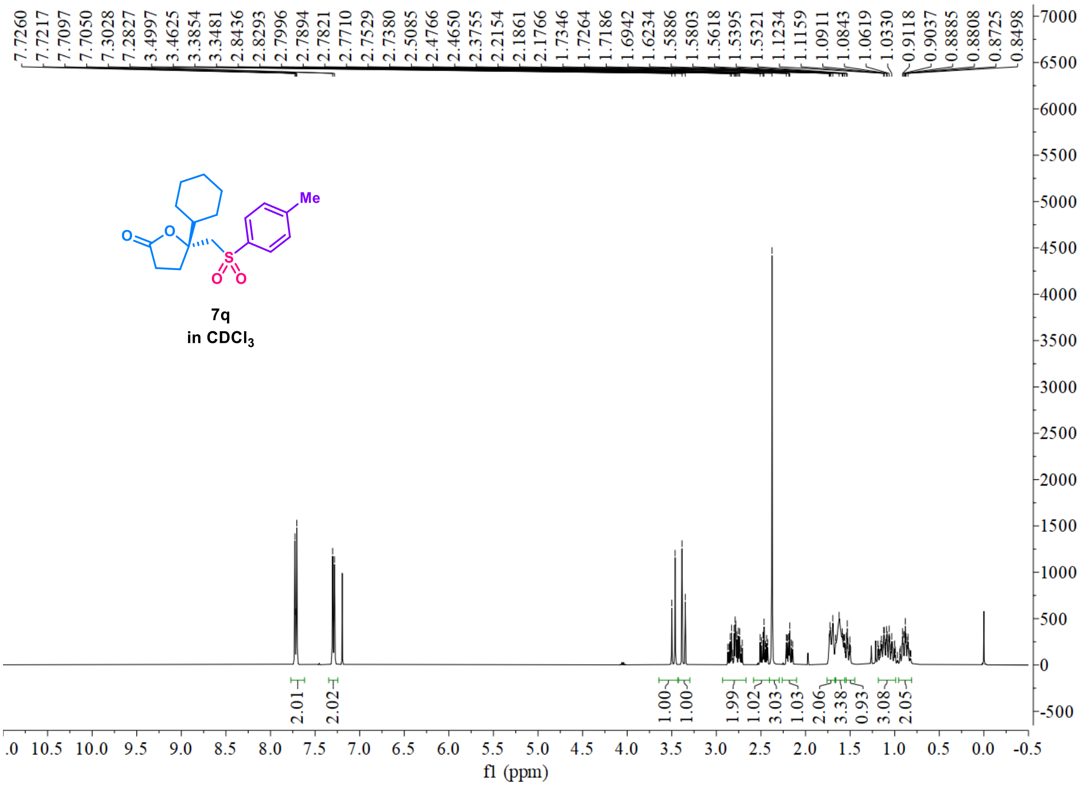
7q
in $CDCl_3$

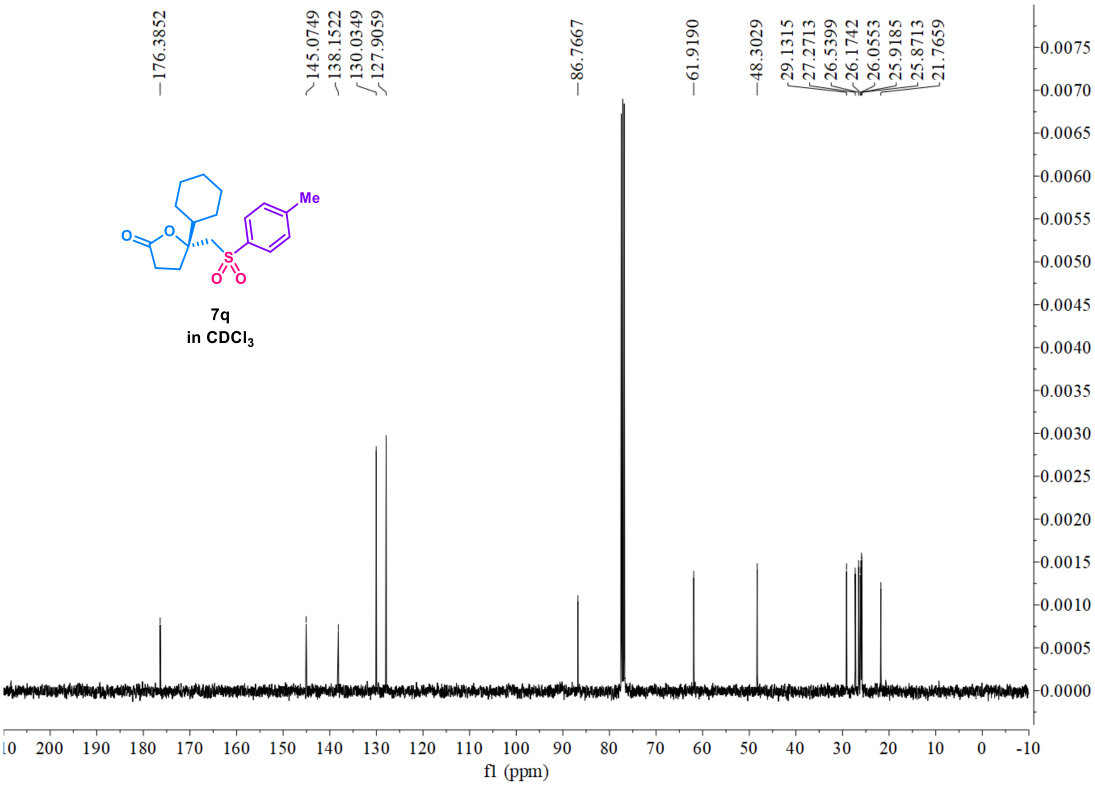
7q
in $CDCl_3$

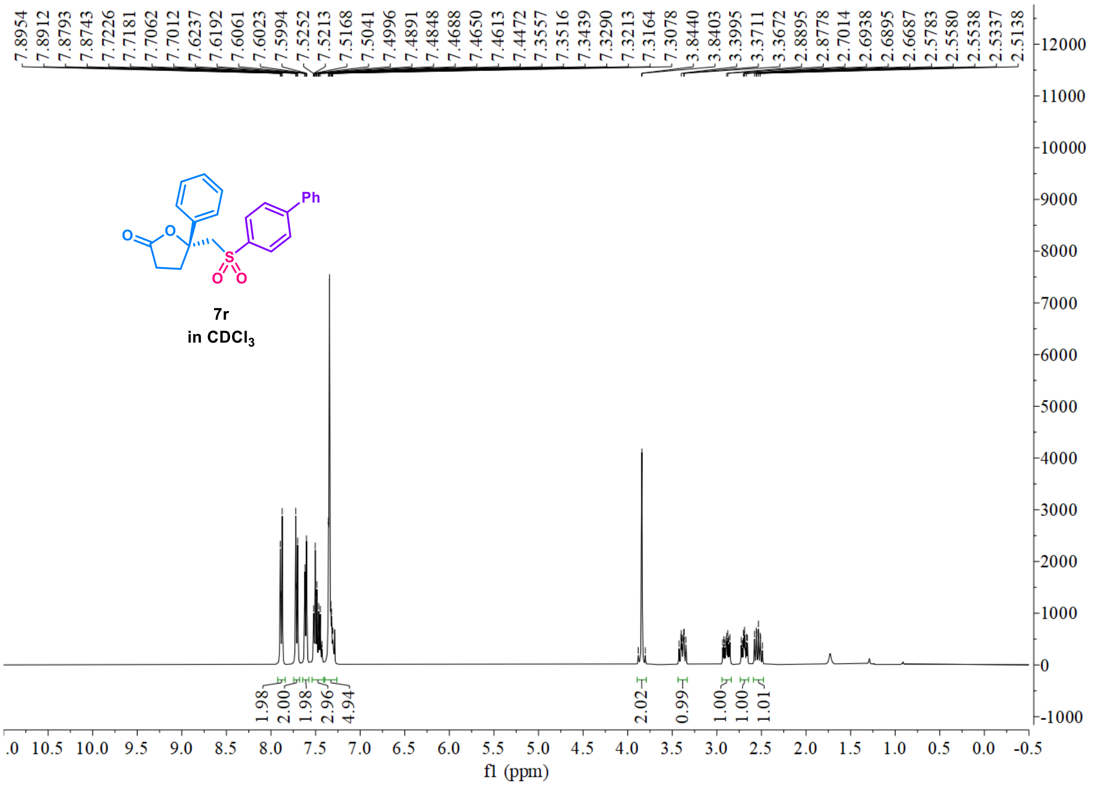
7.8954
7.8912
7.8793
7.8743
7.7226
7.7181
7.7062
7.7012
7.6237
7.6192
7.6061
7.6023
7.5994
7.5252
7.5213
7.5168
7.5041
7.4996
7.4891
7.4848
7.4688
7.4650
7.4613
7.4472
7.3557
7.3516
7.3439
7.3290
7.3213
7.3164
7.3078
3.8440
3.8403
3.3995
3.3711
3.3672
2.8895
2.8778
2.7014
2.6938
2.6895
2.6687
2.5783
2.5580
2.5538
2.5337
2.5138
Ph
O
O
S
O
O
7r
in CDCl3
1.98
2.00
1.98
2.96
4.94
2.02
0.99
1.00
1.00
1.01
f1 (ppm)

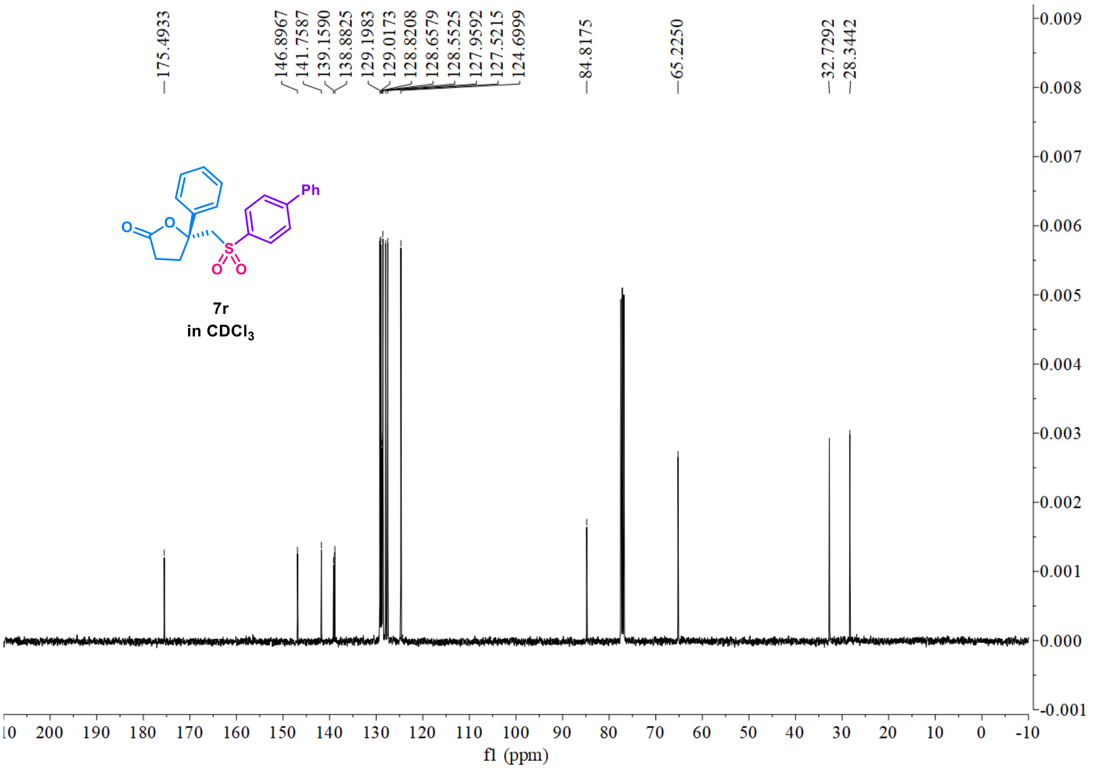
175.4933
146.8967
141.7587
139.1590
138.8825
129.1983
129.0173
128.8208
128.6579
128.5525
127.9592
127.5215
124.6999
84.8175
65.2250
32.7292
28.3442
Ph
O
O
S
O
O
7r
in CDCl3
f1 (ppm)

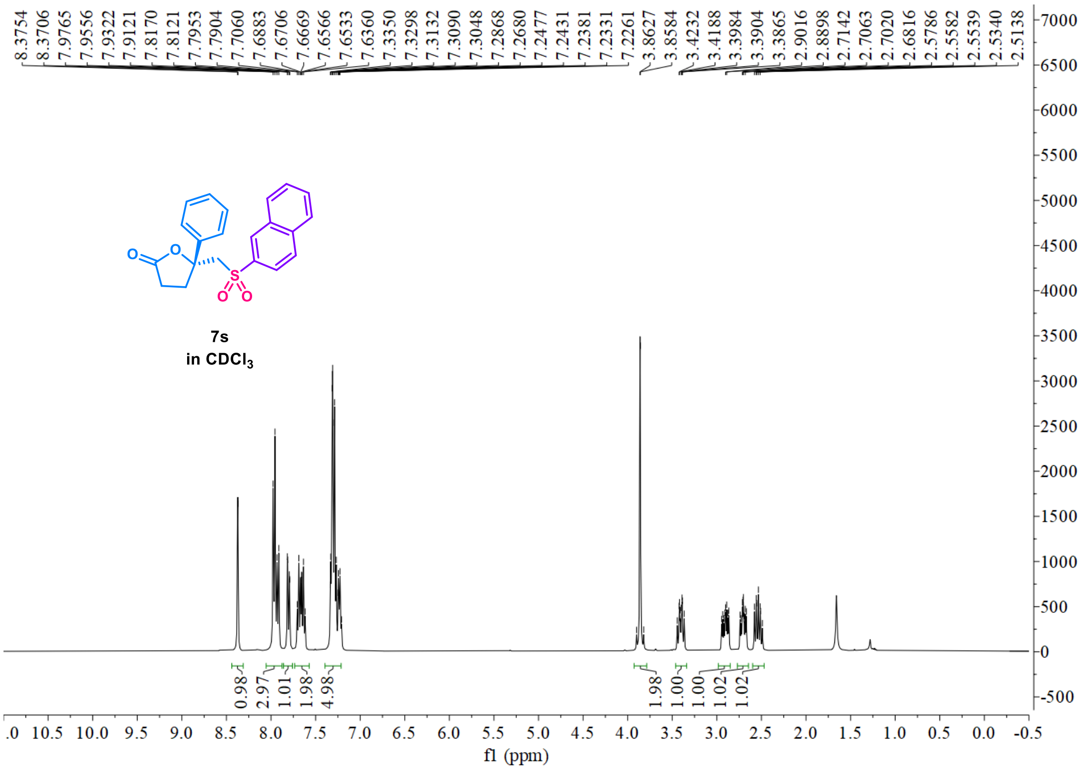
8.3754
8.3706
7.9765
7.9556
7.9322
7.9121
7.8170
7.8121
7.7953
7.7904
7.7060
7.6883
7.6706
7.6669
7.6566
7.6533
7.6360
7.3350
7.3298
7.3132
7.3090
7.3048
7.2868
7.2680
7.2477
7.2431
7.2381
7.2331
7.2261
3.8627
3.8584
3.4232
3.4188
3.3984
3.3904
3.3865
2.9016
2.8898
2.7142
2.7063
2.7020
2.6816
2.5786
2.5582
2.5539
2.5340
2.5138
7s
in CDCl3
0.98
2.97
1.01
1.98
4.98
1.98
1.00
1.00
1.02
1.02
f1 (ppm)

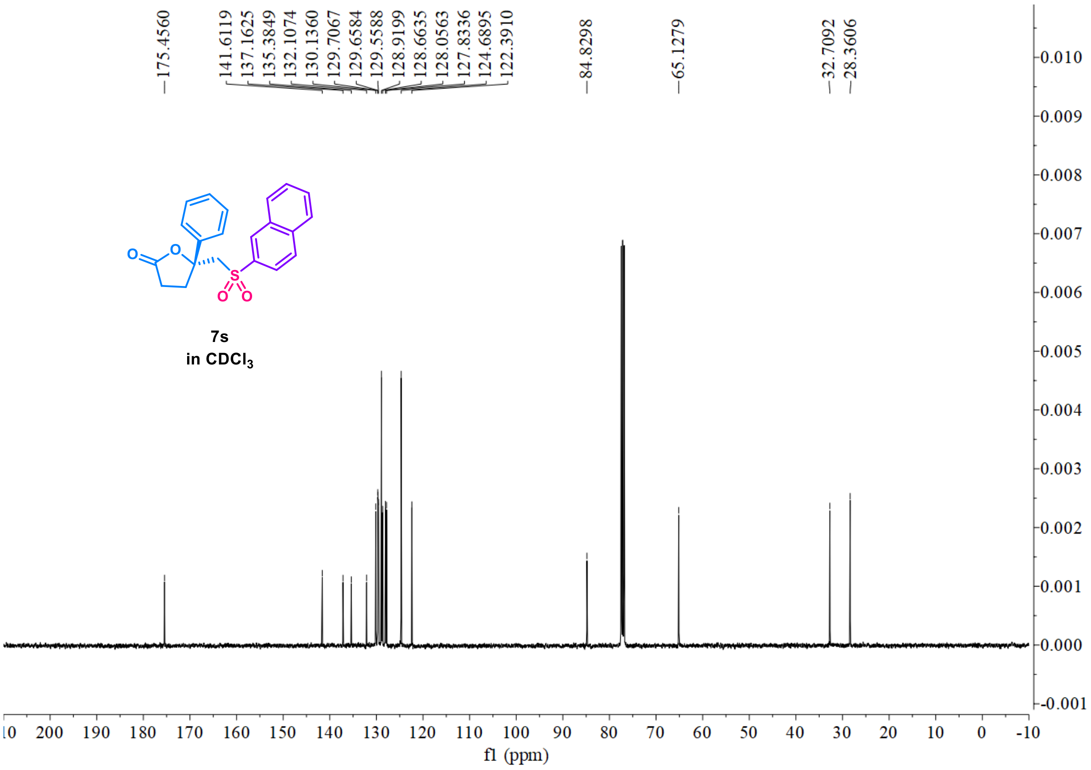
175.4560
141.6119
137.1625
135.3849
132.1074
130.1360
129.7067
129.6584
129.5588
128.9199
128.6635
128.0563
127.8336
124.6895
122.3910
84.8298
65.1279
32.7092
28.3606
7s
in CDCl3
f1 (ppm)

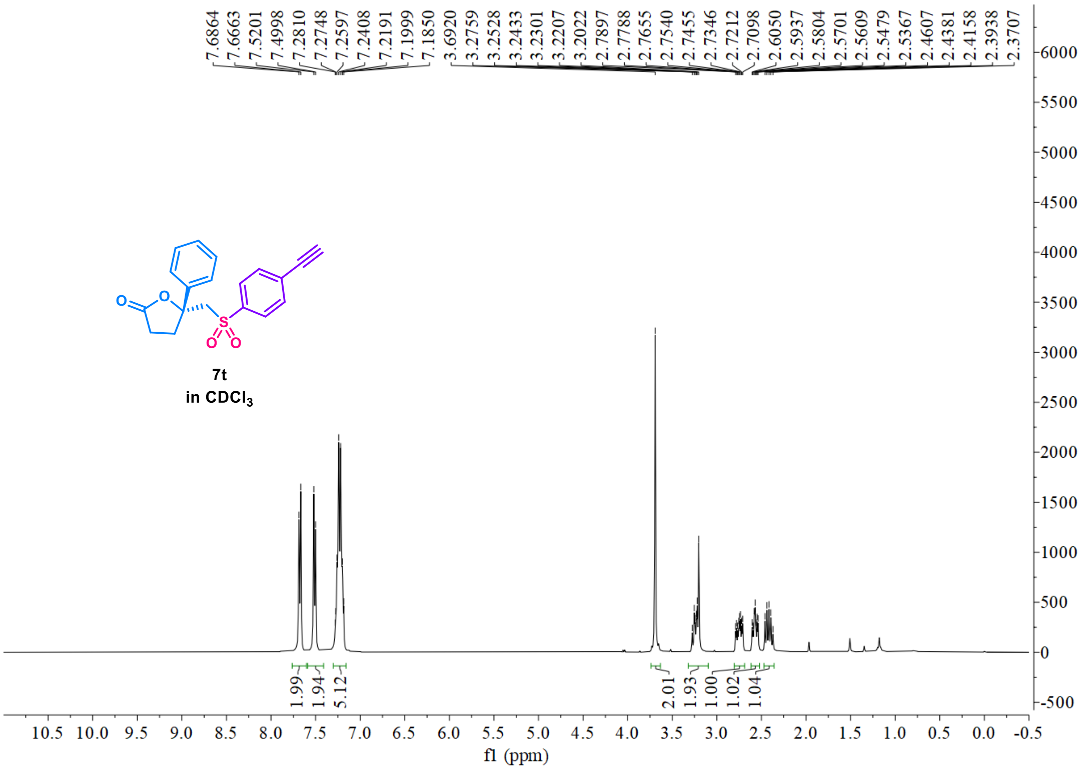
7.6864
7.6663
7.5201
7.4998
7.2810
7.2748
7.2597
7.2408
7.2191
7.1999
7.1850
3.6920
3.2759
3.2528
3.2433
3.2301
3.2207
3.2022
2.7897
2.7788
2.7655
2.7540
2.7455
2.7346
2.7212
2.7098
2.6050
2.5937
2.5804
2.5701
2.5609
2.5479
2.5367
2.4607
2.4381
2.4158
2.3938
2.3707
7t
in CDCl3
1.99
1.94
5.12
2.01
1.93
1.00
1.02
1.04
f1 (ppm)

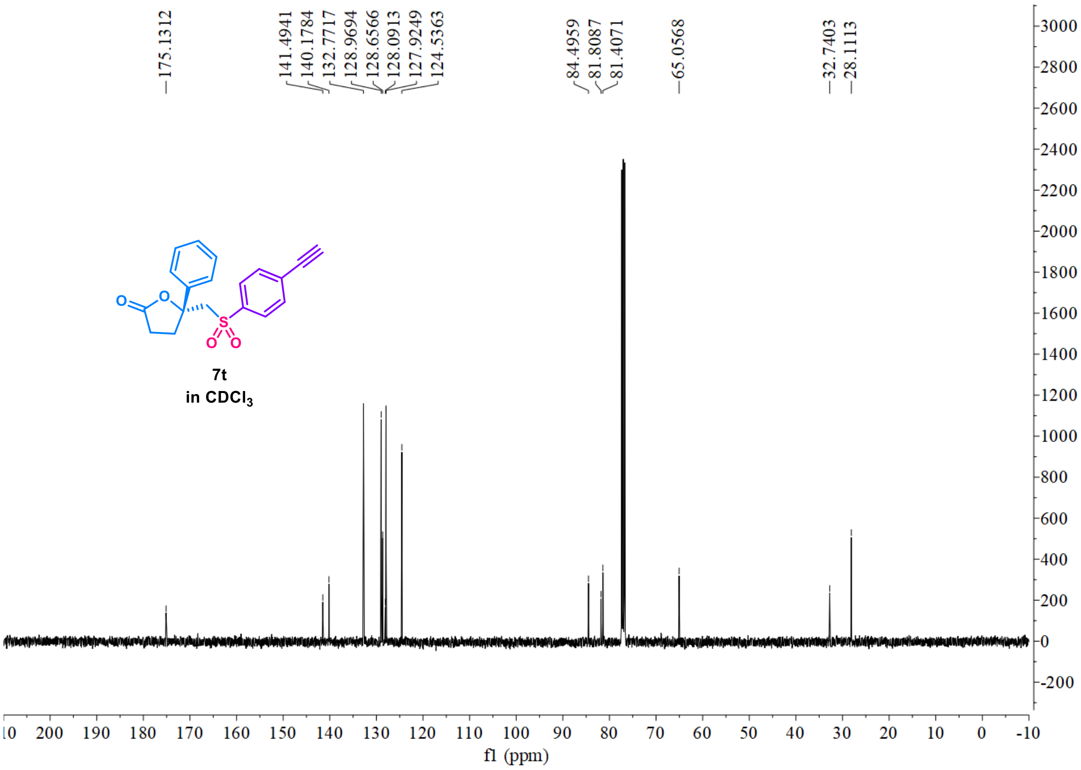
175.1312
141.4941
140.1784
132.7717
128.9694
128.6566
128.0913
127.9249
124.5363
84.4959
81.8087
81.4071
65.0568
32.7403
28.1113
7t
in CDCl3
f1 (ppm)

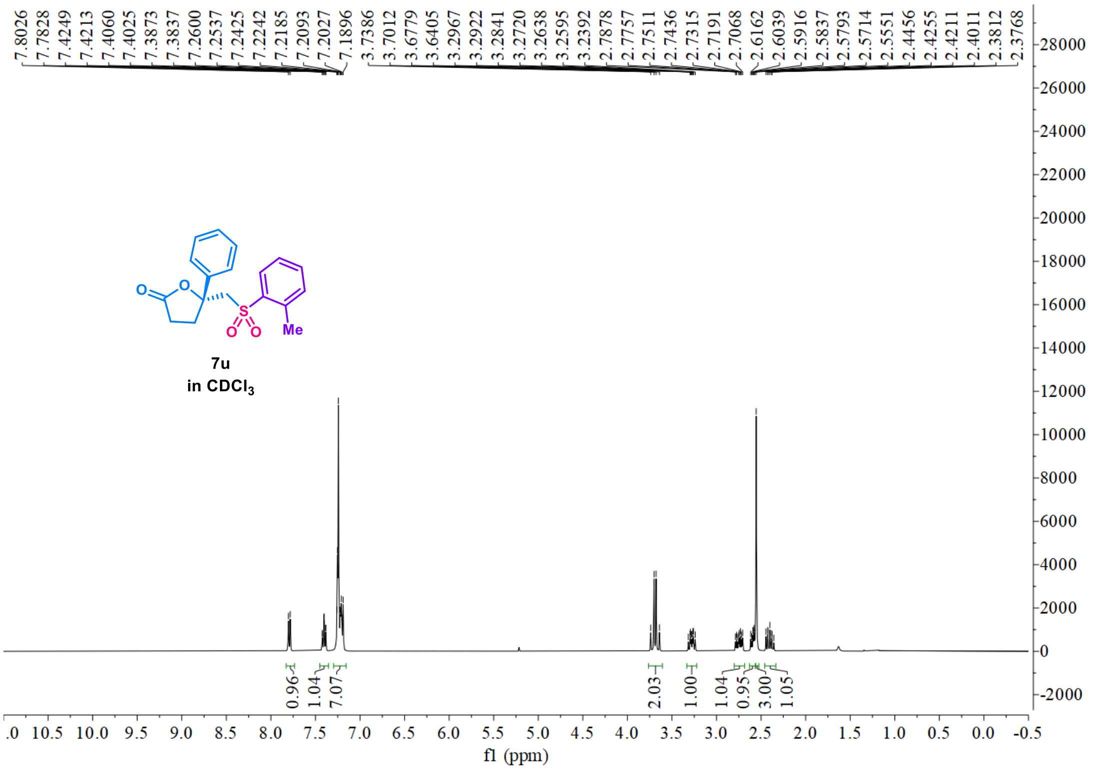

7.8026
7.7828
7.4249
7.4213
7.4060
7.4025
7.3873
7.3837
7.2600
7.2537
7.2425
7.2242
7.2185
7.2093
7.2027
7.1896
3.7386
3.7012
3.6779
3.6405
3.2967
3.2922
3.2841
3.2720
3.2638
3.2595
3.2392
2.7878
2.7757
2.7511
2.7436
2.7315
2.7191
2.7068
2.6162
2.6039
2.5916
2.5837
2.5793
2.5714
2.5551
2.4456
2.4255
2.4211
2.4011
2.3812
2.3768
Me
7u
in $CDCl_3$
0.96
1.04
7.07
2.03
1.00
1.04
0.95
3.00
1.05
f1 (ppm)

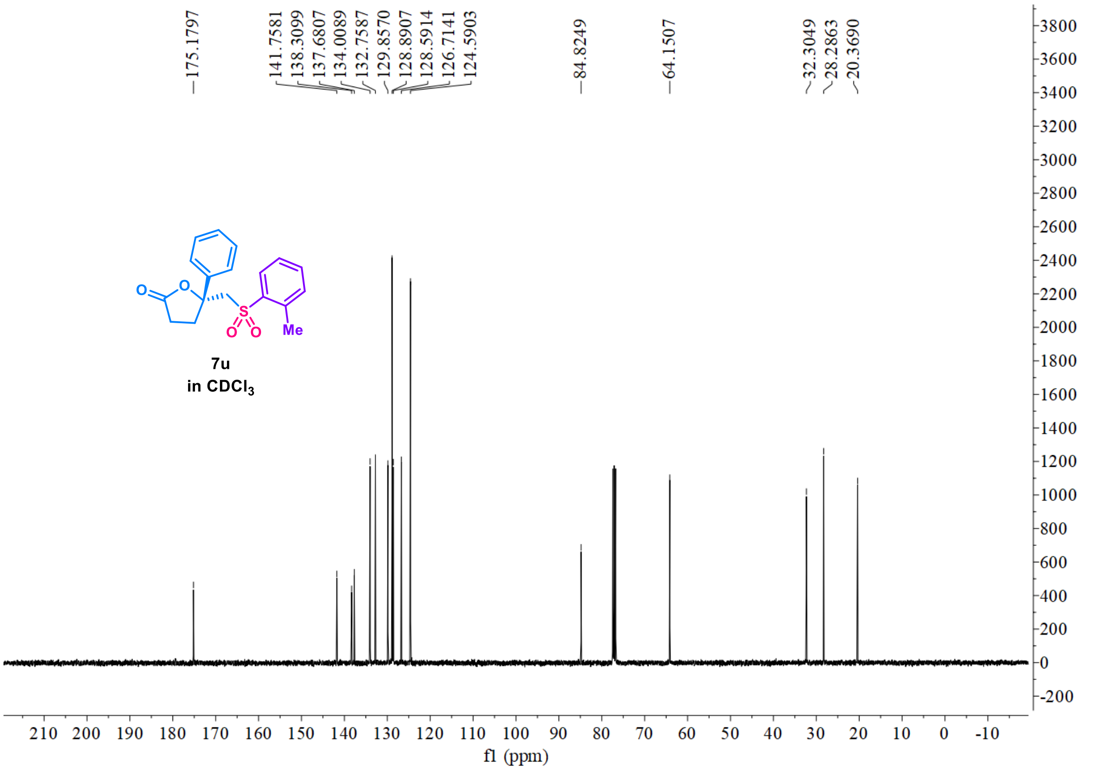

175.1797
141.7581
138.3099
137.6807
134.0089
132.7587
129.8570
128.8907
128.5914
126.7141
124.5903
84.8249
64.1507
32.3049
28.2863
20.3690
Me
7u
in $CDCl_3$
f1 (ppm)

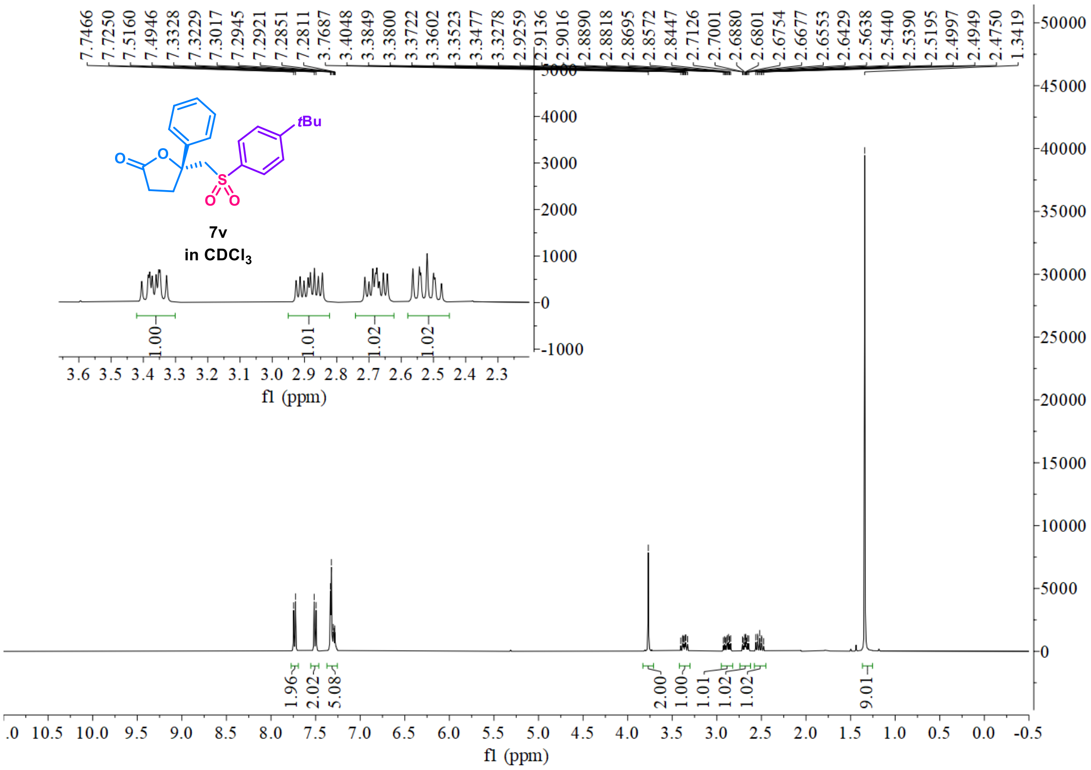
7v
in CDCl3
f1 (ppm)

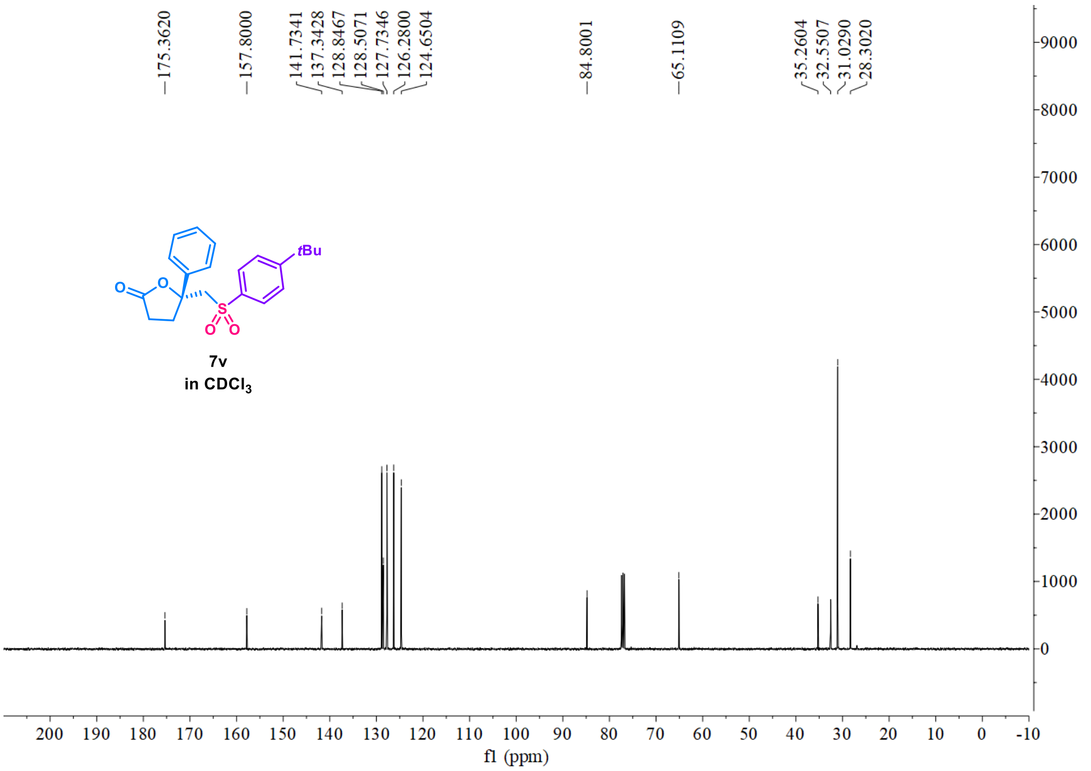
7v
in CDCl3
f1 (ppm)

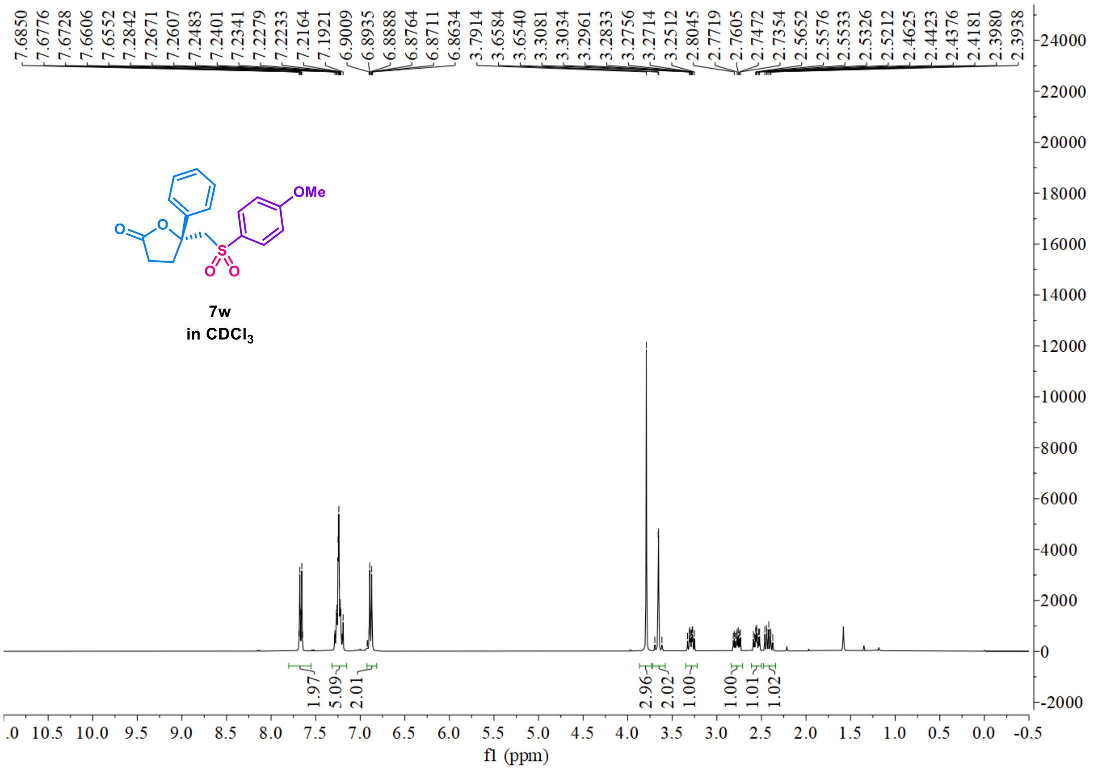
7.6850
7.6776
7.6728
7.6606
7.6552
7.2842
7.2671
7.2607
7.2483
7.2401
7.2341
7.2279
7.2233
7.2164
7.1921
6.9009
6.8935
6.8888
6.8764
6.8711
6.8634
3.7914
3.6584
3.6540
3.3081
3.3034
3.2961
3.2833
3.2756
3.2714
3.2512
2.8045
2.7719
2.7605
2.7472
2.7354
2.5652
2.5576
2.5533
2.5326
2.5212
2.4625
2.4423
2.4376
2.4181
2.3980
2.3938
OMe
O
O
S
O
O
7w
in CDCl3
1.97
5.09
2.01
2.96
2.02
1.00
1.00
1.01
1.02
f1 (ppm)

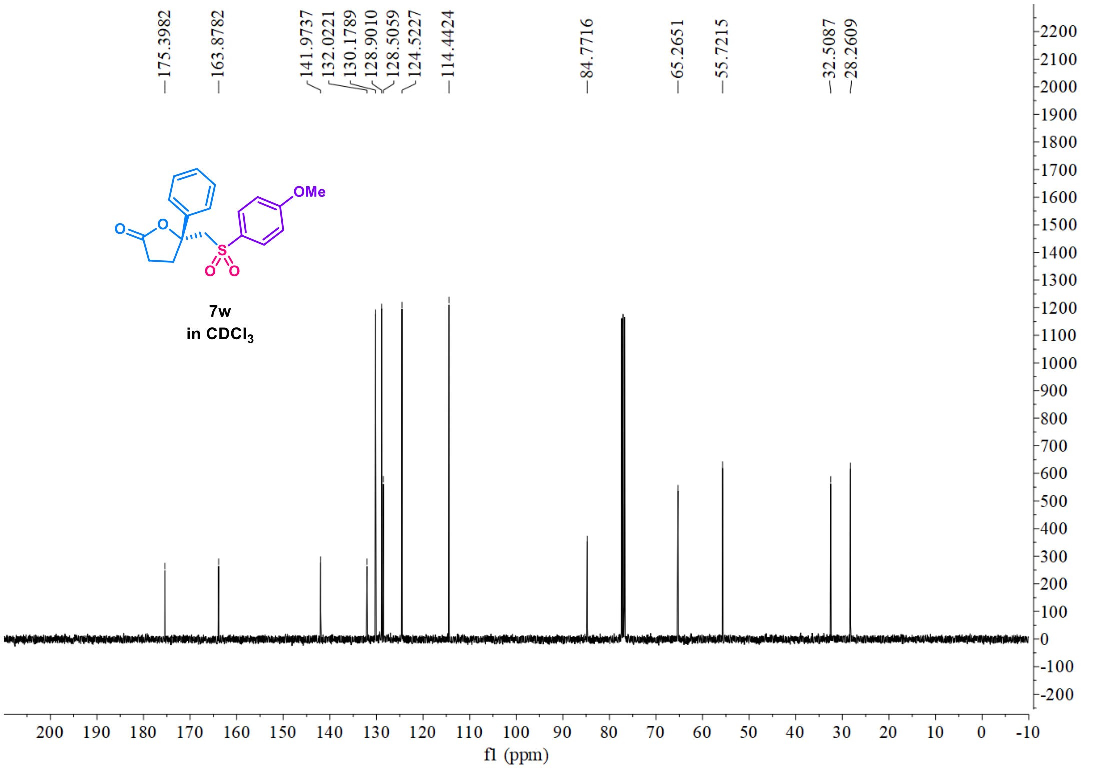
175.3982
163.8782
141.9737
132.0221
130.1789
128.9010
128.5059
124.5227
114.4424
84.7716
65.2651
55.7215
32.5087
28.2609
OMe
O
O
S
O
O
7w
in CDCl3
f1 (ppm)

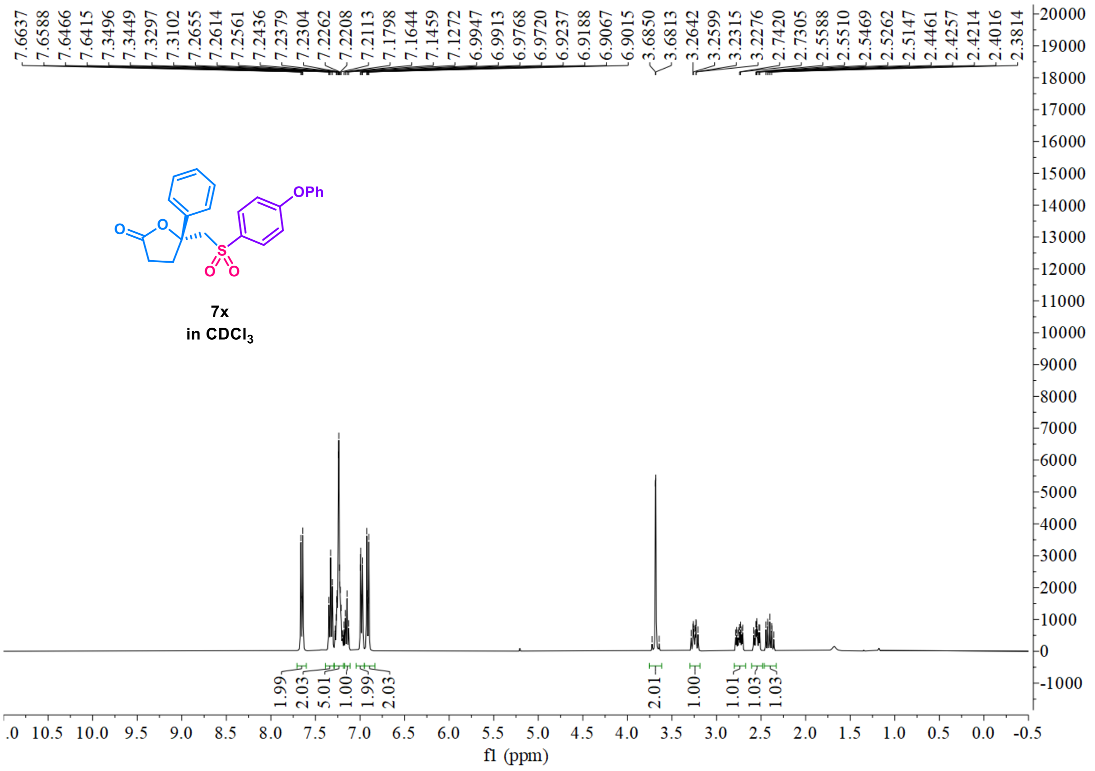
7.6637 7.6588 7.6466 7.6415 7.3496 7.3449 7.3297 7.3102 7.2655 7.2614 7.2561 7.2436 7.2379 7.2304 7.2262 7.2208 7.2113 7.1798 7.1644 7.1459 7.1272 6.9947 6.9913 6.9768 6.9720 6.9237 6.9188 6.9067 6.9015 3.6850 3.6813 3.2642 3.2599 3.2315 3.2276 2.7420 2.7305 2.5588 2.5510 2.5469 2.5262 2.5147 2.4461 2.4257 2.4214 2.4016 2.3814
OPh
O
O
S
O
O
7x
in CDCl3
1.99 2.03 5.01 1.00 1.99 2.03 2.01 1.00 1.01 1.03 1.03
f1 (ppm)

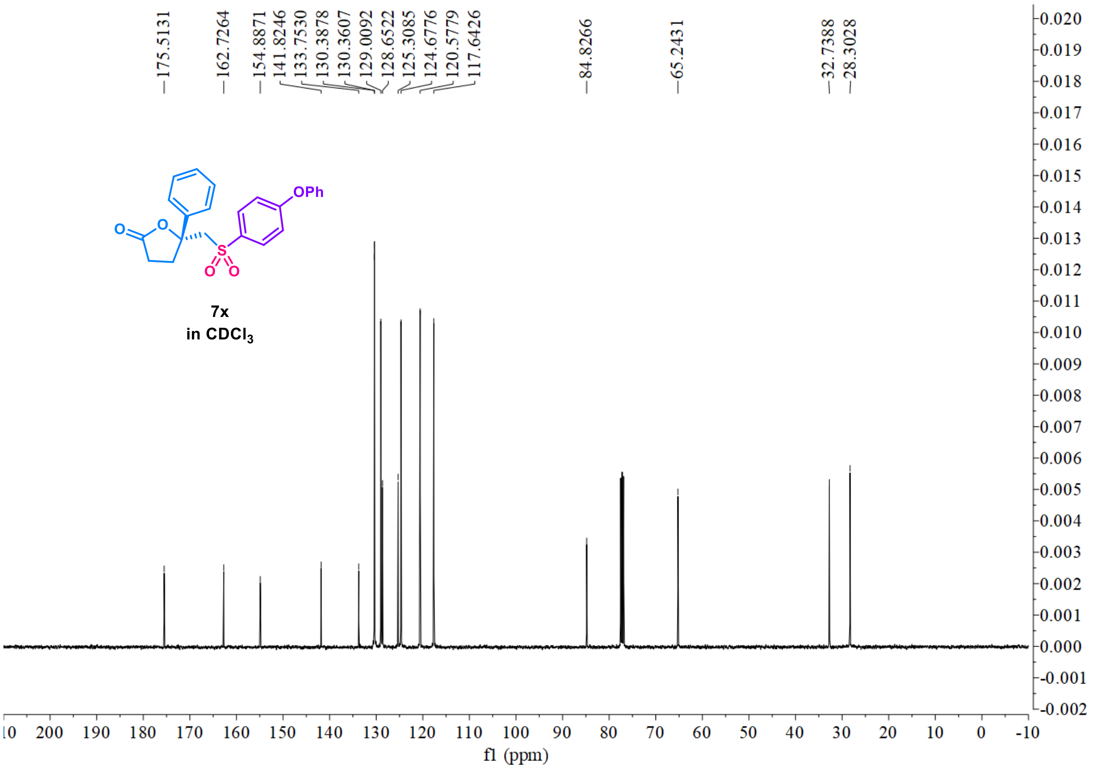
175.5131 162.7264 154.8871 141.8246 133.7530 130.3878 130.3607 129.0092 128.6522 125.3085 124.6776 120.5779 117.6426 84.8266 65.2431 32.7388 28.3028
OPh
O
O
S
O
O
7x
in CDCl3
f1 (ppm)

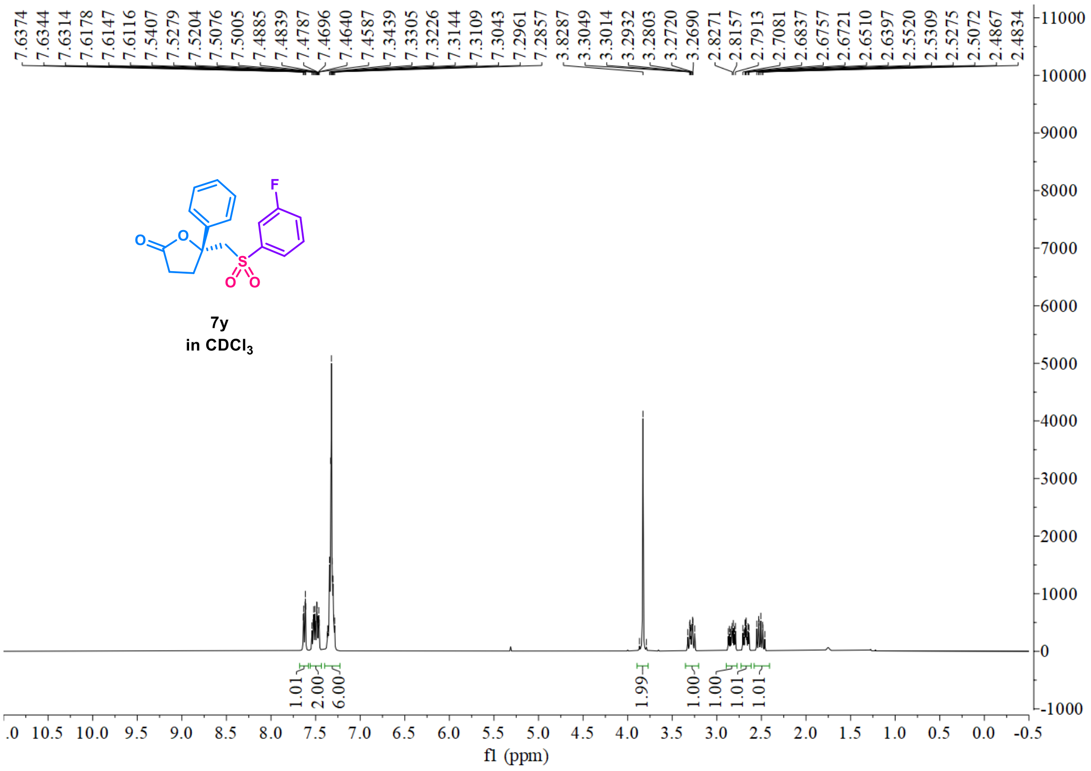
7.6374
7.6344
7.6314
7.6178
7.6147
7.6116
7.5407
7.5279
7.5204
7.5076
7.5005
7.4885
7.4839
7.4787
7.4696
7.4640
7.4587
7.3439
7.3305
7.3226
7.3144
7.3109
7.3043
7.2961
7.2857
3.8287
3.3049
3.3014
3.2932
3.2803
3.2720
3.2690
2.8271
2.8157
2.7913
2.7081
2.6837
2.6757
2.6721
2.6510
2.6397
2.5520
2.5309
2.5275
2.5072
2.4867
2.4834
7y
in CDCl3
1.01
2.00
6.00
1.99
1.00
1.00
1.01
1.01
f1 (ppm)

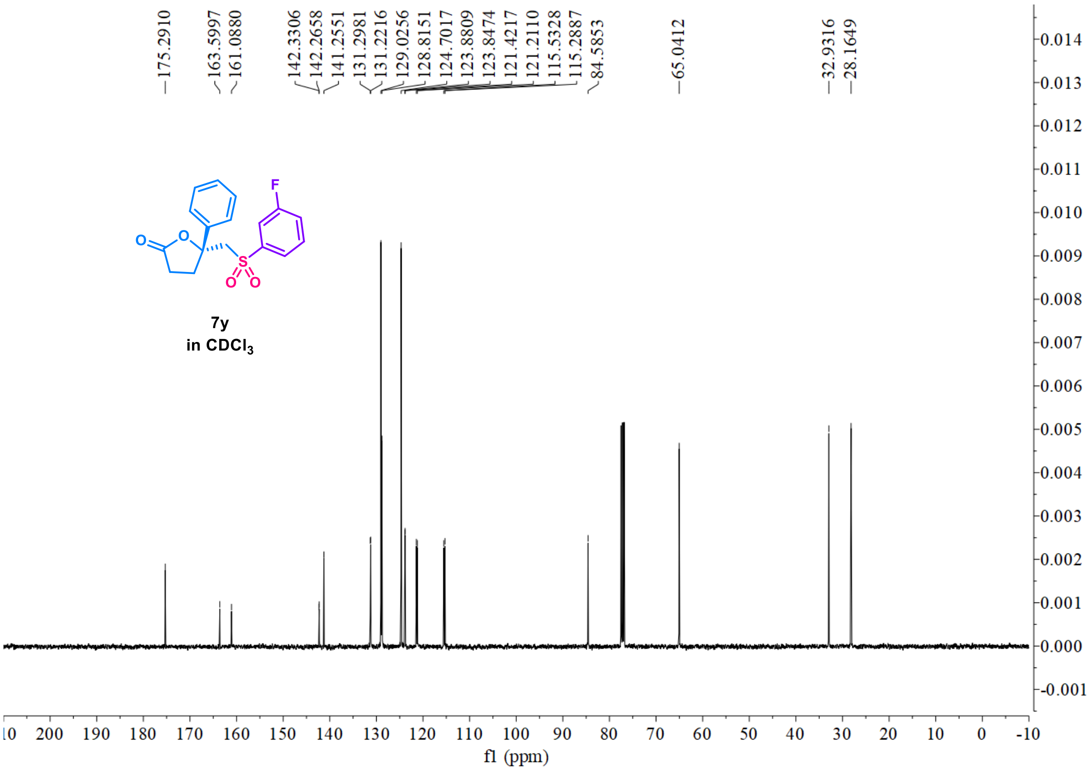
175.2910
163.5997
161.0880
142.3306
142.2658
141.2551
131.2981
131.2216
129.0256
128.8151
124.7017
123.8809
123.8474
121.4217
121.2110
115.5328
115.2887
84.5853
65.0412
32.9316
28.1649
7y
in CDCl3
f1 (ppm)

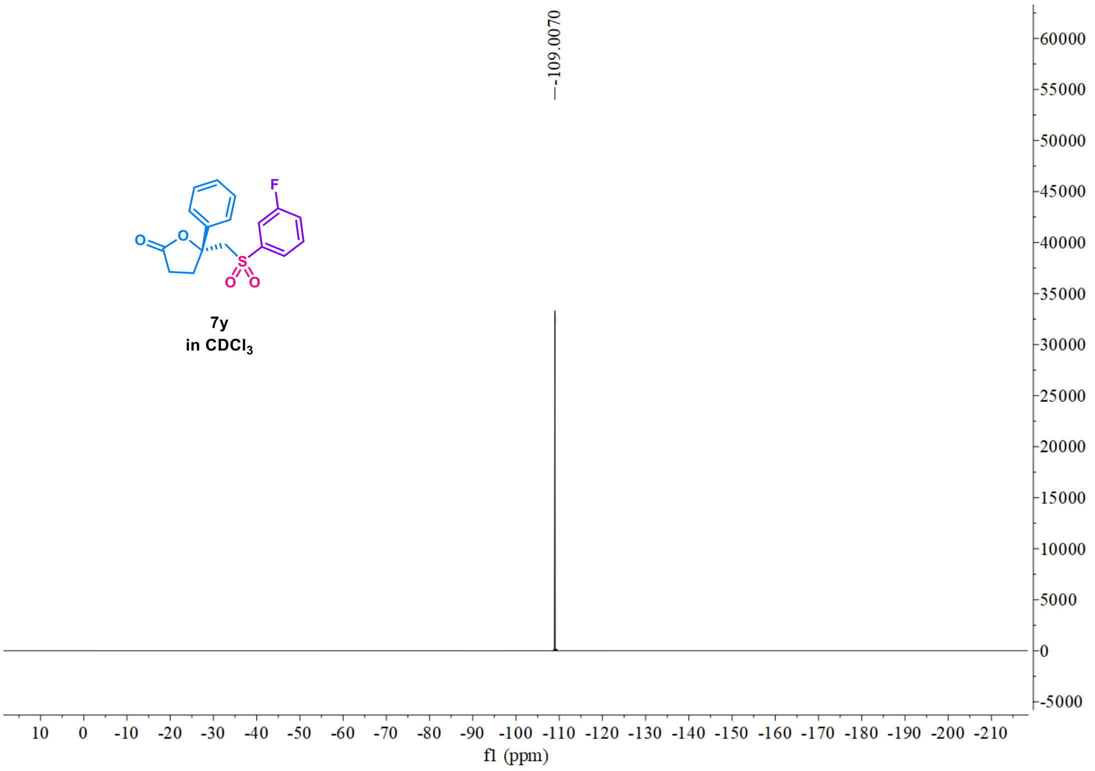

-109.0070

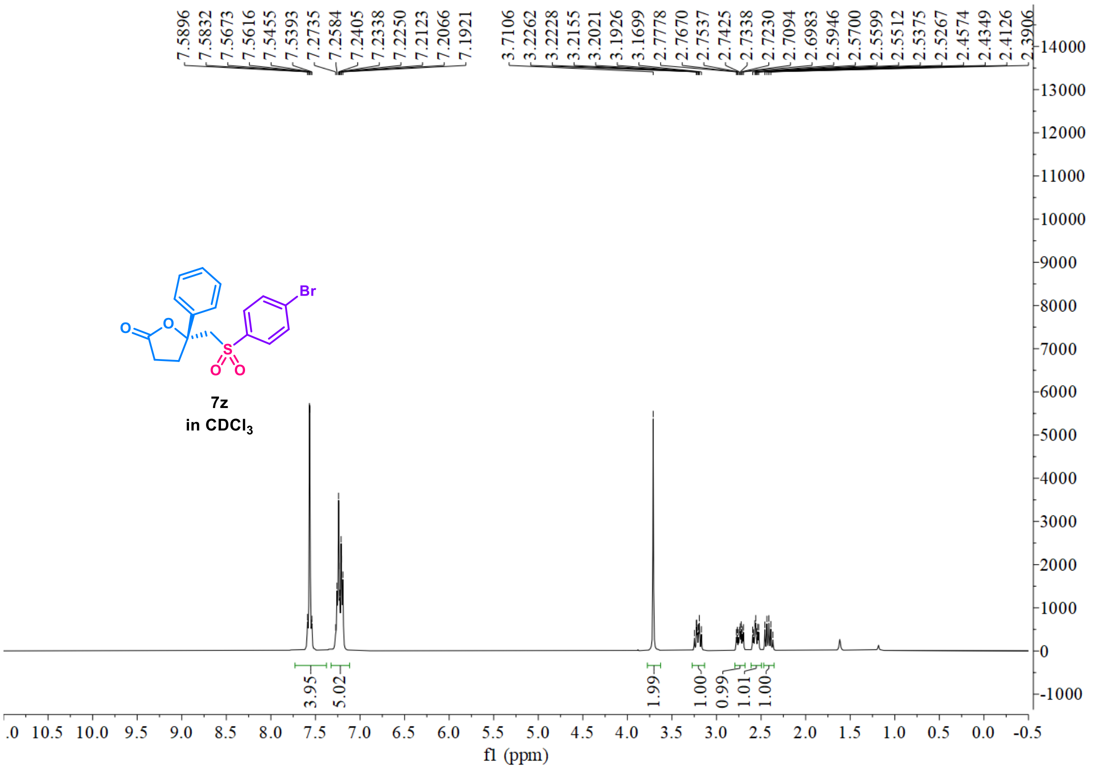

7.5896
7.5832
7.5673
7.5616
7.5455
7.5393
7.2735
7.2584
7.2405
7.2338
7.2250
7.2123
7.2066
7.1921
3.7106
3.2262
3.2228
3.2155
3.2021
3.1926
3.1699
2.7778
2.7670
2.7537
2.7425
2.7338
2.7230
2.7094
2.6983
2.5946
2.5700
2.5599
2.5512
2.5375
2.5267
2.4574
2.4349
2.4126
2.3906
3.95
5.02
1.99
1.00
0.99
1.01
1.00
f1 (ppm)

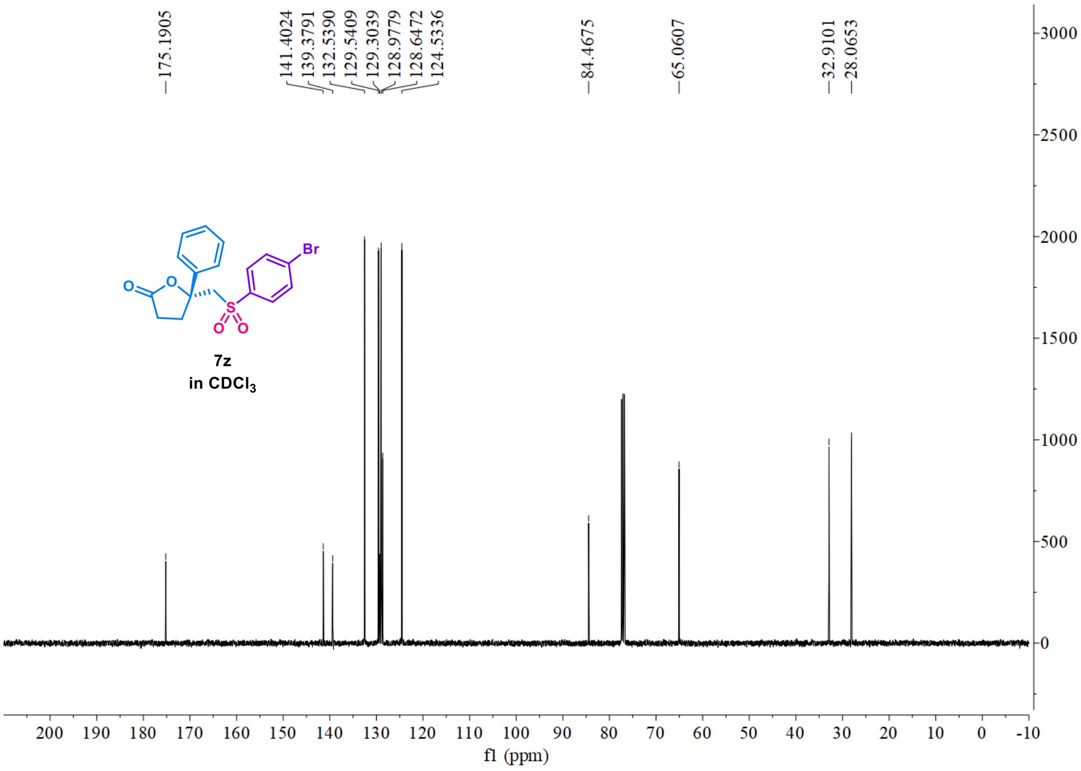
175.1905
141.4024
139.3791
132.5390
129.5409
129.3039
128.9779
128.6472
124.5336
84.4675
65.0607
32.9101
28.0653
Br
O
S
7z
in $CDCl_3$
f1 (ppm)

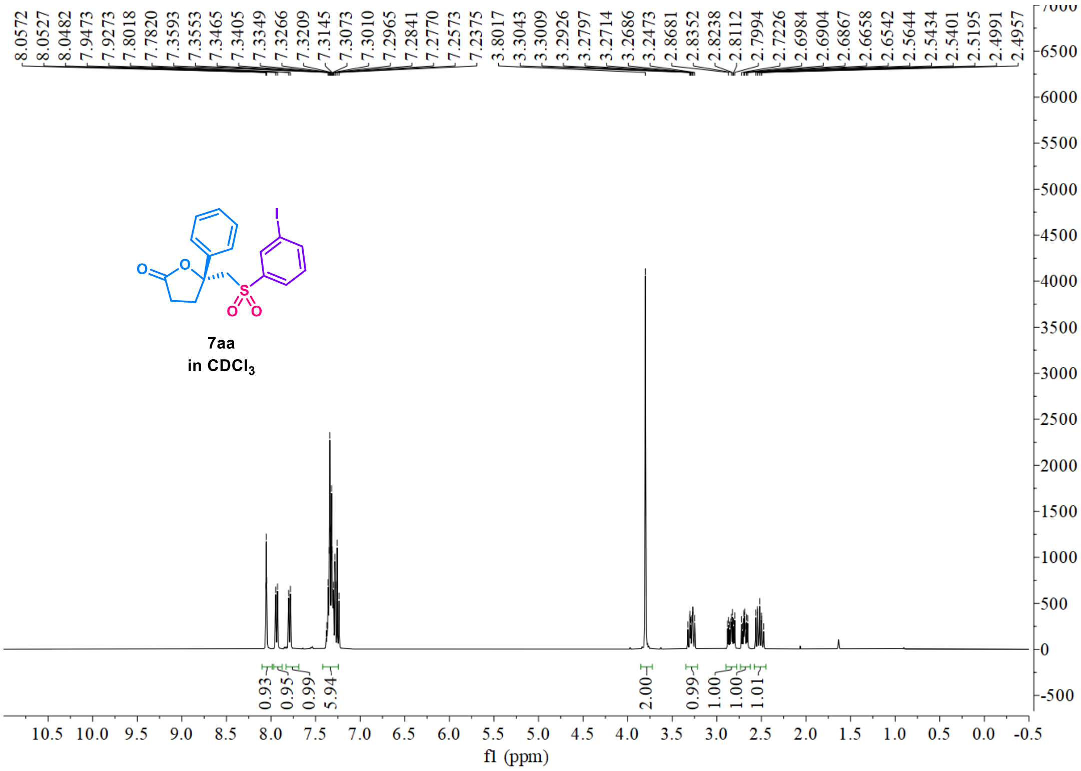
8.0572
8.0527
8.0482
7.9473
7.9273
7.8018
7.7820
7.3593
7.3553
7.3465
7.3405
7.3349
7.3266
7.3209
7.3145
7.3073
7.3010
7.2965
7.2841
7.2770
7.2573
7.2375
3.8017
3.3043
3.3009
3.2926
3.2797
3.2714
3.2686
3.2473
2.8681
2.8352
2.8238
2.8112
2.7994
2.7226
2.6984
2.6904
2.6867
2.6658
2.6542
2.5644
2.5434
2.5401
2.5195
2.4991
2.4957
I
O
S
7aa
in $CDCl_3$
0.93
0.95
0.99
5.94
2.00
0.99
1.00
1.00
1.01
f1 (ppm)

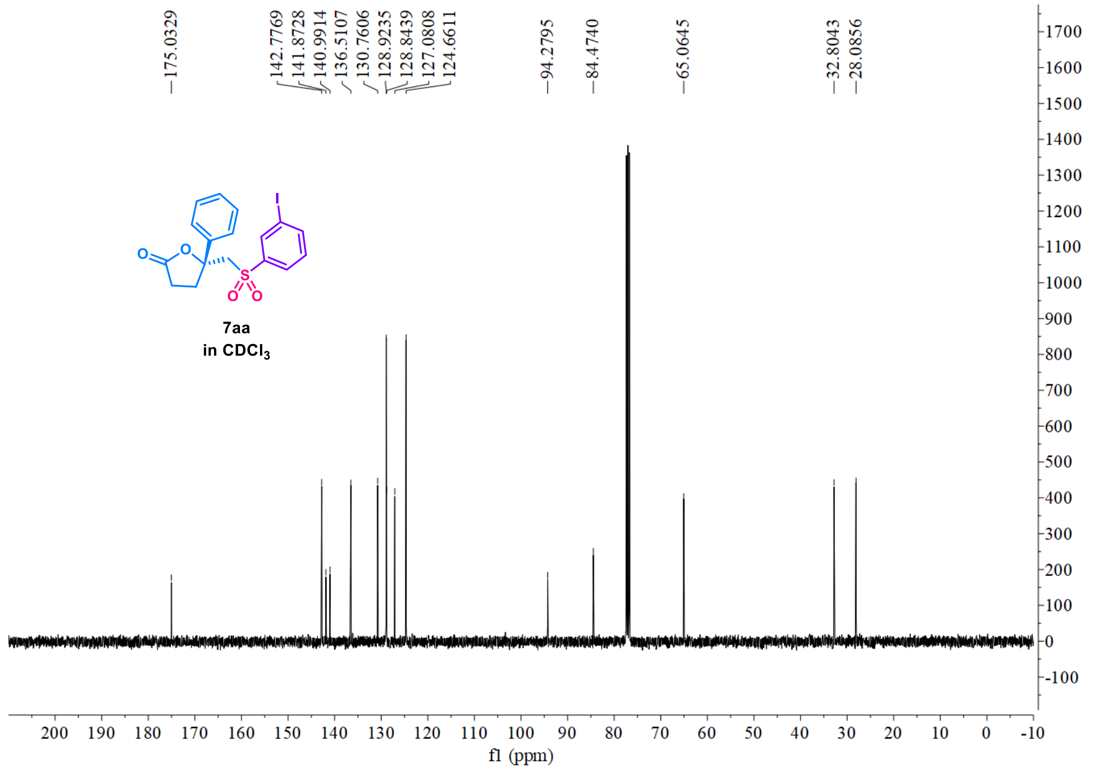
175.0329
142.7769
141.8728
140.9914
136.5107
130.7606
128.9235
128.8439
127.0808
124.6611
94.2795
84.4740
65.0645
32.8043
28.0856
7aa
in $CDCl_3$
f1 (ppm)

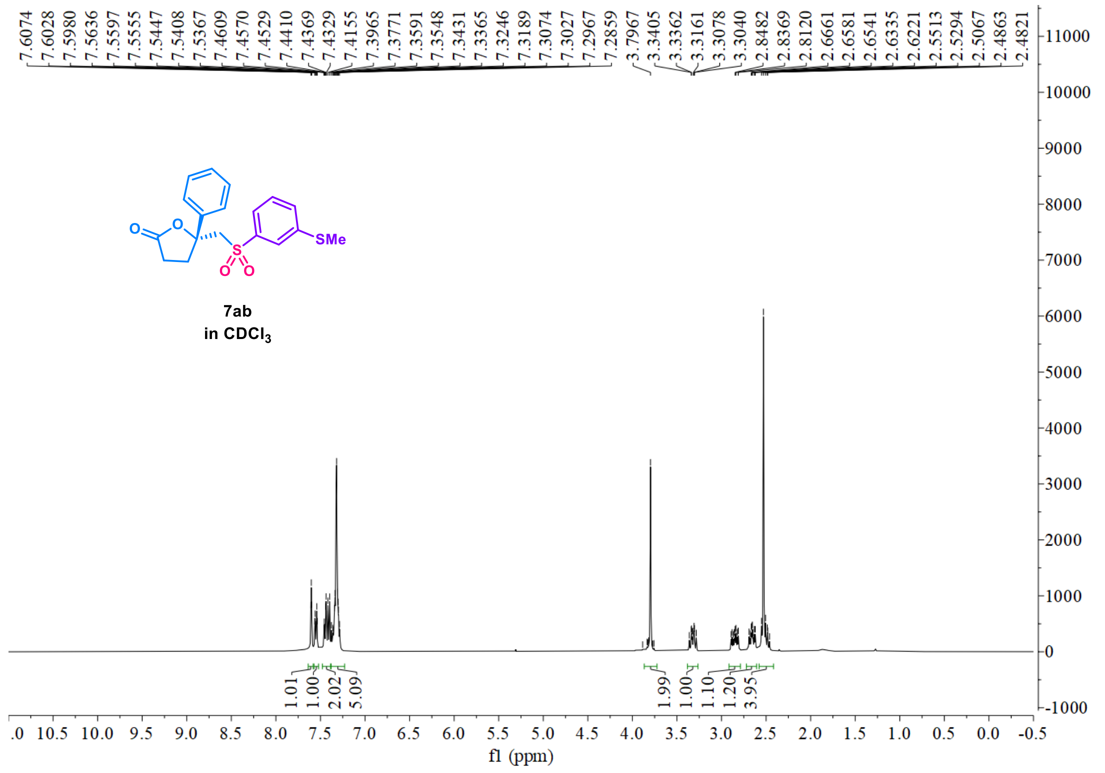
7.6074
7.6028
7.5980
7.5636
7.5597
7.5555
7.5447
7.5408
7.5367
7.4609
7.4570
7.4529
7.4410
7.4369
7.4329
7.4155
7.3965
7.3771
7.3591
7.3548
7.3431
7.3365
7.3246
7.3189
7.3074
7.3027
7.2967
7.2859
3.7967
3.3405
3.3362
3.3161
3.3078
3.3040
2.8482
2.8369
2.8120
2.6661
2.6581
2.6541
2.6335
2.6221
2.5513
2.5294
2.5067
2.4863
2.4821
SMe
7ab
in $CDCl_3$
1.01
1.00
2.02
5.09
1.99
1.00
1.10
1.20
3.95
f1 (ppm)

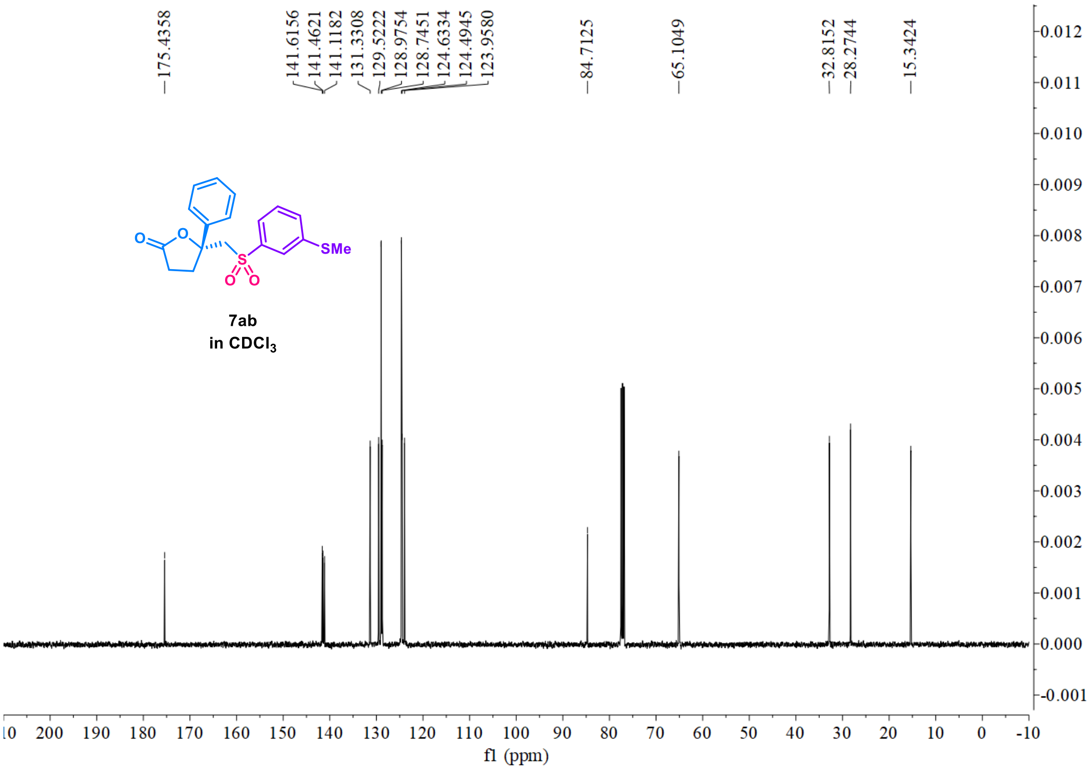

175.4358
141.6156
141.4621
141.1182
131.3308
129.5222
128.9754
128.7451
124.6334
124.4945
123.9580
84.7125
65.1049
32.8152
28.2744
15.3424
SMe
7ab
in CDCl3
f1 (ppm)

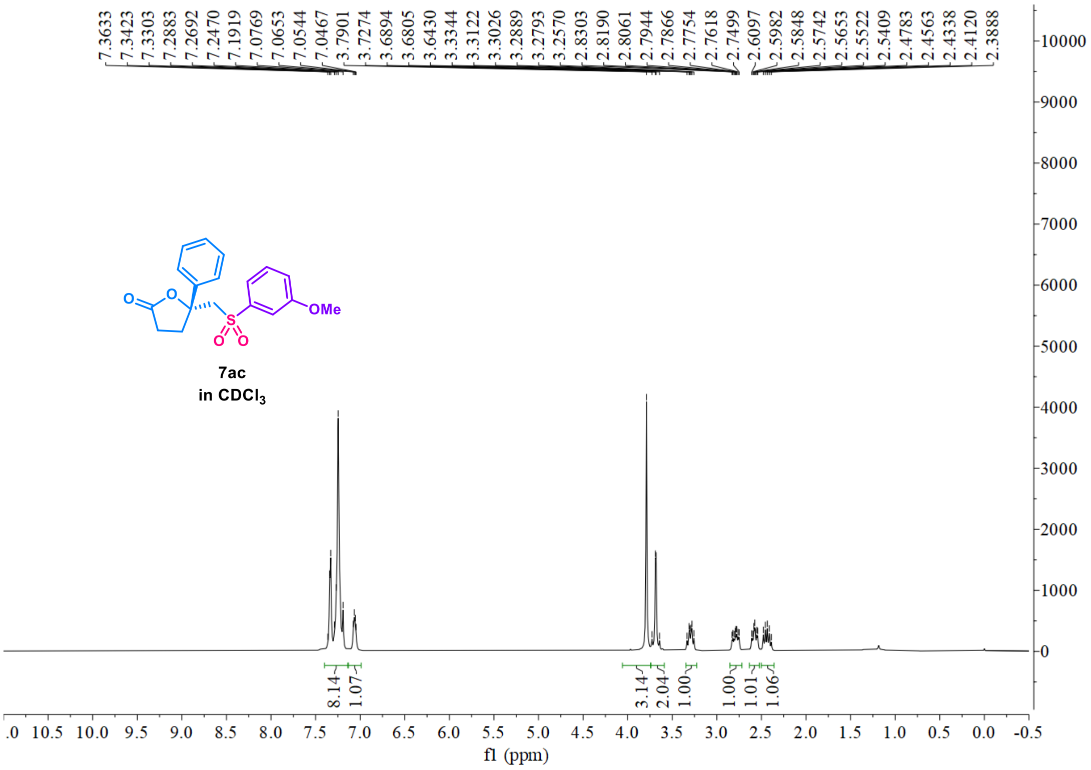

7.3633
7.3423
7.3303
7.2883
7.2692
7.2470
7.1919
7.0769
7.0653
7.0544
7.0467
3.7901
3.7274
3.6894
3.6805
3.6430
3.3344
3.3122
3.3026
3.2889
3.2793
3.2570
2.8303
2.8190
2.8061
2.7944
2.7866
2.7754
2.7618
2.7499
2.6097
2.5982
2.5848
2.5742
2.5653
2.5522
2.5409
2.4783
2.4563
2.4338
2.4120
2.3888
OMe
7ac
in CDCl3
8.14
1.07
3.14
2.04
1.00
1.00
1.01
1.06
f1 (ppm)

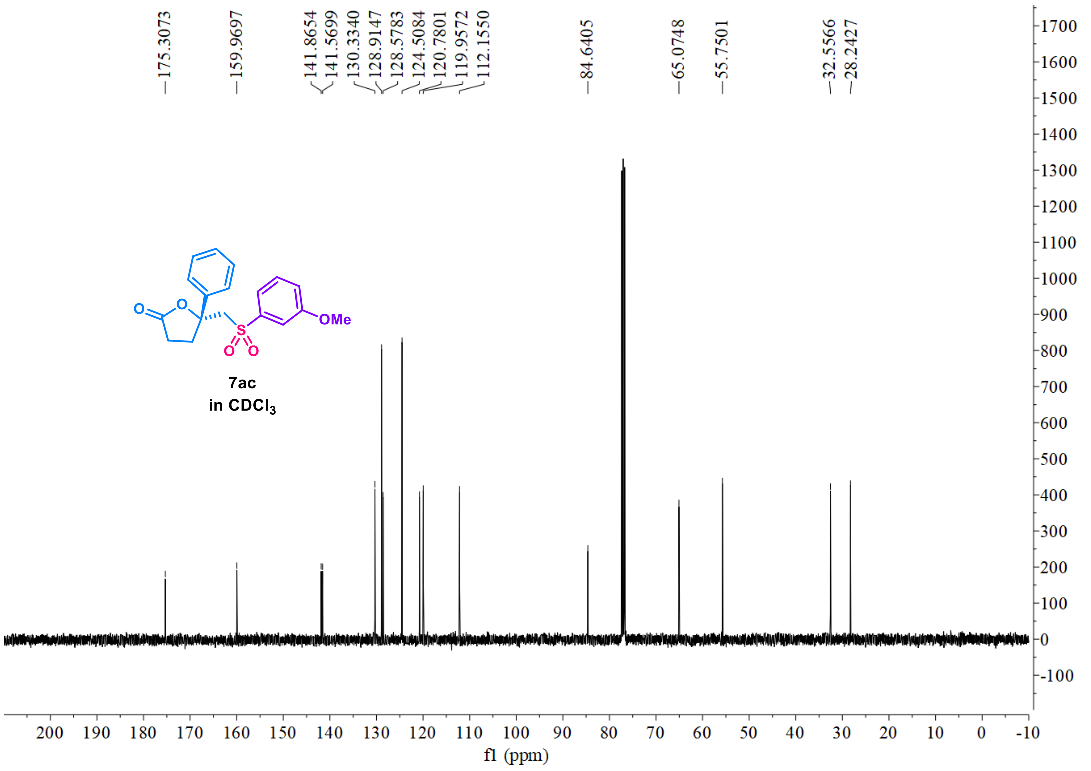
175.3073
159.9697
141.8654
141.5699
130.3340
128.9147
128.5783
124.5084
120.7801
119.9572
112.1550
84.6405
65.0748
55.7501
32.5566
28.2427
OMe
7ac
in $CDCl_3$
f1 (ppm)

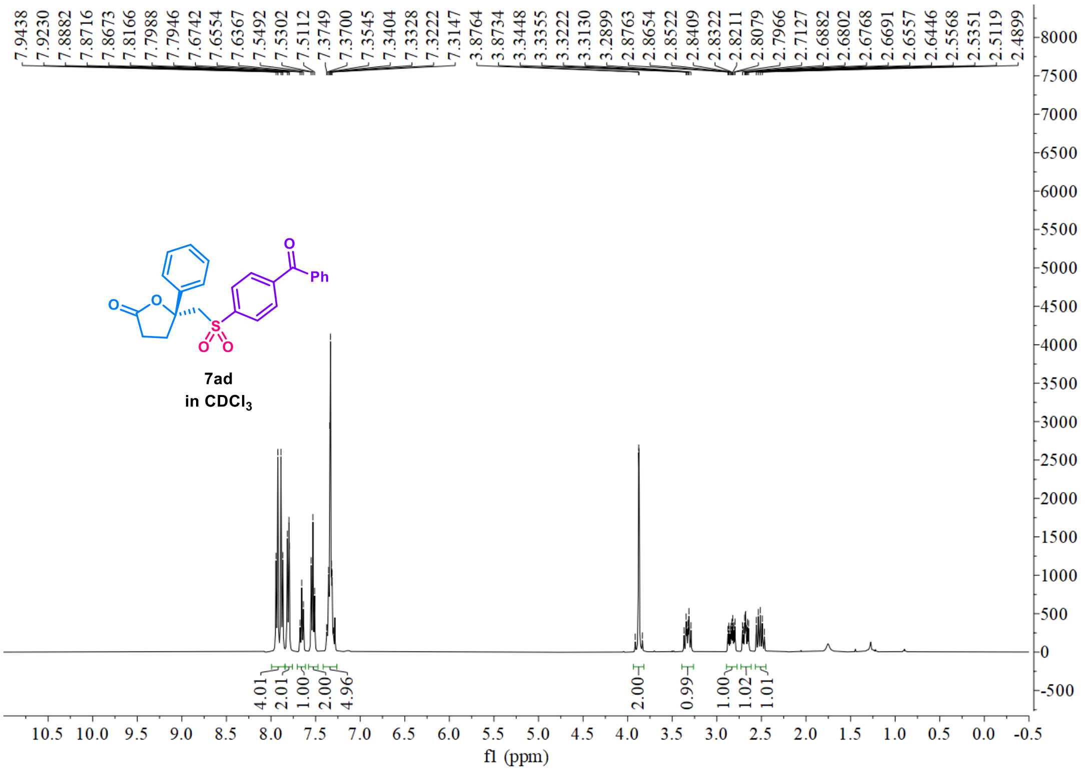
7.9438
7.9230
7.8882
7.8716
7.8673
7.8166
7.7988
7.7946
7.6742
7.6554
7.6367
7.5492
7.5302
7.5112
7.3749
7.3700
7.3545
7.3404
7.3328
7.3222
7.3147
3.8764
3.8734
3.3448
3.3355
3.3222
3.3130
3.2899
2.8763
2.8654
2.8522
2.8409
2.8322
2.8211
2.8079
2.7966
2.7127
2.6882
2.6802
2.6768
2.6691
2.6557
2.6446
2.5568
2.5351
2.5119
2.4899
Ph
7ad
in $CDCl_3$
4.01
2.01
1.00
2.00
4.96
2.00
0.99
1.00
1.02
1.01
f1 (ppm)

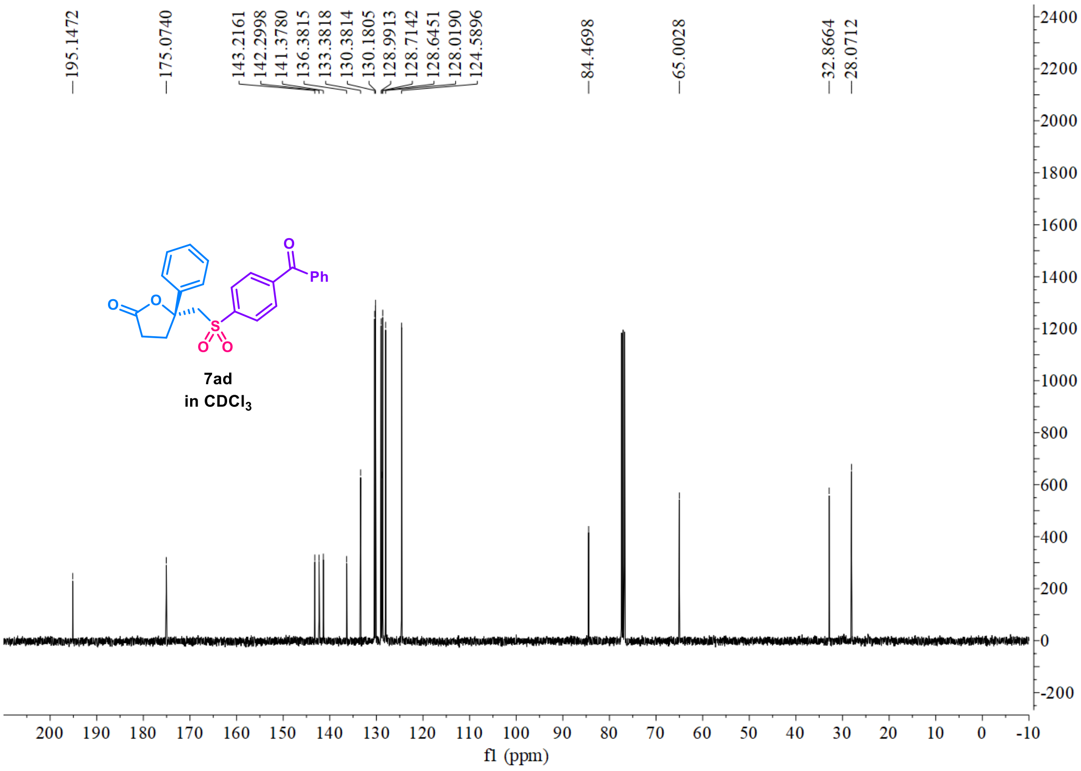

7ad
in $CDCl_3$

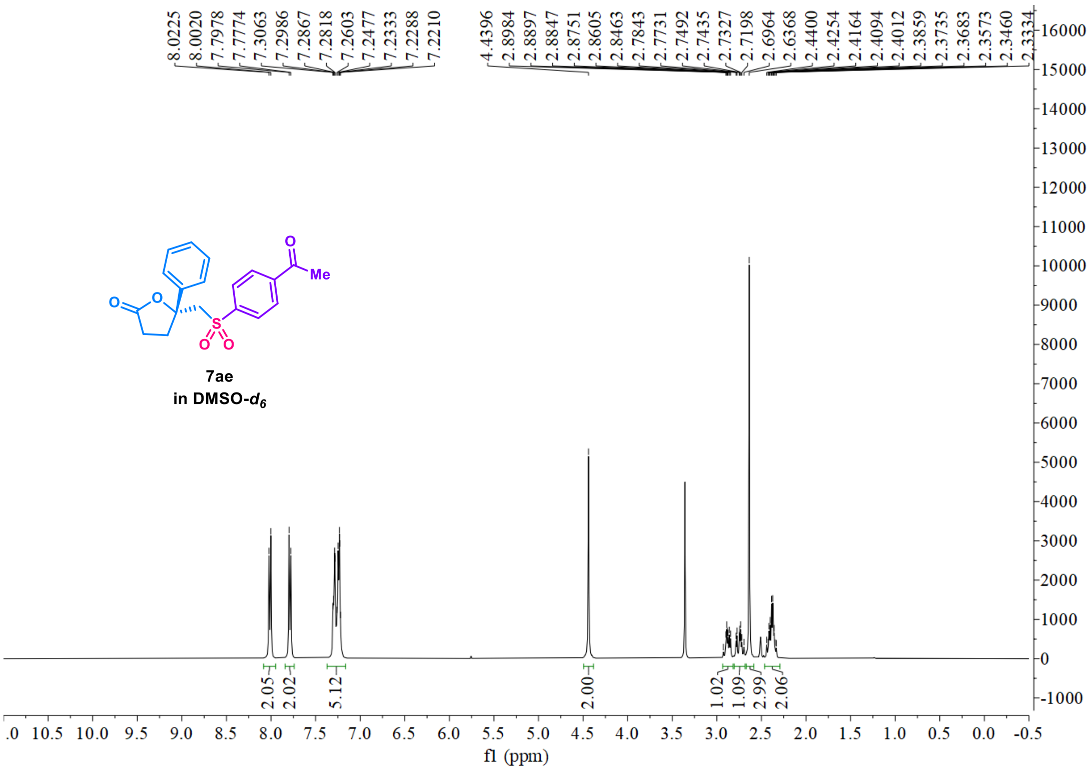

7ae
in DMSO-d_6

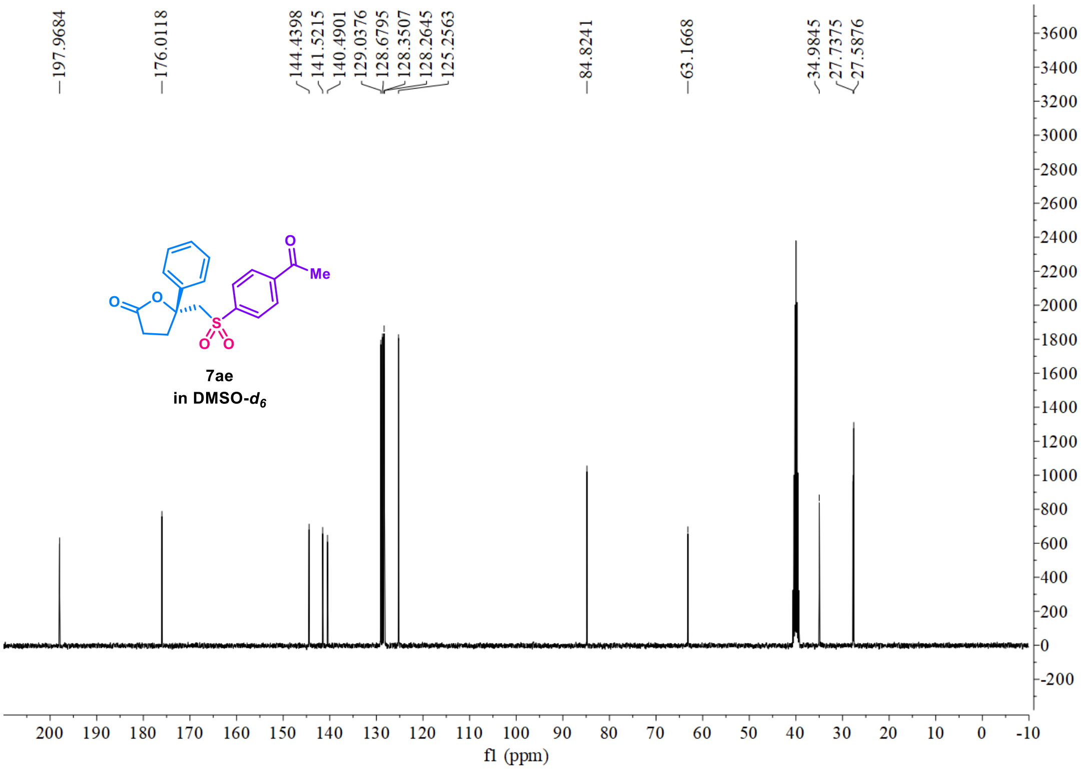
197.9684
176.0118
144.4398
141.5215
140.4901
129.0376
128.6795
128.3507
128.2645
125.2563
84.8241
63.1668
34.9845
27.7375
27.5876
O
Me
O
O
S
O
O
7ae
in DMSO-d_6
f1 (ppm)

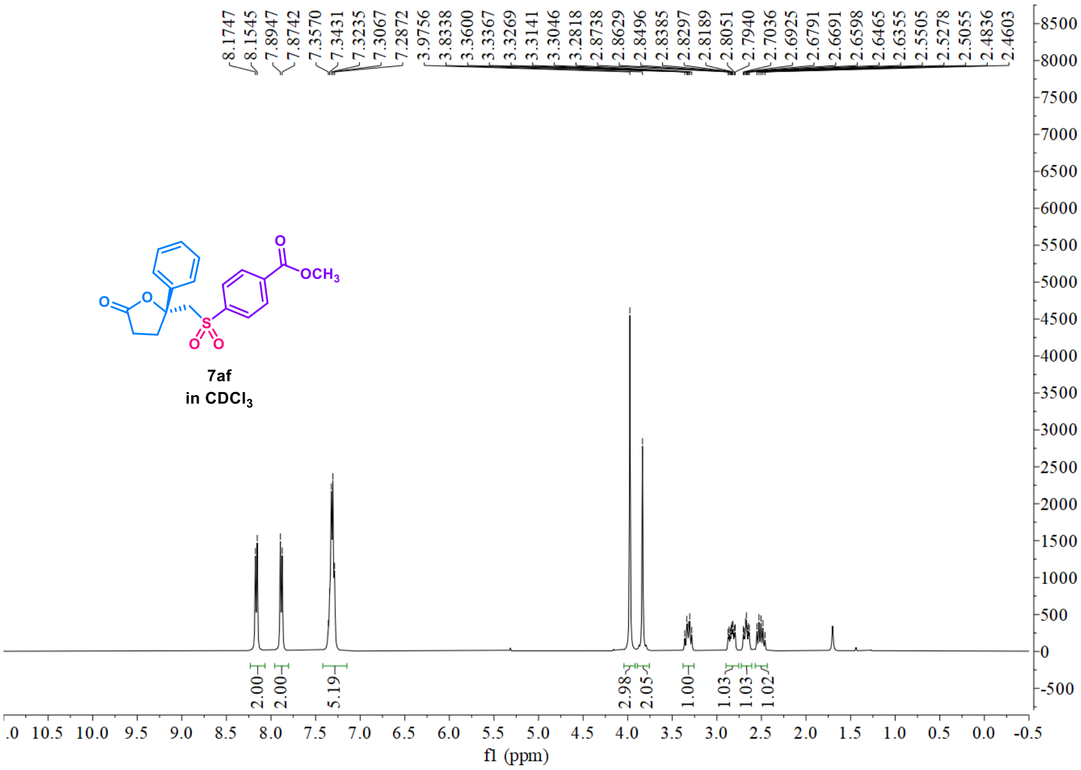
8.1747
8.1545
7.8947
7.8742
7.3570
7.3431
7.3235
7.3067
7.2872
3.9756
3.8338
3.3600
3.3367
3.3269
3.3141
3.3046
3.2818
2.8738
2.8629
2.8496
2.8385
2.8297
2.8189
2.8051
2.7940
2.7036
2.6925
2.6791
2.6691
2.6598
2.6465
2.6355
2.5505
2.5278
2.5055
2.4836
2.4603
O
OCH_3
O
O
S
O
O
7af
in $CDCl_3$
2.00
2.00
5.19
2.98
2.05
1.00
1.03
1.03
1.02
f1 (ppm)

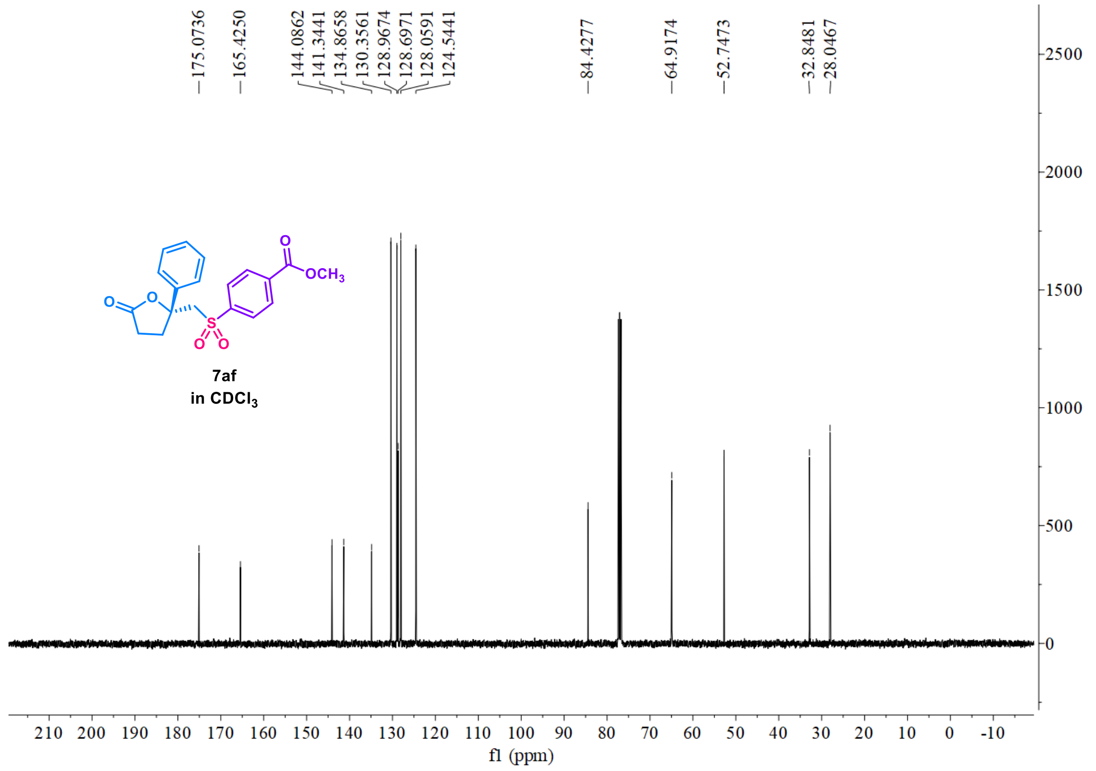
175.0736
165.4250
144.0862
141.3441
134.8658
130.3561
128.9674
128.6971
128.0591
124.5441
84.4277
64.9174
52.7473
32.8481
28.0467
OCH3
7af
in CDCl3
f1 (ppm)

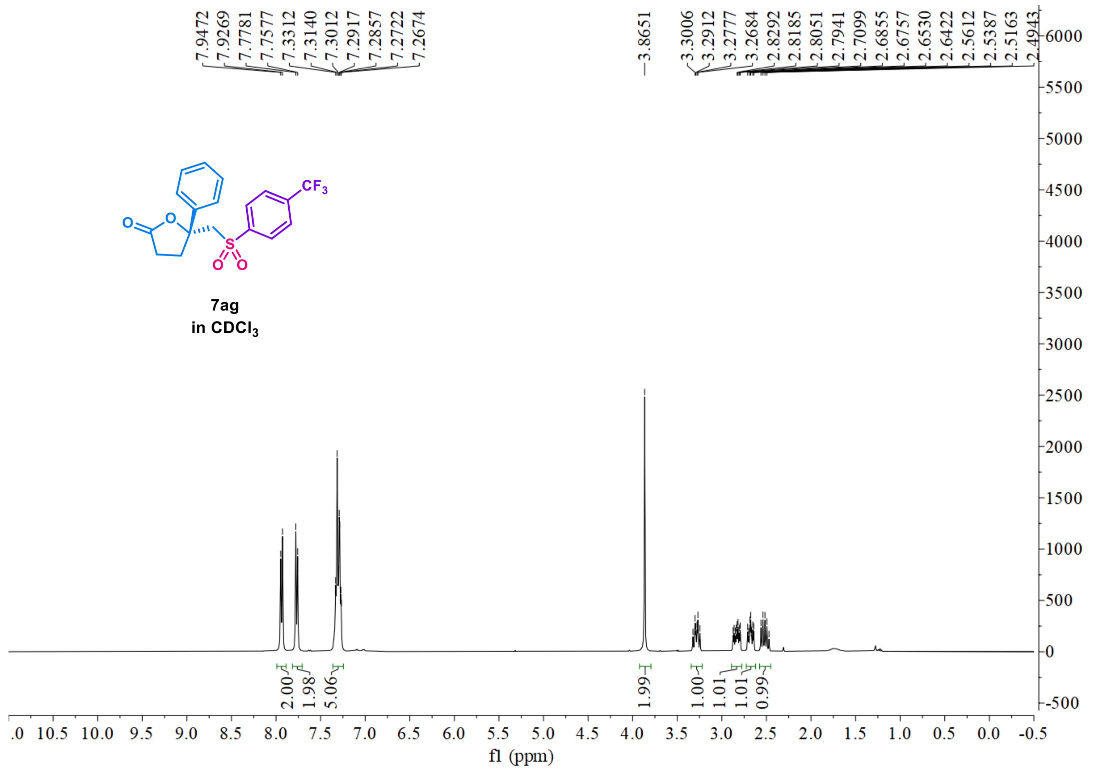
7.9472
7.9269
7.7781
7.7577
7.3312
7.3140
7.3012
7.2917
7.2857
7.2722
7.2674
3.8651
3.3006
3.2912
3.2777
3.2684
2.8292
2.8185
2.8051
2.7941
2.7099
2.6855
2.6757
2.6530
2.6422
2.5612
2.5387
2.5163
2.4943
CF3
7ag
in CDCl3
2.00
1.98
5.06
1.99
1.00
1.01
1.01
0.99
f1 (ppm)

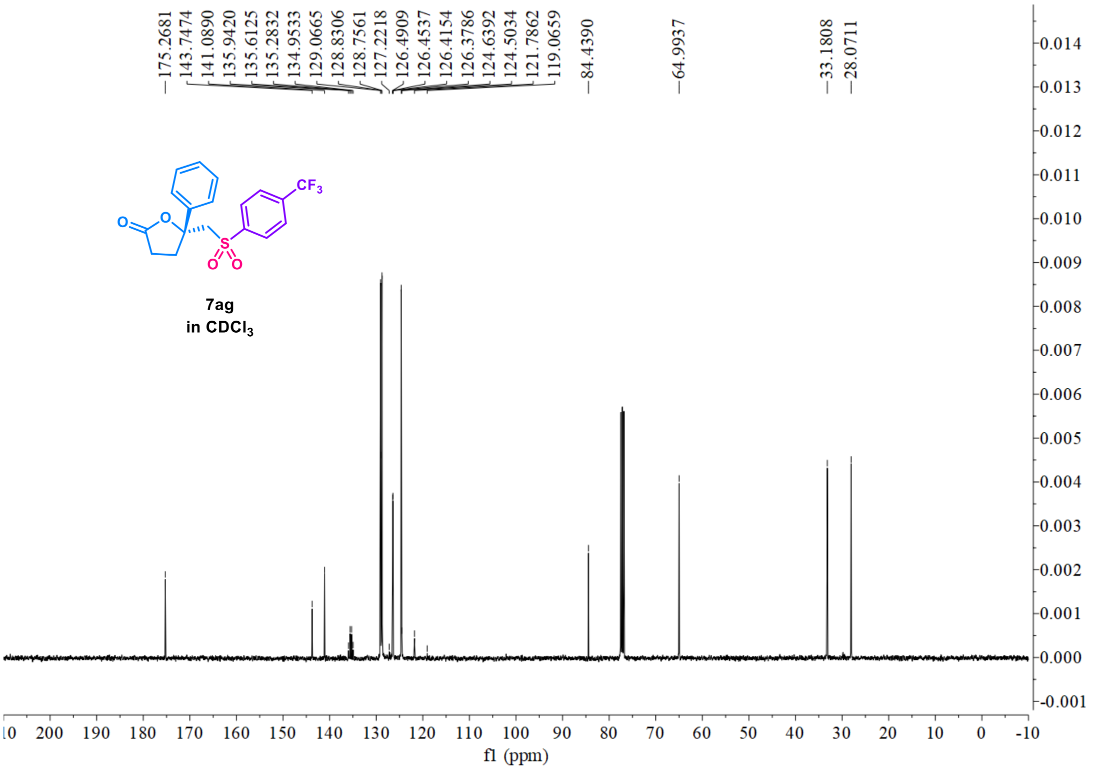
175.2681
143.7474
141.0890
135.9420
135.6125
135.2832
134.9533
129.0665
128.8306
128.7561
127.2218
126.4909
126.4537
126.4154
126.3786
124.6392
124.5034
121.7862
119.0659
84.4390
64.9937
33.1808
28.0711
CF3
O
S
7ag
in CDCl3
f1 (ppm)

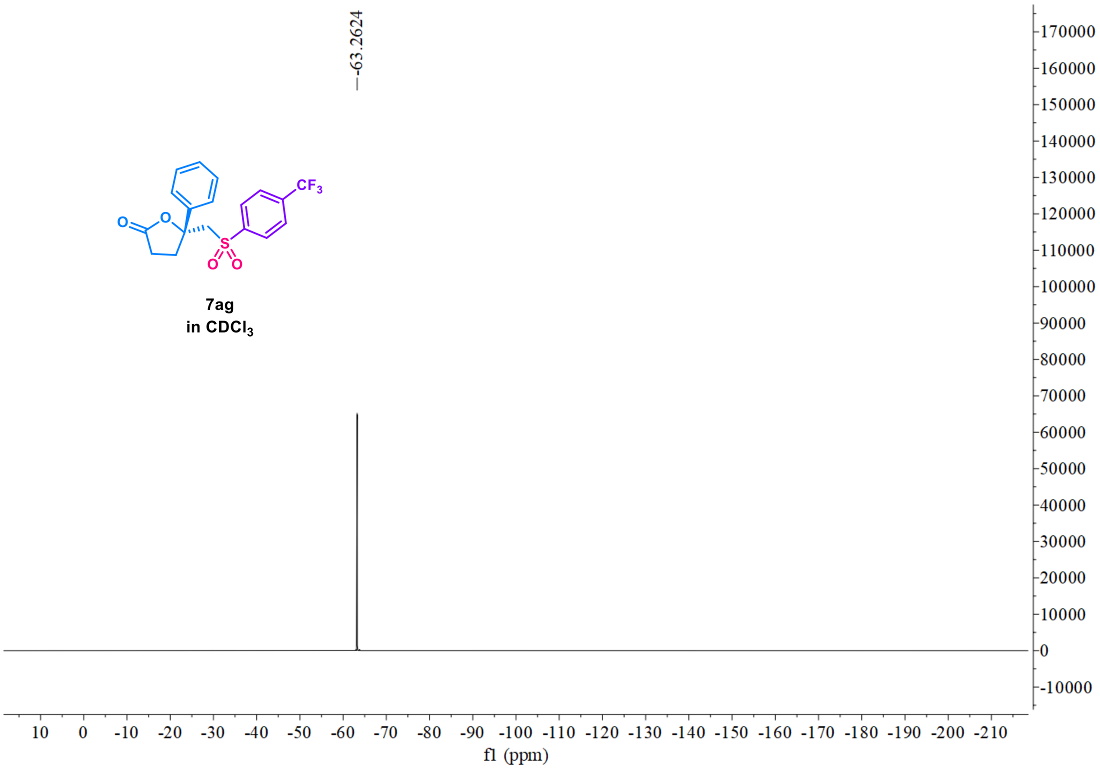
-63.2624
CF3
O
S
7ag
in CDCl3
f1 (ppm)

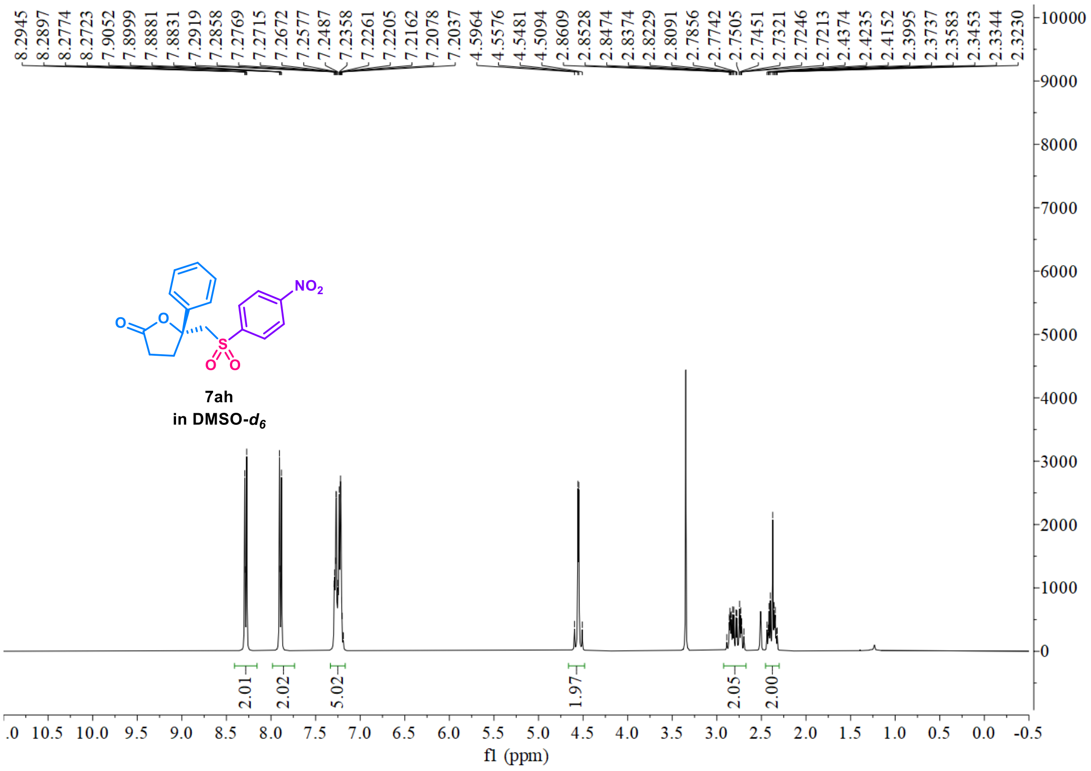
8.2945 8.2897 8.2774 8.2723 7.9052 7.8999 7.8881 7.8831 7.2919 7.2858 7.2769 7.2715 7.2672 7.2577 7.2487 7.2358 7.2261 7.2205 7.2162 7.2078 7.2037 4.5964 4.5576 4.5481 4.5094 2.8609 2.8528 2.8474 2.8374 2.8229 2.8091 2.7856 2.7742 2.7505 2.7451 2.7321 2.7246 2.7213 2.4374 2.4235 2.4152 2.3995 2.3737 2.3583 2.3453 2.3344 2.3230
NO2
O
O
S
O O
7ah
in DMSO-d6
2.01
2.02
5.02
1.97
2.05
2.00
f1 (ppm)

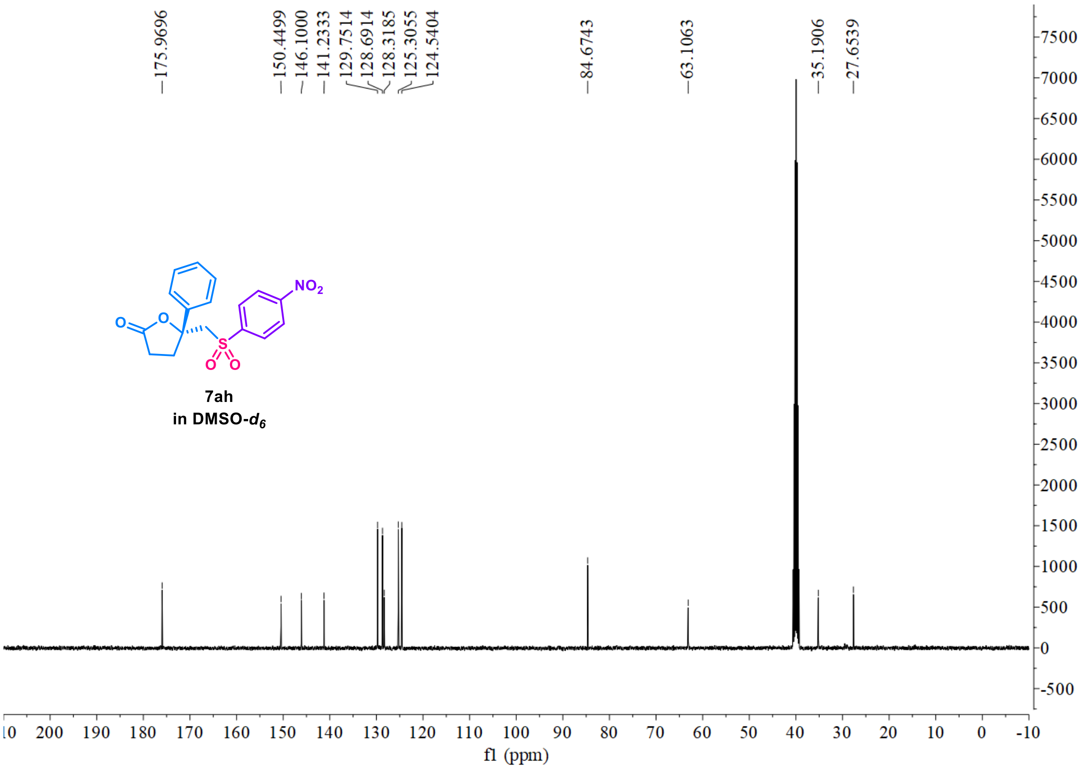
175.9696
150.4499 146.1000 141.2333 129.7514 128.6914 128.3185 125.3055 124.5404
84.6743
63.1063
35.1906 27.6539
NO2
O
O
S
O O
7ah
in DMSO-d6
f1 (ppm)

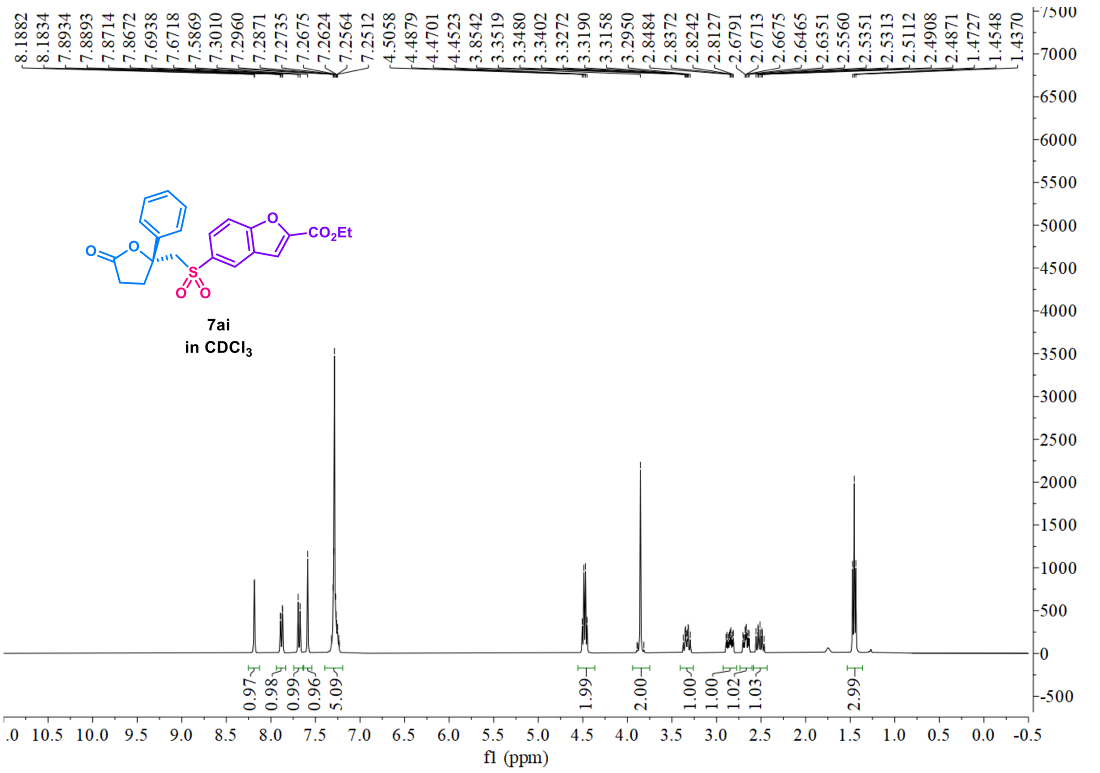
8.1882
8.1834
7.8934
7.8893
7.8714
7.8672
7.6938
7.6718
7.5869
7.3010
7.2960
7.2871
7.2735
7.2675
7.2624
7.2564
7.2512
4.5058
4.4879
4.4701
4.4523
3.8542
3.3519
3.3480
3.3402
3.3272
3.3190
3.3158
3.2950
2.8484
2.8372
2.8242
2.8127
2.6791
2.6713
2.6675
2.6465
2.6351
2.5560
2.5351
2.5313
2.5112
2.4908
2.4871
1.4727
1.4548
1.4370
7ai
in CDCl3
0.97
0.98
0.99
0.96
5.09
1.99
2.00
1.00
1.00
1.02
1.03
2.99
f1 (ppm)

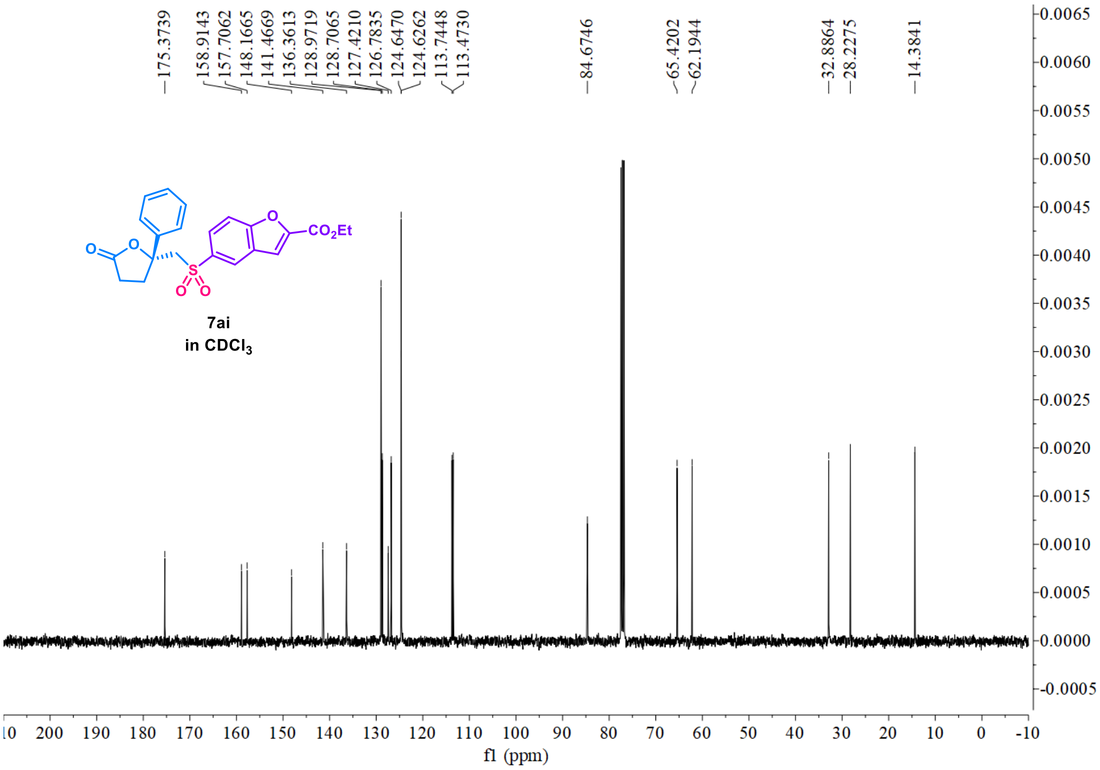
175.3739
158.9143
157.7062
148.1665
141.4669
136.3613
128.9719
128.7065
127.4210
126.7835
124.6470
124.6262
113.7448
113.4730
84.6746
65.4202
62.1944
32.8864
28.2275
14.3841
7ai
in CDCl3
f1 (ppm)

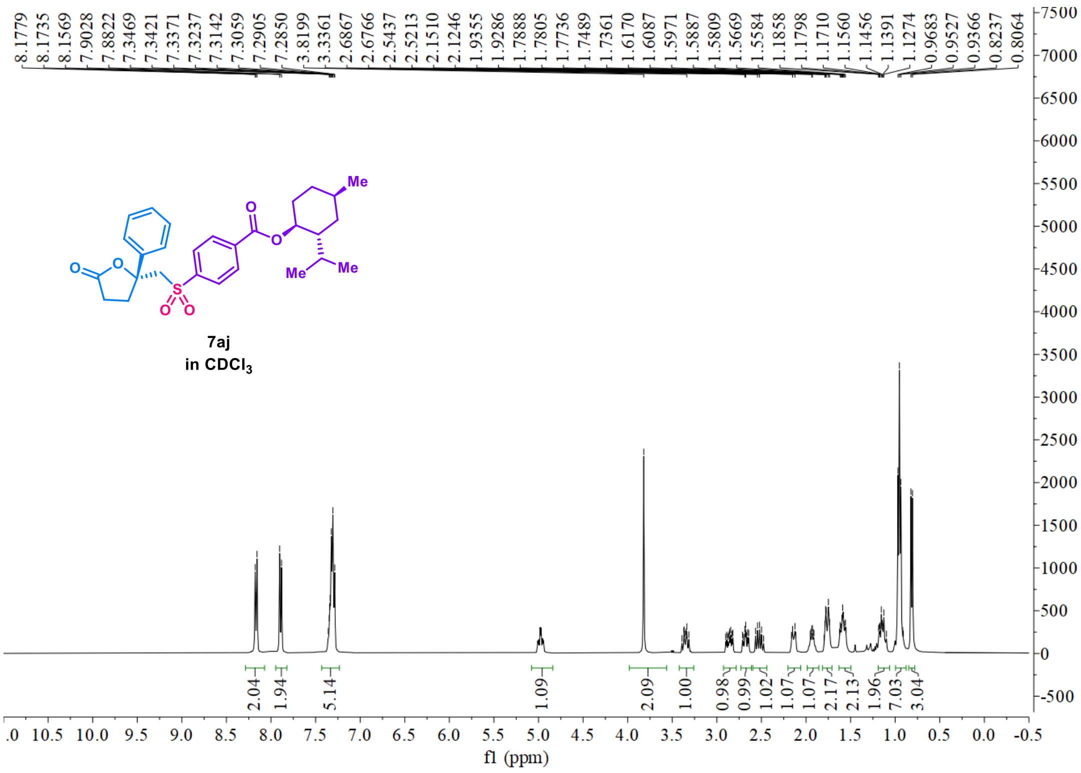
7aj
in CDCl3
f1 (ppm)

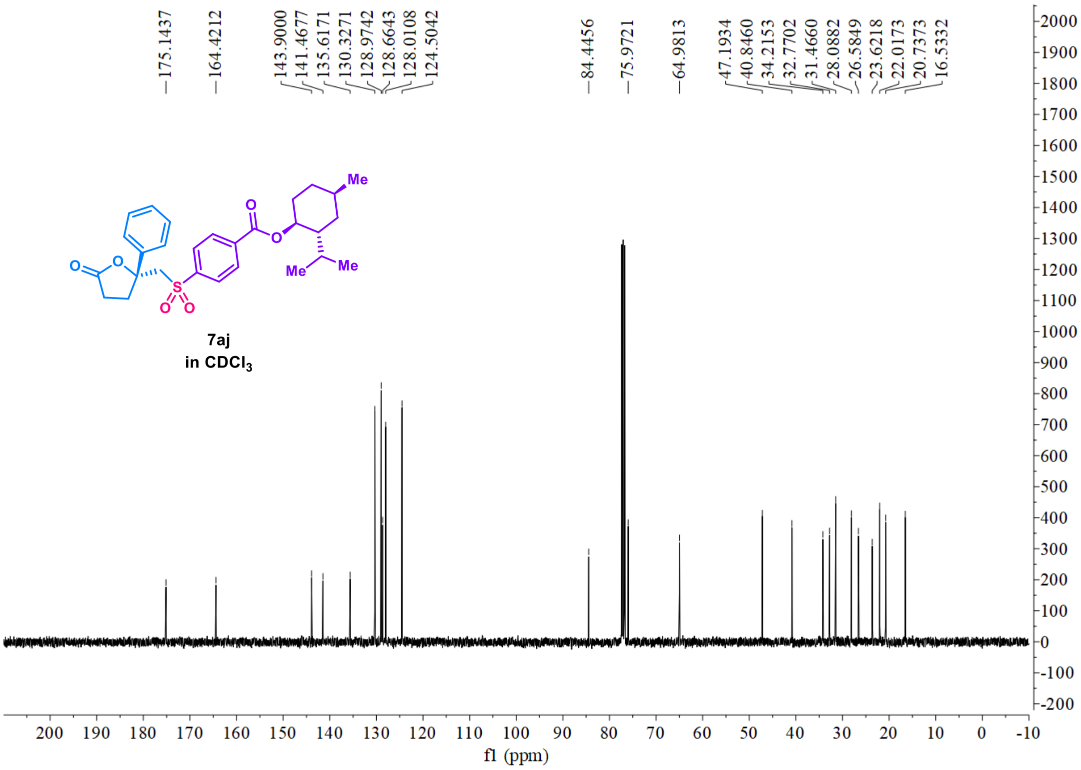
7aj
in CDCl3
f1 (ppm)

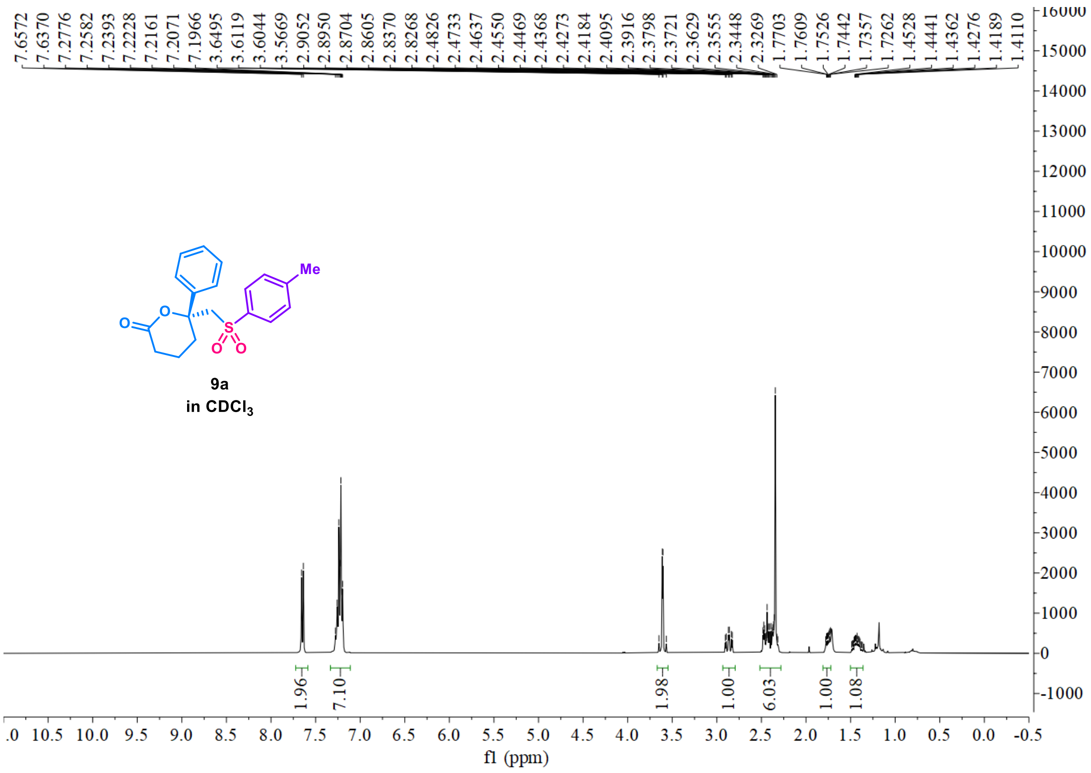
Me
O
O
S
O
O
9a
in CDCl3
1.96
7.10
1.98
1.00
6.03
1.00
1.08
f1 (ppm)

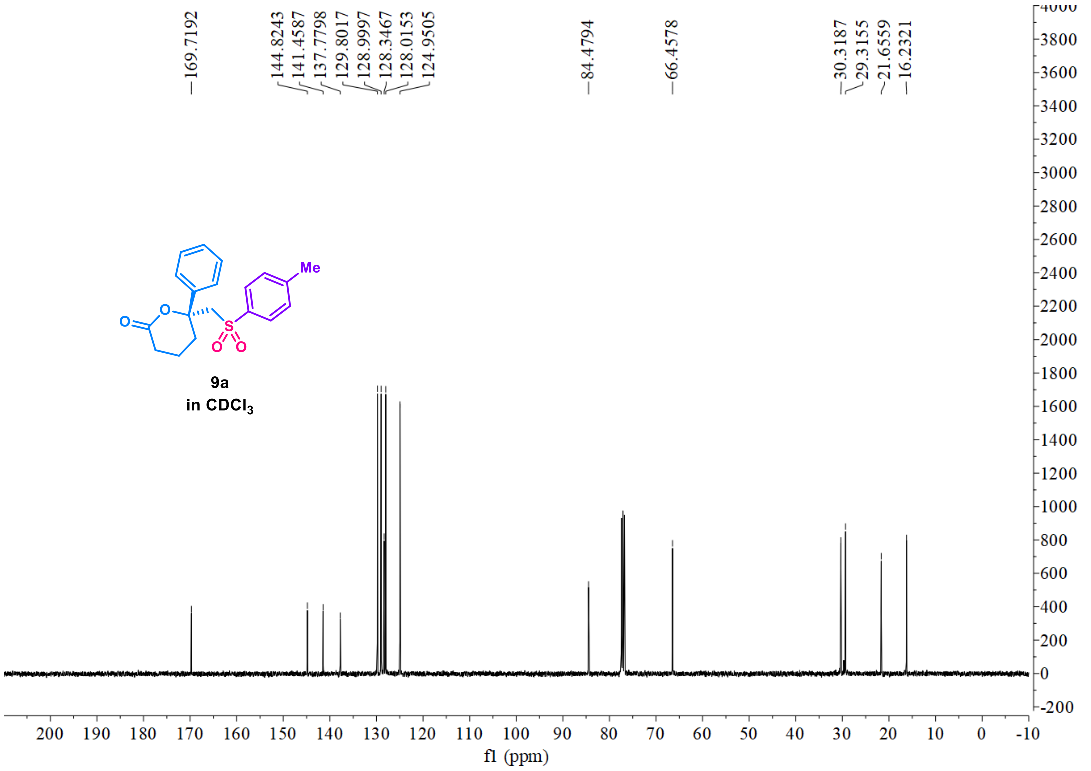
169.7192
144.8243
141.4587
137.7798
129.8017
128.9997
128.3467
128.0153
124.9505
84.4794
66.4578
30.3187
29.3155
21.6559
16.2321
Me
O
O
S
O
O
9a
in CDCl3
f1 (ppm)

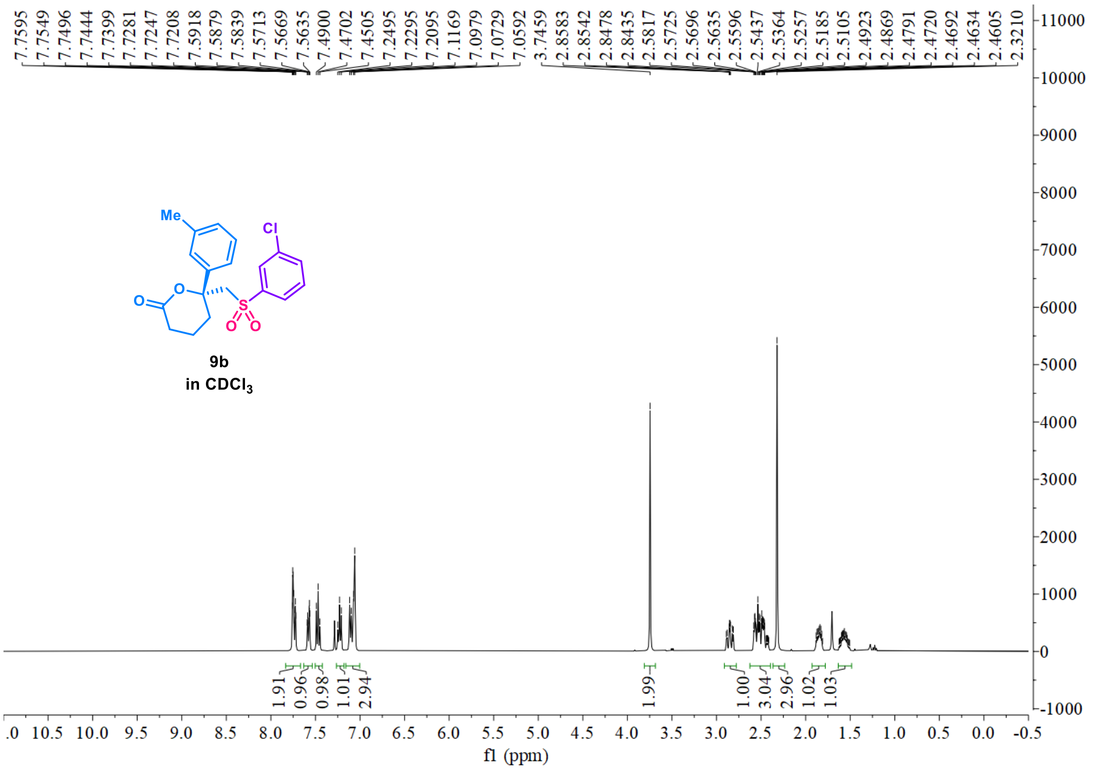
7.7595
7.7549
7.7496
7.7444
7.7399
7.7281
7.7247
7.7208
7.5918
7.5879
7.5839
7.5713
7.5669
7.5635
7.4900
7.4702
7.4505
7.2495
7.2295
7.2095
7.1169
7.0979
7.0729
7.0592
3.7459
2.8583
2.8542
2.8478
2.8435
2.5817
2.5725
2.5696
2.5635
2.5596
2.5437
2.5364
2.5257
2.5185
2.5105
2.4923
2.4869
2.4791
2.4720
2.4692
2.4634
2.4605
2.3210
Me
Cl
O
O
S
O
O
9b
in CDCl3
1.91
0.96
0.98
1.01
2.94
1.99
1.00
3.04
2.96
1.02
1.03
f1 (ppm)

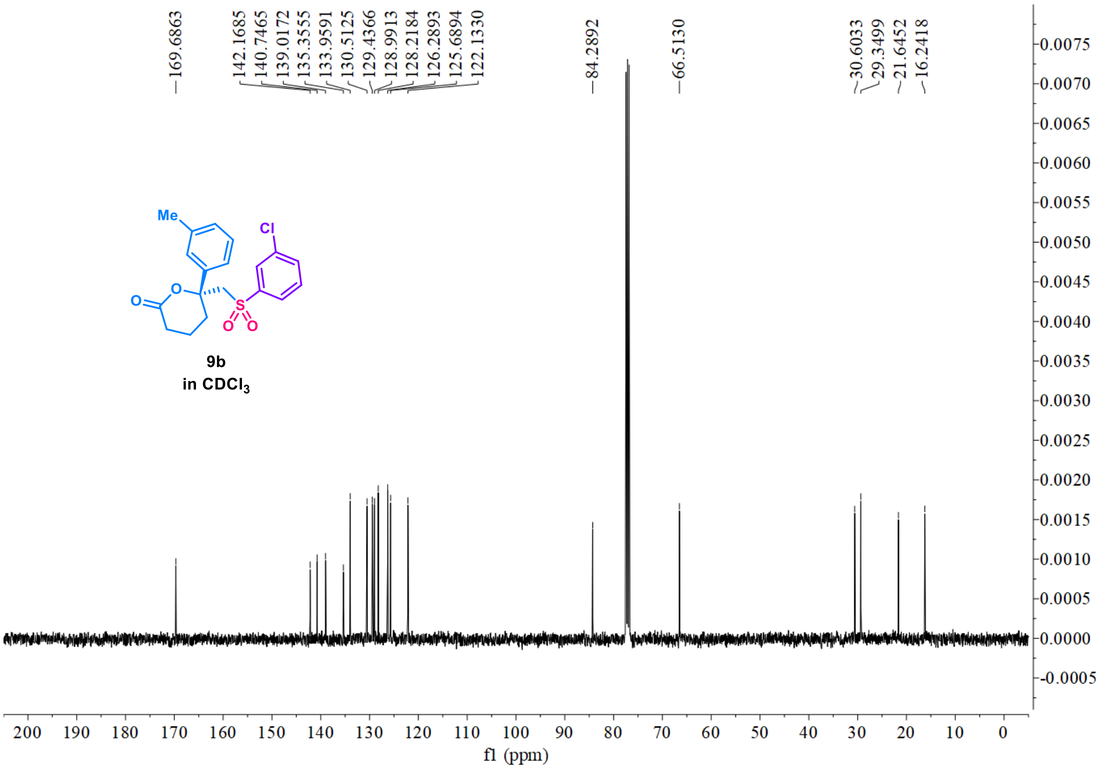
169.6863
142.1685
140.7465
139.0172
135.3555
133.9591
130.5125
129.4366
128.9913
128.2184
126.2893
125.6894
122.1330
84.2892
66.5130
30.6033
29.3499
21.6452
16.2418
Me
Cl
O
O
S
O
O
9b
in CDCl3
f1 (ppm)

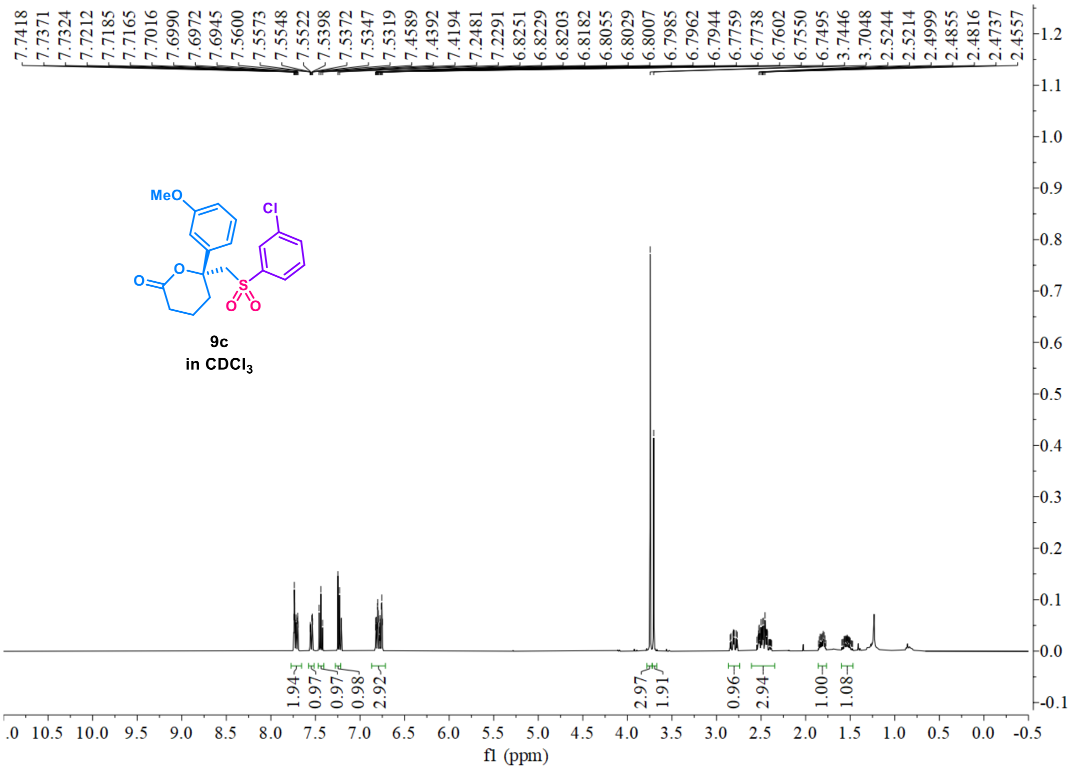
7.7418
7.7371
7.7324
7.7212
7.7185
7.7165
7.7016
7.6990
7.6972
7.6945
7.5600
7.5573
7.5548
7.5522
7.5398
7.5372
7.5347
7.5319
7.4589
7.4392
7.4194
7.2481
7.2291
6.8251
6.8229
6.8203
6.8182
6.8055
6.8029
6.8007
6.7985
6.7962
6.7944
6.7759
6.7738
6.7602
6.7550
6.7495
3.7446
3.7048
2.5244
2.5214
2.4999
2.4855
2.4816
2.4737
2.4557
MeO
Cl
O
O
S
O
O
9c
in $CDCl_3$
1.94
0.97
0.97
0.98
2.92
2.97
1.91
0.96
2.94
1.00
1.08
f1 (ppm)

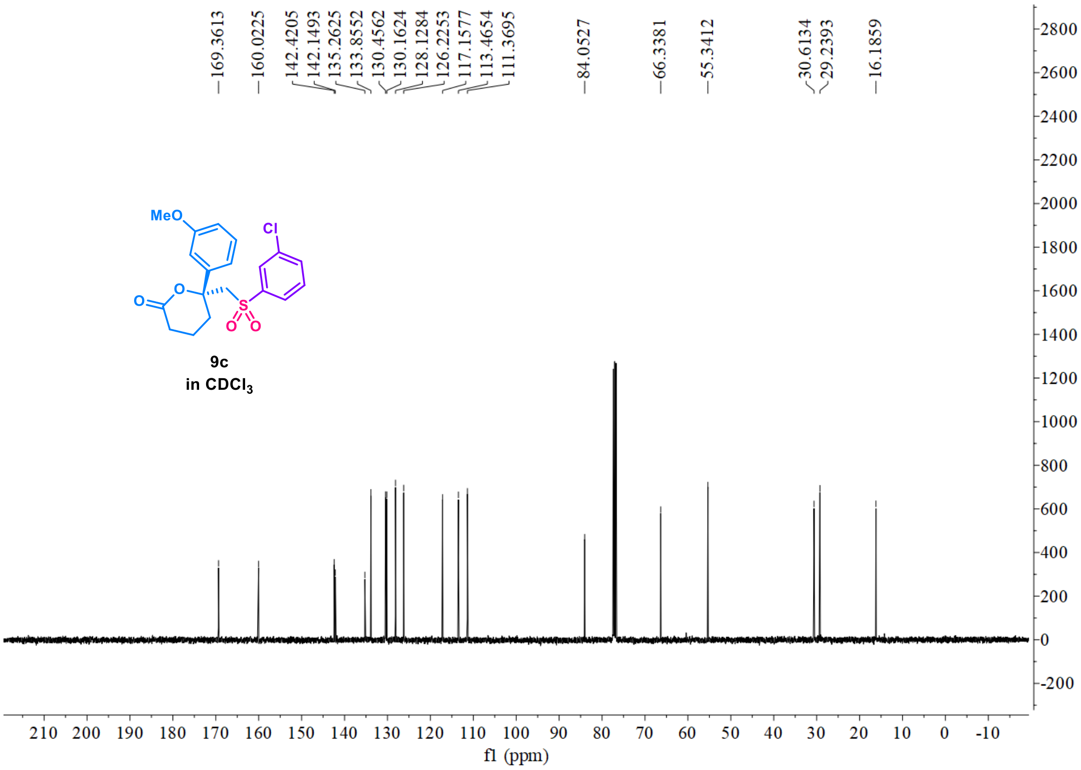
169.3613
160.0225
142.4205
142.1493
135.2625
133.8552
130.4562
130.1624
128.1284
126.2253
117.1577
113.4654
111.3695
84.0527
66.3381
55.3412
30.6134
29.2393
16.1859
MeO
Cl
O
O
S
O
O
9c
in $CDCl_3$
f1 (ppm)

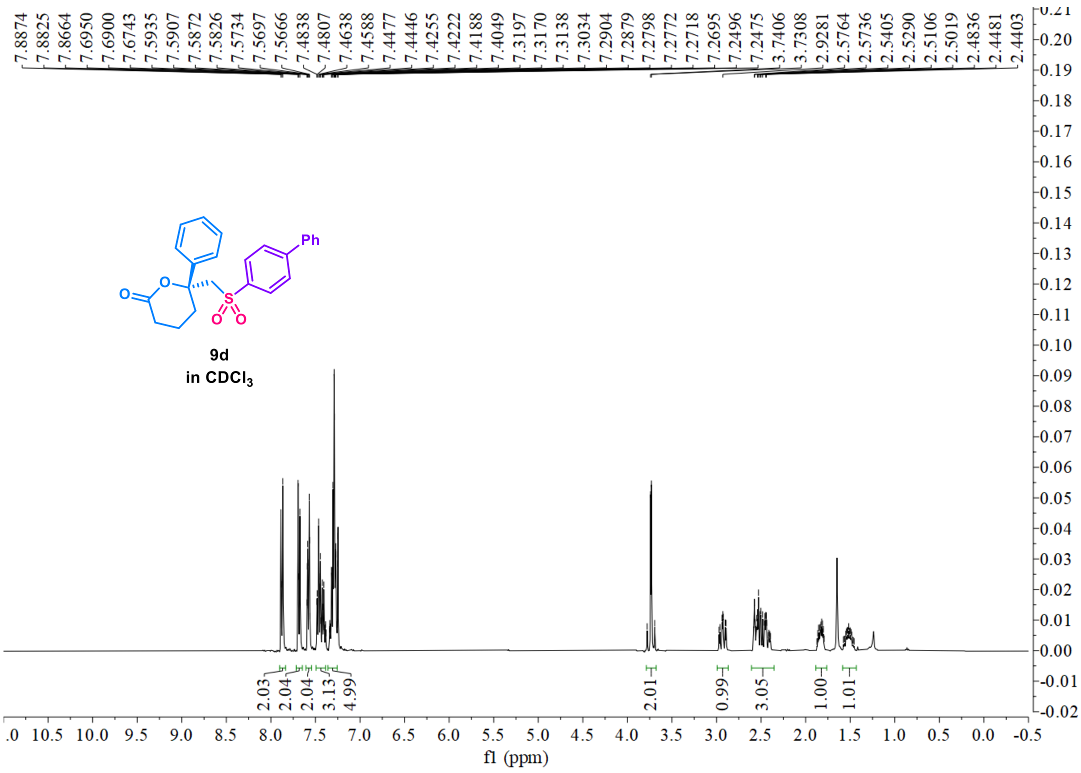
7.8874
7.8825
7.8664
7.6950
7.6900
7.6743
7.5935
7.5907
7.5872
7.5826
7.5734
7.5697
7.5666
7.4838
7.4807
7.4638
7.4588
7.4477
7.4446
7.4255
7.4222
7.4188
7.4049
7.3197
7.3170
7.3138
7.3034
7.2904
7.2879
7.2798
7.2772
7.2718
7.2695
7.2496
7.2475
3.7406
3.7308
2.9281
2.5764
2.5736
2.5405
2.5290
2.5106
2.5019
2.4836
2.4481
2.4403
Ph
O
O
S
O
O
9d
in CDCl3
2.03
2.04
2.04
3.13
4.99
2.01
0.99
3.05
1.00
1.01
f1 (ppm)

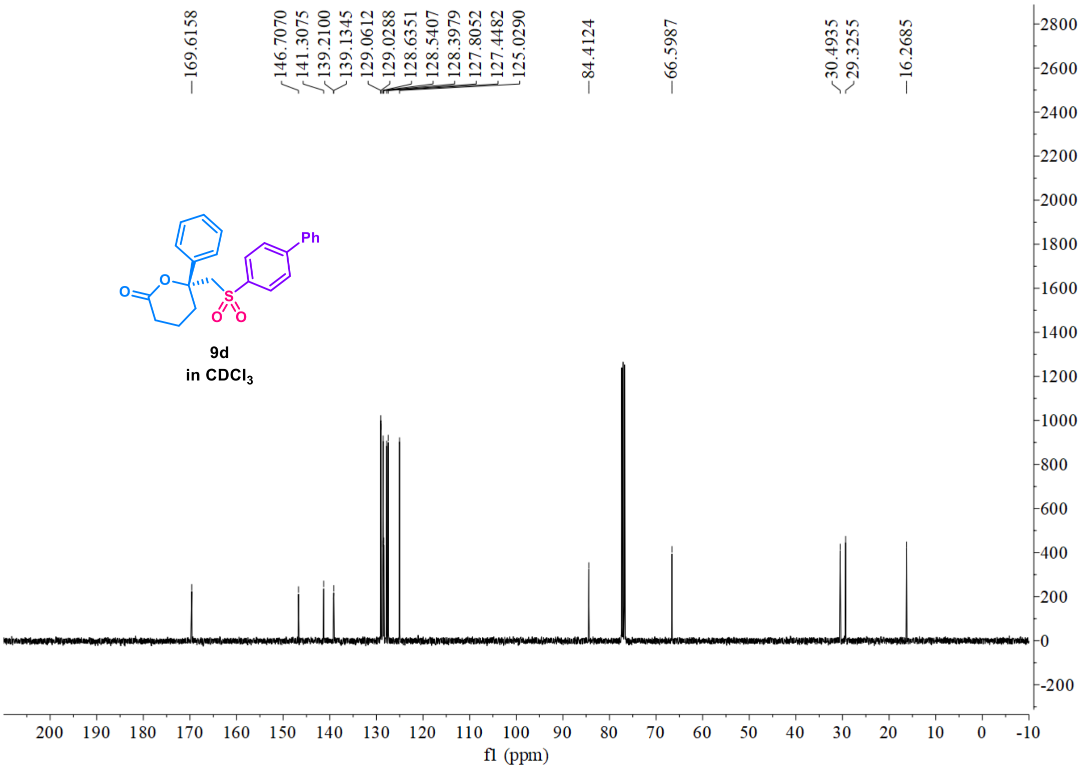
169.6158
146.7070
141.3075
139.2100
139.1345
129.0612
129.0288
128.6351
128.5407
128.3979
127.8052
127.4482
125.0290
84.4124
66.5987
30.4935
29.3255
16.2685
Ph
O
O
S
O
O
9d
in CDCl3
f1 (ppm)

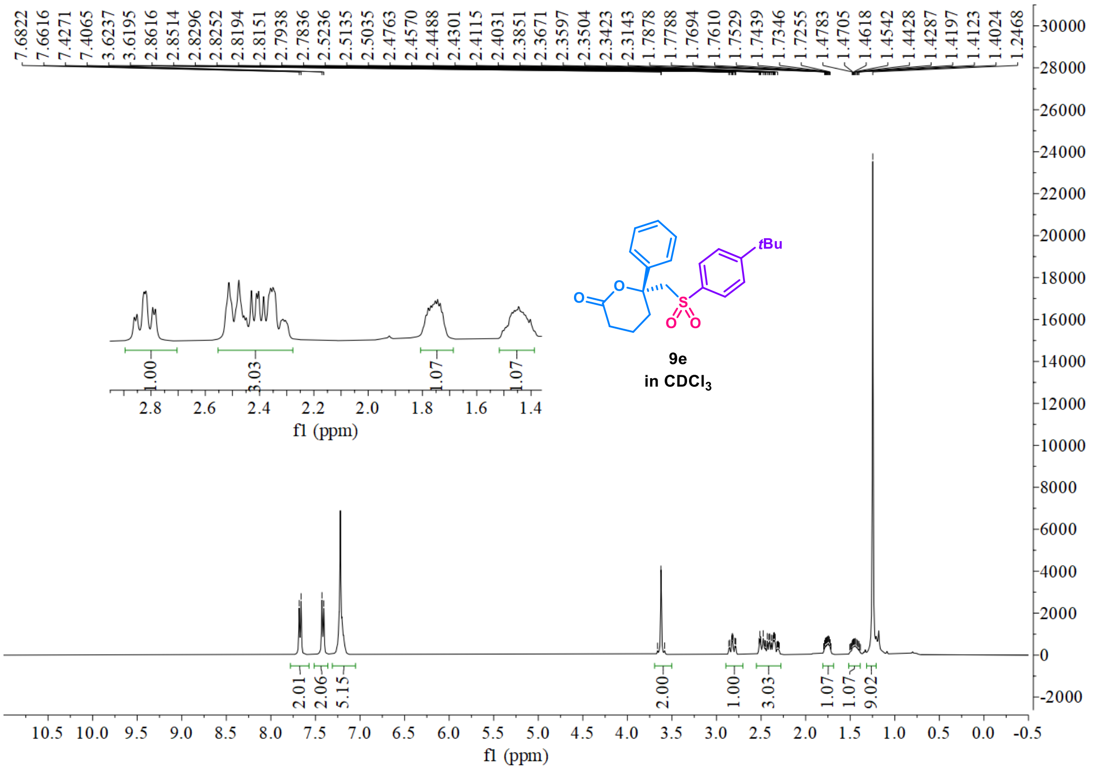
7.6822
7.6616
7.4271
7.4065
3.6237
3.6195
2.8616
2.8514
2.8296
2.8252
2.8194
2.8151
2.7938
2.7836
2.5236
2.5135
2.5035
2.4763
2.4570
2.4488
2.4301
2.4115
2.4031
2.3851
2.3671
2.3597
2.3504
2.3423
2.3143
1.7878
1.7788
1.7694
1.7610
1.7529
1.7439
1.7346
1.7255
1.4783
1.4705
1.4618
1.4542
1.4428
1.4287
1.4197
1.4123
1.4024
1.2468
tBu
9e
in $CDCl_3$
f1 (ppm)

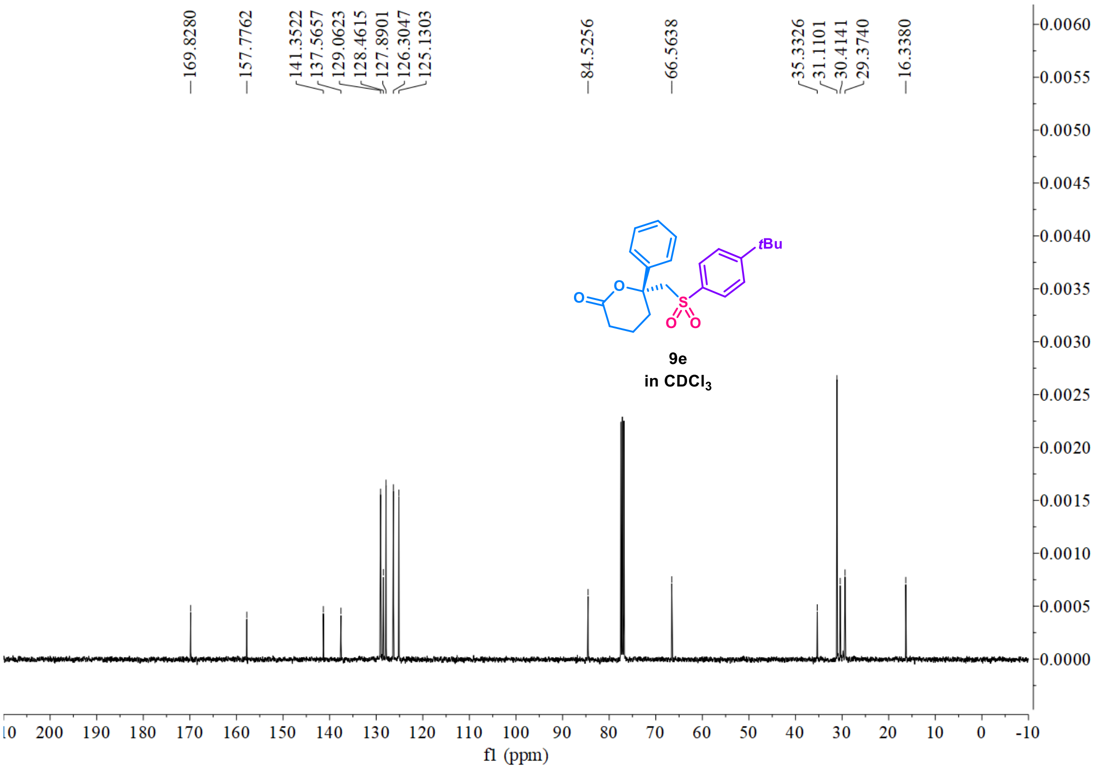
169.8280
157.7762
141.3522
137.5657
129.0623
128.4615
127.8901
126.3047
125.1303
84.5256
66.5638
35.3326
31.1101
30.4141
29.3740
16.3380
tBu
9e
in $CDCl_3$
f1 (ppm)

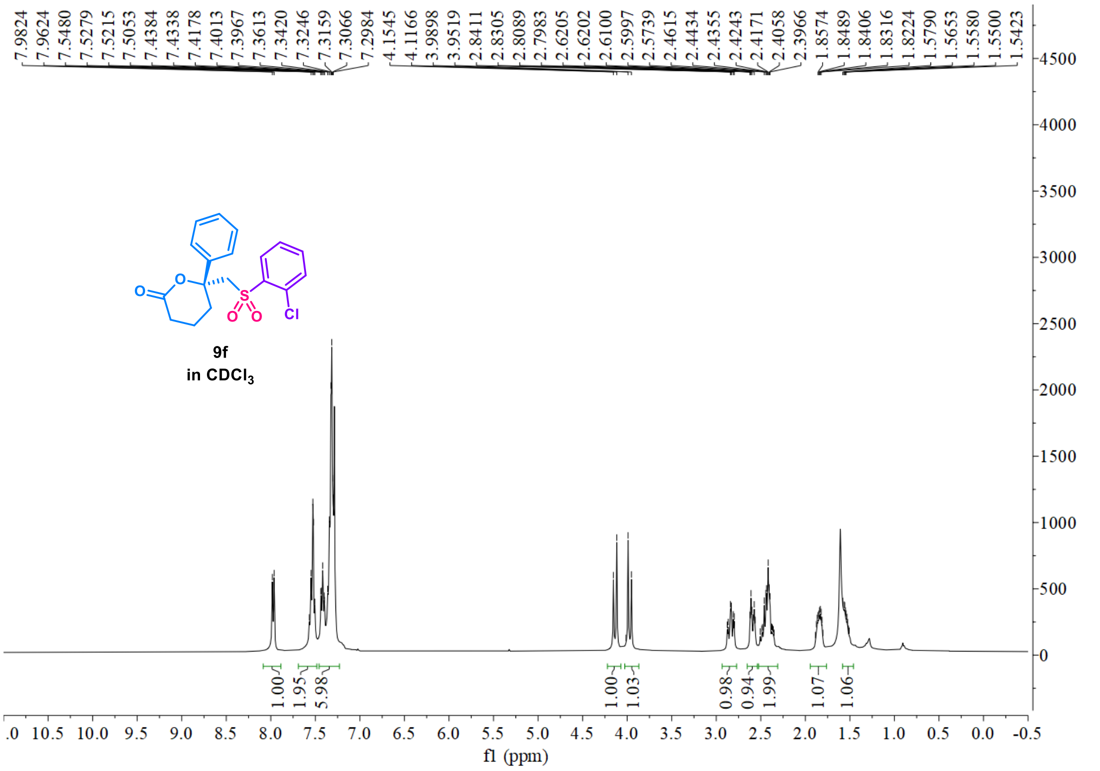
9f
in CDCl3
1.00
1.95
5.98
1.00
1.03
0.98
0.94
1.99
1.07
1.06
f1 (ppm)

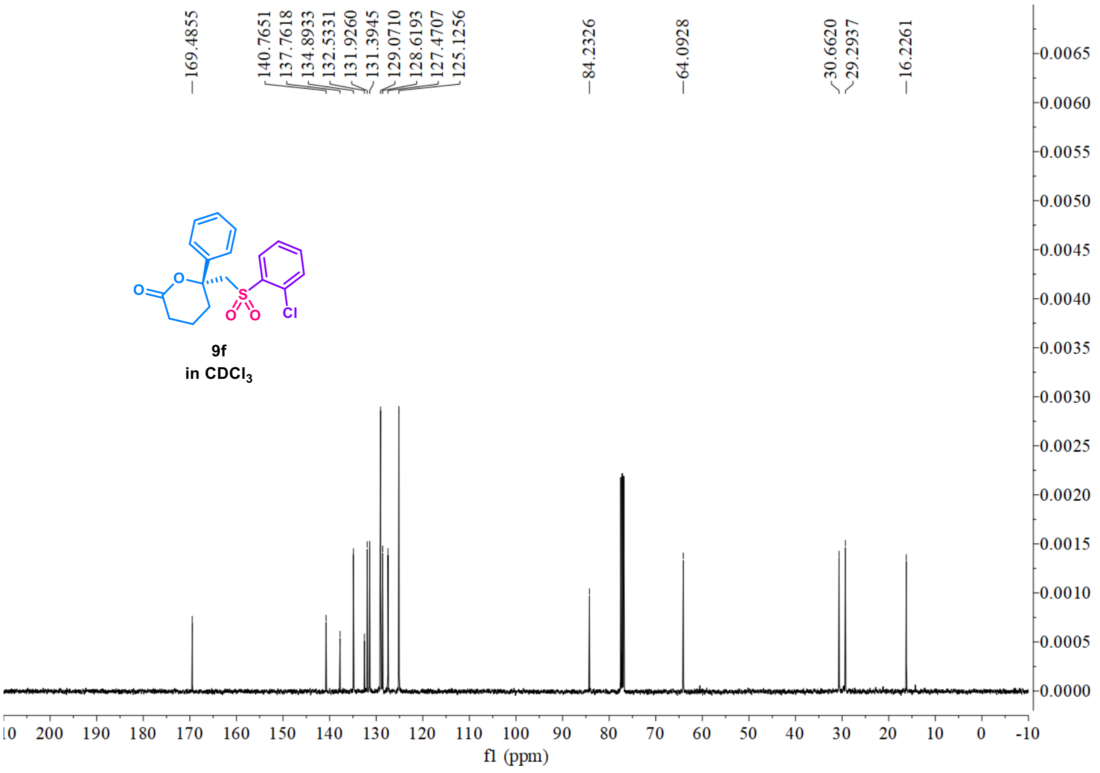
169.4855
140.7651
137.7618
134.8933
132.5331
131.9260
131.3945
129.0710
128.6193
127.4707
125.1256
84.2326
64.0928
30.6620
29.2937
16.2261
9f
in CDCl3
f1 (ppm)

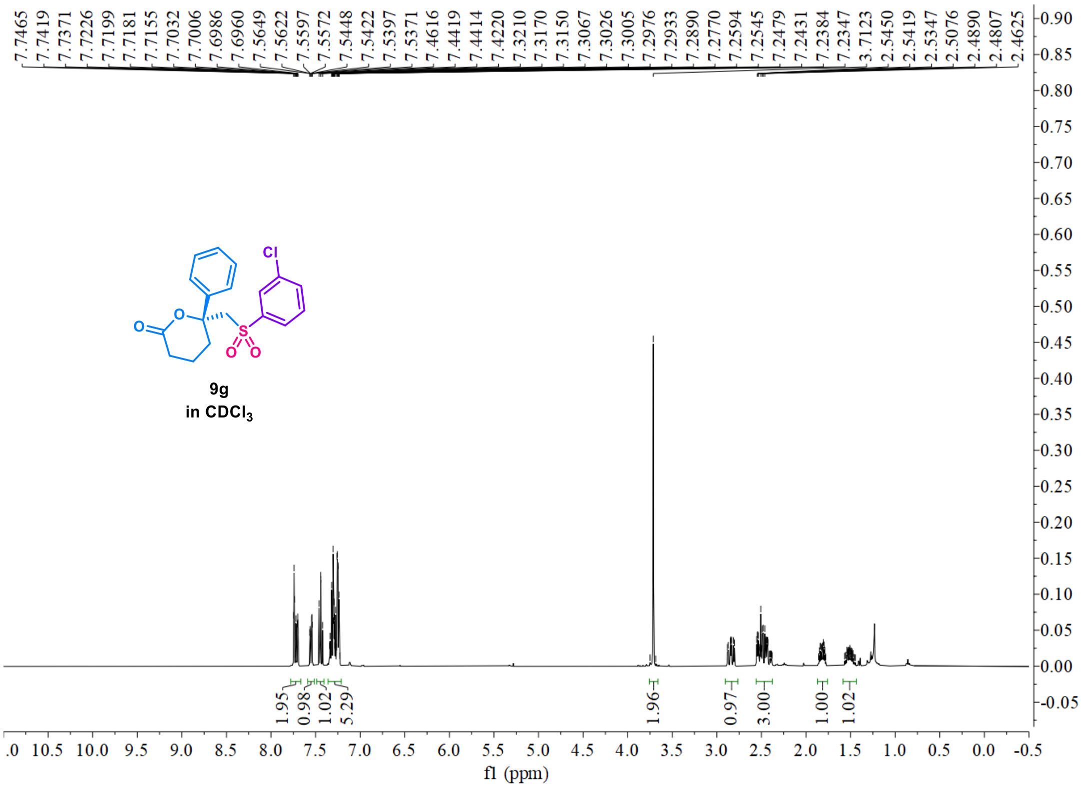

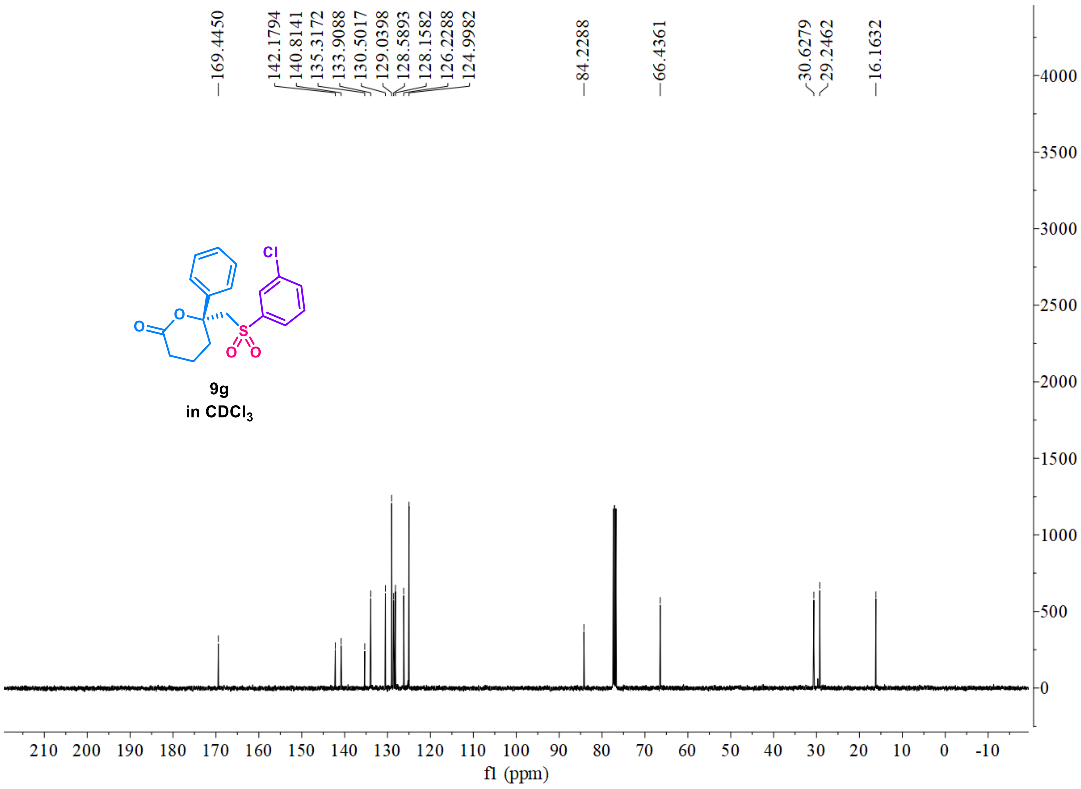

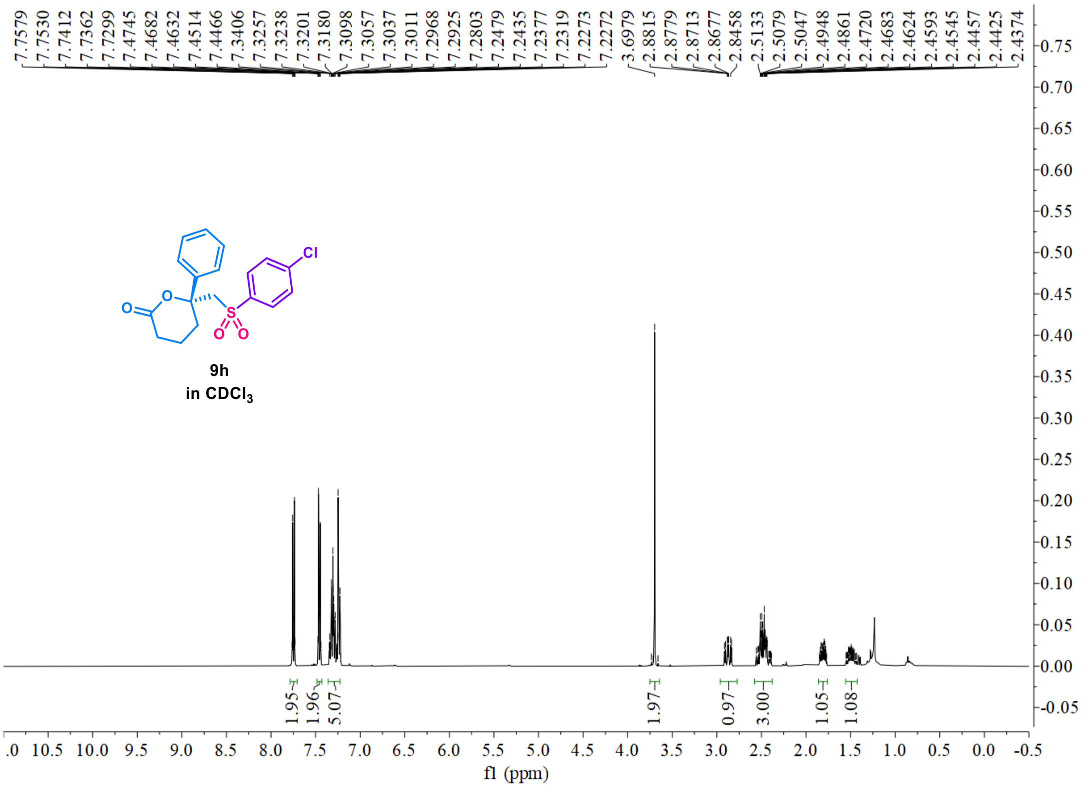
9h
in CDCl3
f1 (ppm)

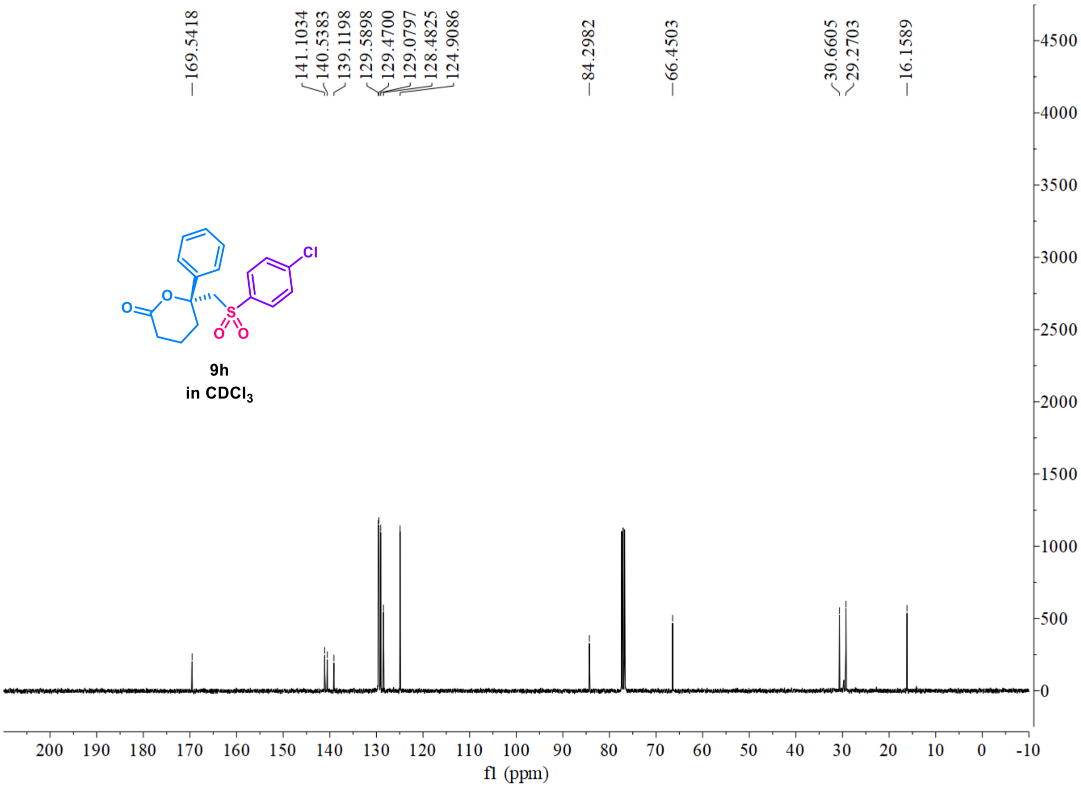
9h
in CDCl3
f1 (ppm)

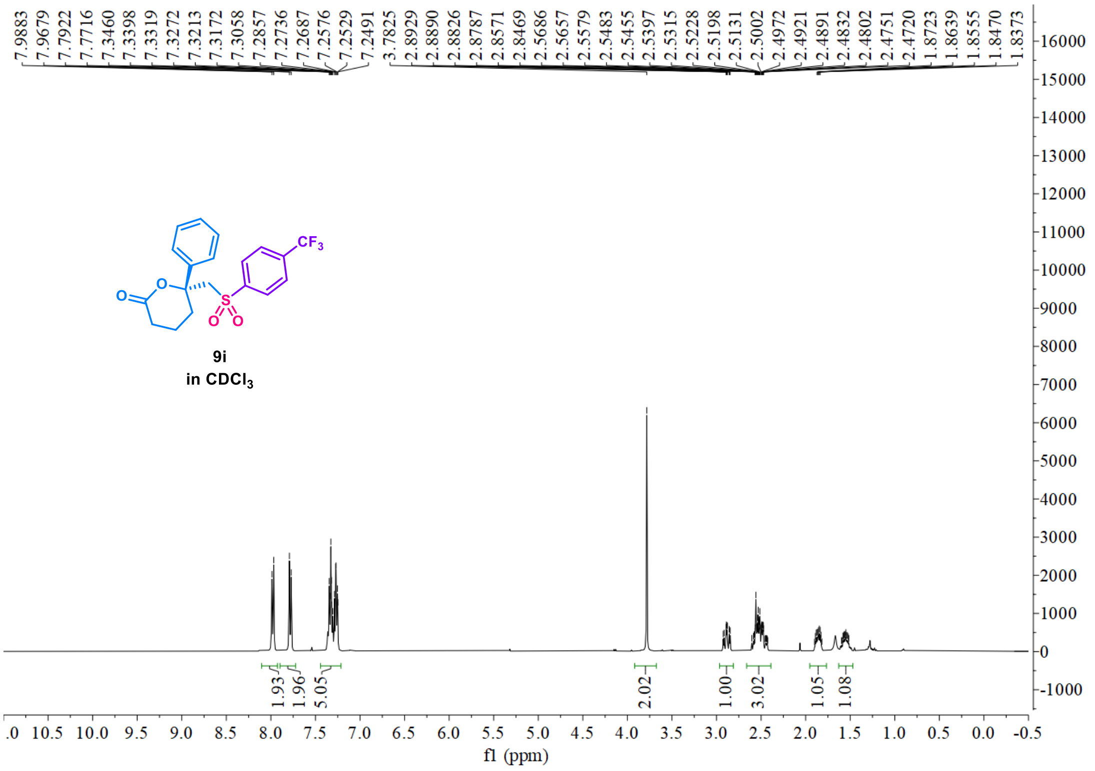
7.9883
7.9679
7.7922
7.7716
7.3460
7.3398
7.3319
7.3272
7.3213
7.3172
7.3058
7.2857
7.2736
7.2687
7.2576
7.2529
7.2491
3.7825
2.8929
2.8890
2.8826
2.8787
2.8571
2.8469
2.5686
2.5657
2.5579
2.5483
2.5455
2.5397
2.5315
2.5228
2.5198
2.5131
2.5002
2.4972
2.4921
2.4891
2.4832
2.4802
2.4751
2.4720
1.8723
1.8639
1.8555
1.8470
1.8373
CF3
O
O
S
O O
9i
in CDCl3
1.93
1.96
5.05
2.02
1.00
3.02
1.05
1.08
f1 (ppm)

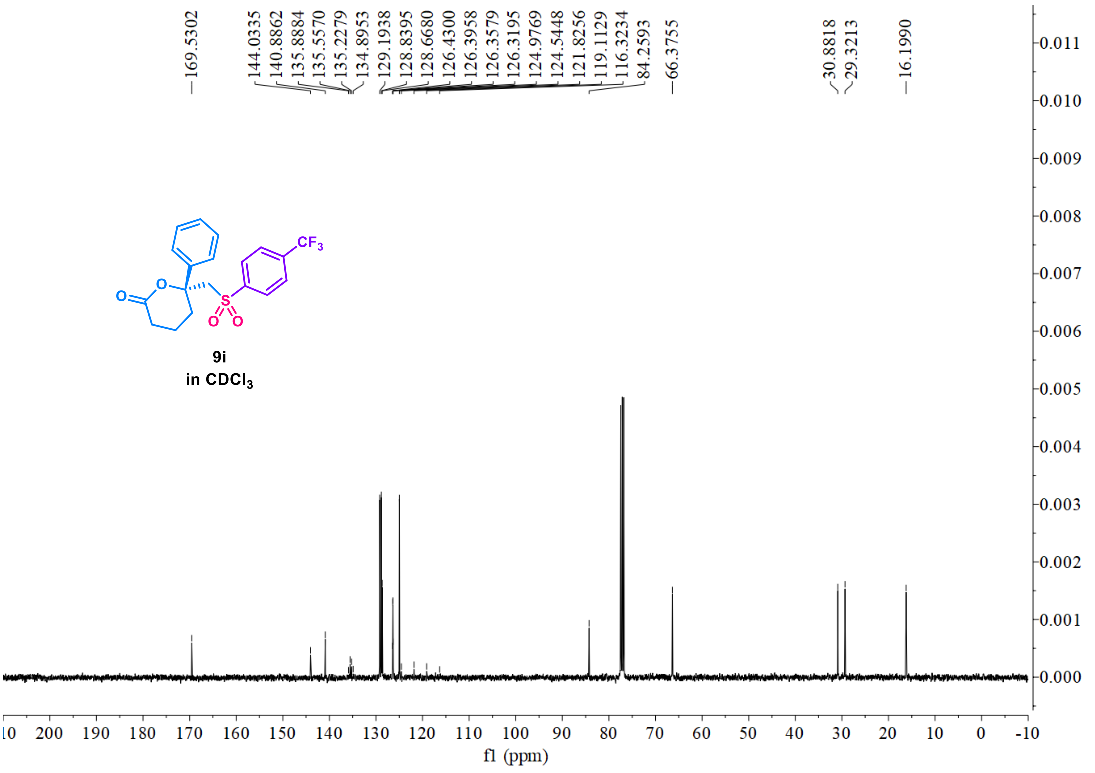
169.5302
144.0335
140.8862
135.8884
135.5570
135.2279
134.8953
129.1938
128.8395
128.6680
126.4300
126.3958
126.3579
126.3195
124.9769
124.5448
121.8256
119.1129
116.3234
84.2593
66.3755
30.8818
29.3213
16.1990
CF3
O
O
S
O O
9i
in CDCl3
f1 (ppm)

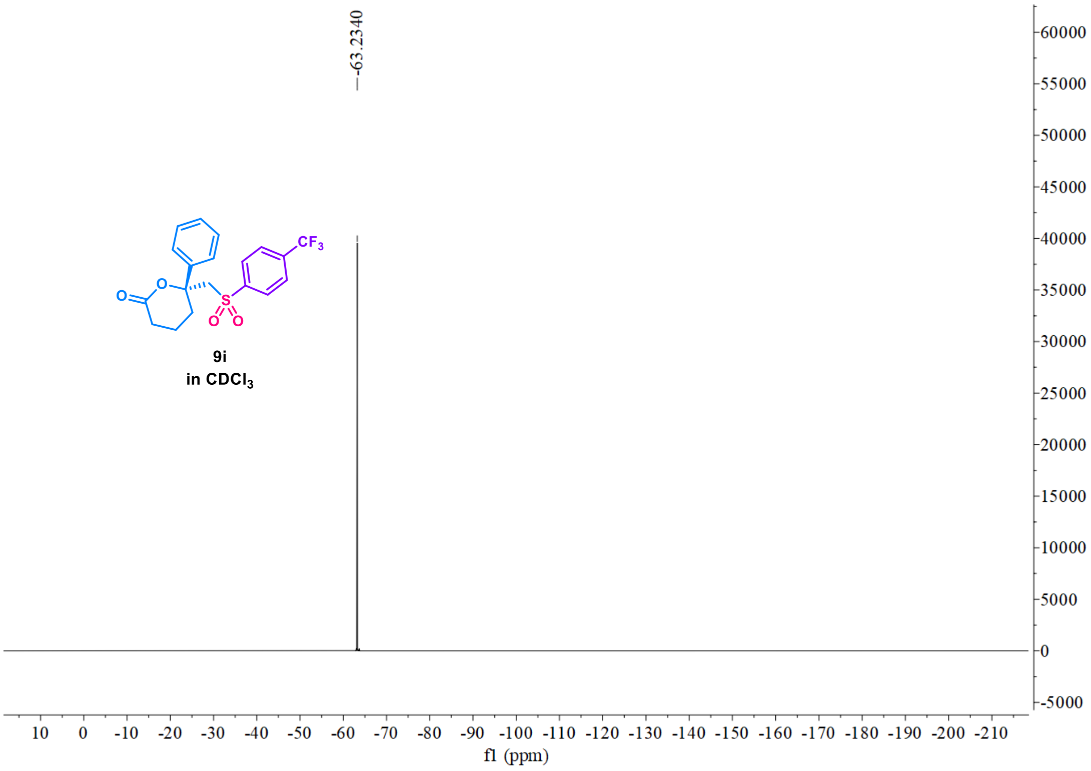

−63.2340
CF3
O
O
S
O
O
9i
in CDCl3
f1 (ppm)

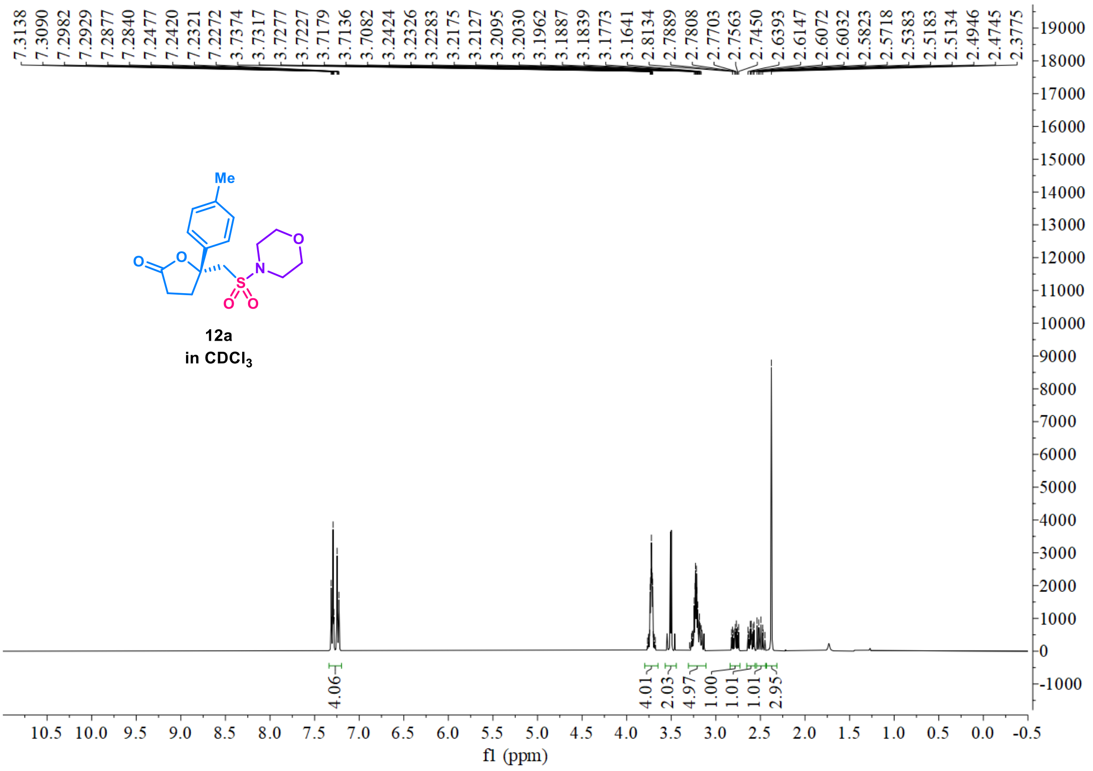

Me
O
O
N
O
S
O
O
12a
in CDCl3
4.06
4.01
2.03
4.97
1.00
1.01
1.01
2.95
f1 (ppm)

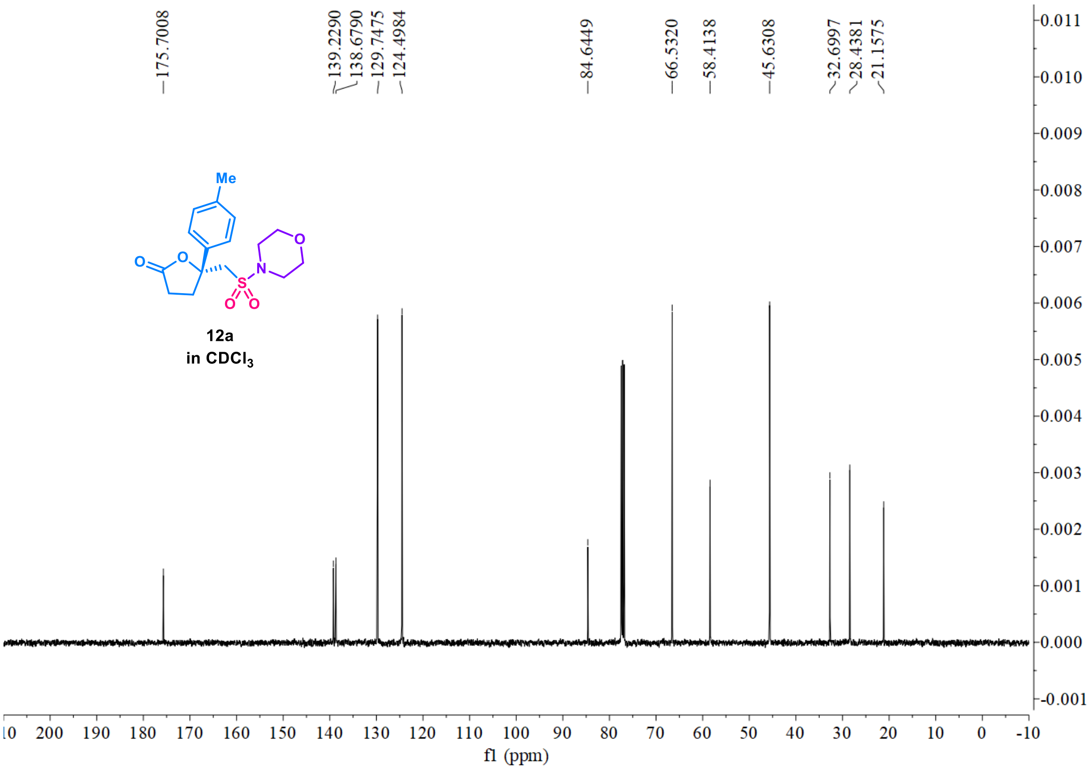
12a
in CDCl$_3$
f1 (ppm)

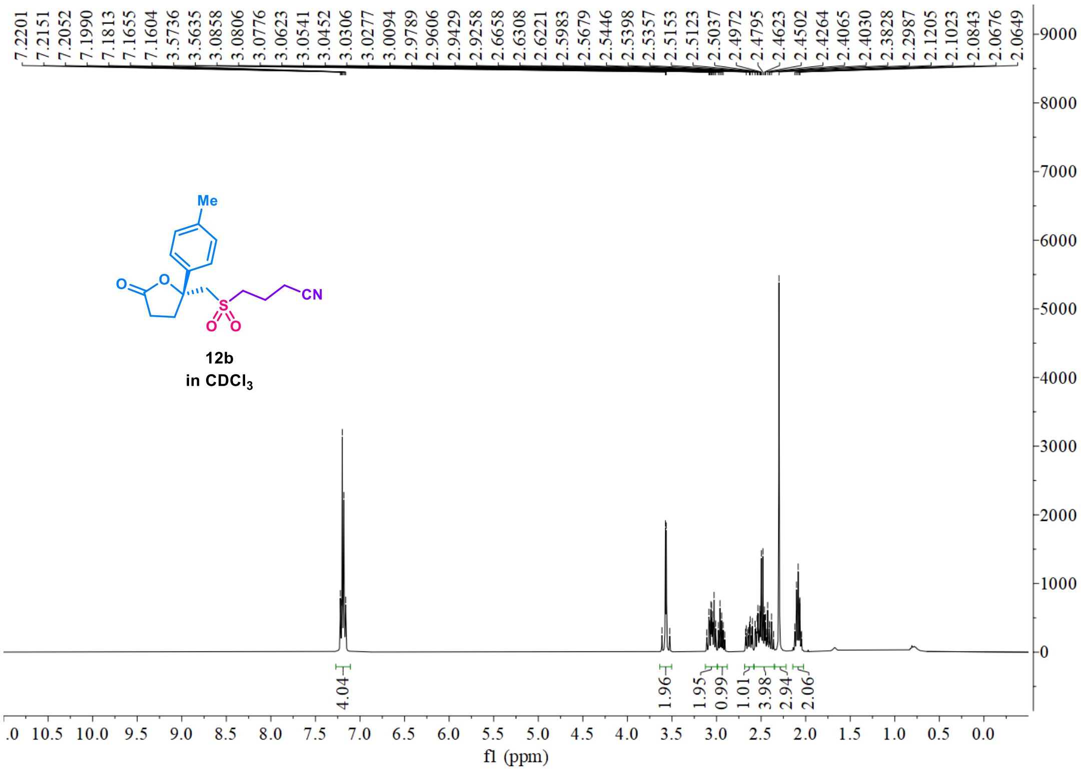
12b
in CDCl$_3$
f1 (ppm)

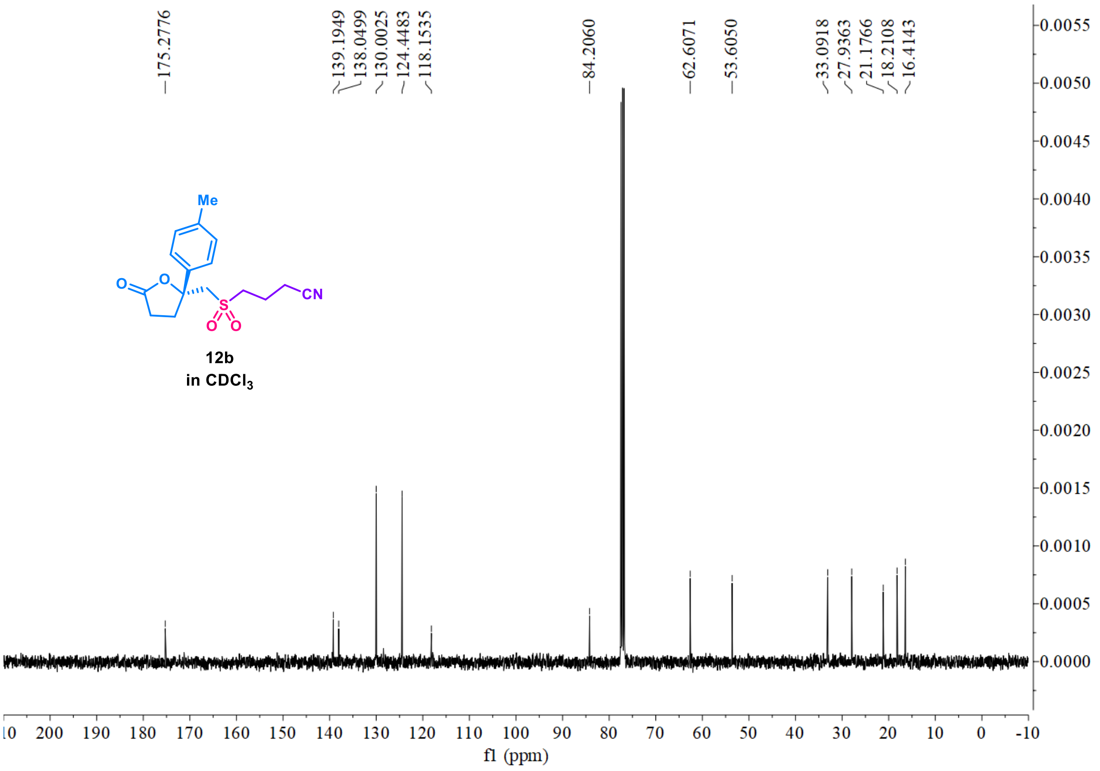

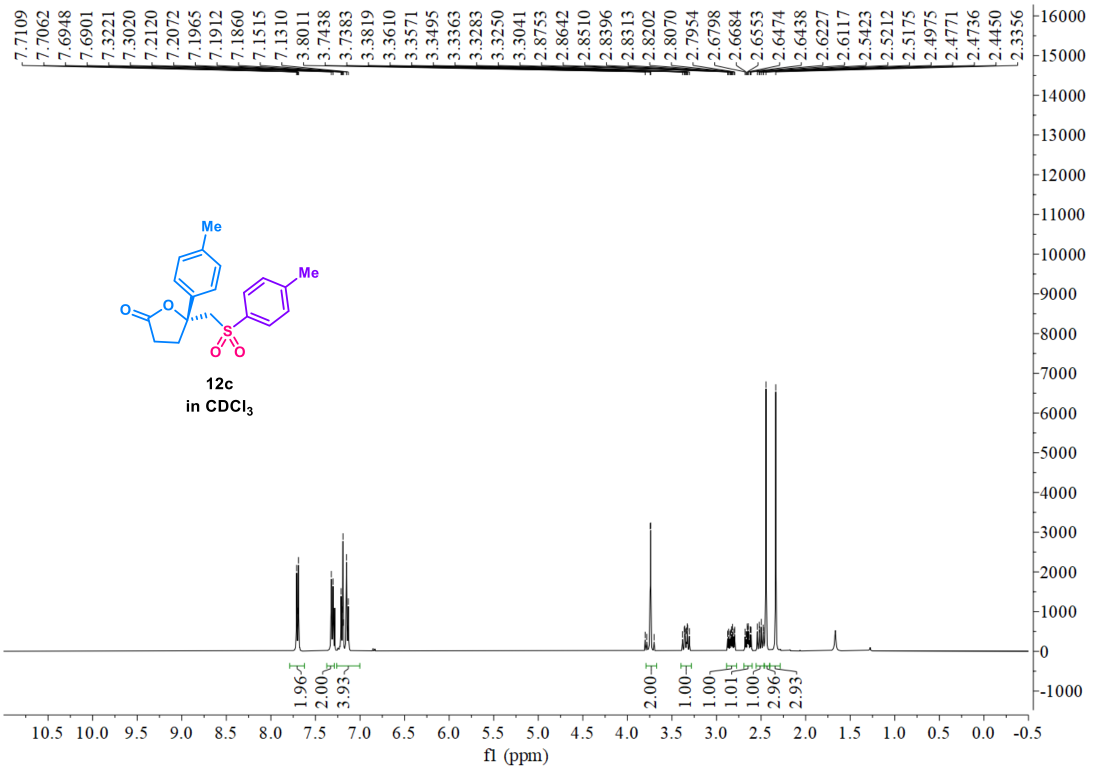

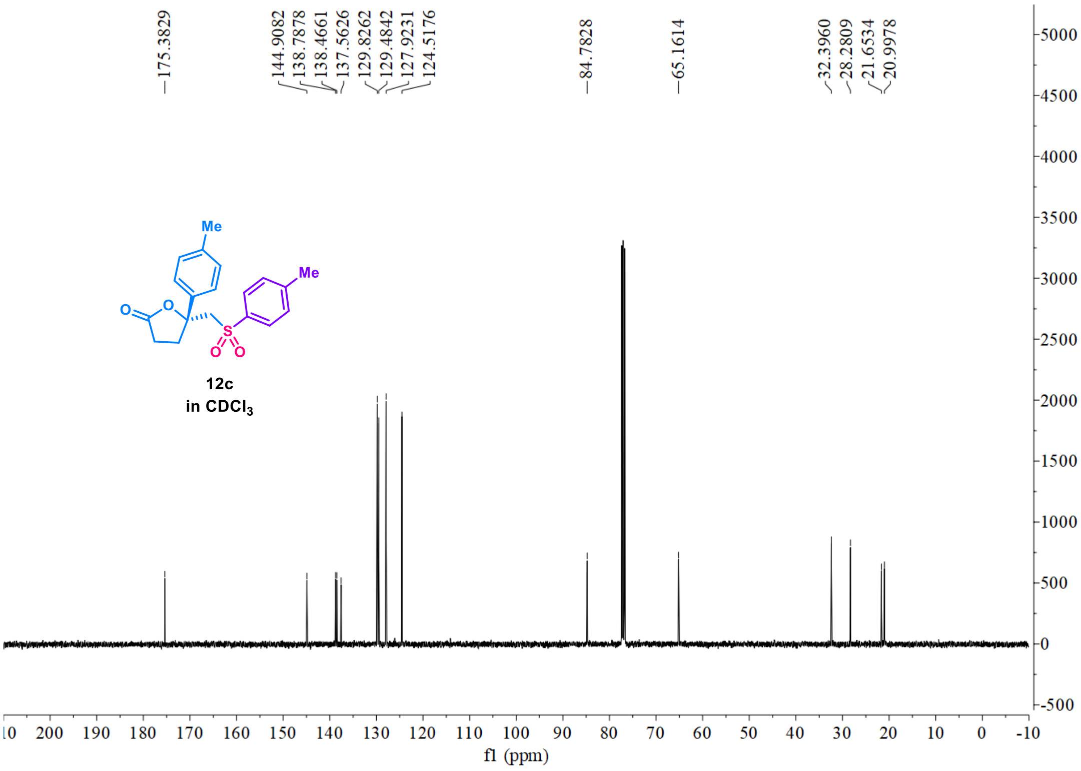
175.3829
144.9082
138.7878
138.4661
137.5626
129.8262
129.4842
127.9231
124.5176
84.7828
65.1614
32.3960
28.2809
21.6534
20.9978
Me
Me
O
O
S
O
O
12c
in CDCl3
f1 (ppm)

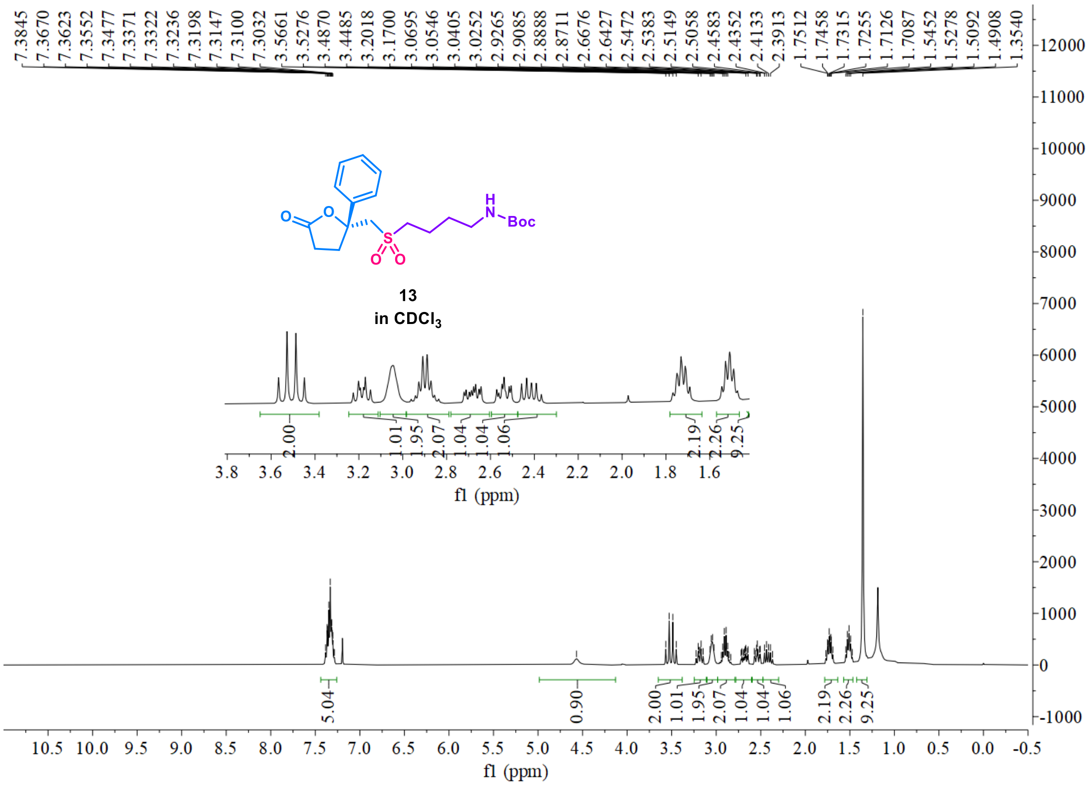
7.3845
7.3670
7.3623
7.3552
7.3477
7.3371
7.3322
7.3236
7.3198
7.3147
7.3100
7.3032
3.5661
3.5276
3.4870
3.4485
3.2018
3.1700
3.0695
3.0546
3.0405
3.0252
2.9265
2.9085
2.8888
2.8711
2.6676
2.6427
2.5472
2.5383
2.5149
2.5058
2.4583
2.4352
2.4133
2.3913
1.7512
1.7458
1.7315
1.7255
1.7126
1.7087
1.5452
1.5278
1.5092
1.4908
1.3540
H
N
Boc
13
in CDCl3
f1 (ppm)

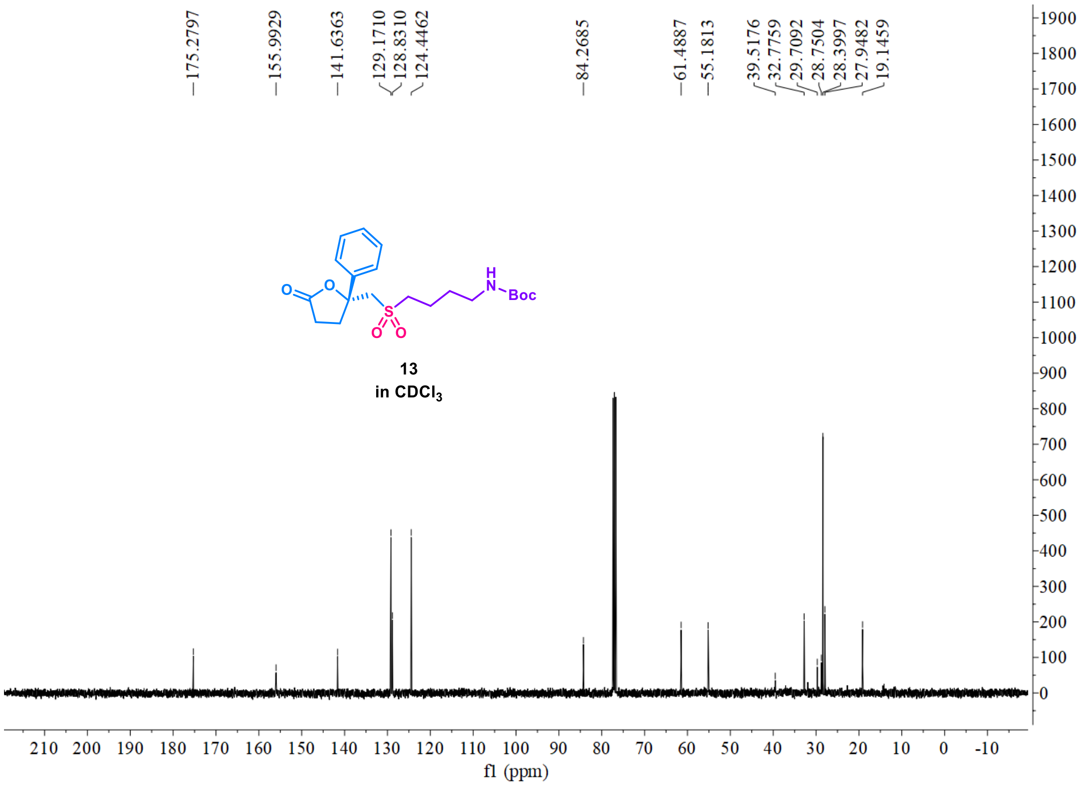

13
in $CDCl_3$

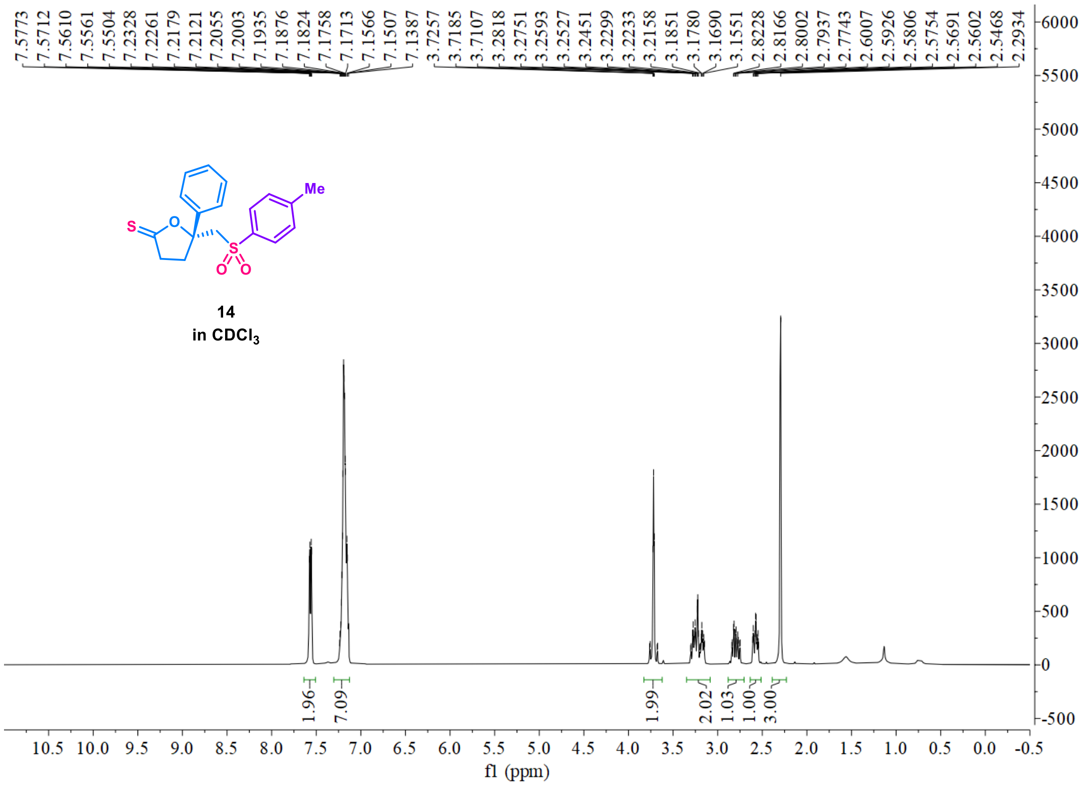

14
in $CDCl_3$

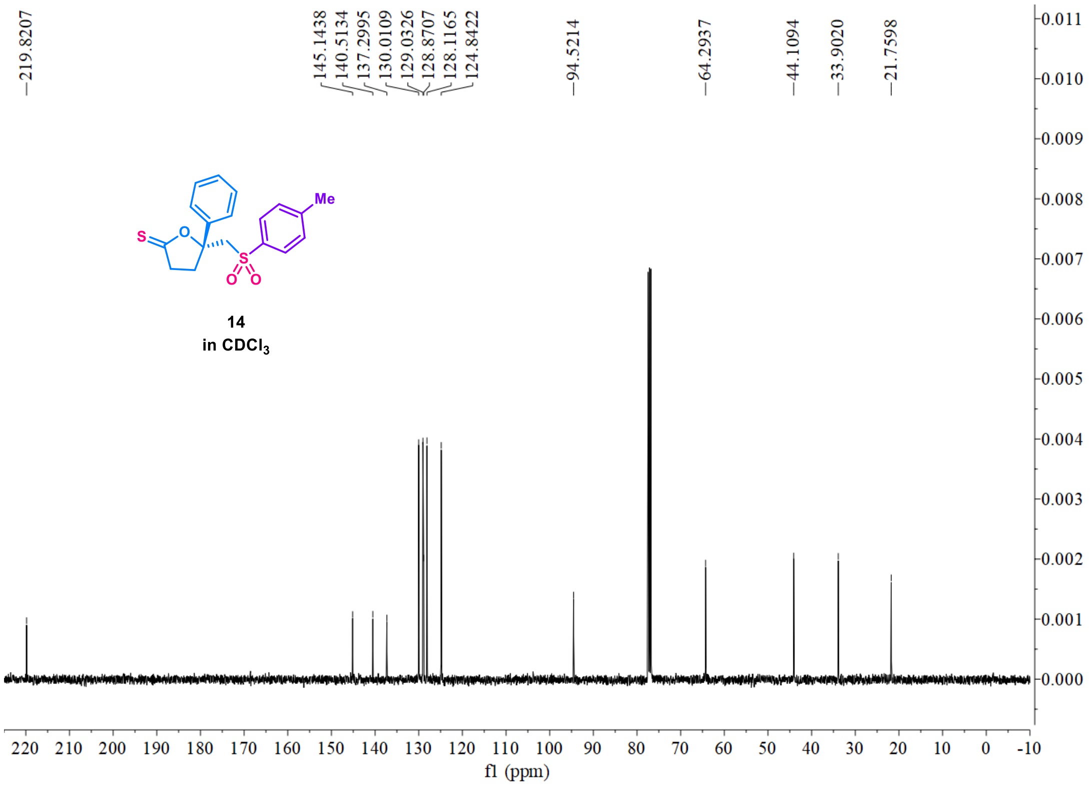

219.8207
145.1438
140.5134
137.2995
130.0109
129.0326
128.8707
128.1165
124.8422
94.5214
64.2937
44.1094
33.9020
21.7598
14
in $CDCl_3$
f1 (ppm)

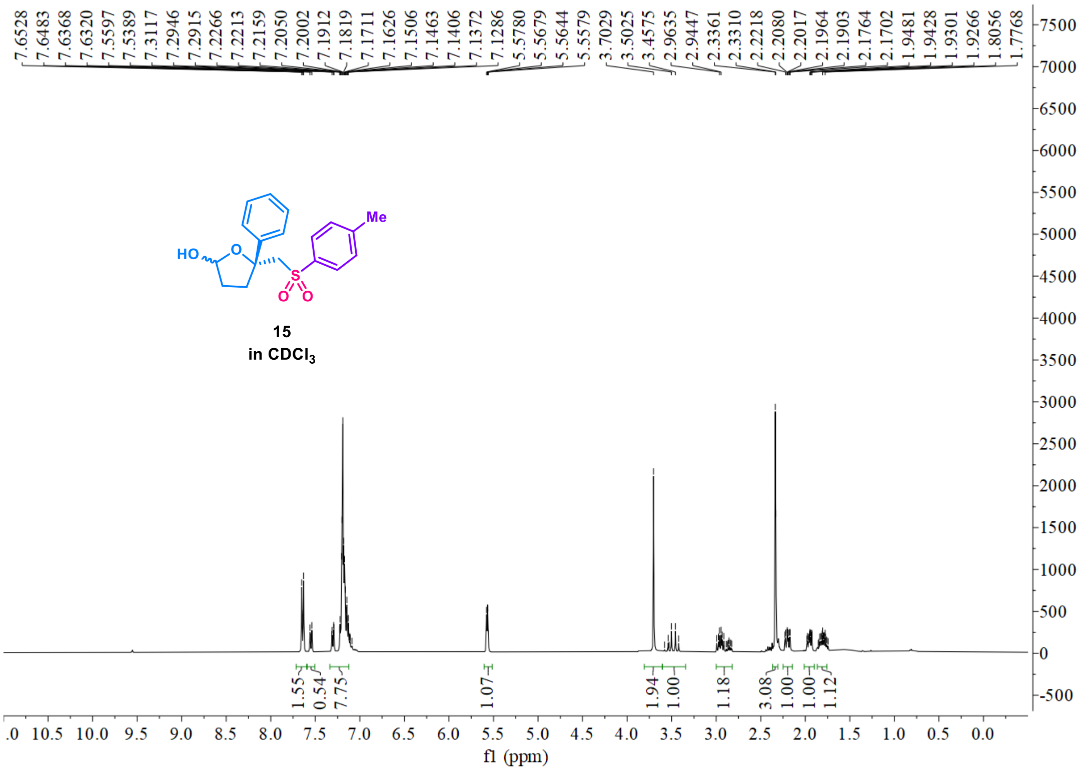

7.6528
7.6483
7.6368
7.6320
7.5597
7.5389
7.3117
7.2946
7.2915
7.2266
7.2213
7.2159
7.2050
7.2002
7.1912
7.1819
7.1711
7.1626
7.1506
7.1463
7.1406
7.1372
7.1286
5.5780
5.5679
5.5644
5.5579
3.7029
3.5025
3.4575
2.9635
2.9447
2.3361
2.3310
2.2218
2.2080
2.2017
2.1964
2.1903
2.1764
2.1702
1.9481
1.9428
1.9301
1.9266
1.8056
1.7768
15
in $CDCl_3$
1.55
0.54
7.75
1.07
1.94
1.00
1.18
3.08
1.00
1.00
1.12
f1 (ppm)

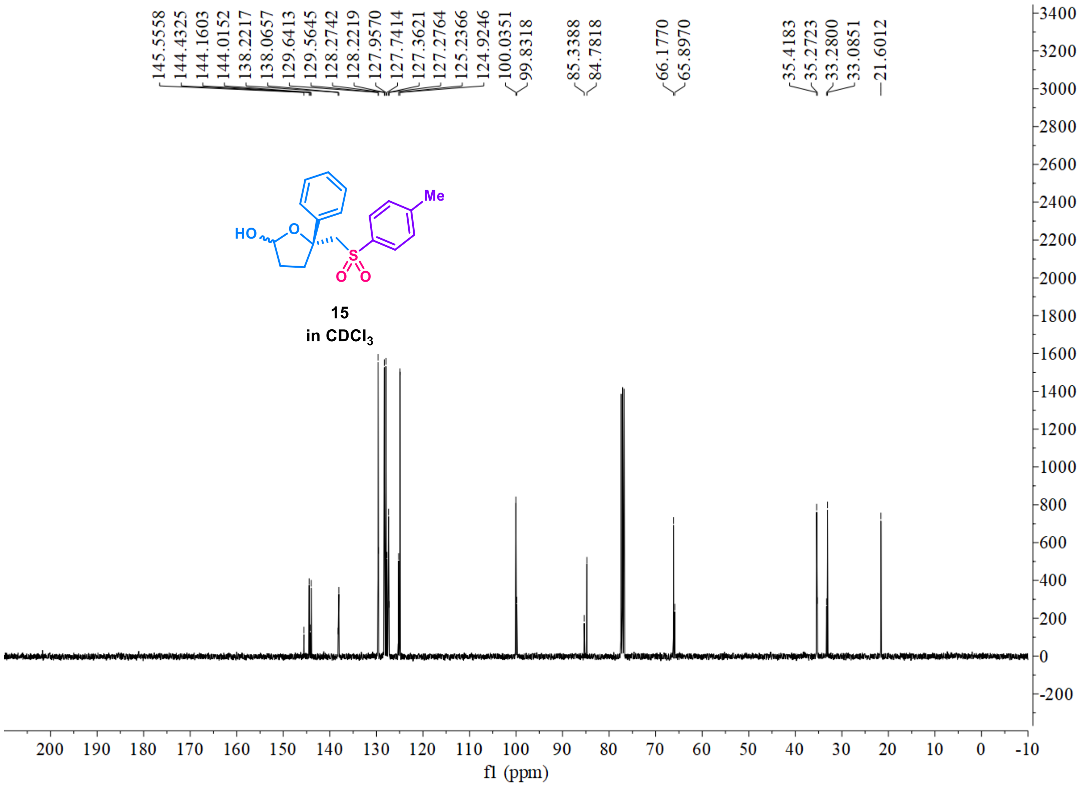
145.5558
144.4325
144.1603
144.0152
138.2217
138.0657
129.6413
129.5645
128.2742
128.2219
127.9570
127.7414
127.3621
127.2764
125.2366
124.9246
100.0351
99.8318
85.3388
84.7818
66.1770
65.8970
35.4183
35.2723
33.2800
33.0851
21.6012
HO
O
Me
S
O
O
15
in CDCl3
f1 (ppm)

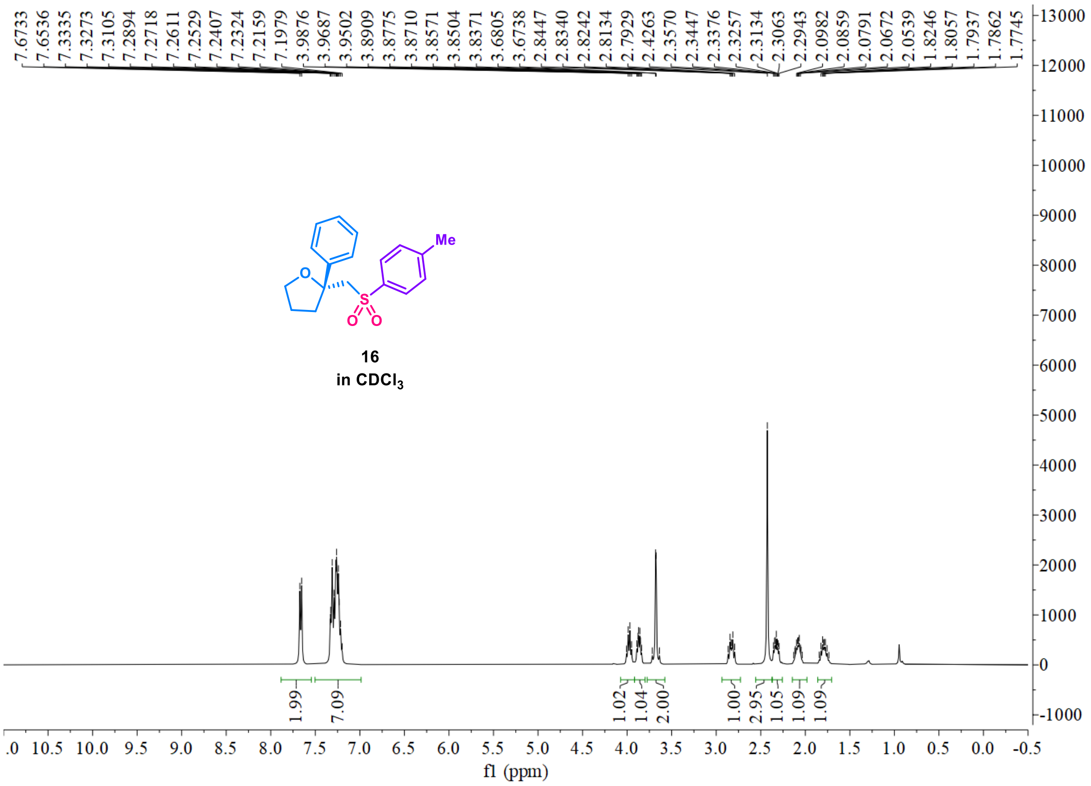
7.6733
7.6536
7.3335
7.3273
7.3105
7.2894
7.2718
7.2611
7.2529
7.2407
7.2324
7.2159
7.1979
3.9876
3.9687
3.9502
3.8909
3.8775
3.8710
3.8571
3.8504
3.8371
3.6805
3.6738
2.8447
2.8340
2.8242
2.8134
2.7929
2.4263
2.3570
2.3447
2.3376
2.3257
2.3134
2.3063
2.2943
2.0982
2.0859
2.0791
2.0672
2.0539
1.8246
1.8057
1.7937
1.7862
1.7745
O
Me
S
O
O
16
in CDCl3
1.99
7.09
1.02
1.04
2.00
1.00
2.95
1.05
1.09
1.09
f1 (ppm)

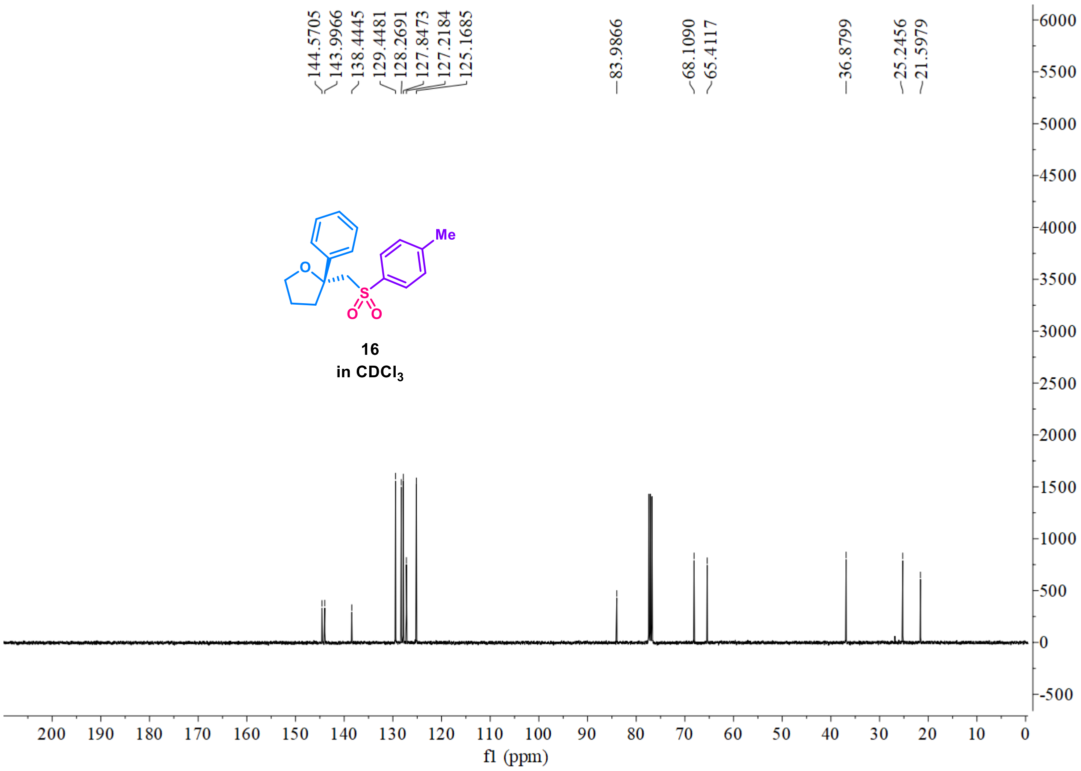
144.5705
143.9966
138.4445
129.4481
128.2691
127.8473
127.2184
125.1685
83.9866
68.1090
65.4117
36.8799
25.2456
21.5979
Me
O
S
O
O
16
in CDCl3
f1 (ppm)

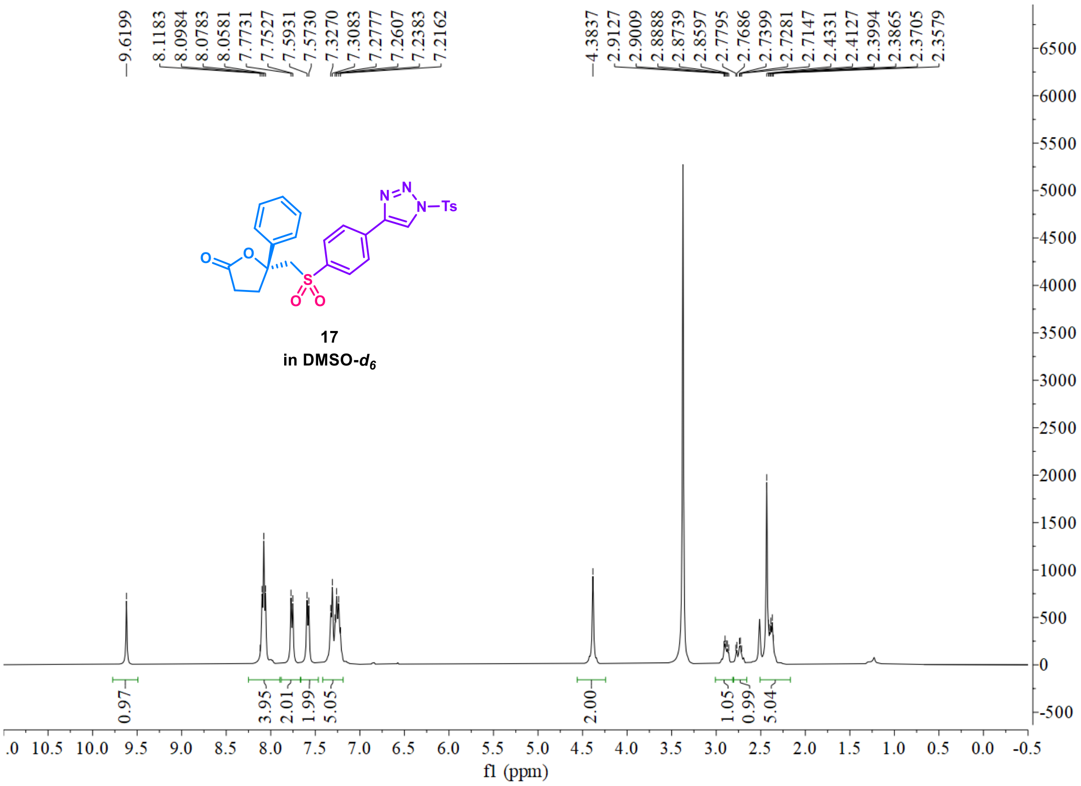
9.6199
8.1183
8.0984
8.0783
8.0581
7.7731
7.7527
7.5931
7.5730
7.3270
7.3083
7.2777
7.2607
7.2383
7.2162
4.3837
2.9127
2.9009
2.8888
2.8739
2.8597
2.7795
2.7686
2.7399
2.7281
2.7147
2.4331
2.4127
2.3994
2.3865
2.3705
2.3579
N
N
N–Ts
O
O
S
O
O
17
in DMSO-d6
0.97
3.95
2.01
1.99
5.05
2.00
1.05
0.99
5.04
f1 (ppm)

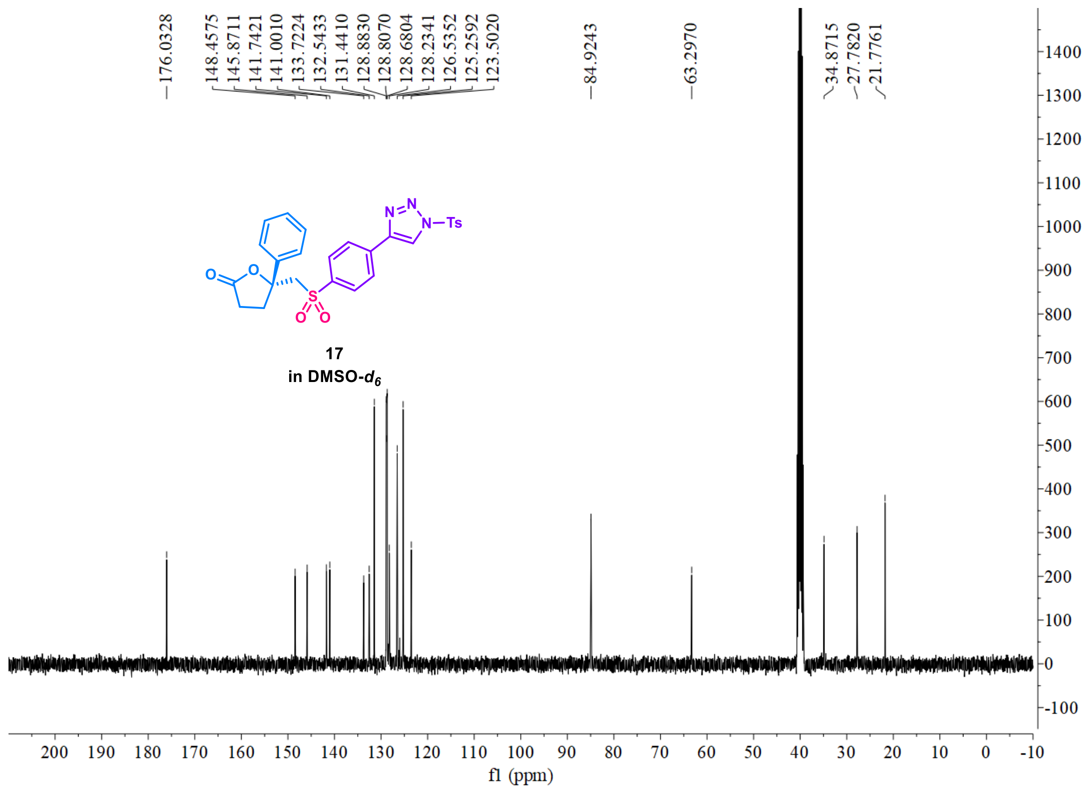

176.0328
148.4575
145.8711
141.7421
141.0010
133.7224
132.5433
131.4410
128.8830
128.8070
128.6804
128.2341
126.5352
125.2592
123.5020
84.9243
63.2970
34.8715
27.7820
21.7761
17
in DMSO-d_6
f1 (ppm)

(*R*)-5-((morpholinosulfonyl)methyl)-5-phenyldihydrofuran-2(3*H*)-one

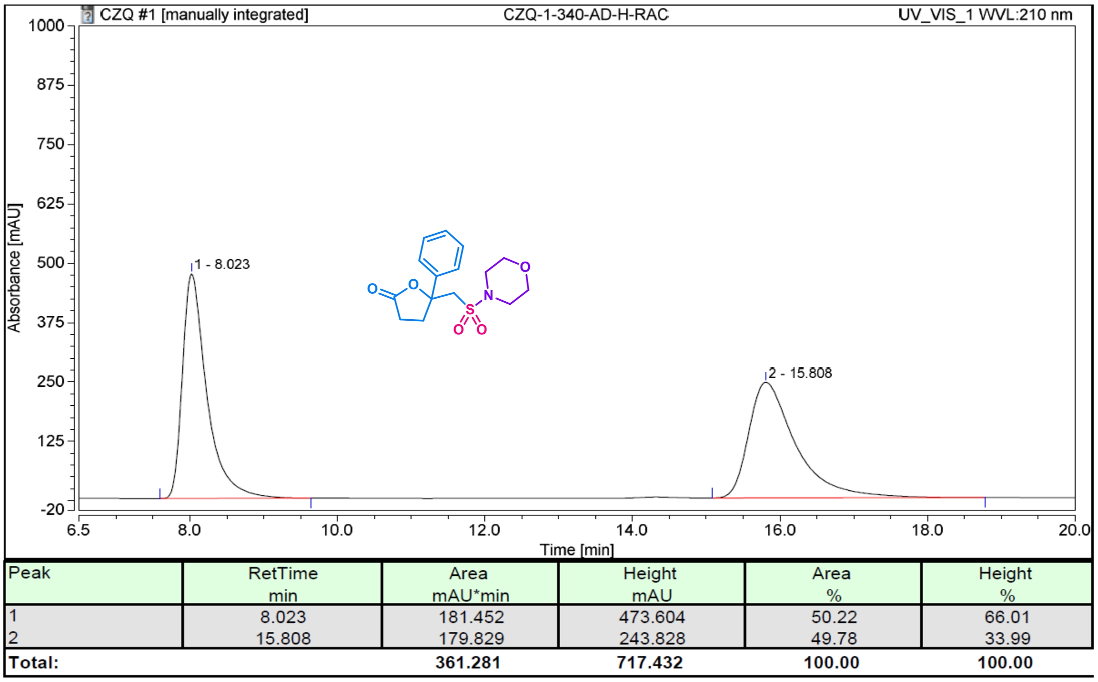

Peak	RetTime min	Area mAU*min	Height mAU	Area %	Height %
1	8.023	181.452	473.604	50.22	66.01
2	15.808	179.829	243.828	49.78	33.99
**Total:**		**361.281**	**717.432**	**100.00**	**100.00**

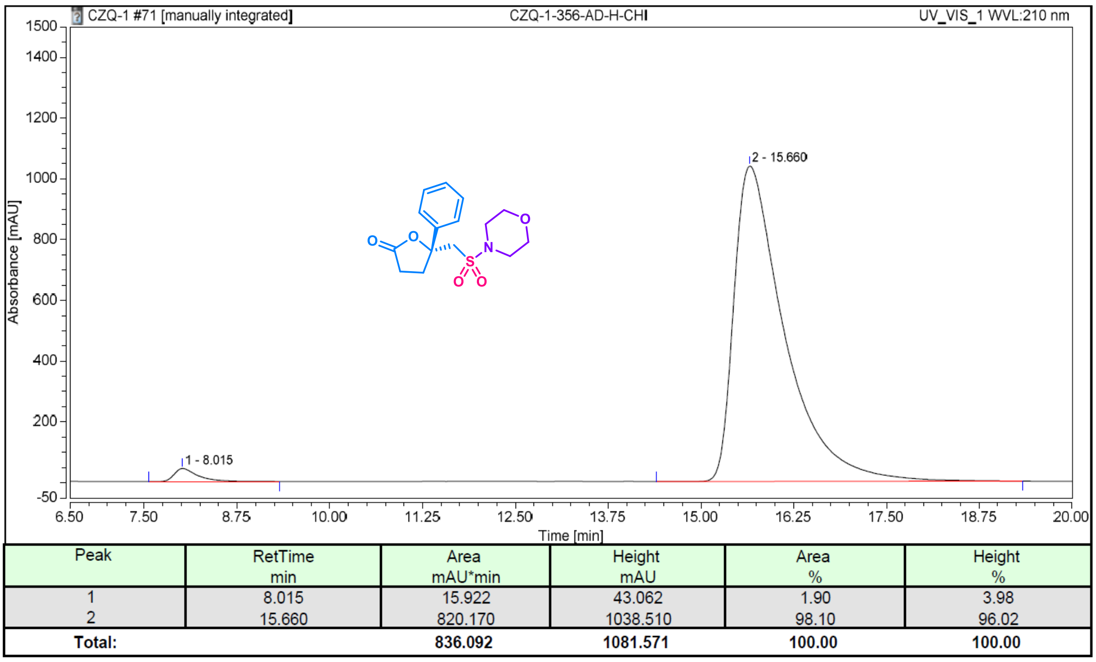

Peak	RetTime min	Area mAU*min	Height mAU	Area %	Height %
1	8.015	15.922	43.062	1.90	3.98
2	15.660	820.170	1038.510	98.10	96.02
**Total:**		**836.092**	**1081.571**	**100.00**	**100.00**

(*R*)-5-([1,1'-biphenyl]-4-yl)-5-((morpholinosulfonyl)methyl)dihydrofuran-2(3*H*)-one

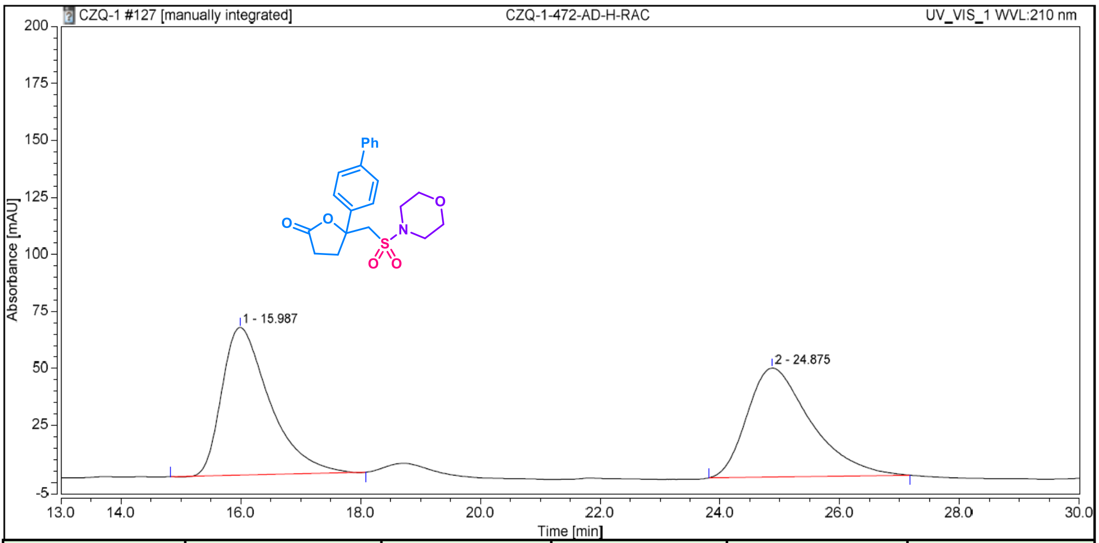

Peak	RetTime min	Area mAU*min	Height mAU	Area %	Height %
1	15.987	60.019	64.860	50.44	57.56
2	24.875	58.980	47.823	49.56	42.44
**Total:**		**118.998**	**112.683**	**100.00**	**100.00**

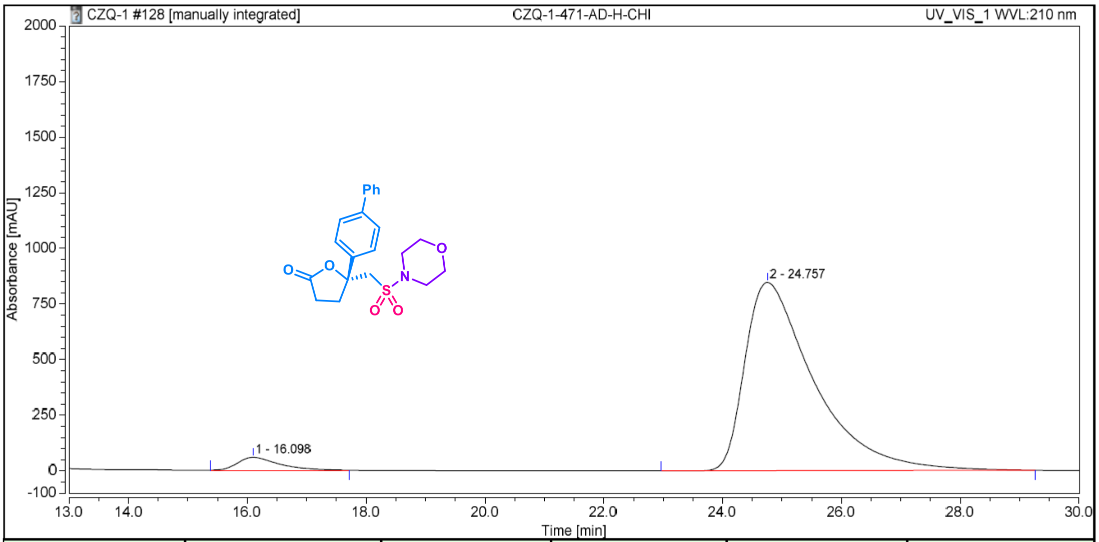

Peak	RetTime min	Area mAU*min	Height mAU	Area %	Height %
1	16.098	45.954	57.149	3.88	6.32
2	24.757	1138.346	846.392	96.12	93.68
**Total:**		**1184.300**	**903.541**	**100.00**	**100.00**

(*R*)-5-((morpholinosulfonyl)methyl)-5-(naphthalen-2-yl)dihydrofuran-2(3*H*)-one

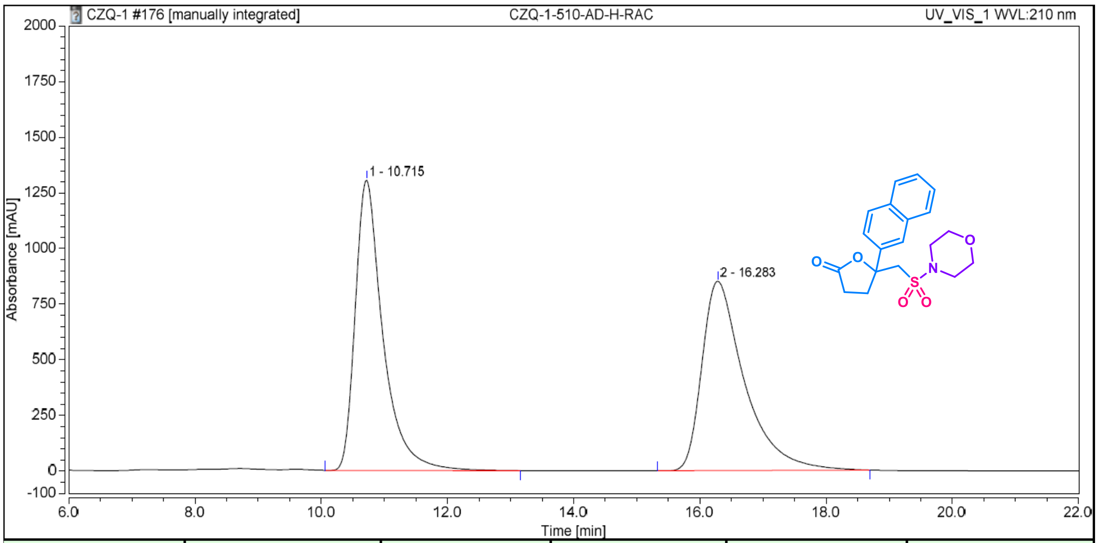

Peak	RetTime min	Area mAU*min	Height mAU	Area %	Height %
1	10.715	662.784	1305.039	49.92	60.53
2	16.283	664.951	851.074	50.08	39.47
**Total:**		**1327.735**	**2156.114**	**100.00**	**100.00**

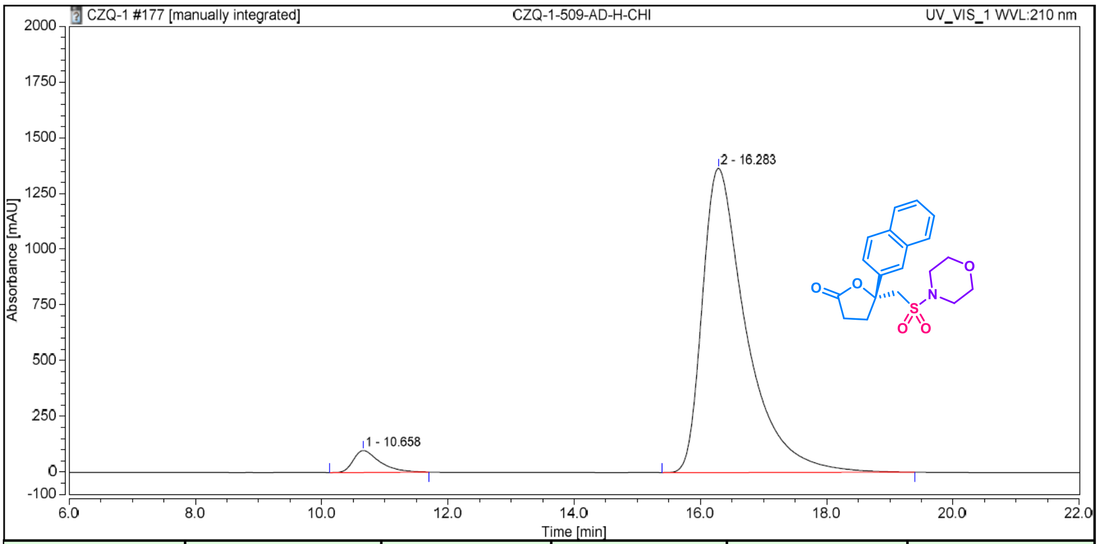

Peak	RetTime min	Area mAU*min	Height mAU	Area %	Height %
1	10.658	47.521	98.266	4.13	6.71
2	16.283	1102.080	1365.814	95.87	93.29
**Total:**		**1149.601**	**1464.080**	**100.00**	**100.00**

(*R*)-5-((morpholinosulfonyl)methyl)-5-(pyren-1-yl)dihydrofuran-2(3*H*)-one

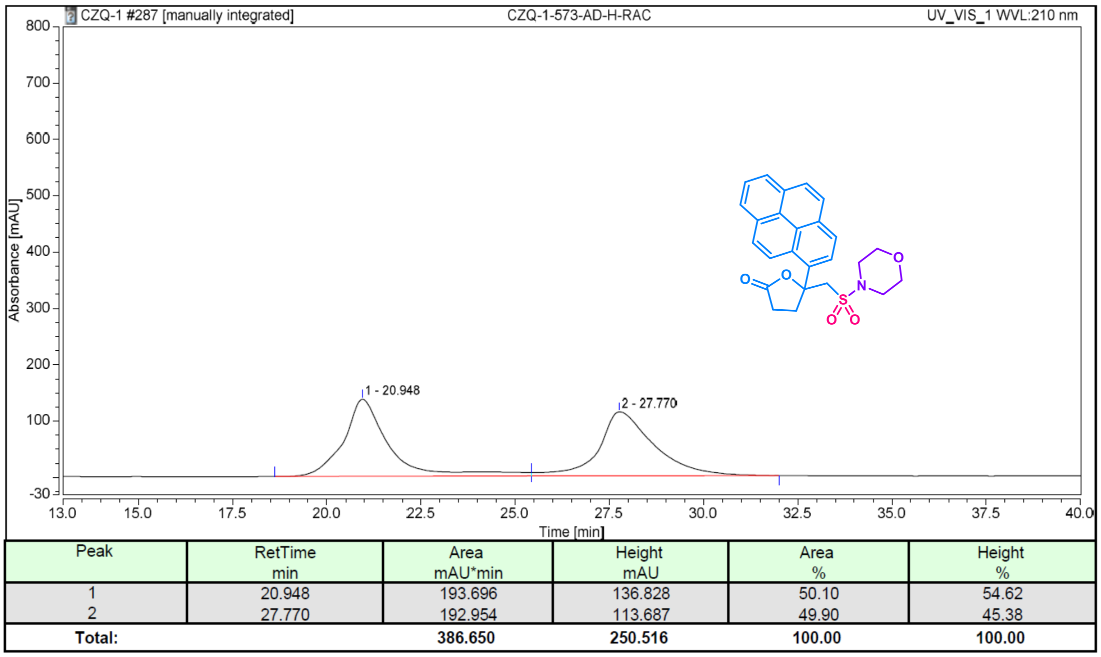

Peak	RetTime min	Area mAU*min	Height mAU	Area %	Height %
1	20.948	193.696	136.828	50.10	54.62
2	27.770	192.954	113.687	49.90	45.38
**Total:**		**386.650**	**250.516**	**100.00**	**100.00**

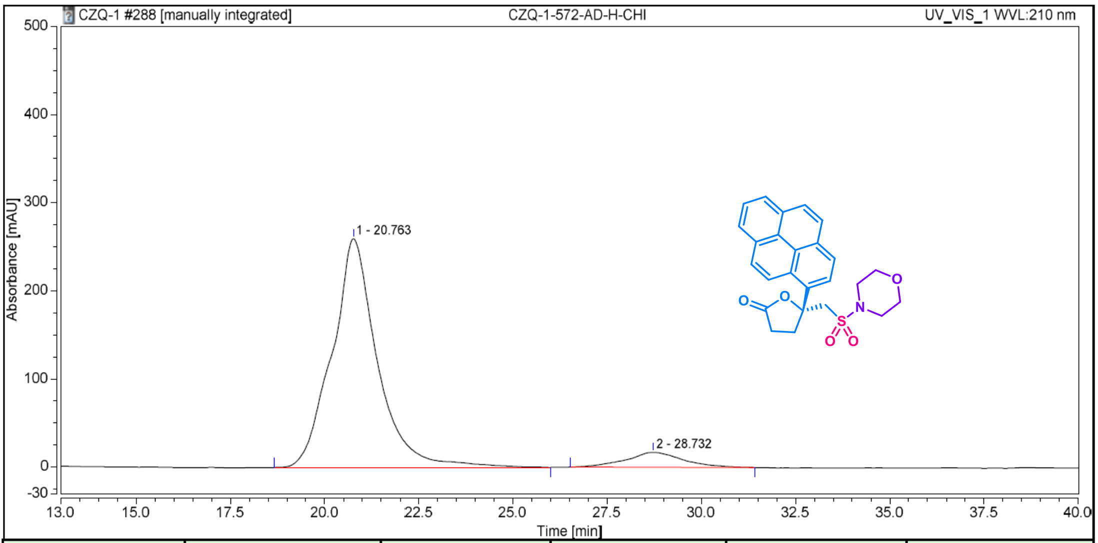

Peak	RetTime min	Area mAU*min	Height mAU	Area %	Height %
1	20.763	362.048	259.449	92.44	93.88
2	28.732	29.608	16.904	7.56	6.12
**Total:**		**391.656**	**276.353**	**100.00**	**100.00**

(*R*)-5-((morpholinosulfonyl)methyl)-5-(*m*-tolyl)dihydrofuran-2(3*H*)-one

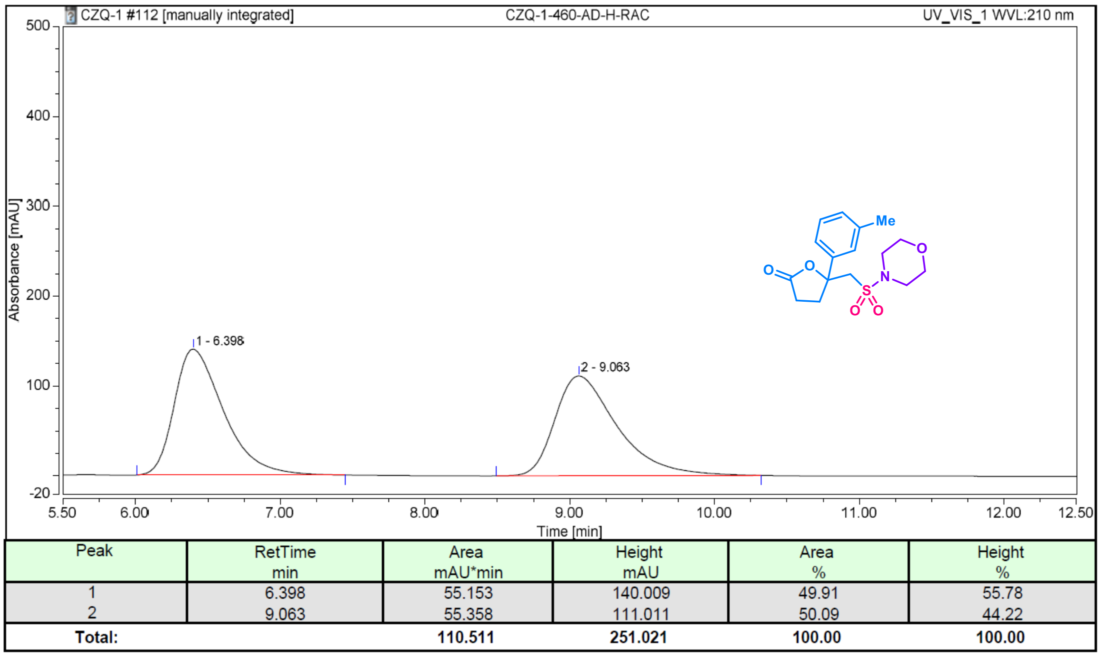

Peak	RetTime min	Area mAU*min	Height mAU	Area %	Height %
1	6.398	55.153	140.009	49.91	55.78
2	9.063	55.358	111.011	50.09	44.22
**Total:**		**110.511**	**251.021**	**100.00**	**100.00**

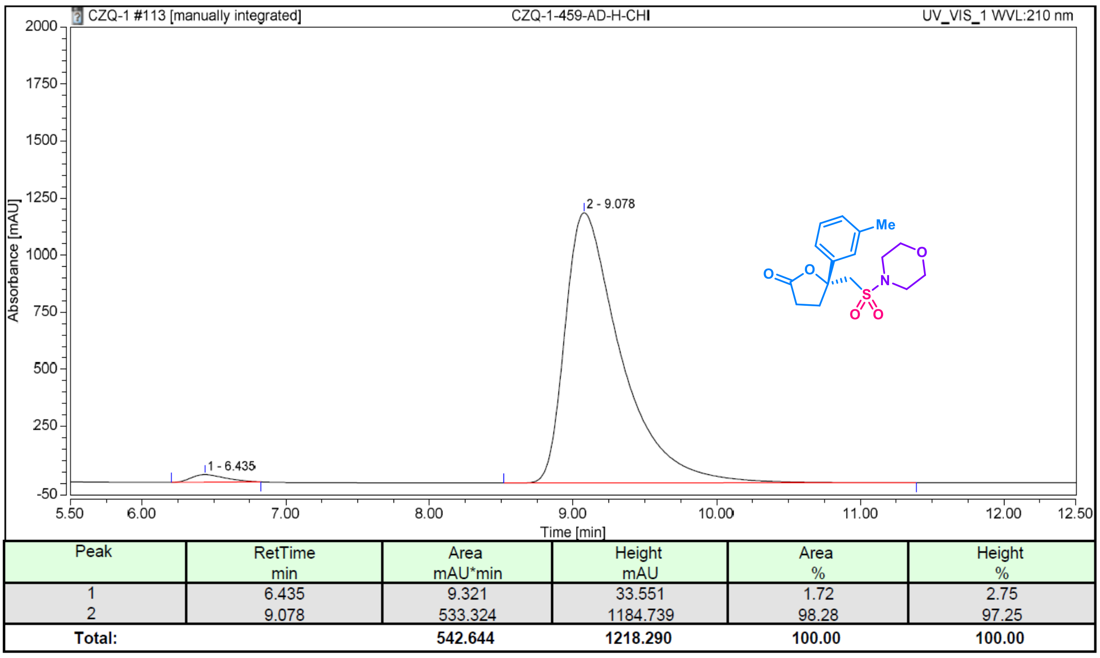

Peak	RetTime min	Area mAU*min	Height mAU	Area %	Height %
1	6.435	9.321	33.551	1.72	2.75
2	9.078	533.324	1184.739	98.28	97.25
**Total:**		**542.644**	**1218.290**	**100.00**	**100.00**

(*R*)-5-(4-(*tert*-butyl)phenyl)-5-((morpholinosulfonyl)methyl)dihydrofuran-2(3*H*)-one

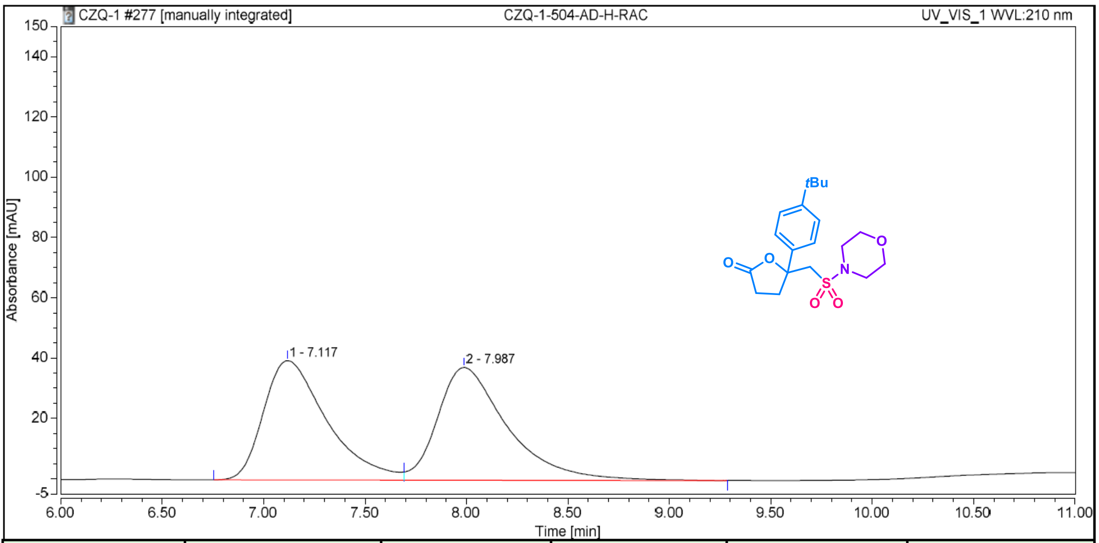

Peak	RetTime min	Area mAU*min	Height mAU	Area %	Height %
1	7.117	14.331	39.584	49.02	51.43
2	7.987	14.903	37.384	50.98	48.57
**Total:**		**29.233**	**76.968**	**100.00**	**100.00**

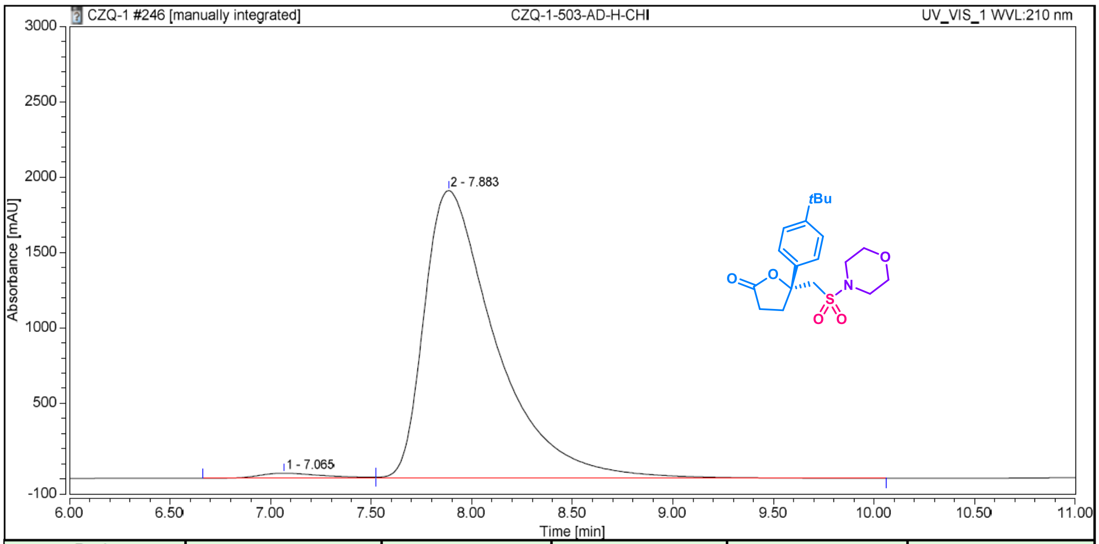

Peak	RetTime min	Area mAU*min	Height mAU	Area %	Height %
1	7.065	12.816	34.055	1.59	1.75
2	7.883	791.151	1907.911	98.41	98.25
**Total:**		**803.967**	**1941.966**	**100.00**	**100.00**

(*R*)-5-(3-methoxyphenyl)-5-((morpholinosulfonyl)methyl)dihydrofuran-2(3*H*)-one

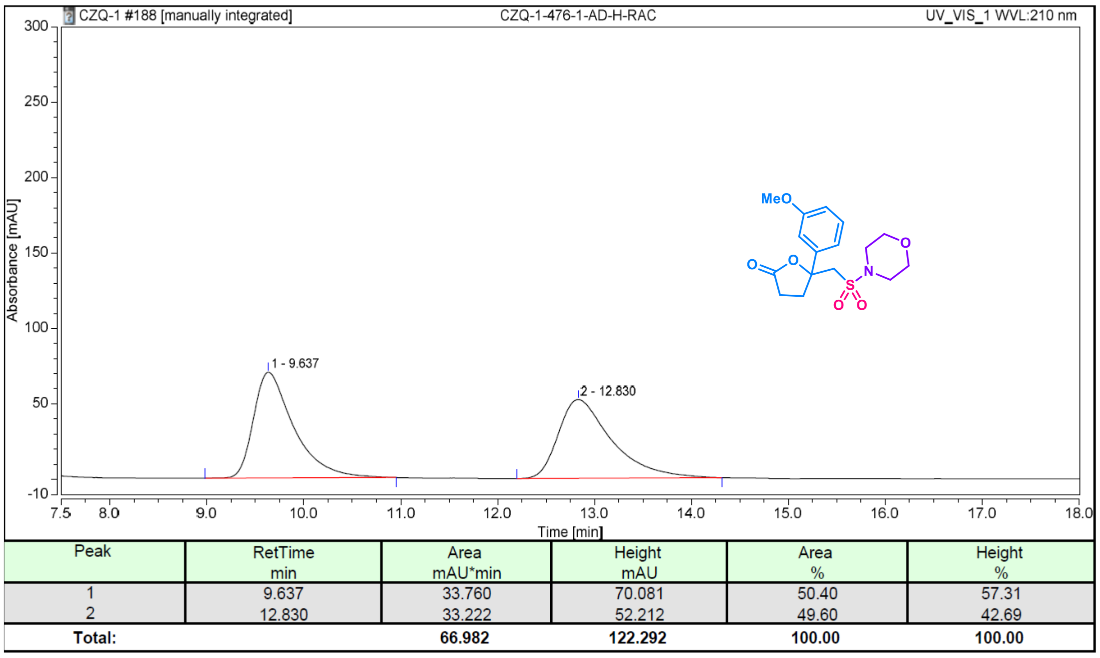

Peak	RetTime min	Area mAU*min	Height mAU	Area %	Height %
1	9.637	33.760	70.081	50.40	57.31
2	12.830	33.222	52.212	49.60	42.69
**Total:**		**66.982**	**122.292**	**100.00**	**100.00**

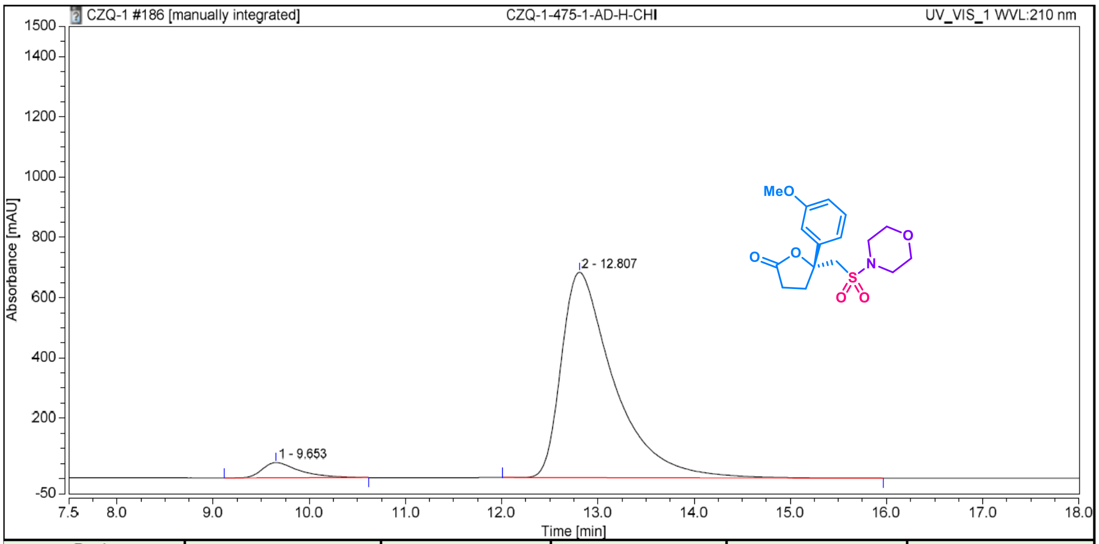

Peak	RetTime min	Area mAU*min	Height mAU	Area %	Height %
1	9.653	22.413	50.521	4.93	6.90
2	12.807	432.517	681.259	95.07	93.10
**Total:**		**454.930**	**731.779**	**100.00**	**100.00**

(*R*)-5-(3-fluorophenyl)-5-((morpholinosulfonyl)methyl)dihydrofuran-2(3*H*)-one

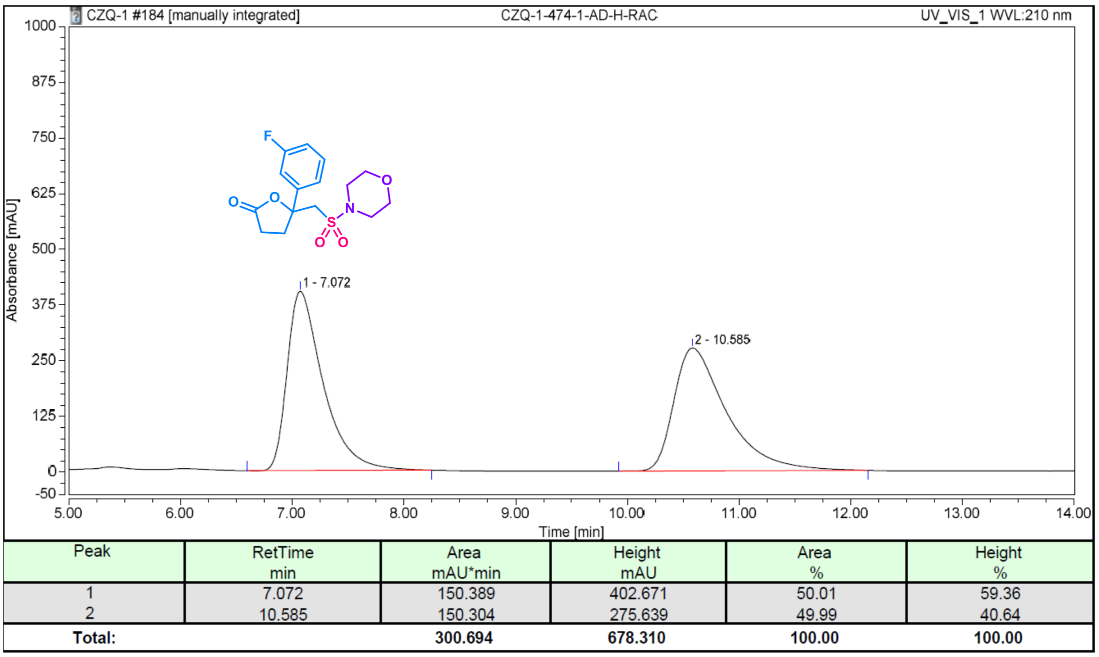

Peak	RetTime<br>min	Area<br>mAU*min	Height<br>mAU	Area<br>%	Height<br>%
1	7.072	150.389	402.671	50.01	59.36
2	10.585	150.304	275.639	49.99	40.64
**Total:**		**300.694**	**678.310**	**100.00**	**100.00**

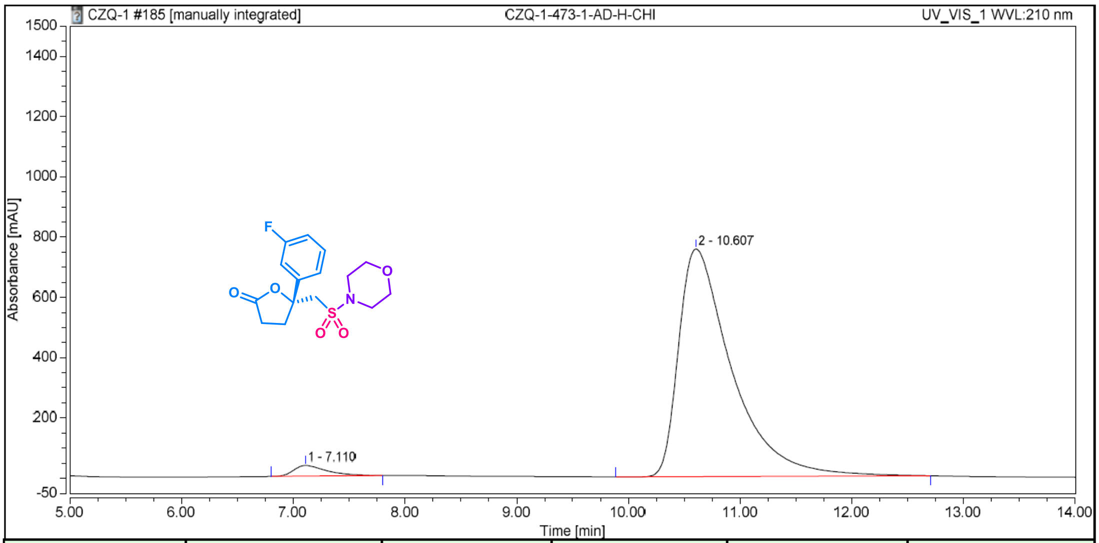

Peak	RetTime<br>min	Area<br>mAU*min	Height<br>mAU	Area<br>%	Height<br>%
1	7.110	12.266	35.363	2.87	4.47
2	10.607	415.543	755.282	97.13	95.53
**Total:**		**427.809**	**790.646**	**100.00**	**100.00**

(*R*)-5-(4-fluorophenyl)-5-((morpholinosulfonyl)methyl)dihydrofuran-2(3*H*)-one

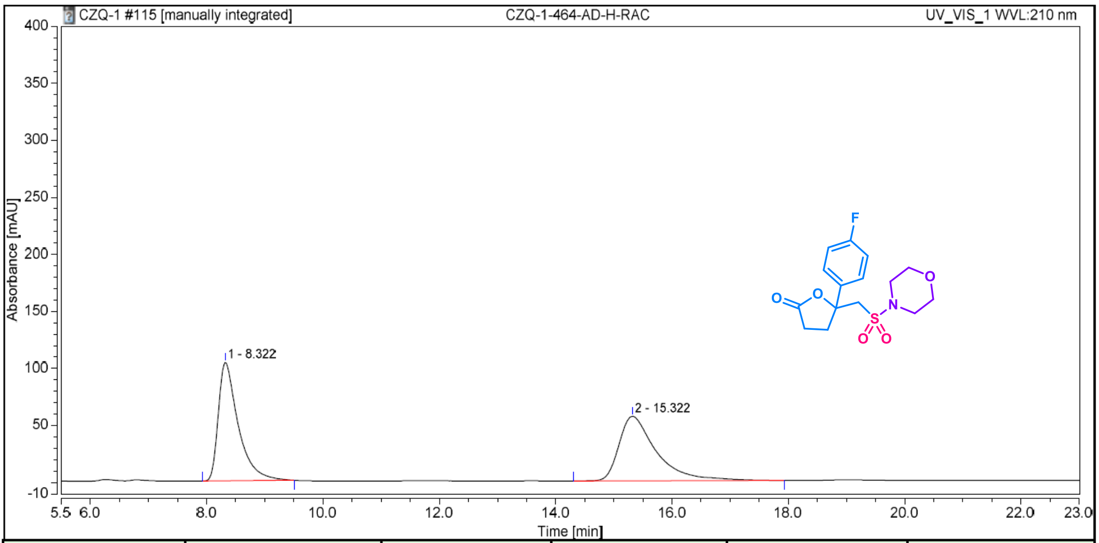

Peak	RetTime min	Area mAU*min	Height mAU	Area %	Height %
1	8.322	41.232	103.947	49.50	64.76
2	15.322	42.058	56.570	50.50	35.24
**Total:**		**83.291**	**160.517**	**100.00**	**100.00**

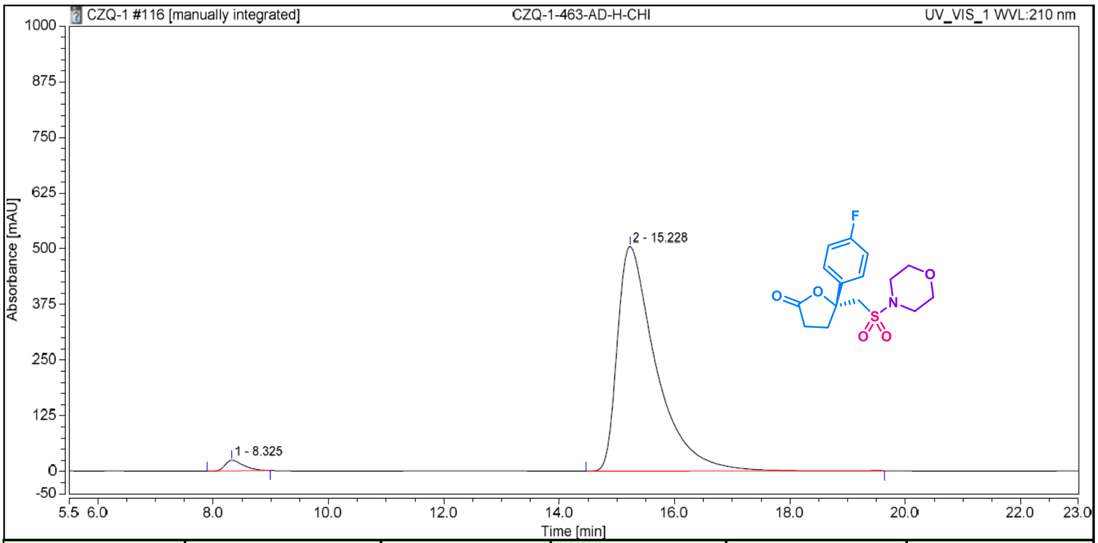

Peak	RetTime min	Area mAU*min	Height mAU	Area %	Height %
1	8.325	8.732	23.970	2.19	4.53
2	15.228	389.820	504.608	97.81	95.47
**Total:**		**398.552**	**528.578**	**100.00**	**100.00**

(*R*)-5-(4-iodophenyl)-5-((morpholinosulfonyl)methyl)dihydrofuran-2(3*H*)-one

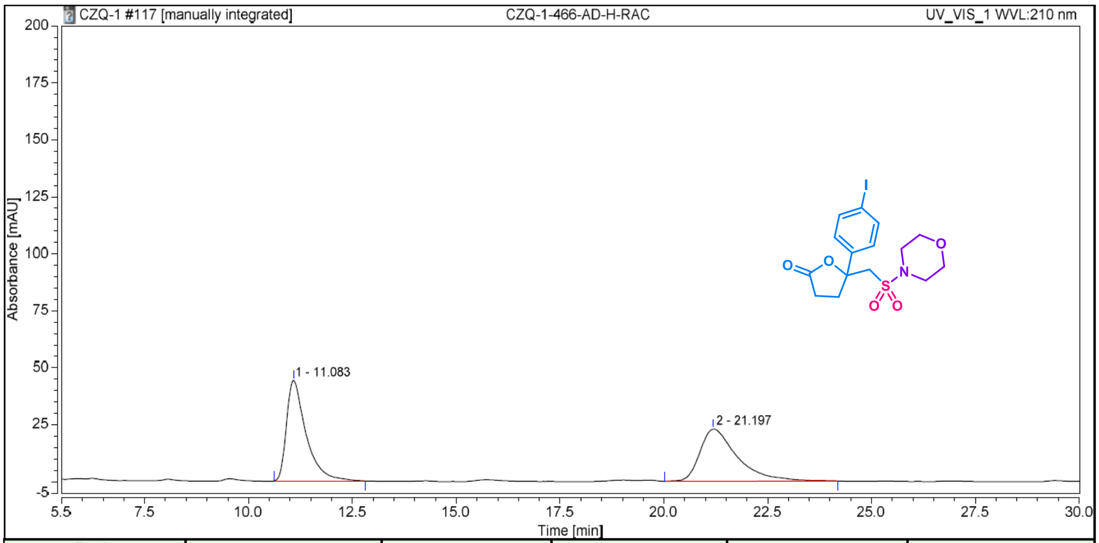

Peak	RetTime min	Area mAU*min	Height mAU	Area %	Height %
1	11.083	24.112	44.177	50.27	65.86
2	21.197	23.856	22.897	49.73	34.14
**Total:**		**47.968**	**67.074**	**100.00**	**100.00**

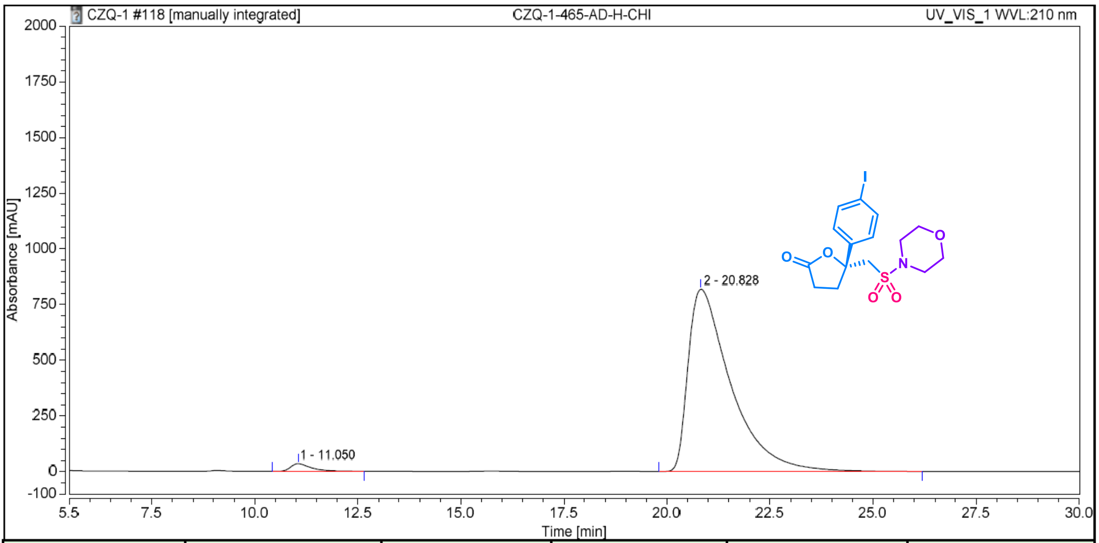

Peak	RetTime min	Area mAU*min	Height mAU	Area %	Height %
1	11.050	20.017	34.046	1.99	3.99
2	20.828	984.297	818.894	98.01	96.01
**Total:**		**1004.314**	**852.940**	**100.00**	**100.00**

(*R*)-5-((azepan-1-ylsulfonyl)methyl)-5-phenyldihydrofuran-2(3*H*)-one

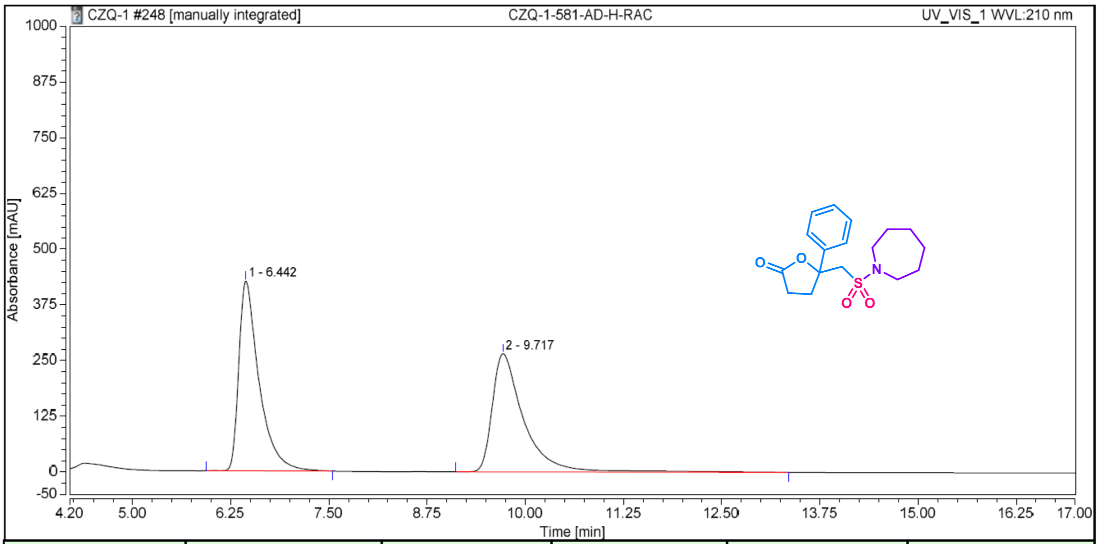

Peak	RetTime min	Area mAU*min	Height mAU	Area %	Height %
1	6.442	127.242	426.278	50.79	61.66
2	9.717	123.260	265.071	49.21	38.34
**Total:**		**250.502**	**691.349**	**100.00**	**100.00**

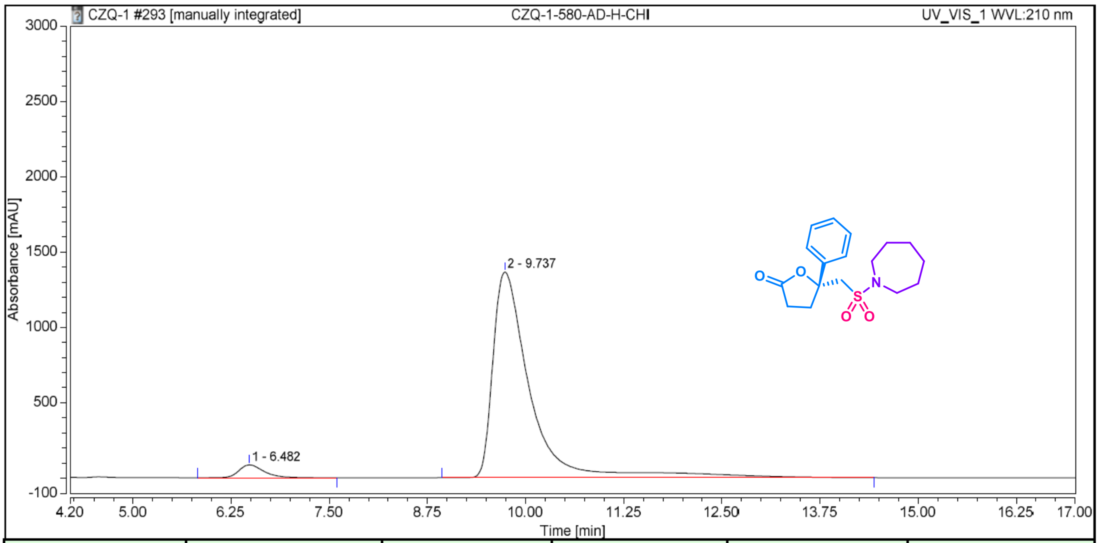

Peak	RetTime min	Area mAU*min	Height mAU	Area %	Height %
1	6.482	32.031	86.006	4.06	5.92
2	9.737	757.108	1365.811	95.94	94.08
**Total:**		**789.139**	**1451.817**	**100.00**	**100.00**

(*R*)-*N*,*N*-dibenzyl-1-(5-oxo-2-phenyltetrahydrofuran-2-yl)methanesulfonamide

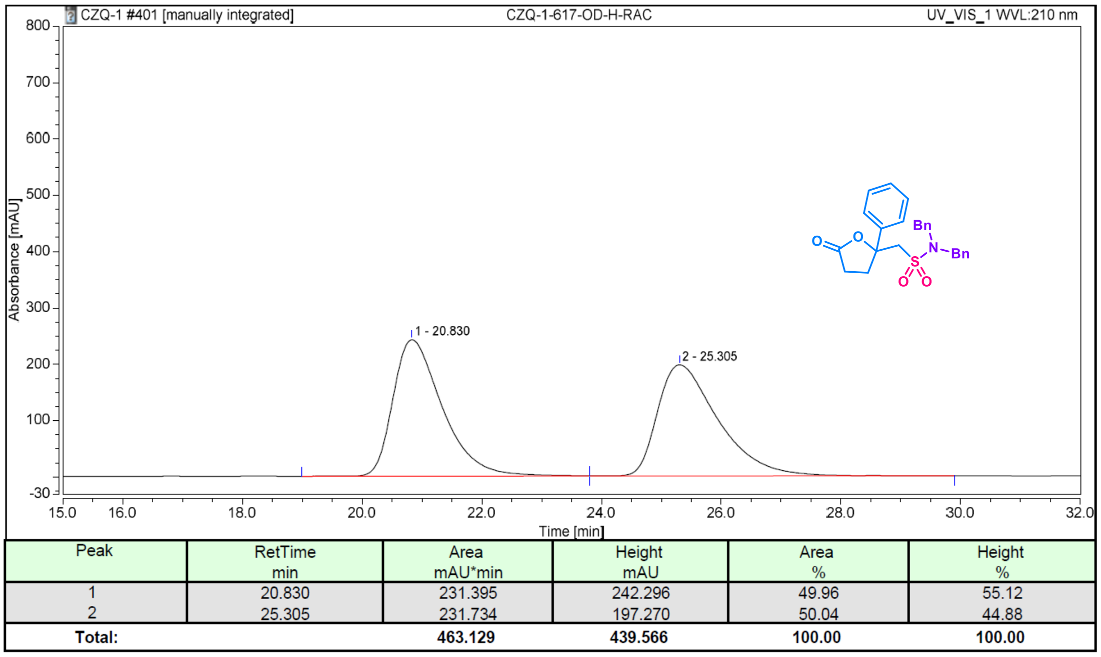

Peak	RetTime min	Area mAU*min	Height mAU	Area %	Height %
1	20.830	231.395	242.296	49.96	55.12
2	25.305	231.734	197.270	50.04	44.88
**Total:**		**463.129**	**439.566**	**100.00**	**100.00**

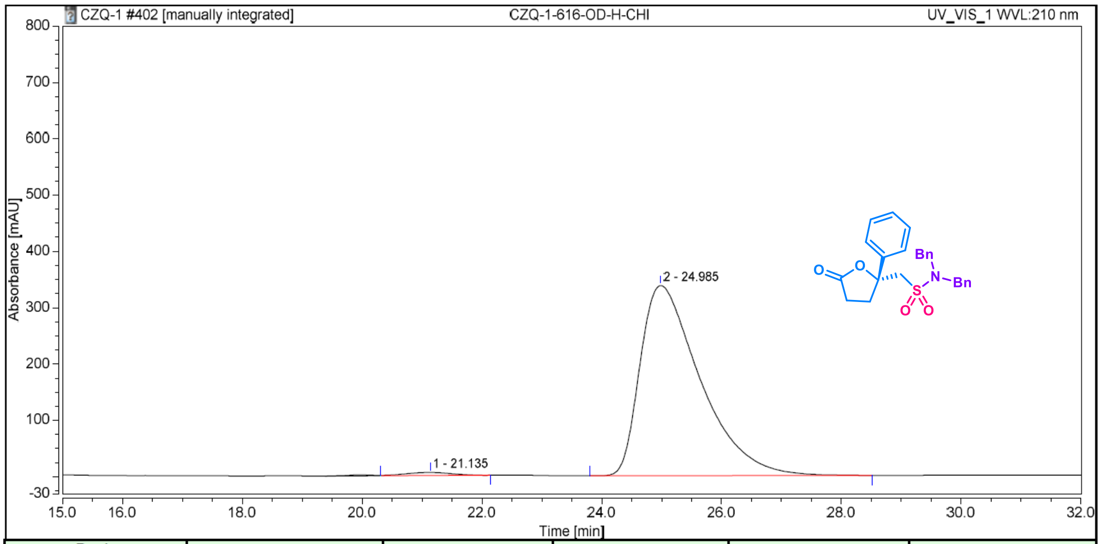

Peak	RetTime min	Area mAU*min	Height mAU	Area %	Height %
1	21.135	4.849	5.466	1.22	1.59
2	24.985	392.123	337.667	98.78	98.41
**Total:**		**396.973**	**343.132**	**100.00**	**100.00**

(*R*)-*N*,*N*-diethyl-1-(5-oxo-2-phenyltetrahydrofuran-2-yl)methanesulfonamide

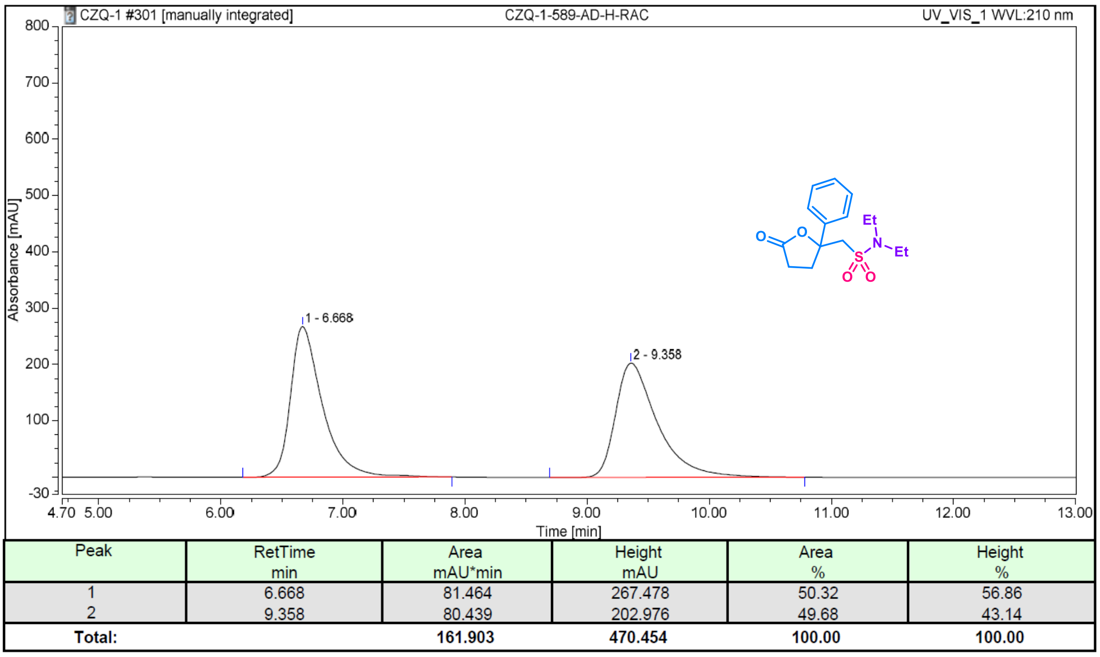

Peak	RetTime min	Area mAU*min	Height mAU	Area %	Height %
1	6.668	81.464	267.478	50.32	56.86
2	9.358	80.439	202.976	49.68	43.14
**Total:**		**161.903**	**470.454**	**100.00**	**100.00**

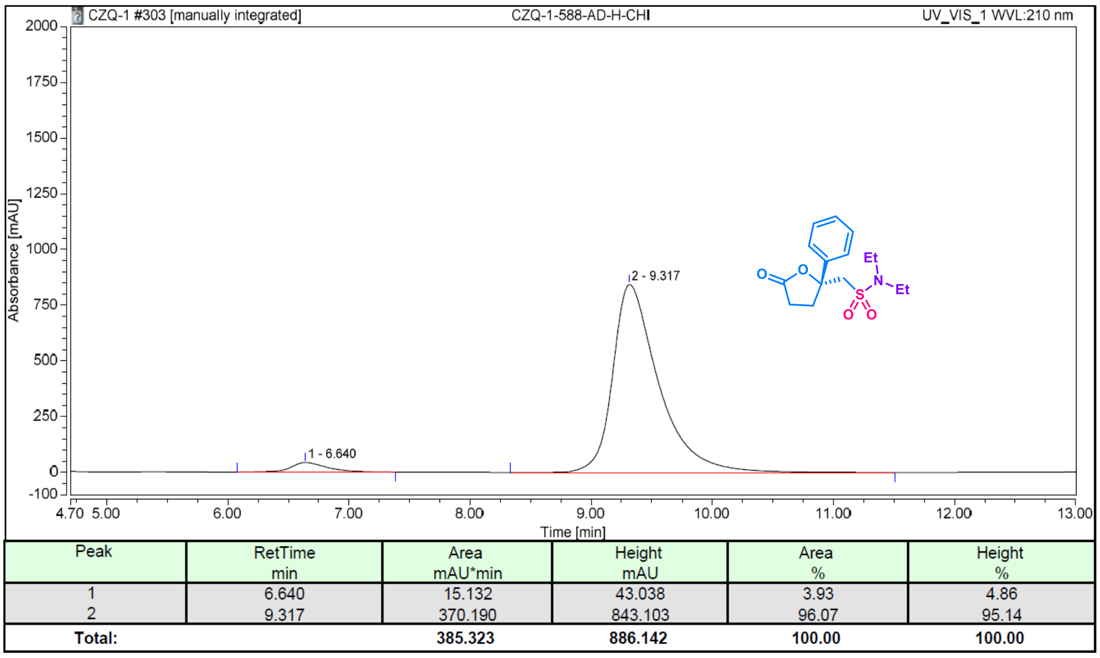

Peak	RetTime min	Area mAU*min	Height mAU	Area %	Height %
1	6.640	15.132	43.038	3.93	4.86
2	9.317	370.190	843.103	96.07	95.14
**Total:**		**385.323**	**886.142**	**100.00**	**100.00**

(*R*)-*N*-benzyl-*N*-ethyl-1-(5-oxo-2-phenyltetrahydrofuran-2-yl)methanesulfonamide

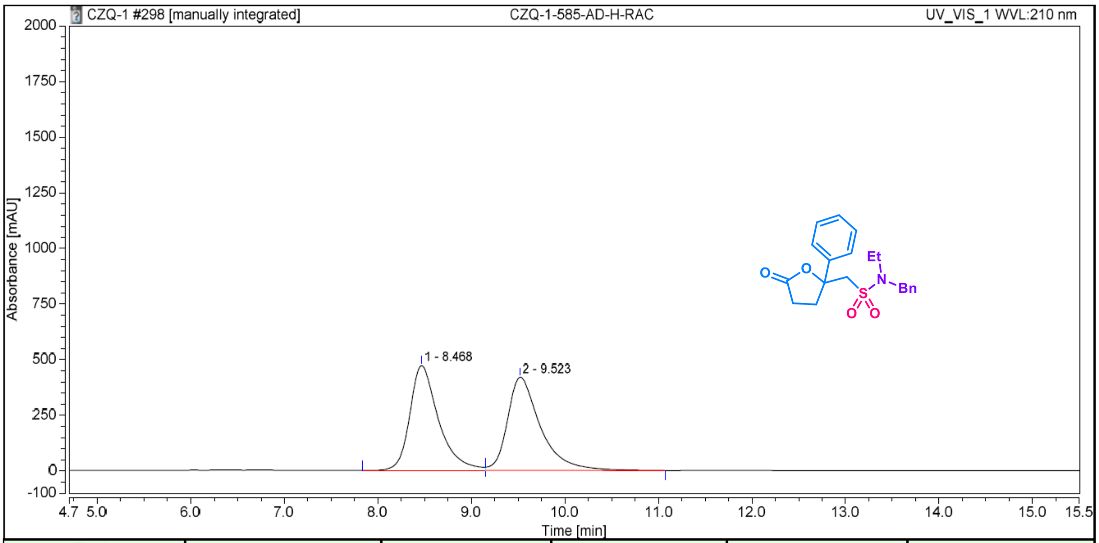

Peak	RetTime min	Area mAU*min	Height mAU	Area %	Height %
1	8.468	168.026	471.546	49.51	52.95
2	9.523	171.374	419.038	50.49	47.05
**Total:**		**339.401**	**890.584**	**100.00**	**100.00**

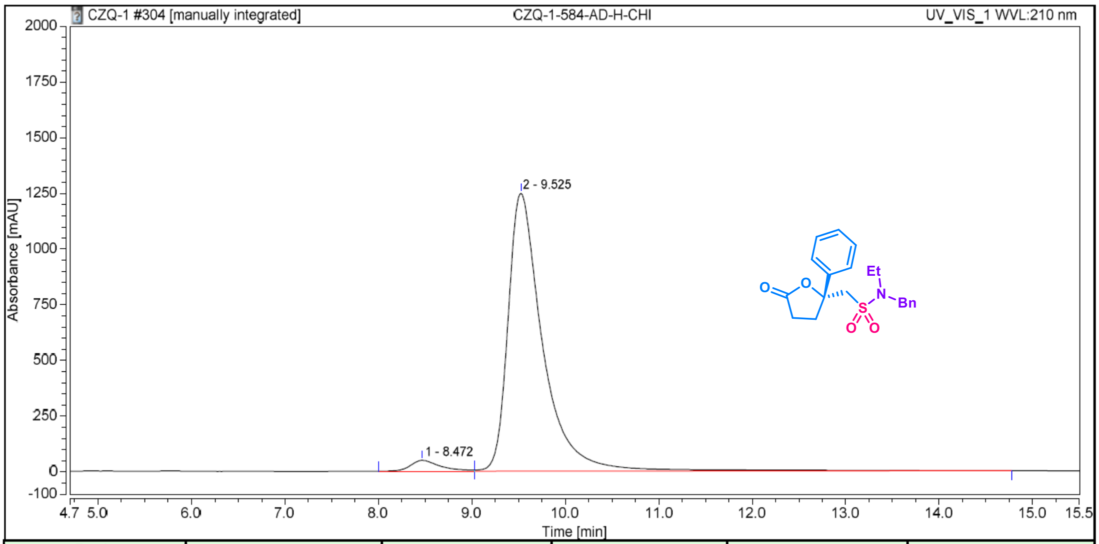

Peak	RetTime min	Area mAU*min	Height mAU	Area %	Height %
1	8.472	17.924	48.642	3.20	3.75
2	9.525	541.351	1247.798	96.80	96.25
**Total:**		**559.275**	**1296.440**	**100.00**	**100.00**

(*R*)-*N*-methyl-*N*-(naphthalen-1-ylmethyl)-1-(5-oxo-2-phenyltetrahydrofuran-2-yl)methane-sulfonamide

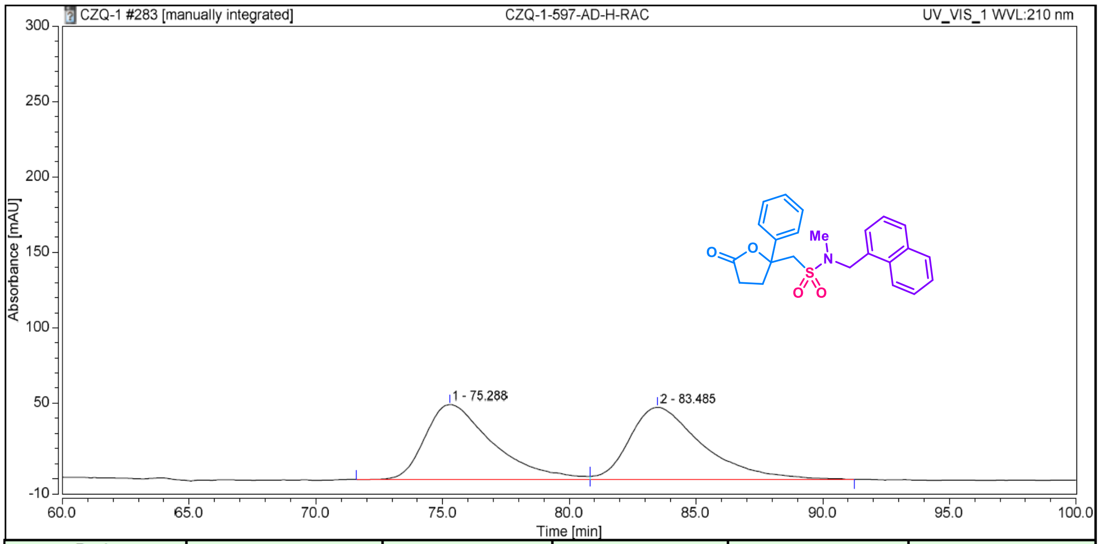

Peak	RetTime min	Area mAU*min	Height mAU	Area %	Height %
1	75.288	152.630	49.575	49.10	50.94
2	83.485	158.197	47.742	50.90	49.06
**Total:**		**310.827**	**97.317**	**100.00**	**100.00**

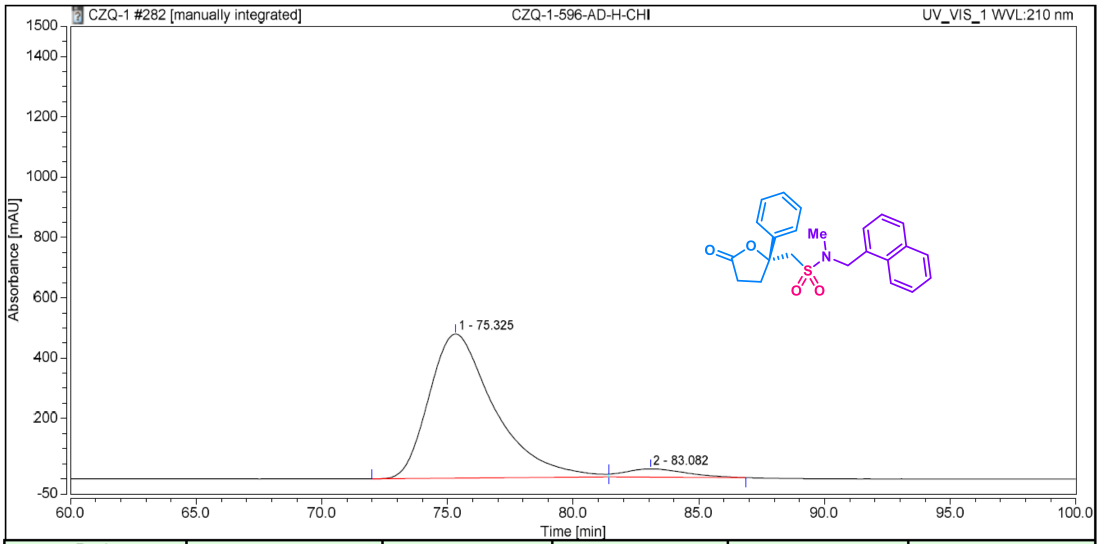

Peak	RetTime min	Area mAU*min	Height mAU	Area %	Height %
1	75.325	1444.471	477.930	95.04	94.62
2	83.082	75.442	27.177	4.96	5.38
**Total:**		**1519.914**	**505.108**	**100.00**	**100.00**

(*R*)-*N*-(2-cyanoethyl)-*N*-methyl-1-(5-oxo-2-phenyltetrahydrofuran-2-yl)methanesulfon-amide

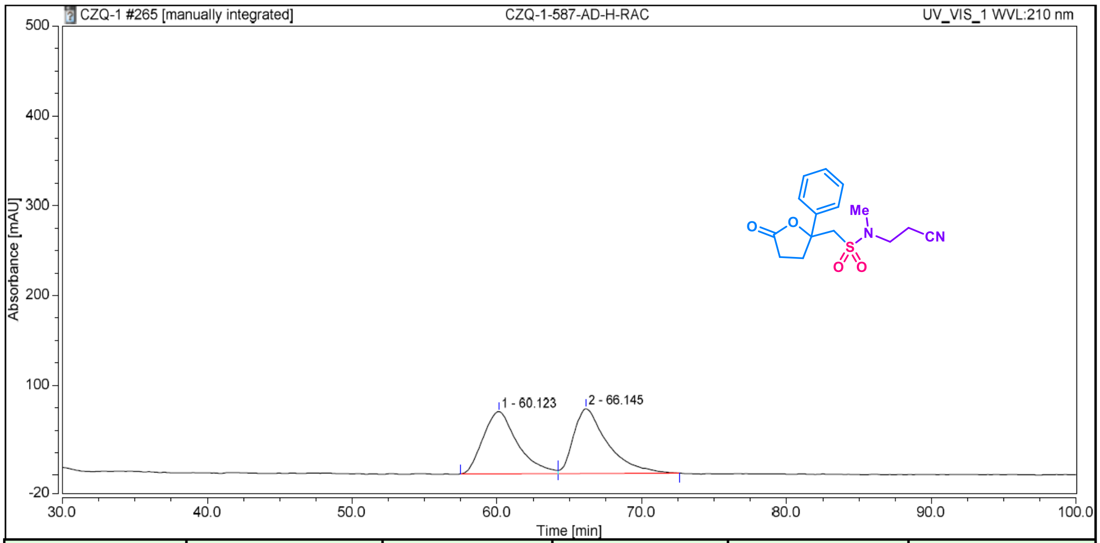

Peak	RetTime<br>min	Area<br>mAU*min	Height<br>mAU	Area<br>%	Height<br>%
1	60.123	191.549	69.309	49.46	49.03
2	66.145	195.741	72.054	50.54	50.97
**Total:**		**387.291**	**141.364**	**100.00**	**100.00**

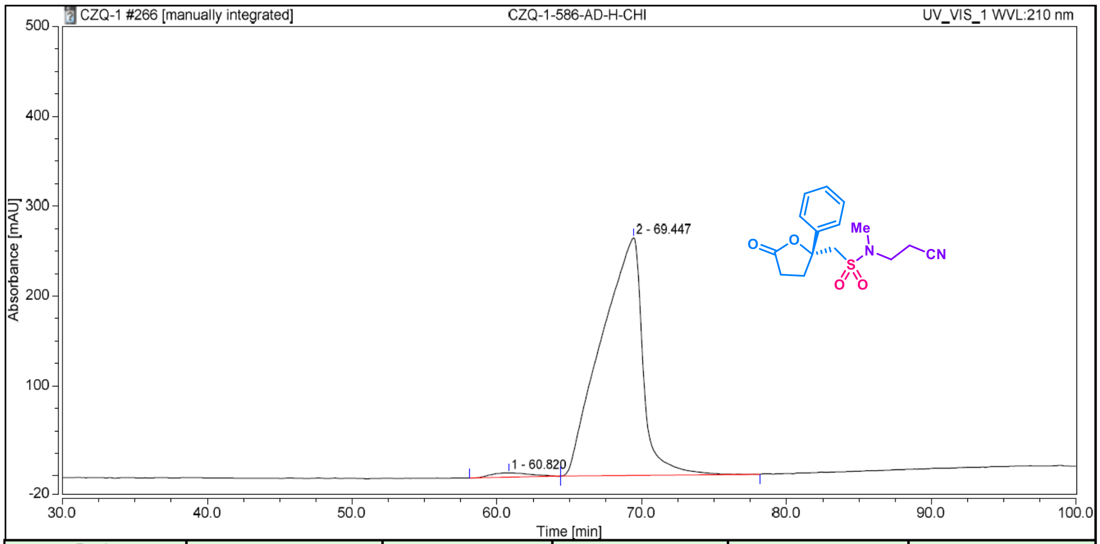

Peak	RetTime<br>min	Area<br>mAU*min	Height<br>mAU	Area<br>%	Height<br>%
1	60.820	14.851	4.719	1.67	1.75
2	69.447	874.282	264.456	98.33	98.25
**Total:**		**889.133**	**269.175**	**100.00**	**100.00**

(*R*)-*N*-(3-(10,11-dihydro-5*H*-dibenzo[*a*,*d*][7]annulen-5-ylidene)propyl)-*N*-methyl-1-(5-oxo-2-phenyltetrahydrofuran-2-yl)methanesulfonamide

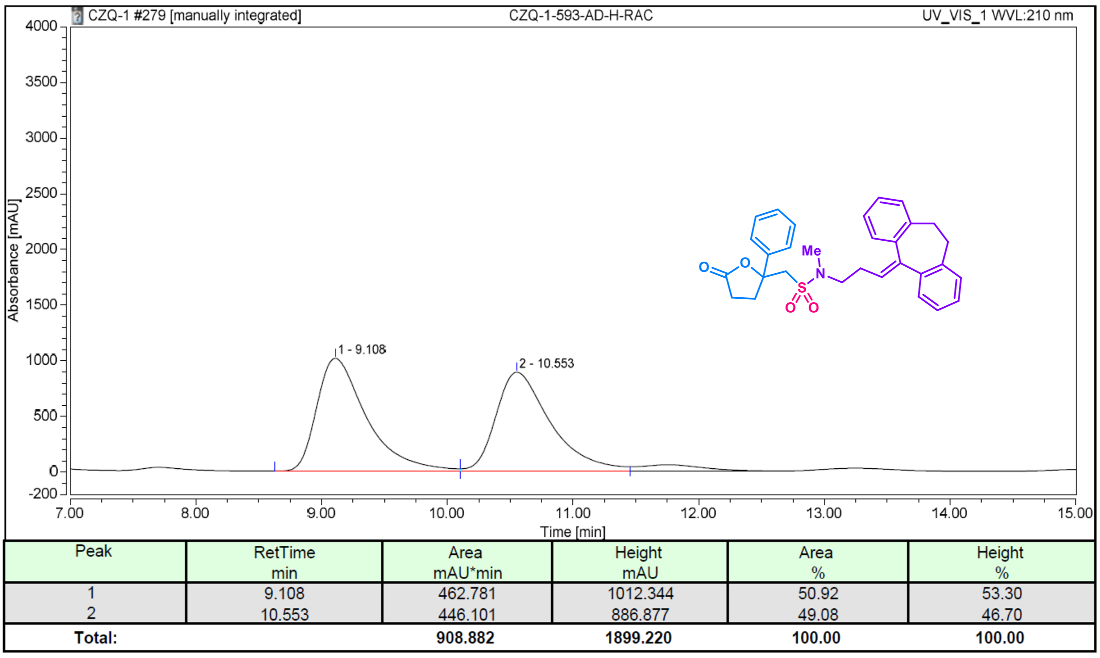

Peak	RetTime min	Area mAU*min	Height mAU	Area %	Height %
1	9.108	462.781	1012.344	50.92	53.30
2	10.553	446.101	886.877	49.08	46.70
**Total:**		**908.882**	**1899.220**	**100.00**	**100.00**

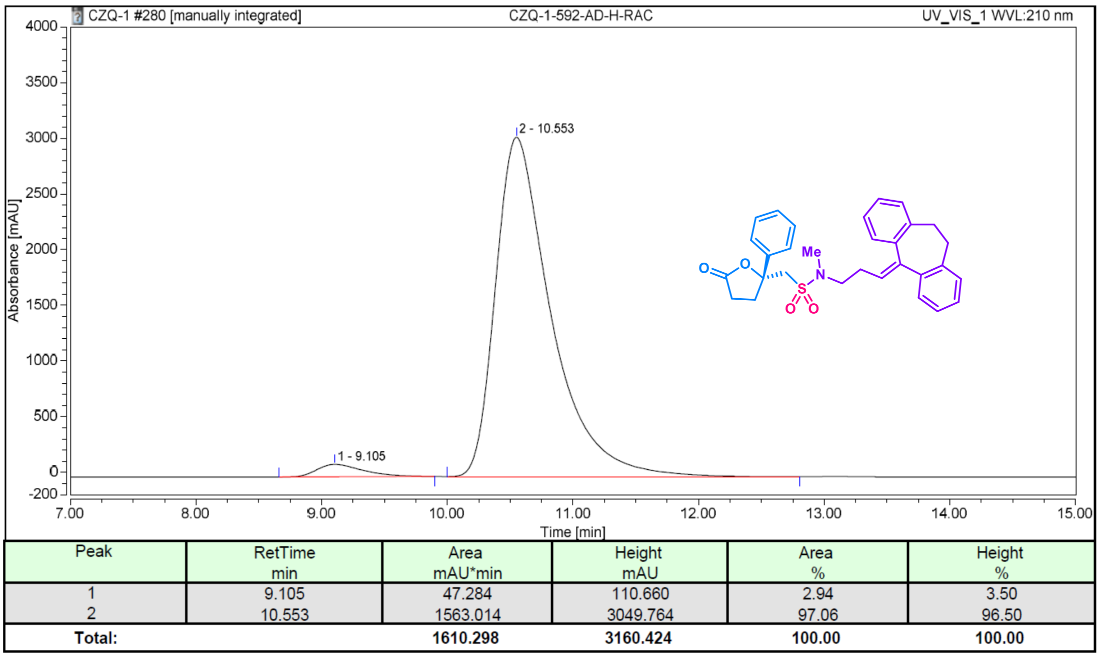

Peak	RetTime min	Area mAU*min	Height mAU	Area %	Height %
1	9.105	47.284	110.660	2.94	3.50
2	10.553	1563.014	3049.764	97.06	96.50
**Total:**		**1610.298**	**3160.424**	**100.00**	**100.00**

N-(3-((9r,10R)-9,10-ethanoanthracen-9(10H)-yl)propyl)-N-methyl-1-((R)-5-oxo-2-phenyltetrahydrofuran-2-yl)methanesulfonamide

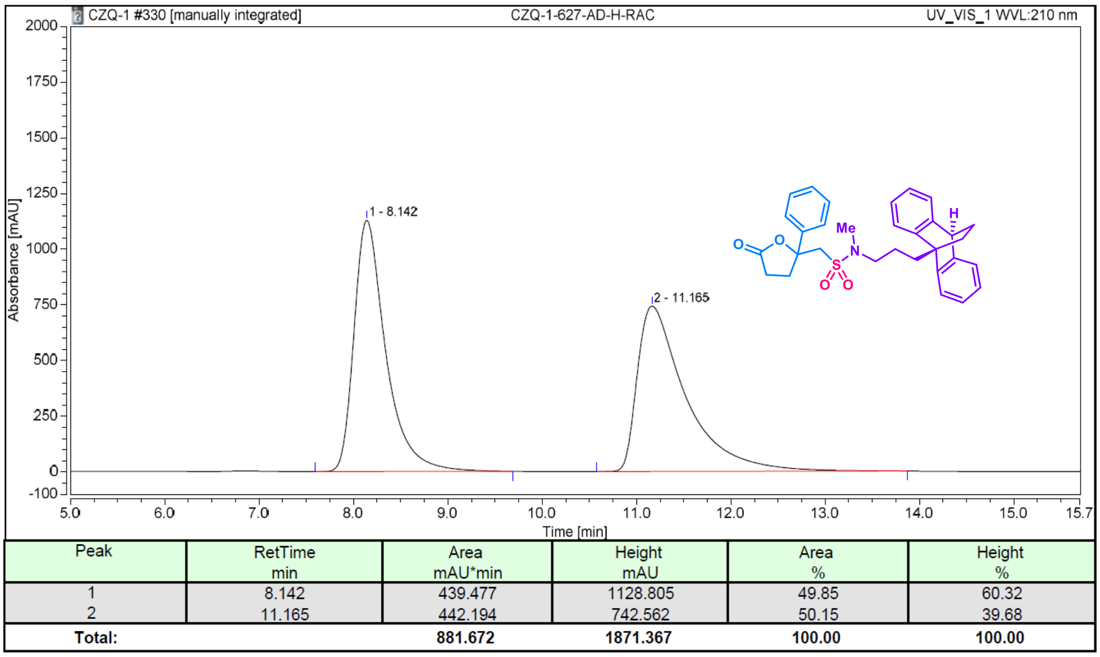

Peak	RetTime min	Area mAU*min	Height mAU	Area %	Height %
1	8.142	439.477	1128.805	49.85	60.32
2	11.165	442.194	742.562	50.15	39.68
**Total:**		**881.672**	**1871.367**	**100.00**	**100.00**

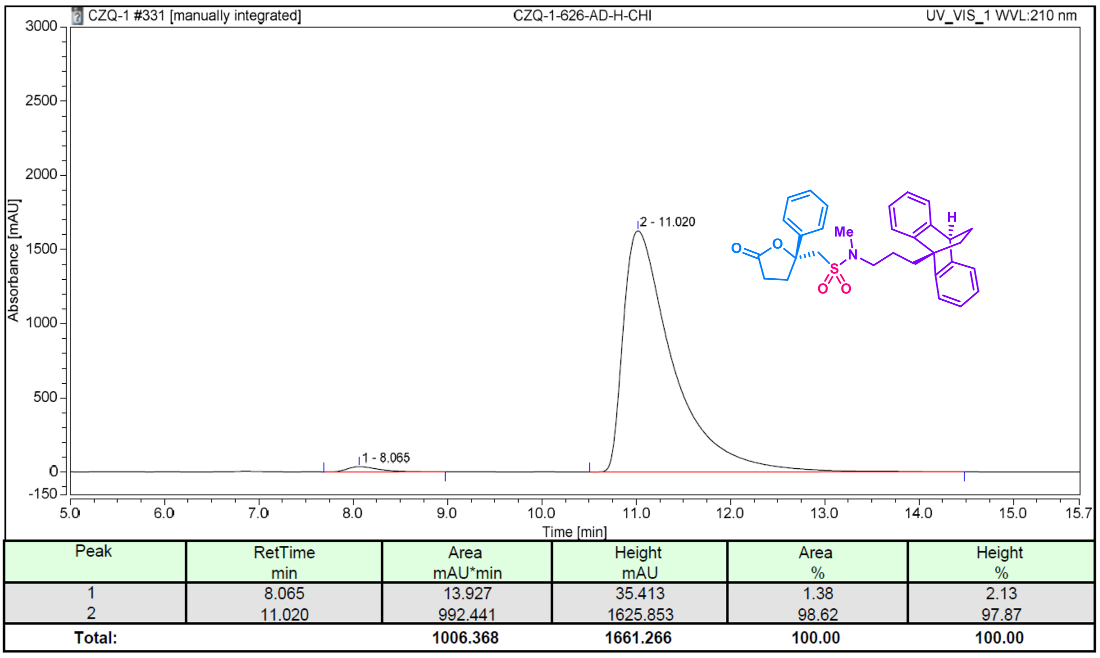

Peak	RetTime min	Area mAU*min	Height mAU	Area %	Height %
1	8.065	13.927	35.413	1.38	2.13
2	11.020	992.441	1625.853	98.62	97.87
**Total:**		**1006.368**	**1661.266**	**100.00**	**100.00**

_N_-methyl-_N_-((_S_)-3-(naphthalen-1-yloxy)-3-(thiophen-2-yl)propyl)-1-((_R_)-5-oxo-2-phenyl-tetrahydrofuran-2-yl)methanesulfonamide

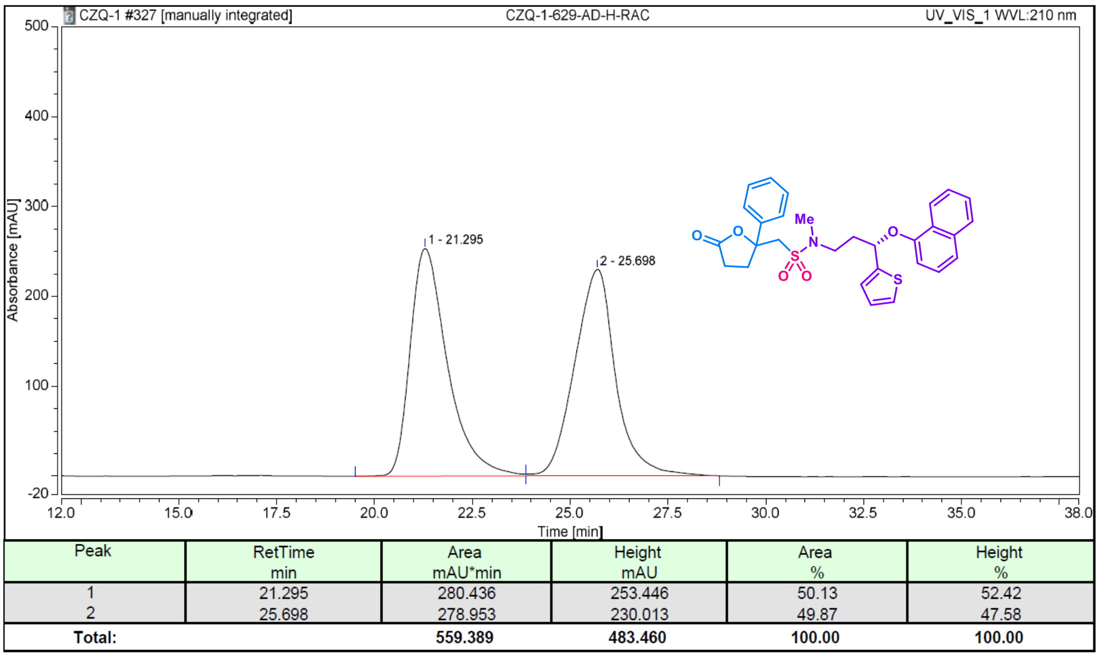

Peak	RetTime min	Area mAU*min	Height mAU	Area %	Height %
1	21.295	280.436	253.446	50.13	52.42
2	25.698	278.953	230.013	49.87	47.58
**Total:**		**559.389**	**483.460**	**100.00**	**100.00**

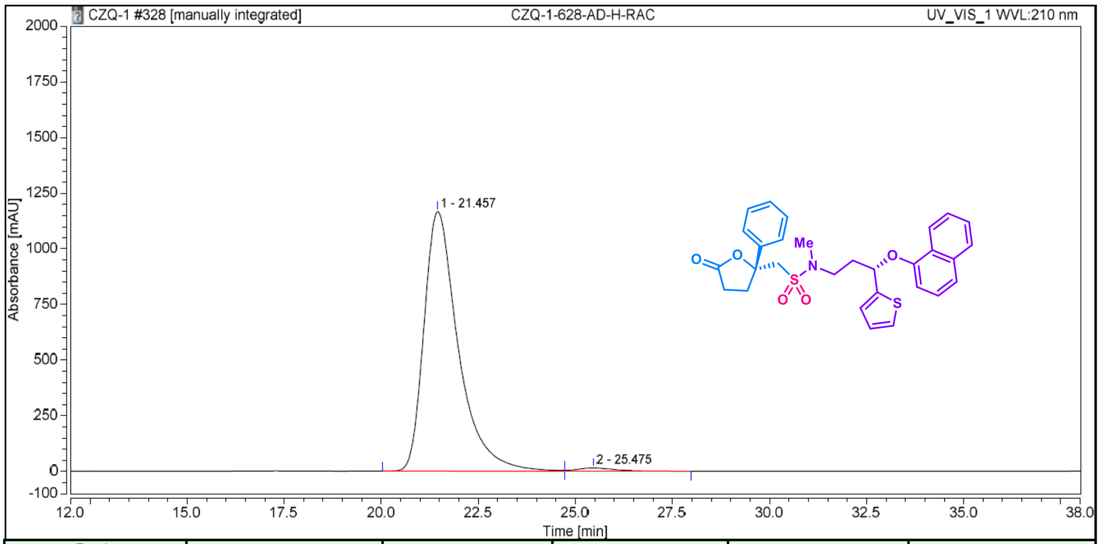

Peak	RetTime min	Area mAU*min	Height mAU	Area %	Height %
1	21.457	1193.976	1167.083	98.63	98.76
2	25.475	16.645	14.609	1.37	1.24
**Total:**		**1210.621**	**1181.692**	**100.00**	**100.00**

(*R*)-5-((((3*S*,4*R*)-3-((benzo[*d*][1,3]dioxol-5-yloxy)methyl)-4-(4-fluorophenyl)piperidin-1-yl)sulfonyl) methyl)-5-phenyldihydrofuran-2(3*H*)-one

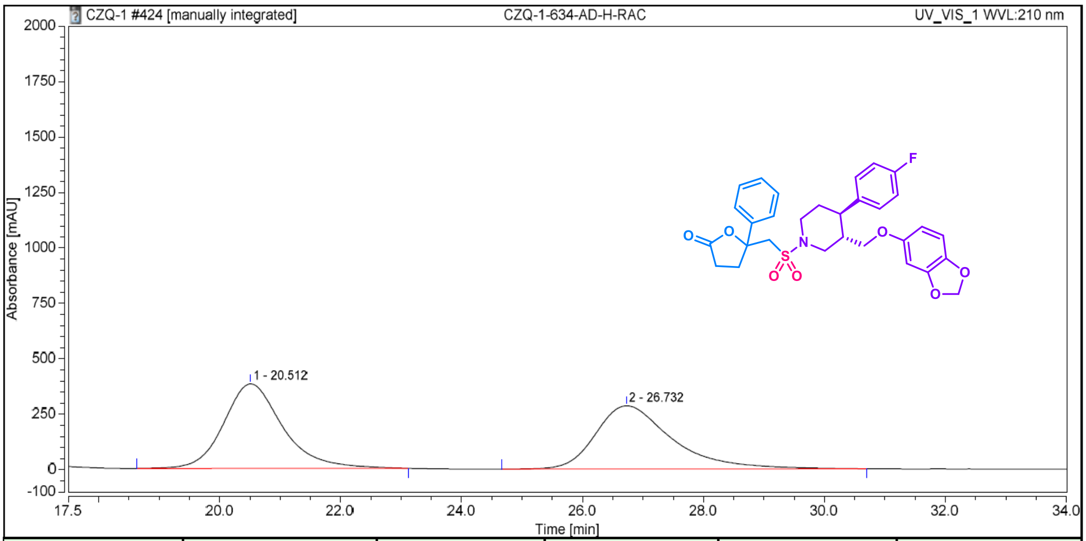

Peak	RetTime<br>min	Area<br>mAU*min	Height<br>mAU	Area<br>%	Height<br>%
1	20.512	431.500	381.052	50.82	57.19
2	26.732	417.541	285.256	49.18	42.81
**Total:**		**849.042**	**666.308**	**100.00**	**100.00**

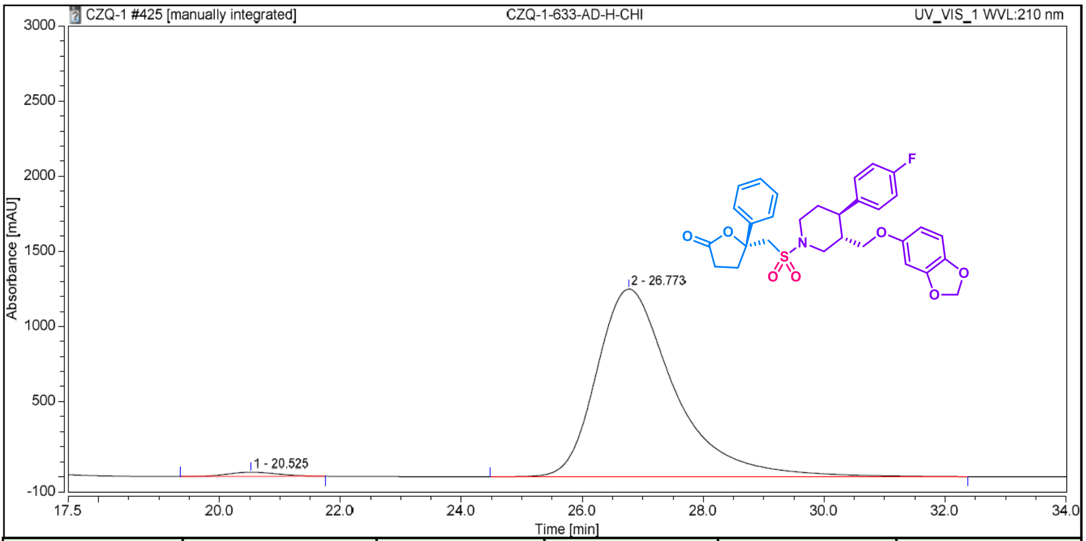

Peak	RetTime<br>min	Area<br>mAU*min	Height<br>mAU	Area<br>%	Height<br>%
1	20.525	27.366	27.989	1.45	2.19
2	26.773	1864.384	1249.424	98.55	97.81
**Total:**		**1891.750**	**1277.414**	**100.00**	**100.00**

(5*R*)-5-(((4-((5,6-dimethoxy-1-oxo-2,3-dihydro-1*H*-inden-2-yl)methyl)piperidin-1-yl)sulfonyl) methyl)-5-phenyldihydrofuran-2(3*H*)-one

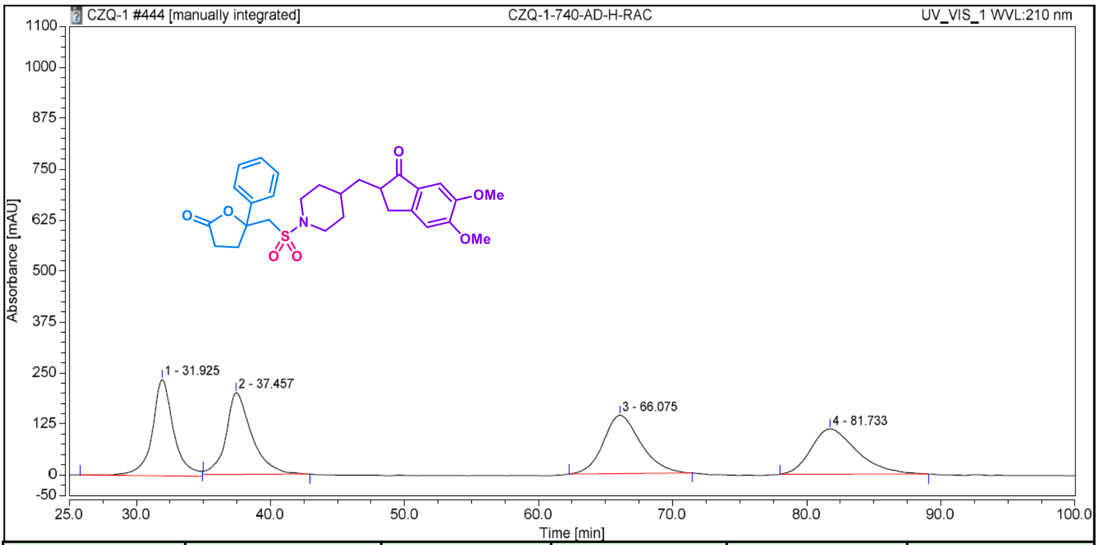

Peak	RetTime min	Area mAU*min	Height mAU	Area %	Height %
1	31.925	435.568	236.135	24.34	34.18
2	37.457	449.640	200.692	25.13	29.05
3	66.075	462.947	143.024	25.87	20.70
4	81.733	441.434	110.952	24.67	16.06
**Total:**		**1789.589**	**690.803**	**100.00**	**100.00**

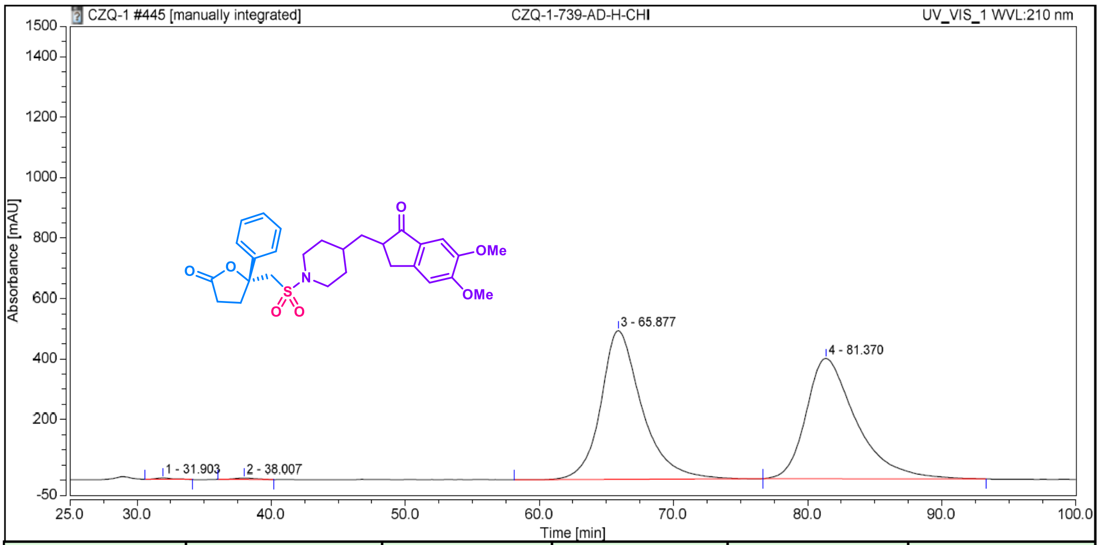

Peak	RetTime min	Area mAU*min	Height mAU	Area %	Height %
1	31.903	8.920	5.567	0.26	0.62
2	38.007	8.980	4.826	0.26	0.54
3	65.877	1747.008	492.092	50.10	54.65
4	81.370	1722.476	397.987	49.39	44.20
**Total:**		**3487.384**	**900.472**	**100.00**	**100.00**

(*R*)-4-(((5-oxo-2-phenyltetrahydrofuran-2-yl)methyl)sulfonyl)butanenitrile

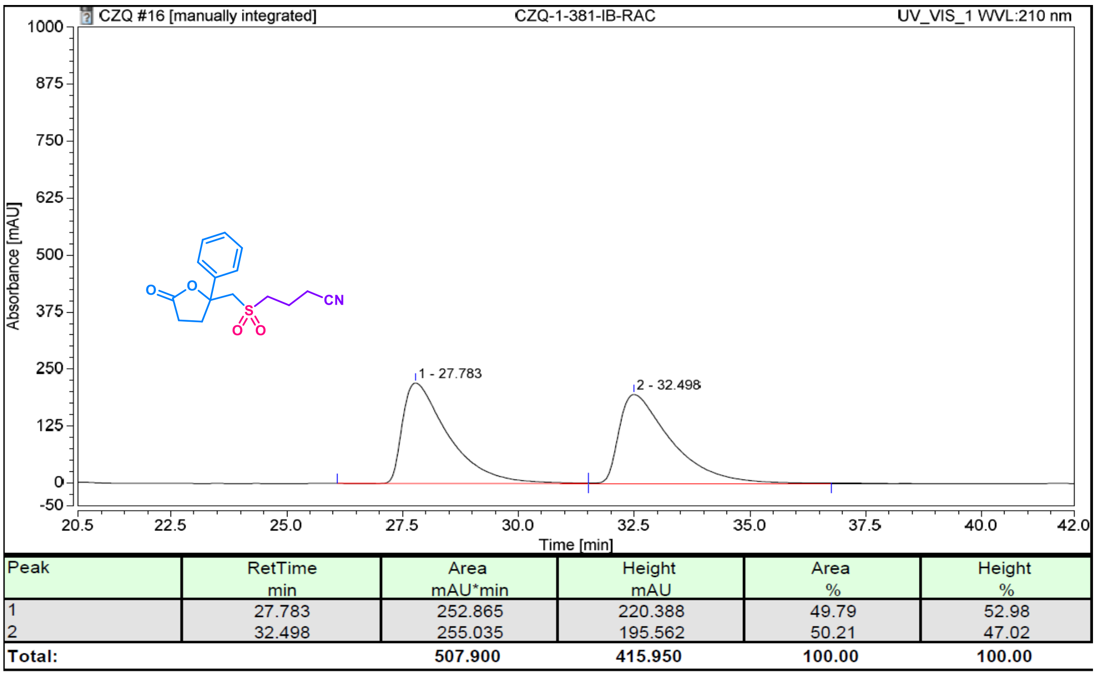

Peak	RetTime min	Area mAU*min	Height mAU	Area %	Height %
1	27.783	252.865	220.388	49.79	52.98
2	32.498	255.035	195.562	50.21	47.02
**Total:**		**507.900**	**415.950**	**100.00**	**100.00**

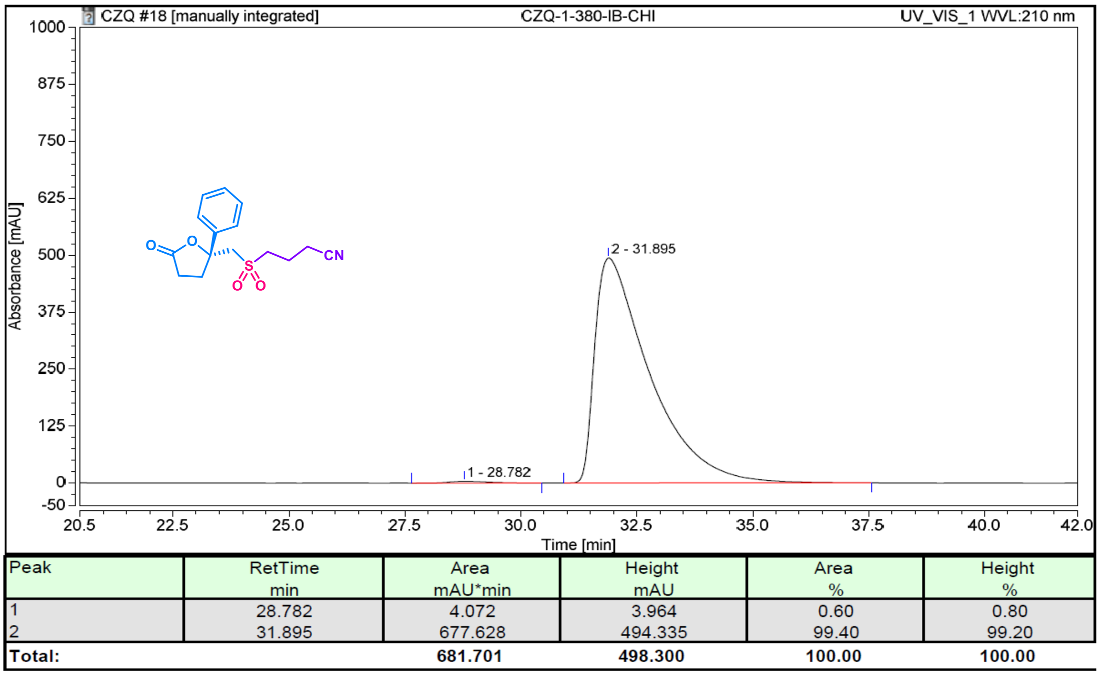

Peak	RetTime min	Area mAU*min	Height mAU	Area %	Height %
1	28.782	4.072	3.964	0.60	0.80
2	31.895	677.628	494.335	99.40	99.20
**Total:**		**681.701**	**498.300**	**100.00**	**100.00**

(*R*)-4-(((2-([1,1'-biphenyl]-4-yl)-5-oxotetrahydrofuran-2-yl)methyl)sulfonyl) butanenitrile

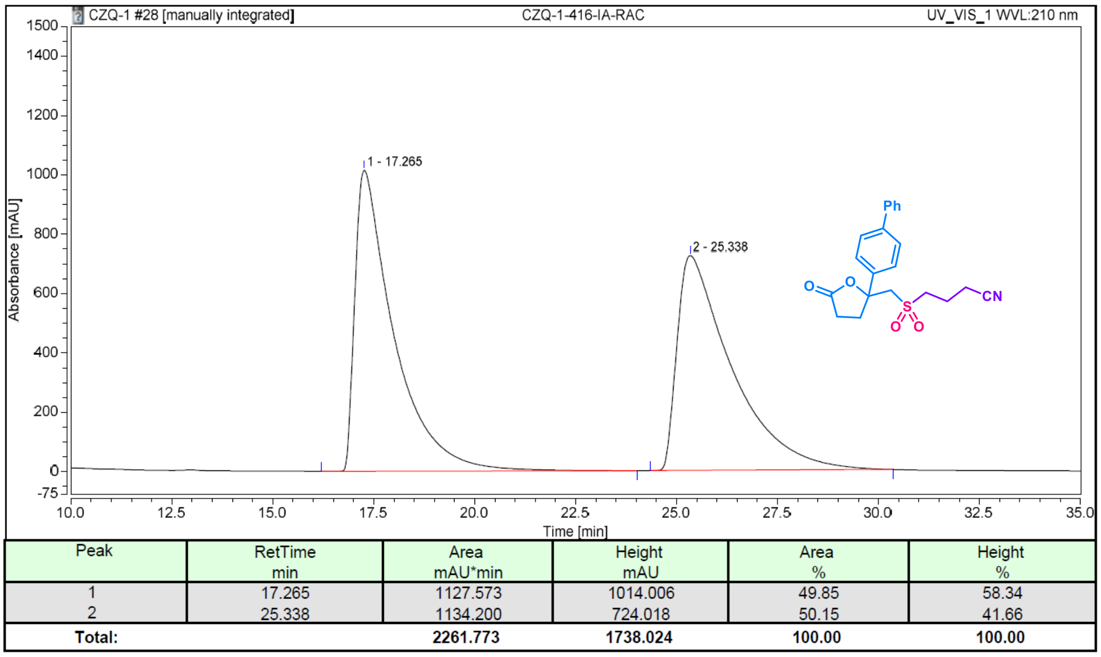

Peak	RetTime min	Area mAU*min	Height mAU	Area %	Height %
1	17.265	1127.573	1014.006	49.85	58.34
2	25.338	1134.200	724.018	50.15	41.66
**Total:**		**2261.773**	**1738.024**	**100.00**	**100.00**

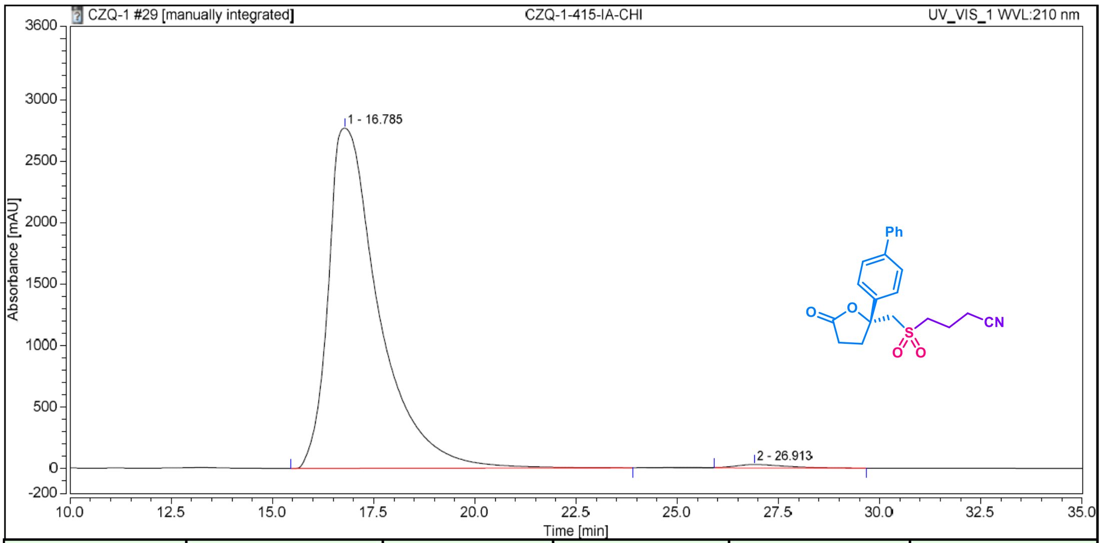

Peak	RetTime min	Area mAU*min	Height mAU	Area %	Height %
1	16.785	4010.555	2768.110	99.02	99.01
2	26.913	39.706	27.538	0.98	0.99
**Total:**		**4050.261**	**2795.648**	**100.00**	**100.00**

(*R*)-4-(((2-(naphthalen-2-yl)-5-oxotetrahydrofuran-2-yl)methyl)sulfonyl) butanenitrile

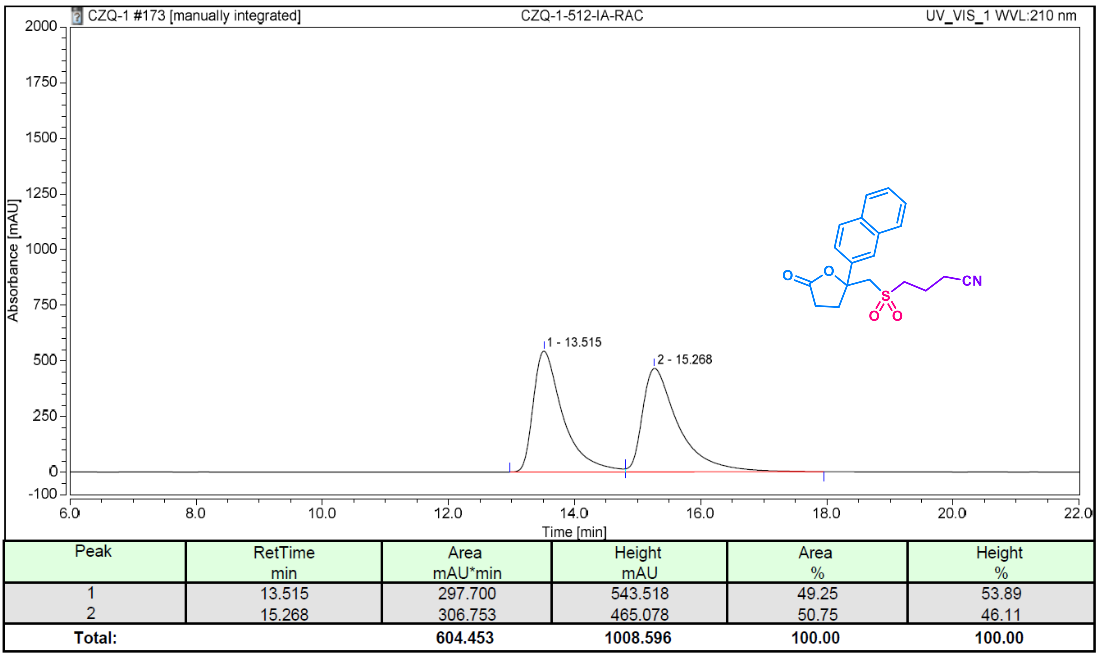

Peak	RetTime min	Area mAU*min	Height mAU	Area %	Height %
1	13.515	297.700	543.518	49.25	53.89
2	15.268	306.753	465.078	50.75	46.11
**Total:**		**604.453**	**1008.596**	**100.00**	**100.00**

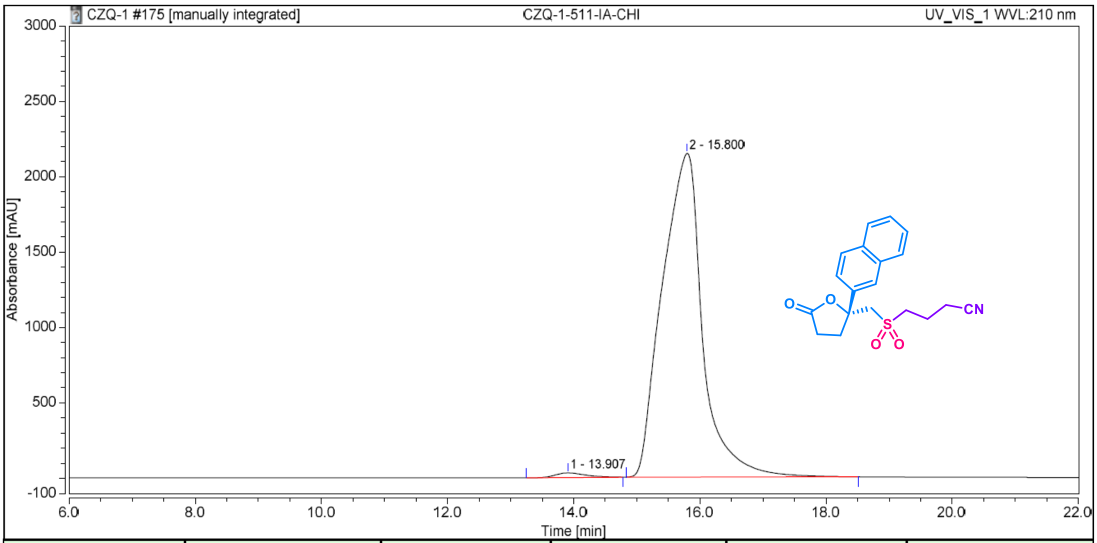

Peak	RetTime min	Area mAU*min	Height mAU	Area %	Height %
1	13.907	16.756	30.637	1.05	1.41
2	15.800	1581.210	2147.820	98.95	98.59
**Total:**		**1597.966**	**2178.458**	**100.00**	**100.00**

(*R*)-4-(((5-oxo-2-(pyren-1-yl)tetrahydrofuran-2-yl)methyl)sulfonyl)butanenitrile

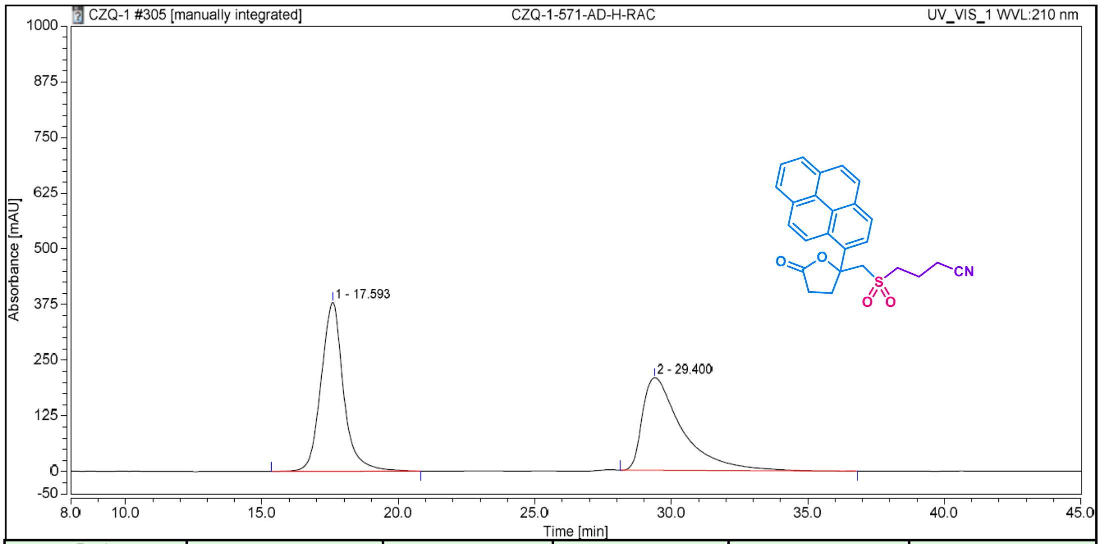

Peak	RetTime min	Area mAU*min	Height mAU	Area %	Height %
1	17.593	360.970	379.423	50.78	64.60
2	29.400	349.940	207.908	49.22	35.40
**Total:**		**710.909**	**587.330**	**100.00**	**100.00**

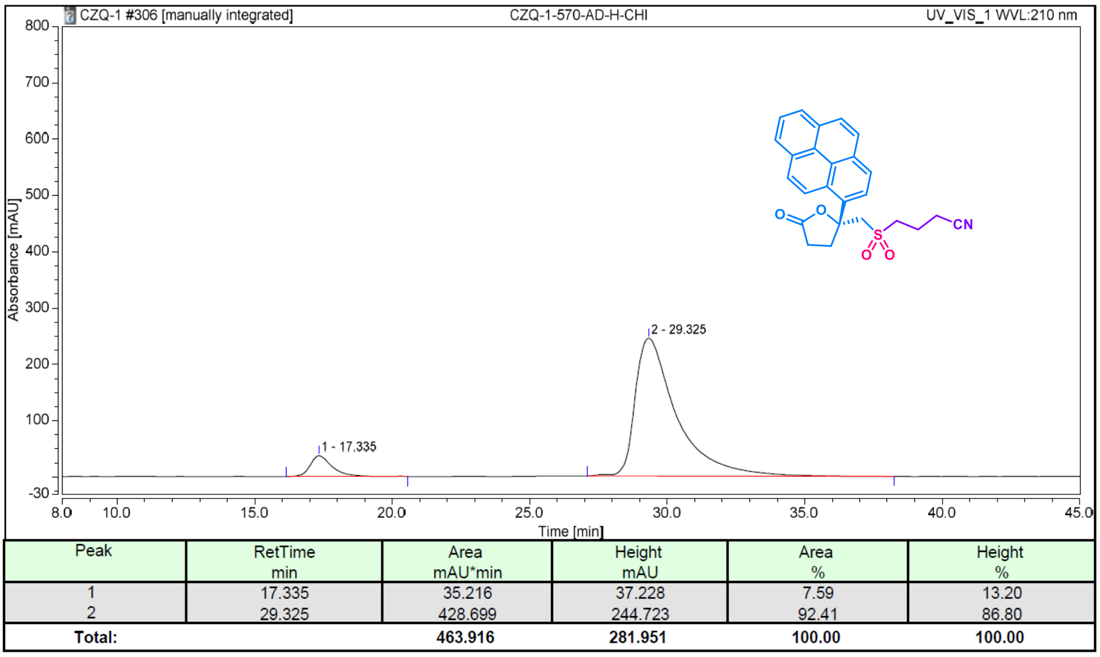

Peak	RetTime min	Area mAU*min	Height mAU	Area %	Height %
1	17.335	35.216	37.228	7.59	13.20
2	29.325	428.699	244.723	92.41	86.80
**Total:**		**463.916**	**281.951**	**100.00**	**100.00**

(*R*)-4-(((5-oxo-2-(o-tolyl)tetrahydrofuran-2-yl)methyl)sulfonyl)butanenitrile

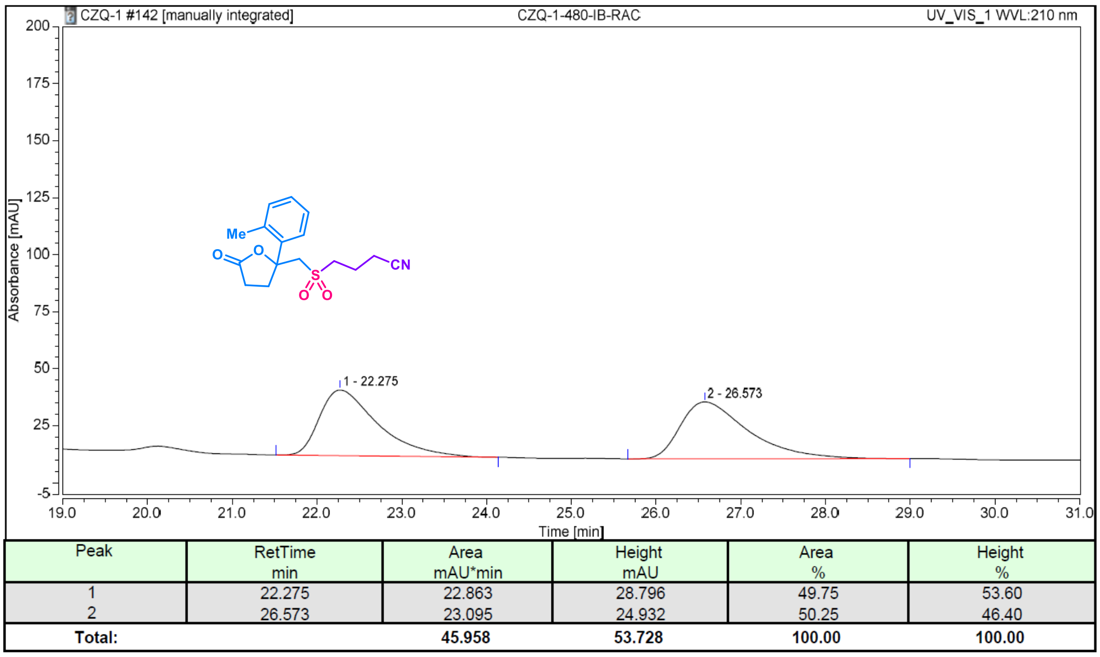

Peak	RetTime min	Area mAU*min	Height mAU	Area %	Height %
1	22.275	22.863	28.796	49.75	53.60
2	26.573	23.095	24.932	50.25	46.40
**Total:**		**45.958**	**53.728**	**100.00**	**100.00**

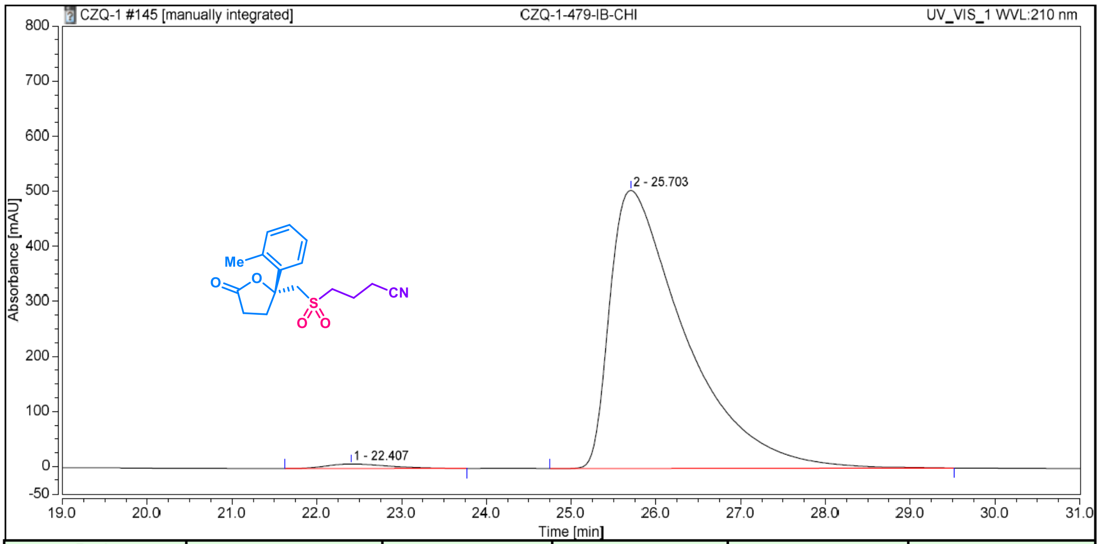

Peak	RetTime min	Area mAU*min	Height mAU	Area %	Height %
1	22.407	6.992	8.083	1.33	1.57
2	25.703	519.819	505.265	98.67	98.43
**Total:**		**526.811**	**513.348**	**100.00**	**100.00**

(*R*)-4-(((5-oxo-2-(m-tolyl)tetrahydrofuran-2-yl)methyl)sulfonyl)butanenitrile

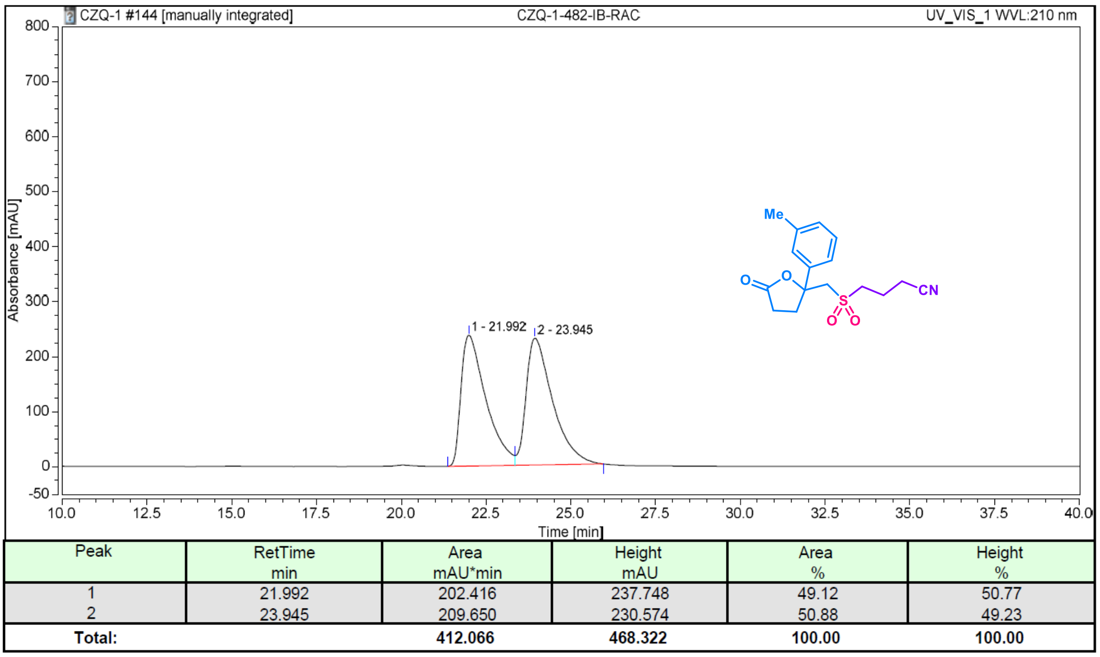

Peak	RetTime min	Area mAU*min	Height mAU	Area %	Height %
1	21.992	202.416	237.748	49.12	50.77
2	23.945	209.650	230.574	50.88	49.23
**Total:**		**412.066**	**468.322**	**100.00**	**100.00**

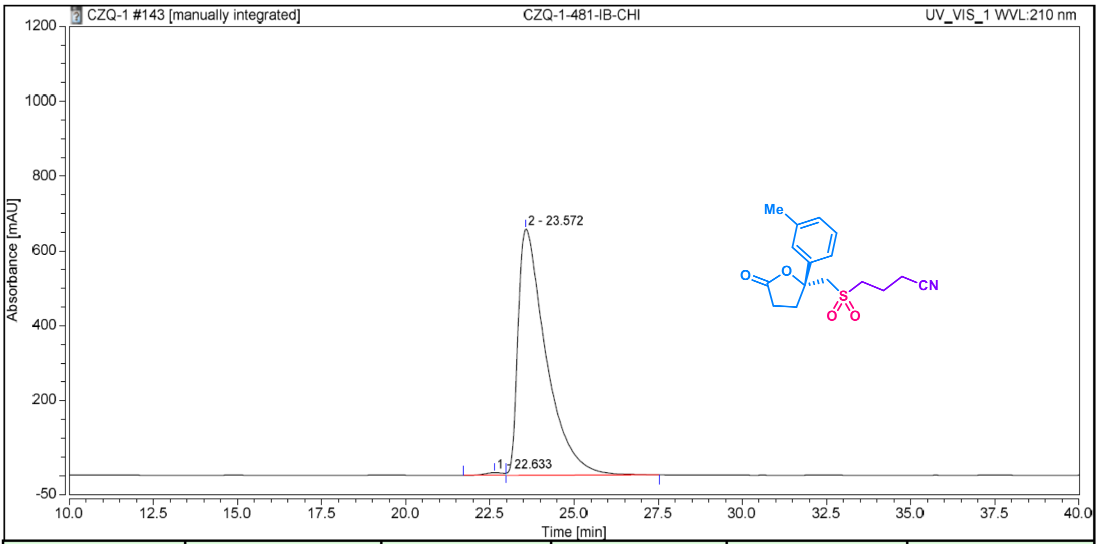

Peak	RetTime min	Area mAU*min	Height mAU	Area %	Height %
1	22.633	4.283	6.853	0.67	1.03
2	23.572	631.346	658.016	99.33	98.97
**Total:**		**635.629**	**664.869**	**100.00**	**100.00**

(*R*)-4-(((2-(4-(*tert*-butyl)phenyl)-5-oxotetrahydrofuran-2-yl)methyl)sulfonyl) butanenitrile

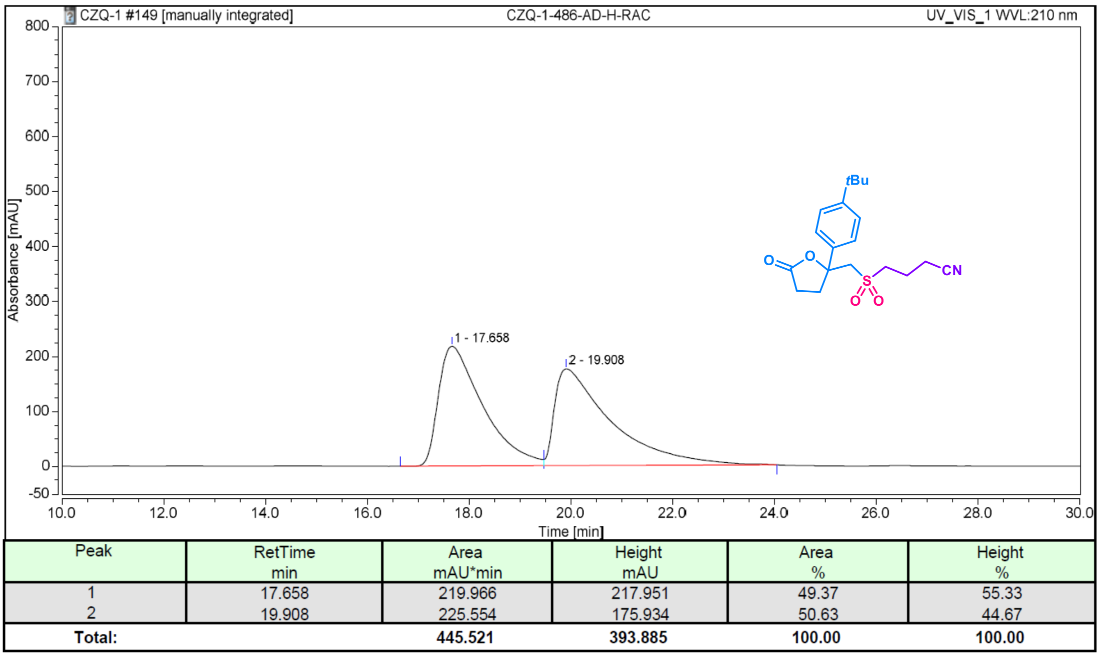

Peak	RetTime min	Area mAU*min	Height mAU	Area %	Height %
1	17.658	219.966	217.951	49.37	55.33
2	19.908	225.554	175.934	50.63	44.67
**Total:**		**445.521**	**393.885**	**100.00**	**100.00**

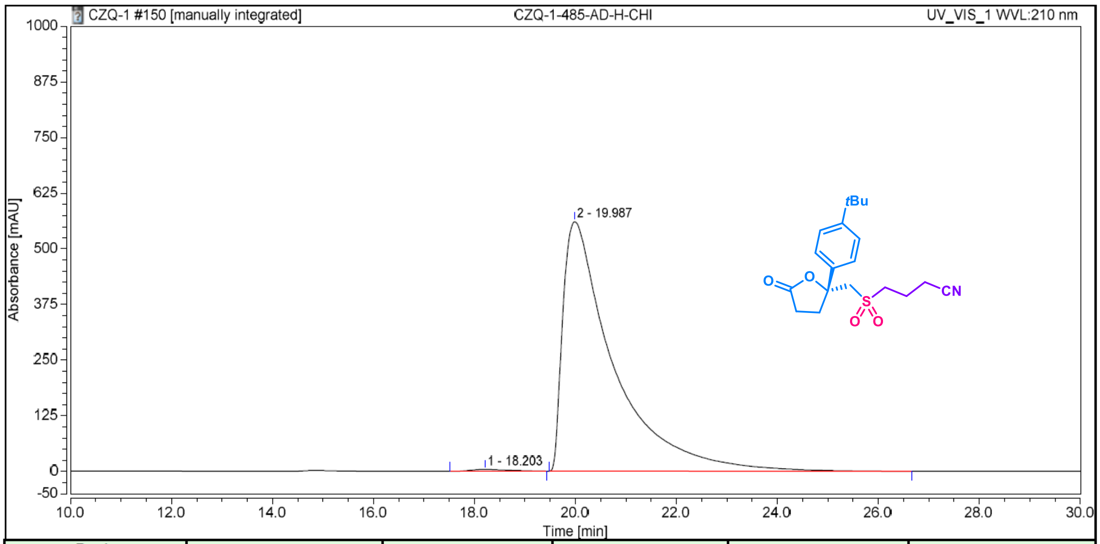

Peak	RetTime min	Area mAU*min	Height mAU	Area %	Height %
1	18.203	3.649	4.301	0.56	0.76
2	19.987	653.391	560.820	99.44	99.24
**Total:**		**657.040**	**565.121**	**100.00**	**100.00**

(*R*)-4-(((2-(3-methoxyphenyl)-5-oxotetrahydrofuran-2-yl)methyl)sulfonyl) butanenitrile

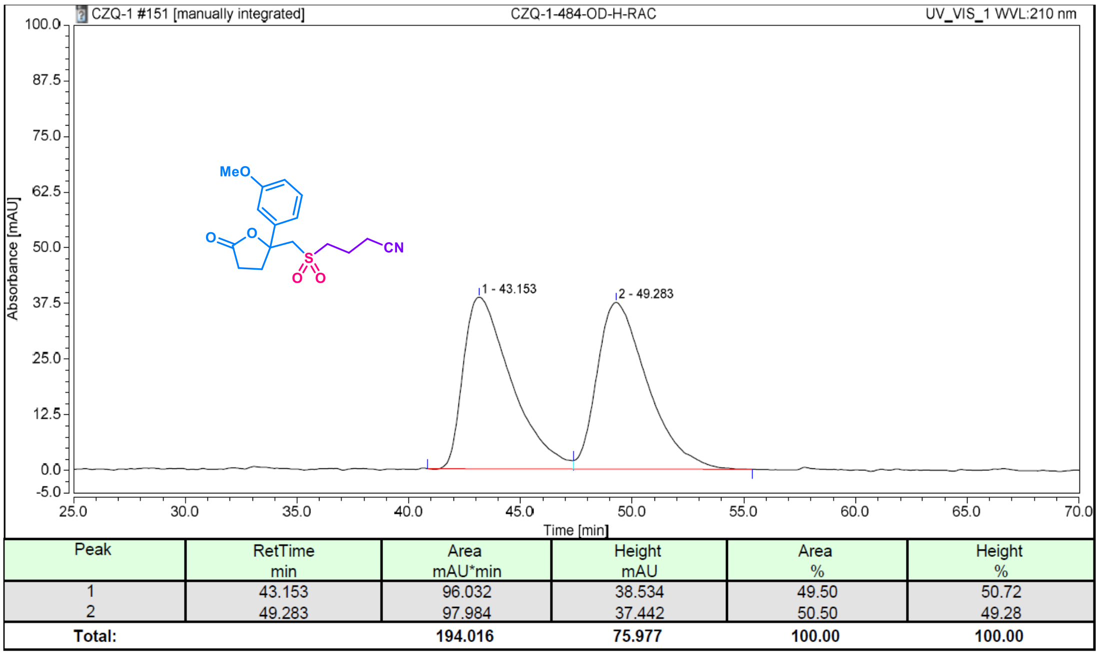

Peak	RetTime min	Area mAU*min	Height mAU	Area %	Height %
1	43.153	96.032	38.534	49.50	50.72
2	49.283	97.984	37.442	50.50	49.28
**Total:**		**194.016**	**75.977**	**100.00**	**100.00**

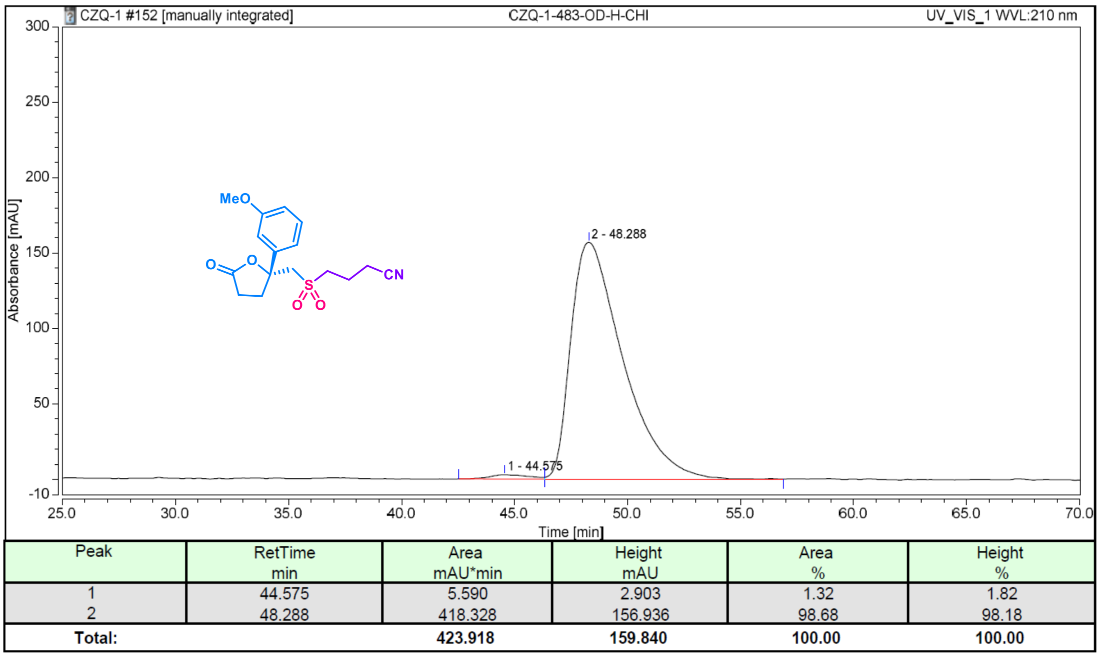

Peak	RetTime min	Area mAU*min	Height mAU	Area %	Height %
1	44.575	5.590	2.903	1.32	1.82
2	48.288	418.328	156.936	98.68	98.18
**Total:**		**423.918**	**159.840**	**100.00**	**100.00**

(*R*)-4-(((2-(3-fluorophenyl)-5-oxotetrahydrofuran-2-yl)methyl)sulfonyl) butanenitrile

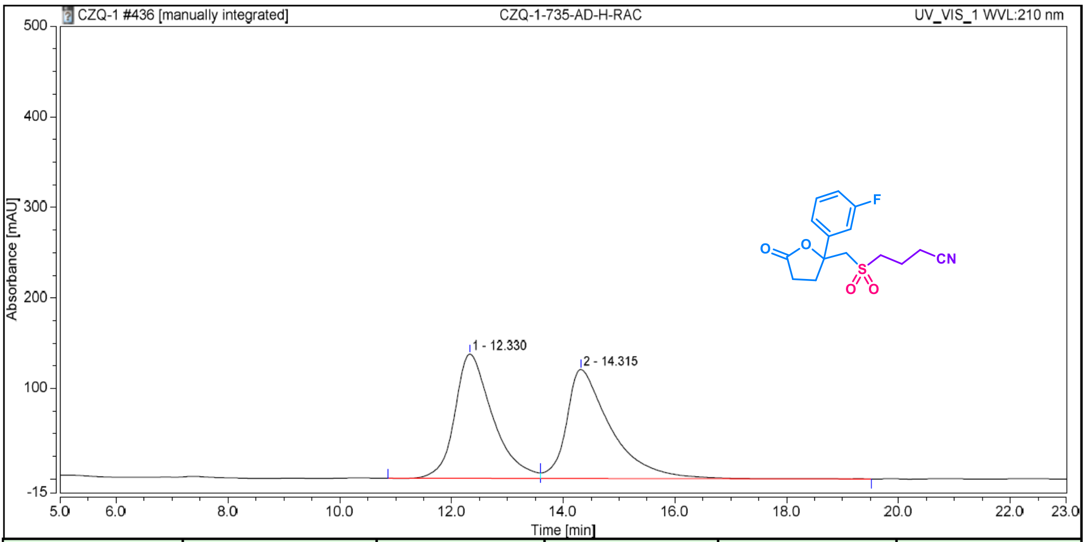

Peak	RetTime min	Area mAU*min	Height mAU	Area %	Height %
1	12.330	107.536	137.304	49.31	53.26
2	14.315	110.560	120.506	50.69	46.74
**Total:**		**218.095**	**257.810**	**100.00**	**100.00**

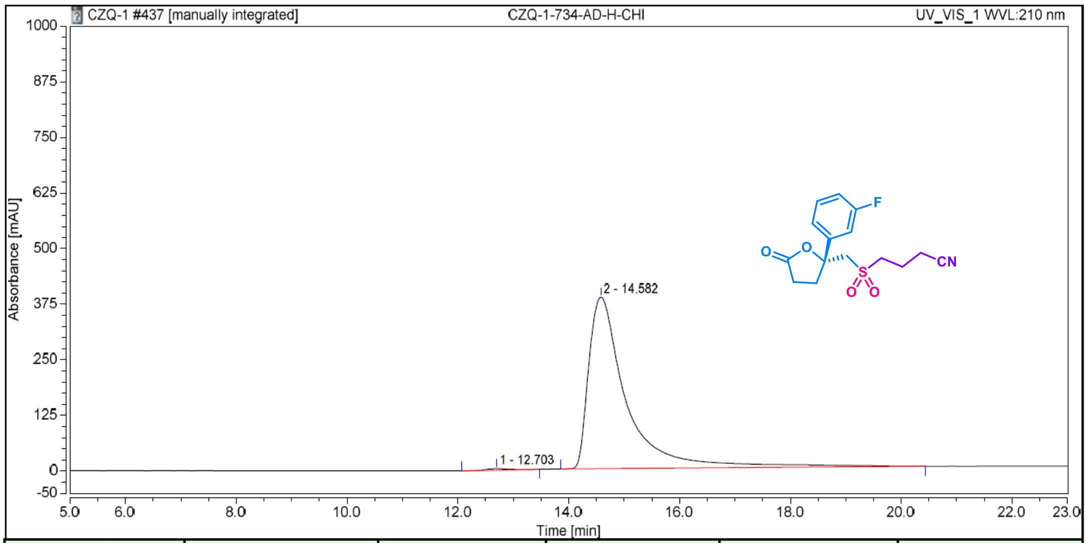

Peak	RetTime min	Area mAU*min	Height mAU	Area %	Height %
1	12.703	1.539	3.320	0.50	0.85
2	14.582	307.758	385.962	99.50	99.15
**Total:**		**309.296**	**389.282**	**100.00**	**100.00**

(*R*)-4-(((2-(4-fluorophenyl)-5-oxotetrahydrofuran-2-yl)methyl)sulfonyl) butanenitrile

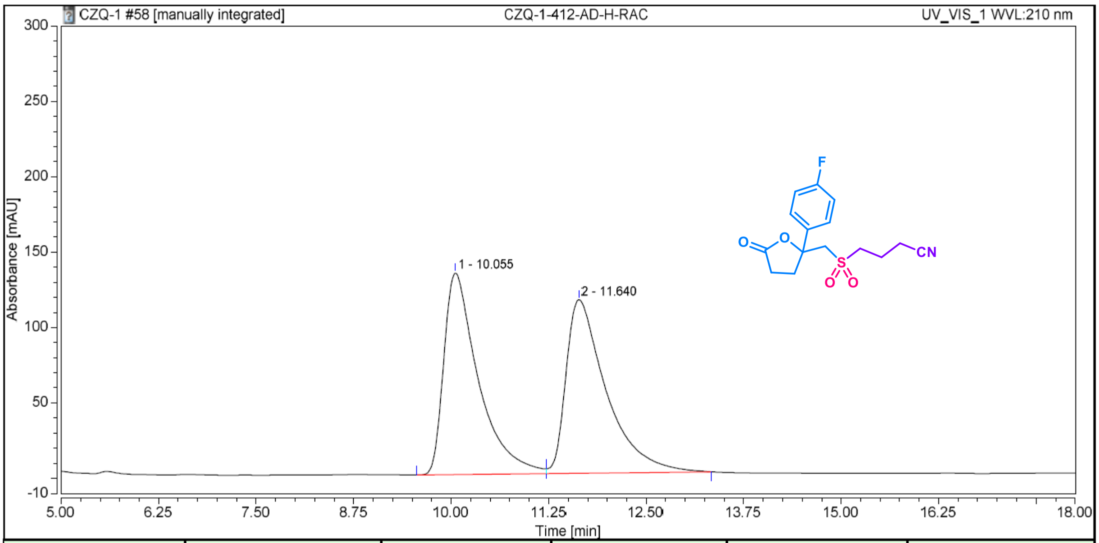

Peak	RetTime min	Area mAU*min	Height mAU	Area %	Height %
1	10.055	68.525	133.695	49.91	53.73
2	11.640	68.784	115.133	50.09	46.27
**Total:**		**137.309**	**248.828**	**100.00**	**100.00**

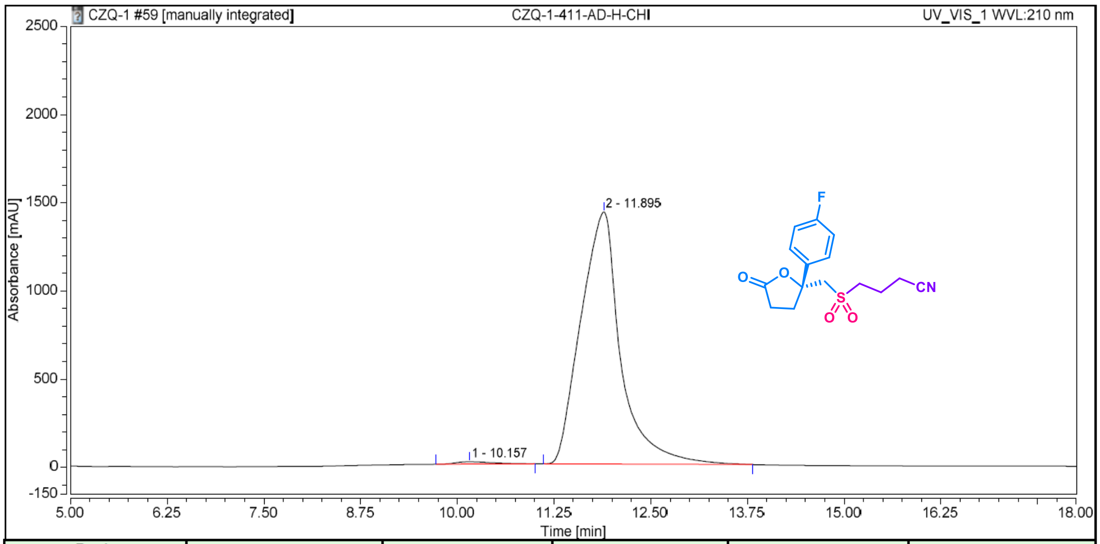

Peak	RetTime min	Area mAU*min	Height mAU	Area %	Height %
1	10.157	7.302	13.651	0.85	0.95
2	11.895	854.443	1429.835	99.15	99.05
**Total:**		**861.745**	**1443.485**	**100.00**	**100.00**

(*R*)-4-(((2-(4-iodophenyl)-5-oxotetrahydrofuran-2-yl)methyl)sulfonyl) butanenitrile

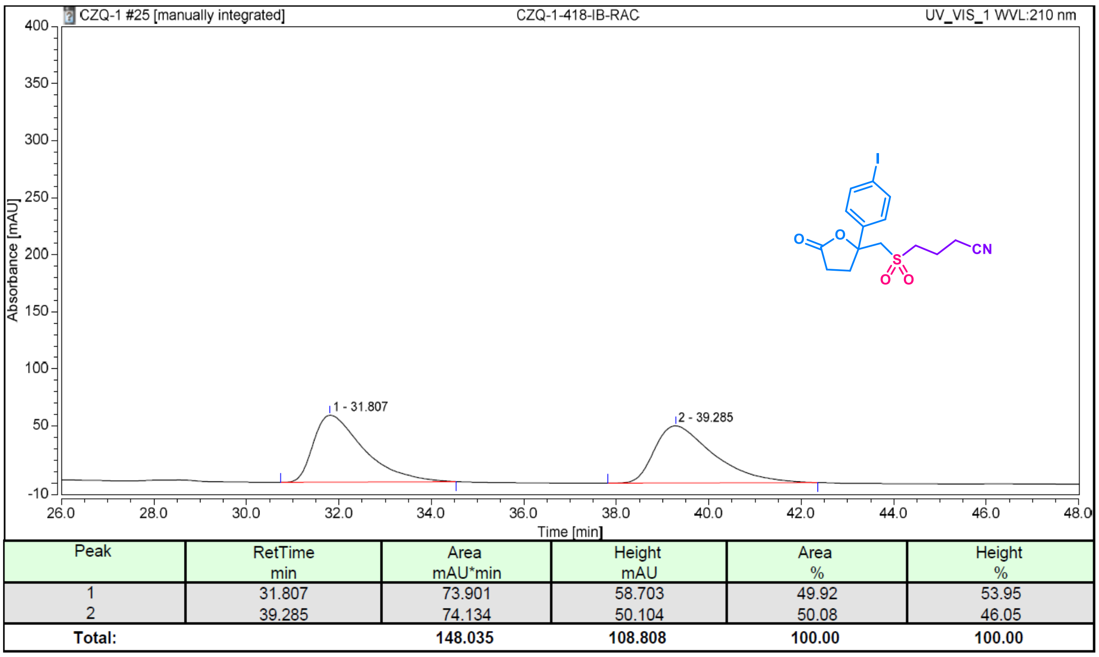

Peak	RetTime min	Area mAU*min	Height mAU	Area %	Height %
1	31.807	73.901	58.703	49.92	53.95
2	39.285	74.134	50.104	50.08	46.05
**Total:**		**148.035**	**108.808**	**100.00**	**100.00**

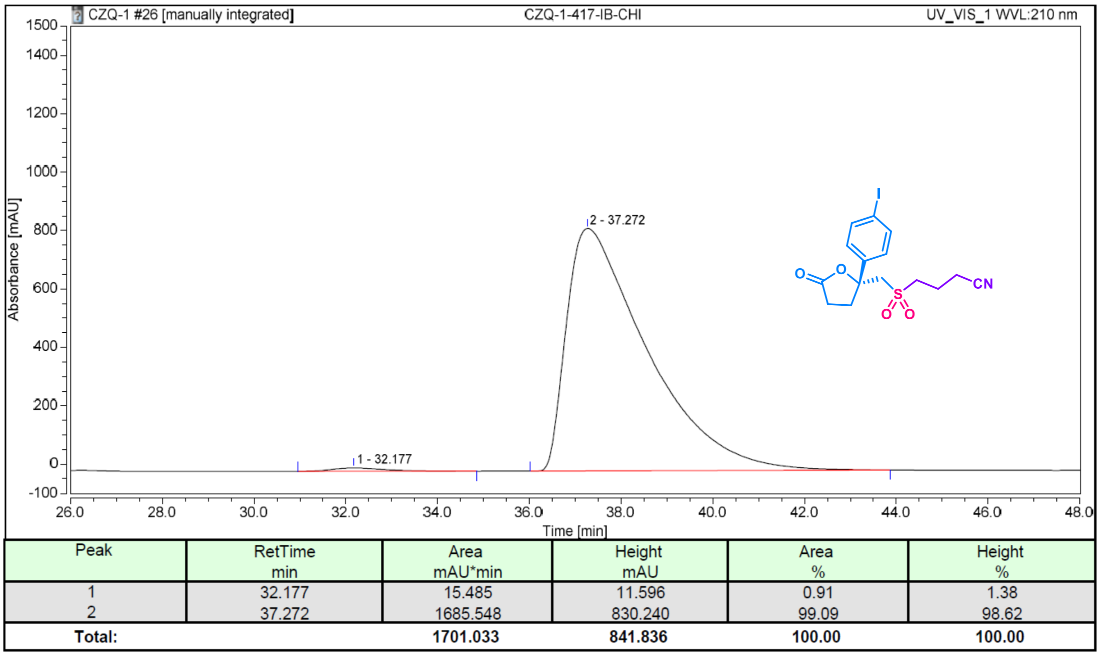

Peak	RetTime min	Area mAU*min	Height mAU	Area %	Height %
1	32.177	15.485	11.596	0.91	1.38
2	37.272	1685.548	830.240	99.09	98.62
**Total:**		**1701.033**	**841.836**	**100.00**	**100.00**

(*R*)-4-(((4,4-dimethyl-5-oxo-2-phenyltetrahydrofuran-2-yl)methyl)sulfonyl) butanenitrile

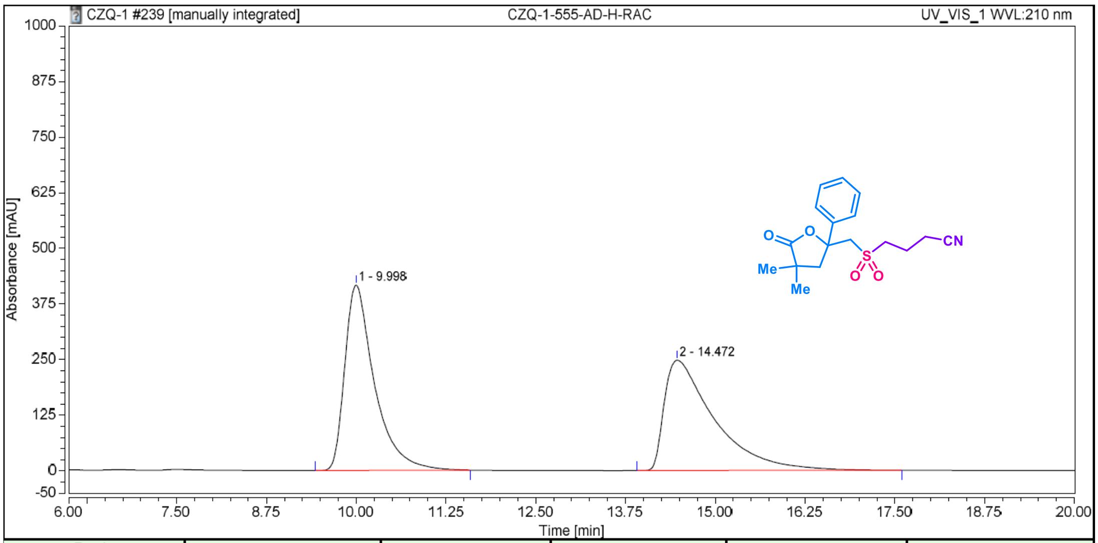

Peak	RetTime min	Area mAU*min	Height mAU	Area %	Height %
1	9.998	199.071	416.968	49.92	62.70
2	14.472	199.688	248.005	50.08	37.30
**Total:**		**398.759**	**664.973**	**100.00**	**100.00**

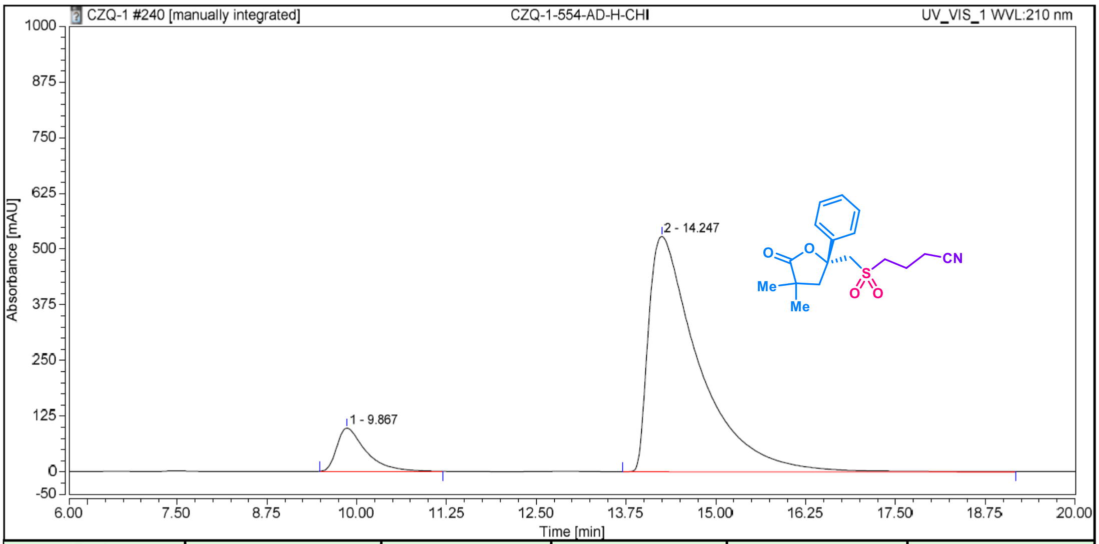

Peak	RetTime min	Area mAU*min	Height mAU	Area %	Height %
1	9.867	45.157	97.118	9.45	15.52
2	14.247	432.846	528.466	90.55	84.48
**Total:**		**478.003**	**625.585**	**100.00**	**100.00**

(*R*)-4-(((5-oxo-2-(phenylethynyl)tetrahydrofuran-2-yl)methyl)sulfonyl)butanenitrile

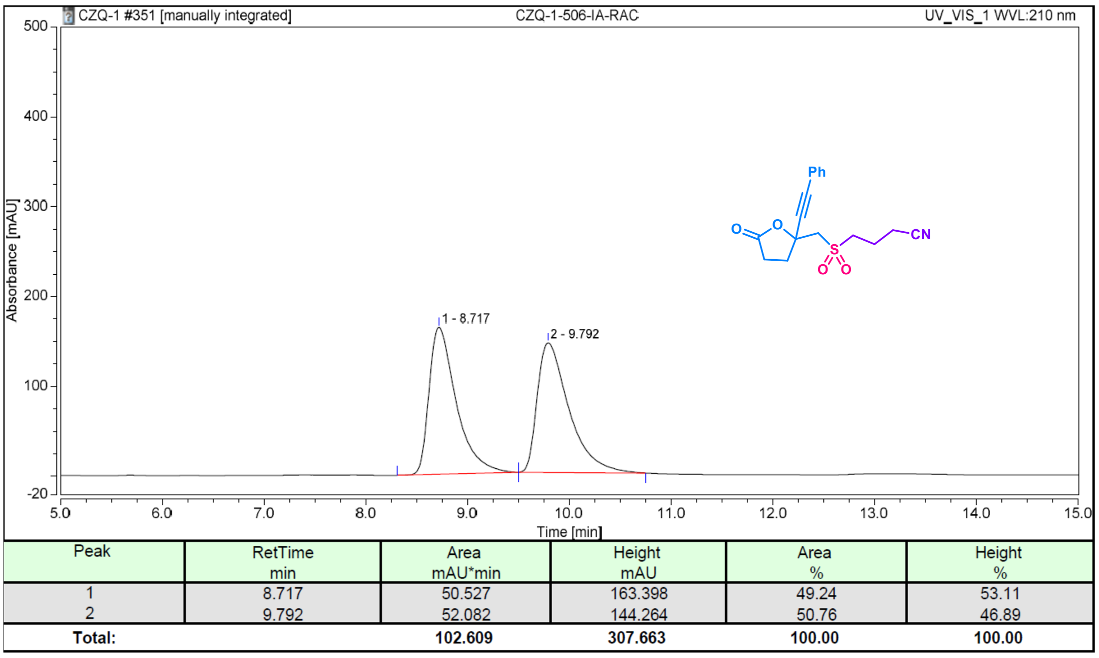

Peak	RetTime min	Area mAU*min	Height mAU	Area %	Height %
1	8.717	50.527	163.398	49.24	53.11
2	9.792	52.082	144.264	50.76	46.89
**Total:**		**102.609**	**307.663**	**100.00**	**100.00**

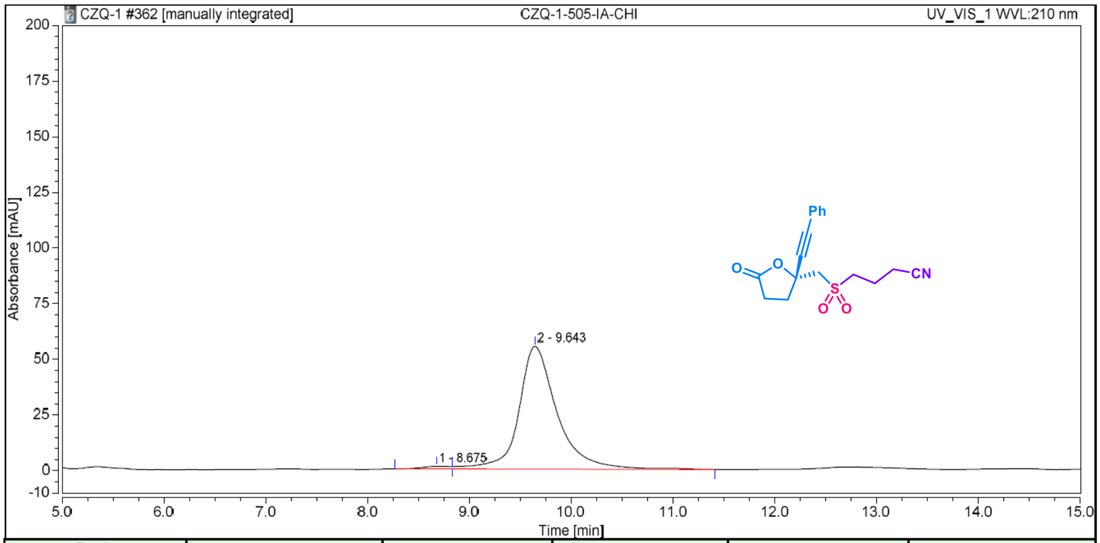

Peak	RetTime min	Area mAU*min	Height mAU	Area %	Height %
1	8.675	0.387	1.200	1.57	2.13
2	9.643	24.237	55.205	98.43	97.87
**Total:**		**24.624**	**56.405**	**100.00**	**100.00**

tert-butyl (R)-3-(cyanomethyl)-3-((((5-oxo-2-phenyltetrahydrofuran-2-yl)methyl) sulfonyl)methyl) azetidine-1-carboxylate

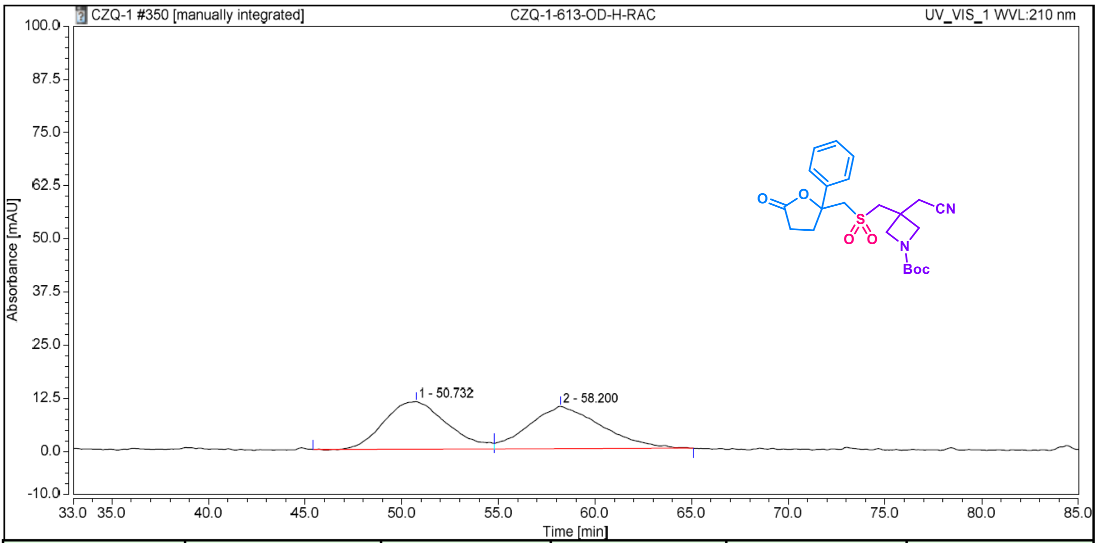

Peak	RetTime min	Area mAU*min	Height mAU	Area %	Height %
1	50.732	42.117	11.155	49.51	52.96
2	58.200	42.942	9.906	50.49	47.04
**Total:**		**85.059**	**21.061**	**100.00**	**100.00**

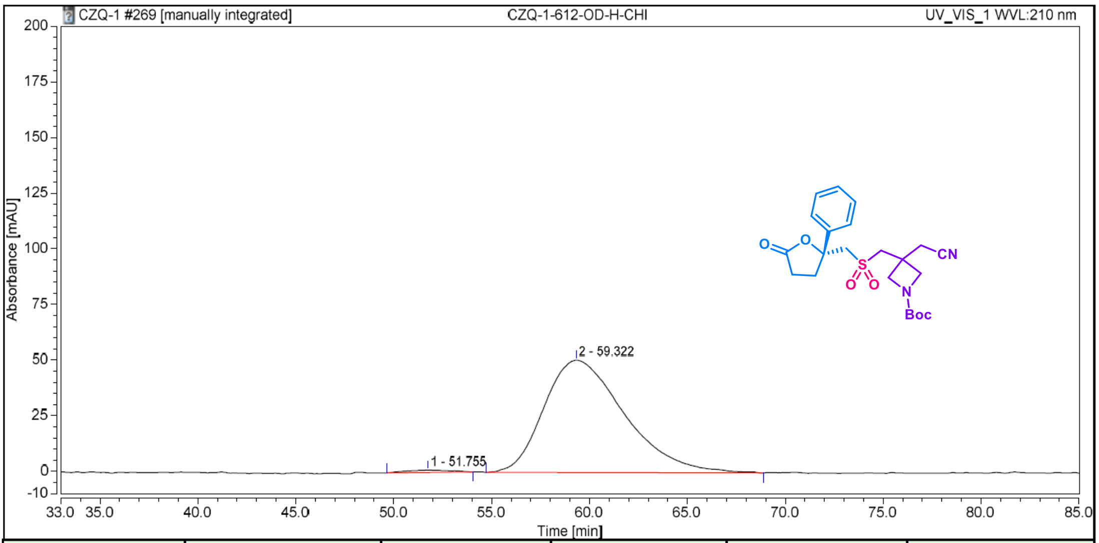

Peak	RetTime min	Area mAU*min	Height mAU	Area %	Height %
1	51.755	2.896	1.125	1.19	2.18
2	59.322	240.474	50.461	98.81	97.82
**Total:**		**243.370**	**51.586**	**100.00**	**100.00**

tert-butyl (_R_)-4-(cyanomethyl)-4-((((5-oxo-2-phenyltetrahydrofuran-2-yl)methyl) sulfonyl) methyl) piperidine-1-carboxylate

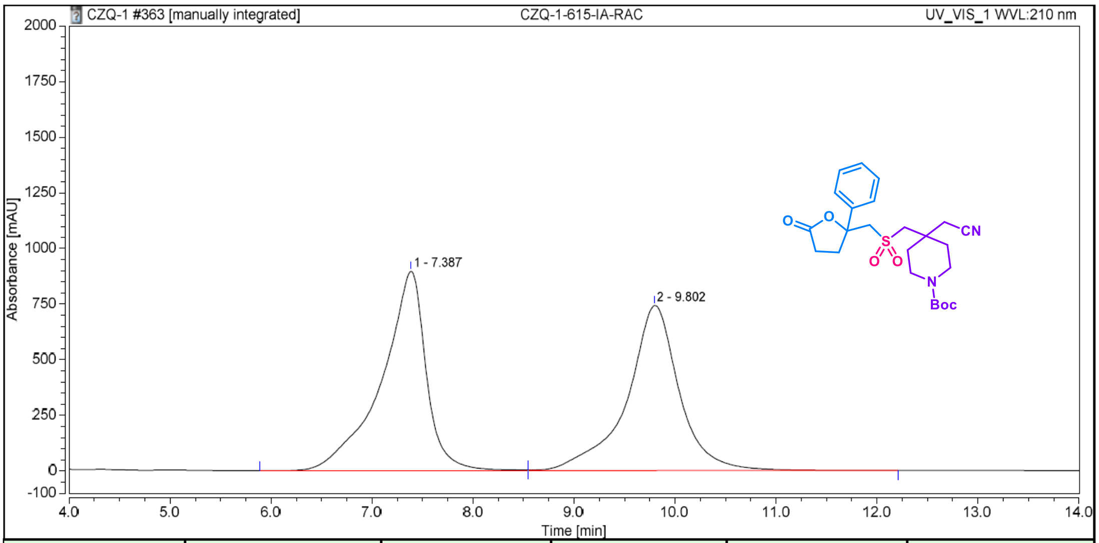

Peak	RetTime min	Area mAU*min	Height mAU	Area %	Height %
1	7.387	459.293	896.761	50.76	54.72
2	9.802	445.610	742.185	49.24	45.28
**Total:**		**904.904**	**1638.946**	**100.00**	**100.00**

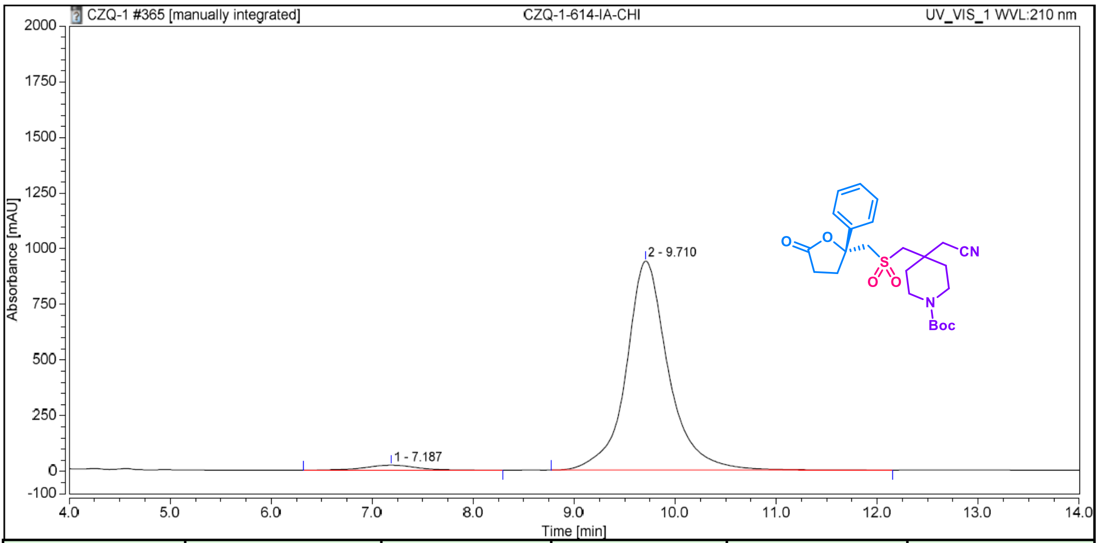

Peak	RetTime min	Area mAU*min	Height mAU	Area %	Height %
1	7.187	13.249	22.132	2.83	2.30
2	9.710	455.713	939.032	97.17	97.70
**Total:**		**468.962**	**961.164**	**100.00**	**100.00**

(*R*)-5-phenyl-5-(tosylmethyl)dihydrofuran-2(3*H*)-one

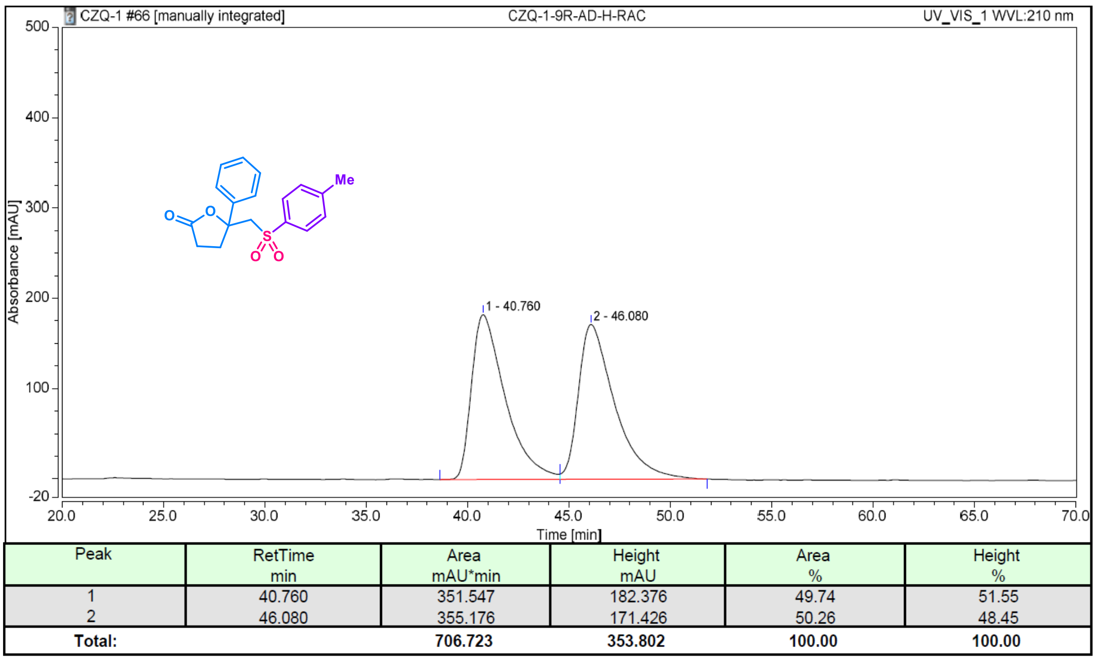

Peak	RetTime min	Area mAU*min	Height mAU	Area %	Height %
1	40.760	351.547	182.376	49.74	51.55
2	46.080	355.176	171.426	50.26	48.45
**Total:**		**706.723**	**353.802**	**100.00**	**100.00**

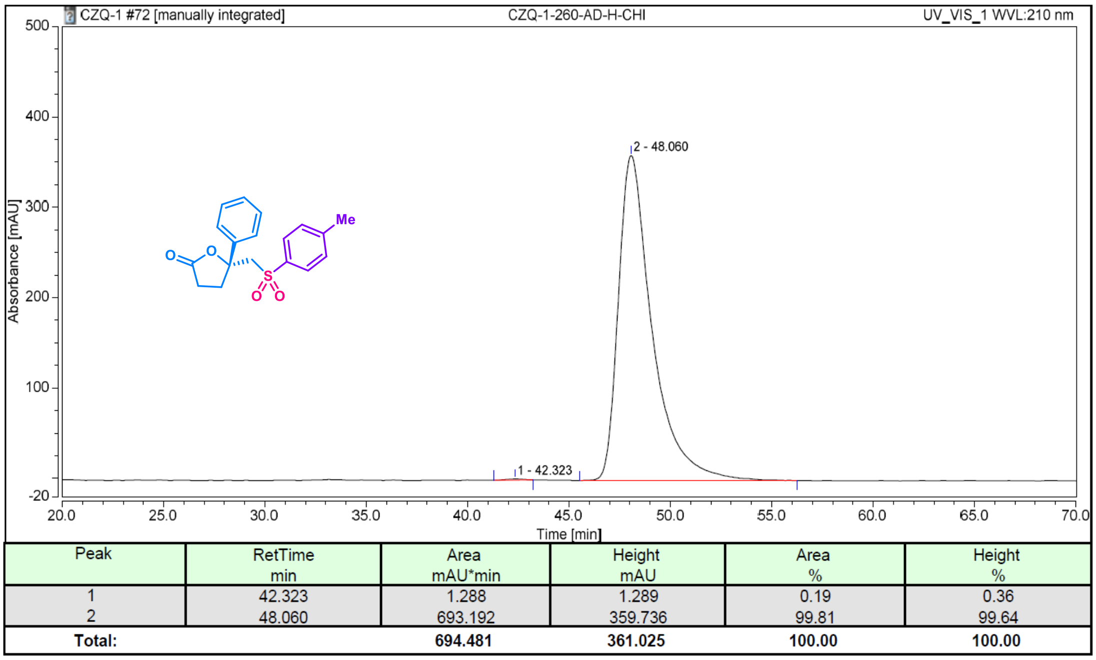

Peak	RetTime min	Area mAU*min	Height mAU	Area %	Height %
1	42.323	1.288	1.289	0.19	0.36
2	48.060	693.192	359.736	99.81	99.64
**Total:**		**694.481**	**361.025**	**100.00**	**100.00**

(*R*)-5-([1,1'-biphenyl]-4-yl)-5-(tosylmethyl)dihydrofuran-2(3*H*)-one

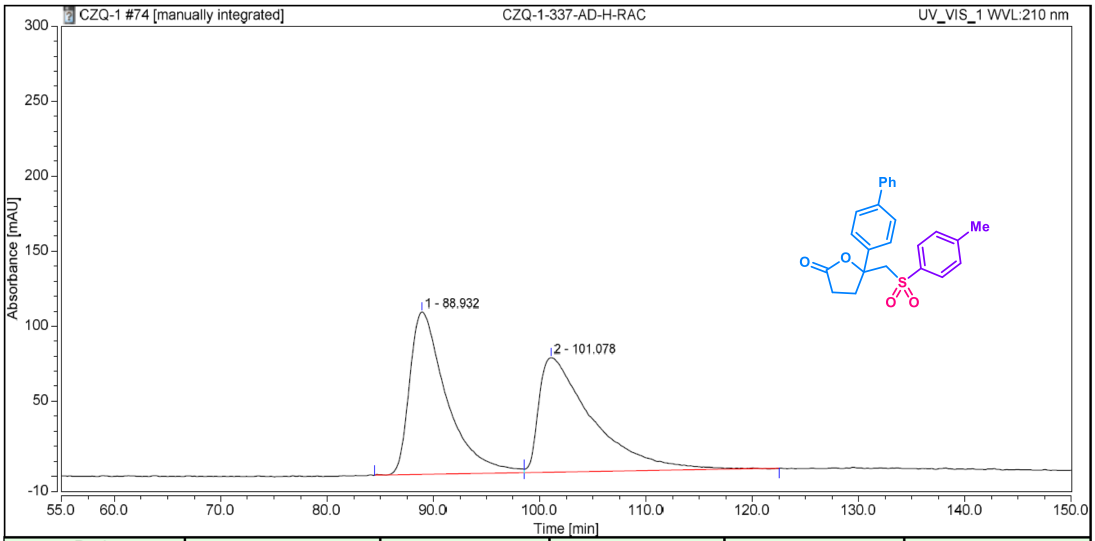

Peak	RetTime min	Area mAU*min	Height mAU	Area %	Height %
1	88.932	436.260	108.283	49.54	58.67
2	101.078	444.281	76.292	50.46	41.33
**Total:**		**880.542**	**184.575**	**100.00**	**100.00**

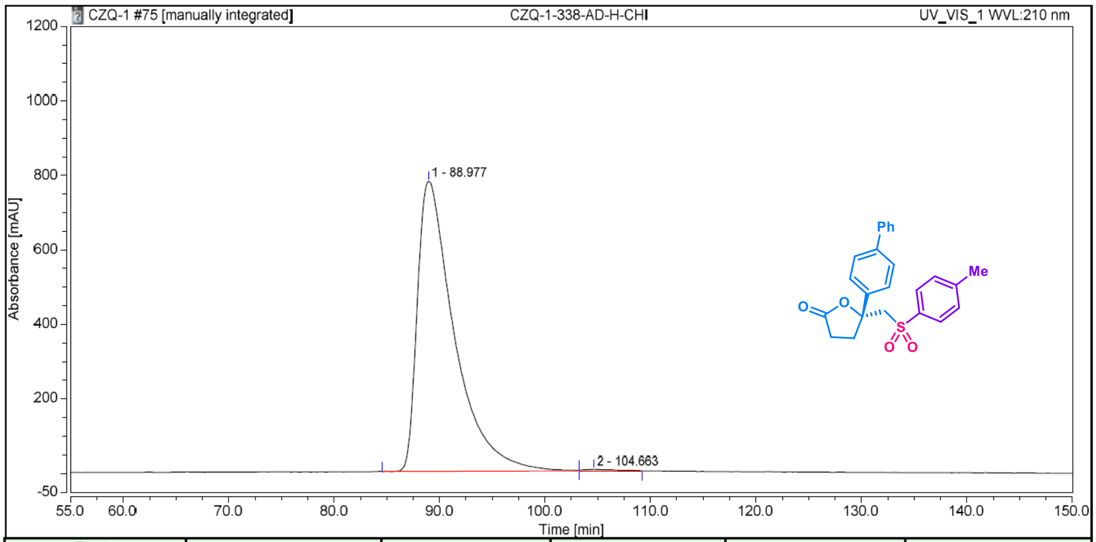

Peak	RetTime min	Area mAU*min	Height mAU	Area %	Height %
1	88.977	3151.936	778.718	99.57	99.47
2	104.663	13.538	4.144	0.43	0.53
**Total:**		**3165.474**	**782.862**	**100.00**	**100.00**

(*R*)-5-(naphthalen-2-yl)-5-(tosylmethyl)dihydrofuran-2(3*H*)-one

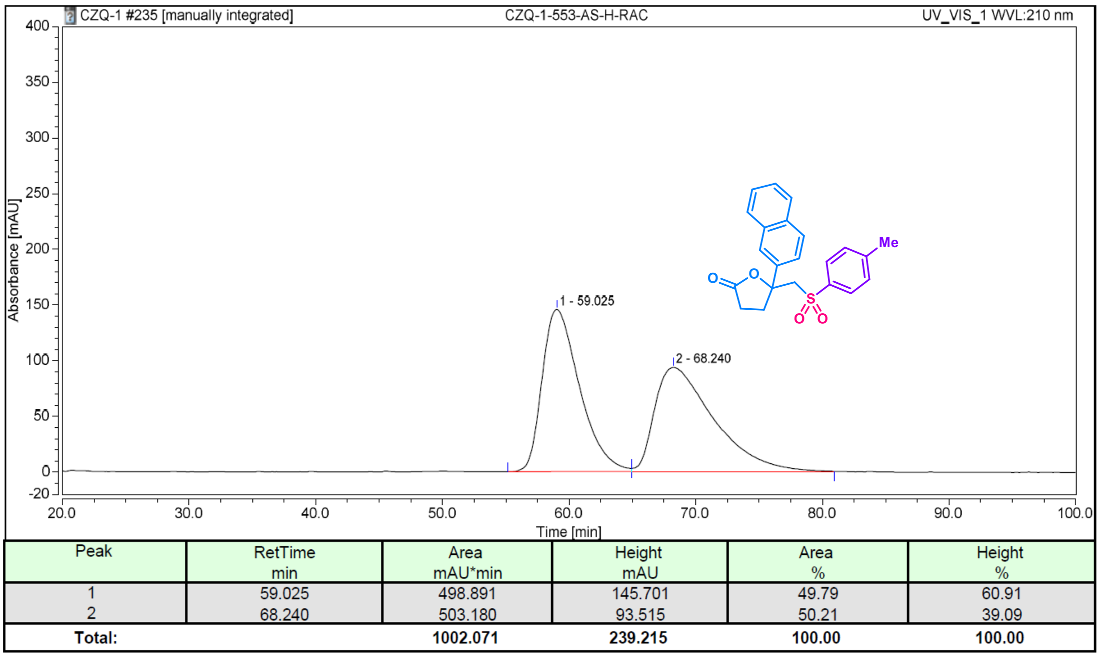

Peak	RetTime min	Area mAU*min	Height mAU	Area %	Height %
1	59.025	498.891	145.701	49.79	60.91
2	68.240	503.180	93.515	50.21	39.09
**Total:**		**1002.071**	**239.215**	**100.00**	**100.00**

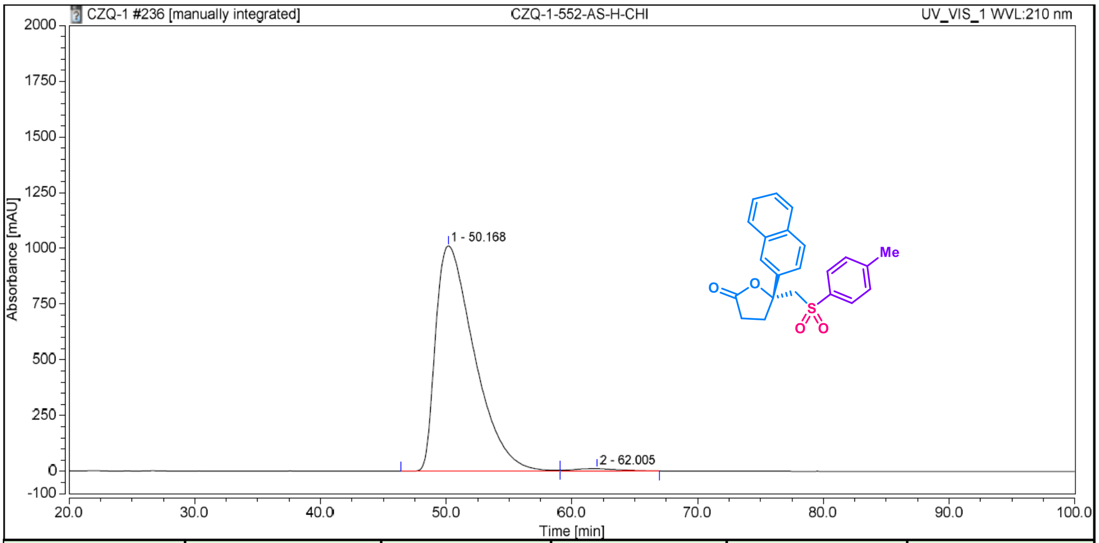

Peak	RetTime min	Area mAU*min	Height mAU	Area %	Height %
1	50.168	3612.813	1011.786	98.80	98.98
2	62.005	43.925	10.458	1.20	1.02
**Total:**		**3656.738**	**1022.244**	**100.00**	**100.00**

(*R*)-5-(pyren-1-yl)-5-(tosylmethyl)dihydrofuran-2(3*H*)-one

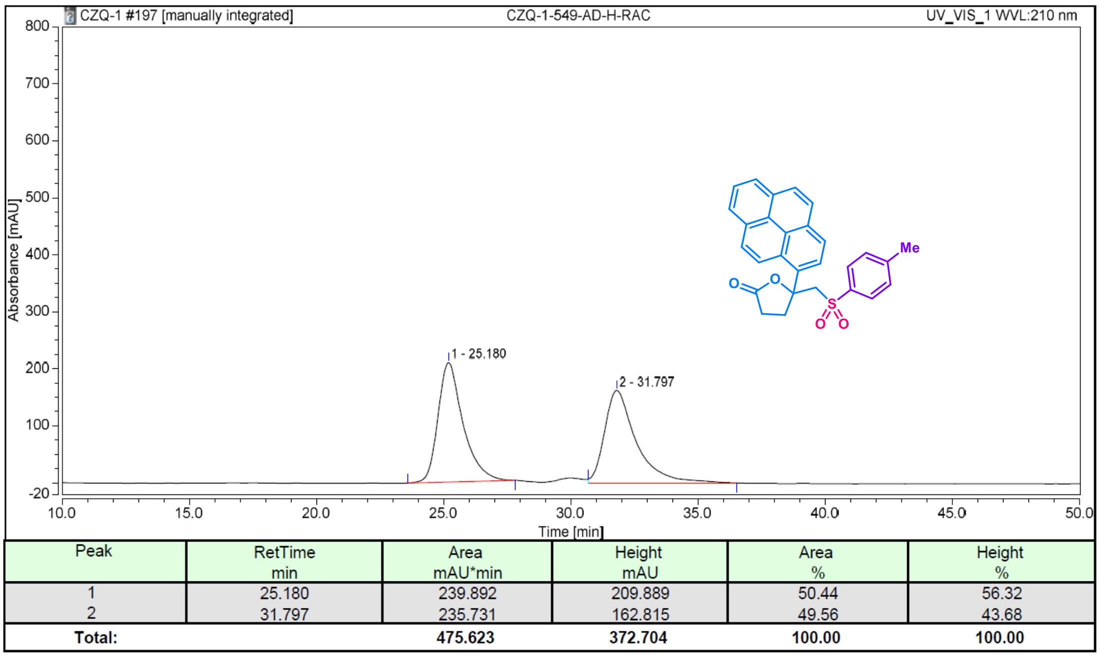

Peak	RetTime min	Area mAU*min	Height mAU	Area %	Height %
1	25.180	239.892	209.889	50.44	56.32
2	31.797	235.731	162.815	49.56	43.68
**Total:**		**475.623**	**372.704**	**100.00**	**100.00**

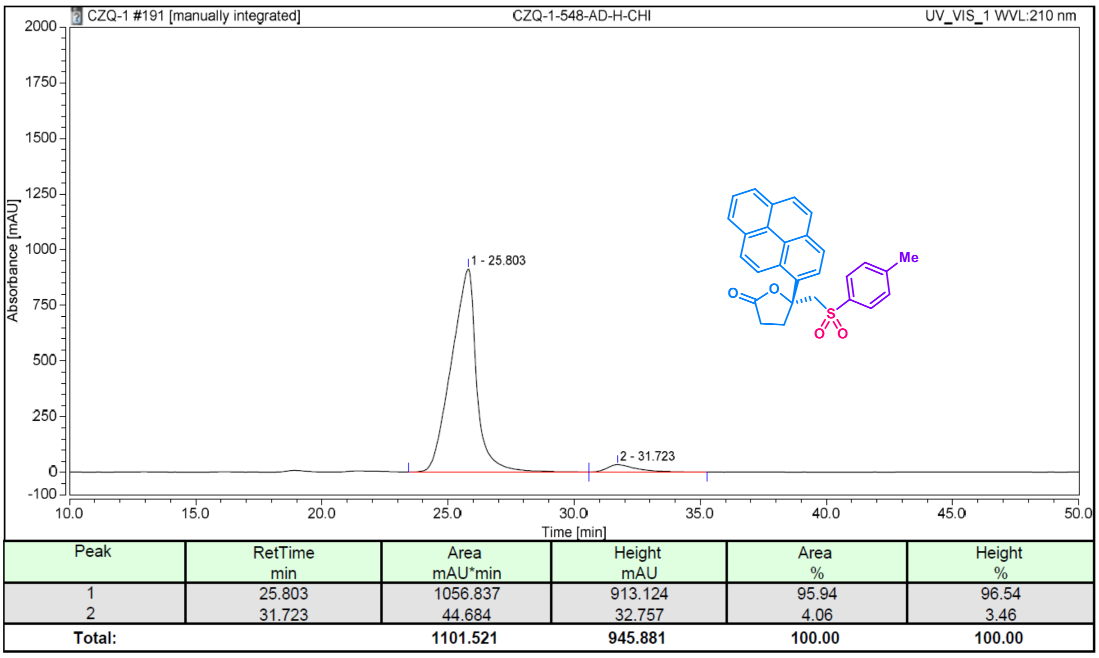

Peak	RetTime min	Area mAU*min	Height mAU	Area %	Height %
1	25.803	1056.837	913.124	95.94	96.54
2	31.723	44.684	32.757	4.06	3.46
**Total:**		**1101.521**	**945.881**	**100.00**	**100.00**

(*R*)-5-(o-tolyl)-5-(tosylmethyl)dihydrofuran-2(3*H*)-one

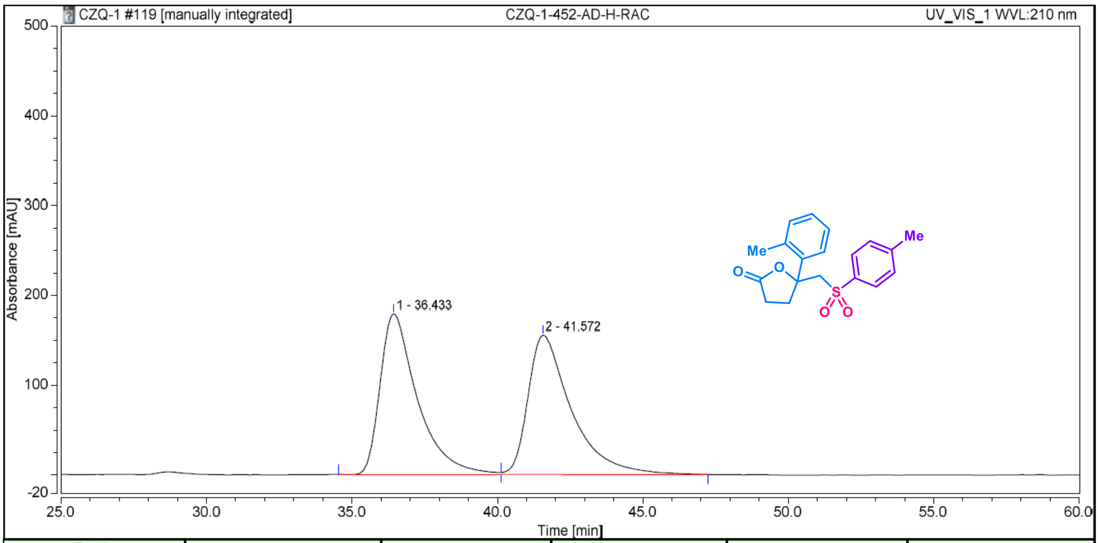

Peak	RetTime min	Area mAU*min	Height mAU	Area %	Height %
1	36.433	264.346	178.763	49.73	53.57
2	41.572	267.226	154.962	50.27	46.43
**Total:**		**531.572**	**333.725**	**100.00**	**100.00**

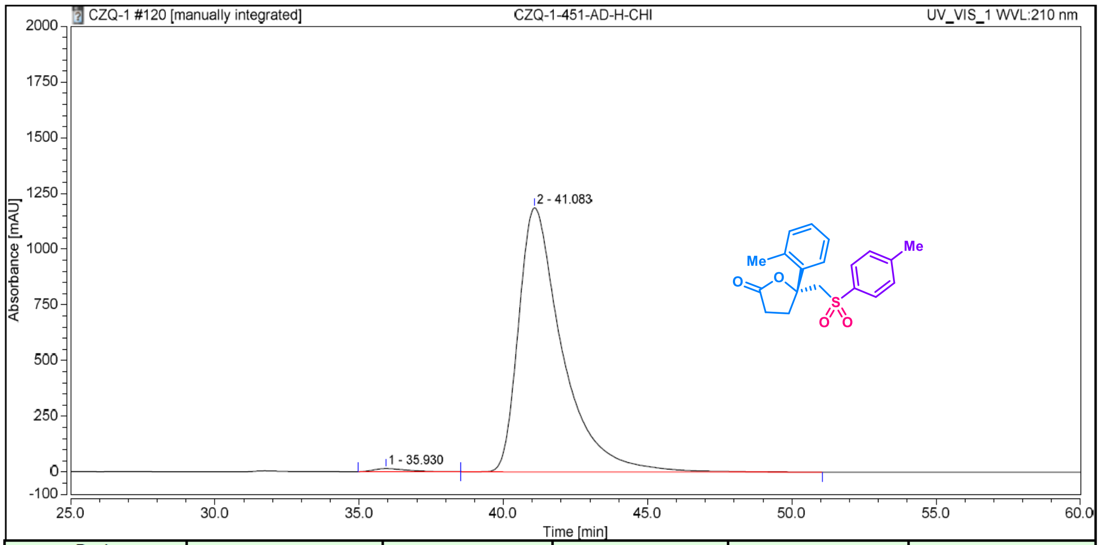

Peak	RetTime min	Area mAU*min	Height mAU	Area %	Height %
1	35.930	20.478	14.462	0.97	1.20
2	41.083	2086.437	1185.984	99.03	98.80
**Total:**		**2106.916**	**1200.446**	**100.00**	**100.00**

(*R*)-5-(m-tolyl)-5-(tosylmethyl)dihydrofuran-2(3*H*)-one

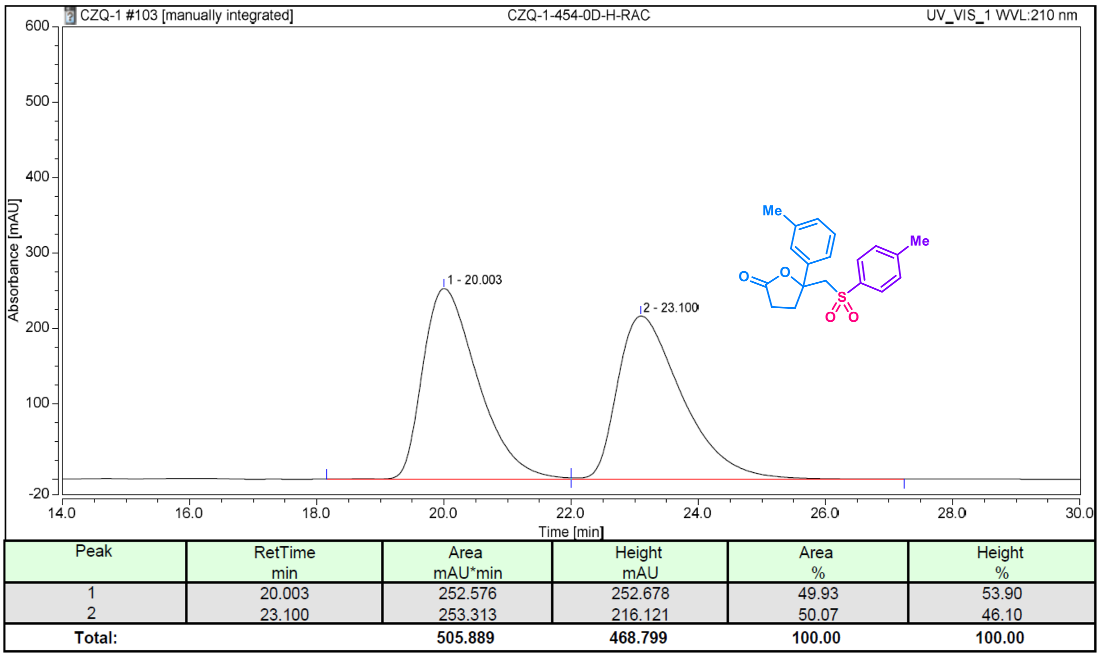

Peak	RetTime min	Area mAU*min	Height mAU	Area %	Height %
1	20.003	252.576	252.678	49.93	53.90
2	23.100	253.313	216.121	50.07	46.10
**Total:**		**505.889**	**468.799**	**100.00**	**100.00**

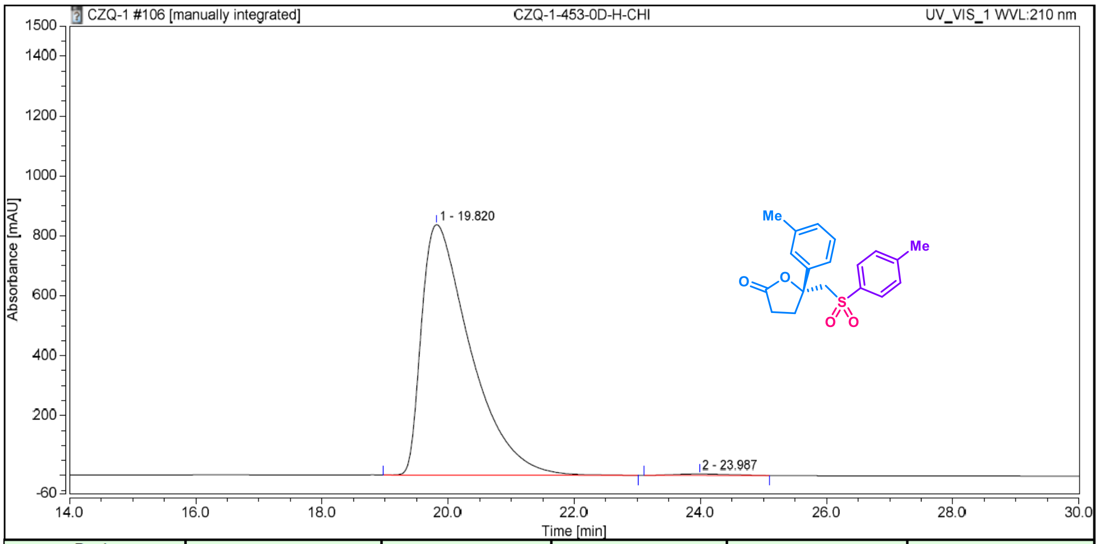

Peak	RetTime min	Area mAU*min	Height mAU	Area %	Height %
1	19.820	751.523	836.370	99.37	99.43
2	23.987	4.760	4.772	0.63	0.57
**Total:**		**756.283**	**841.142**	**100.00**	**100.00**

(*R*)-5-(4-(*tert*-butyl)phenyl)-5-(tosylmethyl)dihydrofuran-2(3*H*)-one

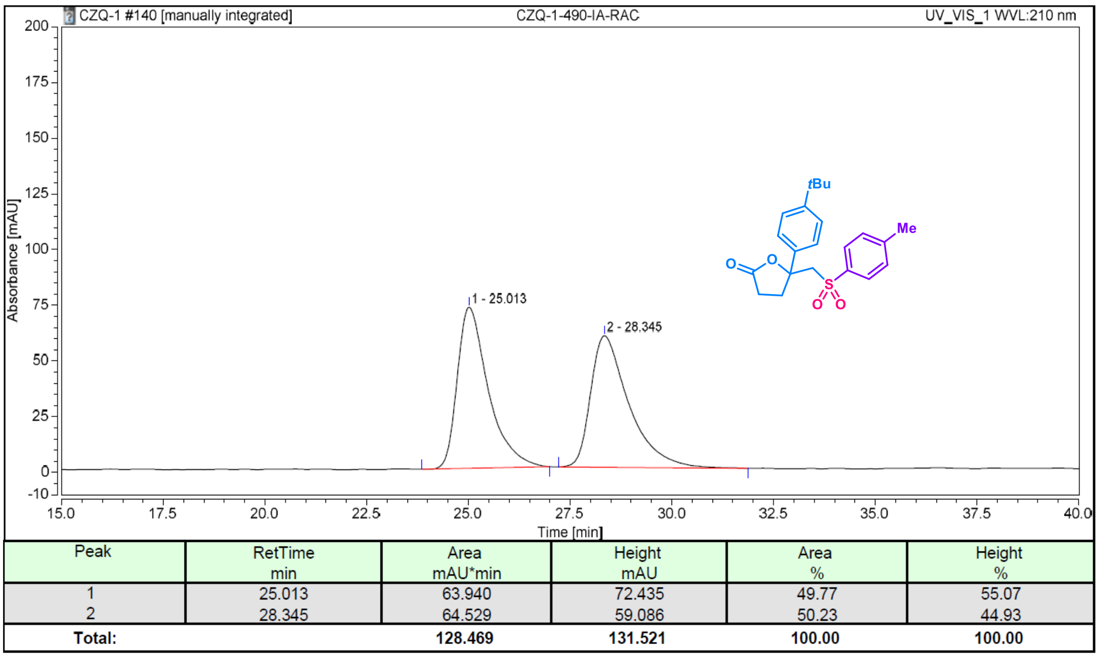

Peak	RetTime min	Area mAU*min	Height mAU	Area %	Height %
1	25.013	63.940	72.435	49.77	55.07
2	28.345	64.529	59.086	50.23	44.93
**Total:**		**128.469**	**131.521**	**100.00**	**100.00**

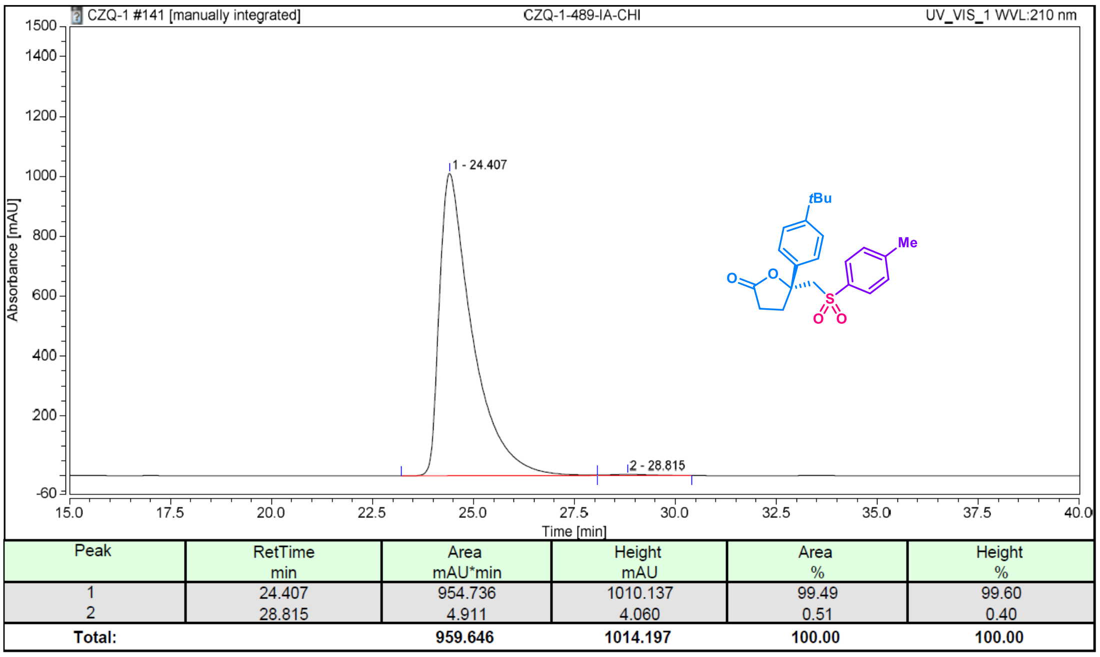

Peak	RetTime min	Area mAU*min	Height mAU	Area %	Height %
1	24.407	954.736	1010.137	99.49	99.60
2	28.815	4.911	4.060	0.51	0.40
**Total:**		**959.646**	**1014.197**	**100.00**	**100.00**

(*R*)-5-(3-methoxyphenyl)-5-(tosylmethyl)dihydrofuran-2(3*H*)-one

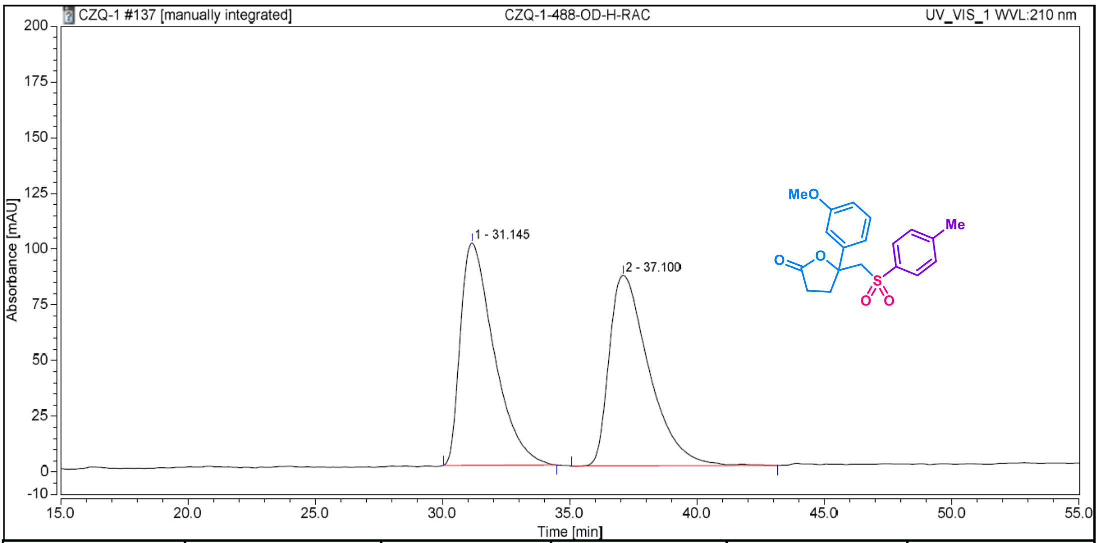

Peak	RetTime min	Area mAU*min	Height mAU	Area %	Height %
1	31.145	152.675	99.829	49.54	53.87
2	37.100	155.541	85.488	50.46	46.13
**Total:**		**308.216**	**185.317**	**100.00**	**100.00**

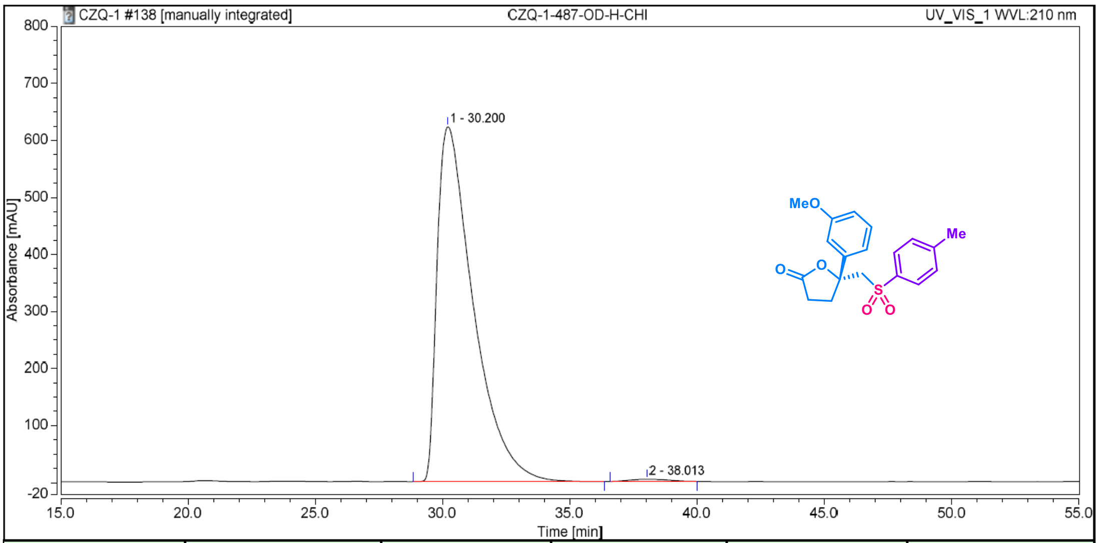

Peak	RetTime min	Area mAU*min	Height mAU	Area %	Height %
1	30.200	1024.825	622.597	99.32	99.37
2	38.013	7.024	3.976	0.68	0.63
**Total:**		**1031.849**	**626.573**	**100.00**	**100.00**

(*R*)-5-(3-fluorophenyl)-5-(tosylmethyl)dihydrofuran-2(3*H*)-one

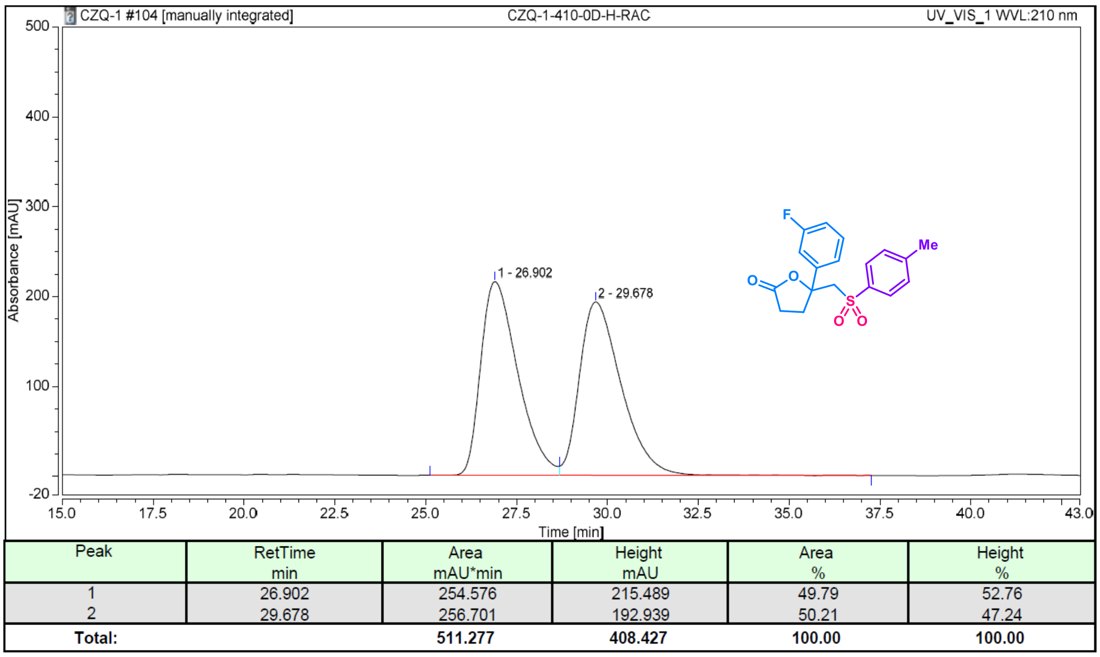

Peak	RetTime min	Area mAU*min	Height mAU	Area %	Height %
1	26.902	254.576	215.489	49.79	52.76
2	29.678	256.701	192.939	50.21	47.24
**Total:**		**511.277**	**408.427**	**100.00**	**100.00**

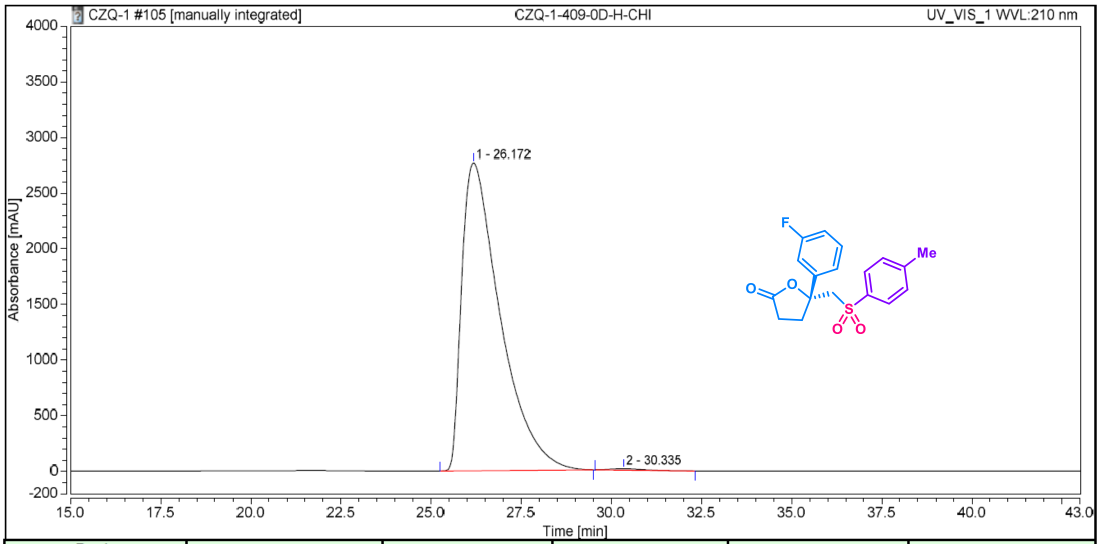

Peak	RetTime min	Area mAU*min	Height mAU	Area %	Height %
1	26.172	3415.872	2767.137	99.66	99.57
2	30.335	11.811	11.921	0.34	0.43
**Total:**		**3427.683**	**2779.058**	**100.00**	**100.00**

(*R*)-5-(4-fluorophenyl)-5-(tosylmethyl)dihydrofuran-2(3*H*)-one

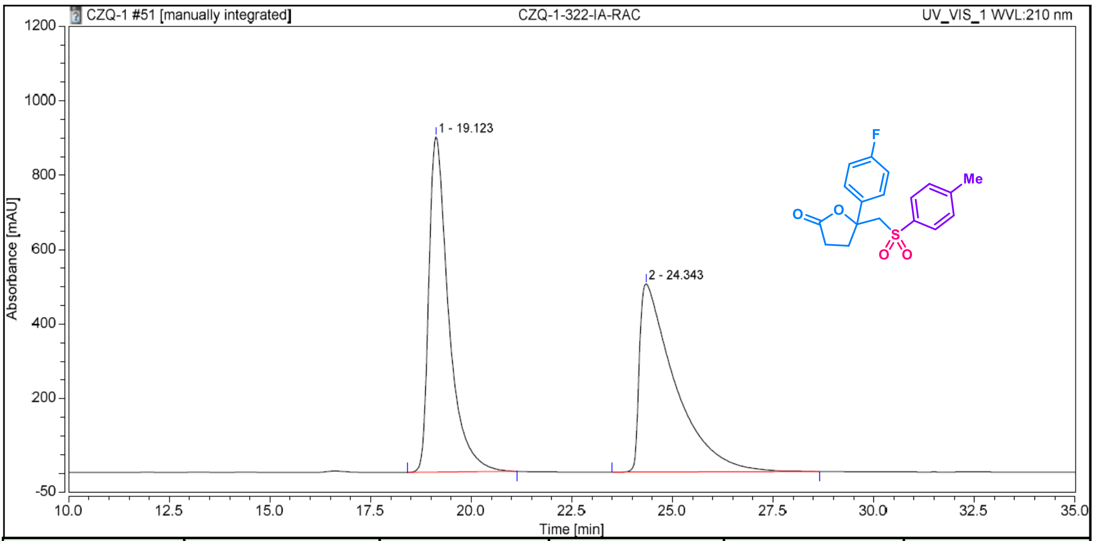

Peak	RetTime min	Area mAU*min	Height mAU	Area %	Height %
1	19.123	513.822	900.545	49.79	64.04
2	24.343	518.198	505.593	50.21	35.96
**Total:**		**1032.020**	**1406.138**	**100.00**	**100.00**

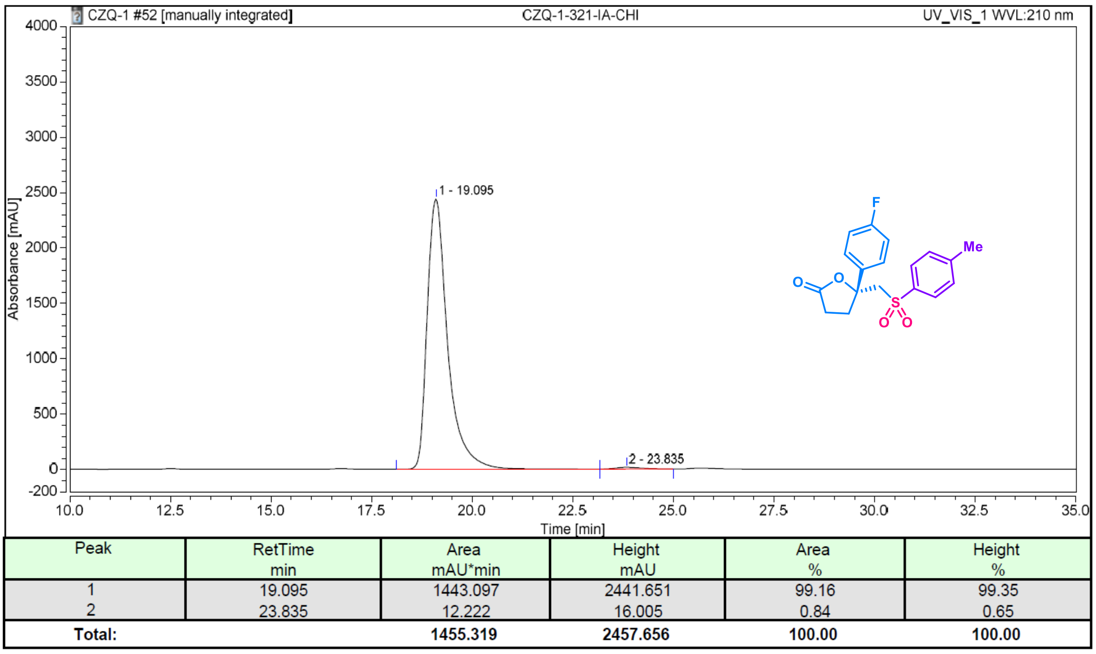

Peak	RetTime min	Area mAU*min	Height mAU	Area %	Height %
1	19.095	1443.097	2441.651	99.16	99.35
2	23.835	12.222	16.005	0.84	0.65
**Total:**		**1455.319**	**2457.656**	**100.00**	**100.00**

(*R*)-5-(4-iodophenyl)-5-(tosylmethyl)dihydrofuran-2(3*H*)-one

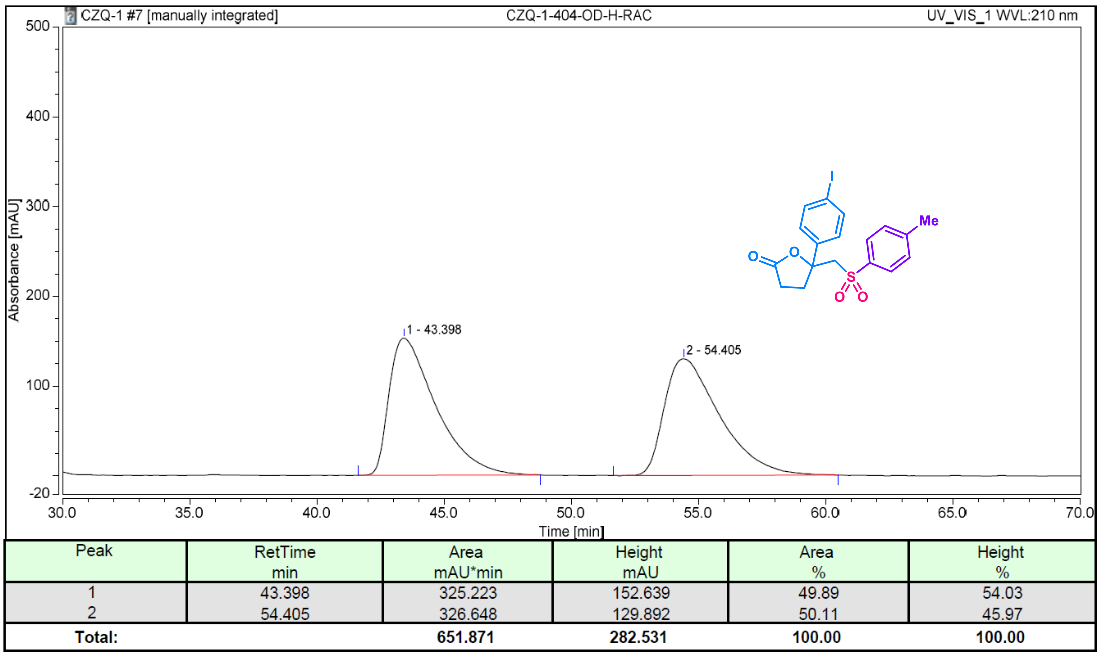

Peak	RetTime<br>min	Area<br>mAU*min	Height<br>mAU	Area<br>%	Height<br>%
1	43.398	325.223	152.639	49.89	54.03
2	54.405	326.648	129.892	50.11	45.97
**Total:**		**651.871**	**282.531**	**100.00**	**100.00**

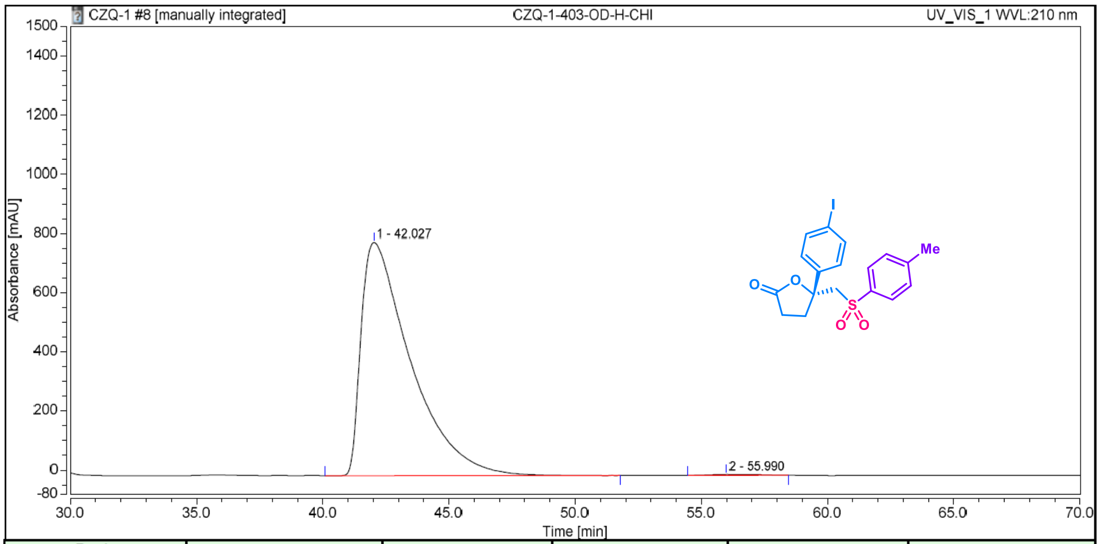

Peak	RetTime<br>min	Area<br>mAU*min	Height<br>mAU	Area<br>%	Height<br>%
1	42.027	1792.727	788.294	99.63	99.61
2	55.990	6.609	3.051	0.37	0.39
**Total:**		**1799.336**	**791.345**	**100.00**	**100.00**

(*S*)-5-(thiophen-2-yl)-5-(tosylmethyl)dihydrofuran-2(3*H*)-one

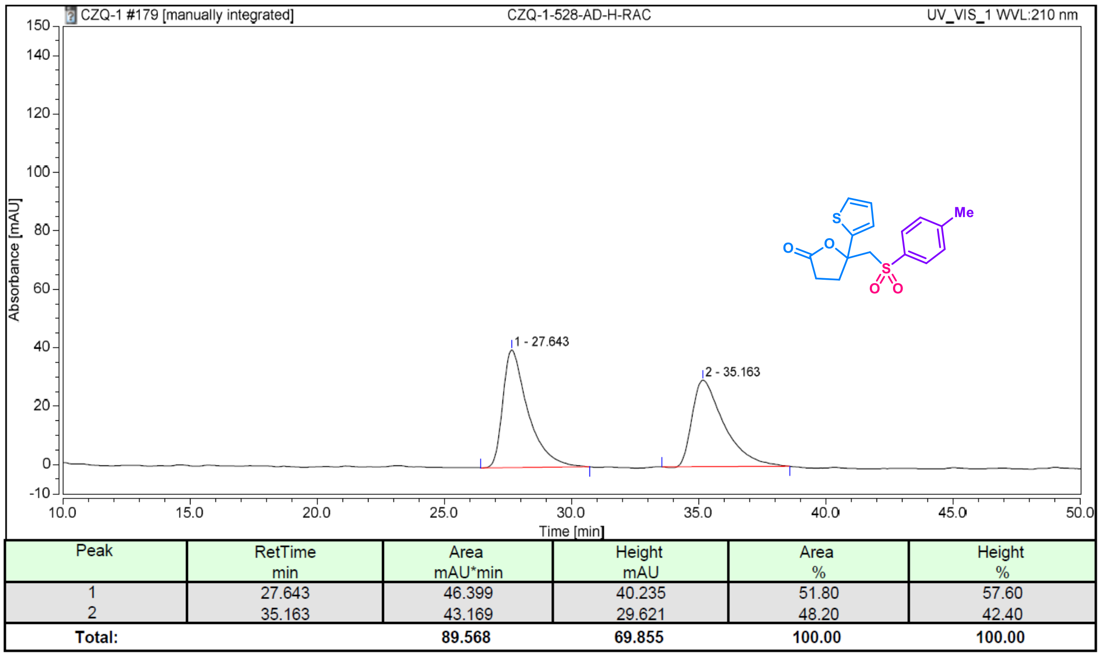

Peak	RetTime min	Area mAU*min	Height mAU	Area %	Height %
1	27.643	46.399	40.235	51.80	57.60
2	35.163	43.169	29.621	48.20	42.40
**Total:**		**89.568**	**69.855**	**100.00**	**100.00**

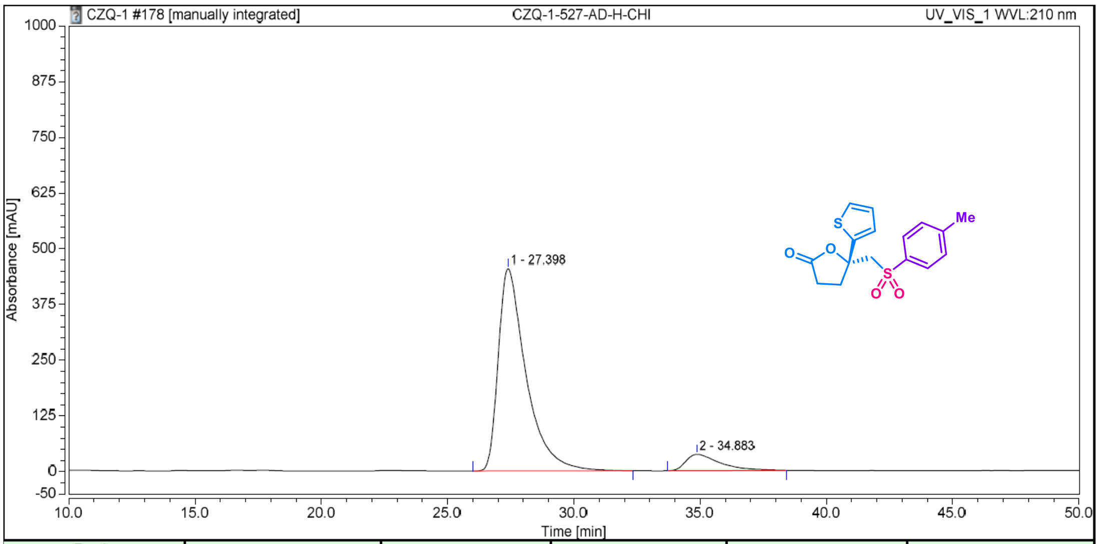

Peak	RetTime min	Area mAU*min	Height mAU	Area %	Height %
1	27.398	578.212	454.426	91.04	92.54
2	34.883	56.919	36.610	8.96	7.46
**Total:**		**635.132**	**491.037**	**100.00**	**100.00**

(*R*)-3,3-dimethyl-5-phenyl-5-(tosylmethyl)dihydrofuran-2(3*H*)-one

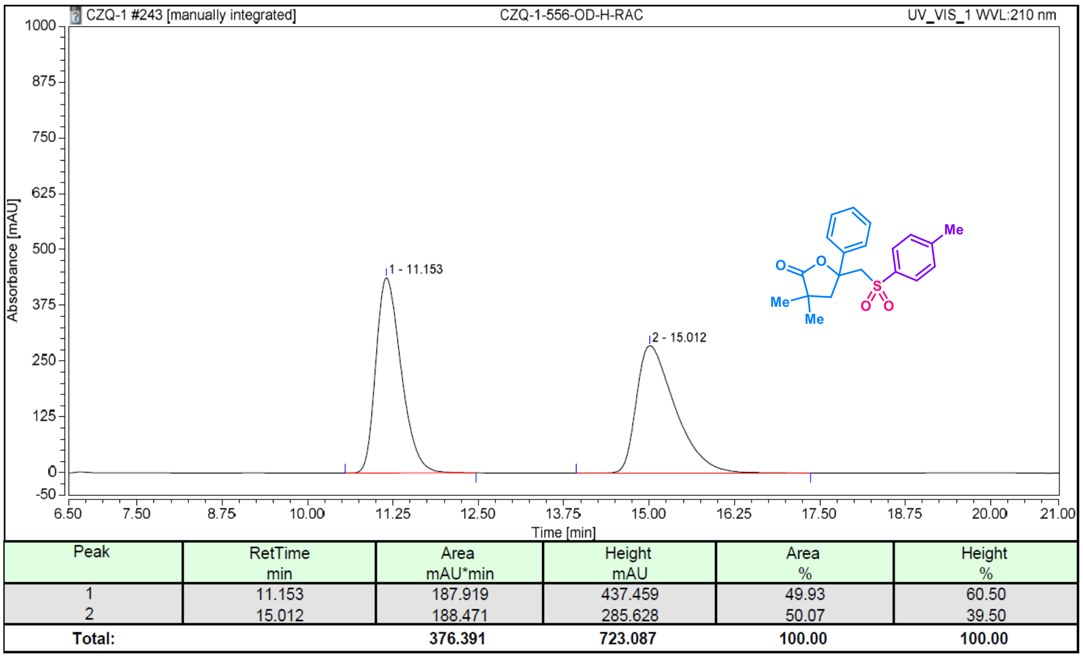

Peak	RetTime min	Area mAU*min	Height mAU	Area %	Height %
1	11.153	187.919	437.459	49.93	60.50
2	15.012	188.471	285.628	50.07	39.50
**Total:**		**376.391**	**723.087**	**100.00**	**100.00**

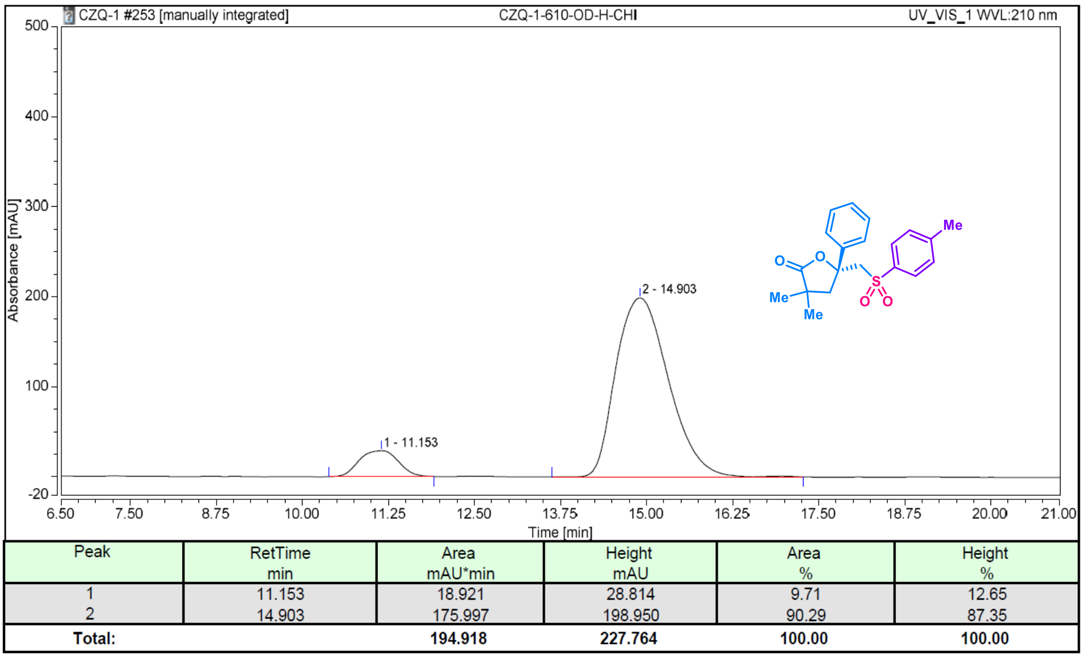

Peak	RetTime min	Area mAU*min	Height mAU	Area %	Height %
1	11.153	18.921	28.814	9.71	12.65
2	14.903	175.997	198.950	90.29	87.35
**Total:**		**194.918**	**227.764**	**100.00**	**100.00**

(*R*)-5-(phenylethynyl)-5-(tosylmethyl)dihydrofuran-2(3*H*)-one

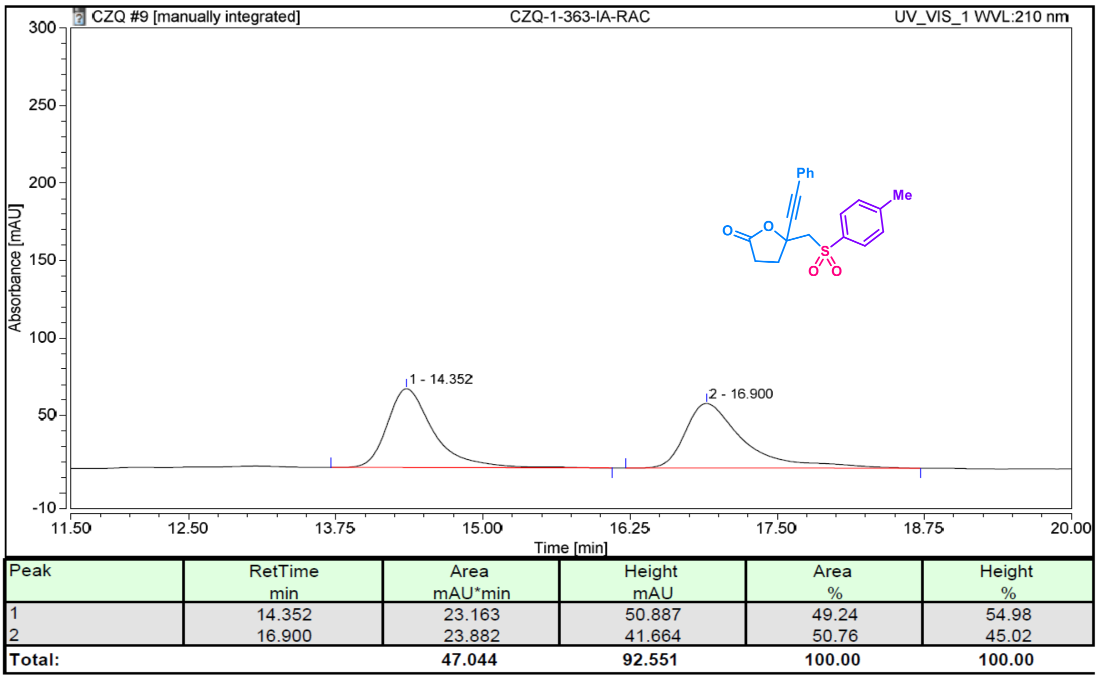

Peak	RetTime min	Area mAU*min	Height mAU	Area %	Height %
1	14.352	23.163	50.887	49.24	54.98
2	16.900	23.882	41.664	50.76	45.02
**Total:**		**47.044**	**92.551**	**100.00**	**100.00**

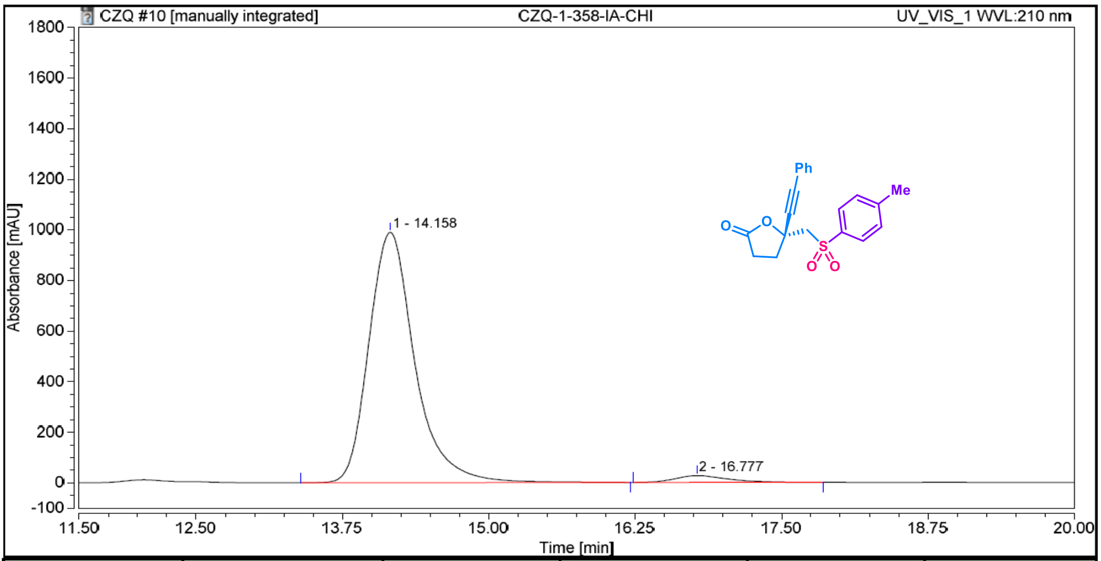

Peak	RetTime min	Area mAU*min	Height mAU	Area %	Height %
1	14.158	449.063	989.214	97.06	97.41
2	16.777	13.614	26.299	2.94	2.59
**Total:**		**462.677**	**1015.513**	**100.00**	**100.00**

(*R*)-5-benzoyl-5-(tosylmethyl)dihydrofuran-2(3*H*)-one

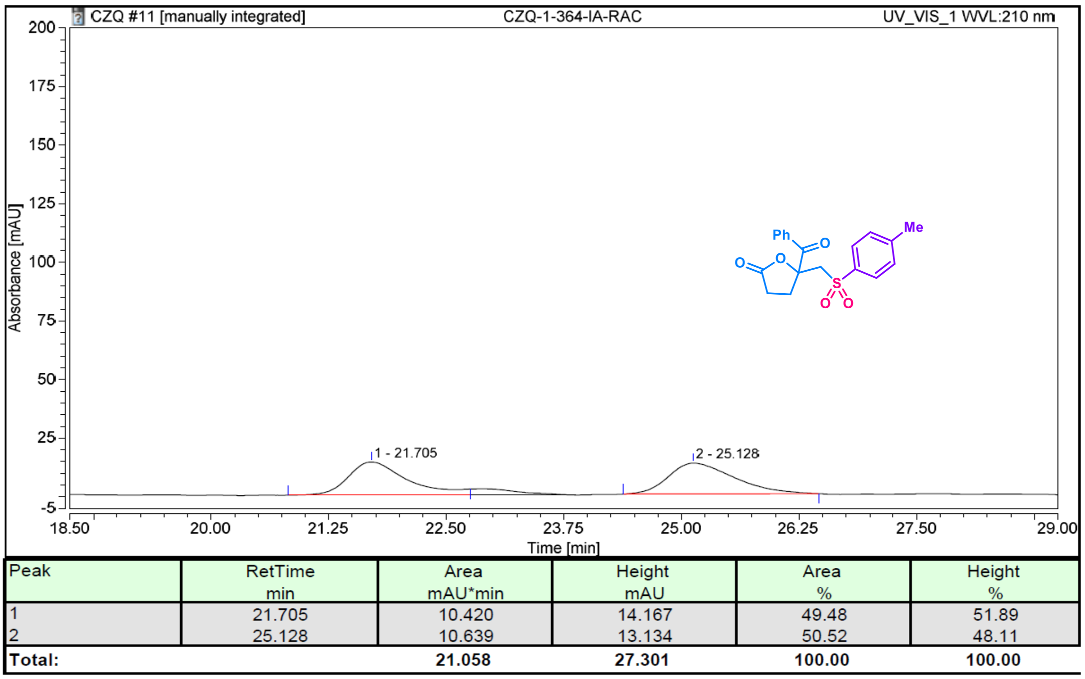

Peak	RetTime min	Area mAU*min	Height mAU	Area %	Height %
1	21.705	10.420	14.167	49.48	51.89
2	25.128	10.639	13.134	50.52	48.11
**Total:**		**21.058**	**27.301**	**100.00**	**100.00**

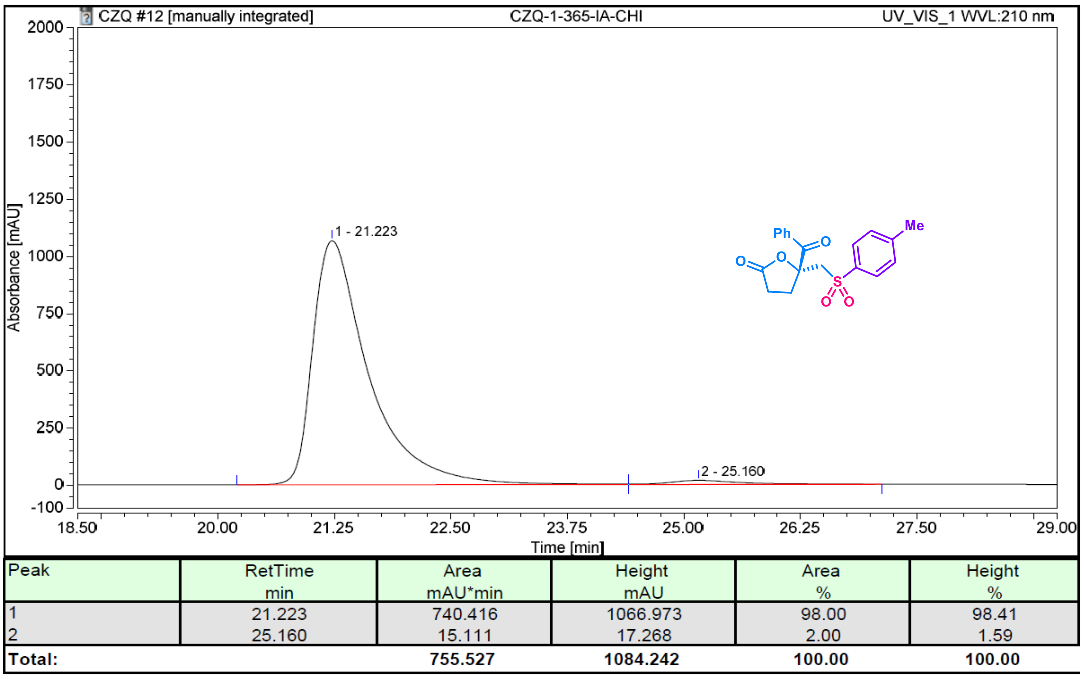

Peak	RetTime min	Area mAU*min	Height mAU	Area %	Height %
1	21.223	740.416	1066.973	98.00	98.41
2	25.160	15.111	17.268	2.00	1.59
**Total:**		**755.527**	**1084.242**	**100.00**	**100.00**

(5*R*)-5-(1-([1,1'-biphenyl]-4-ylsulfonyl)ethyl)-5-phenyldihydrofuran-2(3*H*)-one

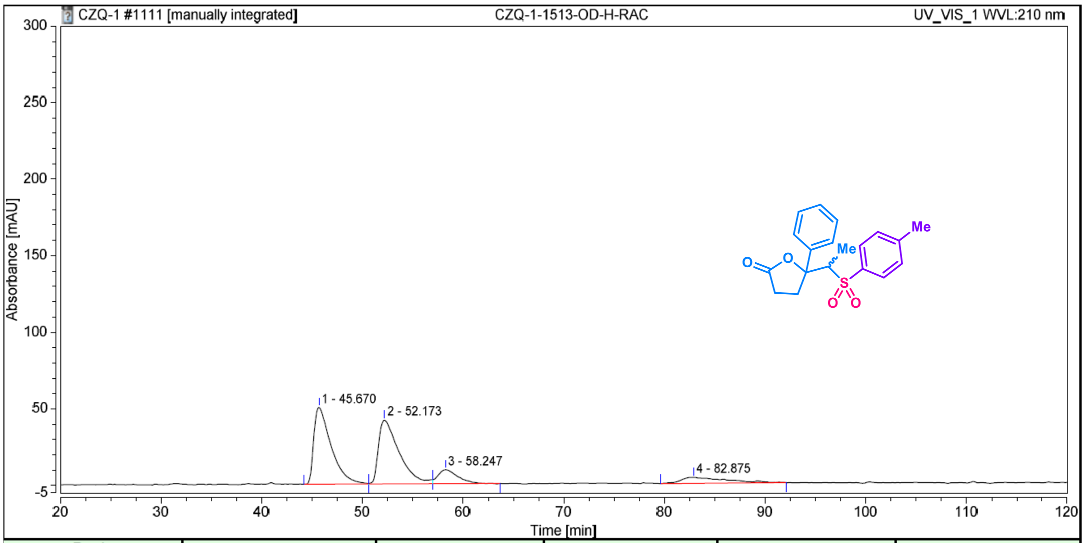

Peak	RetTime min	Area mAU*min	Height mAU	Area %	Height %
1	45.670	100.279	50.184	41.61	47.77
2	52.173	98.832	41.717	41.01	39.71
3	58.247	20.859	9.167	8.66	8.73
4	82.875	21.008	3.976	8.72	3.79
**Total:**		**240.978**	**105.044**	**100.00**	**100.00**

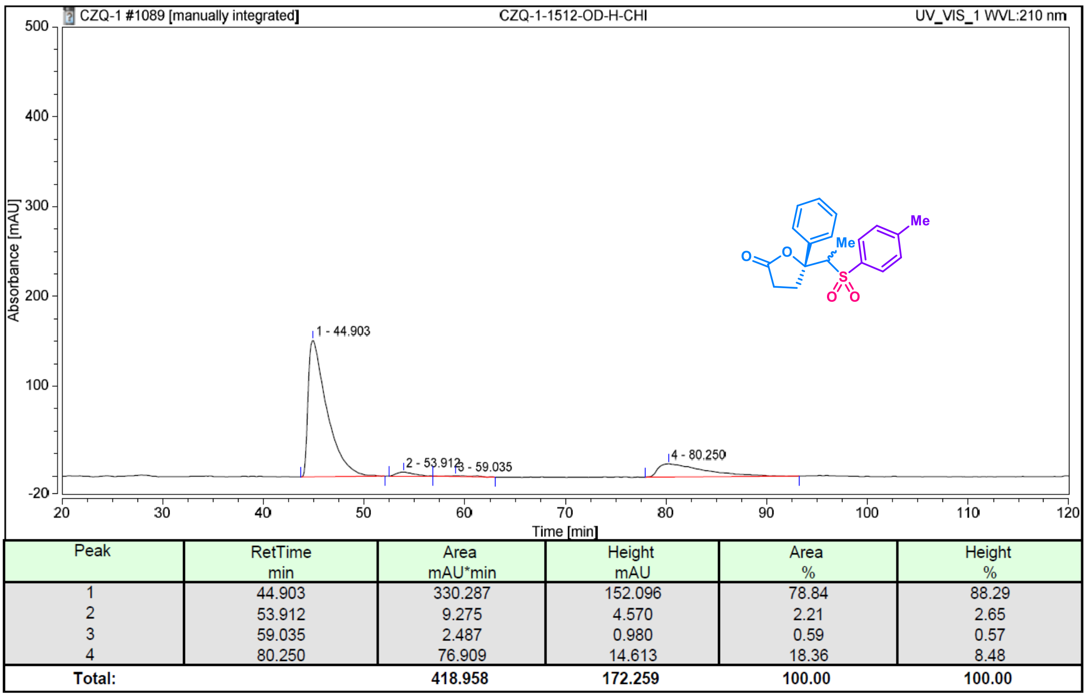

Peak	RetTime min	Area mAU*min	Height mAU	Area %	Height %
1	44.903	330.287	152.096	78.84	88.29
2	53.912	9.275	4.570	2.21	2.65
3	59.035	2.487	0.980	0.59	0.57
4	80.250	76.909	14.613	18.36	8.48
**Total:**		**418.958**	**172.259**	**100.00**	**100.00**

(*R*)-5-cyclohexyl-5-(tosylmethyl)dihydrofuran-2(3*H*)-one

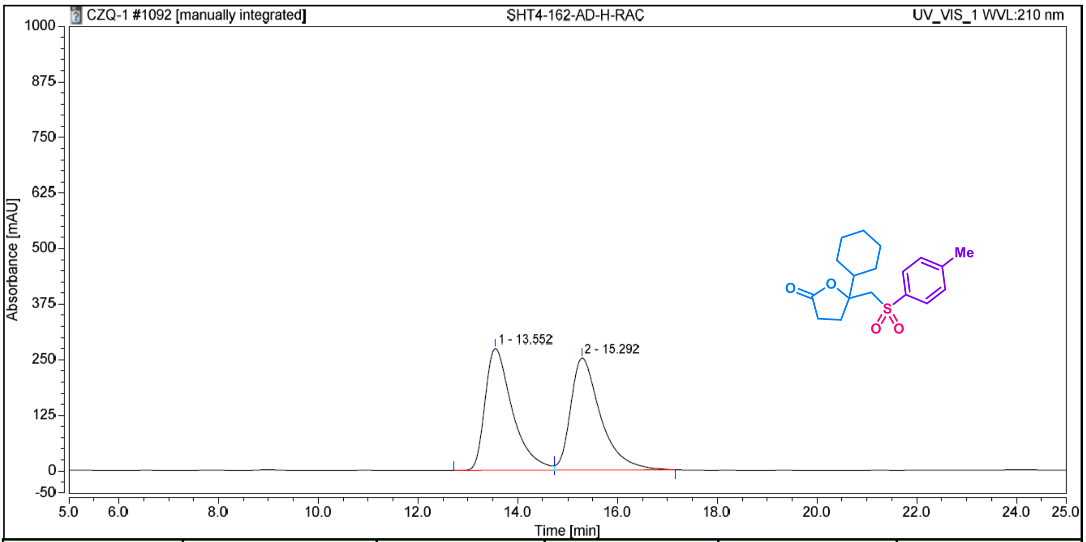

Peak	RetTime min	Area mAU*min	Height mAU	Area %	Height %
1	13.552	174.061	273.884	49.78	52.07
2	15.292	175.600	252.083	50.22	47.93
**Total:**		**349.661**	**525.966**	**100.00**	**100.00**

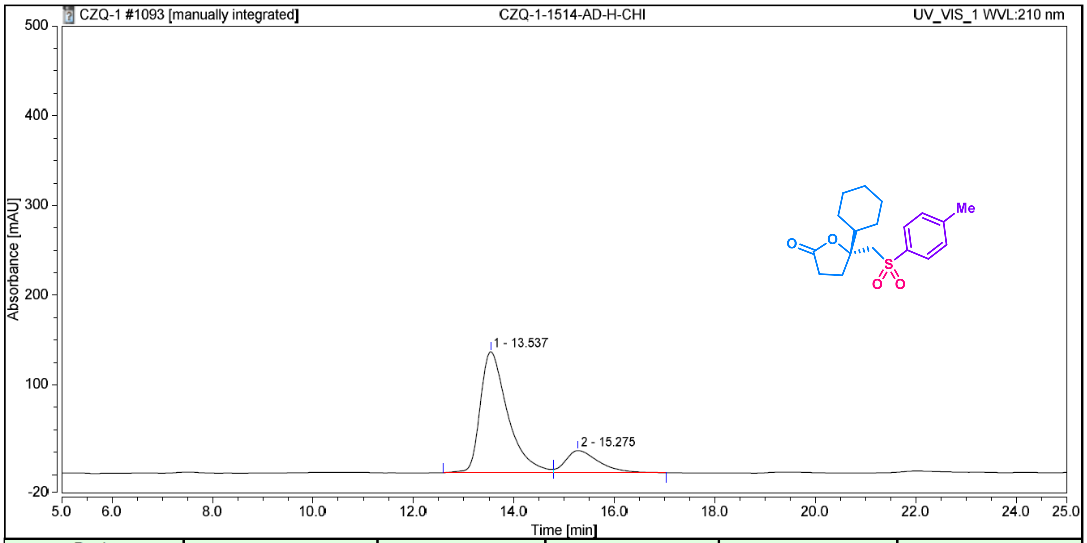

Peak	RetTime min	Area mAU*min	Height mAU	Area %	Height %
1	13.537	85.602	134.999	81.88	84.50
2	15.275	18.945	24.771	18.12	15.50
**Total:**		**104.547**	**159.769**	**100.00**	**100.00**

(*R*)-5-(([1,1'-biphenyl]-4-ylsulfonyl)methyl)-5-phenyldihydrofuran-2(3*H*)-one

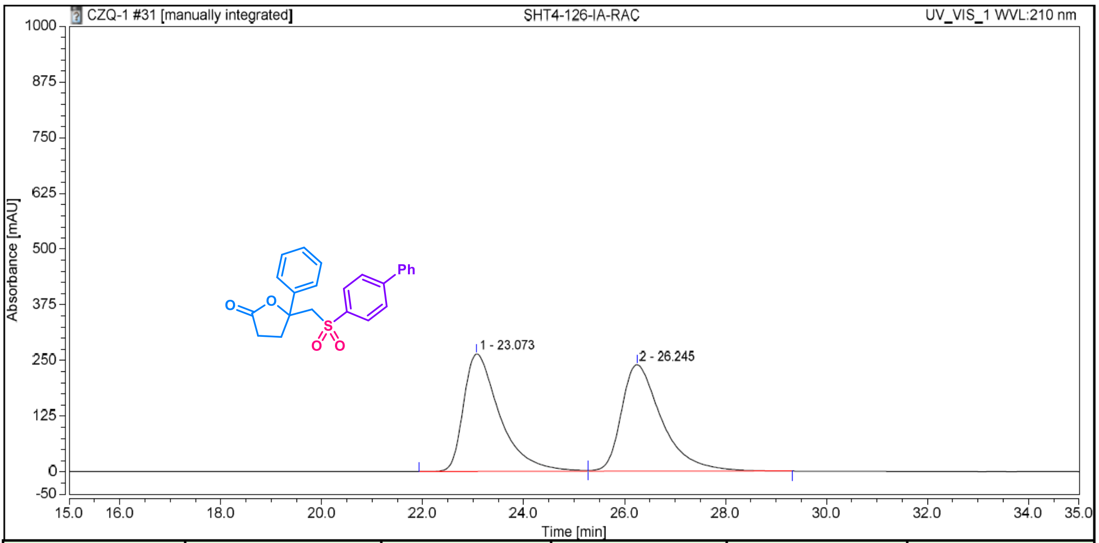

Peak	RetTime min	Area mAU*min	Height mAU	Area %	Height %
1	23.073	219.340	263.608	49.74	52.44
2	26.245	221.655	239.088	50.26	47.56
**Total:**		**440.995**	**502.696**	**100.00**	**100.00**

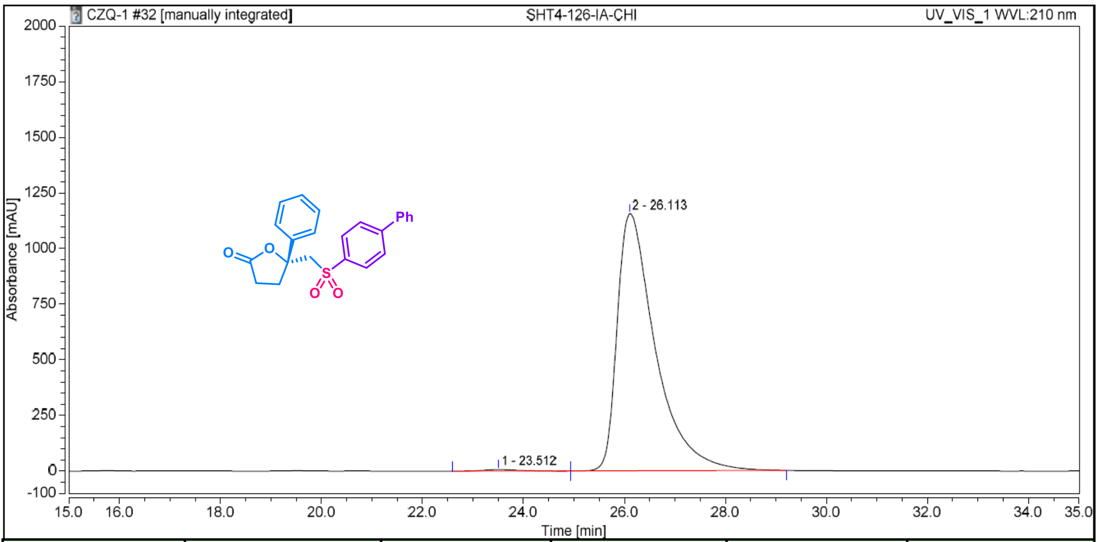

Peak	RetTime min	Area mAU*min	Height mAU	Area %	Height %
1	23.512	4.194	6.501	0.41	0.56
2	26.113	1009.931	1156.253	99.59	99.44
**Total:**		**1014.125**	**1162.754**	**100.00**	**100.00**

(*R*)-5-((naphthalen-2-ylsulfonyl)methyl)-5-phenyldihydrofuran-2(3*H*)-one

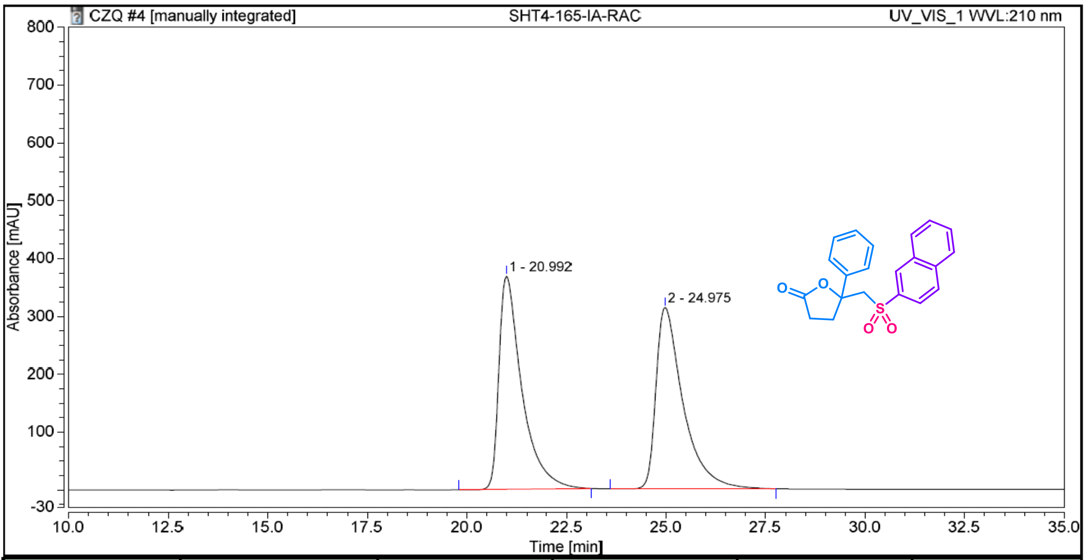

Peak	RetTime min	Area mAU*min	Height mAU	Area %	Height %
1	20.992	245.677	368.718	49.87	54.03
2	24.975	246.986	313.686	50.13	45.97
**Total:**		**492.663**	**682.404**	**100.00**	**100.00**

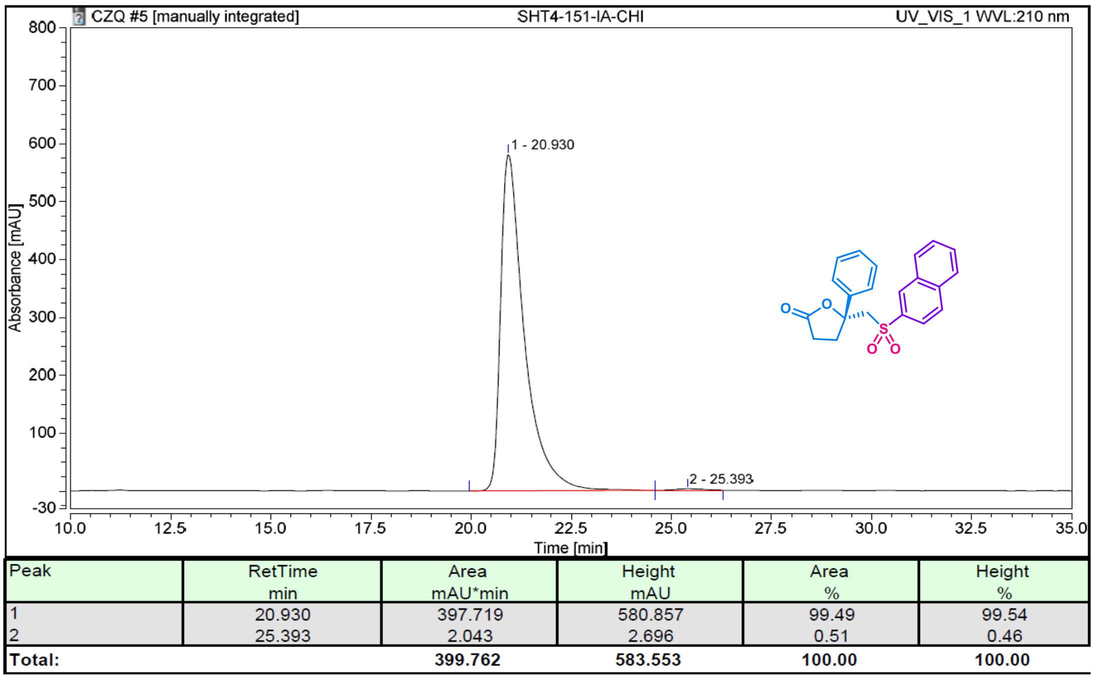

Peak	RetTime min	Area mAU*min	Height mAU	Area %	Height %
1	20.930	397.719	580.857	99.49	99.54
2	25.393	2.043	2.696	0.51	0.46
**Total:**		**399.762**	**583.553**	**100.00**	**100.00**

(*R*)-5-(((4-ethynylphenyl)sulfonyl)methyl)-5-phenyldihydrofuran-2(3*H*)-one

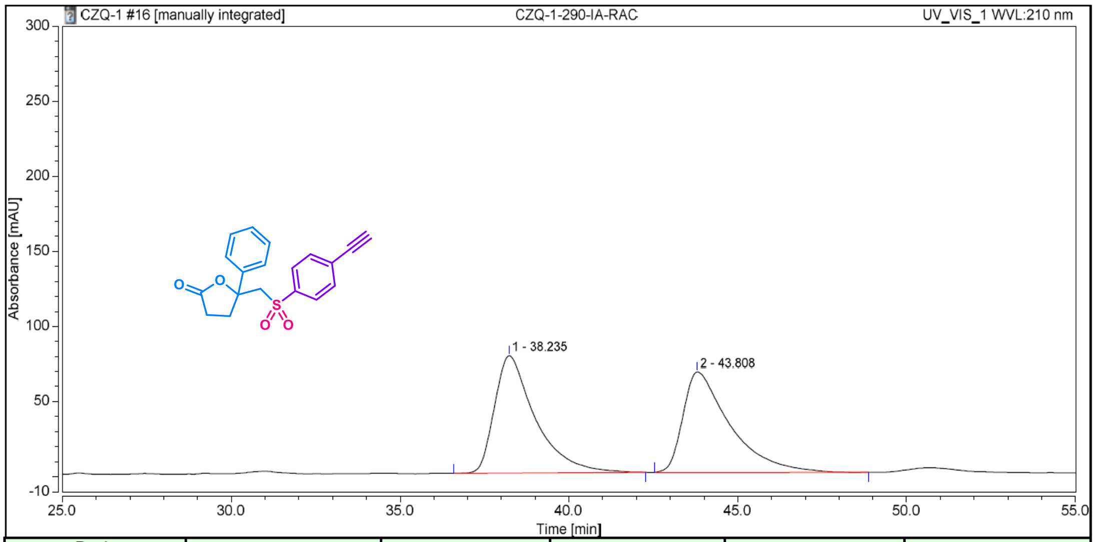

Peak	RetTime<br>min	Area<br>mAU*min	Height<br>mAU	Area<br>%	Height<br>%
1	38.235	109.224	78.203	50.36	53.89
2	43.808	107.643	66.902	49.64	46.11
**Total:**		**216.867**	**145.105**	**100.00**	**100.00**

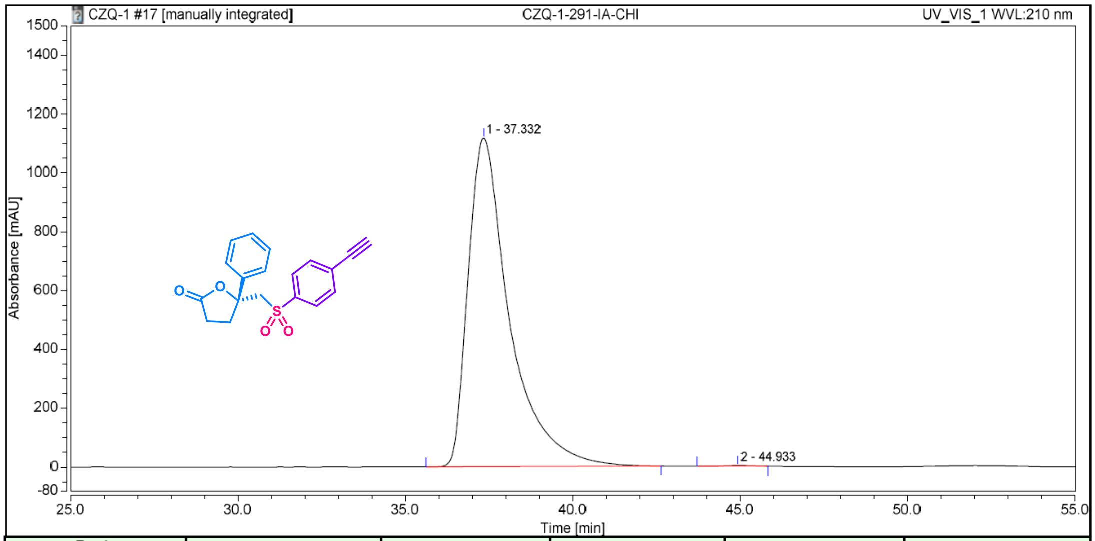

Peak	RetTime<br>min	Area<br>mAU*min	Height<br>mAU	Area<br>%	Height<br>%
1	37.332	1601.259	1117.510	99.84	99.75
2	44.933	2.537	2.747	0.16	0.25
**Total:**		**1603.797**	**1120.256**	**100.00**	**100.00**

(*R*)-5-phenyl-5-((*o*-tolylsulfonyl)methyl)dihydrofuran-2(3*H*)-one

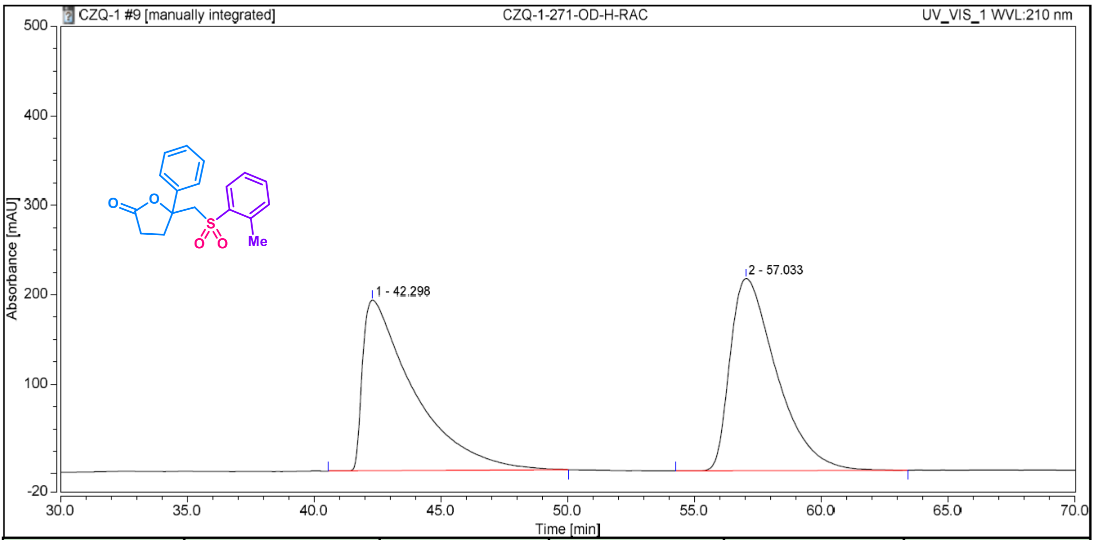

Peak	RetTime min	Area mAU*min	Height mAU	Area %	Height %
1	42.298	445.924	190.684	49.39	47.03
2	57.033	457.015	214.792	50.61	52.97
**Total:**		**902.938**	**405.475**	**100.00**	**100.00**

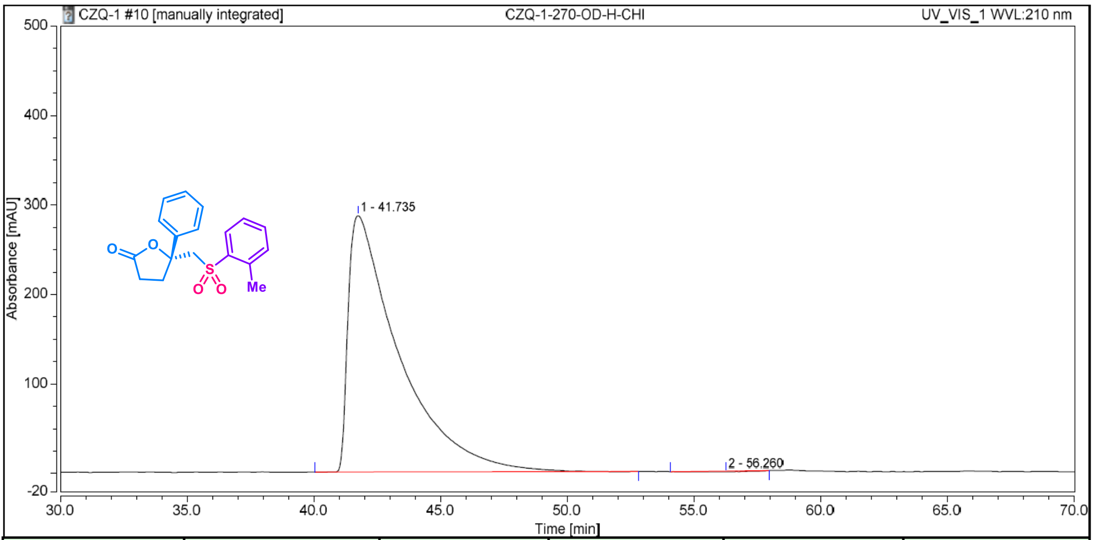

Peak	RetTime min	Area mAU*min	Height mAU	Area %	Height %
1	41.735	666.488	286.613	99.70	99.68
2	56.260	2.014	0.930	0.30	0.32
**Total:**		**668.502**	**287.543**	**100.00**	**100.00**

(*R*)-5-(((4-(*tert*-butyl)phenyl)sulfonyl)methyl)-5-phenyldihydrofuran-2(3*H*)-one

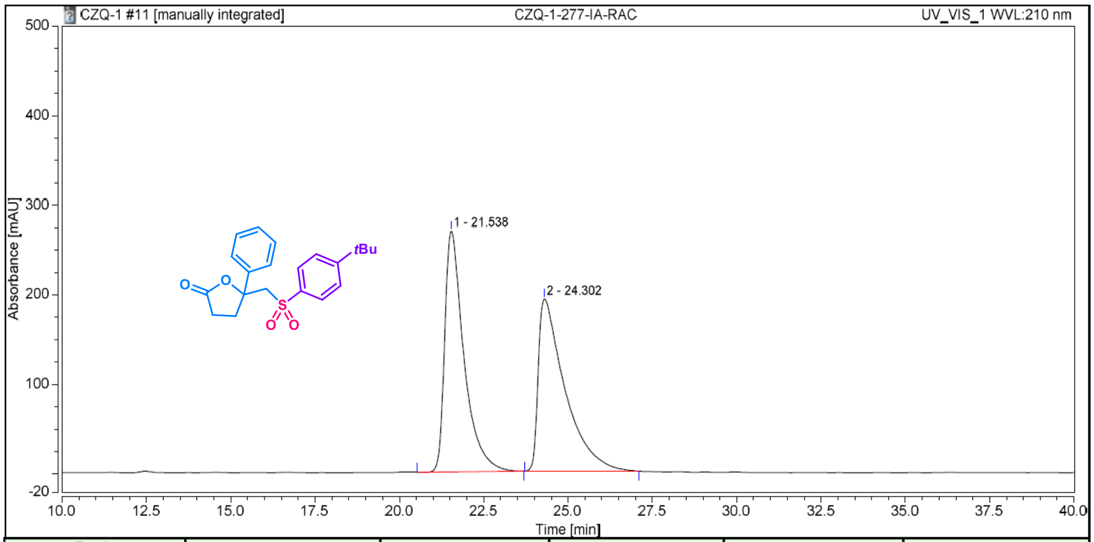

Peak	RetTime min	Area mAU*min	Height mAU	Area %	Height %
1	21.538	177.018	269.025	50.17	58.31
2	24.302	175.812	192.335	49.83	41.69
**Total:**		**352.829**	**461.360**	**100.00**	**100.00**

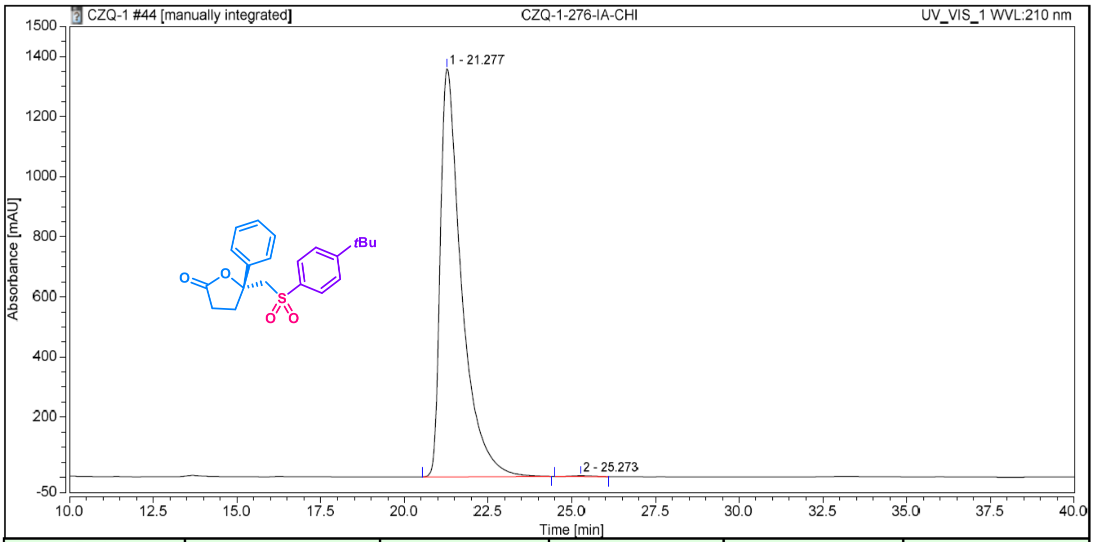

Peak	RetTime min	Area mAU*min	Height mAU	Area %	Height %
1	21.277	999.825	1359.037	99.80	99.80
2	25.273	1.995	2.739	0.20	0.20
**Total:**		**1001.820**	**1361.777**	**100.00**	**100.00**

(*R*)-5-(((4-methoxyphenyl)sulfonyl)methyl)-5-phenyldihydrofuran-2(3*H*)-one

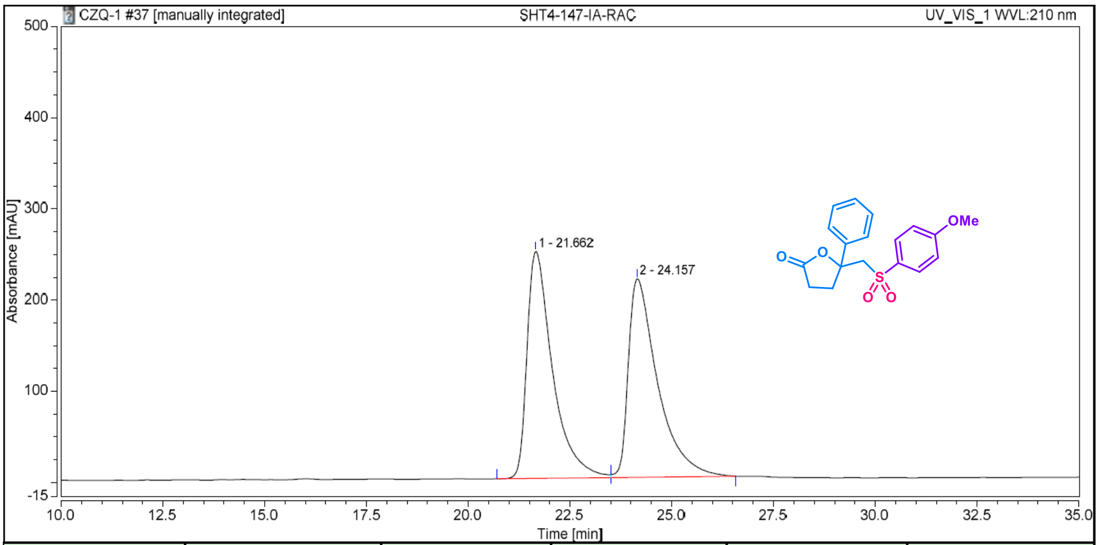

Peak	RetTime min	Area mAU*min	Height mAU	Area %	Height %
1	21.662	180.663	248.796	49.98	53.34
2	24.157	180.786	217.635	50.02	46.66
**Total:**		**361.449**	**466.431**	**100.00**	**100.00**

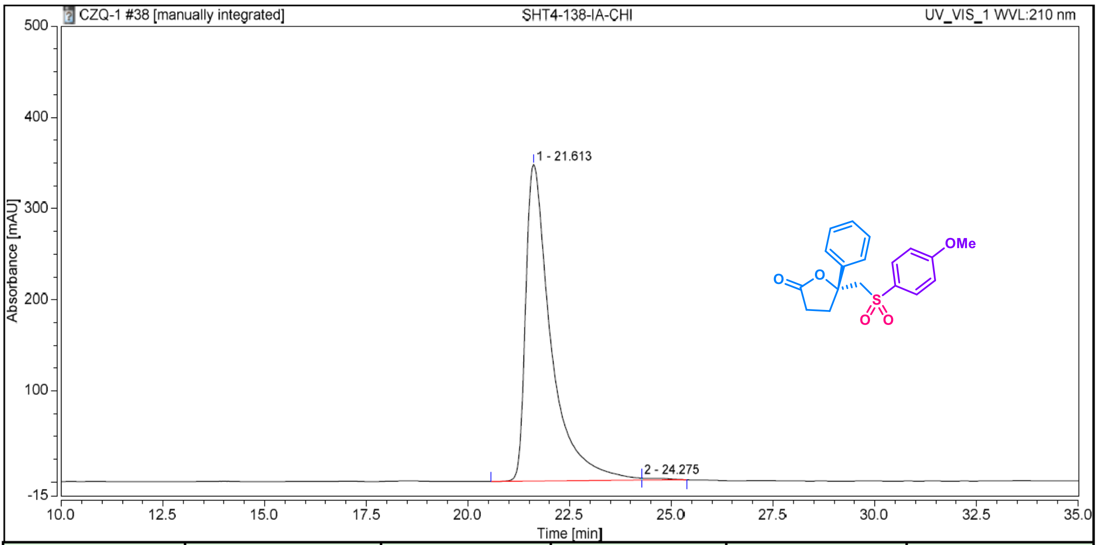

Peak	RetTime min	Area mAU*min	Height mAU	Area %	Height %
1	21.613	250.895	347.386	99.44	99.38
2	24.275	1.422	2.175	0.56	0.62
**Total:**		**252.317**	**349.561**	**100.00**	**100.00**

(*R*)-5-(((4-phenoxyphenyl)sulfonyl)methyl)-5-phenyldihydrofuran-2(3*H*)-one

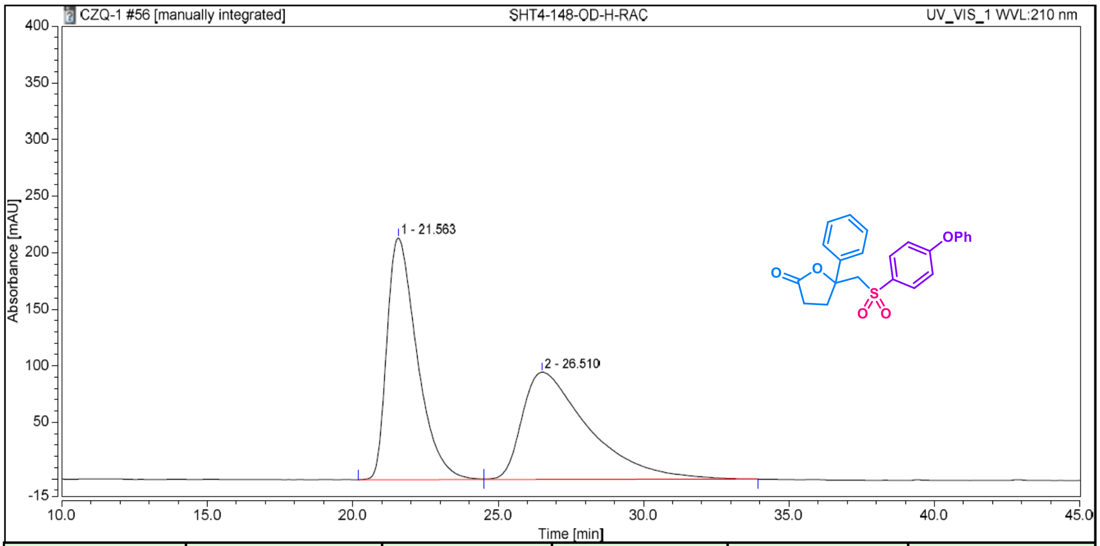

Peak	RetTime min	Area mAU*min	Height mAU	Area %	Height %
1	21.563	256.975	213.661	50.63	69.28
2	26.510	250.623	94.725	49.37	30.72
**Total:**		**507.599**	**308.385**	**100.00**	**100.00**

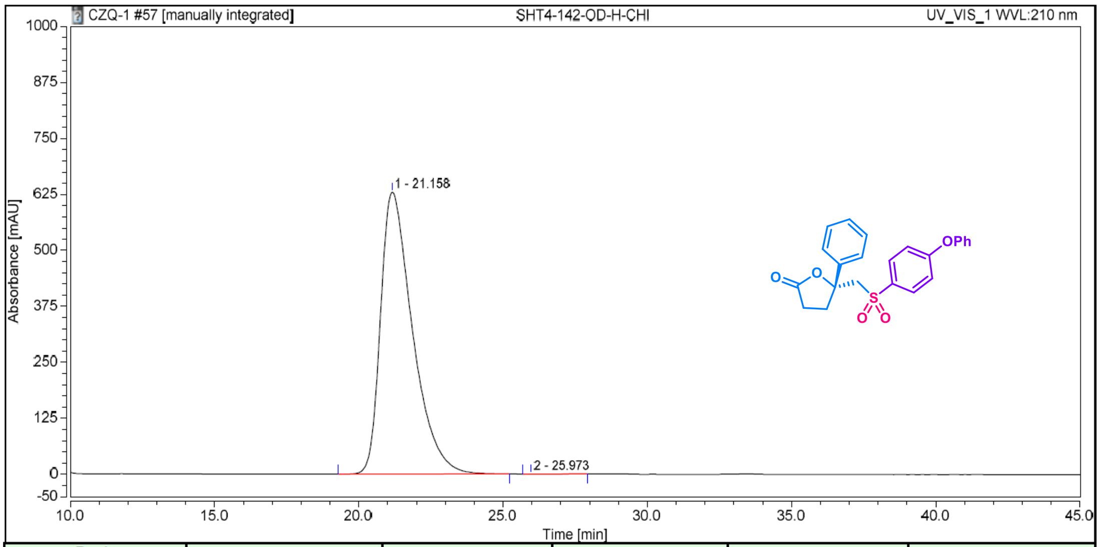

Peak	RetTime min	Area mAU*min	Height mAU	Area %	Height %
1	21.158	808.573	630.182	99.97	99.97
2	25.973	0.265	0.168	0.03	0.03
**Total:**		**808.838**	**630.350**	**100.00**	**100.00**

(*R*)-5-(((3-fluorophenyl)sulfonyl)methyl)-5-phenyldihydrofuran-2(3*H*)-one

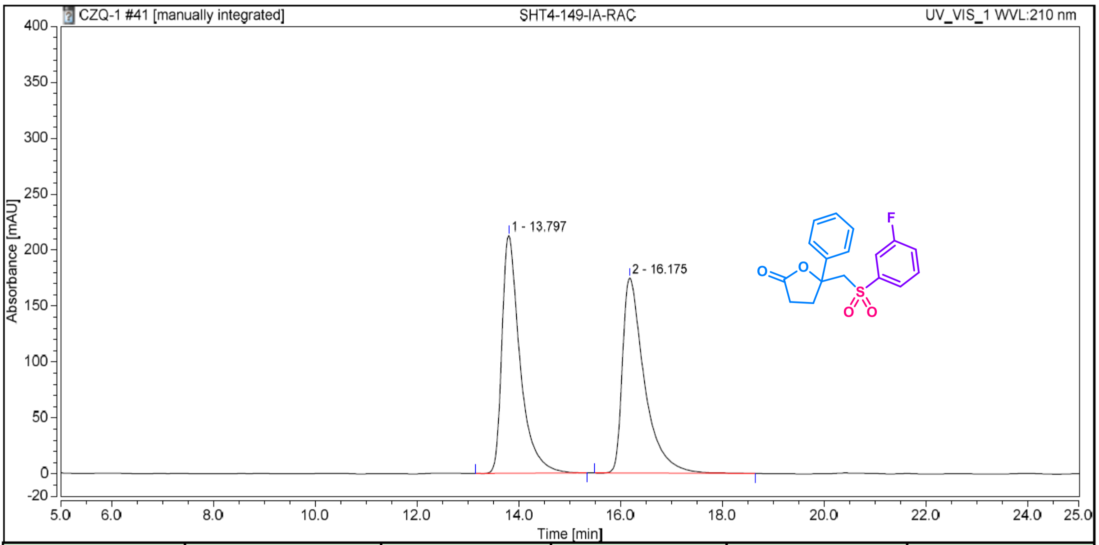

Peak	RetTime min	Area mAU*min	Height mAU	Area %	Height %
1	13.797	87.204	212.815	50.03	54.93
2	16.175	87.105	174.642	49.97	45.07
**Total:**		**174.309**	**387.457**	**100.00**	**100.00**

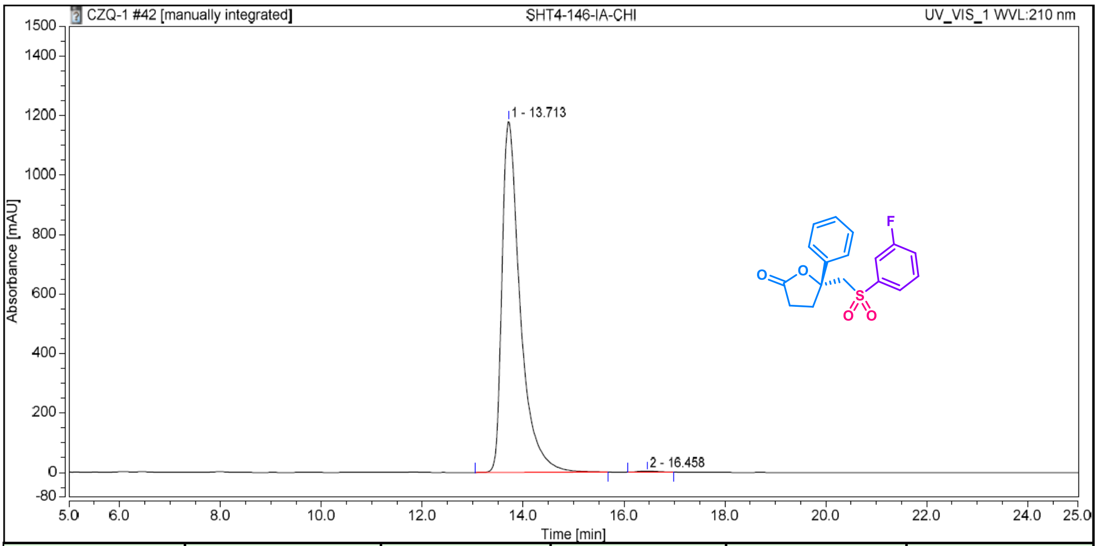

Peak	RetTime min	Area mAU*min	Height mAU	Area %	Height %
1	13.713	494.026	1181.513	99.69	99.70
2	16.458	1.544	3.586	0.31	0.30
**Total:**		**495.570**	**1185.099**	**100.00**	**100.00**

(*R*)-5-(((4-bromophenyl)sulfonyl)methyl)-5-phenyldihydrofuran-2(3*H*)-one

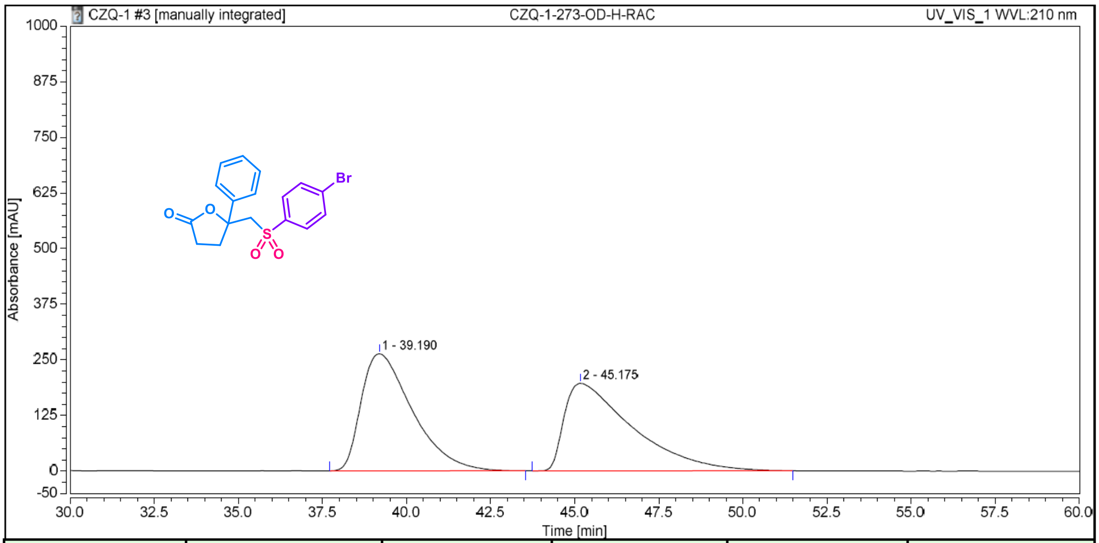

Peak	RetTime min	Area mAU*min	Height mAU	Area %	Height %
1	39.190	464.524	263.290	50.15	57.24
2	45.175	461.714	196.694	49.85	42.76
**Total:**		**926.238**	**459.985**	**100.00**	**100.00**

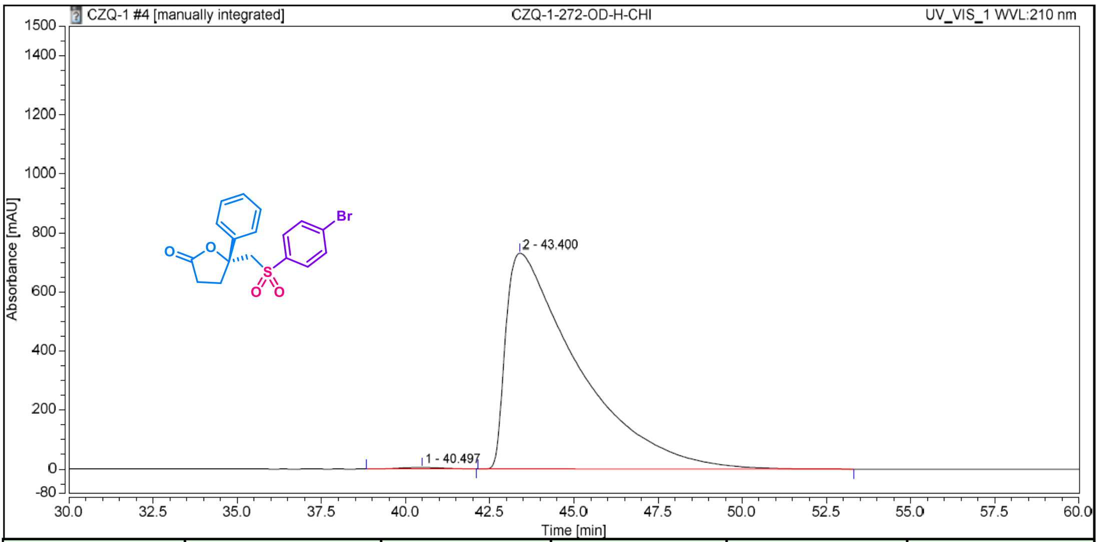

Peak	RetTime min	Area mAU*min	Height mAU	Area %	Height %
1	40.497	6.742	4.826	0.37	0.66
2	43.400	1821.381	730.340	99.63	99.34
**Total:**		**1828.122**	**735.166**	**100.00**	**100.00**

(*R*)-5-(((3-iodophenyl)sulfonyl)methyl)-5-phenyldihydrofuran-2(3*H*)-one

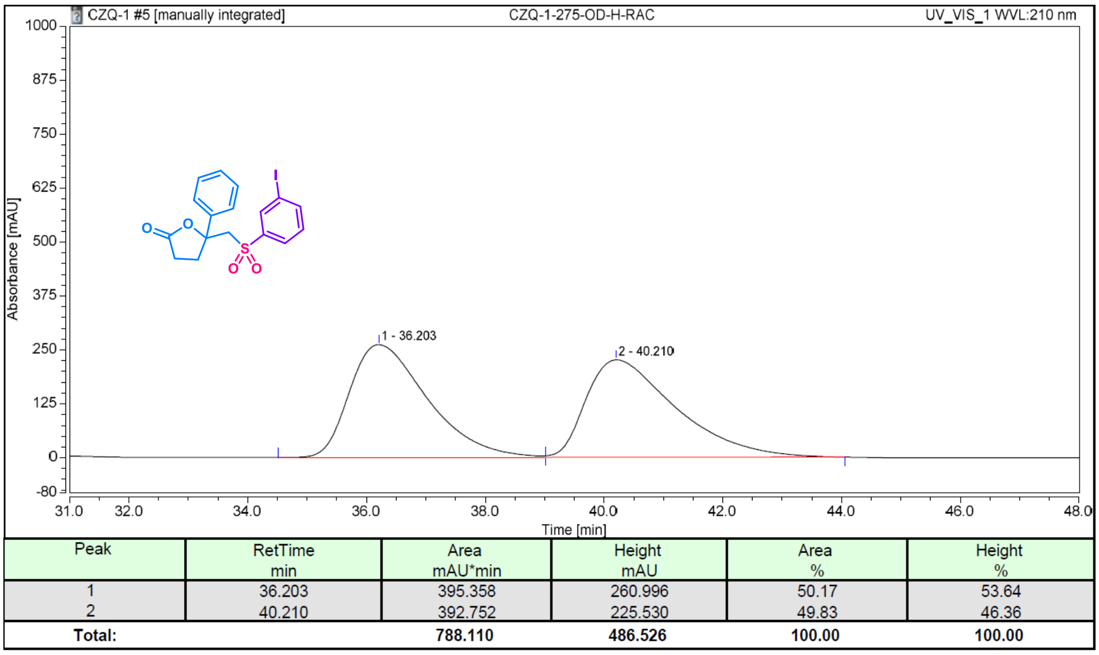

Peak	RetTime<br>min	Area<br>mAU*min	Height<br>mAU	Area<br>%	Height<br>%
1	36.203	395.358	260.996	50.17	53.64
2	40.210	392.752	225.530	49.83	46.36
**Total:**		**788.110**	**486.526**	**100.00**	**100.00**

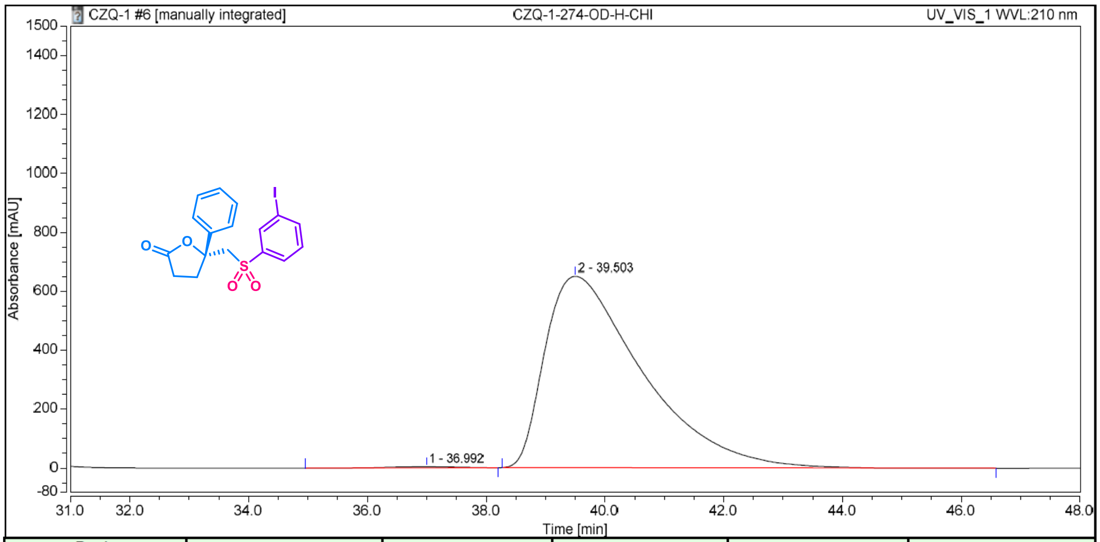

Peak	RetTime<br>min	Area<br>mAU*min	Height<br>mAU	Area<br>%	Height<br>%
1	36.992	4.898	3.710	0.40	0.57
2	39.503	1232.591	649.465	99.60	99.43
**Total:**		**1237.489**	**653.174**	**100.00**	**100.00**

(*R*)-5-(((3-(methylthio)phenyl)sulfonyl)methyl)-5-phenyldihydrofuran-2(3*H*)-one

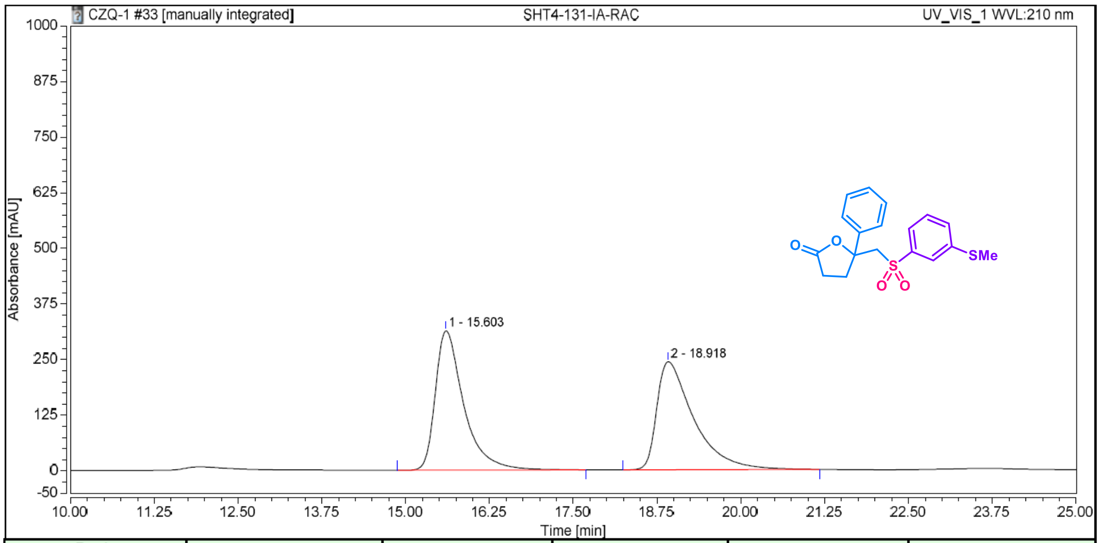

Peak	RetTime min	Area mAU*min	Height mAU	Area %	Height %
1	15.603	154.252	313.613	49.90	56.35
2	18.918	154.892	242.928	50.10	43.65
**Total:**		**309.144**	**556.541**	**100.00**	**100.00**

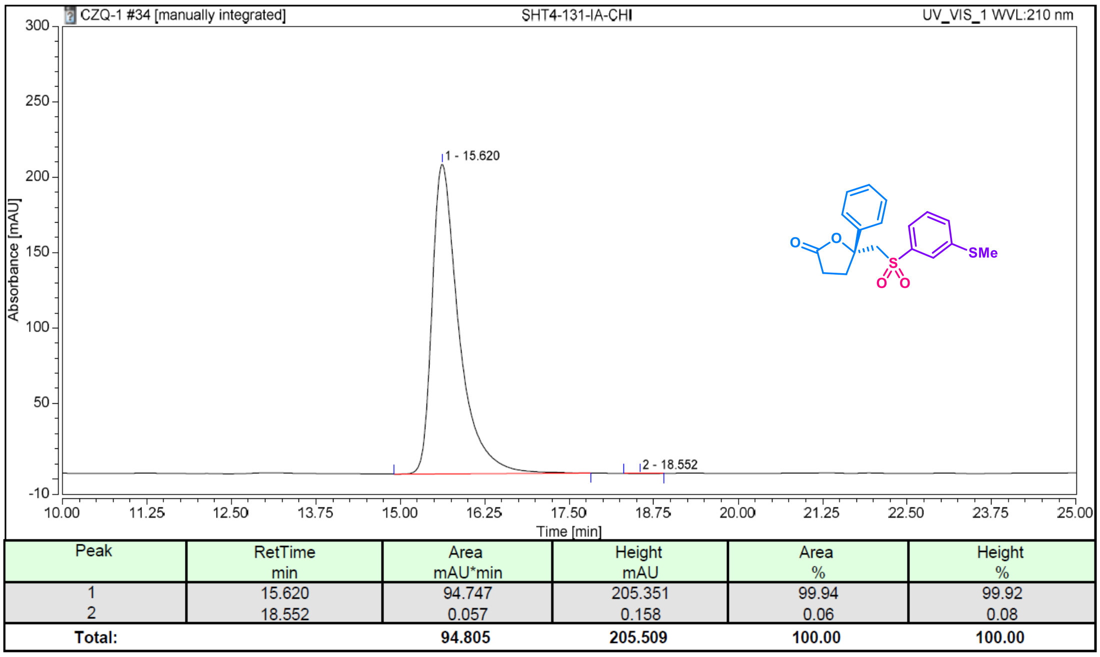

Peak	RetTime min	Area mAU*min	Height mAU	Area %	Height %
1	15.620	94.747	205.351	99.94	99.92
2	18.552	0.057	0.158	0.06	0.08
**Total:**		**94.805**	**205.509**	**100.00**	**100.00**

(*R*)-5-(((3-methoxyphenyl)sulfonyl)methyl)-5-phenyldihydrofuran-2(3*H*)-one

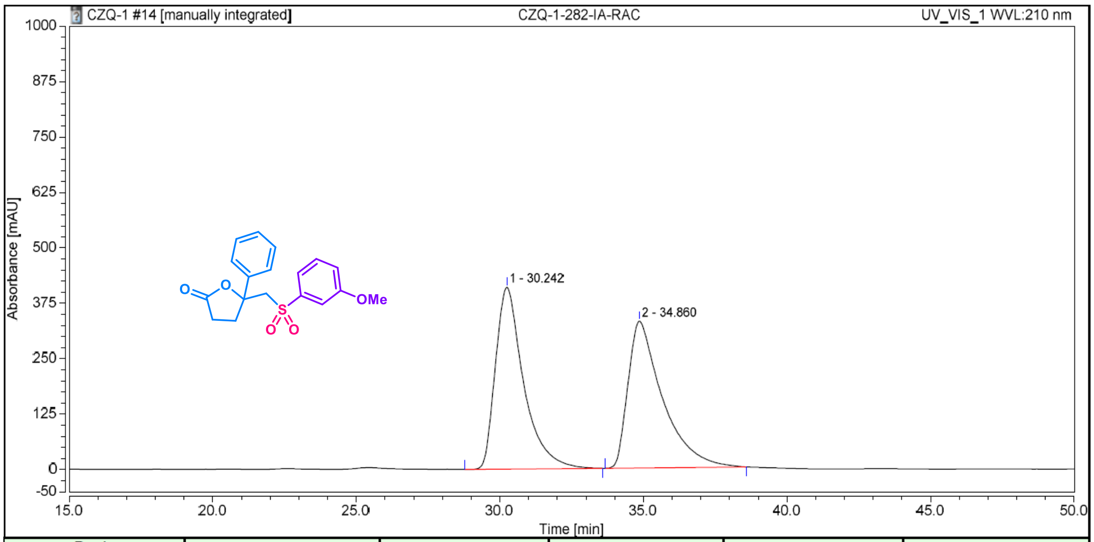

Peak	RetTime<br>min	Area<br>mAU*min	Height<br>mAU	Area<br>%	Height<br>%
1	30.242	466.218	410.659	50.29	55.33
2	34.860	460.820	331.490	49.71	44.67
**Total:**		**927.039**	**742.150**	**100.00**	**100.00**

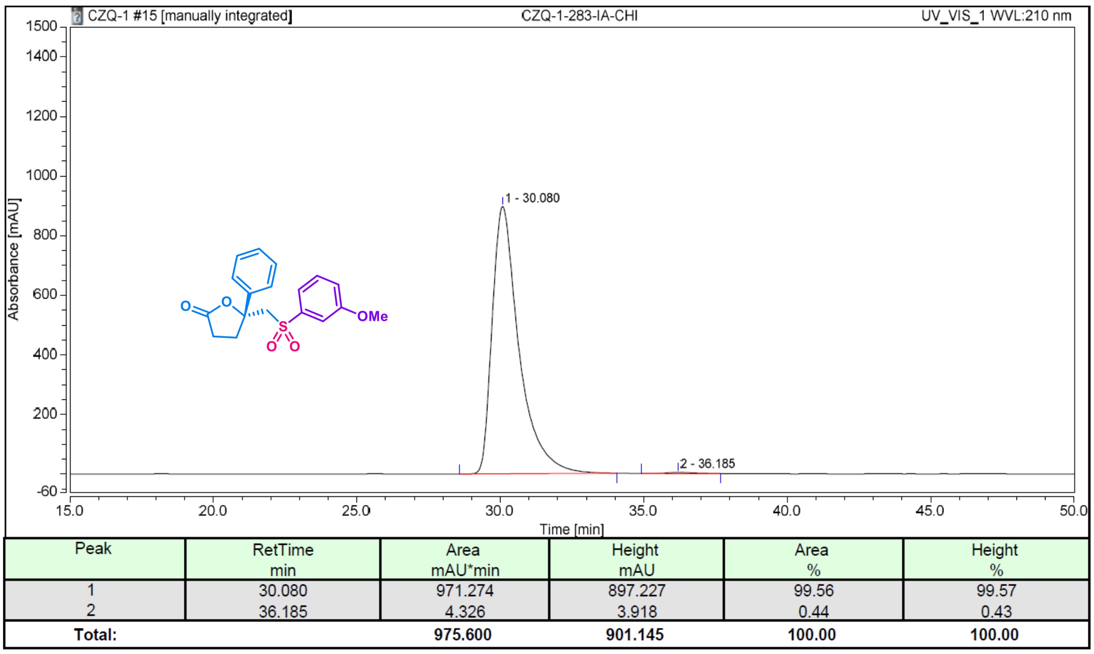

Peak	RetTime<br>min	Area<br>mAU*min	Height<br>mAU	Area<br>%	Height<br>%
1	30.080	971.274	897.227	99.56	99.57
2	36.185	4.326	3.918	0.44	0.43
**Total:**		**975.600**	**901.145**	**100.00**	**100.00**

(*R*)-5-(((4-benzoylphenyl)sulfonyl)methyl)-5-phenyldihydrofuran-2(3*H*)-one

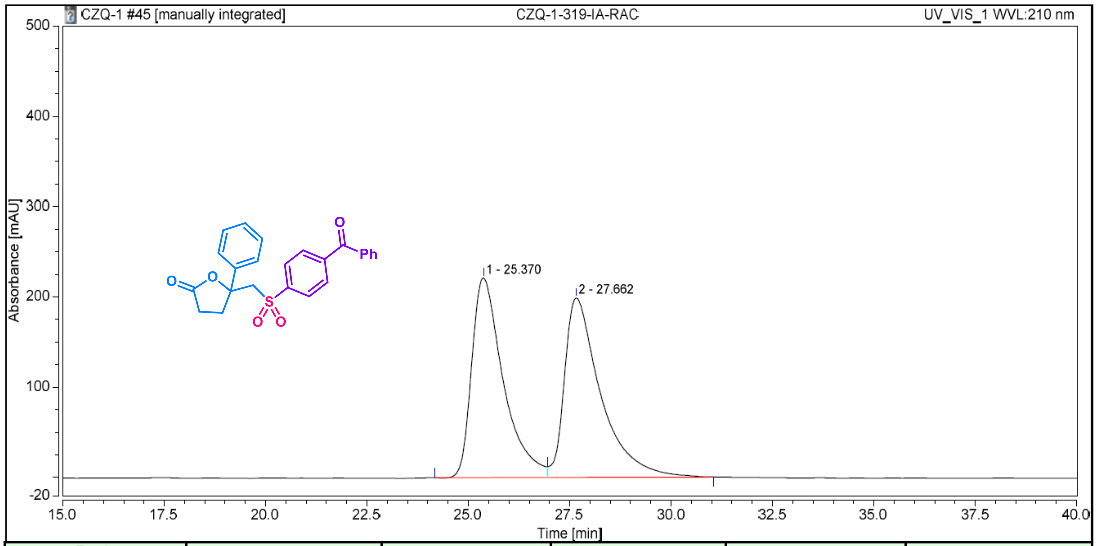

Peak	RetTime<br>min	Area<br>mAU*min	Height<br>mAU	Area<br>%	Height<br>%
1	25.370	199.289	221.305	49.28	52.69
2	27.662	205.084	198.747	50.72	47.31
**Total:**		**404.373**	**420.052**	**100.00**	**100.00**

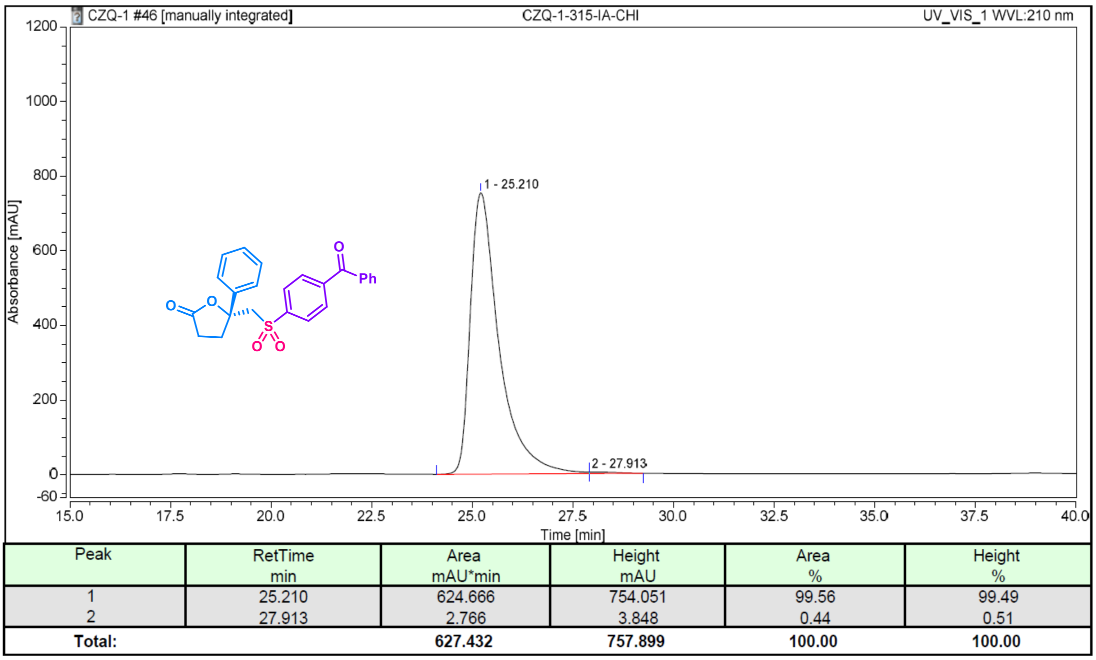

Peak	RetTime<br>min	Area<br>mAU*min	Height<br>mAU	Area<br>%	Height<br>%
1	25.210	624.666	754.051	99.56	99.49
2	27.913	2.766	3.848	0.44	0.51
**Total:**		**627.432**	**757.899**	**100.00**	**100.00**

(*R*)-5-(((4-acetylphenyl)sulfonyl)methyl)-5-phenyldihydrofuran-2(3*H*)-one

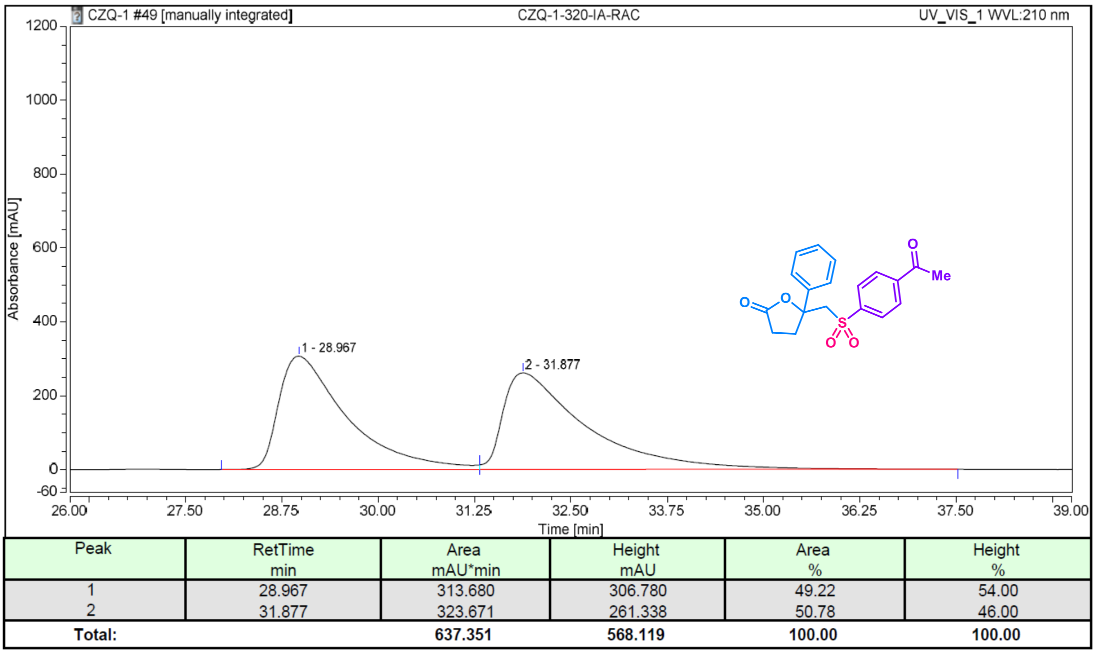

Peak	RetTime min	Area mAU*min	Height mAU	Area %	Height %
1	28.967	313.680	306.780	49.22	54.00
2	31.877	323.671	261.338	50.78	46.00
**Total:**		**637.351**	**568.119**	**100.00**	**100.00**

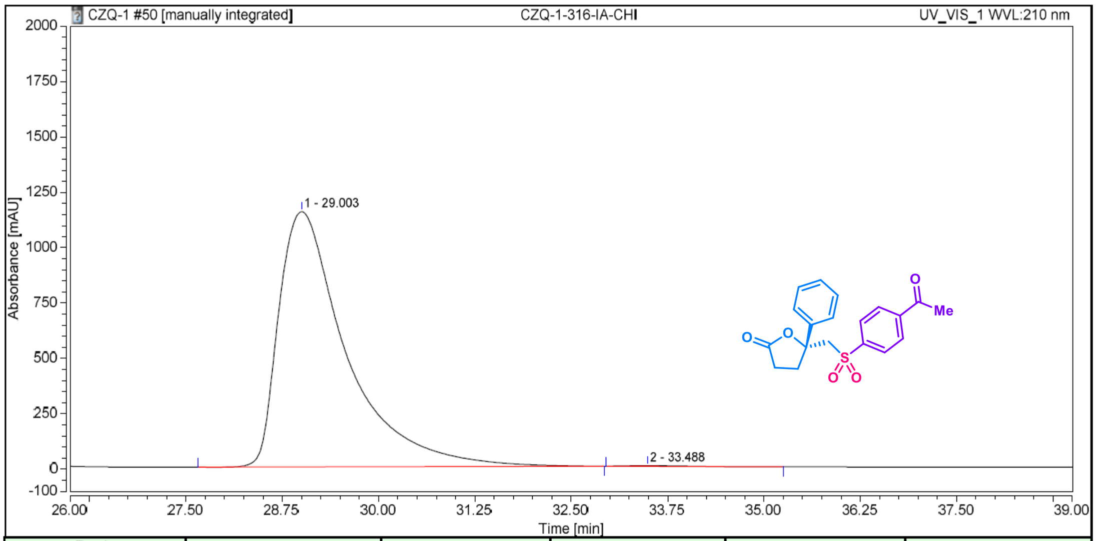

Peak	RetTime min	Area mAU*min	Height mAU	Area %	Height %
1	29.003	1172.984	1153.127	99.81	99.75
2	33.488	2.216	2.946	0.19	0.25
**Total:**		**1175.200**	**1156.073**	**100.00**	**100.00**

methyl (*R*)-4-(((5-oxo-2-phenyltetrahydrofuran-2-yl)methyl)sulfonyl)benzoate

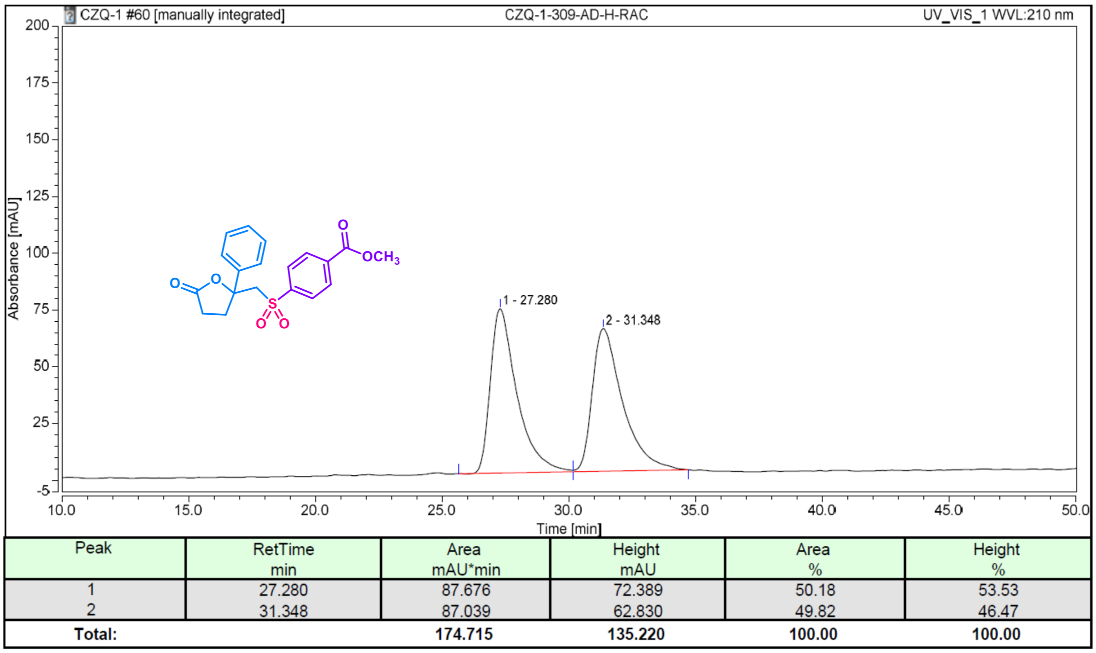

Peak	RetTime min	Area mAU*min	Height mAU	Area %	Height %
1	27.280	87.676	72.389	50.18	53.53
2	31.348	87.039	62.830	49.82	46.47
**Total:**		**174.715**	**135.220**	**100.00**	**100.00**

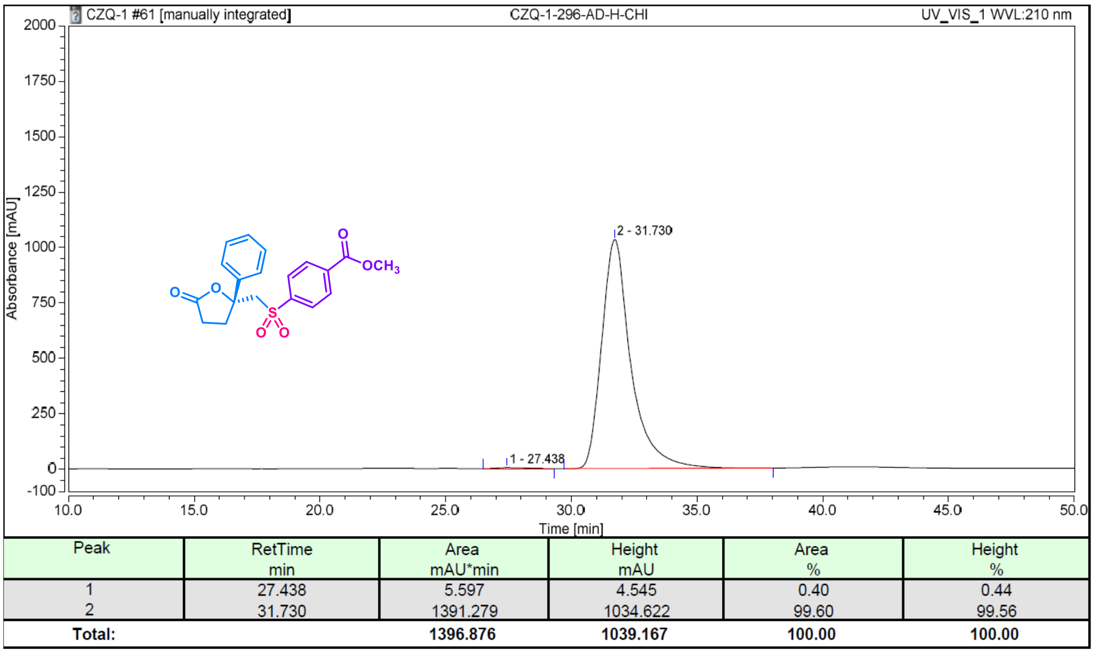

Peak	RetTime min	Area mAU*min	Height mAU	Area %	Height %
1	27.438	5.597	4.545	0.40	0.44
2	31.730	1391.279	1034.622	99.60	99.56
**Total:**		**1396.876**	**1039.167**	**100.00**	**100.00**

(*R*)-5-phenyl-5-(((4-(trifluoromethyl)phenyl)sulfonyl)methyl)dihydrofuran-2(3*H*)-one

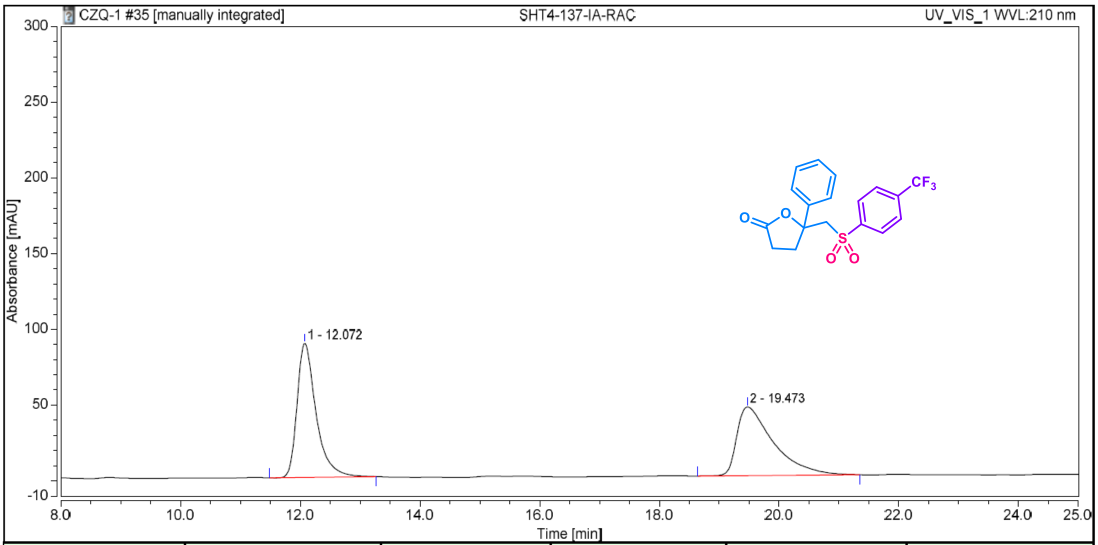

Peak	RetTime min	Area mAU*min	Height mAU	Area %	Height %
1	12.072	32.569	88.622	50.32	66.17
2	19.473	32.157	45.304	49.68	33.83
**Total:**		**64.726**	**133.927**	**100.00**	**100.00**

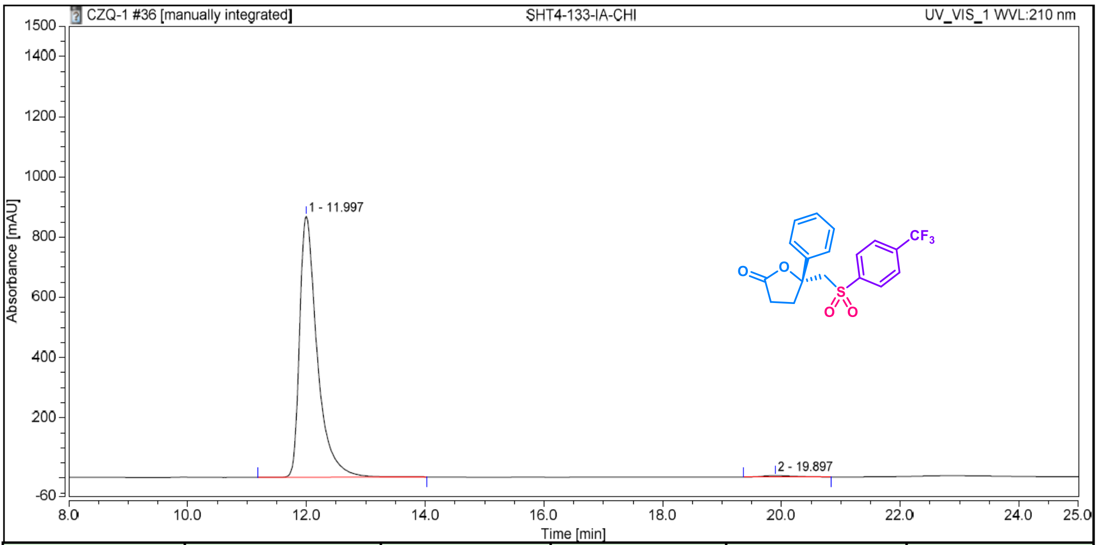

Peak	RetTime min	Area mAU*min	Height mAU	Area %	Height %
1	11.997	297.891	867.667	99.00	99.45
2	19.897	3.013	4.762	1.00	0.55
**Total:**		**300.904**	**872.429**	**100.00**	**100.00**

(*R*)-5-(((4-nitrophenyl)sulfonyl)methyl)-5-phenyldihydrofuran-2(3*H*)-one

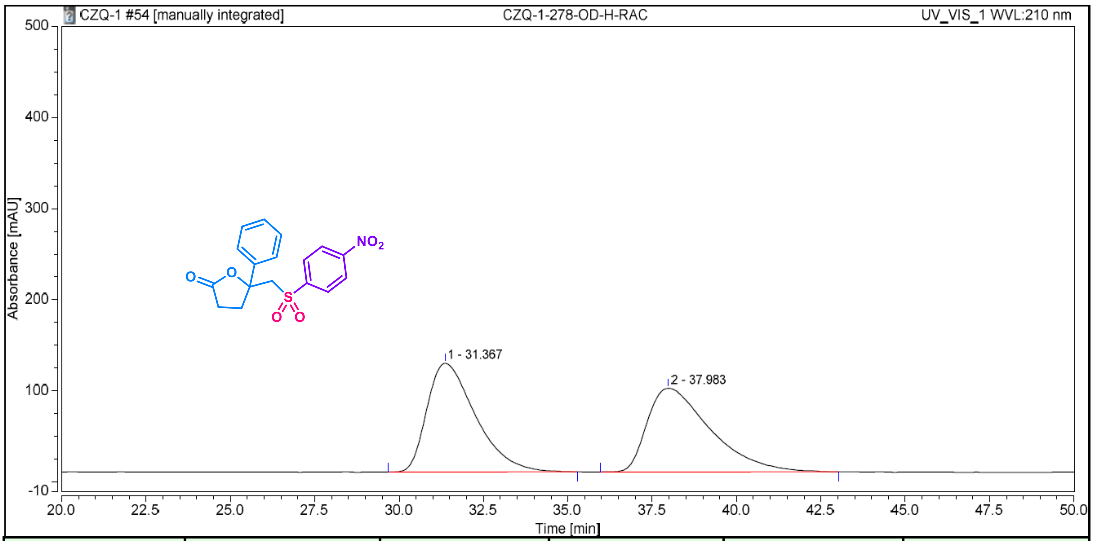

Peak	RetTime min	Area mAU*min	Height mAU	Area %	Height %
1	31.367	199.127	119.333	50.07	56.56
2	37.983	198.552	91.655	49.93	43.44
**Total:**		**397.679**	**210.989**	**100.00**	**100.00**

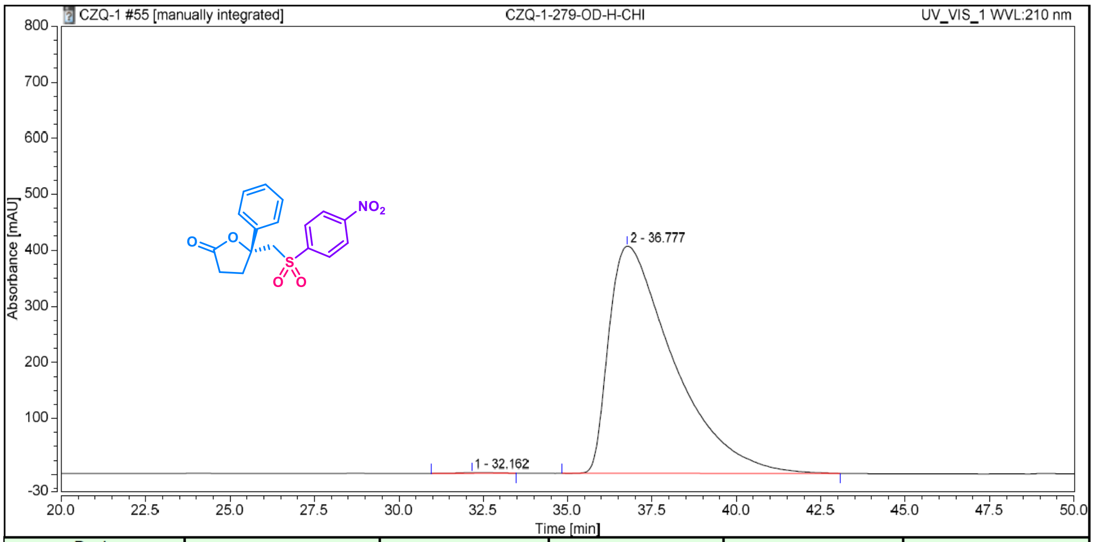

Peak	RetTime min	Area mAU*min	Height mAU	Area %	Height %
1	32.162	1.444	1.199	0.16	0.29
2	36.777	902.580	405.754	99.84	99.71
**Total:**		**904.025**	**406.953**	**100.00**	**100.00**

ethyl (*R*)-5-(((5-oxo-2-phenyltetrahydrofuran-2-yl)methyl)sulfonyl)benzofuran - 2-carboxylate

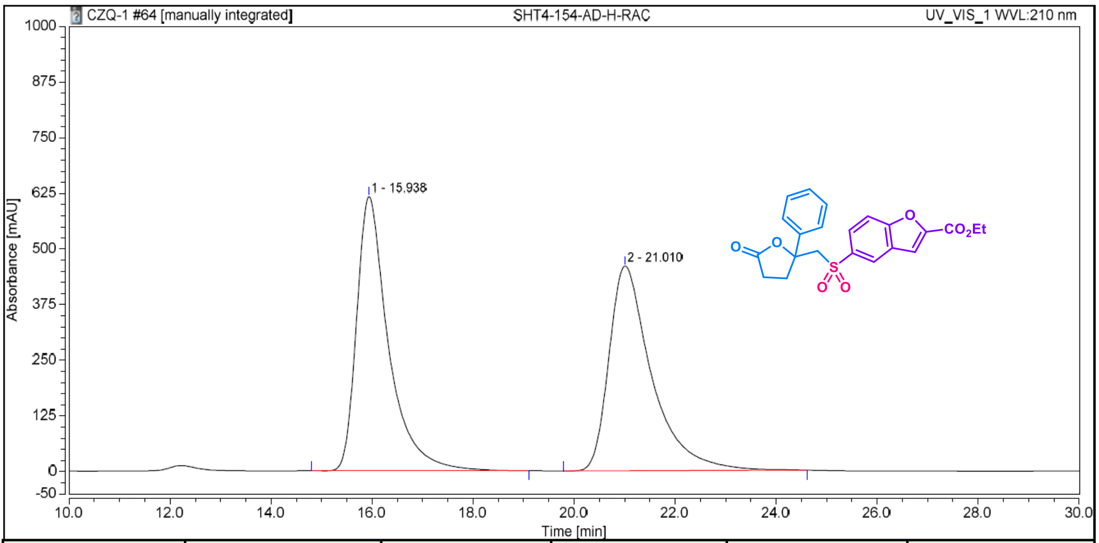

Peak	RetTime min	Area mAU*min	Height mAU	Area %	Height %
1	15.938	448.741	615.672	49.93	57.23
2	21.010	450.051	460.128	50.07	42.77
**Total:**		**898.792**	**1075.800**	**100.00**	**100.00**

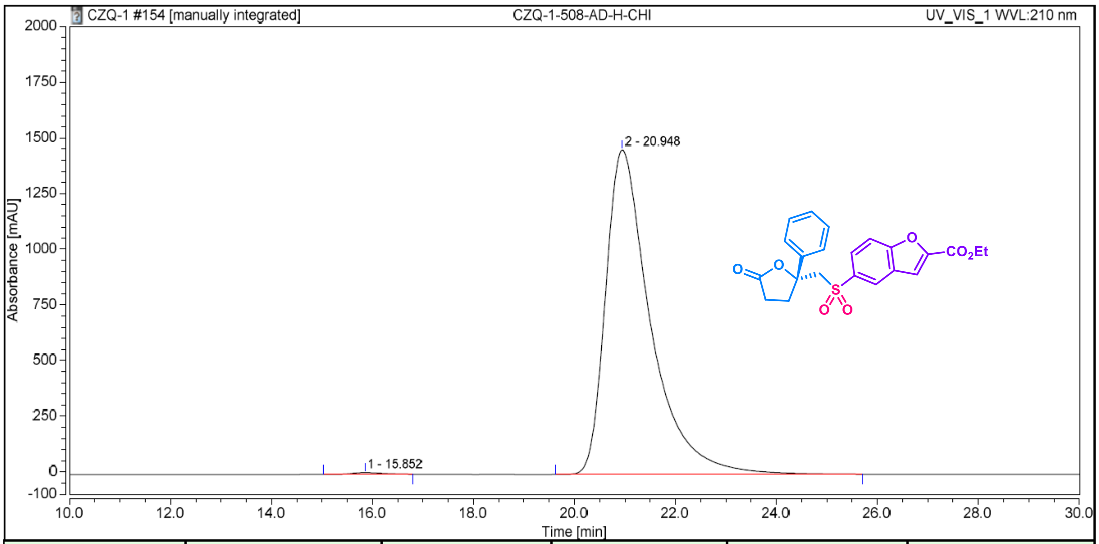

Peak	RetTime min	Area mAU*min	Height mAU	Area %	Height %
1	15.852	4.972	7.442	0.33	0.51
2	20.948	1501.269	1456.762	99.67	99.49
**Total:**		**1506.241**	**1464.204**	**100.00**	**100.00**

(1*S*,2*R*,4*R*)-2-isopropyl-4-methylcyclohexyl 4-((((*R*)-5-oxo-2-phenyltetrahydro-furan-2-yl)methyl)sulfonyl)benzoate

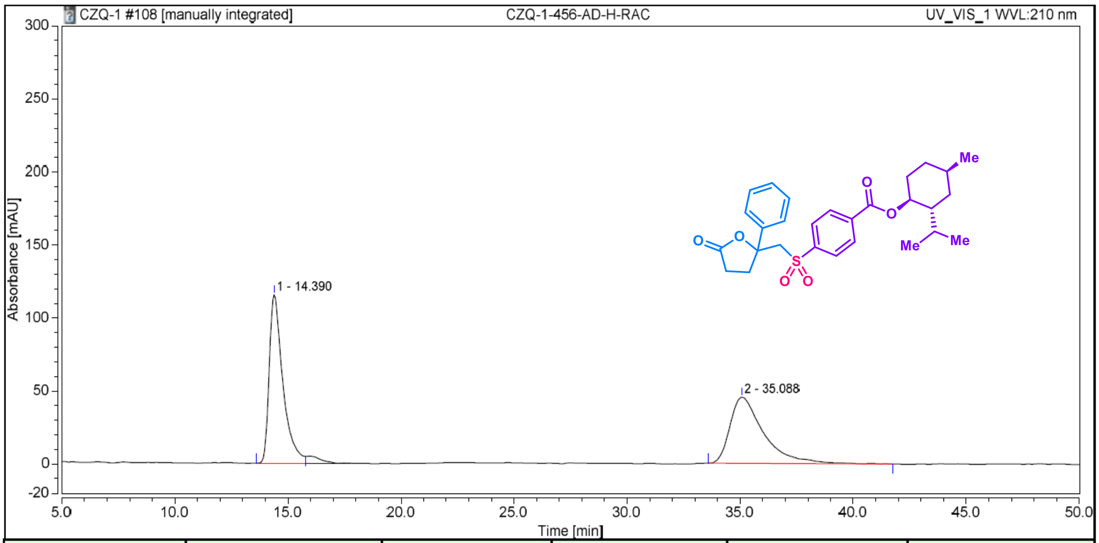

Peak	RetTime min	Area mAU*min	Height mAU	Area %	Height %
1	14.390	80.572	115.390	50.80	71.84
2	35.088	78.035	45.226	49.20	28.16
**Total:**		**158.607**	**160.616**	**100.00**	**100.00**

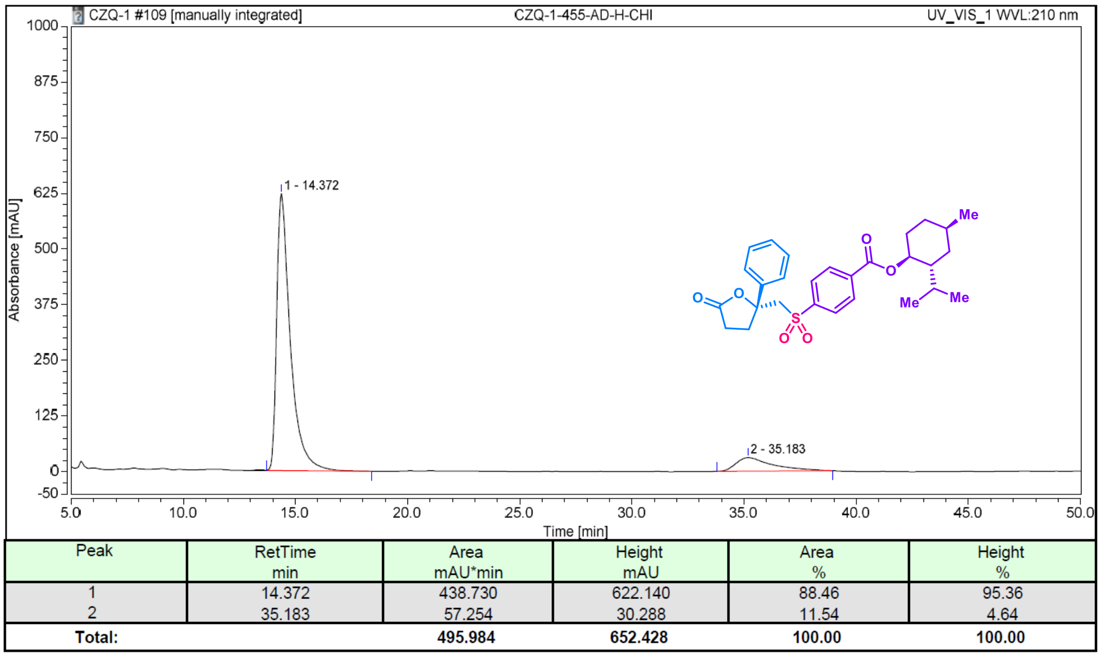

Peak	RetTime min	Area mAU*min	Height mAU	Area %	Height %
1	14.372	438.730	622.140	88.46	95.36
2	35.183	57.254	30.288	11.54	4.64
**Total:**		**495.984**	**652.428**	**100.00**	**100.00**

(*R*)-6-phenyl-6-(tosylmethyl)tetrahydro-2*H*-pyran-2-one

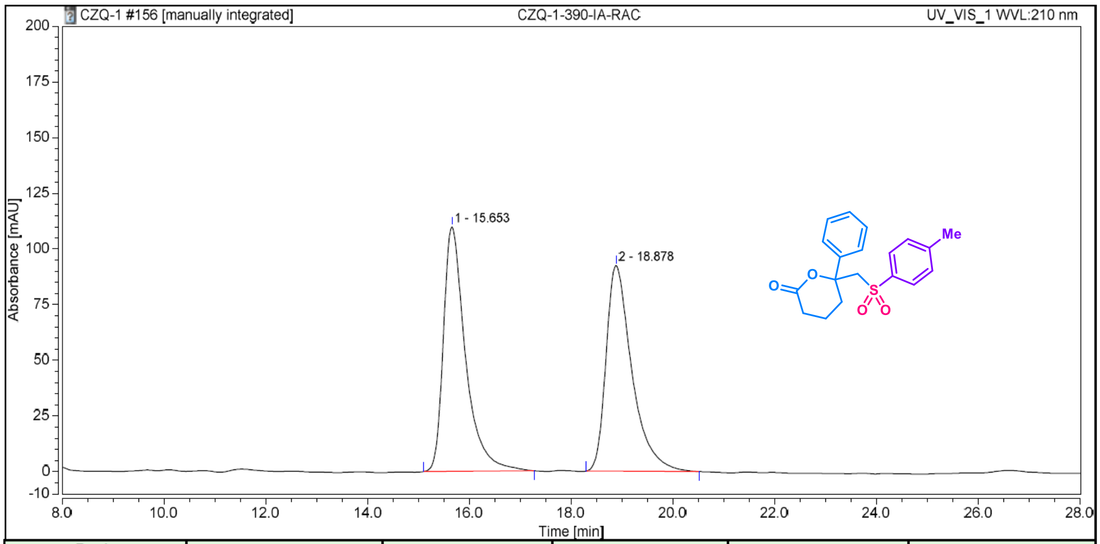

Peak	RetTime min	Area mAU*min	Height mAU	Area %	Height %
1	15.653	55.181	109.892	50.35	54.32
2	18.878	54.422	92.427	49.65	45.68
**Total:**		**109.602**	**202.318**	**100.00**	**100.00**

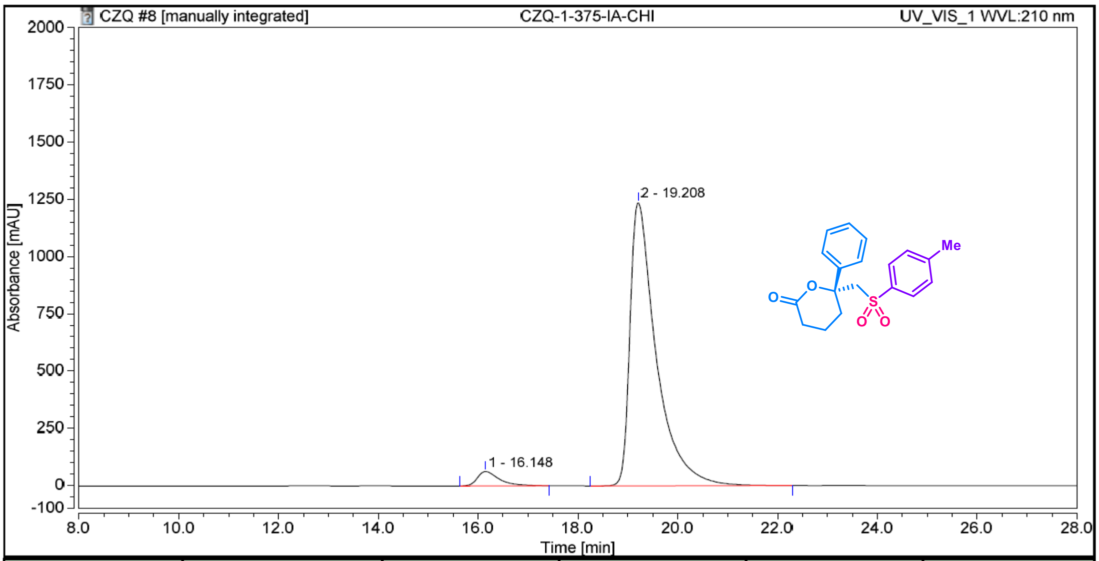

Peak	RetTime min	Area mAU*min	Height mAU	Area %	Height %
1	16.148	31.963	63.267	4.07	4.86
2	19.208	753.036	1238.949	95.93	95.14
**Total:**		**784.998**	**1302.217**	**100.00**	**100.00**

(*R*)-6-(((3-chlorophenyl)sulfonyl)methyl)-6-(m-tolyl)tetrahydro-2*H*-pyran-2-one

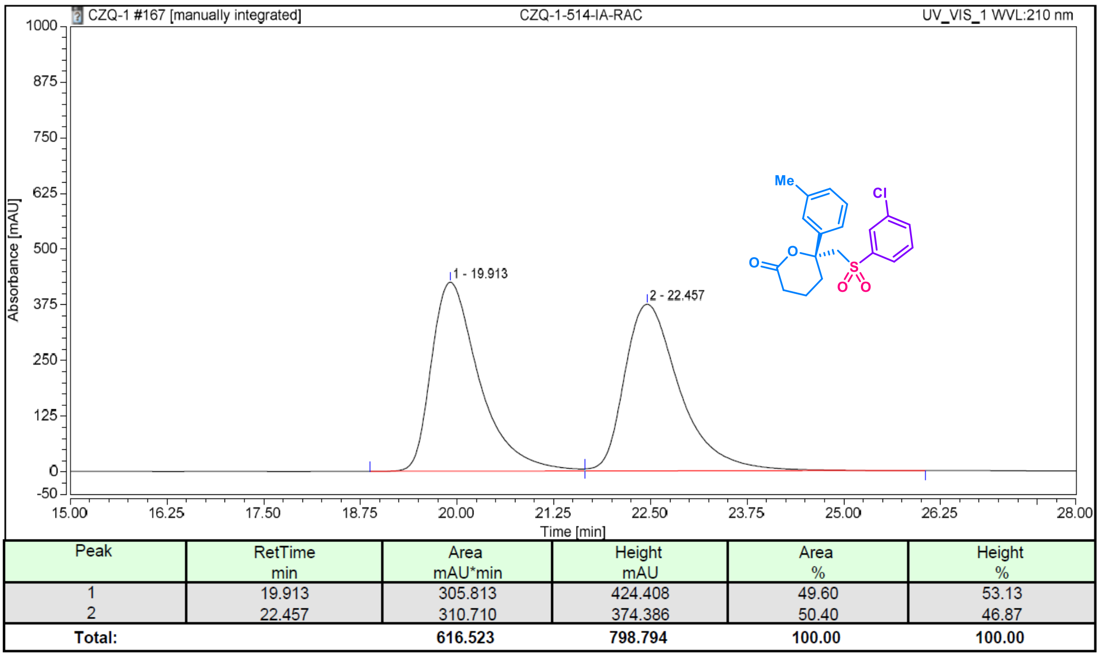

Peak	RetTime min	Area mAU*min	Height mAU	Area %	Height %
1	19.913	305.813	424.408	49.60	53.13
2	22.457	310.710	374.386	50.40	46.87
**Total:**		**616.523**	**798.794**	**100.00**	**100.00**

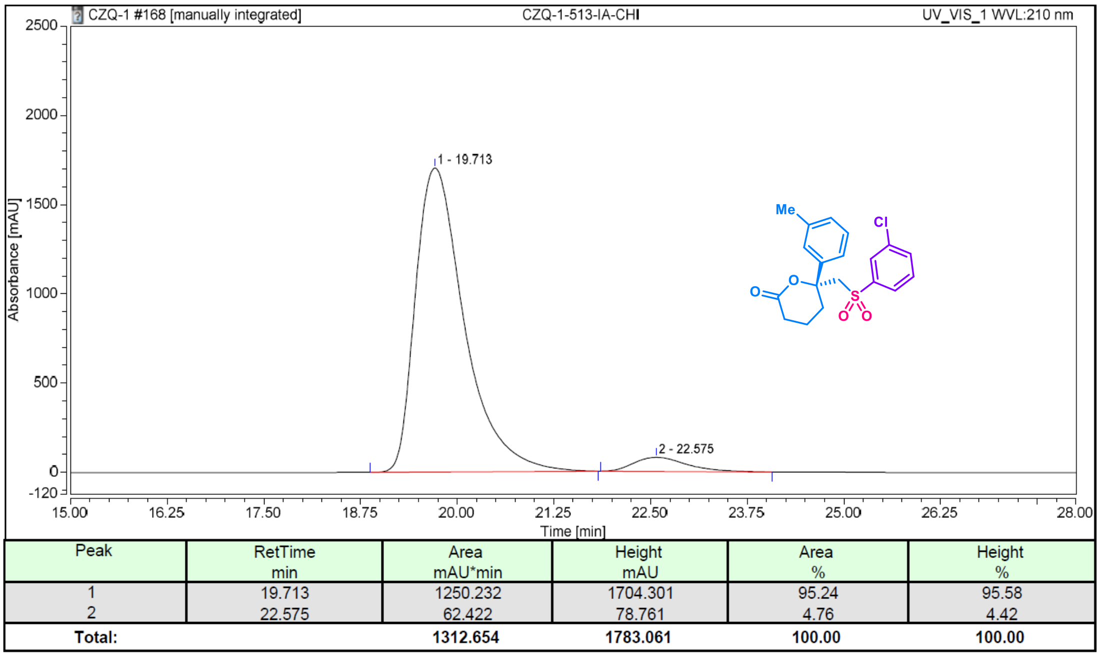

Peak	RetTime min	Area mAU*min	Height mAU	Area %	Height %
1	19.713	1250.232	1704.301	95.24	95.58
2	22.575	62.422	78.761	4.76	4.42
**Total:**		**1312.654**	**1783.061**	**100.00**	**100.00**

(*R*)-6-(((3-chlorophenyl)sulfonyl)methyl)-6-(3-methoxyphenyl)tetrahydro-2*H*-pyran-2-one

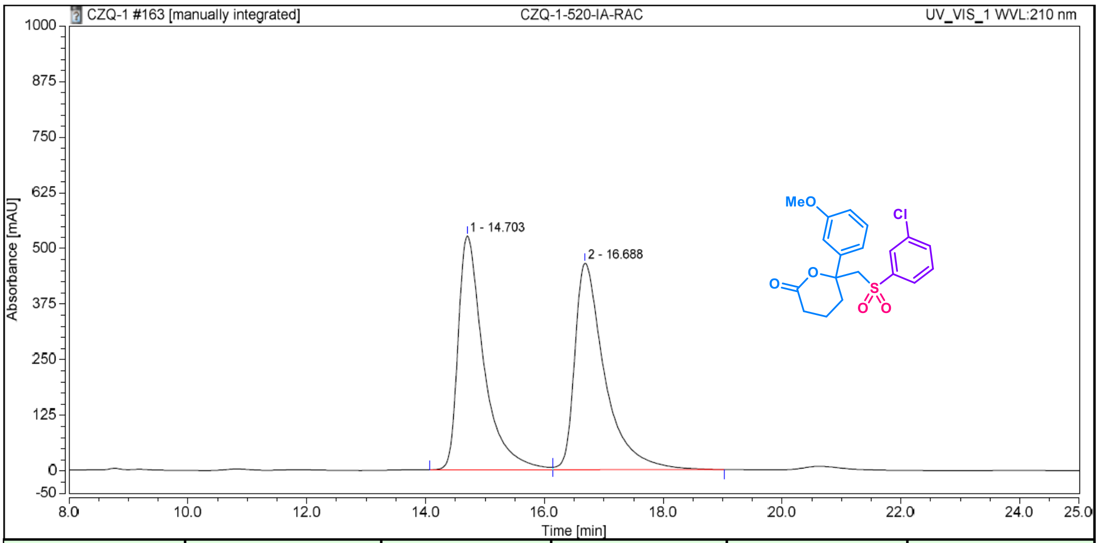

Peak	RetTime min	Area mAU*min	Height mAU	Area %	Height %
1	14.703	261.919	525.707	49.11	53.10
2	16.688	271.463	464.407	50.89	46.90
**Total:**		**533.382**	**990.114**	**100.00**	**100.00**

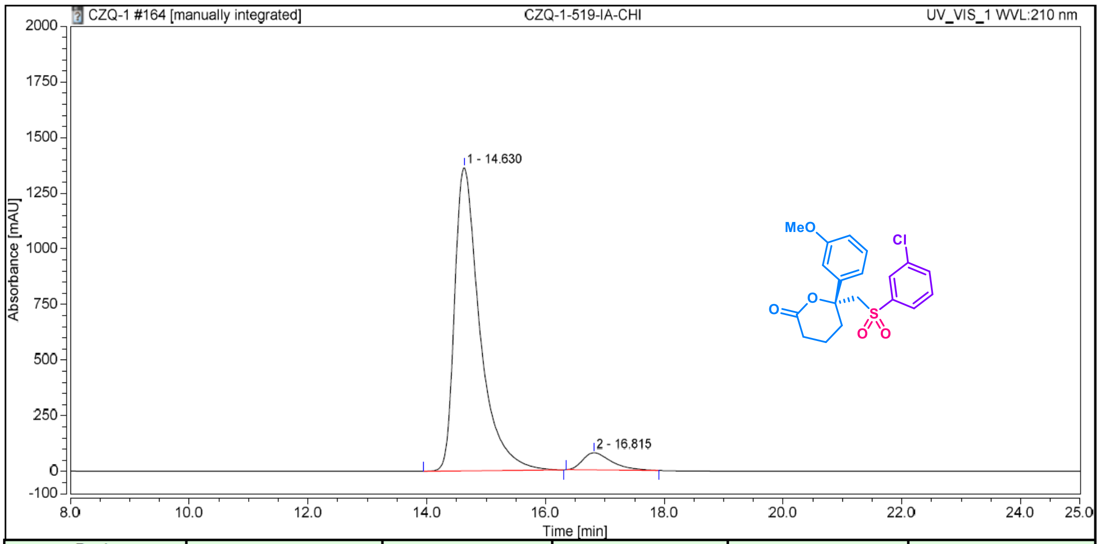

Peak	RetTime min	Area mAU*min	Height mAU	Area %	Height %
1	14.630	669.044	1363.748	93.92	94.66
2	16.815	43.281	76.947	6.08	5.34
**Total:**		**712.325**	**1440.695**	**100.00**	**100.00**

(*R*)-6-(([1,1'-biphenyl]-4-ylsulfonyl)methyl)-6-phenyltetrahydro-2*H*-pyran-2-one

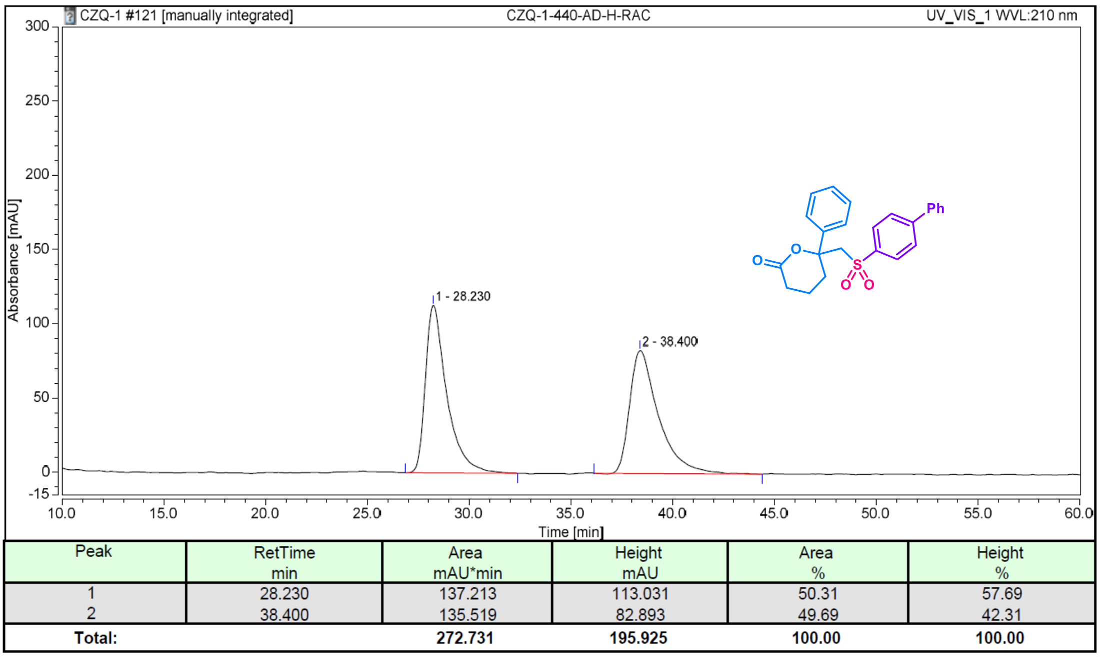

Peak	RetTime min	Area mAU*min	Height mAU	Area %	Height %
1	28.230	137.213	113.031	50.31	57.69
2	38.400	135.519	82.893	49.69	42.31
**Total:**		**272.731**	**195.925**	**100.00**	**100.00**

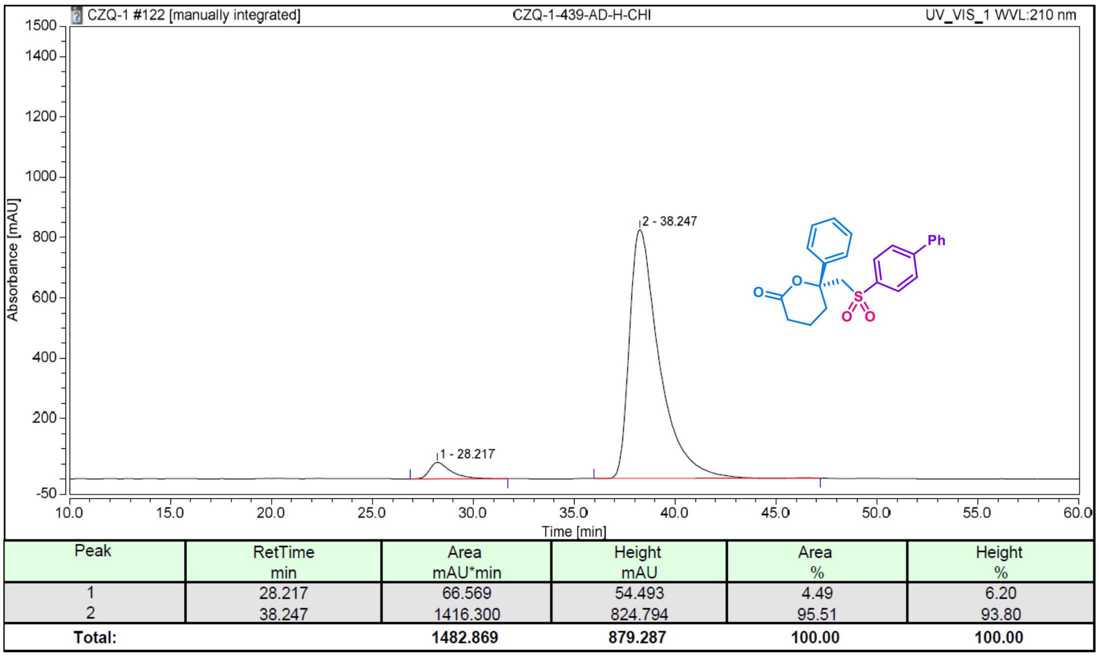

Peak	RetTime min	Area mAU*min	Height mAU	Area %	Height %
1	28.217	66.569	54.493	4.49	6.20
2	38.247	1416.300	824.794	95.51	93.80
**Total:**		**1482.869**	**879.287**	**100.00**	**100.00**

(*R*)-6-(((4-(tert-butyl)phenyl)sulfonyl)methyl)-6-phenyltetrahydro-2*H*-pyran-2-one

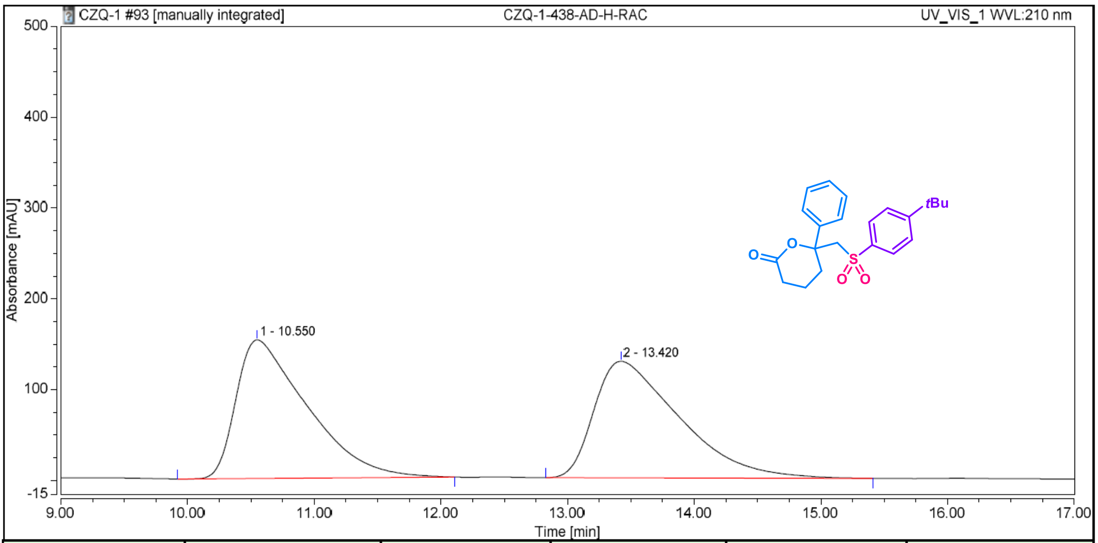

Peak	RetTime min	Area mAU*min	Height mAU	Area %	Height %
1	10.550	99.005	152.539	49.76	54.30
2	13.420	99.946	128.369	50.24	45.70
**Total:**		**198.951**	**280.908**	**100.00**	**100.00**

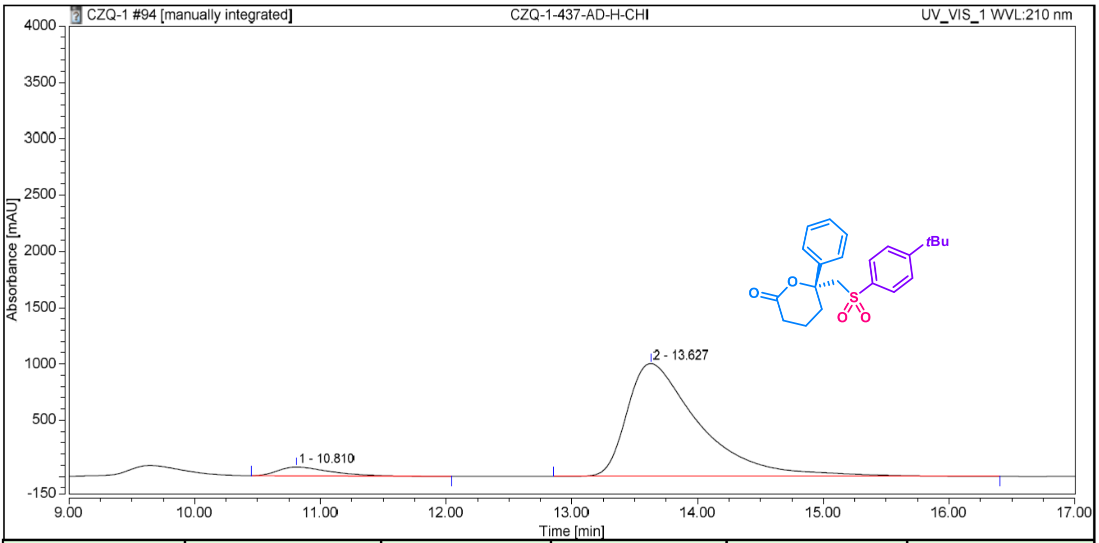

Peak	RetTime min	Area mAU*min	Height mAU	Area %	Height %
1	10.810	36.934	78.416	5.24	7.26
2	13.627	668.234	1002.355	94.76	92.74
**Total:**		**705.169**	**1080.770**	**100.00**	**100.00**

(*R*)-6-(((2-chlorophenyl)sulfonyl)methyl)-6-phenyltetrahydro-2*H*-pyran-2-one

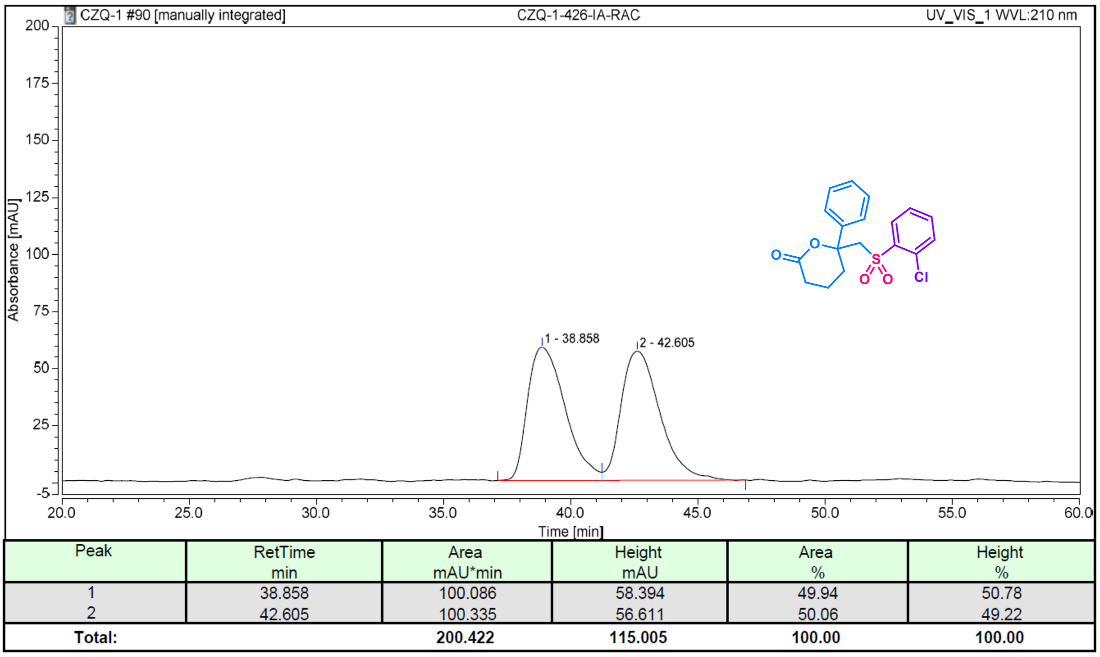

Peak	RetTime min	Area mAU*min	Height mAU	Area %	Height %
1	38.858	100.086	58.394	49.94	50.78
2	42.605	100.335	56.611	50.06	49.22
**Total:**		**200.422**	**115.005**	**100.00**	**100.00**

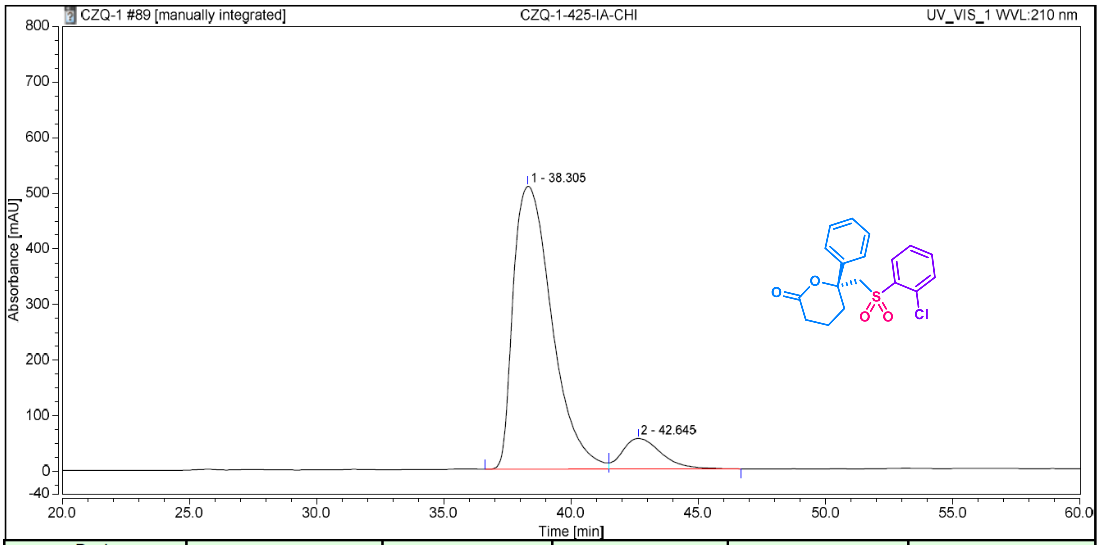

Peak	RetTime min	Area mAU*min	Height mAU	Area %	Height %
1	38.305	897.321	508.859	90.17	90.25
2	42.645	97.807	54.945	9.83	9.75
**Total:**		**995.128**	**563.804**	**100.00**	**100.00**

(*R*)-6-(((3-chlorophenyl)sulfonyl)methyl)-6-phenyltetrahydro-2*H*-pyran-2-one

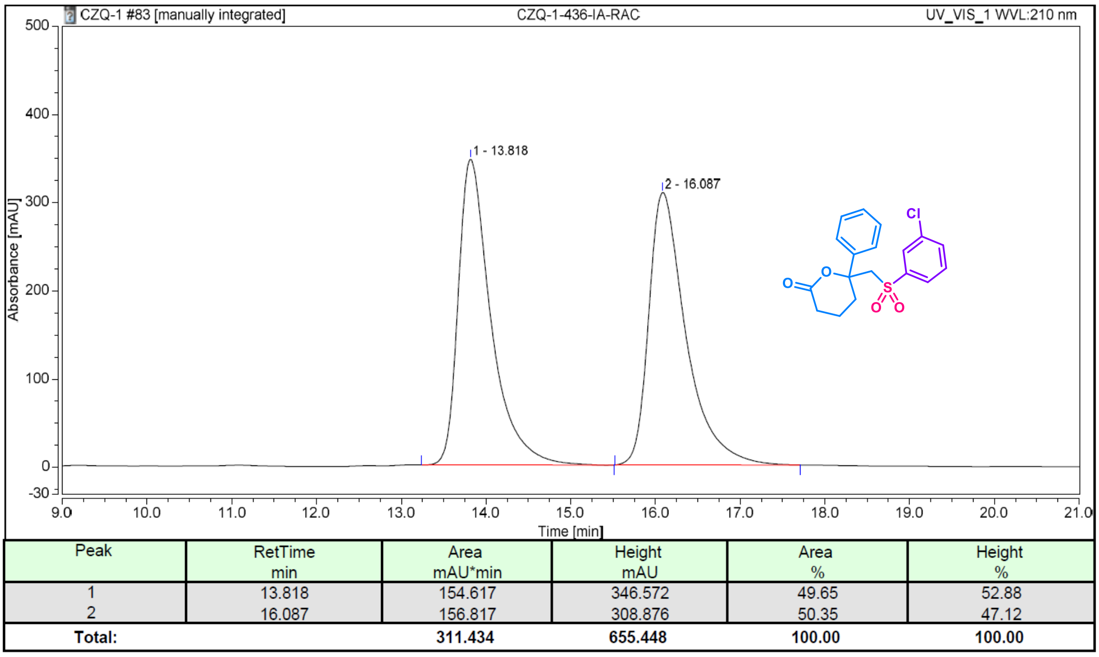

Peak	RetTime min	Area mAU*min	Height mAU	Area %	Height %
1	13.818	154.617	346.572	49.65	52.88
2	16.087	156.817	308.876	50.35	47.12
**Total:**		**311.434**	**655.448**	**100.00**	**100.00**

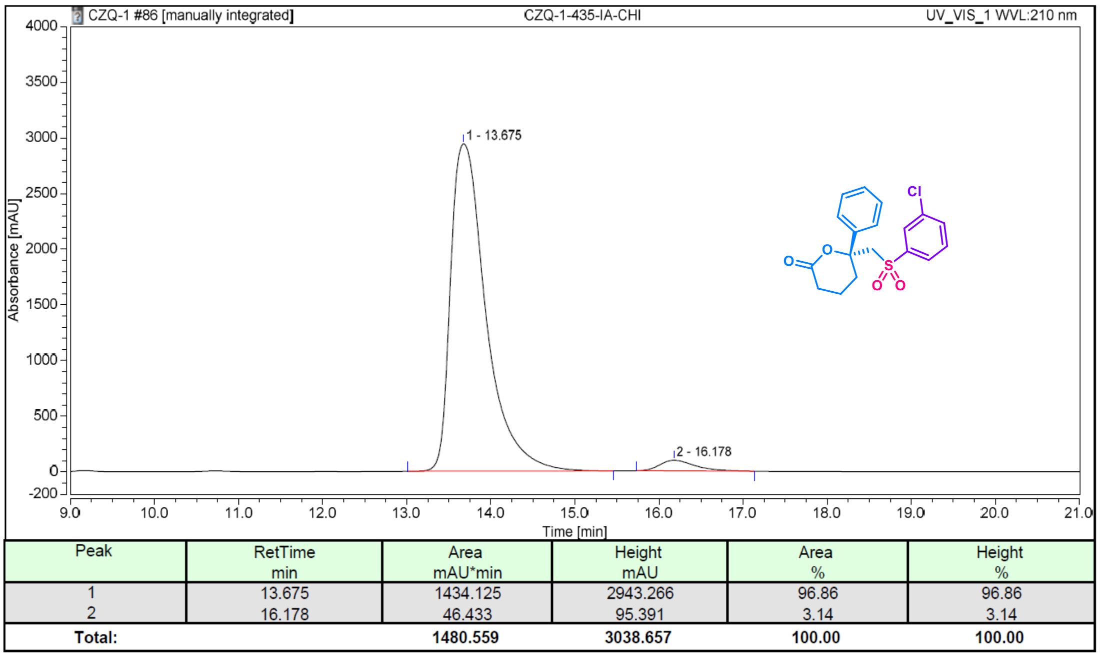

Peak	RetTime min	Area mAU*min	Height mAU	Area %	Height %
1	13.675	1434.125	2943.266	96.86	96.86
2	16.178	46.433	95.391	3.14	3.14
**Total:**		**1480.559**	**3038.657**	**100.00**	**100.00**

(*R*)-6-(((4-chlorophenyl)sulfonyl)methyl)-6-phenyltetrahydro-2*H*-pyran-2-one

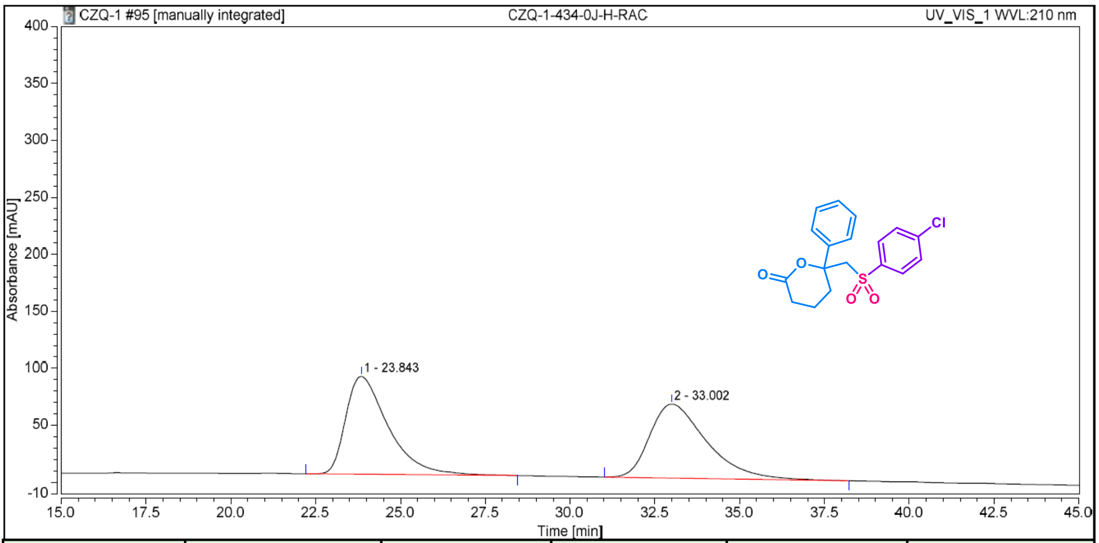

Peak	RetTime min	Area mAU*min	Height mAU	Area %	Height %
1	23.843	124.716	85.859	49.75	56.93
2	33.002	125.957	64.952	50.25	43.07
**Total:**		**250.673**	**150.811**	**100.00**	**100.00**

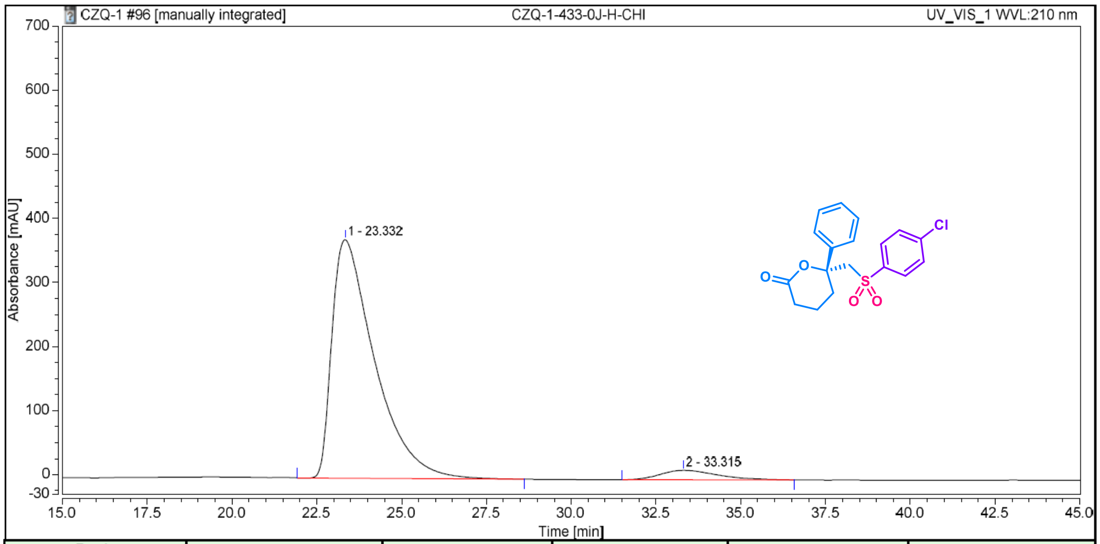

Peak	RetTime min	Area mAU*min	Height mAU	Area %	Height %
1	23.332	542.261	372.165	95.01	96.19
2	33.315	28.463	14.723	4.99	3.81
**Total:**		**570.724**	**386.888**	**100.00**	**100.00**

(*R*)-6-phenyl-6-(((4-(trifluoromethyl)phenyl)sulfonyl)methyl)tetrahydro-2*H*-pyran-2-one

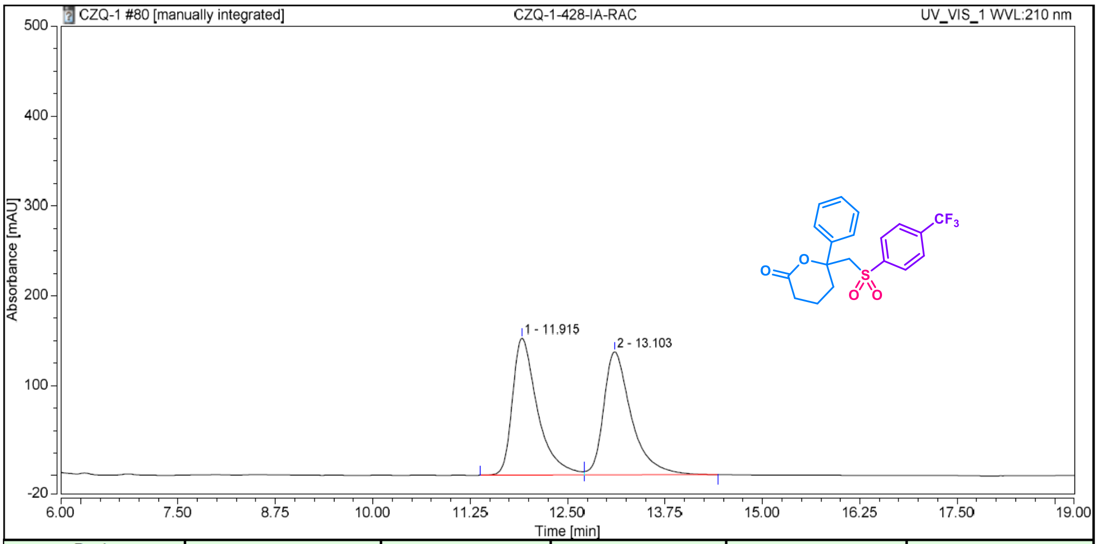

Peak	RetTime min	Area mAU*min	Height mAU	Area %	Height %
1	11.915	57.036	152.481	50.20	52.66
2	13.103	56.571	137.052	49.80	47.34
**Total:**		**113.608**	**289.532**	**100.00**	**100.00**

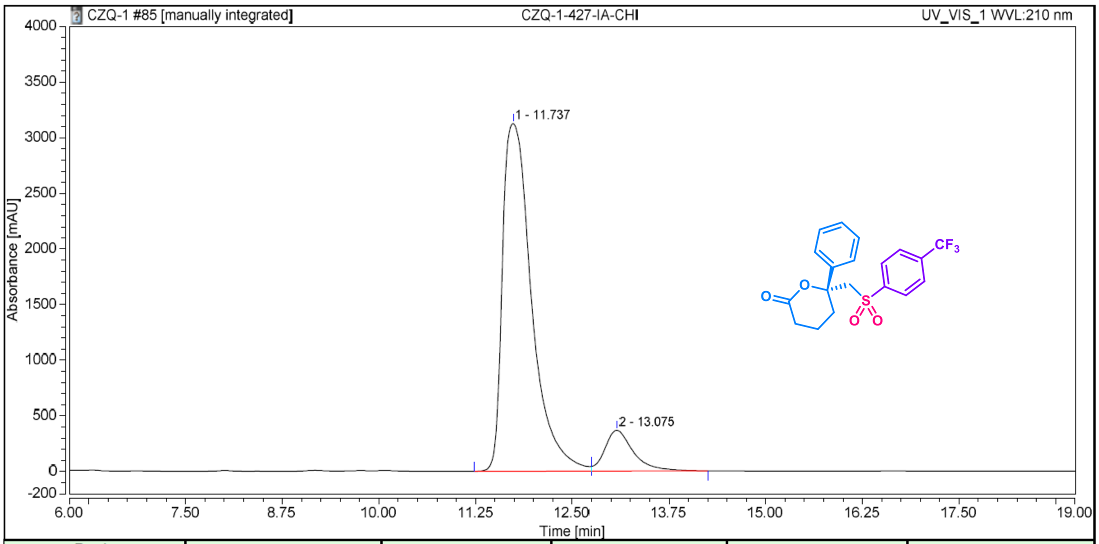

Peak	RetTime min	Area mAU*min	Height mAU	Area %	Height %
1	11.737	1364.065	3127.503	89.76	89.52
2	13.075	155.567	366.166	10.24	10.48
**Total:**		**1519.633**	**3493.668**	**100.00**	**100.00**

(*R*)-5-((morpholinosulfonyl)methyl)-5-(*p*-tolyl)dihydrofuran-2(3*H*)-one

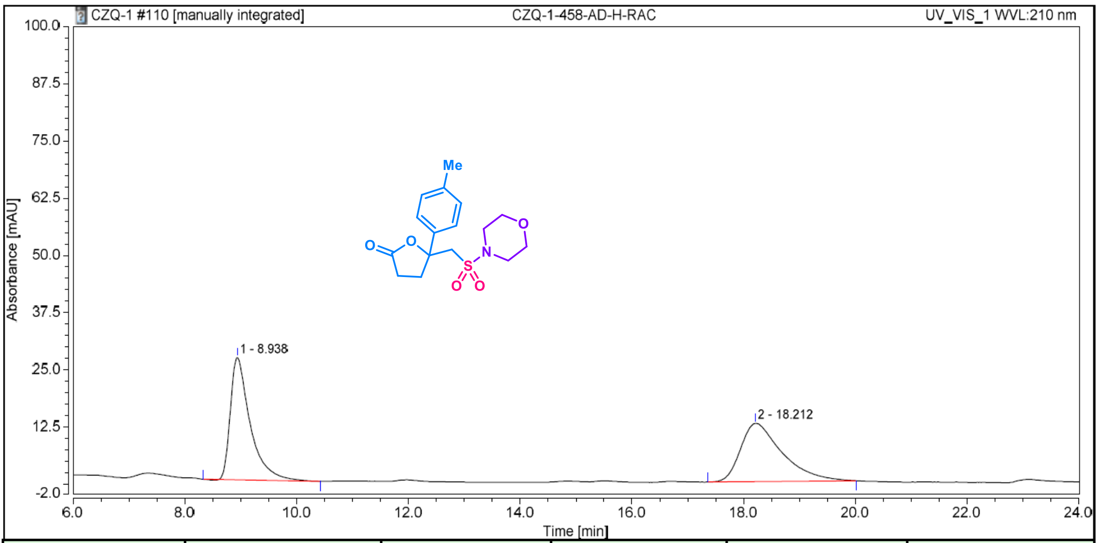

Peak	RetTime min	Area mAU*min	Height mAU	Area %	Height %
1	8.938	10.975	26.745	49.97	67.71
2	18.212	10.987	12.752	50.03	32.29
**Total:**		**21.962**	**39.497**	**100.00**	**100.00**

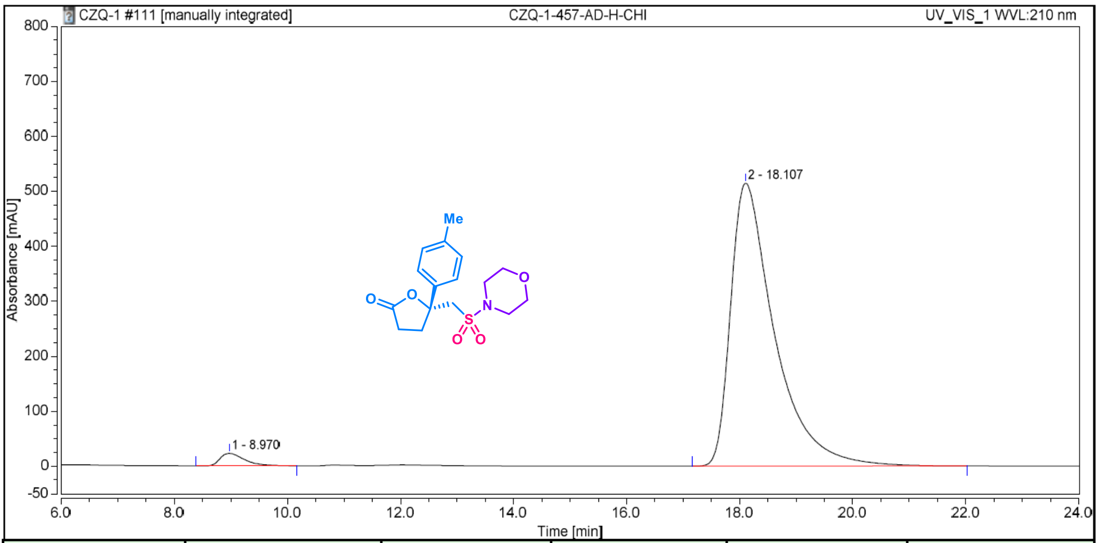

Peak	RetTime min	Area mAU*min	Height mAU	Area %	Height %
1	8.970	11.309	22.566	2.39	4.20
2	18.107	462.244	514.985	97.61	95.80
**Total:**		**473.552**	**537.551**	**100.00**	**100.00**

(*R*)-4-(((5-oxo-2-(p-tolyl)tetrahydrofuran-2-yl)methyl)sulfonyl)butanenitrile

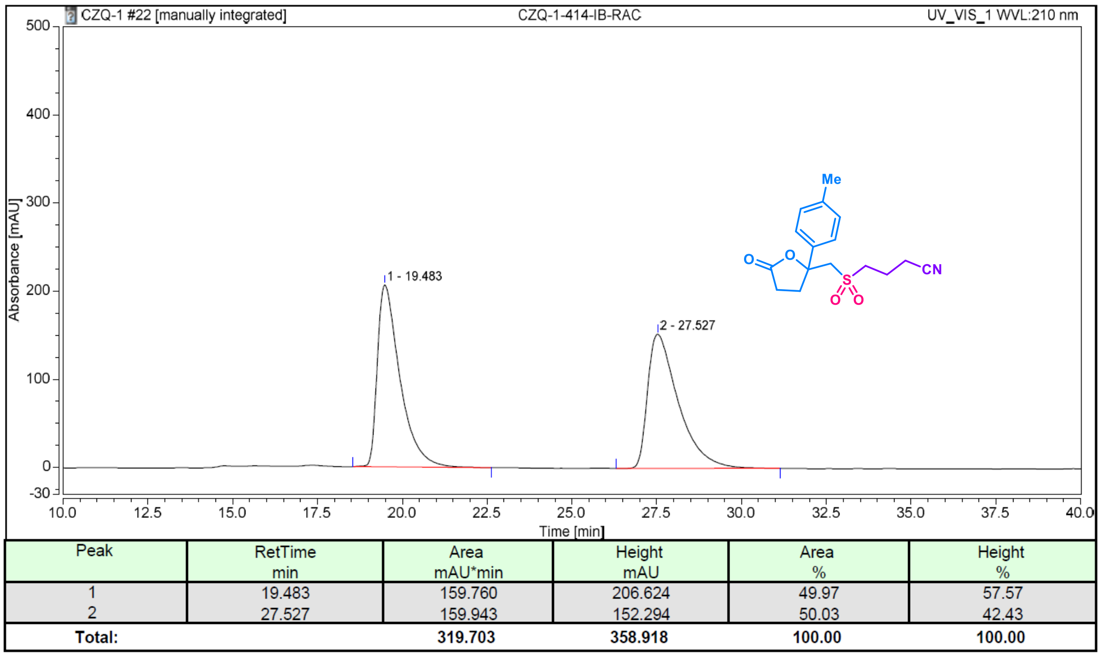

Peak	RetTime min	Area mAU*min	Height mAU	Area %	Height %
1	19.483	159.760	206.624	49.97	57.57
2	27.527	159.943	152.294	50.03	42.43
**Total:**		**319.703**	**358.918**	**100.00**	**100.00**

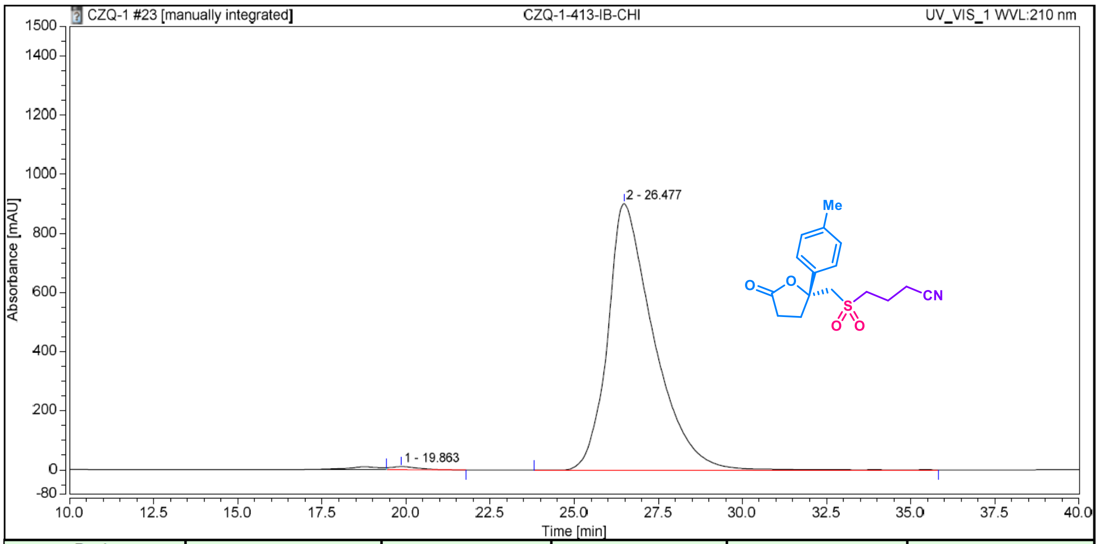

Peak	RetTime min	Area mAU*min	Height mAU	Area %	Height %
1	19.863	10.020	10.630	0.70	1.17
2	26.477	1424.588	900.800	99.30	98.83
**Total:**		**1434.608**	**911.431**	**100.00**	**100.00**

(*R*)-5-(p-tolyl)-5-(tosylmethyl)dihydrofuran-2(3*H*)-one

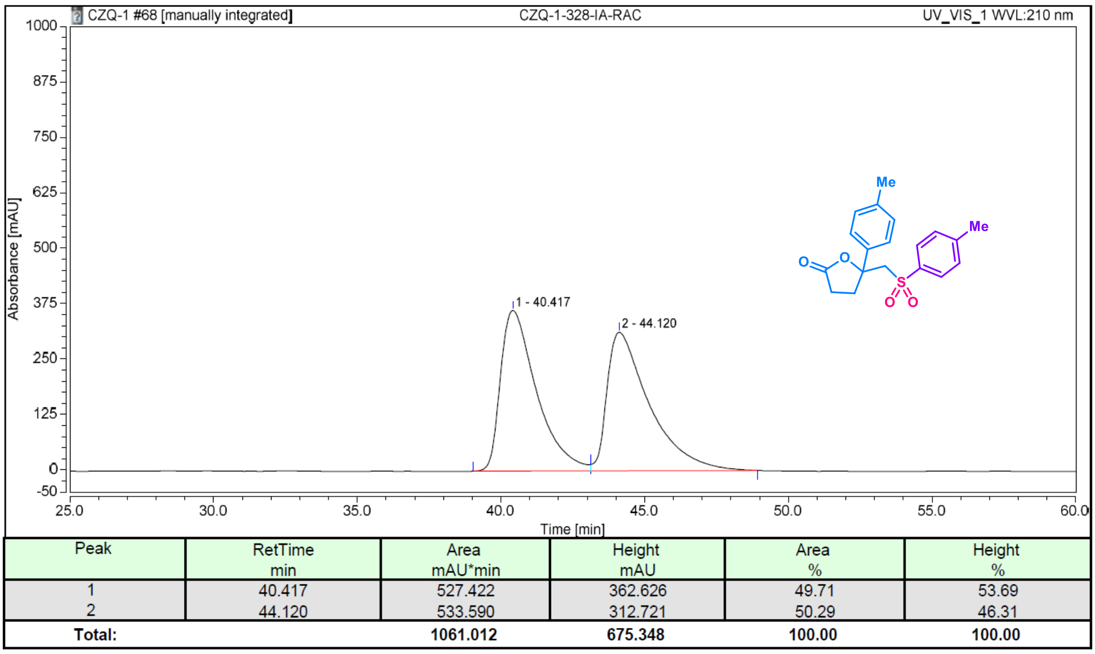

Peak	RetTime min	Area mAU*min	Height mAU	Area %	Height %
1	40.417	527.422	362.626	49.71	53.69
2	44.120	533.590	312.721	50.29	46.31
**Total:**		**1061.012**	**675.348**	**100.00**	**100.00**

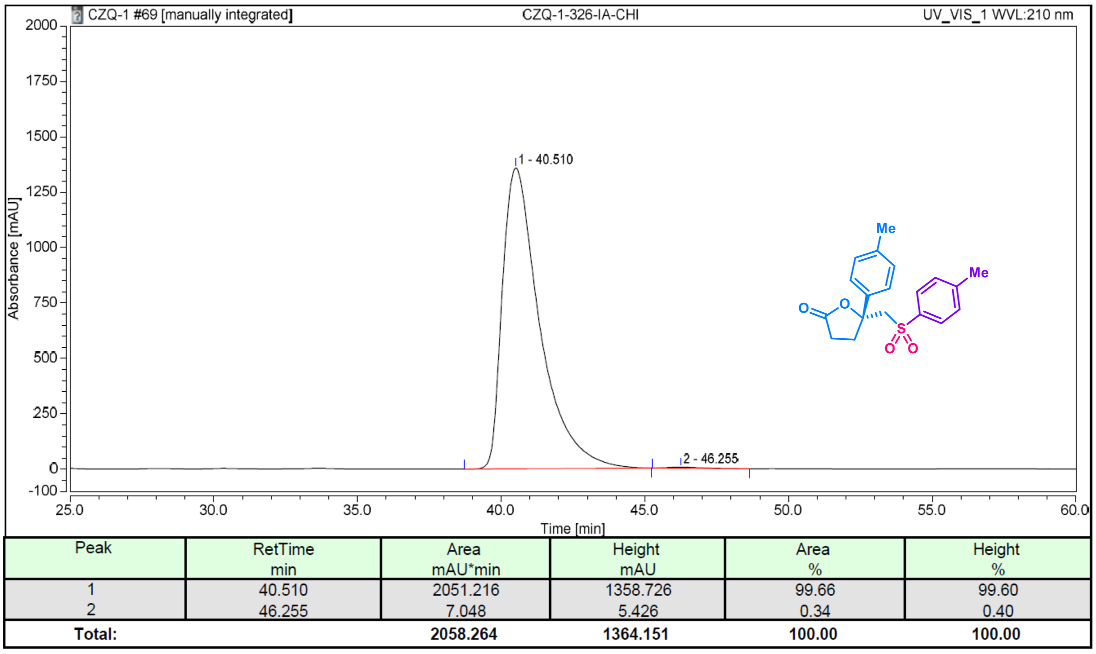

Peak	RetTime min	Area mAU*min	Height mAU	Area %	Height %
1	40.510	2051.216	1358.726	99.66	99.60
2	46.255	7.048	5.426	0.34	0.40
**Total:**		**2058.264**	**1364.151**	**100.00**	**100.00**

***tert*-butyl (*R*)-(4-(((5-oxo-2-phenyltetrahydrofuran-2-yl)methyl)sulfonyl)butyl) carbamate**

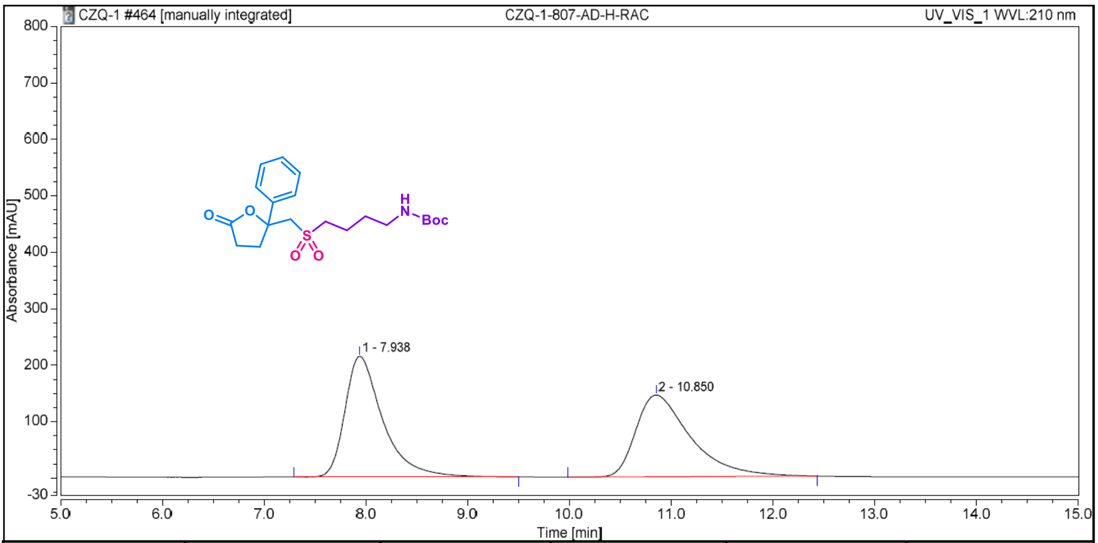

Peak	RetTime min	Area mAU*min	Height mAU	Area %	Height %
1	7.938	91.810	213.692	50.47	59.65
2	10.850	90.083	144.561	49.53	40.35
**Total:**		**181.894**	**358.253**	**100.00**	**100.00**

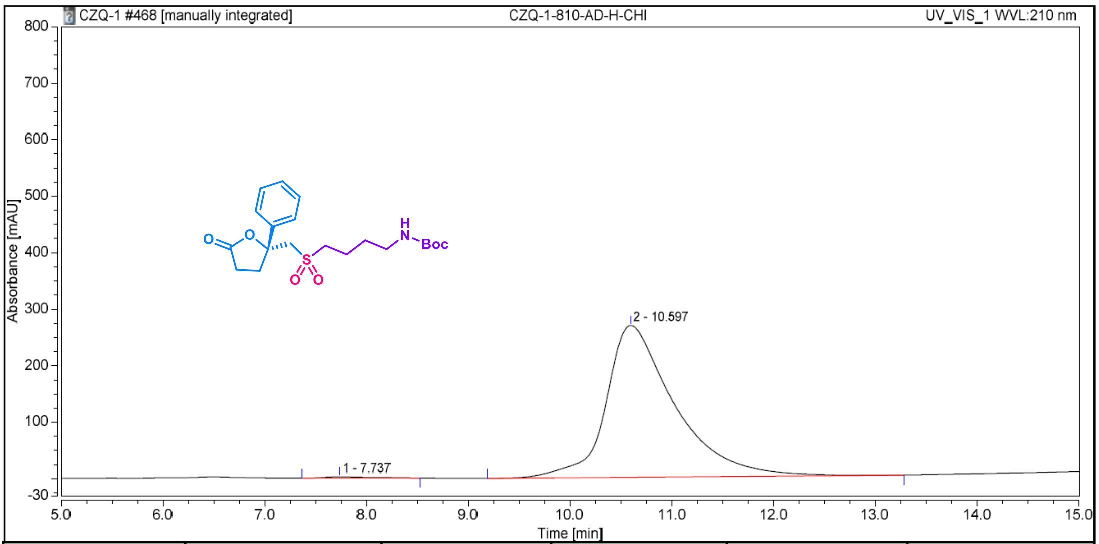

Peak	RetTime min	Area mAU*min	Height mAU	Area %	Height %
1	7.737	1.092	2.105	0.52	0.78
2	10.597	209.959	268.639	99.48	99.22
**Total:**		**211.051**	**270.744**	**100.00**	**100.00**

(*R*)-5-phenyl-5-(tosylmethyl)dihydrofuran-2(3*H*)-thione

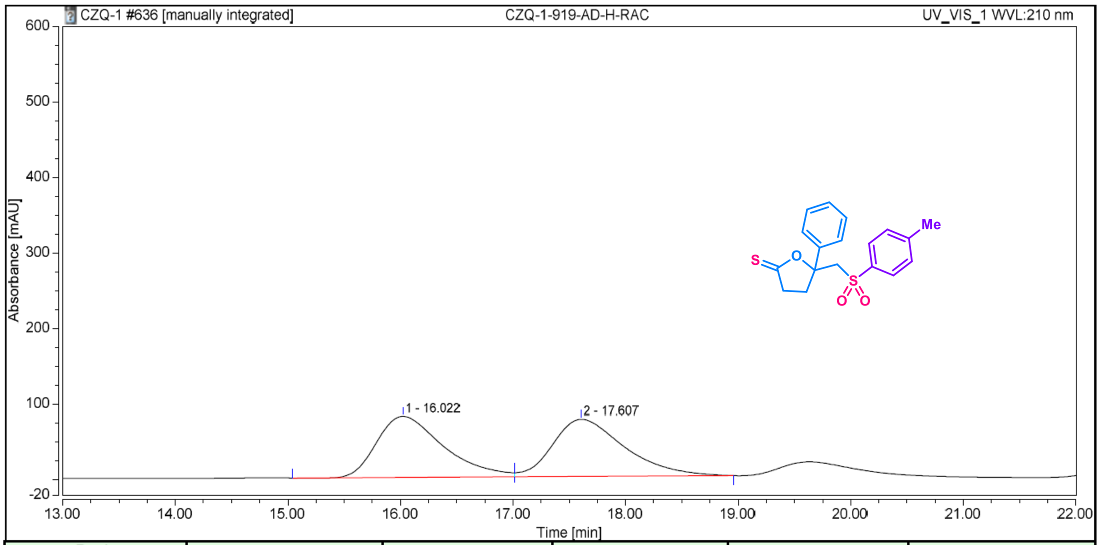

Peak	RetTime min	Area mAU*min	Height mAU	Area %	Height %
1	16.022	55.341	80.736	49.70	51.66
2	17.607	56.009	75.536	50.30	48.34
**Total:**		**111.350**	**156.272**	**100.00**	**100.00**

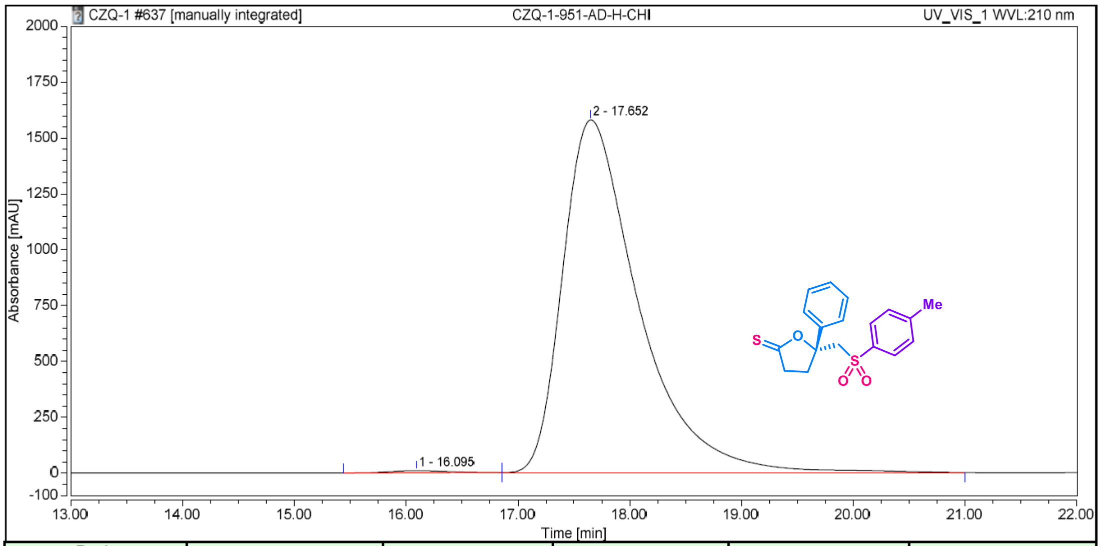

Peak	RetTime min	Area mAU*min	Height mAU	Area %	Height %
1	16.095	5.452	9.178	0.44	0.58
2	17.652	1243.057	1579.783	99.56	99.42
**Total:**		**1248.509**	**1588.961**	**100.00**	**100.00**

(5*R*)-5-phenyl-5-(tosylmethyl)tetrahydrofuran-2-ol

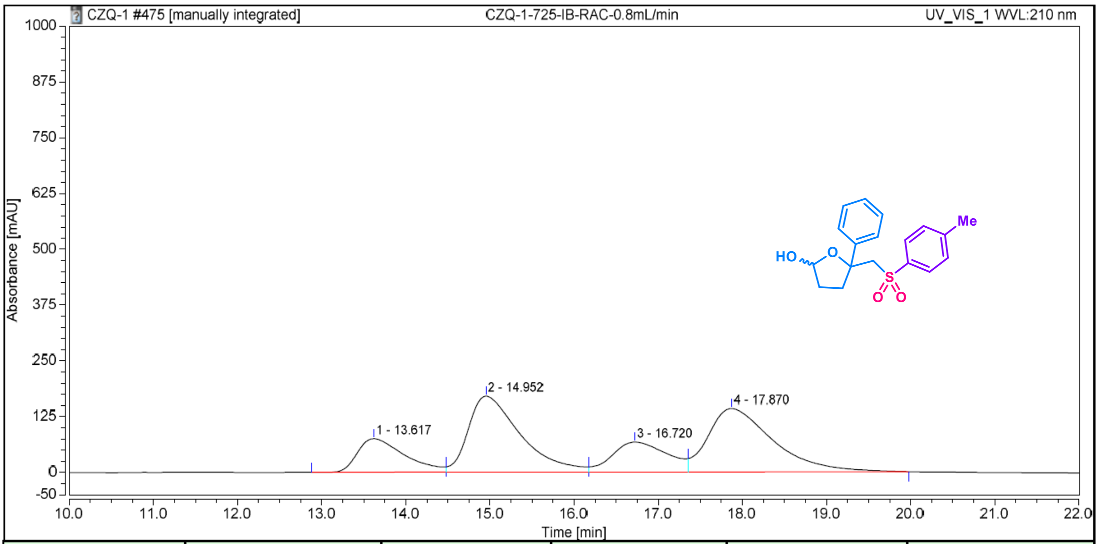

Peak	RetTime min	Area mAU*min	Height mAU	Area %	Height %
1	13.617	48.478	74.943	13.95	16.51
2	14.952	122.037	170.374	35.11	37.53
3	16.720	50.601	66.885	14.56	14.73
4	17.870	126.499	141.750	36.39	31.23
**Total:**		**347.615**	**453.952**	**100.00**	**100.00**

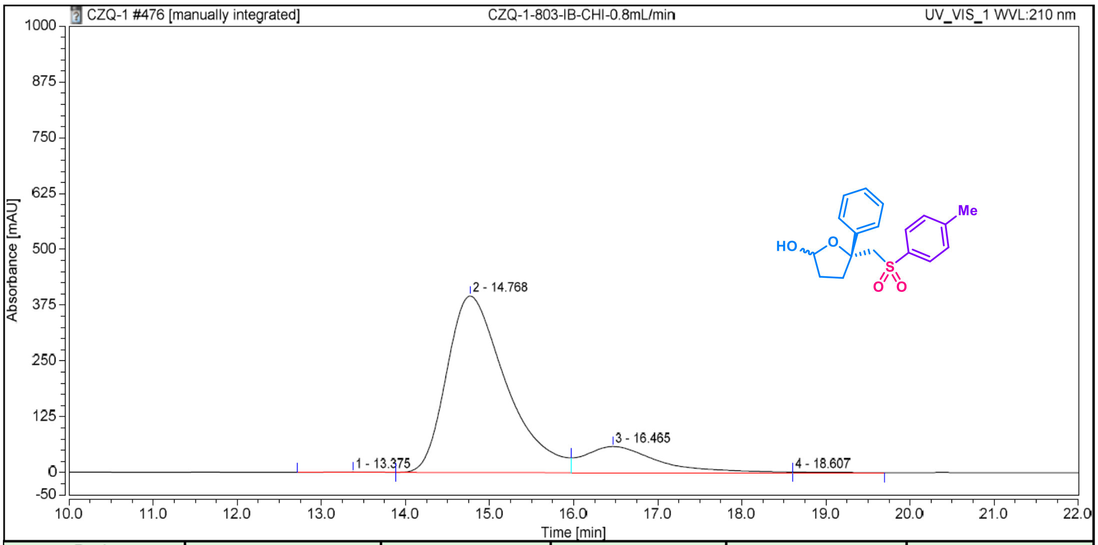

Peak	RetTime min	Area mAU*min	Height mAU	Area %	Height %
1	13.375	0.700	1.081	0.18	0.24
2	14.768	324.708	395.593	84.17	86.62
3	16.465	59.628	58.629	15.46	12.84
4	18.607	0.722	1.411	0.19	0.31
**Total:**		**385.758**	**456.714**	**100.00**	**100.00**

(*R*)-2-phenyl-2-(tosylmethyl)tetrahydrofuran

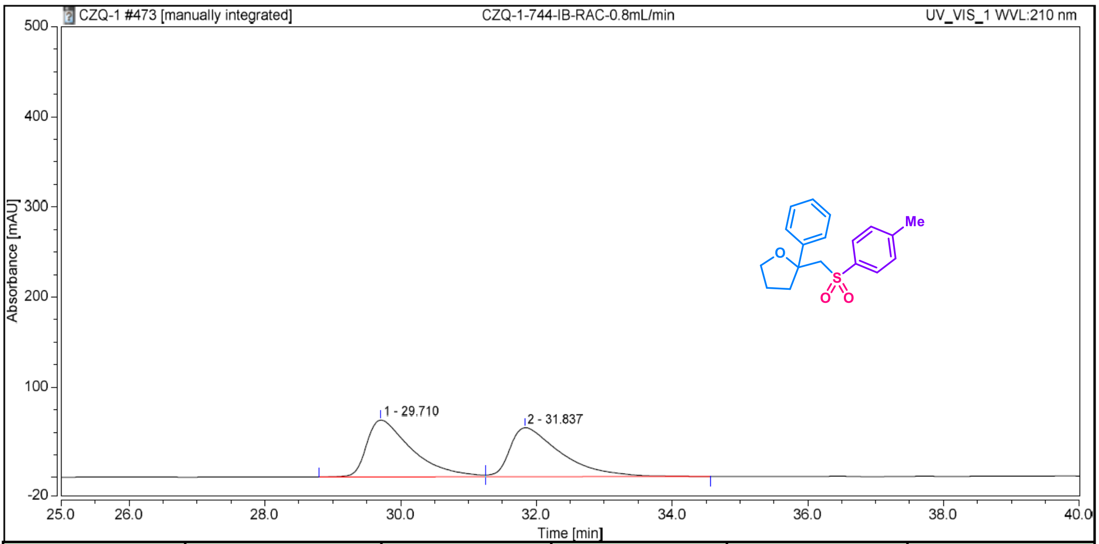

Peak	RetTime min	Area mAU*min	Height mAU	Area %	Height %
1	29.710	48.519	63.014	50.68	53.70
2	31.837	47.208	54.326	49.32	46.30
**Total:**		**95.727**	**117.339**	**100.00**	**100.00**

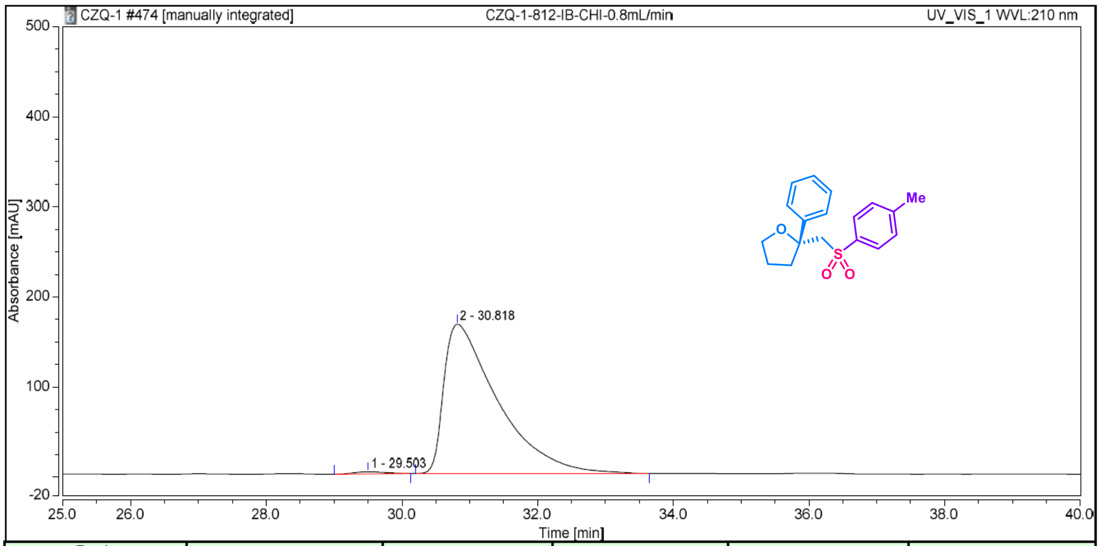

Peak	RetTime min	Area mAU*min	Height mAU	Area %	Height %
1	29.503	1.293	2.487	0.84	1.48
2	30.818	152.495	165.581	99.16	98.52
**Total:**		**153.788**	**168.068**	**100.00**	**100.00**

(*R*)-5-phenyl-5-(((4-(1-tosyl-1H-1,2,3-triazol-4-yl)phenyl)sulfonyl)methyl) dihydrofuran-2(3*H*)-one

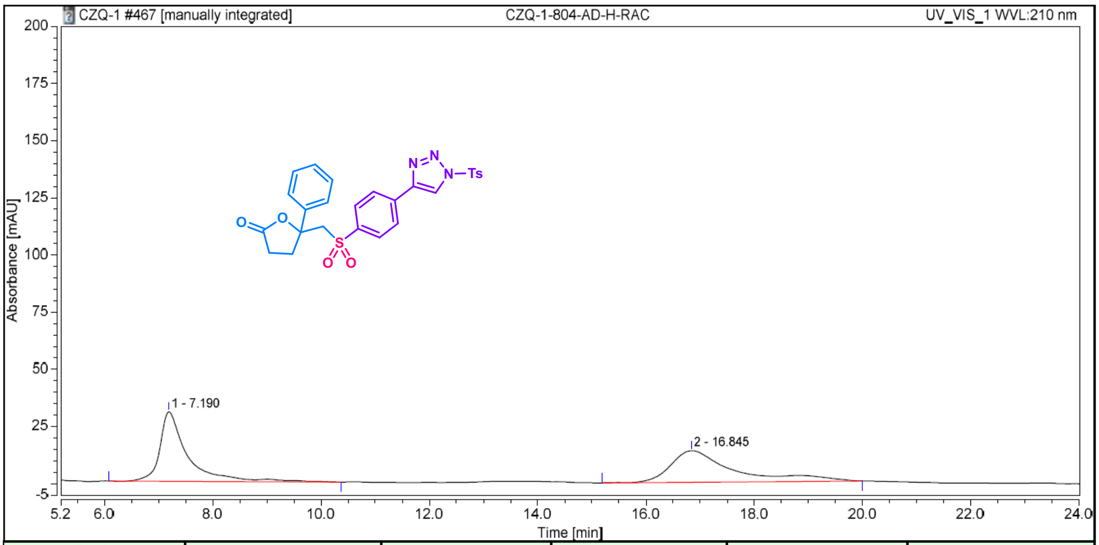

Peak	RetTime min	Area mAU*min	Height mAU	Area %	Height %
1	7.190	18.057	30.445	49.27	68.80
2	16.845	18.594	13.807	50.73	31.20
**Total:**		**36.652**	**44.252**	**100.00**	**100.00**

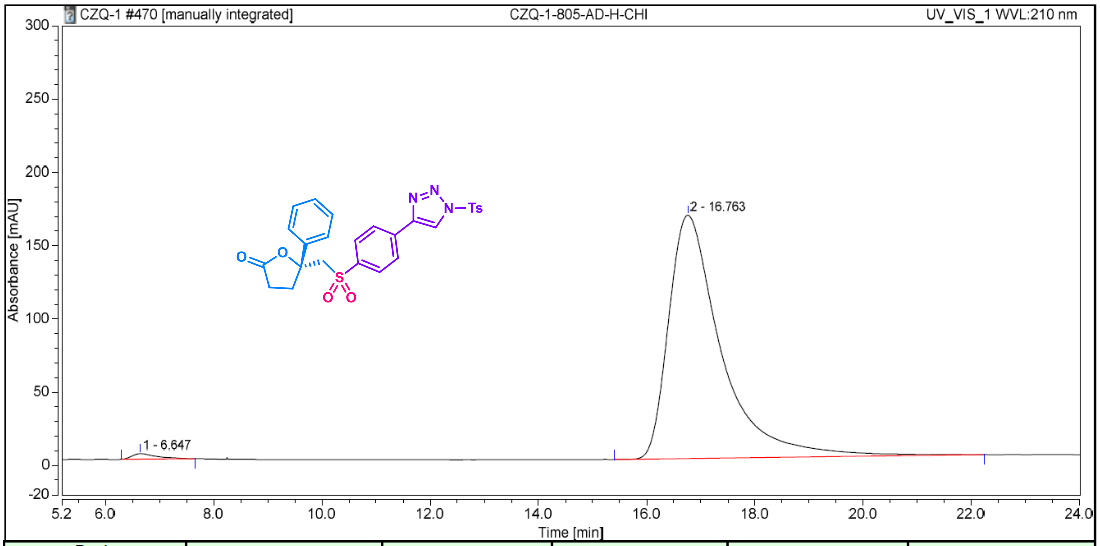

Peak	RetTime min	Area mAU*min	Height mAU	Area %	Height %
1	6.647	1.965	3.676	1.04	2.16
2	16.763	187.000	166.119	98.96	97.84
**Total:**		**188.966**	**169.795**	**100.00**	**100.00**
